# IFIT3 promotes lymph node metastasis by interacting with LASP1 to activate FAK-ERK signaling in esophageal squamous cell carcinoma

**DOI:** 10.1038/s41419-025-08327-z

**Published:** 2025-12-18

**Authors:** Qixiao Cui, Xiaolin Zhou, Xiaojie Chen, Xiaochuan Zhao, Rui Zhang, Jince Wang, Xiaoli Li, Guoli Wang, Bohan Li, Minjing Li, Jiajia Chen, Xihang Gao, Yanbing Shi, Wenting Li, Pan Hong, Defang Li

**Affiliations:** 1 Featured Laboratory for Biosynthesis and Target Discovery of Active Components of Traditional Chinese Medicine, School of Traditional Chinese Medicine, Binzhou Medical University, Yantai, Shandong PR China; 2 Yantai Key Laboratory of Pharmacology of Traditional Chinese Medicine in Tumor Metabolism, School of Traditional Chinese Medicine, Binzhou Medical University, Yantai, Shandong PR China; 3 The Second School of Clinical Medicine of Binzhou Medical University, Yantai, Shandong PR China; 4 The First School of Clinical Medicine of Binzhou Medical University, Yantai, Shandong PR China

**Keywords:** Tumour biomarkers, Cell invasion

## Abstract

Lymph node metastasis (LNM) is an important cause of poor prognosis in patients with esophageal squamous cell carcinoma (ESCC). However, the mechanism of LNM in ESCC has not been elucidated. Here, we identified interferon-induced proteins with tetratricopeptide repeats 3 (IFIT3) highly expressed in ESCC, especially in LNM tissues. IFIT3 overexpression promoted ESCC cell metastasis in vitro and in vivo. Mechanistically, IFIT3 interacted with LIM and SH3 protein 1 (LASP1) and facilitated the localization of LASP1 to the cell edge, promoting the interaction between LASP1 and Talin1 and the binding of Talin1 to integrin, ultimately activating the FAK-ERK signaling pathway. Clinically, IFIT3 and LASP1 were upregulated in ESCC and LNM tissues and associated with poor prognosis. Moreover, patients with high expression of both IFIT3 and LASP1 have a poorer prognosis. In conclusion, IFIT3 promotes LNM in ESCC through the LASP1/FAK/ERK axis, and IFIT3 is a potential therapeutic target for LNM in ESCC.

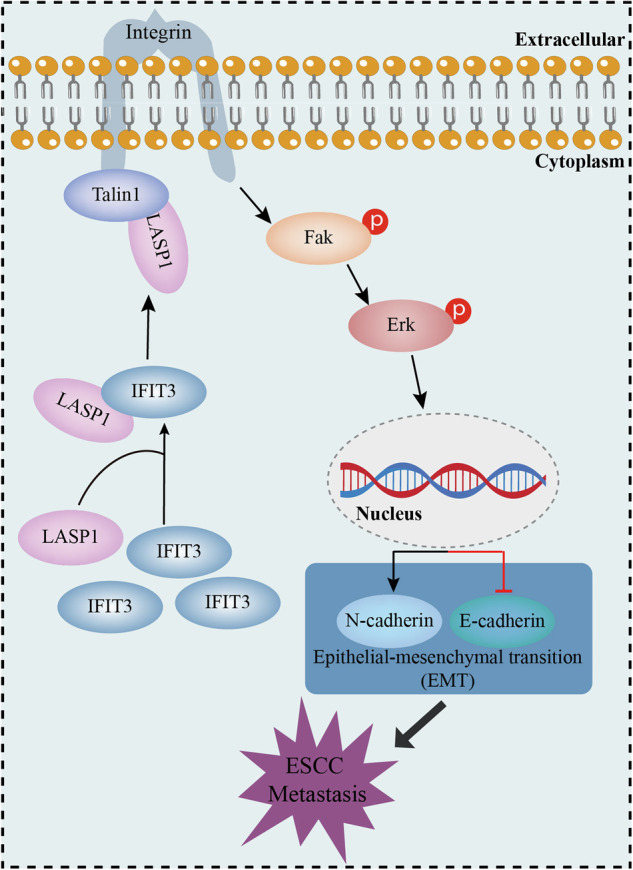

## Introduction

Esophageal cancer (ESCA) is the seventh most diagnosed cancer worldwide and the sixth leading cause of cancer-related death [1]. Chinese cases account for over 50% of global ESCA cases, and esophageal squamous cell carcinoma (ESCC) is the predominant histological subtype [2]. Metastasis, especially lymph node metastasis (LNM), is one of the main reasons for poor prognosis among ESCA patients. Whether LNM occurs and the number and location of LNMs are important factors influencing the ESCA patient prognosis [3]. The LNM rate of ESCA is high due to the complex lymphatic drainage system [4, 5]. Although many functional genes have been confirmed to be involved in LNM [6–8], the mechanism underlying LNM in ESCC remains poorly understood. Therefore, there is an urgent need to identify effective biomarkers and better understand the molecular mechanism of LNM to improve the survival rate of ESCC patients.

Human interferon-induced proteins with tetratricopeptide repeats (IFITs) are a family of interferon-induced proteins [9]. The IFIT family consists of IFIT1, IFIT2, IFIT3, and IFIT5. IFITs show no or low expression in most cell types but are upregulated after viral infection [10]. IFITs consist of multiple tetratricopeptide repeats (TPRs), which are mainly involved in protein‒protein interactions. IFITs containing TPR motifs have been reported to perform various functions, such as regulating translation initiation [11], protein transport [10], protein folding [12], and cell proliferation [13].

Recently, IFITs were reported to be widely expressed in human cancer cells and usually correlated with cancer prognosis. IFIT1 has been found to be highly expressed in many tumors and mediates CXCL11-regulated hepatocellular carcinoma metastasis [14]. The IFIT2 gene is mutated, deleted, or downregulated in various tumors and is associated with tumor chemotherapy resistance and metastasis [15]. IFIT5 can induce bladder cancer cell metastasis and is negatively associated with bladder cancer [16]. Moreover, IFIT3 has been reported to promote drug resistance in pancreatic ductal adenocarcinoma [17]. Notably, a recent study revealed that IFIT3 expression is high in oral squamous cell carcinoma (OSCC) patients and positively correlated with LNM [18]. However, the relationship between IFITs and ESCC, especially LNMs of ESCC, has not been reported, and the mechanism by which IFITs promote the LNM of cancer cells remains unclear.

Herein, the association between IFITs and ESCC was first analyzed using registered and publicly available ESCC gene expression profile data. Only IFIT3 expression was found to be upregulated in tumors with LNM versus those without, and high IFIT3 expression was demonstrated to be closely related to poor prognosis. Furthermore, silencing IFIT3 expression inhibits the invasion and migration of ESCC cells in vitro and suppresses the LNM of ESCC cells in vivo. Mechanistically, IFIT3 promotes the movement of LIM and SH3 protein 1 (LASP1) to the cell edge by binding with LASP1 and subsequently leading to ESCC metastasis. This study elucidates a new mechanism underlying ESCC metastasis and provides new therapeutic targets and diagnostic markers for ESCC metastasis.

## Materials and methods

### Cell culture, transfection, and lentiviral shRNA vectors

The human ESCC cell KYSE30 (Cat no. CBP60457) was procured from Cobioer Biosciences Co., Ltd (Nanjing, China), the human ESCC cell KYSE150 (TCHu236) and the SV40-transformed kidney cell line 293T (Cat no. SCSP-502) were procured from the Cell Bank of the Type Culture Collection Committee of the Chinese Academy of Sciences (Shanghai, China), the human ESCC cell KYSE410 (Cat no. CL-0586) was procured from Wuhan Prioella Biotechnology Co., Ltd (Wuhan, China). STR identification and mycoplasma contamination testing had been conducted prior to the commencement of the experiments. The cells were cultured in Roswell Park Memorial Institute (RPMI)-1640 (Cat no. 11875119) or Dulbecco’s modified Eagle medium (DMEM) (Cat no.11965092) (Gibco, Shanghai, China) containing 10% fetal bovine serum (FBS) (Cat no. FSP500, ExCell Bio, Shanghai, China) and 1% penicillin–streptomycin liquid (100×, Cat no. P1400, Solarbio, Beijing, China) in 37 °C with 5% CO_2_. The cells were passaged 10 times. ESCC cell lines with IFIT3 and LASP1 overexpressed or knocked down were established using the third-generation lentivirus packaging system pMDLg/pRRE (Addgene, Cat no. #12251), pRSV-Rev (Addgene, Cat no. #12253), and pMD2. G (Addgene, Cat no. #12259) and maintained using puromycin or geneticin (Cat no. ST551 and ST081, Beyotime, Shanghai, China). The shRNAs producing the best knockdown efficiency are listed in Table S1.

### Cell migration and invasion assays

In vitro cell migration assays were performed by using uncoated, 8 μm pore-size Transwell chambers (Cat no. 353097, BD Biosciences). Cells (KYSE410: 1.5 × 10^5^, KYSE150: 4 × 10^5^, KYSE30: 3 × 10^5^) in serum-free medium were seeded into the upper chamber, and a complete growth medium was added to the lower chamber as the chemoattractant. After 24 h, the migrated cells were fixed with methanol and stained with crystal violet. The cells that had not migrated were removed from the upper surface of the filters using cotton swabs. Images of each well were obtained from three different fields in each membrane using an inverted microscope (Leica, Wetzlar, Germany), and the number of migrated cells was counted. The mean of the values from triplicate assays for each experimental condition was calculated. Matrigel (Cat no. 356234, BD Biosciences)-coated chambers were used for the invasion assay following a similar protocol.

### Cell adhesion assay

The 24-well plates were coated with 2 μg/ml fibronectin (Cat no. F8180, Solarbio, Beijing, China) in a 37 °C incubator for 1 h. The plates dried at room temperature for 45 min and blocked with 1% bovine serum protein for 1 h. A total of 1 × 10^4^ cells per well were seeded and incubated in an incubator at 37 °C for 30 min, fixed with 4% paraformaldehyde, and stained with 0.2% crystal violet. Images were obtained using an inverted microscope, and cells were counted using ImageJ software.

### Model of popliteal lymphatic metastasis in vivo

Six-to-eight-week-old BALB/c nude male mice (Gempharmatech, Nanjing, China) were housed in specific pathogen-free (SPF) barrier facilities. 5 × 10^5^ ESCC cells expressing luciferase were injected into the mice’s footpads with 50 μL PBS and an equal volume of Matrigel after overexpression or knockdown of IFIT3. Six weeks after injection, mice were subjected to bioluminescence imaging using a bioluminescence system to evaluate lymphatic metastasis (NightOwl II LB983, Berthold Technologies, Germany). All popliteal lymph nodes were harvested and embedded in paraffin for HE analysis. The lymph node volumes were calculated using the following formula: V = (length × width^2^)/2. All the animal experiments were conducted as per the National Institutes of Health Guide for the Care and Use of Laboratory Animals (NIH Publications No. 8023, revised 1978). The Laboratory Animal Ethics Committee of Binzhou Medical University approved the animal studies. Animals were not randomized in this study due to the fact that the experimental design required direct comparison of genetically modified groups (e.g., IFIT3-overexpressing vs. knockdown). To reduce bias, all animals were housed under identical conditions, and tumor measurements were performed by blinded investigators.

### Western blotting

The proteins were extracted and subsequently quantified using a BCA kit (PC0020, Solarbio, Beijing, China). Thereafter, sodium dodecyl sulfate–polyacrylamide gel electrophoresis (SDS–PAGE) was utilized to separate protein extracts (30–40 µg) before transferring them onto a polyvinylidene difluoride membrane. The membrane was blocked with 5% bovine albumin (BSA) solution at room temperature for 1 h and then incubated with the primary antibody overnight at 4 °C. The primary antibodies used were as follows: anti-β-actin (1:2000, TA-09, Zsbio, Beijing, China), anti-IFIT3 (1:5000, Cat no. 15201-1-AP, Proteintech, Wuhan, China), anti-N-Cadherin (1:1000, Cat no. #13116S, CST, Boston, USA), anti-E-Cadherin (1:1000, Cat no. #3195S, CST, Boston, USA), anti-Fak (1:1000, Cat no. 66258-1-Ig, Proteintech, Wuhan, China), anti-p-Fak-Try397 (1:1000, Cat no. #8556S, CST, Boston, USA), anti-LASP1 (1:1000, Cat no. ab117806, Abcam, Cambridge, UK), anti-Talin1 (1:40,000, Cat no. 14168-1-AP, Proteintech, Wuhan, China), anti-Integrin β1 (1:2000, Cat no. 26918-1-AP, Proteintech, Wuhan, China), anti-Flag (Cat no. M20008, Abmart, Shanghai, China), anti-HA (1:40,000, Cat no. 81290-1-RR, Proteintech, Wuhan, China), Rabbit IgG (Cat no. B30011, Abmart, Shanghai, China), and Mouse IgG (Cat no. B30010, Abmart, Shanghai, China). Subsequently, Tris-buffered saline tween (TBST) buffer was used to wash the membranes, after which they were subjected to incubation for 40 min with secondary antibodies (1:10000, Cat no. ZB-2305/ZB-2301, Zsbio, Beijing, China) at room temperature, followed by rinsing them again using TBST buffer. Thereafter, the membranes were then treated with Supersensitive ECL chemiluminescent substrate (Cat no. BL520A, Biosharp, Hefei, China) for 2 min and photographed using a chemiluminescence image analysis system (5200, Tanon, Shanghai, China).

### Co-immunoprecipitation coupled with mass spectrometry analysis

Lipofectamine™ 3000 (Cat no. L3000015, Thermo Fisher Scientific, Shanghai, China) was utilized for the transfection of the plasmids into cell lines. Following 48 h of transfection, the cells were exposed to an IP lysis buffer for lysis (Cat no. KGB5202, KeyGen Biotech, Nanjing, China). Protein A/G magnetic beads (Cat no. HY-K0202, MCE, New Jersey, USA) were incubated with the primary antibody or control IgG at 4 °C for 2 h. The treated magnetic beads were then incubated with the protein extract for 2 h. Unbound proteins were removed by washing with PBST buffer, and the bound proteins were separated by SDS‒PAGE and identified by MS analysis (SHANGHAI BIOTREE BIOTECH CO, LTD, Shanghai, China).

### Immunohistochemistry (IHC)

Analysis of IFIT3 and LASP1 expression was performed utilizing tissue microarrays (Shanghai Outdo Biotech). The ethics committee of Shanghai Qutdo Biotech Company approved the research protocols. The tests were conducted in adherence to all applicable rules, and signed informed consent was collected from all patients. Two tissue microarrays were used in this study, one of which contained 112 primary ESCC tumors and 68 matched adjacent normal tissues, and the other was a tissue microarray containing 53 primary ESCC tissues, 30 adjacent normal tissues, and 50 metastatic or non-metastatic lymph node tissues. Sections were blocked with 1% BSA in PBST buffer and incubated with IFIT3 (1:20000, Cat no. 15201-1-AP, Proteintech, Wuhan, China) or LASP1 (1:3000, Cat no. ab117806, Abcam, Cambridge, UK) antibodies overnight at 4 °C. After secondary antibody incubation, the sections were stained with 3,3′-diaminobenzidine (DAB) substrate. Scores were based on the % of cells with positive staining for IFIT3 or LASP1 (1 ≤ 25%, 2 = 26–50%, 3 = 51–75%, 4 > 75%) and the intensity of IFIT3 or LASP1 staining in positive areas (0 = no staining, 1 = weak staining, 2 = moderate staining, 3 = strong staining). The final IHC score was calculated by multiplying the percentage score and the intensity score, and categorized into three groups: ≤2 (Weak), >2 and ≤5 (Moderate), and > 5 (Strong).

To assess p-ERK and p-FAK levels in LNM, IHC was performed on metastatic LNM from nude mice using an anti-p-ERK antibody (1:1000, Cat no. 28733-1-AP, Proteintech, Wuhan, China) and an anti-p-FAK antibody (1:500, Cat no. 700255, Invitrogen, CA, USA). For quantification, the percentage of positively stained cells was counted in randomly selected high-power fields per section. A cell was considered positive if showing strong granular cytoplasmic staining. The mean percentage of positive cells across fields was calculated for each sample.

### Immunofluorescence

The ESCC cells were fixed with 4% PFA for 15 min, followed by permeabilizing with 0.1% Triton X-100 for another 15 min and blocking with Immunol Staining Blocking Buffer (Cat no. P0102, Beyotime, Shanghai, China) for an hour at 25 °C room temperature. Thereafter, primary antibodies were used to incubate the samples at 4 °C throughout the night. Finally, the samples were exposed to secondary antibodies that were labeled with fluorescence for 1 h at room temperature. Nuclear DNA staining was performed with DAPI (C1002, Beyotime, Shanghai, China), and confocal microscopy (STELLARIS 5, Leica, Wetzlar, Germany) was utilized to capture the images.

### Reanalysis of TCGA-ESCA and GEO public databases

UCSC Xena (http://xena.ucsc.edu/) was searched to compile TCGA-ESCA data, including patient clinical materials and gene expression data. We downloaded ESCC samples and matched normal sample RNA sequencing data from the GEO database (www.ncbi.nlm.nih.gov/geo) to measure the transcriptional levels of IFIT3 and other genes (GSE118493, *n* = 6; GSE53625, *n* = 358; GSE23400, *n* = 208). The above datasets were reanalyzed to obtain the differences in IFIT or LASP1 expression levels between samples with different ESCA histological subtypes, ESCC and normal tissues, different metastasis statuses, and different pathological stages.

### Label-free quantitative proteomics

To investigate the effect of IFIT3 overexpression, we performed label-free proteomics experiments by transfecting KYSE410 cells with an empty or Flag-tagged IFIT3 plasmid using Lip3000 (Life Technologies). The cells were collected and lysed 48 h following transfection. LC‒MS was then used to identify the peptides produced by protein enzymatic digestion. The mass spectrometry raw file was used to construct the sample-specific protein database. MaxQuant (v1.6.15.0) was used to search the database, and we performed the quality control analysis on the levels of peptides and proteins according to the database search results. To get the relative quantitative value (R), we centrally transformed the LFQ intensity (I) of the protein in various samples according to the corrected results of the original protein intensity value (LFQ intensity) between samples. The following was the equation: Rij = Iij/Mean (Ij) (i for sample, j for protein). Finally, to detect differentially expressed proteins between the control group and the IFIT3 overexpression group, bioinformatics techniques were applied. Gene Ontology-Biological Process (GO-BP) and Kyoto Encyclopedia of Genes and Genomes (KEGG) enrichment analyses of differentially expressed proteins were performed to obtain the signaling pathways regulated by IFIT3.

### Collection of genes correlated with the focal adhesion signaling pathway and intracellular sublocalization analysis

A total of 1257 focal adhesion-related genes were extracted from the GeneCards Suite (https://auth.lifemapsc.com/) with a relevance score ≥5 (Supplementary File S4). The intracellular localization of proteins was derived from The Human Protein Atlas (https://www.proteinatlas.org/).

### Statistical analysis

Statistical analyses were performed using GraphPad Prism 10 software. All in vitro experiments were repeated three times. Quantitative data are expressed as the mean ± SD; a two-tailed Student’s t-test or one-way analysis of variance (ANOVA) were used for parametric variables, and Fisher’s accuracy or χ^2^ tests were used for nonparametric variables. A paired t-test was used for paired samples. Survival analysis was performed by using the Kaplan–Meier method with the log-rank test. P values < 0.05 were considered to indicate significance.

## Results

### IFIT3 is highly expressed in ESCC tissues and is associated with LNM and poor prognosis in ESCC patients

**Fig. 1:**
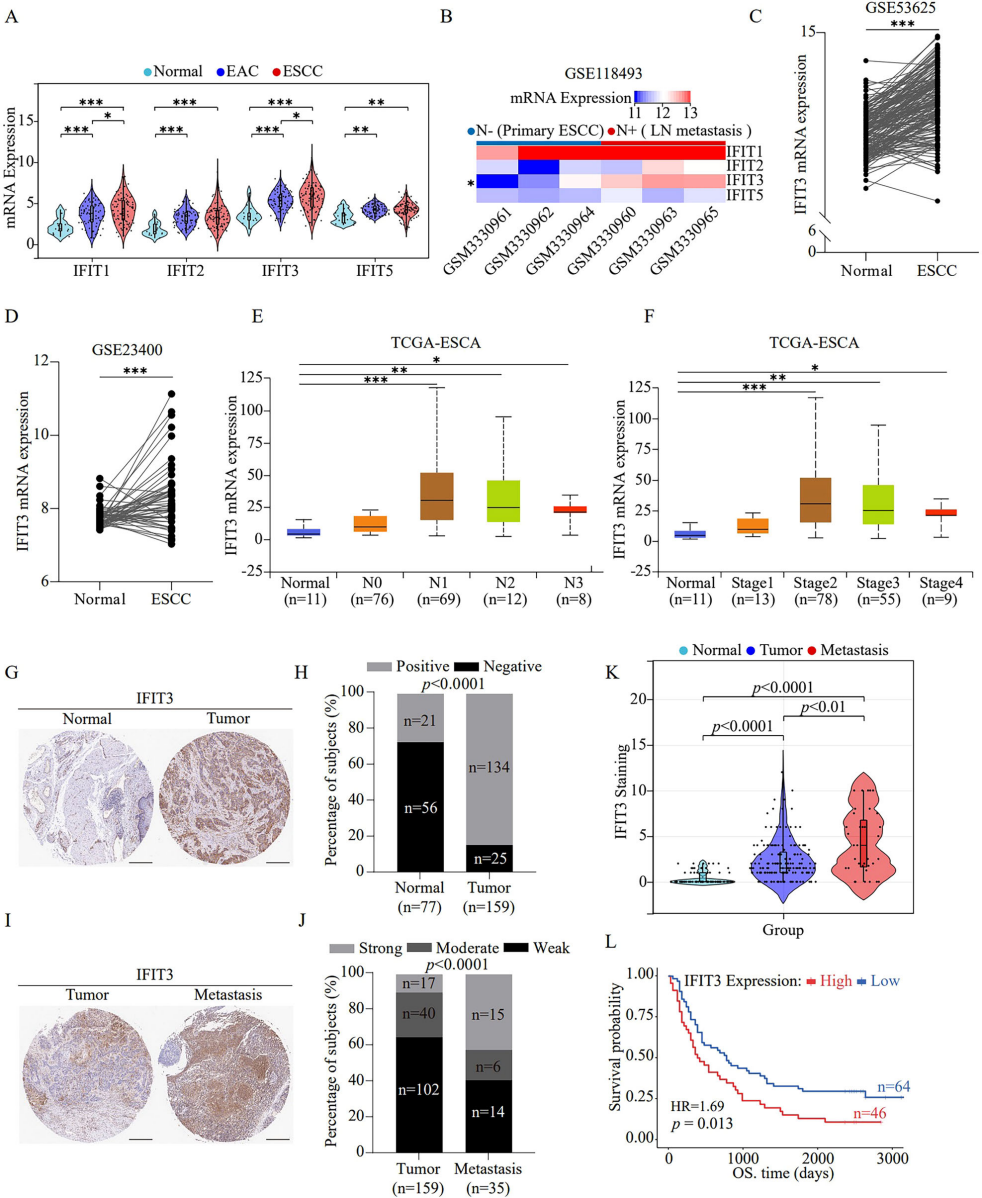
IFIT3 is expressed at high levels in ESCC and LNM tissues and is correlated with dismal prognosis in ESCC patients. **A** The levels of IFIT1, IFIT2, IFIT3, and IFIT5 mRNA expression in ESCC, ECA, and normal tissues from the TCGA-ESCA dataset were analyzed. **B** Heatmap demonstrating the IFIT1, IFIT2, IFIT3, and IFIT5 mRNA expression in tumor tissues from ESCC patients with or without LNM. Maps based on GEO gene expression data (GSE118493). **A**, **B** were analyzed using the online bioinformatics tool SangerBox 3.0 (http://www.sangerbox.com/tool). **C**, **D** The IFIT3 mRNA levels were investigated with the GEO database (GSE53625, GSE23400) to compare IFIT3 mRNA expression in normal esophageal tissue and ESCC tissue. **E**, **F** The IFIT3 mRNA levels in patients with different N stages and different stages of esophageal cancer were examined based on the UALCAN website (http://ualcan.path.uab.edu). Illustrative immunohistochemical images (**G**) and quantitative analysis (**H**) of IFIT3 staining in 159 ESCC tissues and 77 matched normal tissues (scale bar, 300 μm). Representative immunohistochemical images (**I**) and quantitative analysis (**J**) of IFIT3 staining in 159 ESCC tissues and 35 matched metastatic tissues (scale bar, 300 μm). **K** Differences in IFIT3 expression scores between normal tissues (*n* = 77), ESCC tissues (*n* = 159), and metastatic tissues (*n* = 35) were analyzed using the online bioinformatics tool SangerBox 3.0. **L** Survival analysis of 110 ESCC patients stratified by the IFIT3 level (survival curve was generated using https://www.bioinformatics.com.cn, a web-based tool for visualizing and analyzing data). Data information: Graphs show the mean ± SD. **A**, **K** One-way ANOVA; **B**, **E**, **F** Student’s t-test; **C**, **D** paired t-test; **H** Fisher’s exact test; **J** chi-squared test; **L** log-rank test. ***p < 0.001, **p < 0.01, *p < 0.05.

To assess the involvement of IFITs in the progression of ESCC, we first analyzed the mRNA expression of IFITs in ESCC tissues using the Gene Expression Omnibus (GEO) database. The mRNA levels of IFITs in tumor tissues from esophageal adenocarcinoma (EAC) or ESCC patients were significantly higher than those in normal tissues, and the mRNA levels of IFIT1 and IFIT3 in ESCC tissues were visibly higher than those in EAC tissues (Fig. 1A). We subsequently analyzed IFIT mRNA expression in ESCC patients with or without LNM (GSE118493), and heatmaps highlighted that only IFIT3 mRNA expression was significantly higher in patients with LNM than in those without (Fig. 1B). Moreover, additional GEO datasets (GSE53625 and GSE23400) demonstrated that IFIT3 mRNA was significantly upregulated in ESCC tumor tissues compared to that in normal esophageal tissues (Fig. 1C, D). In addition, the expression of IFIT3 in tumor samples of different N and clinical stages was higher than that in adjacent normal tissues (Fig. 1E, F).

We next measured the levels of IFIT3 by IHC in a tissue microarray containing 159 ESCC tissues, 77 adjacent normal tissues, and 35 paired LNM tissues (Supplementary Fig. S1). The tissue microarray arrangement and patient information were listed in Supplementary File S1. The IHC results showed that IFIT3 was primarily expressed in the cytosol and plasma membrane, and 84.28% (134/159) of the ESCC tumors showed positive cytoplasmic staining for IFIT3, whereas only 27.27% (21/77) of nontumor tissues were positive (*P* < 0.0001) (Fig. 1G, H). Importantly, IFIT3 was more strongly expressed (42.9%, 15/35) in metastatic ESCC tissues than in ESCC tissues (10.7%, 17/159) (*P* < 0.0001) (Fig. 1I, J). Moreover, the levels of IFIT3 in ESCC tissues were higher than those in nontumor tissues, while its expression in LNM tissues was higher than that in ESCC tissues (Fig. 1K). Further analysis with the log-rank test revealed that IFIT3 overexpression was tightly related to a shorter survival probability (Fig. 1L). Collectively, these data suggest that IFIT3 is associated with LNM status and may serve as a prognostic marker in ESCC.

### Silencing IFIT3 inhibits ESCC cell metastasis in vitro and LNM in ESCC-bearing nude mice

**Fig. 2:**
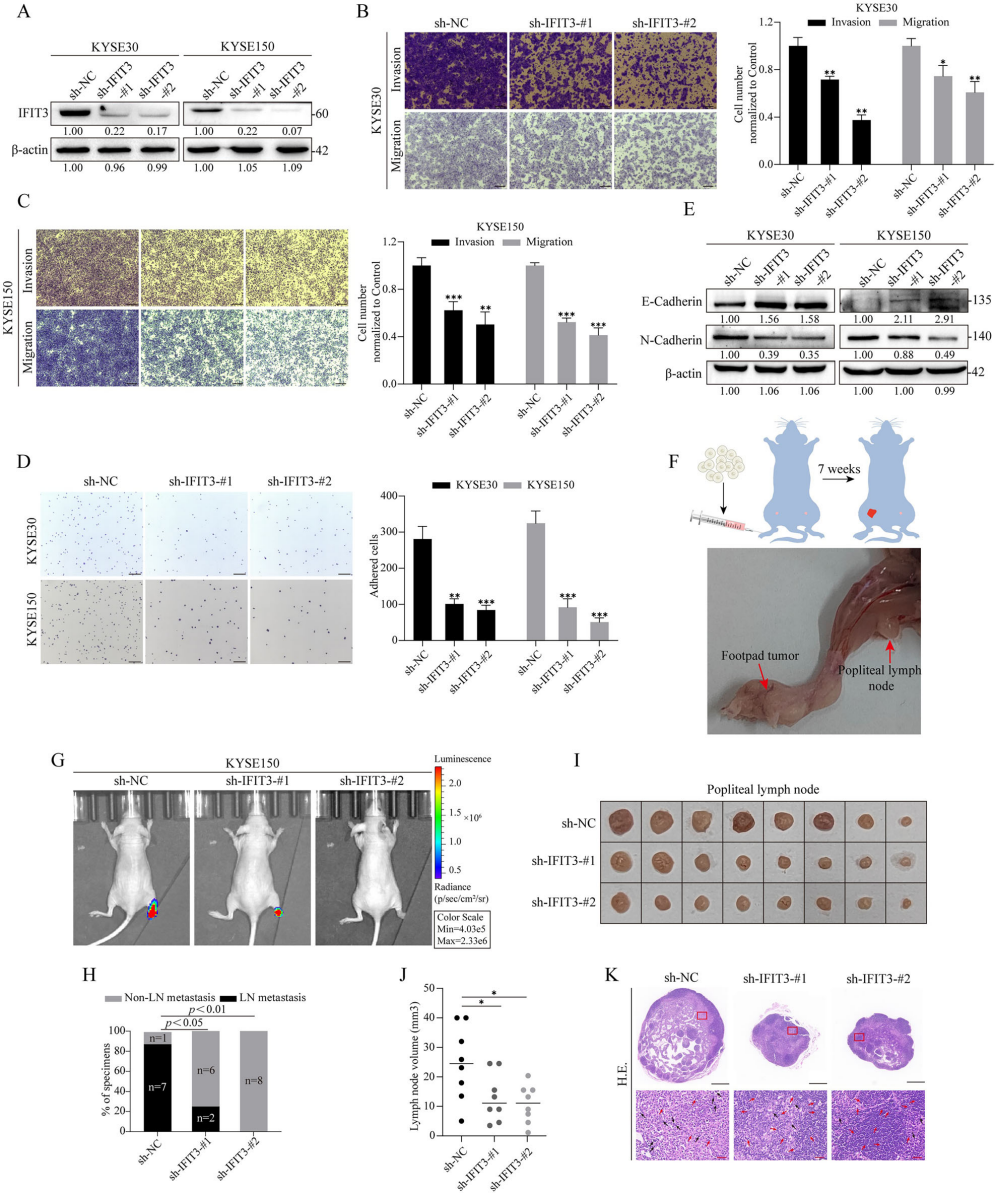
Silencing IFIT3 suppresses ESCC cell metastasis in vitro and LNM in ESCC-bearing nude mice. **A** The IFIT3 protein levels were examined by western blotting in KYSE30 and KYSE150 cells after transfection with shRNA-IFIT3 lentivirus (sh-IFIT3-#1 and sh-IFIT3-#2). The impact of silencing IFIT3 on the invasive and migratory capabilities of KYSE30 (**B**) and KYSE150 (**C**) cells was determined via a Transwell assay. The right panel illustrates the quantification of the invaded cells (scale bar, 250 μm, *n* = 3). **D** A time-limited fibronectin adhesion assay was implemented to quantify the level of FA formation in IFIT3-knockdown KYSE30 and KYSE150 cells. The right panel displays a statistical study of adherent cell quantity (scale bar, 250 μm, *n* = 3). **E** The E-cadherin and N-cadherin expression levels in KYSE30 and KYSE150 cells were analyzed using western blotting after silencing IFIT3. **F** Schematic representation of the protocol used to create a nude mouse model of popliteal lymph node metastasis and a representative image of the popliteal lymph node metastasis model. **G** Bioluminescence images of popliteal LN metastases following IFIT3 silencing using shRNA-IFIT3 lentivirus. **H** Percentage of LN metastases in all groups (*n* = 8). Representative images of popliteal lymph nodes (**I**) and statistical analysis (**J**) of the lymph node volume of all groups (*n* = 8). **K** Representative images of HE staining of popliteal lymph nodes in all groups. Note: black scale bar, 500 μm, red scale bar, 26 μm; black arrows indicate metastasized tumor cells, and red arrows indicate normal cells. Data information: Graphs report the mean ± SD. **C**, **D**, **J** Student’s t-test; **H** Fisher’s exact test. ***p < 0.001; **p < 0.01; *p < 0.05.

To investigate the role of IFIT3 in ESCC cell metastasis, we first measured the expression of IFIT3 in several ESCC cell lines. The protein levels of IFIT3 in KYSE30 and KYSE150 cells were obviously higher than those in KYSE410 cells (Supplementary Fig. S2A). We constructed stable IFIT3-knockdown cell lines in KYSE150 cells by short hairpin RNA; hereafter, the cell lines are abbreviated as KYSE150^shIFIT3^ (Supplementary Fig. S2B). Then, we tested the effect of IFIT3 on metastasis in KYSE30 and KYSE150 cells by silencing IFIT3. After silencing IFIT3 by shRNA-IFIT3-#1 and shRNA-IFIT3-#2 (Fig. 2A), the invasion and migration abilities of KYSE30 and KYSE150 cells were significantly inhibited (Fig. 2B, C). In contrast, the invasion and migration abilities were markedly enhanced after IFIT3 overexpression in KYSE410 and KYSE150^shIFIT3^ cells (Supplementary Fig. S2C–F). Meanwhile, a cell adhesion assay revealed that silencing IFIT3 obviously reduced the adhesion of ESCC cells to fibronectin, whereas IFIT3 overexpression had the opposite effect (Fig. 2D and Supplementary Fig. S2G, H). Next, we measured epithelial–mesenchymal transition (EMT) markers to further determine the effect of IFIT3 on metastasis. The results showed that the level of E-cadherin was obviously increased and the level of N-cadherin was markedly decreased in ESCC cells after silencing IFIT3 (Fig. 2E), while overexpressing IFIT3 clearly downregulated the level of E-cadherin and upregulated the level of N-cadherin in KYSE410 and KYSE150^shIFIT3^ cells (Supplementary Fig. S2I).

An LNM model in nude mice was established to explore the role of IFIT3 in LNM in ESCC cells (Fig. 2F). Notably, silencing IFIT3 by shRNA-IFIT3-#1 and shRNA-IFIT3-#2 obviously inhibited the ability of ESCC cells to metastasize to the popliteal lymph nodes (Fig. 2G). Conversely, IFIT3 overexpression clearly promoted the opposite phenomenon (Supplementary Fig. S2J). Moreover, the LNM rate and the volume of popliteal lymph nodes in the shRNA-IFIT3-#1 and shRNA-IFIT3-#2 groups were significantly lower or smaller than those in the shRNA-negative control (sh-NC) group (Fig. 2H–J), and IFIT3 overexpression exhibited contrary results (Supplementary Fig. S2K–M). Importantly, H&E staining showed that metastatic lymph nodes exhibited more tumor cell characteristics, including irregularly arranged cells, disordered nuclei, and a central area (Fig. 2K and Supplementary Fig. S2N). Taken together, these data suggest that IFIT3 promotes ESCC cell metastasis and LNM in ESCC-bearing nude mice.

### Activation of the FAK-ERK pathway is required for IFIT3-mediated ESCC cell metastasis

**Fig. 3:**
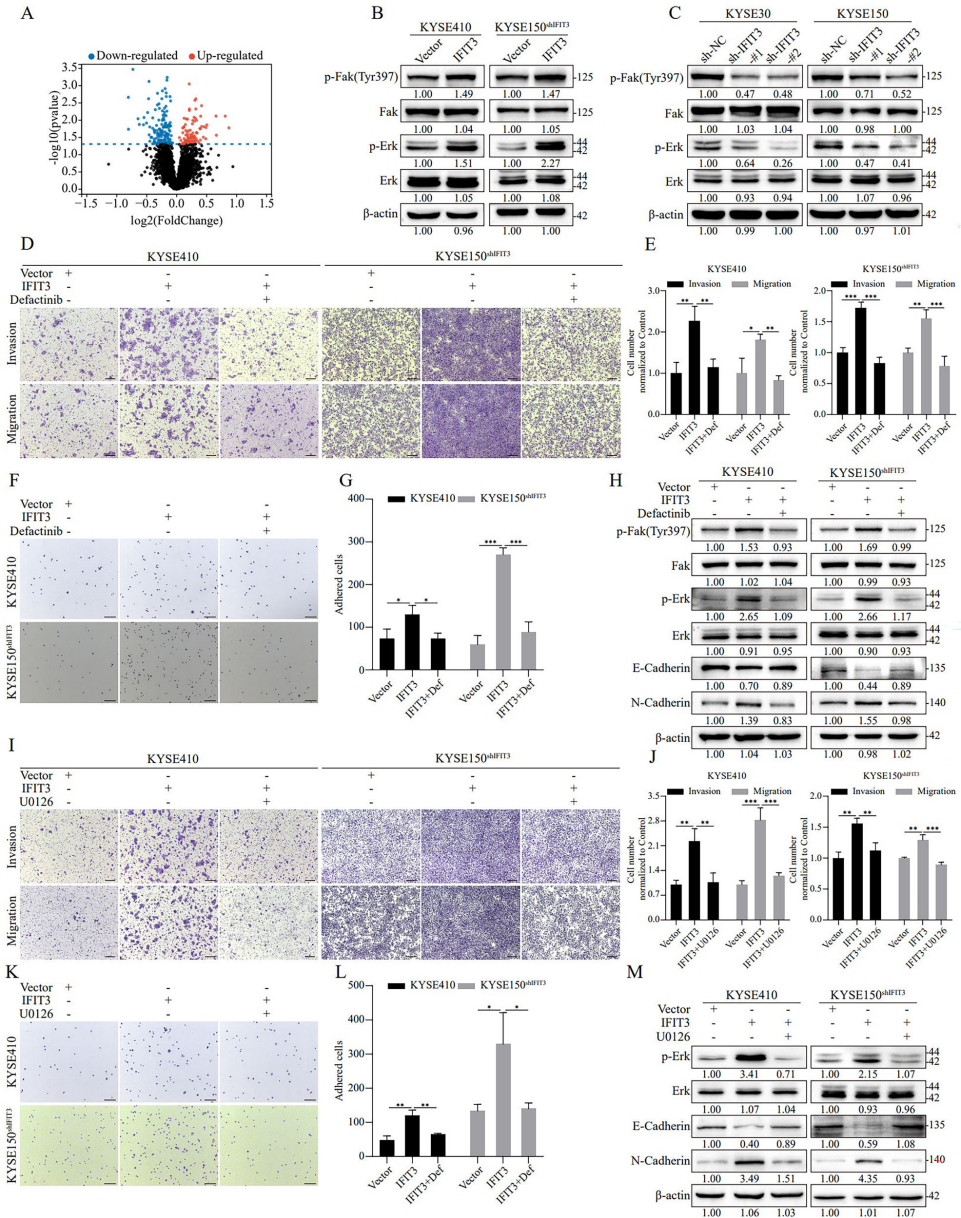
IFIT3 promotes ESCC cell metastasis through FAK-ERK pathway activation. **A** Differentially expressed protein levels in ESCC cells overexpressing IFIT3 were quantified by label-free proteomics and are presented in a volcano plot. The ERK, p-ERK, FAK, and p-FAK (Tyr397) protein levels were analyzed in ESCC cells with IFIT3 overexpression (**B**) or knockdown (**C**) using western blotting. **D**, **E** The invasion and migration ability of KYSE410 and KYSE150shIFIT3 cells was determined via a Transwell assay after co-treatment with IFIT3-overexpressing lentivirus and defactinib. **E** provides the quantification of invasive cells (scale bar, 250 μm, *n* = 3). **F**, **G** FA formation levels in KYSE410 and KYSE150shIFIT3 cells were quantified by a time-limited fibronectin adhesion assay after co-treatment with IFIT3-overexpressing lentivirus and defactinib. **G** The statistical analysis of adherent cell quantity (scale bar, 250 μm, *n* = 3). **H** The p-FAK, FAK, p-ERK, ERK, E-cadherin, and N-cadherin protein levels in KYSE410 and KYSE150shIFIT3 cells were measured by western blotting after co-treatment with IFIT3-overexpressing lentivirus and defactinib. **I**, **J** The invasion and migratory ability of KYSE410 and KYSE150shIFIT3 cells were assessed via a Transwell assay after co-treatment with IFIT3-overexpressing lentivirus and U0126. Quantification of invasive cells is displayed in (**J**) (scale bar, 250 μm, *n* = 3). **K**, **L** FA formation levels in KYSE410 and KYSE150shIFIT3 cells were quantified by a time-limited fibronectin adhesion assay after co-treatment with IFIT3-overexpressing lentivirus and U0126. **L** The statistical analysis of adherent cell count (scale bar 250 μm, *n* = 3). **M** The p-FAK, FAK, p-ERK, ERK, E-cadherin, and N-cadherin protein levels in KYSE410 and KYSE150shIFIT3 cells were measured by western blotting after co-treatment with IFIT3-overexpressing lentivirus and U0126. Data information: Graphs report the mean ± SD. **E**, **G**, **J**, **L** one-way ANOVA. ***p < 0.001; **p < 0.01; *p < 0.05.

To identify the signaling pathway regulated by IFIT3 and involved in the mechanism of ESCC cell metastasis, we used label-free quantitative proteomics to compare the significantly differentially expressed proteins between the IFIT3 overexpression group and the control group (Supplementary File S2). The results were visualized using a volcano map (Fig. 3A), and significantly differentially expressed proteins (FC > 1.5, *P* < 0.05) were analyzed by GO-BP. The analysis revealed that IFIT3 overexpression was significantly correlated with cell migration and the ERK pathway (Supplementary Fig. S3A). Moreover, a KEGG analysis indicated that IFIT3-regulated proteins activated focal adhesion signaling (Supplementary Fig. S3B), which is primarily regulated by focal adhesion kinase (FAK) [19]. Western blotting further showed that IFIT3 overexpression obviously activated ERK and focal adhesion signaling pathways, as evidenced by the upregulated levels of p-FAK and p-ERK in KYSE410 and KYSE150^shIFIT3^ cells (Fig. 3B). Conversely, silencing IFIT3 downregulated the levels of p-FAK and p-ERK in KYSE30 and KYSE150 cells (Fig. 3C). In addition, IHC analysis of metastatic lymph nodes from the LNM model in nude mice was performed to evaluate the effects of IFIT3 on FAK-ERK pathway. In the IFIT3-overexpressing group, p-FAK and p-ERK expression levels were significantly upregulated, whereas their expression was markedly downregulated in the IFIT3-knockdown group. The results revealed that IFIT3 expression correlates with FAK-ERK pathway activation (Supplementary Fig. S3C, D).

Since the ERK pathway can be activated by FAK [19, 20], we applied the FAK inhibitor defactinib to investigate whether the IFIT3-induced ESCC cell metastasis and IFIT3-dependent activation of the ERK pathway were mediated by FAK. We found that defactinib significantly reduced IFIT3 overexpression-induced cell invasion and migration (Fig. 3D, E) and inhibited IFIT3 overexpression-induced adhesion of ESCC cells to fibronectin (Fig. 3F, G). Consistently, the overexpression of IFIT3-mediated increases in p-ERK and N-cadherin and decreases in E-cadherin were attenuated by defactinib (Fig. 3H). Furthermore, we used U0126, an ERK inhibitor, to investigate whether the ERK pathway functionally mediates the prometastatic effects of IFIT3. U0126 treatment significantly reduced the IFIT3 overexpression-mediated increase in invasion, migration, and adhesion in ESCC cells (Fig. 3I–L) and also significantly attenuated the IFIT3 overexpression-mediated changes in the levels of EMT markers (Fig. 3M). These data indicate that IFIT3 promotes ESCC cell metastasis through the FAK/ERK pathway.

### IFIT3 physically interacts and colocalizes with LASP1

To dissect the molecules underlying IFIT3 regulation of the FAK/ERK signaling pathway, we identified IFIT3-interacting proteins by immunoprecipitation coupled with mass spectrometry (IP–MS) (Fig. 3A, B). A total of 148 proteins were pulled down by IFIT3 (Supplementary File S3), among which 39 were potentially involved in the regulation of the focal adhesion signaling pathway (Supplementary Fig. S4A). IFIT3 was primarily expressed in the cytosol and plasma membrane (Fig. 1G). We then analyzed the intracellular sublocalization of the above 39 proteins and found that only ANXA1, CTTN, HSPB1, LASP1, and RPSA were simultaneously localized in the plasma membrane and cytosol (Supplementary Fig. S4A). Further analysis showed that only LASP1 mRNA expression was increased in ESCA versus adjacent normal tissues (Supplementary Fig. S4B–F).

**Fig. 4:**
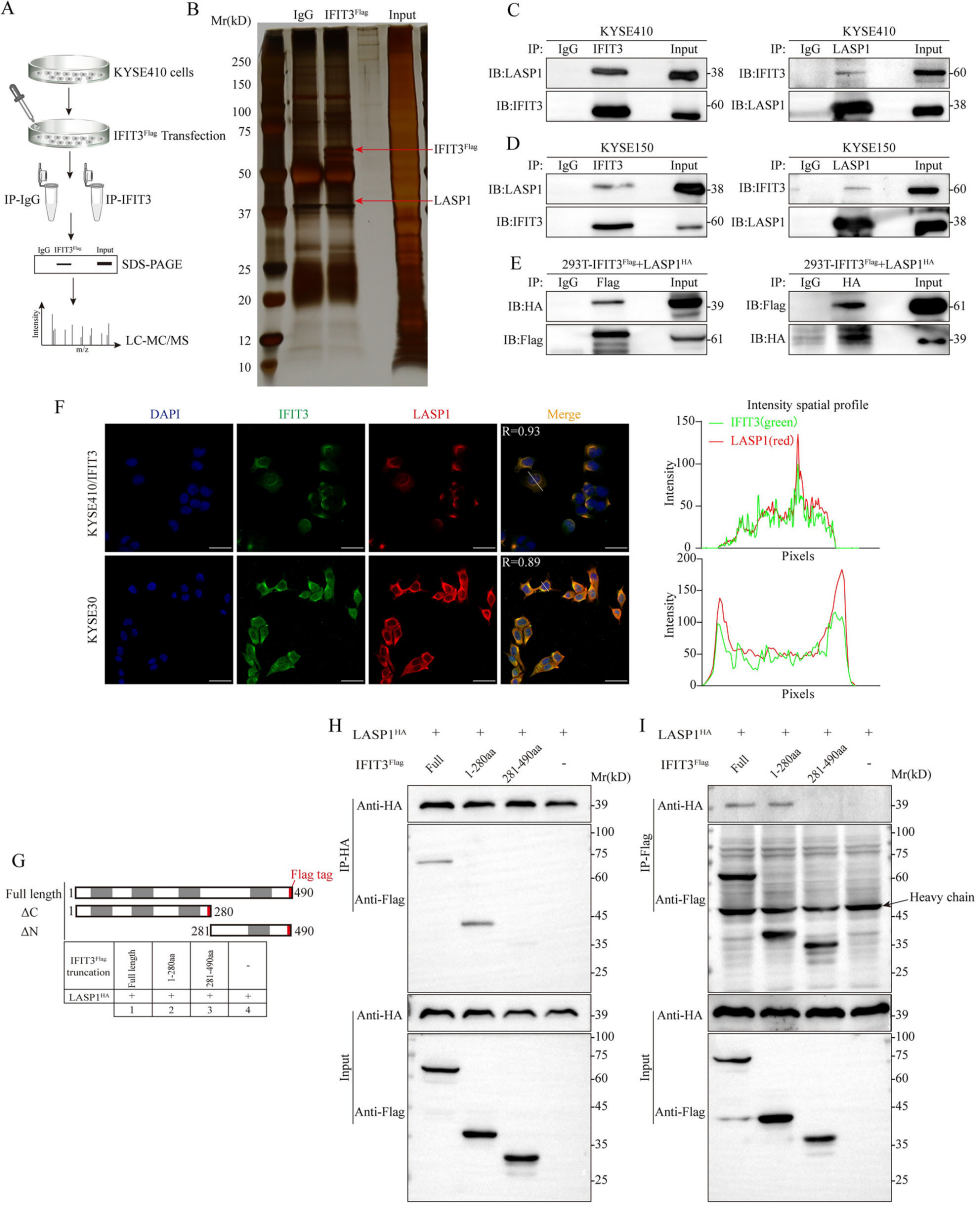
IFIT3 colocalizes and interacts physically with LASP1. **A** Schematic diagram for the identification of IFIT3-interacting proteins. **B** Silver-stained proteins that coimmunoprecipitated with IFIT3 exhibit a distinct 38 kDa band corresponding to LASP1. **C**, **D** Immunoprecipitated (IP) immunoblotting of endogenous or exogenous IFIT3 and endogenous LASP1 from KYSE410 and KYSE150 cell extracts. The negative control used was immunoglobulin G (IgG). **E** Plasmids expressing IFIT3-Flag and LASP1-HA were transfected into 293T cells. Co-immunoprecipitation tests showed that IFIT3 and LASP1 interacted. **F** Immunofluorescence experiments using confocal microscopy showed that IFIT3 (green) and LASP1 (red) colocalized in KYSE410 and KYSE30 cells. The right is the intensity spatial distribution map. Scale bar, 50 μm. **G** The flag-tagged IFIT3 constructs used in this study, which were either full-length or deleted, are shown schematically. Coprecipitation of HA-tagged LASP1 with Flag-tagged IFIT3 or its mutants, examined by anti-HA (**H**) or anti-Flag (**I**) immunoprecipitation and anti-FLAG/HA immunoblots.

Notably, LASP1 is a structural scaffold protein that can be localized to focal adhesion [21], consistent with the role of IFIT3, which can regulate focal adhesion signaling (Supplementary Fig. S3B). The exogenous and endogenous interactions between IFIT3 and LASP1 were verified by IP coupled with western blotting in KYSE410 and KYSE150 cells, respectively (Fig. 4C, D). We also confirmed the exogenous interaction between IFIT3 and LASP1 by using Flag-tagged IFIT3 (IFIT3^Flag^) and HA-tagged LASP1 (LASP1^HA^) plasmids in HEK293T cells (Fig. 4E). IF assays demonstrated that IFIT3 and LASP1 were significantly colocalized in the cytoplasm and at the cell edge of KYSE410 and KYSE30 cells (Fig. 4F).

IFIT3 consists of four TPR domains, which are 34-amino acid degenerate repeat motifs that mediate protein‒protein interactions [22]. The three TPR domains at the N-terminus of IFIT3 are conserved in species such as humans (Supplementary Fig. S4G), bovines, and mice. We then fused IFIT3 with a Flag-tag into N-terminal IFIT3 (ΔC, 1–280 aa) and C-terminal IFIT3 (ΔN, 281–490 aa) (Fig. 4G). Co-IP experiments showed that the N-terminus could interact with LASP1; however, the C-terminus failed to bind to LASP1 (Fig. 4H, I). These findings suggest that IFIT3 could be a promoter of ESCC cell metastasis by interacting with LASP1.

### LASP1 is essential for IFIT3-induced activation of the FAK-ERK signaling pathway

To investigate the role of LASP1 in ESCC metastasis, LASP1 was overexpressed or knocked down in KYSE150 cells (Supplementary Fig. S5A), and a nude mouse model of LNM was generated to validate its function in vivo. Notably, LASP1 overexpression significantly promoted the metastatic ability of ESCC cells to popliteal lymph nodes (Supplementary Fig. S5B). The LNM rate and the volume of popliteal lymph nodes in the LASP1 overexpression group were significantly higher than in the control (vector) group (Supplementary Fig. S5C–E). Importantly, H&E staining revealed that tumor cells in metastatic lymph nodes displayed characteristic malignant features, including irregular cell arrangement, nuclear pleomorphism, and necrotic foci (Supplementary Fig. S5F). Conversely, LASP1 knockdown (shRNA-LASP1-#1 and shRNA-LASP1-#2) markedly suppressed LNM (Supplementary Fig. S5G–K). Collectively, these data demonstrated that LASP1 promotes LNM in ESCC-bearing nude mice.

**Fig. 5:**
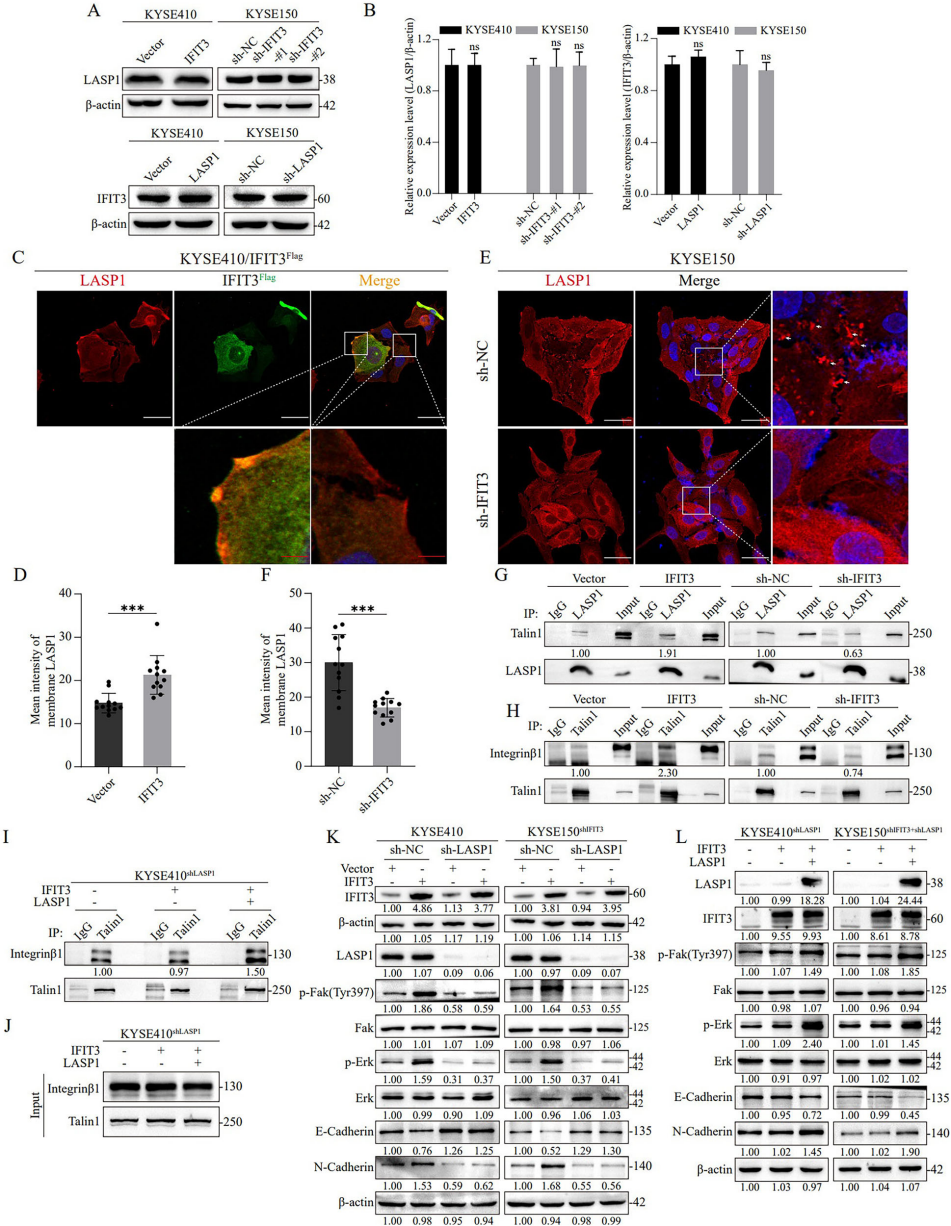
LASP1 is transported to the cell edge by IFIT3, promoting focal adhesion assembly and activating the FAK-ERK signaling pathway. **A**, **B** Western blot shows the LASP1 (top) and IFIT3 (bottom) protein expression levels in ESCC cells after overexpressing or silencing IFIT3 and LASP1 (**A**). **B** Statistical analysis of the above protein levels (*n* = 3). **C**, **D** Transfection of IFIT3^Flag^ or Vector plasmid into KYSE410 cells for 48 h, and the cells above were co-inoculated in the same confocal dish. LASP1 distribution in the cells was then detected by immunofluorescence assay using confocal microscopy (**C**). The mean intensity levels of surface LASP1 are presented in (**D**). White scale bar, 50 μm; red scale bar, 8 μm. *N* = 12 cells analyzed per group. **E**, **F** After the shRNA IFIT3 plasmid was transfected into KYSE150 cells, the distribution of LASP1 in the cells was detected by immunofluorescence assay using confocal microscopy I. The mean intensity levels of surface LASP1 are presented in (**F**). White scale bar, 50 μm; red scale bar, 8 μm. *n* = 12 cells analyzed per group. **G** After transfecting ESCC cells with IFIT3^Flag^ or Vector plasmid (left panel) and shRNA IFIT3 or shRNA-NC plasmid (right panel), the binding of LASP1 to talin1 was detected by co-immunoprecipitation. **H** After transfecting ESCC cells with IFIT3^Flag^ or Vector plasmid (left panel) and shRNA IFIT3 or shRNA-NC plasmid (right panel), the binding of talin1 to Integrin β1 was detected by co-immunoprecipitation. **I**, **J** After overexpression of IFIT3 and LASP1 in KYSE410^shLASP1^ cells, the binding of talin1 to integrin β1 was examined by immunoprecipitation (**I**). **J** Whole-cell lysates as input controls. **K** The LASP1, p-FAK (Tyr397), FAK, p-ERK, ERK, E-cadherin, and N-cadherin expression levels after IFIT3 overexpression in KYSE410 and KYSE150^shIFIT3^ cells with LASP1 knockdown or control, as determined by Western blotting analysis. **L** The levels of IFIT3, LASP1, p-FAK (Tyr397), FAK, p-ERK, ERK, E-cadherin, and N-cadherin expression in KYSE410^shLASP1^ and KYSE150^shIFIT3+shLASP1^ cells following IFIT3 and LASP1 overexpression, as determined by Western blotting analysis. Data information: Graphs report the mean ± SD. **B**, **D**, **F** Student’s t-test. ***p < 0.001.

Considering the interaction between IFIT3 and LASP1, we next investigated their synergistic regulatory effects. The results indicated that the expression of LASP1 could not be regulated by IFIT3, and vice versa (Fig. 5A, B). It has been reported that LASP1 is localized in focal adhesions and is vital for cell migration [21]. Consistent with this report, we found that IFIT3 and LASP1 were colocalized not only in the cytoplasm but also at the edge of the cell membrane (Fig. 4F). Therefore, we hypothesized that IFIT3 may promote cell invasion and migration by affecting the localization of LASP1. Consistently, IF assays validated that IFIT3 overexpression led to increased localization of LASP1 at the cell edge (Fig. 5C, D), whereas silencing IFIT3 reduced the localization of LASP1 on the cell edge (Fig. 5E, F).

LASP1 has been reported to contribute to the migration and invasion of cancer cells by regulating FAK signaling [23]. However, further clarification is required regarding how IFIT3 activates FAK through LASP1. Talin can bind to the integrin β subunit and promote integrin activation, subsequently activating FAK [24]. LASP1 is located in focal contacts, lamellipodia membrane ruffles, pseudopodia, and focal adhesion and is involved in the regulation of cell adhesion and cell migration by binding to several proteins, such as Talin1 [25]. Thus, we hypothesized that IFIT3 promotes the binding of LASP1 to Talin1, thereby promoting the binding of Talin1 to integrin and ultimately activating FAK. The co-IP results confirmed that IFIT3 overexpression promoted the binding of LASP1 and Talin1, whereas silencing IFIT3 had the opposite effect (Fig. 5G). Consistently, Talin1 could bind more integrin β1 in IFIT3-overexpressing cells; however, the opposite pattern was observed in IFIT3-knockdown cells (Fig. 5H).

To explore whether LASP1 is required for the functional effect of IFIT3, we constructed LASP1-knockdown cell lines in KYSE410 cells; hereafter, the cell lines are abbreviated as KYSE410^shLASP1^. IFIT3 overexpression in KYSE410^shLASP1^ cells did not promote an increase in Talin1 binding to integrin β1, whereas overexpression of IFIT3 in KYSE410^shLASP1^ cells with restored LASP1 expression promoted this binding (Fig. 5I, J). Moreover, IFIT3 overexpression increased the levels of p-FAK, p-ERK, and N-cadherin and decreased the level of E-cadherin in KYSE410 and KYSE150^shIFIT3^ cells; however, IFIT3 overexpression did not affect the levels of p-FAK, p-ERK, N-cadherin, and E-cadherin in KYSE410 and KYSE150^shIFIT3^ cells after silencing LASP1 (Fig. 5K). Furthermore, we constructed LASP1-knockdown cell lines in KYSE410 and KYSE150^shIFIT3^ cells; hereafter, the cell lines are abbreviated as KYSE410^shLASP1^ and KYSE150^shIFIT3+shLASP1^. Western blotting showed that restoring LASP1 expression effectively rescued the effect of IFIT3 on the activation of FAK-ERK signaling and the change in the levels of EMT markers (Fig. 5L). These results suggest that LASP1 is required for IFIT3-induced activation of the FAK-ERK signaling pathway.

### LASP1 is essential for IFIT3-induced ESCC cell metastasis

**Fig. 6:**
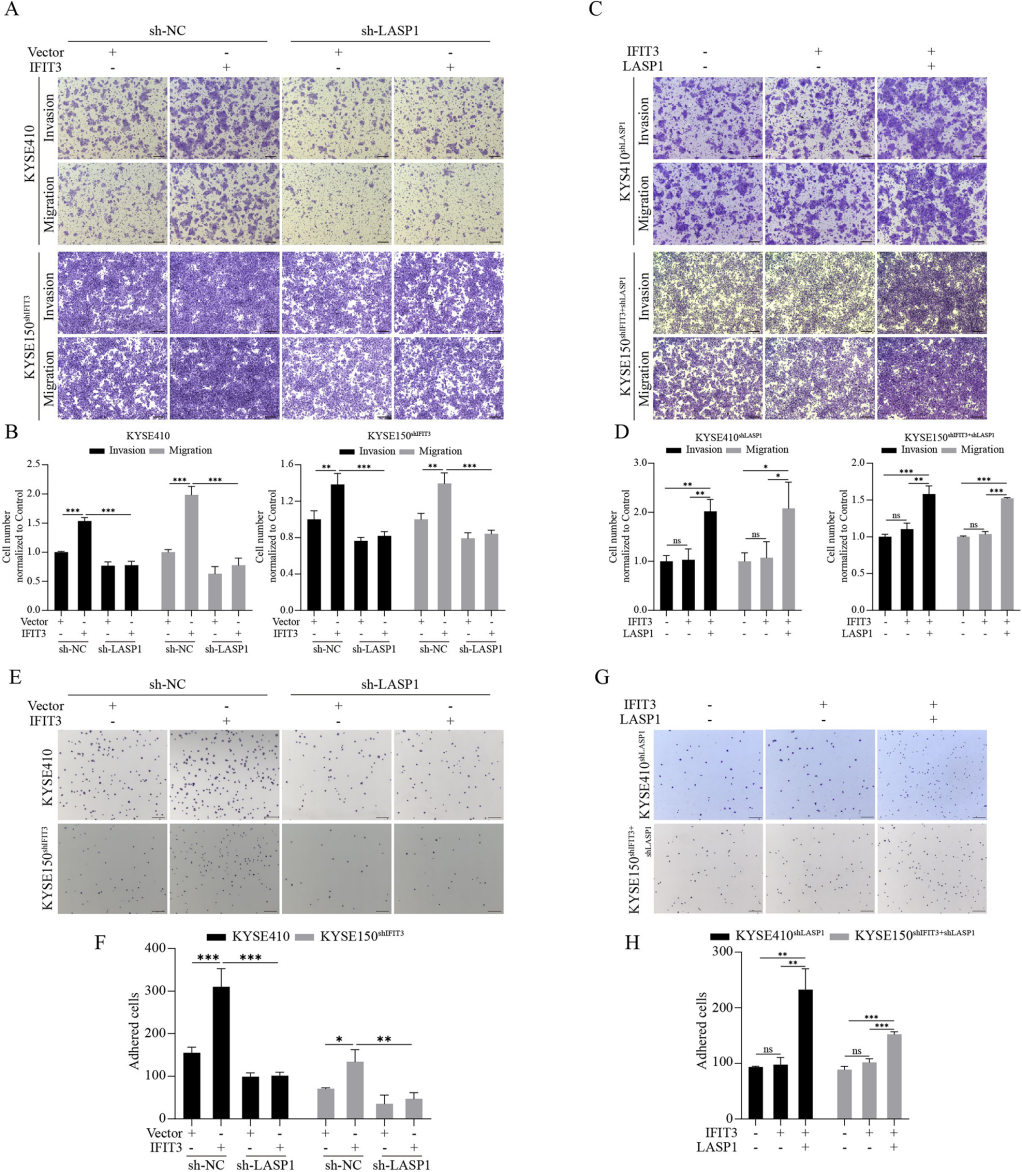
Silencing LASP1 suppresses the prometastatic effect and pro-adhesion ability of IFIT3. **A**, **B** Changes in invasion and migratory ability after IFIT3 overexpression in KYSE410 (top) and KYSE150^shIFIT3^ (bottom) cells with or without LASP1 silencing, as determined by transwell assays. **B** The quantification of invaded cells. Scale bar, 250 μm. **C**, **D** Changes in invasion and migratory ability after overexpression of IFIT3 and LASP1 in KYSE410^shLASP1^ (top) and KYSE150^shIFIT3+shLASP1^ (bottom) cells, as determined by transwell assays. **D** The quantification of invaded cells. Scale bar, 250 μm. **E**, **F** A time-limited fibronectin adhesion assay was used to measure FA formation after IFIT3 overexpression in KYSE410 (top) and KYSE150^shIFIT3^ (bottom) cells with or without LASP1 knockdown. **F** The statistical analysis of the adherent cell count. Scale bar, 250 μm. **G**, **H** A time-limited fibronectin adhesion assay was used to measure FA formation after overexpression of IFIT3 and LASP1 in KYSE410^shLASP1^ (top) and KYSE150^shIFIT3+shLASP1^ (bottom) cells. **H** Statistical analysis of adherent cell count. Scale bar, 250 μm. Data information: Graphs report the mean ± SD. **B**, **D**, **F**, **H** One-way ANOVA. ***p < 0.001; **p < 0.01; *p < 0.05.

A Transwell assay was used to test the cell invasion and migration ability of ESCC cells to investigate the essential role of LASP1 in the regulation of IFIT3-driven ESCC cell metastasis. Silencing LASP1 significantly inhibited IFIT3 overexpression-induced increases in invasion and migration in KYSE410 and KYSE150^shIFIT3^ cells (Fig. 6A, B), and the inhibitory effect of silencing LASP1 on IFIT3-induced invasion and migration was significantly reversed by the reoverexpression of LASP1 in KYSE410^shLASP1^ and KYSE150^shIFIT3+shLASP1^ cells (Fig. 6C, D). Consistently, silencing LASP1 remarkably suppressed the IFIT3 overexpression-induced increase in adhesion in KYSE410 and KYSE150^shIFIT3^ cells (Fig. 6E, F), and the suppressive effect of silencing LASP1 on the IFIT3 overexpression-induced adhesion of tumor cells to fibronectin was significantly attenuated by the reoverexpression of LASP1 in KYSE410^shLASP1^ and KYSE150^shIFIT3+shLASP1^ cells (Fig. 6G, H). These findings demonstrate that LASP1 is required for IFIT3-induced ESCC cell metastasis.

### Clinical significance of IFIT3 and LASP1 in ESCC patients

**Fig. 7:**
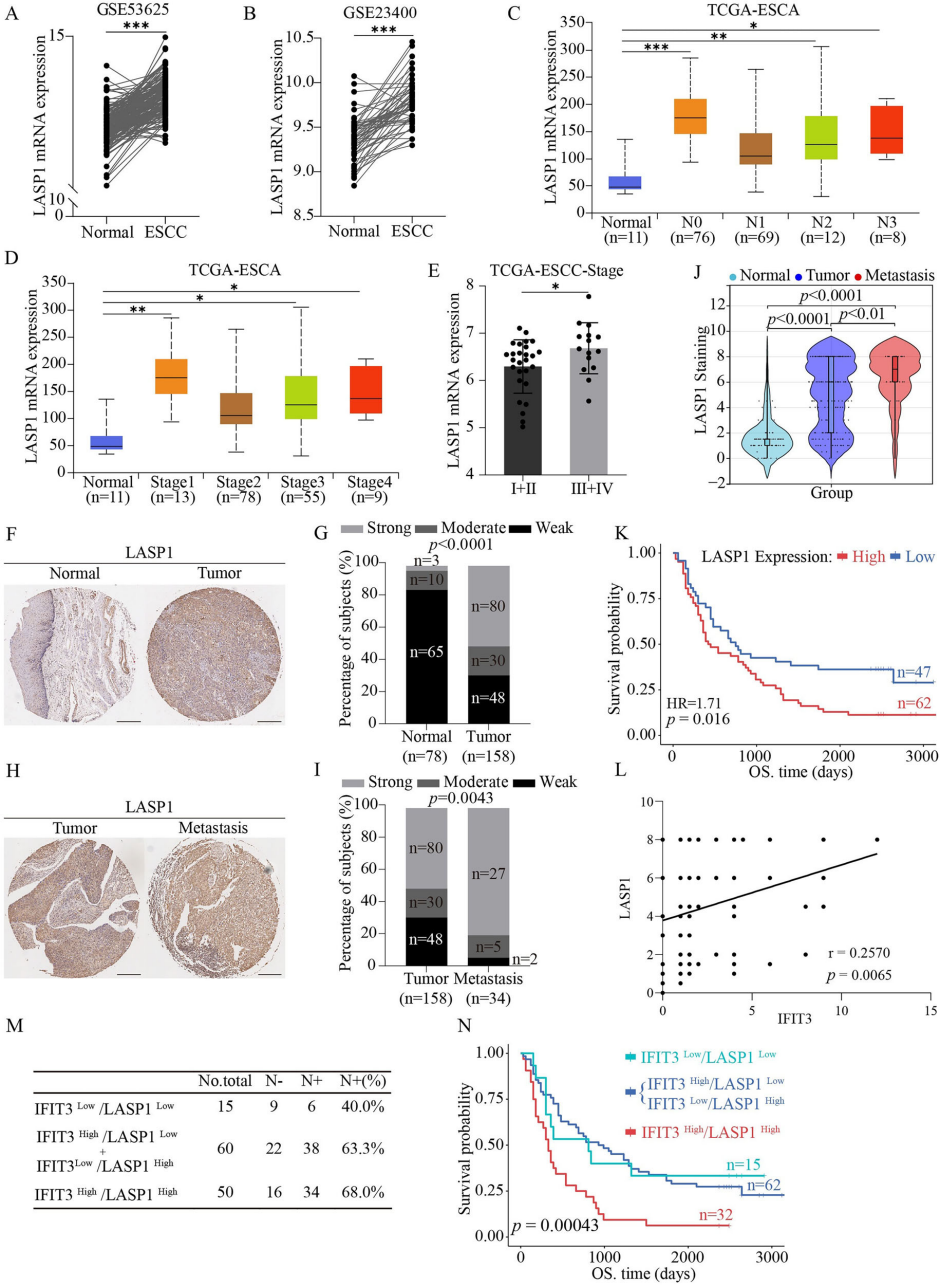
Clinical value of IFIT3 and LASP1 in ESCC patients. **A**, **B** The mRNA expression of LASP1 was analyzed using the GEO database. Maps based on GEO gene expression data (GSE53625, GSE23400) comparing LASP1 gene expression in normal esophageal tissue and ESCC tissue. **C**, **D** LASP1 expression analysis of patients with different N stages and different stages of esophageal cancer on the UALCAN website. **E** Analysis of LASP1 levels in ESCC patients at different stages in the TCGA database. Sample immunohistochemical images (**F**) and quantitative analysis (**G**) of LASP1 staining in 158 ESCC tissues and 78 matched normal tissues (scale bar, 300 μm). Sample immunohistochemical images (**H**) and quantitative analysis (**I**) of LASP1 staining in 158 ESCC tissues and 34 matched metastatic tissues (scale bar, 300 μm). **J** Differences in LASP1 expression scores between normal tissues (*n* = 78), ESCC tissues (*n* = 158), and metastatic tissues (*n* = 34) were analyzed using the online bioinformatics tool SangerBox 3.0. **K** Survival analysis of 109 ESCC patients stratified by the LASP1 level was analyzed using the online bioinformatics tool (https://www.bioinformatics.com.cn). **L** The correlation between IFIT3 and LASP1 in ESCC tissues. **M** The ratio of LNM in patients with different IFIT3 and LASP1 expression. **N** Kaplan–Meier survival analysis was analyzed using the online bioinformatics tool (https://www.bioinformatics.com.cn) to assess the link between expression levels of IFIT3 and LASP1 in tumors and overall survival rates of ESCC patients. Data information: Graphs report the mean ± SD. **A**, **B** Paired t-test; **C**–**E** Student’s t-test; **J** one-way ANOVA; **G**, **I** chi-square test; **K**, **M** log-rank test; **L** Pearson’s correlation analysis. ***p < 0.001; **p < 0.01; *p < 0.05.

To assess the involvement of LASP1 in the progression of ESCC (GSE53625 and GSE23400), we analyzed the expression of LASP1 mRNA in ESCC using the GEO database. The mRNA expression of LASP1 in tumor tissues was significantly higher than that in normal tissues (Fig. 7A, B). In the TCGA-ESCA cohort, the expression of LASP1 in tumor samples of different N stages and clinical stages was higher than that in adjacent normal tissues (Fig. 7C, D). Patients in the TCGA-ESCC datasets in advanced clinical stages tended to have higher LASP1 expression (Fig. 7E). Moreover, the levels of LASP1 were measured by IHC of a tissue microarray containing 158 cases of ESCC tissues, 78 cases of adjacent normal tissues, and 34 paired LNM tissues (Supplementary Fig. S6). The IHC results showed that 50.63% (80/158) of the ESCC tumors stained strongly for cytoplasmic LASP1, whereas only 3.85% (3/78) of nontumor tissues showed strong IFIT3 expression (*P* < 0.0001) (Fig. 7F, G). Interestingly, a higher ratio of strong expression levels of LASP1 (79.4%, 27/34) was observed in metastatic ESCC tissues than in ESCC tissues (50.6%, 80/158) (*P* = 0.0043) (Fig. 7H, I). Moreover, the expression of LASP1 in ESCC tissues was higher than that in nontumor tissues, while its expression in LNM tissues was higher than that in ESCC tissues (Fig. 7J). Further analysis with the log-rank test revealed that LASP1 overexpression was tightly related to a shorter survival probability (Fig. 7K). A significant positive correlation between IFIT3 and LASP1 was observed in ESCC tissues (Fig. 7L). More importantly, IFIT3^high^/LASP1^high^ expression was associated with pathological N-stage (Fig. 7M), and the IFIT3^high^/LASP1^high^ group had a significantly poorer prognosis than the other groups (Fig. 7N). Collectively, these data suggest that IFIT3 and LASP1 may serve as prognostic markers for ESCC patients.

We calculated the activation scores of the FAK and ERK pathways in the GEO dataset (GSE53625). The expression levels of IFIT3 and LASP1 were stratified into high, medium, and low groups based on tertiles. Patients were classified as follows: 24 cases had low expression of both genes, 19 cases had medium expression, and 21 cases had high expression.

Analysis of pathway activation across groups revealed significant differences: FAK pathway activity was significantly higher in the IFIT3^High^/LASP1^High^ group compared to the IFIT3^Low^/LASP1^Low^ group, while ERK pathway activity was elevated in both the IFIT3^Middle^/LASP1^Middle^ group and IFIT3^High^/LASP1^High^ group (Supplementary Fig. S7A, B). Additionally, we analyzed a tissue microarray containing 53 primary ESCC tissues, 30 adjacent normal tissues, and 50 metastatic/non-metastatic lymph node tissues. Samples with IHC scores ≥5 for both IFIT3 and LASP1 were defined as high expression, and those with scores <5 as low expression. The results showed that p-FAK levels in the IFIT3^High^-LASP1^High^ group were higher than in the IFIT3^Low^-LASP1^Low^ group (*P* = 0.02) (Supplementary Fig. S7C). Similarly, p-ERK levels were significantly elevated in the IFIT3^High^-LASP1^High^ group (*P* = 0.05) (Supplementary Fig. S7D). These results demonstrated that IFIT3 and LASP1 expression is associated with FAK/ERK pathway activation in ESCC.

## Discussion

LNM is a clear indicator of tumor invasiveness and transmission capacity [26] and a major focal point in current cancer research [27]. In contrast to other malignant tumors, due to the unique anatomical structure and abundant submucosal lymphatic vessels in the esophagus, ESCA cells can metastasize to nearby and distant lymph nodes through extensive lymphatic drainage even in early-stage esophageal cancer patients [4, 5]. In general, LNM is considered a key independent factor affecting the prognosis of ESCA patients. The 5-year overall survival rate of ESCA patients with a lymph node ratio (LNR) ≤ 10% is 57%, while that of patients with an LNR > 10% is approximately 0% [28]. Thus, clarifying the key factors underlying LNM is urgent for the development of reasonable therapeutic strategies. Here, our data demonstrate that a high level of IFIT3 may be a poor prognostic factor in ESCC patients and that overexpression of IFIT3 enhances the migration and invasion properties of ESCC cells, which are important steps in LNM. More importantly, IFIT3 was found to directly interact with LASP1 and promote the assembly of focal adhesions, subsequently leading to lymph node metastasis of ESCC through activating the FAK-ERK pathway. These findings identify IFIT3 as a prognostic biomarker in ESCC and provide novel mechanistic insight into ESCC metastasis.

Previous studies have shown that IFIT3 plays a crucial role in antiviral innate immunity [29]. Recent studies have shown that IFIT3 is involved in cellular biological processes, particularly in cancer development, including oral squamous cell carcinoma [30], pancreatic cancer [17], and hepatocellular carcinoma [14]. IFIT3 exhibits gemcitabine resistance in pancreatic ductal adenocarcinoma cells and can be used as an indicator for judging the use of gemcitabine targeted therapy [31]. Further study showed that IFIT3 directly binds to voltage-dependent anion-selective channel protein 2 and promotes chemotherapy resistance [17]. IFIT3 has also been reported to be involved in cancer metastasis [14, 18]. However, to date, few detailed studies have characterized the relationship between IFIT3 and LNM, especially in ESCC.

Here, we first reported and elucidated the mechanism by which IFIT3 promotes LNM in ESCC. Aberrant reactivation of EMT plays a crucial role in promoting cancer migration and invasion [32]. In this study, we found that IFIT3 could promote the migration and invasion of ESCC cells and EMT progression. Importantly, the FAK-ERK signaling pathway is associated with cancer progression, especially metastasis [20, 33]. Our results indicated that IFIT3 could activate the FAK-ERK signaling pathway, and we confirmed that IFIT3 regulates tumor invasion and metastasis through the FAK-ERK signaling pathway by introducing inhibitors of FAK and ERK. These results further support the carcinogenic role of IFIT3 in the metastasis of ESCC cells.

Subsequently, we identified LASP1 as a novel binding partner of IFIT3. Our results confirmed the interaction and colocalization of IFIT3 and LASP1 and revealed that the N-terminus rather than the C-terminus of IFIT3 is essential for the binding between IFIT3 and LASP1. LASP1 is a scaffold protein originally identified from a cDNA library of axillary lymph node metastasis in breast cancer [34]. Increasing evidence suggests that LASP1 is a tumor-promoting factor abnormally expressed in breast cancer [35], ovarian cancer [36], hepatocellular carcinoma [37], colorectal cancer [14, 38], and ESCC [39]. Recently, several studies have shown that LASP1 is involved in the invasion and migration of tumor cells. LASP1 has been shown to interact with lipoma preferred partners and zyxin, affecting the rate and organization of actin polymerization and the turnover of focal adhesion in protrusions, which mediate the invasive phenotype of ovarian cancer and hepatocellular carcinoma cells [40, 41]. Another study showed that LASP1 promotes EMT in tumor cells by activating MAPK, PI3K/AKT, and Smad signaling pathways in colorectal cancer [42]. Importantly, LASP1 has been reported to enhance the invasion and metastasis ability of ESCC [39, 43]; however, the roles of LASP1 in ESCC metastasis and the regulatory mechanisms remain unclear.

LASP1 is localized to focal adhesions, pseudopodia, and membrane folds, which may affect the invasion and migration of tumor cells. We first confirmed that IFIT3 and LASP1 do not affect each other’s expression. Surprisingly, IFIT3 can promote the localization of LASP1 at the cell edge. Considering that the phosphorylation of FAK is induced by activated integrin, which can be activated by Talin1, LASP1 could interact with Talin1 at the cell edge to activate cell adhesion. Therefore, we speculate that IFIT3 may promote the interaction of LASP1 and Talin1, thereby promoting the binding of Talin1 to integrin and ultimately activating FAK. Consistent with this hypothesis, our results confirmed the interaction of LASP1 with Talin1 and Talin1 with integrin by co-IP experiments in gain- and loss-of-IFIT3 ESCC cells.

Finally, we analyzed the expression of LASP1 in ESCC in situ and LNM tissues by immunohistochemical and bioinformatics methods. The results showed that LASP1 expression in ESCC was significantly higher than that in paracancerous normal samples, and the expression of LASP1 in ESCC tissues with LN metastasis (N+) was higher than that in ESCC tissues without LN metastasis (N−). Moreover, ESCC patients with high LASP1 expression have a worse survival prognosis. Notably, IFIT3^high^/LASP1^high^ was associated with pathological N-stage ESCC, and such patients had a significantly poorer prognosis.

This study had some limitations. The number of specimens, especially LNM specimens, was limited. In addition, the mechanism by which IFIT3 promotes LASP1 localization to the cell edge requires further exploration, which is a focus of our future research. Third, this study did not include in vivo experiments to further validate the identified mechanistic pathway due to limited research funding. Fourth, although high expression of both IFIT3 and LASP1 is associated with poor prognosis in patients, and patients with high expression of both molecules have the worst prognosis, the incomplete clinical follow-up information of the current ESCC cohort prevents us from drawing the conclusion that the expression of IFIT3 and LASP1 serves as independent prognostic factors for ESCC patients. Therefore, the prognostic significance of IFIT3 and LASP1 requires further verification in larger ESCC cohorts with more comprehensive clinical data and complete follow-up information.

To address these limitations, we have outlined clear directions for future work. Beyond investigating the mechanism underlying IFIT3-mediated LASP1 localization, we plan to prioritize in vivo validation of the pathway once additional research funding is secured. Concurrently, we are also actively collecting larger and independent validation cohorts with complete clinical follow-up information, which will focus on verifying the independent prognostic value of IFIT3 and LASP1 in ESCC. These efforts aim to strengthen the translational relevance and robustness of our findings to clinical practice.

In conclusion, our study suggested that IFIT3 promoted LNM in ESCC by activating the FAK-ERK signaling pathway and promoting EMT in ESCC cells in a LASP1-dependent manner. To the best of our knowledge, no previous studies have addressed the specific function of IFIT3 in ESCC and the interaction between IFIT3 and LASP1. Our data suggested that the targeted interaction of IFIT3 and LASP1 may be a promising strategy for the treatment of ESCC LNM.

## Supplementary information

Supplementary Materials

Raw Data-Western Blot

Supplementary Information

Supplementary Information

Supplementary Information

Supplementary Information

Supplementary Information

Supplementary Information

Supplementary Information

## Data Availability

All necessary data for assessing the results presented in the study may be found either in the manuscript or in the Supplementary Materials. Further information about this manuscript may be requested from the corresponding authors.
