# Supplementary material for: IFIT3 promotes lymph node metastasis by interacting with LASP1 to activate FAK-ERK signaling in esophageal squamous cell carcinoma
Source: Cell Death Dis. 2025 Dec 18;17(1):110. doi: 10.1038/s41419-025-08327-z (PMC12847741; doi:10.1038/s41419-025-08327-z)
Supplement: Supplementary file 1 — Supplementary Materials [file 41419_2025_8327_MOESM1_ESM.pdf]

Supplementary Figure legends

Figure S1. All tissue array data used for Fig. 1G and Fig. 1I.

Figure S2. IFIT3 overexpression promotes ESCC cell metastasis in vitro and LNM in ESCC-bearing nude mice.

(A) The protein levels of IFIT3 in ESCC cells. (B) The protein levels of IFIT3 in KYSE410 and KYSE150shIFIT3 cells after transfection with IFIT3-overexpressing lentivirus. (C-F) The impact of IFIT3 overexpression on the migration and invasion of KYSE410 (C) and KYSE150shIFIT3 (E) cells was assessed using a Transwell assay. The number of invasive cells was quantified in panels (D) and (F). (scale bar, 250 μm, n = 3). (G-H) A time-limited fibronectin adhesion assay was implemented to quantify the level of FA formation in KYSE410 and KYSE150 cells after overexpressing IFIT3. (G) Statistical analysis of the number of adherent cells (scale bar, 250 μm, n = 3). (I) The protein levels of E-cadherin and N-cadherin in KYSE410 and KYSE150shIFIT3 cells after IFIT3 overexpression. (J) Representative images of bioluminescence of popliteal LN metastasis after IFIT3 overexpression. (K) Percentage of LN metastases in all groups (n = 8). (L-M) Representative images of popliteal lymph nodes (L) and statistical analysis (M) of the lymph node volume of all groups (n = 8). (N) Representative images of HE staining of popliteal lymph nodes in all groups. Note: black scale bar, 500 μm, red scale, bar 26 μm; black arrows indicate metastasized tumor cells, and red arrows indicate normal cells. Data information: Graphs report the mean ± SD. Significance was evaluated using a two-tailed Student's t test, except for in K, for which Fisher's exact test was utilized. *** $P < 0.001$; ** $P < 0.01$; * $P < 0.05$.

Figure S3. IFIT3 promotes ESCC cell metastasis through FAK-ERK pathway activation.

Significantly differentially expressed proteins (FC > 1.5, $P < 0.05$) were analyzed using GO-BP (A) and KEGG (B) enrichment analyses. (C) Representative immunohistochemical staining images and quantitative statistical graphs of p-Fak and p-Erk in popliteal lymph node metastatic

tissues from control and IFIT3-overexpressing groups. scale bar, 50 μm, (D) Representative immunohistochemical staining images and quantitative statistical graphs of p-Fak and p-Erk in popliteal lymph node metastatic tissues from control and IFIT3-knockdown groups. Data information: Graphs report the mean ± SD. C and D, Student's t test. *** $p < 0.001$; ** $p < 0.01$.

Figure S4. IFIT3 interacts with LASP1.

(A) Venn diagram showing genes involved in the focal adhesion pathway that overlap with proteins identified by mass spectrometry (left), with genes in red font localized to the plasma membrane and cytosol (right). (B-F) Analysis of ANXA1, CTTN, HSPB1, LASP1, and RPSA mRNA expression levels in ESCA and normal esophageal tissues in the TCGA and GTEx databases using GEPIA2 (http://gepia2.cancer-pku.cn/). (G) Domain structure of the IFIT3 protein from different species. Data information: Graphs report the mean ± SD. Significance was evaluated using a two-tailed Student's t test. * $P < 0.05$.

Figure S5. LASP1 promotes LNM in ESCC-bearing nude mice.

(A) The LASP1 protein levels were examined by western blotting in KYSE150 cells after transfection with LASP1 or shRNA-LASP1 lentivirus (sh-LASP1-#1 and sh-LASP1-#2). (B) Bioluminescence images of popliteal LN metastases following LASP1 overexpression. (C) Percentage of LN metastases in all groups ($n = 8$). (D-E) Representative images of popliteal lymph nodes (D) and statistical analysis (E) of the lymph node volume of all groups (n = 8). (F) Representative images of HE staining of popliteal lymph nodes in all groups. Note: black scale bar, 500 μm, red scale bar, 26 μm; black arrows indicate metastasized tumor cells, and red arrows indicate normal cells. (G) Bioluminescence images of popliteal LN metastases following LASP1 silencing. (H) Percentage of LN metastases in all groups ($n = 8$). (I-J) Representative images of popliteal lymph nodes (I) and statistical analysis (J) of the lymph node volume of all groups (n = 8). (K) Representative images of HE staining of popliteal lymph nodes in all groups. Note: black scale bar, 500 μm, red scale bar, 26 μm; black arrows indicate metastasized tumor cells, and red arrows indicate normal cells. Data information: Graphs report

the mean ± SD. E and J, Student's t test; C and H, Fisher's exact test. *** $p < 0.001$; ** $p < 0.01$; * $p < 0.05$.

Figure S6. All tissue array data used for Fig. 7H and Fig. 7J.

Figure S7. Clinical value of FAK/ERK activation and IFIT3/LASP1 expression in ESCC patients.

(A-B) The Correlation between the FAK (A) and ERK (B) pathway activation and IFIT3/LASP1 expression was analyzed using the GEO database (GSE53625). (C-D) The Correlation between p-FAK (C)/ p-ERK (D) expression and IFIT3/LASP1 expression was analyzed using a tissue microarray, which consists of 53 primary ESCC tissues, 30 adjacent normal tissues, and 50 metastatic/non-metastatic lymph node tissues. A-D were analyzed using the online bioinformatics tool SangerBox 3.0.

Supplementary Material

Table S1. The sequences of IFIT3-targeting and LASP1-targeting shRNA.

shRNA name	shRNA sequence
shIFIT3#1	CCGGGCTATGGACTATTCGAATAAACTCGAGTTTATTCGAATAGTCCATAGCTTTTTT
shIFIT3#2	CCGGATGTTGCTCTAAGGTACATTTCTCGAGAAATGACCTTAGAGCAACATTTTTT
shLASP1	CCGGACCTGCGACAGCTTGTGATTCCTCGAGGAATCACAAGCTGTCGCAGGTTTTTTT
shLASP1#1	CCGGACCTGCGACAGCTTGTGATTCCTCGAGGAATCACAAGCTGTCGCAGGTTTTTTT
shLASP1#2	CCGGCTGGATAAGTTCTGGCATAAACTCGAGTTTATGCCAGAACTTATCCAGTTTTT

Supplementary file 1:

Tissue microarray arrangement

Point position	ID	Tissue type	Pathological classification
A1	D08A0181-B30-C1	Tumor	ESCC
A2	D08A0181-B30-P1	Normal	Normal esophageal tissue
A3	D08A0184-B30-C1	Tumor	ESCC
A4	D08A0184-B30-P1	Normal	Normal esophageal tissue
A5	D08A0185-B30-C1	Tumor	ESCC
A6	D08A0185-B30-P1	Normal	Normal esophageal tissue
A7	D08A0187-B30-C1	Tumor	ESCC
A8	D08A0187-B30-P1	Normal	Normal esophageal tissue
A9	D08A0196-B30-C1	Tumor	ESCC
A10	D08A0196-B30-P1	Normal	Normal esophageal tissue
A11	D08A0201-B30-C1	Tumor	ESCC
A12	D08A0201-B30-P1	Normal	Normal esophageal tissue
A13	D08A0203-B30-C1	Tumor	ESCC
A14	D08A0203-B30-P1	Normal	Normal esophageal tissue
A15	D08A0211-B30-C1	Tumor	ESCC
A16	D08A0211-B30-P1	Normal	Normal esophageal tissue
A17	D08A0214-B30-C1	Tumor	ESCC
A18	D08A0214-B30-P1	Normal	Normal esophageal tissue
B1	D08A0256-B30-C1	Tumor	ESCC
B2	D08A0256-B30-P1	Normal	Normal esophageal tissue
B3	D08A0257-B30-C1	Tumor	ESCC
B4	D08A0257-B30-P1	Normal	Normal esophageal tissue
B5	D08A0260-B30-C1	Tumor	ESCC
B6	D08A0260-B30-P1	Normal	Normal esophageal tissue
B7	D08A0284-B30-C1	Tumor	ESCC
B8	D08A0284-B30-P1	Normal	Normal esophageal tissue
B9	D08A0286-B30-C1	Tumor	ESCC
B10	D08A0286-B30-P1	Normal	Normal esophageal tissue
B11	D08A0293-B30-C1	Tumor	ESCC
B12	D08A0293-B30-P1	Normal	Normal esophageal tissue
B13	D08A0373-B30-C1	Tumor	ESCC
B14	D08A0373-B30-P1	Normal	Normal esophageal tissue
B15	D08A0376-B30-C1	Tumor	ESCC
B16	D08A0376-B30-P1	Normal	Normal esophageal tissue
B17	D08A0379-B30-C1	Tumor	ESCC
B18	D08A0379-B30-P1	Normal	Normal esophageal tissue
C1	D08A0380-B30-C1	Tumor	ESCC
C2	D08A0380-B30-P1	Normal	Normal esophageal tissue
C3	D08A0381-B30-C1	Tumor	ESCC
C4	D08A0381-B30-P1	Normal	Normal esophageal tissue

C5	D08A0383-B30-C1	Tumor	ESCC
C6	D08A0383-B30-P1	Normal	Normal esophageal tissue
C7	D08A0385-B30-C1	Tumor	ESCC
C8	D08A0385-B30-P1	Normal	Normal esophageal tissue
C9	D08A0393-B30-C1	Tumor	ESCC
C10	D08A0393-B30-P1	Normal	Normal esophageal tissue
C11	D08A0401-B30-C1	Tumor	ESCC
C12	D08A0401-B30-P1	Normal	Normal esophageal tissue
C13	D08A0402-B30-C1	Tumor	ESCC
C14	D08A0402-B30-P1	Normal	Normal esophageal tissue
C15	D08A0403-B30-C1	Tumor	ESCC
C16	D08A0403-B30-P1	Normal	Normal esophageal tissue
C17	D08A0404-B30-C1	Tumor	ESCC
C18	D08A0404-B30-P1	Normal	Normal esophageal tissue
D1	D08A0405-B30-C1	Tumor	ESCC
D2	D08A0405-B30-P1	Normal	Normal esophageal tissue
D3	D08A1772-B30-C1	Tumor	ESCC
D4	D08A1772-B30-N1	Normal	Normal esophageal tissue
D5	D08A0514-B30-C1	Tumor	ESCC
D6	D08A0514-B30-P1	Normal	Normal esophageal tissue
D7	D08A0515-B30-C1	Tumor	ESCC
D8	D08A0515-B30-P1	Normal	Normal esophageal tissue
D9	D08A0571-B30-C1	Tumor	ESCC
D10	D08A0571-B30-P1	Normal	Normal esophageal tissue
D11	D08A0572-B30-C1	Tumor	ESCC
D12	D08A0572-B30-P1	Normal	Normal esophageal tissue
D13	D08A0573-B30-C1	Tumor	ESCC
D14	D08A0573-B30-P1	Normal	Normal esophageal tissue
D15	D08A0577-B30-C1	Tumor	ESCC
D16	D08A0577-B30-P1	Normal	Normal esophageal tissue
D17	D08A0579-B30-C1	Tumor	ESCC
D18	D08A0579-B30-P1	Normal	Normal esophageal tissue
E1	D08A0580-B30-C1	Tumor	ESCC
E2	D08A0580-B30-P1	Normal	Normal esophageal tissue
E3	D08A0581-B30-C1	Tumor	ESCC
E4	D08A0581-B30-P1	Normal	Normal esophageal tissue
E5	D08A0583-B30-C1	Tumor	ESCC
E6	D08A0583-B30-P1	Normal	Normal esophageal tissue
E7	D08A0584-B30-C1	Tumor	ESCC
E8	D08A0584-B30-P1	Normal	Normal esophageal tissue
E9	D08A0588-B30-C1	Tumor	ESCC
E10	D08A0588-B30-P1	Normal	Normal esophageal tissue
E11	D08A0594-B30-C1	Tumor	ESCC
E12	D08A0594-B30-P1	Normal	Normal esophageal tissue

E13	D08A0596-B30-C1	Tumor	ESCC
E14	D08A0596-B30-P1	Normal	Normal esophageal tissue
E15	D08A0649-B30-C1	Tumor	ESCC
E16	D08A0649-B30-P1	Normal	Normal esophageal tissue
E17	D08A0692-B30-C1	Tumor	ESCC
E18	D08A0692-B30-P1	Normal	Normal esophageal tissue
F1	D08A0694-B30-C1	Tumor	ESCC
F2	D08A0694-B30-P1	Normal	Normal esophageal tissue
F3	D08A0696-B30-C1	Tumor	ESCC
F4	D08A0696-B30-P1	Normal	Normal esophageal tissue
F5	D08A0697-B30-C1	Tumor	ESCC
F6	D08A0697-B30-P1	Normal	Normal esophageal tissue
F7	D08A0699-B30-C1	Tumor	ESCC
F8	D08A0699-B30-P1	Normal	Normal esophageal tissue
F9	D08A1820-B30-C1	Tumor	ESCC
F10	D08A1820-B30-N1	Normal	Normal esophageal tissue
F11	D08A0718-B30-C1	Tumor	ESCC
F12	D08A0718-B30-P1	Normal	Normal esophageal tissue
F13	D08A0719-B30-C1	Tumor	ESCC
F14	D08A0719-B30-P1	Normal	Normal esophageal tissue
F15	D08A0746-B30-C1	Tumor	ESCC
F16	D08A0746-B30-P1	Normal	Normal esophageal tissue
F17	D08A0753-B30-C1	Tumor	ESCC
F18	D08A0753-B30-P1	Normal	Normal esophageal tissue
G1	D08A1837-B30-C1	Tumor	ESCC
G2	D08A1837-B30-P1	Normal	Normal esophageal tissue
G3	D08A1843-B30-C1	Tumor	ESCC
G4	D08A1843-B30-P1	Normal	Normal esophageal tissue
G5	D08A0212-B30-C1	Tumor	ESCC
G6	D08A0212-B30-P1	Normal	Normal esophageal tissue
G7	D08A0218-B30-C1	Tumor	ESCC
G8	D08A0218-B30-P1	Normal	Normal esophageal tissue
G9	D08A0334-B30-C1	Tumor	ESCC
G10	D08A0334-B30-P1	Normal	Normal esophageal tissue
G11	D08A0337-B30-C1	Tumor	ESCC
G12	D08A0337-B30-P1	Normal	Normal esophageal tissue
G13	D08A0340-B30-C1	Tumor	ESCC
G14	D08A0340-B30-P1	Normal	Normal esophageal tissue
G15	D08A0384-B30-C1	Tumor	ESCC
G16	D08A0384-B30-P1	Normal	Normal esophageal tissue
G17	D08A0648-B30-C1	Tumor	ESCC
G18	D08A0648-B30-P1	Normal	Normal esophageal tissue
H1	D08A0652-B30-C1	Tumor	ESCC
H2	D08A0652-B30-P1	Normal	Normal esophageal tissue

H3	D08A0702-B30-C1	Tumor	ESCC
H4	D08A0702-B30-P1	Normal	Normal esophageal tissue
H5	D08A0701-B30-C1	Tumor	ESCC
H6	D08A0701-B30-P1	Normal	Normal esophageal tissue
H7	D08A0705-B30-C1	Tumor	ESCC
H8	D08A0705-B30-P1	Normal	Normal esophageal tissue
H9	D08A0723-B30-C1	Tumor	ESCC
H10	D08A0723-B30-P1	Normal	Normal esophageal tissue
H11	D08A1731-B30-N1	Tumor	ESCC
H12	D08A0188-B30-C1	Tumor	ESCC
H13	D08A0209-B30-C1	Tumor	ESCC
H14	D08A0213-B30-C1	Tumor	ESCC
H15	D08A0219-B30-C1	Tumor	ESCC
H16	D08A0290-B30-C1	Tumor	ESCC
H17	D08A0651-B30-C1	Tumor	ESCC
H18	D08A0650-B30-C1	Tumor	ESCC
I1	D08A1834-B30-C1	Tumor	ESCC
I2	D08A0748-B30-C1	Tumor	ESCC
I3	D08A1844-B30-C1	Tumor	ESCC
I4	D08A0835-B30-C1	Tumor	ESCC
I5	D08A0194-B30-C1	Tumor	ESCC
I6	D08A0205-B30-C1	Tumor	ESCC
I7	D08A0216-B30-C1	Tumor	ESCC
I8	D08A0285-B30-C1	Tumor	ESCC
I9	D08A0294-B30-C1	Tumor	ESCC
I10	D08A0336-B30-C1	Tumor	ESCC
I11	D08A0388-B30-C1	Tumor	ESCC
I12	D08A0391-B30-C1	Tumor	ESCC
I13	D08A0394-B30-C1	Tumor	ESCC
I14	D08A0395-B30-C1	Tumor	ESCC
I15	D08A0400-B30-C1	Tumor	ESCC
I16	D08A0477-B30-C1	Tumor	ESCC
I17	D08A0510-B30-C1	Tumor	ESCC
I18	D08A0548-B30-C1	Tumor	ESCC
J1	D08A0569-B30-C1	Tumor	ESCC
J2	D08A0582-B30-C1	Tumor	ESCC
J3	D08A0589-B30-C1	Tumor	ESCC
J4	D08A0593-B30-C1	Tumor	ESCC
J5	D08A0595-B30-C1	Tumor	ESCC
J6	D08A0655-B30-C1	Tumor	ESCC
J7	D08A0659-B30-C1	Tumor	ESCC
J8	D08A0660-B30-C1	Tumor	ESCC
J9	D08A0700-B30-C1	Tumor	ESCC
J10	D08A1822-B30-C1	Tumor	ESCC

J11	D08A0720-B30-C1	Tumor	ESCC
J12	D08A0803-B30-C1	Tumor	ESCC
J13	D08A0804-B30-C1	Tumor	ESCC
J14	D08A0724-B30-C1	Tumor	ESCC
J15	D08A0745-B30-C1	Tumor	ESCC
J16	D08A0752-B30-C1	Tumor	ESCC
J17	D08A0807-B30-C1	Tumor	ESCC
J18	D08A0834-B30-C1	Tumor	ESCC
K1	Anchor point		
L1	D08A0276 -B30-C1	Tumor	ESCC
L2	D08A0276 -B30-P1	Normal	Normal esophageal tissue
L3	D08A0276 -B30-L1	Lymph node	Negative lymph node
L4	D08A0464 -B30-C1	Tumor	ESCC
L5	D08A0464 -B30-P1	Normal	Normal esophageal tissue
L6	D08A0464 -B30-L1	Lymph node	Negative lymph node
L7	D08A0469 -B30-C1	Tumor	ESCC
L8	D08A0469 -B30-P1	Normal	Normal esophageal tissue
L9	D08A0469 -B30-L1	Lymph node	Negative lymph node
L10	D08A0509 -B30-C1	Tumor	ESCC
L11	D08A0509 -B30-P4	Normal	Normal esophageal tissue
L12	D08A0509 -B30-L1	Lymph node	Negative lymph node
L13	D08A0631 -B30-C1	Tumor	ESCC
L14	D08A0631 -B30-P1	Normal	Normal esophageal tissue
L15	D08A0631 -B30-L1	Lymph node	Negative lymph node
M1	D08A0634 -B30-C1	Tumor	ESCC
M2	D08A0634 -B30-P1	Normal	Normal esophageal tissue
M3	D08A0634 -B30-L1	Lymph node	Negative lymph node
M4	D08A0636 -B30-C1	Tumor	ESCC
M5	D08A0636 -B30-P1	Normal	Normal esophageal tissue
M6	D08A0636 -B30-L1	Lymph node	Negative lymph node
M7	D08A0687 -B30-C1	Tumor	ESCC
M8	D08A0687 -B30-P1	Normal	Normal esophageal tissue
M9	D08A0687 -B30-L1	Lymph node	Negative lymph node
M10	D08A0726 -B30-C1	Tumor	ESCC
M11	D08A0726 -B30-P1	Normal	Normal esophageal tissue
M12	D08A0726 -B30-L1	Lymph node	Negative lymph node
M13	D08A0730 -B30-C1	Tumor	ESCC
M14	D08A0730 -B30-P1	Normal	Normal esophageal tissue
M15	D08A0730 -B30-L1	Lymph node	Negative lymph node
N1	D08A0540 -B30-C1	Tumor	ESCC
N2	D08A0540 -B30-P1	Normal	Normal esophageal tissue
N3	D08A0540 -B30-L2	Lymph node	Positive lymph node
N4	D08A0550 -B30-C1	Tumor	ESCC
N5	D08A0550 -B30-P1	Normal	Normal esophageal tissue

N6	D08A0550 -B30-L1	Lymph node	Positive lymph node
N7	D08A0611 -B30-C1	Tumor	ESCC
N8	D08A0611 -B30-P1	Normal	Normal esophageal tissue
N9	D08A0611 -B30-L1	Lymph node	Positive lymph node
N10	D08A0988 -B30-C1	Tumor	ESCC
N11	D08A0988 -B30-P1	Normal	Normal esophageal tissue
N12	D08A0988 -B30-L1	Lymph node	Positive lymph node
N13	D08A1041 -B30-C1	Tumor	ESCC
N14	D08A1041 -B30-P1	Normal	Normal esophageal tissue
N15	D08A1041 -B30-L1	Lymph node	Positive lymph node
O1	D08A0598 -B30-C1	Tumor	ESCC
O2	D08A0598 -B30-P1	Normal	Normal esophageal tissue
O3	D08A0598 -B30-L1	Lymph node	Positive lymph node
O4	D08A0667 -B30-C1	Tumor	ESCC
O5	D08A0667 -B30-P1	Normal	Normal esophageal tissue
O6	D08A0667 -B30-L1	Lymph node	Positive lymph node
O7	D08A0733 -B30-C1	Tumor	ESCC
O8	D08A0733 -B30-P1	Normal	Normal esophageal tissue
O9	D08A0733 -B30-L2	Lymph node	Positive lymph node
O10	D08A0637 -B30-C1	Tumor	ESCC
O11	D08A0637 -B30-P1	Normal	Normal esophageal tissue
O12	D08A0637 -B30-L1	Lymph node	Positive lymph node
O13	D08A0971 -B30-C1	Tumor	ESCC
O14	D08A0971 -B30-P1	Normal	Normal esophageal tissue
O15	D08A0971 -B30-L1	Lymph node	Positive lymph node
P1	D08A1118 -B30-C1	Tumor	ESCC
P2	D08A1118 -B30-P1	Normal	Normal esophageal tissue
P3	D08A1118 -B30-L2	Lymph node	Positive lymph node
P4	D08A0535 -B30-C1	Tumor	ESCC
P5	D08A0535 -B30-P1	Normal	Normal esophageal tissue
P6	D08A0535 -B30-L1	Lymph node	Positive lymph node
P7	D08A0735 -B30-C1	Tumor	ESCC
P8	D08A0735 -B30-P1	Normal	Normal esophageal tissue
P9	D08A0735 -B30-L1	Lymph node	Positive lymph node
P10	D08A0639 -B30-C1	Tumor	ESCC
P11	D08A0639 -B30-P1	Normal	Normal esophageal tissue
P12	D08A0639 -B30-L2	Lymph node	Positive lymph node
P13	D08A0716 -B30-P1	Tumor	ESCC
P14	D08A0716 -B30-C1	Normal	Normal esophageal tissue
P15	D08A0716 -B30-L1	Lymph node	Positive lymph node
Q1	D08A0482 -B30-C1	Tumor	ESCC
Q2	D08A0482 -B30-P1	Normal	Normal esophageal tissue
Q3	D08A0482 -B30-L1	Lymph node	Positive lymph node
Q4	D08A0817 -B30-C1	Tumor	ESCC

Q5	D08A0817 -B30-P1	Normal	Normal esophageal tissue
Q6	D08A0817 -B30-L1	Lymph node	Positive lymph node
Q7	D08A0345 -B30-C1	Tumor	ESCC
Q8	D08A0345 -B30-L1	Lymph node	Positive lymph node
Q9	D08A0756 -B30-C1	Tumor	ESCC
Q10	D08A0756 -B30-L1	Lymph node	Positive lymph node
Q11	D08A0948 -B30-C1	Tumor	ESCC
Q12	D08A0948 -B30-L2	Lymph node	Positive lymph node
Q13	D08A0962 -B30-C1	Tumor	ESCC
Q14	D08A0962 -B30-L2	Lymph node	Positive lymph node
Q15	——	——	——
R1	D08A0968 -B30-C1	Tumor	ESCC
R2	D08A0968 -B30-L2	Lymph node	Positive lymph node
R3	D08A1105 -B30-C1	Tumor	ESCC
R4	D08A1105 -B30-L1	Lymph node	Positive lymph node
R5	D08A0156 -B30-C1	Tumor	ESCC
R6	D08A0156 -B30-L2	Lymph node	Positive lymph node
R7	D08A0263 -B30-C1	Tumor	ESCC
R8	D08A0263 -B30-L2	Lymph node	Positive lymph node
R9	D08A0302 -B30-C1	Tumor	ESCC
R10	D08A0302 -B30-L1	Lymph node	Positive lymph node
R11	D08A0442 -B30-C1	Tumor	ESCC
R12	D08A0442 -B30-L1	Lymph node	Positive lymph node
R13	D08A0672 -B30-C1	Tumor	ESCC
R14	D08A0672 -B30-L1	Lymph node	Positive lymph node
R15	——	——	——
S1	D08A0964 -B30-C1	Tumor	ESCC
S2	D08A0964 -B30-L1	Lymph node	Positive lymph node
S3	D08A0601 -B30-P1	Tumor	ESCC
S4	D08A0601 -B30-L2	Lymph node	Positive lymph node
S5	D08A0638 -B30-C1	Tumor	ESCC
S6	D08A0638 -B30-L2	Lymph node	Positive lymph node
S7	D08A0963 -B30-C1	Tumor	ESCC
S8	D08A0963 -B30-L1	Lymph node	Positive lymph node
S9	D08A0801 -B30-C1	Tumor	ESCC
S10	D08A0801 -B30-L2	Lymph node	Positive lymph node
S11	D08A0986 -B30-C1	Tumor	ESCC
S12	D08A0986 -B30-L1	Lymph node	Positive lymph node
S13	D08A1040 -B30-C1	Tumor	ESCC
S14	D08A1040 -B30-L2	Lymph node	Positive lymph node
S15	——	——	——
T1	D08A0864 -B30-C1	Tumor	ESCC
T2	D08A0864 -B30-L1	Lymph node	Positive lymph node
T3	D08A0110 -B30-C1	Tumor	ESCC

T4	D08A0110 -B30-L1	Lymph node	Positive lymph node
T5	D08A0313 -B30-C1	Tumor	ESCC
T6	D08A0313 -B30-L1	Lymph node	Positive lymph node
T7	D08A0538 -B30-C1	Tumor	ESCC
T8	D08A0538 -B30-L1	Lymph node	Positive lymph node
T9	D08A0248 -B30-C1	Tumor	ESCC
T10	D08A0248 -B30-L1	Lymph node	Positive lymph node

Patient information

ID	status	OS	Gender	Age	T	N	M	Pathologic stage
D08A0181	1	1740	Male	70	T3	N0	M0	2A-2B
D08A0184	1	780	Male	73	T3	N1	M0	3B
D08A0185	1	390	Female	74	T3		M0	
D08A0187	0	3210	Male	66	T3	N0	M0	2A-2B
D08A0196	1	1800	Male	78		N1	M0	
D08A0201	1	1080	Male	67				
D08A0203	1	540	Male					
D08A0211	1	30	Male	74				
D08A0214	1	330	Male	29				
D08A0256	1	540	Male	83				
D08A0257	1	120	Male	65				
D08A0260	1	930	Male	75	T3	N0	M0	2A-2B
D08A0284	1	1530	Male	51	T3	N0	M0	2A-2B
D08A0286	1	870	Male	73		N0	M0	
D08A0293	1	1050	Male	51	T3	N2	M0	3B
D08A0373	1	180	Male	64	T3	N3	M0	4A
D08A0376	1	1290	Male	64	T3	N0	M0	2A-2B
D08A0379	1	1320	Male	55	T2	N0	M0	2A
D08A0380	1	240	Male	52	T3	N1	M0	3B
D08A0381	1	270	Male	59	T3	N3	M0	4A
D08A0383	1	480	Male	70	T3	N1	M0	3B
D08A0385	1	780	Male	63	T2	N0	M0	2A
D08A0393	1	300	Female	67	T3	N0	M0	2A-2B
D08A0401	1	1320	Male	53	T3	N1	M0	3B
D08A0402	0	2850	Female	71	T3	N0	M0	2A-2B
D08A0403	1	690	Male	69	T3	N1	M0	3B
D08A0404	0		Male	61	T3	N1	M0	3B
D08A0405	1	120	Male	77	T3	N0	M0	2A-2B
D08A1772	1	930	Male	64	T3	N1	M0	3B
D08A0514	1	390	Male	53				
D08A0515	1	1500	Male	66				
D08A0571	1	450	Female	56	T3	N0	M0	2A-2B
D08A0572	1	390	Female	68	T3	N0	M0	2A-2B
D08A0573	1	60	Male	77	T3	N0	M0	2A-2B
D08A0577	0	2640	Male	57	T2	N1	M0	3A
D08A0579	1	990	Male	61	T3	N0	M0	2A-2B
D08A0580	1	360	Male	68	T3	N3	M0	4A

D08A0581	1	360	Female	72	T3	N0	M0	2A-2B
D08A0583	1	120	Male	74	T3	N0	M0	2A-2B
D08A0584	1	150	Male	72	T2	N2	M0	3B
D08A0588	1	900	Female	65	T3	N0	M0	2A-2B
D08A0594	0	2610	Female	58				
D08A0596	1	660	Female	63	T3	N0	M0	2A-2B
D08A0649	1	0	Male	76	T3	N0	M0	2A-2B
D08A0692	1	450	Male	79				
D08A0694	1	150	Male	72	T3	N1	M0	3B
D08A0696	1	480	Male	54	T3	N1	M0	3B
D08A0697	0	2520	Male	76	T2	N1	M0	3A
D08A0699	1	780	Male	65	T3	N3	M0	4A
D08A1820	1	330	Male	62				
D08A0718	1	150	Male	64	T3	N1	M0	3B
D08A0719	0	2490	Female	71	T1b	N0	M0	1B
D08A0746	0	2460	Female	74	T1b	N0	M0	1B
D08A0753	1	300	Male	52	T3	N1	M0	3B
D08A1837	1	2640	Male	73				
D08A1843	0	2370	Female	56				
D08A0212	1	210	Male	78				
D08A0218	1	180	Male	74				
D08A0334	1	60	Male	72				
D08A0337	1	690	Male	71	T3	N2	M0	3B
D08A0340	1	270	Male	51	T2	N0	M0	2A
D08A0384	0	2910	Male	65	T3	N0	M0	2A-2B
D08A0648	1	150	Female	73	T3	N2	M0	3B
D08A0652	0	2580	Female	79	T3	N0	M0	2A-2B
D08A0702	1	30	Male	81	T3	N1	M0	3B
D08A0701	0	2520	Female	68	T3	N1	M0	3B
D08A0705	0	2490	Female	74	T3	N0	M0	2A-2B
D08A0723	1	750	Female	68	T3	N1	M0	3B
D08A1731	1	570	Male	60	T3	N1	M0	3B
D08A0188	1	450	Male	73	T3	N0	M0	2A-2B
D08A0209	1	150	Male	72				
D08A0213	0	3120	Female	68				
D08A0219	1	60	Male	66				
D08A0290	1	660	Male	63	T3	N1	M0	3B
D08A0651	0	2610	Female	57				
D08A0650	1	150	Male	61	T3	N1	M0	3B

D08A1834	0	2460	Female	67				
D08A0748	0	2430	Male	63	T1a	N0	M0	1B
D08A1844	1	1230	Female	51				
D08A0835	0	2370	Male	69				
D08A0194	1	180	Male	69	T3	N1	M0	3B
D08A0205	1	240	Female	79				
D08A0216	1	180	Male	84				
D08A0285	1	1410	Male	51	T3	N2	M0	3B
D08A0294	1	300	Male	60	T3	N2	M0	3B
D08A0336	0	2910	Female	62				
D08A0388	0	2910	Female	56	T3	N0	M0	2A-2B
D08A0391	1	990	Male	73	T3	N0	M0	2A-2B
D08A0394	1	180	Male	51	T3	N0	M0	2A-2B
D08A0395	1	240	Male	49	T3	N2	M0	3B
D08A0400	1	1290	Male	48	T3	N1	M0	3B
D08A0477	1	270	Male	61	T3	N1	M0	3B
D08A0510	1	360	Male	65				
D08A0548	1	1230	Female	63				
D08A0569	1	390	Male	59	T3	N0	M0	2A-2B
D08A0582	0	2640	Male	57	T3	N0	M0	2A
D08A0589	1	450	Male	65	T3	N1	M0	3B
D08A0593	0	2610	Male	75	T3	N0	M0	2A-2B
D08A0595	1	150	Male	80	T3	N1	M0	3B
D08A0655	1	180	Female	71	T3	N0	M0	2A
D08A0659	1	210	Male	50	T3	N2	M0	3B
D08A0660	1	420	Male	54	T2	N2	M0	3B
D08A0700	0	2520	Female	81	T2	N0	M0	2A
D08A1822	1	360	Male	59	T3	N2	M0	3B
D08A0720	1	120	Male	62	T3	N2	M0	3B
D08A0803	0	2490	Male	52	T2	N1	M0	3A
D08A0804	1	300	Female	74	T3	N0	M0	2A-2B
D08A0724	1	300	Male	74	T3	N2	M0	3B
D08A0745	1	810	Male	79	T3	N1	M0	3B
D08A0752	1	840	Male	65				
D08A0807	1	2100	Female	69				
D08A0834	1	1740	Male	77				

Supplementary file S2:

Proteins quantified by Label-free proteomics

Protein accession	Protein description	Gene name	IFIT3/NC Ratio	IFIT3/NC *P* value
P0DPI2	Glutamine amidotransferase-like class 1 domain-containing protein 3, mitochondrial OS=Homo sapiens OX=9606 GN=GATD3	GATD3	0.857	0.048184
A0AV96	RNA-binding protein 47 OS=Homo sapiens OX=9606 GN=RBM47 PE=1 SV=2	RBM47	0.802	0.024445
A0AVT1	Ubiquitin-like modifier-activating enzyme 6 OS=Homo sapiens OX=9606 GN=UBA6 PE=1 SV=1	UBA6	1.115	0.345233
A0FGR8	Extended synaptotagmin-2 OS=Homo sapiens OX=9606 GN=ESYT2 PE=1 SV=1	ESYT2	1.026	0.470708
A0JLT2	Mediator of RNA polymerase II transcription subunit 19 OS=Homo sapiens OX=9606 GN=MED19 PE=1 SV=2	MED19	0.99	0.956081
A1L0T0	2-hydroxyacyl-CoA lyase 2 OS=Homo sapiens OX=9606 GN=ILVBL PE=1 SV=2	ILVBL	1.141	0.055281
A1L3X0	Elongation of very long chain fatty acids protein 7 OS=Homo sapiens OX=9606 GN=ELOVL7 PE=1 SV=1	ELOVL7	1.024	0.912522
A2RRD8	Zinc finger protein 320 OS=Homo sapiens OX=9606 GN=ZNF320 PE=1 SV=1	ZNF320	1.049	0.230573
A2RRP1	Neuroblastoma-amplified sequence OS=Homo sapiens OX=9606 GN=NBAS PE=1 SV=2	NBAS	1.138	0.151396
A3KMH1	von Willebrand factor A domain-containing protein 8 OS=Homo sapiens OX=9606 GN=VWA8 PE=1 SV=2	VWA8	0.982	0.728943
A4D1E9	GTP-binding protein 10 OS=Homo sapiens OX=9606 GN=GTPBP10 PE=1 SV=1	GTPBP10	0.914	0.447828
A5PLL7	Plasmanylethanolamine desaturase OS=Homo sapiens OX=9606 GN=PEDS1 PE=1 SV=3	PEDS1	1.033	0.852998
A5YKK6	CCR4-NOT transcription complex subunit 1 OS=Homo sapiens OX=9606 GN=CNOT1 PE=1	CNOT1	0.984	0.798658
A6NFI3	Zinc finger protein 316 OS=Homo sapiens OX=9606 GN=ZNF316 PE=1 SV=1	ZNF316	0.631	

A6NHR9	Structural maintenance of chromosomes flexible hinge domain-containing protein 1 OS=Homo sapiens OX=9606 GN=SMCHD1 PE=1 SV=2	SMCHD1	0.856	0.077756
P0DN87	Choriogonadotropin subunit beta 7 OS=Homo sapiens OX=9606 GN=CGB7 PE=2 SV=1	CGB7	1.02	0.792905
A8CG34	Nuclear envelope pore membrane protein POM 121C OS=Homo sapiens OX=9606 GN=POM121C PE=1 SV=3	POM121C	0.995	0.911761
A8MXV4	Acyl-coenzyme A diphosphatase NUDT19 OS=Homo sapiens OX=9606 GN=NUDT19 PE=1 SV=1	NUDT19	1.04	0.476407
E7ERA6	RING finger protein 223 OS=Homo sapiens OX=9606 GN=RNF223 PE=2 SV=1	RNF223	0.821	
E9PAV3	Nascent polypeptide-associated complex subunit alpha, muscle-specific form OS=Homo sapiens OX=9606 GN=NACA PE=1 SV=1	NACA	0.929	0.628065
E9PRG8	Uncharacterized protein C11orf98 OS=Homo sapiens OX=9606 GN=C11orf98 PE=4 SV=2	C11orf98	0.896	0.497288
L0R6Q1	SLC35A4 upstream open reading frame protein OS=Homo sapiens OX=9606 GN=SLC35A4 PE=3 SV=1	SLC35A4	0.989	0.865397
O00116	Alkyldihydroxyacetonephosphate synthase, peroxisomal OS=Homo sapiens OX=9606 GN=AGPS PE=1 SV=1	AGPS	0.904	0.291598
O00139	Kinesin-like protein KIF2A OS=Homo sapiens OX=9606 GN=KIF2A PE=1 SV=3	KIF2A	1.12	0.509811
O00148	ATP-dependent RNA helicase DDX39A OS=Homo sapiens OX=9606 GN=DDX39A PE=1 SV=2	DDX39A	0.833	0.089464
O00151	PDZ and LIM domain protein 1 OS=Homo sapiens OX=9606 GN=PDLIM1 PE=1 SV=4	PDLIM1	1.071	0.632647
O00154	Cytosolic acyl coenzyme A thioester hydrolase OS=Homo sapiens OX=9606 GN=ACOT7	ACOT7	0.872	0.282769
O00159	Unconventional myosin-Ic OS=Homo sapiens OX=9606 GN=MYO1C PE=1 SV=4	MYO1C	0.959	0.564531
O00161	Synaptosomal-associated protein 23 OS=Homo sapiens OX=9606 GN=SNAP23 PE=1 SV=1	SNAP23	0.996	0.962613

O00165	HCLS1-associated protein X-1 OS=Homo sapiens OX=9606 GN=HAX1 PE=1 SV=2	HAX1	0.989	0.874077
O00170	AH receptor-interacting protein OS=Homo sapiens OX=9606 GN=AIP PE=1 SV=2	AIP	0.972	0.766269
O00186	Syntaxin-binding protein 3 OS=Homo sapiens OX=9606 GN=STXBP3 PE=1 SV=2	STXBP3	0.952	0.112727
O00193	Small acidic protein OS=Homo sapiens OX=9606 GN=SMAP PE=1 SV=1	SMAP	0.807	0.306889
O00203	AP-3 complex subunit beta-1 OS=Homo sapiens OX=9606 GN=AP3B1 PE=1 SV=3	AP3B1	1.028	0.751441
O00214	Galectin-8 OS=Homo sapiens OX=9606 GN=LGALS8 PE=1 SV=4	LGALS8	0.91	0.495341
O00217	NADH dehydrogenase [ubiquinone] iron-sulfur protein 8, mitochondrial OS=Homo sapiens OX=9606 GN=NDUFS8 PE=1	NDUFS8	0.995	0.891156
O00231	26S proteasome non-ATPase regulatory subunit 11 OS=Homo sapiens OX=9606 GN=PSMD11 PE=1 SV=3	PSMD11	1.005	0.957083
O00232	26S proteasome non-ATPase regulatory subunit 12 OS=Homo sapiens OX=9606 GN=PSMD12 PE=1 SV=3	PSMD12	1.034	0.051379
O00233	26S proteasome non-ATPase regulatory subunit 9 OS=Homo sapiens OX=9606 GN=PSMD9 PE=1 SV=3	PSMD9	1.388	
O00244	Copper transport protein ATOX1 OS=Homo sapiens OX=9606 GN=ATOX1 PE=1 SV=1	ATOX1	1.121	
O00255	Menin OS=Homo sapiens OX=9606 GN=MEN1 PE=1 SV=4	MEN1	0.861	0.581839
O00264	Membrane-associated progesterone receptor component 1 OS=Homo sapiens OX=9606 GN=PGRMC1 PE=1 SV=3	PGRMC1	1.076	0.293928
O00267	Transcription elongation factor SPT5 OS=Homo sapiens OX=9606 GN=SUPT5H PE=1	SUPT5H	0.929	0.054235
O00273	DNA fragmentation factor subunit alpha OS=Homo sapiens OX=9606 GN=DFFA PE=1 SV=1	DFFA	0.992	0.868725
O00291	Huntingtin-interacting protein 1 OS=Homo sapiens OX=9606 GN=HIP1 PE=1 SV=5	HIP1	0.95	0.401126

O00299	Chloride intracellular channel protein 1 OS=Homo sapiens OX=9606 GN=CLIC1 PE=1 SV=4	CLIC1	1.005	0.977314
O00303	Eukaryotic translation initiation factor 3 subunit F OS=Homo sapiens OX=9606 GN=EIF3F	EIF3F	0.983	0.774308
O00330	Pyruvate dehydrogenase protein X component, mitochondrial OS=Homo sapiens OX=9606 GN=PDHX PE=1 SV=3	PDHX	0.919	0.578368
O00391	Sulfhydryl oxidase 1 OS=Homo sapiens OX=9606 GN=QSOX1 PE=1 SV=3	QSOX1	1.365	0.031667
O00400	Acetyl-coenzyme A transporter 1 OS=Homo sapiens OX=9606 GN=SLC33A1 PE=1 SV=1	SLC33A1	1.038	0.73584
O00410	Importin-5 OS=Homo sapiens OX=9606 GN=IPO5 PE=1 SV=4	IPO5	1.133	0.176137
O00411	DNA-directed RNA polymerase, mitochondrial OS=Homo sapiens OX=9606 GN=POLRMT PE=1 SV=2	POLRMT	1.31	0.072201
O00422	Histone deacetylase complex subunit SAP18 OS=Homo sapiens OX=9606 GN=SAP18 PE=1 SV=1	SAP18	0.99	0.730852
O00425	Insulin-like growth factor 2 mRNA-binding protein 3 OS=Homo sapiens OX=9606 GN=IGF2BP3 PE=1 SV=2	IGF2BP3	0.908	0.147803
O00429	Dynamin-1-like protein OS=Homo sapiens OX=9606 GN=DNM1L PE=1 SV=2	DNM1L	0.881	0.260207
O00442	RNA 3'-terminal phosphate cyclase OS=Homo sapiens OX=9606 GN=RTCA PE=1 SV=1	RTCA	0.924	0.54982
O00443	Phosphatidylinositol 4-phosphate 3-kinase C2 domain-containing subunit alpha OS=Homo sapiens OX=9606 GN=PIK3C2A PE=1 SV=2	PIK3C2A	1.104	0.277072
O00461	Golgi integral membrane protein 4 OS=Homo sapiens OX=9606 GN=GOLIM4 PE=1 SV=1	GOLIM4	0.91	0.306541
O00468	Agrin OS=Homo sapiens OX=9606 GN=AGRN PE=1 SV=6	AGRN	0.895	0.083298
O00469	Procollagen-lysine,2-oxoglutarate 5-dioxygenase 2 OS=Homo sapiens OX=9606 GN=PLOD2	PLOD2	1.02	0.761414
O00471	Exocyst complex component 5 OS=Homo sapiens OX=9606 GN=EXOC5 PE=1 SV=1	EXOC5	0.832	

O00483	Cytochrome c oxidase subunit NDUFA4 OS=Homo sapiens OX=9606 GN=NDUFA4 PE=1 SV=1	NDUFA4	1.018	0.635312
O00487	26S proteasome non-ATPase regulatory subunit 14 OS=Homo sapiens OX=9606 GN=PSMD14 PE=1 SV=1	PSMD14	1.016	0.820423
O00488	Zinc finger protein 593 OS=Homo sapiens OX=9606 GN=ZNF593 PE=1 SV=2	ZNF593	0.814	0.190252
O00505	Importin subunit alpha-4 OS=Homo sapiens OX=9606 GN=KPNA3 PE=1 SV=2	KPNA3	0.985	0.728686
O00515	Ladinin-1 OS=Homo sapiens OX=9606 GN=LAD1 PE=1 SV=2	LAD1	1.019	0.449609
O00541	Pescadillo homolog OS=Homo sapiens OX=9606 GN=PES1 PE=1 SV=1	PES1	0.976	0.800837
O00560	Syntenin-1 OS=Homo sapiens OX=9606 GN=SDCBP PE=1	SDCBP	1.043	0.432962
O00566	U3 small nucleolar ribonucleoprotein protein MPP10 OS=Homo sapiens OX=9606 GN=MPHOSPH10 PE=1 SV=2	MPHOSPH 10	0.886	0.145597
O00567	Nucleolar protein 56 OS=Homo sapiens OX=9606 GN=NOP56 PE=1 SV=4	NOP56	1.019	0.97918
O00571	ATP-dependent RNA helicase DDX3X OS=Homo sapiens OX=9606 GN=DDX3X PE=1	DDX3X	1.047	0.160636
O00592	Podocalyxin OS=Homo sapiens OX=9606 GN=PODXL PE=1	PODXL	1.181	0.193012
O00622	CCN family member 1 OS=Homo sapiens OX=9606 GN=CCN1 PE=1 SV=1	CCN1	1.174	0.230273
O00629	Importin subunit alpha-3 OS=Homo sapiens OX=9606 GN=KPNA4 PE=1 SV=1	KPNA4	1.076	0.023452
O00743	Serine/threonine-protein phosphatase 6 catalytic subunit OS=Homo sapiens OX=9606 GN=PPP6C PE=1 SV=1	PPP6C	1.183	0.371388
O00754	Lysosomal alpha-mannosidase OS=Homo sapiens OX=9606 GN=MAN2B1 PE=1 SV=3	MAN2B1	1.04	0.770045
O00762	Ubiquitin-conjugating enzyme E2 C OS=Homo sapiens OX=9606 GN=UBE2C PE=1 SV=1	UBE2C	1.065	0.661913
O00764	Pyridoxal kinase OS=Homo sapiens OX=9606 GN=PDXK	PDXK	1.108	0.502703

O00767	Stearoyl-CoA desaturase OS=Homo sapiens OX=9606 GN=SCD PE=1 SV=2	SCD	0.881	0.512351
O14493	Claudin-4 OS=Homo sapiens OX=9606 GN=CLDN4 PE=1	CLDN4	1.149	0.224585
O14494	Phospholipid phosphatase 1 OS=Homo sapiens OX=9606 GN=PLPP1 PE=1 SV=1	PLPP1	0.882	0.457983
O14497	AT-rich interactive domain-containing protein 1A OS=Homo sapiens OX=9606 GN=ARID1A PE=1 SV=3	ARID1A	0.982	0.760831
O14519	Cyclin-dependent kinase 2-associated protein 1 OS=Homo sapiens OX=9606 GN=CDK2AP1 PE=1 SV=1	CDK2AP1	1.004	0.909202
O14524	Nuclear envelope integral membrane protein 1 OS=Homo sapiens OX=9606 GN=NEMP1	NEMP1	0.765	0.73985
O14530	Thioredoxin domain-containing protein 9 OS=Homo sapiens OX=9606 GN=TXNDC9 PE=1 SV=2	TXNDC9	1.216	0.227543
O14548	Cytochrome c oxidase subunit 7A-related protein, mitochondrial OS=Homo sapiens OX=9606 GN=COX7A2L PE=1 SV=2	COX7A2L	0.912	0.185542
O14561	Acyl carrier protein, mitochondrial OS=Homo sapiens OX=9606 GN=NDUFAB1 PE=1 SV=3	NDUFAB1	0.933	0.298792
O14579	Coatomer subunit epsilon OS=Homo sapiens OX=9606 GN=COPE PE=1 SV=3	COPE	1.077	0.616316
O14617	AP-3 complex subunit delta-1 OS=Homo sapiens OX=9606 GN=AP3D1 PE=1 SV=1	AP3D1	1.218	0.032604
O14639	Actin-binding LIM protein 1 OS=Homo sapiens OX=9606 GN=ABLIM1 PE=1 SV=3	ABLIM1	1.009	0.99208
O14646	Chromodomain-helicase-DNA-binding protein 1 OS=Homo sapiens OX=9606 GN=CHD1	CHD1	0.803	0.176151
O14653	Golgi SNAP receptor complex member 2 OS=Homo sapiens OX=9606 GN=GOSR2 PE=1	GOSR2	1.153	0.102893
O14656	Torsin-1A OS=Homo sapiens OX=9606 GN=TOR1A PE=1	TOR1A	1.138	0.211319
O14662	Syntaxin-16 OS=Homo sapiens OX=9606 GN=STX16 PE=1	STX16	0.964	0.657529
O14672	Disintegrin and metalloproteinase domain-containing protein 10 OS=Homo sapiens OX=9606 GN=ADAM10 PE=1 SV=1	ADAM10	0.98	0.797696

O14681	Etoposide-induced protein 2.4 homolog OS=Homo sapiens OX=9606 GN=EI24 PE=1 SV=4	EI24	1.146	0.020656
O14684	Prostaglandin E synthase OS=Homo sapiens OX=9606 GN=PTGES PE=1 SV=2	PTGES	1.117	0.333793
O14734	Acyl-coenzyme A thioesterase 8 OS=Homo sapiens OX=9606 GN=ACOT8 PE=1 SV=1	ACOT8	1.016	0.921919
O14735	CDP-diacylglycerol--inositol 3-phosphatidyltransferase OS=Homo sapiens OX=9606 GN=CDIPT PE=1 SV=1	CDIPT	1.066	0.241123
O14737	Programmed cell death protein 5 OS=Homo sapiens OX=9606 GN=PDCD5 PE=1 SV=3	PDCD5	1.102	0.577837
O14744	Protein arginine N-methyltransferase 5 OS=Homo sapiens OX=9606 GN=PRMT5	PRMT5	1.101	0.461682
O14763	Tumor necrosis factor receptor superfamily member 10B OS=Homo sapiens OX=9606 GN=TNFRSF10B PE=1 SV=2	TNFRSF10B	0.942	
O14770	Homeobox protein Meis2 OS=Homo sapiens OX=9606 GN=MEIS2 PE=1 SV=2	MEIS2	0.712	0.239657
O14773	Tripeptidyl-peptidase 1 OS=Homo sapiens OX=9606 GN=TPP1 PE=1 SV=2	TPP1	1.004	
O14776	Transcription elongation regulator 1 OS=Homo sapiens OX=9606 GN=TCERG1 PE=1 SV=2	TCERG1	0.886	0.028117
O14786	Neuropilin-1 OS=Homo sapiens OX=9606 GN=NRP1 PE=1 SV=3	NRP1	1.221	0.3427
O14787	Transportin-2 OS=Homo sapiens OX=9606 GN=TNPO2 PE=1	TNPO2	1.083	
O14818	Proteasome subunit alpha type-7 OS=Homo sapiens OX=9606 GN=PSMA7 PE=1 SV=1	PSMA7	0.98	0.733734
O14828	Secretory carrier-associated membrane protein 3 OS=Homo sapiens OX=9606 GN=SCAMP3 PE=1 SV=3	SCAMP3	0.885	0.324372
O14874	[3-methyl-2-oxobutanoate dehydrogenase [lipoamide]] kinase, mitochondrial OS=Homo sapiens OX=9606 GN=BCKDK	BCKDK	1.138	0.443509
O14879	Interferon-induced protein with tetratricopeptide repeats 3 OS=Homo sapiens OX=9606 GN=IFIT3 PE=1 SV=1	IFIT3	1000	0.001

O14880	Microsomal glutathione S-transferase 3 OS=Homo sapiens OX=9606 GN=MGST3 PE=1	MGST3	1.134	0.022591
O14896	Interferon regulatory factor 6 OS=Homo sapiens OX=9606 GN=IRF6 PE=1 SV=1	IRF6	0.99	0.861907
O14908	PDZ domain-containing protein GIPC1 OS=Homo sapiens OX=9606 GN=GIPC1 PE=1 SV=2	GIPC1	0.854	0.009532
O14925	Mitochondrial import inner membrane translocase subunit Tim23 OS=Homo sapiens OX=9606 GN=TIMM23 PE=1	TIMM23	1.26	0.181194
O14929	Histone acetyltransferase type B catalytic subunit OS=Homo sapiens OX=9606 GN=HAT1	HAT1	0.824	0.088133
O14936	Peripheral plasma membrane protein CASK OS=Homo sapiens OX=9606 GN=CASK PE=1 SV=3	CASK	0.85	0.001437
O14949	Cytochrome b-c1 complex subunit 8 OS=Homo sapiens OX=9606 GN=UQCRQ PE=1 SV=4	UQCRQ	0.883	0.047253
P19105	Myosin regulatory light chain 12A OS=Homo sapiens OX=9606 GN=MYL12A PE=1 SV=2	MYL12A	1.244	0.162791
O14964	Hepatocyte growth factor-regulated tyrosine kinase substrate OS=Homo sapiens OX=9606 GN=HGS PE=1 SV=1	HGS	1.06	0.247426
O14965	Aurora kinase A OS=Homo sapiens OX=9606 GN=AURKA	AURKA	0.936	0.762442
O14974	Protein phosphatase 1 regulatory subunit 12A OS=Homo sapiens OX=9606 GN=PPP1R12A PE=1 SV=1	PPP1R12A	1.184	0.041171
O14975	Very long-chain acyl-CoA synthetase OS=Homo sapiens OX=9606 GN=SLC27A2 PE=1 SV=2	SLC27A2	1.059	0.547643
O14979	Heterogeneous nuclear ribonucleoprotein D-like OS=Homo sapiens OX=9606 GN=HNRNPDL PE=1 SV=3	HNRNPDL	0.968	0.544798
O14980	Exportin-1 OS=Homo sapiens OX=9606 GN=XPO1 PE=1 SV=1	XPO1	1.015	0.882946
O15020	Spectrin beta chain, non-erythrocytic 2 OS=Homo sapiens OX=9606 GN=SPTBN2 PE=1 SV=3	SPTBN2	0.933	0.120746
O15027	Protein transport protein Sec16A OS=Homo sapiens OX=9606 GN=SEC16A PE=1 SV=4	SEC16A	1.076	0.426528

O15031	Plexin-B2 OS=Homo sapiens OX=9606 GN=PLXNB2 PE=1 SV=3	PLXNB2	0.985	0.793045
O15042	U2 snRNP-associated SURP motif-containing protein OS=Homo sapiens OX=9606 GN=U2SURP PE=1 SV=2	U2SURP	0.953	0.355908
O15067	Phosphoribosylformylglycinamidine synthase OS=Homo sapiens OX=9606 GN=PFAS PE=1 SV=4	PFAS	0.919	0.222861
O15091	Mitochondrial ribonuclease P catalytic subunit OS=Homo sapiens OX=9606 GN=PRORP	PRORP	0.96	0.500656
O15118	NPC intracellular cholesterol transporter 1 OS=Homo sapiens OX=9606 GN=NPC1 PE=1 SV=2	NPC1	1.019	0.408751
O15120	1-acyl-sn-glycerol-3-phosphate acyltransferase beta OS=Homo sapiens OX=9606 GN=AGPAT2 PE=1 SV=1	AGPAT2	1.063	0.668944
O15126	Secretory carrier-associated membrane protein 1 OS=Homo sapiens OX=9606 GN=SCAMP1 PE=1 SV=2	SCAMP1	0.986	0.884992
O15127	Secretory carrier-associated membrane protein 2 OS=Homo sapiens OX=9606 GN=SCAMP2 PE=1 SV=2	SCAMP2	0.935	0.239003
O15143	Actin-related protein 2/3 complex subunit 1B OS=Homo sapiens OX=9606 GN=ARPC1B PE=1 SV=3	ARPC1B	1.012	0.823409
O15144	Actin-related protein 2/3 complex subunit 2 OS=Homo sapiens OX=9606 GN=ARPC2 PE=1	ARPC2	1.065	0.214841
O15145	Actin-related protein 2/3 complex subunit 3 OS=Homo sapiens OX=9606 GN=ARPC3 PE=1	ARPC3	1.005	0.904907
O15155	BET1 homolog OS=Homo sapiens OX=9606 GN=BET1 PE=1 SV=1	BET1	1.205	0.329242
O15160	DNA-directed RNA polymerases I and III subunit RPAC1 OS=Homo sapiens OX=9606 GN=POLR1C PE=1 SV=1	POLR1C	1.036	0.514867
O15162	Phospholipid scramblase 1 OS=Homo sapiens OX=9606 GN=PLSCR1 PE=1 SV=1	PLSCR1	1.059	0.393286
O15164	Transcription intermediary factor 1-alpha OS=Homo sapiens OX=9606 GN=TRIM24 PE=1	TRIM24	0.868	0.059859

O15173	Membrane-associated progesterone receptor component 2 OS=Homo sapiens OX=9606 GN=PGRMC2 PE=1 SV=1	PGRMC2	0.969	0.628443
O15212	Prefoldin subunit 6 OS=Homo sapiens OX=9606 GN=PFDN6 PE=1 SV=1	PFDN6	1.002	0.968665
O15213	WD repeat-containing protein 46 OS=Homo sapiens OX=9606 GN=WDR46 PE=1 SV=3	WDR46	1.089	0.681626
O15226	NF-kappa-B-repressing factor OS=Homo sapiens OX=9606 GN=NKRF PE=1 SV=2	NKRF	0.964	0.524184
O15228	Dihydroxyacetone phosphate acyltransferase OS=Homo sapiens OX=9606 GN=GNPAT PE=1	GNPAT	1.089	0.414137
O15230	Laminin subunit alpha-5 OS=Homo sapiens OX=9606 GN=LAMA5 PE=1 SV=8	LAMA5	0.956	0.831926
O15231	Zinc finger protein 185 OS=Homo sapiens OX=9606 GN=ZNF185 PE=1 SV=3	ZNF185	0.996	0.934035
O15235	28S ribosomal protein S12, mitochondrial OS=Homo sapiens OX=9606 GN=MRPS12 PE=1 SV=1	MRPS12	0.815	0.348222
O15239	NADH dehydrogenase [ubiquinone] 1 alpha subcomplex subunit 1 OS=Homo sapiens OX=9606 GN=NDUFA1 PE=1	NDUFA1	1.104	0.552757
O15258	Protein RER1 OS=Homo sapiens OX=9606 GN=RER1 PE=1 SV=1	RER1	0.894	0.313035
O15260	Surfeit locus protein 4 OS=Homo sapiens OX=9606 GN=SURF4 PE=1 SV=3	SURF4	1.124	0.219599
O15269	Serine palmitoyltransferase 1 OS=Homo sapiens OX=9606 GN=SPTLC1 PE=1 SV=1	SPTLC1	1.056	0.367914
O15270	Serine palmitoyltransferase 2 OS=Homo sapiens OX=9606 GN=SPTLC2 PE=1 SV=1	SPTLC2	1.011	0.930972
O15294	UDP-N-acetylglucosamine--peptide N-acetylglucosaminyltransferase 110 kDa subunit OS=Homo sapiens	OGT	1.012	0.916716
O15305	Phosphomannomutase 2 OS=Homo sapiens OX=9606 GN=PMM2 PE=1 SV=1	PMM2	1.147	0.180974
O15321	Transmembrane 9 superfamily member 1 OS=Homo sapiens OX=9606 GN=TM9SF1 PE=2	TM9SF1	0.971	0.76105

O15327	Inositol polyphosphate 4-phosphatase type II OS=Homo sapiens OX=9606 GN=INPP4B PE=1 SV=4	INPP4B	1.248	0.176434
O15347	High mobility group protein B3 OS=Homo sapiens OX=9606 GN=HMGB3 PE=1 SV=4	HMGB3	0.706	0.057321
O15355	Protein phosphatase 1G OS=Homo sapiens OX=9606 GN=PPM1G PE=1 SV=1	PPM1G	0.835	0.169592
O15371	Eukaryotic translation initiation factor 3 subunit D OS=Homo sapiens OX=9606 GN=EIF3D PE=1 SV=1	EIF3D	1.073	0.429435
O15372	Eukaryotic translation initiation factor 3 subunit H OS=Homo sapiens OX=9606 GN=EIF3H PE=1 SV=1	EIF3H	1.078	0.095475
O15379	Histone deacetylase 3 OS=Homo sapiens OX=9606 GN=HDAC3 PE=1 SV=2	HDAC3	1.043	0.651444
O15381	Nuclear valosin-containing protein-like OS=Homo sapiens OX=9606 GN=NVL PE=1 SV=1	NVL	1.321	
O15382	Branched-chain-amino-acid aminotransferase, mitochondrial OS=Homo sapiens OX=9606 GN=BCAT2 PE=1 SV=2	BCAT2	0.992	0.924616
O15397	Importin-8 OS=Homo sapiens OX=9606 GN=IPO8 PE=1 SV=2	IPO8	1.168	0.18019
O15400	Syntaxin-7 OS=Homo sapiens OX=9606 GN=STX7 PE=1 SV=4	STX7	1.041	0.695201
O15427	Monocarboxylate transporter 4 OS=Homo sapiens OX=9606 GN=SLC16A3 PE=1 SV=1	SLC16A3	0.979	0.884115
O15439	ATP-binding cassette sub-family C member 4 OS=Homo sapiens OX=9606 GN=ABCC4 PE=1	ABCC4	1.009	0.892987
O15440	ATP-binding cassette sub-family C member 5 OS=Homo sapiens OX=9606 GN=ABCC5 PE=1	ABCC5	0.977	0.698182
O15446	DNA-directed RNA polymerase I subunit RPA34 OS=Homo sapiens OX=9606 GN=POLR1G PE=1 SV=1	POLR1G	0.97	0.688928
O15460	Prolyl 4-hydroxylase subunit alpha-2 OS=Homo sapiens OX=9606 GN=P4HA2 PE=1	P4HA2	0.936	0.340926
O15498	Synaptobrevin homolog YKT6 OS=Homo sapiens OX=9606 GN=YKT6 PE=1 SV=1	YKT6	1.108	0.516501

O15511	Actin-related protein 2/3 complex subunit 5 OS=Homo sapiens OX=9606 GN=ARPC5 PE=1	ARPC5	1.142	0.302852
O15514	DNA-directed RNA polymerase II subunit RPB4 OS=Homo sapiens OX=9606 GN=POLR2D PE=1 SV=1	POLR2D	1.035	0.747077
O15533	Tapasin OS=Homo sapiens OX=9606 GN=TAPBP PE=1	TAPBP	0.965	0.635281
O15554	Intermediate conductance calcium-activated potassium channel protein 4 OS=Homo sapiens OX=9606 GN=KCNN4 PE=1	KCNN4	0.819	0.038817
O43143	Pre-mRNA-splicing factor ATP-dependent RNA helicase DHX15 OS=Homo sapiens OX=9606 GN=DHX15 PE=1 SV=2	DHX15	0.879	0.168597
O43148	mRNA cap guanine-N7 methyltransferase OS=Homo sapiens OX=9606 GN=RNMT	RNMT	0.826	0.168279
O43156	TELO2-interacting protein 1 homolog OS=Homo sapiens OX=9606 GN=TTI1 PE=1 SV=3	TTI1	1.236	0.096344
O43159	Ribosomal RNA-processing protein 8 OS=Homo sapiens OX=9606 GN=RRP8 PE=1 SV=2	RRP8	1.05	0.497065
O43166	Signal-induced proliferation-associated 1-like protein 1 OS=Homo sapiens OX=9606 GN=SIPA1L1 PE=1 SV=4	SIPA1L1	0.928	
O43169	Cytochrome b5 type B OS=Homo sapiens OX=9606 GN=CYB5B PE=1 SV=3	CYB5B	0.989	0.801143
O43172	U4/U6 small nuclear ribonucleoprotein Prp4 OS=Homo sapiens OX=9606 GN=PRPF4 PE=1 SV=2	PRPF4	0.87	0.00597
O43175	D-3-phosphoglycerate dehydrogenase OS=Homo sapiens OX=9606 GN=PHGDH PE=1	PHGDH	1.144	0.245364
O43181	NADH dehydrogenase [ubiquinone] iron-sulfur protein 4, mitochondrial OS=Homo sapiens OX=9606 GN=NDUFS4 PE=1	NDUFS4	1.004	0.94723
O43237	Cytoplasmic dynein 1 light intermediate chain 2 OS=Homo sapiens OX=9606 GN=DYNC1LI2 PE=1 SV=1	DYNC1LI2	1.195	0.160397
O43242	26S proteasome non-ATPase regulatory subunit 3 OS=Homo sapiens OX=9606 GN=PSMD3 PE=1 SV=2	PSMD3	1.03	0.593155

O43251	RNA binding protein fox-1 homolog 2 OS=Homo sapiens OX=9606 GN=RBFOX2 PE=1	RBFOX2	1.07	0.731691
O43252	Bifunctional 3'-phosphoadenosine 5'-phosphosulfate synthase 1 OS=Homo sapiens OX=9606 GN=PAPSS1 PE=1 SV=2	PAPSS1	0.943	0.691411
O43264	Centromere/kinetochore protein zw10 homolog OS=Homo sapiens OX=9606 GN=ZW10 PE=1 SV=3	ZW10	1.032	0.651657
O43278	Kunitz-type protease inhibitor 1 OS=Homo sapiens OX=9606 GN=SPINT1 PE=1 SV=2	SPINT1	0.934	0.22056
O43286	Beta-1,4-galactosyltransferase 5 OS=Homo sapiens OX=9606 GN=B4GALT5 PE=1 SV=1	B4GALT5	0.999	0.935757
O43290	U4/U6.U5 tri-snRNP-associated protein 1 OS=Homo sapiens OX=9606 GN=SART1 PE=1	SART1	0.961	0.323366
O43291	Kunitz-type protease inhibitor 2 OS=Homo sapiens OX=9606 GN=SPINT2 PE=1 SV=2	SPINT2	1.159	0.170513
O43292	Glycosylphosphatidylinositol anchor attachment 1 protein OS=Homo sapiens OX=9606 GN=GPAA1 PE=1 SV=3	GPAA1	0.979	0.797058
O43324	Eukaryotic translation elongation factor 1 epsilon-1 OS=Homo sapiens OX=9606 GN=EEF1E1	EEF1E1	0.987	0.82429
O43390	Heterogeneous nuclear ribonucleoprotein R OS=Homo sapiens OX=9606 GN=HNRNPR PE=1 SV=1	HNRNPR	0.968	0.499503
O43395	U4/U6 small nuclear ribonucleoprotein Prp3 OS=Homo sapiens OX=9606 GN=PRPF3 PE=1 SV=2	PRPF3	0.798	0.020474
O43396	Thioredoxin-like protein 1 OS=Homo sapiens OX=9606 GN=TXNL1 PE=1 SV=3	TXNL1	0.902	0.371326
O43399	Tumor protein D54 OS=Homo sapiens OX=9606 GN=TPD52L2 PE=1 SV=2	TPD52L2	1.229	0.010892
O43402	ER membrane protein complex subunit 8 OS=Homo sapiens OX=9606 GN=EMC8 PE=1 SV=1	EMC8	1.068	0.285286
O43432	Eukaryotic translation initiation factor 4 gamma 3 OS=Homo sapiens OX=9606 GN=EIF4G3	EIF4G3	1.078	0.67124
O43447	Peptidyl-prolyl cis-trans isomerase H OS=Homo sapiens OX=9606 GN=PPIH PE=1 SV=1	PPIH	0.938	0.544599

O43464	Serine protease HTRA2, mitochondrial OS=Homo sapiens OX=9606 GN=HTRA2 PE=1	HTRA2	0.892	0.223133
O43488	Aflatoxin B1 aldehyde reductase member 2 OS=Homo sapiens OX=9606 GN=AKR7A2 PE=1 SV=3	AKR7A2	0.847	
O43491	Band 4.1-like protein 2 OS=Homo sapiens OX=9606 GN=EPB41L2 PE=1 SV=1	EPB41L2	0.802	0.003664
O43493	Trans-Golgi network integral membrane protein 2 OS=Homo sapiens OX=9606 GN=TGOLN2 PE=1 SV=4	TGOLN2	0.94	0.495568
O43504	Ragulator complex protein LAMTOR5 OS=Homo sapiens OX=9606 GN=LAMTOR5 PE=1 SV=1	LAMTOR5	1.086	0.383787
O43556	Epsilon-sarcoglycan OS=Homo sapiens OX=9606 GN=SGCE PE=1 SV=6	SGCE	0.94	0.29552
O43583	Density-regulated protein OS=Homo sapiens OX=9606 GN=DENR PE=1 SV=2	DENR	0.987	0.921271
O43592	Exportin-T OS=Homo sapiens OX=9606 GN=XPOT PE=1 SV=2	XPOT	1.076	0.260378
O43615	Mitochondrial import inner membrane translocase subunit TIM44 OS=Homo sapiens OX=9606 GN=TIMM44 PE=1	TIMM44	0.97	0.72467
O43617	Trafficking protein particle complex subunit 3 OS=Homo sapiens OX=9606 GN=TRAPPC3 PE=1 SV=1	TRAPPC3	1.161	0.156079
O43633	Charged multivesicular body protein 2a OS=Homo sapiens OX=9606 GN=CHMP2A PE=1	CHMP2A	1.053	0.528784
O43657	Tetraspanin-6 OS=Homo sapiens OX=9606 GN=TSPAN6 PE=1 SV=1	TSPAN6	1.035	0.702767
O43660	Pleiotropic regulator 1 OS=Homo sapiens OX=9606 GN=PLRG1 PE=1 SV=1	PLRG1	0.912	0.048951
O43663	Protein regulator of cytokinesis 1 OS=Homo sapiens OX=9606 GN=PRC1 PE=1 SV=2	PRC1	1.089	0.268033
O43670	BUB3-interacting and GLEBS motif-containing protein ZNF207 OS=Homo sapiens OX=9606 GN=ZNF207 PE=1 SV=1	ZNF207	0.833	0.021343

O43674	NADH dehydrogenase [ubiquinone] 1 beta subcomplex subunit 5, mitochondrial OS=Homo sapiens OX=9606 GN=NDUFB5 PE=1 SV=1	NDUFB5	1.026	0.810071
O43676	NADH dehydrogenase [ubiquinone] 1 beta subcomplex subunit 3 OS=Homo sapiens OX=9606 GN=NDUFB3 PE=1	NDUFB3	0.91	0.125258
O43677	NADH dehydrogenase [ubiquinone] 1 subunit C1, mitochondrial OS=Homo sapiens OX=9606 GN=NDUFC1 PE=1	NDUFC1	0.874	0.413502
O43678	NADH dehydrogenase [ubiquinone] 1 alpha subcomplex subunit 2 OS=Homo sapiens OX=9606 GN=NDUFA2 PE=1	NDUFA2	1.053	0.407297
O43681	ATPase GET3 OS=Homo sapiens OX=9606 GN=GET3 PE=1 SV=2	GET3	1.012	0.879345
O43684	Mitotic checkpoint protein BUB3 OS=Homo sapiens OX=9606 GN=BUB3 PE=1 SV=1	BUB3	0.96	0.487033
O43688	Phospholipid phosphatase 2 OS=Homo sapiens OX=9606 GN=PLPP2 PE=1 SV=1	PLPP2	0.947	0.61096
O43707	Alpha-actinin-4 OS=Homo sapiens OX=9606 GN=ACTN4 PE=1	ACTN4	1.089	0.482932
O43708	Maleylacetoacetate isomerase OS=Homo sapiens OX=9606 GN=GSTZ1 PE=1 SV=3	GSTZ1	0.987	0.839376
O43709	Probable 18S rRNA (guanine-N(7))-methyltransferase OS=Homo sapiens OX=9606	BUD23	0.856	0.396194
O43716	Glutamyl-tRNA(Gln) amidotransferase subunit C, mitochondrial OS=Homo sapiens OX=9606 GN=GATC PE=1 SV=1	GATC	0.916	0.176199
O43719	HIV Tat-specific factor 1 OS=Homo sapiens OX=9606 GN=HTATSF1 PE=1 SV=1	HTATSF1	0.909	0.033817
O43747	AP-1 complex subunit gamma-1 OS=Homo sapiens OX=9606 GN=AP1G1 PE=1 SV=5	AP1G1	1.03	0.842003
O43752	Syntaxin-6 OS=Homo sapiens OX=9606 GN=STX6 PE=1 SV=1	STX6	1.046	0.507983
O43760	Synaptogyrin-2 OS=Homo sapiens OX=9606 GN=SYNGR2 PE=1 SV=1	SYNGR2	1.098	0.332059
O43765	Small glutamine-rich tetratricopeptide repeat-containing protein alpha OS=Homo sapiens OX=9606 GN=SGTA PE=1 SV=1	SGTA	0.971	0.8573

O43768	Alpha-endosulfine OS=Homo sapiens OX=9606 GN=ENSA PE=1 SV=1	ENSA	0.994	0.932404
O43776	Asparagine--tRNA ligase, cytoplasmic OS=Homo sapiens OX=9606 GN=NARS1 PE=1	NARS1	1.006	0.95166
O43795	Unconventional myosin-Ib OS=Homo sapiens OX=9606 GN=MYO1B PE=1 SV=3	MYO1B	0.987	0.7769
O43808	Peroxisomal membrane protein PMP34 OS=Homo sapiens OX=9606 GN=SLC25A17 PE=1 SV=1	SLC25A17	0.859	0.064909
O43809	Cleavage and polyadenylation specificity factor subunit 5 OS=Homo sapiens OX=9606 GN=NUDT21 PE=1 SV=1	NUDT21	0.974	0.570627
O43815	Striatin OS=Homo sapiens OX=9606 GN=STRN PE=1 SV=4	STRN	1.124	0.17346
O43818	U3 small nucleolar RNA-interacting protein 2 OS=Homo sapiens OX=9606 GN=RRP9	RRP9	0.953	0.424313
O43819	Protein SCO2 homolog, mitochondrial OS=Homo sapiens OX=9606 GN=SCO2 PE=1 SV=3	SCO2	0.001	0.001
O43823	A-kinase anchor protein 8 OS=Homo sapiens OX=9606 GN=AKAP8 PE=1 SV=1	AKAP8	1.066	0.16076
O43837	Isocitrate dehydrogenase [NAD] subunit beta, mitochondrial OS=Homo sapiens OX=9606 GN=IDH3B PE=1 SV=2	IDH3B	1.068	0.373055
O43852	Calumenin OS=Homo sapiens OX=9606 GN=CALU PE=1 SV=2	CALU	1.055	0.247237
O43854	EGF-like repeat and discoidin I-like domain-containing protein 3 OS=Homo sapiens OX=9606 GN=EDIL3 PE=1 SV=1	EDIL3	1.014	0.88754
O43865	S-adenosylhomocysteine hydrolase-like protein 1 OS=Homo sapiens OX=9606 GN=AHCYL1 PE=1 SV=2	AHCYL1	0.99	0.975423
O43920	NADH dehydrogenase [ubiquinone] iron-sulfur protein 5 OS=Homo sapiens OX=9606 GN=NDUFS5 PE=1 SV=3	NDUFS5	1.026	0.814673
O60216	Double-strand-break repair protein rad21 homolog OS=Homo sapiens OX=9606 GN=RAD21 PE=1	RAD21	0.878	0.012804
O60220	Mitochondrial import inner membrane translocase subunit Tim8 A OS=Homo sapiens OX=9606 GN=TIMM8A PE=1	TIMM8A	0.977	0.780694

O60231	Pre-mRNA-splicing factor ATP-dependent RNA helicase DHX16 OS=Homo sapiens OX=9606 GN=DHX16 PE=1 SV=2	DHX16	1.092	0.810401
O60244	Mediator of RNA polymerase II transcription subunit 14 OS=Homo sapiens OX=9606 GN=MED14 PE=1 SV=2	MED14	0.839	0.202698
O60245	Protocadherin-7 OS=Homo sapiens OX=9606 GN=PCDH7	PCDH7	0.906	0.387513
O60264	SWI/SNF-related matrix-associated actin-dependent regulator of chromatin subfamily A member 5 OS=Homo sapiens OX=9606 GN=SMARCA5 PE=1	SMARCA5	0.943	0.555348
O60287	Nucleolar pre-ribosomal-associated protein 1 OS=Homo sapiens OX=9606 GN=URB1	URB1	0.954	0.570411
O60291	E3 ubiquitin-protein ligase MGRN1 OS=Homo sapiens OX=9606 GN=MGRN1 PE=1	MGRN1	1.11	0.316423
O60292	Signal-induced proliferation-associated 1-like protein 3 OS=Homo sapiens OX=9606 GN=SIPA1L3 PE=1 SV=3	SIPA1L3	0.87	
O60306	RNA helicase aquarius OS=Homo sapiens OX=9606 GN=AQR PE=1 SV=4	AQR	0.984	0.779968
O60313	Dynamin-like 120 kDa protein, mitochondrial OS=Homo sapiens OX=9606 GN=OPA1 PE=1 SV=3	OPA1	0.975	0.589902
O60341	Lysine-specific histone demethylase 1A OS=Homo sapiens OX=9606 GN=KDM1A	KDM1A	0.928	0.12474
O60353	Frizzled-6 OS=Homo sapiens OX=9606 GN=FZD6 PE=1 SV=2	FZD6	1.044	0.501422
O60437	Periplakin OS=Homo sapiens OX=9606 GN=PPL PE=1 SV=4	PPL	1.047	0.38932
O60487	Myelin protein zero-like protein 2 OS=Homo sapiens OX=9606 GN=MPZL2 PE=1 SV=1	MPZL2	0.865	0.089191
O60488	Long-chain-fatty-acid--CoA ligase 4 OS=Homo sapiens OX=9606 GN=ACSL4 PE=1 SV=2	ACSL4	0.997	0.978047
O60493	Sorting nexin-3 OS=Homo sapiens OX=9606 GN=SNX3 PE=1 SV=3	SNX3	0.824	0.018428
O60499	Syntaxin-10 OS=Homo sapiens OX=9606 GN=STX10 PE=1	STX10	1.058	0.135782
O60502	Protein O-GlcNAcase OS=Homo sapiens OX=9606 GN=OGA PE=1 SV=2	OGA	1.139	0.166322

O60506	Heterogeneous nuclear ribonucleoprotein Q OS=Homo sapiens OX=9606 GN=SYNCRIP PE=1 SV=2	SYNCRIP	0.946	0.170107
O60507	Protein-tyrosine sulfotransferase 1 OS=Homo sapiens OX=9606 GN=TPST1 PE=1 SV=1	TPST1	1.068	0.289344
O60508	Pre-mRNA-processing factor 17 OS=Homo sapiens OX=9606 GN=CDC40 PE=1 SV=1	CDC40	0.987	0.83338
O60563	Cyclin-T1 OS=Homo sapiens OX=9606 GN=CCNT1 PE=1	CCNT1	0.899	0.29506
O60568	Multifunctional procollagen lysine hydroxylase and glycosyltransferase LH3 OS=Homo sapiens OX=9606	PLOD3	1.028	0.656689
O60610	Protein diaphanous homolog 1 OS=Homo sapiens OX=9606 GN=DIAPH1 PE=1 SV=2	DIAPH1	1.256	0.034687
O60613	Selenoprotein F OS=Homo sapiens OX=9606 GN=SELENOF PE=1 SV=4	SELENOF	0.932	0.584498
O60664	Perilipin-3 OS=Homo sapiens OX=9606 GN=PLIN3 PE=1 SV=3	PLIN3	1.245	0.009054
O60684	Importin subunit alpha-7 OS=Homo sapiens OX=9606 GN=KPNA6 PE=1 SV=1	KPNA6	1.164	0.185366
O60701	UDP-glucose 6-dehydrogenase OS=Homo sapiens OX=9606 GN=UGDH PE=1 SV=1	UGDH	0.927	0.69167
O60716	Catenin delta-1 OS=Homo sapiens OX=9606 GN=CTNND1 PE=1 SV=1	CTNND1	0.926	0.048801
O60749	Sorting nexin-2 OS=Homo sapiens OX=9606 GN=SNX2 PE=1 SV=2	SNX2	1.075	0.42444
O60762	Dolichol-phosphate mannosyltransferase subunit 1 OS=Homo sapiens OX=9606 GN=DPM1 PE=1 SV=1	DPM1	1.034	0.838642
O60763	General vesicular transport factor p115 OS=Homo sapiens OX=9606 GN=USO1 PE=1 SV=2	USO1	0.943	0.05728
O60783	28S ribosomal protein S14, mitochondrial OS=Homo sapiens OX=9606 GN=MRPS14 PE=1 SV=1	MRPS14	0.824	0.091771
O60784	Target of Myb protein 1 OS=Homo sapiens OX=9606 GN=TOM1 PE=1 SV=2	TOM1	1.025	
O60828	Polyglutamine-binding protein 1 OS=Homo sapiens OX=9606 GN=PQBP1 PE=1 SV=1	PQBP1	0.849	0.048162

O60830	Mitochondrial import inner membrane translocase subunit Tim17-B OS=Homo sapiens OX=9606 GN=TIMM17B PE=1 SV=1	TIMM17B	0.515	
O60832	H/ACA ribonucleoprotein complex subunit DKC1 OS=Homo sapiens OX=9606 GN=DKC1 PE=1 SV=3	DKC1	1.012	0.995786
O60841	Eukaryotic translation initiation factor 5B OS=Homo sapiens OX=9606 GN=EIF5B PE=1 SV=4	EIF5B	1.047	0.432677
O60884	DnaJ homolog subfamily A member 2 OS=Homo sapiens OX=9606 GN=DNAJA2 PE=1	DNAJA2	1.07	0.481785
O60885	Bromodomain-containing protein 4 OS=Homo sapiens OX=9606 GN=BRD4 PE=1 SV=2	BRD4	0.817	0.027089
O60925	Prefoldin subunit 1 OS=Homo sapiens OX=9606 GN=PFDN1 PE=1 SV=2	PFDN1	0.999	0.917234
O60934	Nibrin OS=Homo sapiens OX=9606 GN=NBN PE=1 SV=1	NBN	1.01	0.875599
O60942	mRNA-capping enzyme OS=Homo sapiens OX=9606 GN=RNGTT PE=1 SV=1	RNGTT	0.801	0.047523
O75027	Iron-sulfur clusters transporter ABCB7, mitochondrial OS=Homo sapiens OX=9606 GN=ABCB7 PE=1 SV=2	ABCB7	1.073	0.475359
O75051	Plexin-A2 OS=Homo sapiens OX=9606 GN=PLXNA2 PE=1 SV=4	PLXNA2	1.273	0.156729
O75083	WD repeat-containing protein 1 OS=Homo sapiens OX=9606 GN=WDR1 PE=1 SV=4	WDR1	1.004	0.936425
O75084	Frizzled-7 OS=Homo sapiens OX=9606 GN=FZD7 PE=1 SV=2	FZD7	0.817	
O75110	Probable phospholipid-transporting ATPase IIA OS=Homo sapiens OX=9606 GN=ATP9A PE=1	ATP9A	0.999	0.957844
O75122	CLIP-associating protein 2 OS=Homo sapiens OX=9606 GN=CLASP2 PE=1 SV=3	CLASP2	1.12	0.113424
O75127	Pentatricopeptide repeat-containing protein 1, mitochondrial OS=Homo sapiens OX=9606 GN=PTCD1 PE=1 SV=2	PTCD1	0.896	0.088796
O75131	Copine-3 OS=Homo sapiens OX=9606 GN=CPNE3 PE=1	CPNE3	1.046	0.522757
O75146	Huntingtin-interacting protein 1-related protein OS=Homo sapiens OX=9606 GN=HIP1R PE=1 SV=2	HIP1R	1.191	0.336142

O75150	E3 ubiquitin-protein ligase BRE1B OS=Homo sapiens OX=9606 GN=RNF40 PE=1 SV=5	RNF40	0.962	0.47426
O75151	Lysine-specific demethylase PHF2 OS=Homo sapiens OX=9606 GN=PHF2 PE=1 SV=4	PHF2	0.976	0.773541
O75152	Zinc finger CCCH domain-containing protein 11A OS=Homo sapiens OX=9606 GN=ZC3H11A PE=1 SV=3	ZC3H11A	0.895	0.088687
O75153	Clustered mitochondria protein homolog OS=Homo sapiens OX=9606 GN=CLUH PE=1 SV=2	CLUH	0.996	0.952731
O75165	DnaJ homolog subfamily C member 13 OS=Homo sapiens OX=9606 GN=DNAJC13 PE=1	DNAJC13	1.421	0.013976
O75175	CCR4-NOT transcription complex subunit 3 OS=Homo sapiens OX=9606 GN=CNOT3 PE=1	CNOT3	0.889	0.273163
O75190	DnaJ homolog subfamily B member 6 OS=Homo sapiens OX=9606 GN=DNAJB6 PE=1	DNAJB6	0.986	0.789514
O75204	Transmembrane protein 127 OS=Homo sapiens OX=9606 GN=TMEM127 PE=1 SV=1	TMEM127	1.124	0.35231
O75208	Ubiquinone biosynthesis protein COQ9, mitochondrial OS=Homo sapiens OX=9606 GN=COQ9 PE=1 SV=1	COQ9	0.952	0.402586
O75223	Gamma-glutamylcyclotransferase OS=Homo sapiens OX=9606 GN=GGCT PE=1 SV=1	GGCT	0.863	0.56815
O75251	NADH dehydrogenase [ubiquinone] iron-sulfur protein 7, mitochondrial OS=Homo sapiens OX=9606 GN=NDUFS7 PE=1	NDUFS7	1.082	0.579992
O75306	NADH dehydrogenase [ubiquinone] iron-sulfur protein 2, mitochondrial OS=Homo sapiens OX=9606 GN=NDUFS2 PE=1	NDUFS2	1.01	0.851727
O75312	Zinc finger protein ZPR1 OS=Homo sapiens OX=9606 GN=ZPR1 PE=1 SV=1	ZPR1	0.962	0.659874
O75323	Protein NipSnap homolog 2 OS=Homo sapiens OX=9606 GN=NIPSNAP2 PE=1 SV=1	NIPSNAP2	1.037	0.692886
O75326	Semaphorin-7A OS=Homo sapiens OX=9606 GN=SEMA7A PE=1 SV=1	SEMA7A	0.925	0.440395
O75340	Programmed cell death protein 6 OS=Homo sapiens OX=9606 GN=PDCD6 PE=1 SV=1	PDCD6	0.975	0.81926

O75347	Tubulin-specific chaperone A OS=Homo sapiens OX=9606 GN=TBCA PE=1 SV=3	TBCA	0.887	0.438117
O75348	V-type proton ATPase subunit G 1 OS=Homo sapiens OX=9606 GN=ATP6V1G1 PE=1 SV=3	ATP6V1G1	1.027	0.867229
O75351	Vacuolar protein sorting-associated protein 4B OS=Homo sapiens OX=9606 GN=VPS4B PE=1	VPS4B	1.009	0.915234
O75352	Mannose-P-dolichol utilization defect 1 protein OS=Homo sapiens OX=9606 GN=MPDU1 PE=1	MPDU1	0.994	0.931971
O75367	Core histone macro-H2A.1 OS=Homo sapiens OX=9606 GN=MACROH2A1 PE=1 SV=4	MACROH2A1	1.16	0.334305
O75368	SH3 domain-binding glutamic acid-rich-like protein OS=Homo sapiens OX=9606 GN=SH3BGRL PE=1 SV=1	SH3BGRL	1.108	0.348447
O75369	Filamin-B OS=Homo sapiens OX=9606 GN=FLNB PE=1 SV=2	FLNB	0.911	0.231798
O75380	NADH dehydrogenase [ubiquinone] iron-sulfur protein 6, mitochondrial OS=Homo sapiens OX=9606 GN=NDUFS6 PE=1	NDUFS6	1.038	0.595791
O75381	Peroxisomal membrane protein PEX14 OS=Homo sapiens OX=9606 GN=PEX14 PE=1	PEX14	1.073	0.399685
O75390	Citrate synthase, mitochondrial OS=Homo sapiens OX=9606 GN=CS PE=1 SV=2	CS	1.095	0.16778
O75396	Vesicle-trafficking protein SEC22b OS=Homo sapiens OX=9606 GN=SEC22B PE=1	SEC22B	1.088	0.177455
O75400	Pre-mRNA-processing factor 40 homolog A OS=Homo sapiens OX=9606 GN=PRPF40A PE=1 SV=2	PRPF40A	0.911	0.072149
O75414	Nucleoside diphosphate kinase 6 OS=Homo sapiens OX=9606 GN=NME6 PE=1 SV=3	NME6	0.844	0.013492
O75427	Leucine-rich repeat and calponin homology domain-containing protein 4 OS=Homo sapiens OX=9606 GN=LRCH4 PE=1	LRCH4	1.151	0.363139
O75431	Metaxin-2 OS=Homo sapiens OX=9606 GN=MTX2 PE=1 SV=1	MTX2	1.063	0.279173
O75436	Vacuolar protein sorting-associated protein 26A OS=Homo sapiens OX=9606 GN=VPS26A PE=1	VPS26A	0.952	0.557731

O75438	NADH dehydrogenase [ubiquinone] 1 beta subcomplex subunit 1 OS=Homo sapiens OX=9606 GN=NDUFB1 PE=1	NDUFB1	0.925	0.141535
O75439	Mitochondrial-processing peptidase subunit beta OS=Homo sapiens OX=9606 GN=PMPCB	PMPCB	1.017	0.783389
O75448	Mediator of RNA polymerase II transcription subunit 24 OS=Homo sapiens OX=9606 GN=MED24 PE=1 SV=1	MED24	1.027	0.908001
O75475	PC4 and SFRS1-interacting protein OS=Homo sapiens OX=9606 GN=PSIP1 PE=1 SV=1	PSIP1	0.883	0.012168
O75477	Erlin-1 OS=Homo sapiens OX=9606 GN=ERLIN1 PE=1	ERLIN1	1.072	0.222351
O75486	Transcription initiation protein SPT3 homolog OS=Homo sapiens OX=9606 GN=SUPT3H PE=1 SV=3	SUPT3H	1.315	0.394728
O75487	Glypican-4 OS=Homo sapiens OX=9606 GN=GPC4 PE=1 SV=4	GPC4	0.951	0.716409
O75489	NADH dehydrogenase [ubiquinone] iron-sulfur protein 3, mitochondrial OS=Homo sapiens OX=9606 GN=NDUFS3 PE=1	NDUFS3	1.082	0.369332
O75494	Serine/arginine-rich splicing factor 10 OS=Homo sapiens OX=9606 GN=SRSF10 PE=1 SV=1	SRSF10	0.885	0.097112
O75506	Heat shock factor-binding protein 1 OS=Homo sapiens OX=9606 GN=HSBP1 PE=1 SV=1	HSBP1	0.99	0.939121
O75509	Tumor necrosis factor receptor superfamily member 21 OS=Homo sapiens OX=9606 GN=TNFRSF21 PE=1 SV=1	TNFRSF21	1.106	0.210512
O75530	Polycomb protein EED OS=Homo sapiens OX=9606 GN=EED PE=1 SV=2	EED	0.97	0.300548
O75531	Barrier-to-autointegration factor OS=Homo sapiens OX=9606 GN=BANF1 PE=1 SV=1	BANF1	1.209	0.144551
O75533	Splicing factor 3B subunit 1 OS=Homo sapiens OX=9606 GN=SF3B1 PE=1 SV=3	SF3B1	0.966	0.51773
O75534	Cold shock domain-containing protein E1 OS=Homo sapiens OX=9606 GN=CSDE1 PE=1	CSDE1	1.035	0.678063
O75554	WW domain-binding protein 4 OS=Homo sapiens OX=9606 GN=WBP4 PE=1 SV=1	WBP4	0.657	

O75569	Interferon-inducible double-stranded RNA-dependent protein kinase activator A OS=Homo sapiens OX=9606 GN=PRKRA	PRKRA	1.196	0.042418
O75607	Nucleoplasmin-3 OS=Homo sapiens OX=9606 GN=NPM3	NPM3	0.905	0.196053
O75608	Acyl-protein thioesterase 1 OS=Homo sapiens OX=9606 GN=LYPLA1 PE=1 SV=1	LYPLA1	1.085	0.176724
O75616	GTPase Era, mitochondrial OS=Homo sapiens OX=9606 GN=ERAL1 PE=1 SV=2	ERAL1	0.913	0.296944
O75629	Protein CREG1 OS=Homo sapiens OX=9606 GN=CREG1 PE=1	CREG1	1.041	0.755195
O75643	U5 small nuclear ribonucleoprotein 200 kDa helicase OS=Homo sapiens OX=9606 GN=SNRNP200 PE=1 SV=2	SNRNP200	0.949	0.298558
O75683	Surfeit locus protein 6 OS=Homo sapiens OX=9606 GN=SURF6 PE=1 SV=3	SURF6	0.914	0.362925
O75691	Small subunit processome component 20 homolog OS=Homo sapiens OX=9606 GN=UTP20 PE=1 SV=3	UTP20	0.96	0.600918
O75694	Nuclear pore complex protein Nup155 OS=Homo sapiens OX=9606 GN=NUP155 PE=1	NUP155	1.006	0.886688
O75695	Protein XRP2 OS=Homo sapiens OX=9606 GN=RP2 PE=1 SV=4	RP2	1.072	0.514191
O75717	WD repeat and HMG-box DNA-binding protein 1 OS=Homo sapiens OX=9606 GN=WDHD1 PE=1 SV=1	WDHD1	0.824	0.144451
O75718	Cartilage-associated protein OS=Homo sapiens OX=9606 GN=CRTAP PE=1 SV=1	CRTAP	1.003	0.98215
O75746	Calcium-binding mitochondrial carrier protein Aralar1 OS=Homo sapiens OX=9606 GN=SLC25A12 PE=1 SV=2	SLC25A12	1.018	0.831338
O75787	Renin receptor OS=Homo sapiens OX=9606 GN=ATP6AP2 PE=1 SV=2	ATP6AP2	0.923	0.239442
O75817	Ribonuclease P protein subunit p20 OS=Homo sapiens OX=9606 GN=POP7 PE=1 SV=2	POP7	0.916	0.120709
O75818	Ribonuclease P protein subunit p40 OS=Homo sapiens OX=9606 GN=RPP40 PE=1 SV=3	RPP40	0.958	0.768236

O75821	Eukaryotic translation initiation factor 3 subunit G OS=Homo sapiens OX=9606 GN=EIF3G PE=1 SV=2	EIF3G	1.026	0.807562
O75822	Eukaryotic translation initiation factor 3 subunit J OS=Homo sapiens OX=9606 GN=EIF3J	EIF3J	0.982	0.70796
O75832	26S proteasome non-ATPase regulatory subunit 10 OS=Homo sapiens OX=9606 GN=PSMD10 PE=1 SV=1	PSMD10	1.315	0.113933
O75844	CAAX prenyl protease 1 homolog OS=Homo sapiens OX=9606 GN=ZMPSTE24 PE=1 SV=2	ZMPSTE24	0.979	0.600593
O75874	Isocitrate dehydrogenase [NADP] cytoplasmic OS=Homo sapiens OX=9606 GN=IDH1 PE=1 SV=2	IDH1	1.077	0.656703
O75879	Glutamyl-tRNA(Gln) amidotransferase subunit B, mitochondrial OS=Homo sapiens OX=9606 GN=GATB PE=1 SV=1	GATB	1.246	0.040111
O75880	Protein SCO1 homolog, mitochondrial OS=Homo sapiens OX=9606 GN=SCO1 PE=1 SV=1	SCO1	0.972	0.670959
O75882	Attractin OS=Homo sapiens OX=9606 GN=ATRN PE=1 SV=2	ATRN	0.996	
O75907	Diacylglycerol O-acyltransferase 1 OS=Homo sapiens OX=9606 GN=DGAT1 PE=1 SV=2	DGAT1	0.94	0.473083
O75909	Cyclin-K OS=Homo sapiens OX=9606 GN=CCNK PE=1 SV=2	CCNK	0.847	0.05011
O75911	Short-chain dehydrogenase/reductase 3 OS=Homo sapiens OX=9606 GN=DHRS3 PE=1 SV=2	DHRS3	0.887	0.06093
O75915	PRA1 family protein 3 OS=Homo sapiens OX=9606 GN=ARL6IP5 PE=1 SV=1	ARL6IP5	0.985	0.802059
O75934	Pre-mRNA-splicing factor SPF27 OS=Homo sapiens OX=9606 GN=BCAS2 PE=1 SV=1	BCAS2	1.008	0.832247
O75937	DnaJ homolog subfamily C member 8 OS=Homo sapiens OX=9606 GN=DNAJC8 PE=1	DNAJC8	0.895	0.57832
O75940	Survival of motor neuron-related-splicing factor 30 OS=Homo sapiens OX=9606 GN=SMNDC1 PE=1 SV=1	SMNDC1	0.862	0.171804
O75947	ATP synthase subunit d, mitochondrial OS=Homo sapiens OX=9606 GN=ATP5PD PE=1 SV=3	ATP5PD	0.948	0.572571

O75955	Flotillin-1 OS=Homo sapiens OX=9606 GN=FLOT1 PE=1	FLOT1	0.949	0.208746
O75964	ATP synthase subunit g, mitochondrial OS=Homo sapiens OX=9606 GN=ATP5MG PE=1 SV=3	ATP5MG	0.86	0.095694
O75976	Carboxypeptidase D OS=Homo sapiens OX=9606 GN=CPD PE=1 SV=2	CPD	1.029	0.503138
O76003	Glutaredoxin-3 OS=Homo sapiens OX=9606 GN=GLRX3 PE=1	GLRX3	1.092	0.428545
O76021	Ribosomal L1 domain-containing protein 1 OS=Homo sapiens OX=9606 GN=RSL1D1 PE=1	RSL1D1	1.127	0.735968
O76024	Wolframin OS=Homo sapiens OX=9606 GN=WFS1 PE=1 SV=2	WFS1	1.204	0.080738
O76031	ATP-dependent Clp protease ATP-binding subunit clpX-like, mitochondrial OS=Homo sapiens OX=9606 GN=CLPX PE=1 SV=2	CLPX	0.918	0.172583
O76070	Gamma-synuclein OS=Homo sapiens OX=9606 GN=SNCG PE=1 SV=2	SNCG	1.175	0.270043
O76094	Signal recognition particle subunit SRP72 OS=Homo sapiens OX=9606 GN=SRP72 PE=1 SV=3	SRP72	1.069	0.182348
O94766	Galactosylgalactosylxylosylprotein 3-beta-glucuronosyltransferase 3 OS=Homo sapiens OX=9606 GN=B3GAT3 PE=1 SV=2	B3GAT3	0.941	0.575033
O94776	Metastasis-associated protein MTA2 OS=Homo sapiens OX=9606 GN=MTA2 PE=1 SV=1	MTA2	0.921	0.387806
O94826	Mitochondrial import receptor subunit TOM70 OS=Homo sapiens OX=9606 GN=TOMM70 PE=1 SV=1	TOMM70	0.986	0.783761
O94832	Unconventional myosin-Id OS=Homo sapiens OX=9606 GN=MYO1D PE=1 SV=2	MYO1D	1.034	0.661755
O94842	TOX high mobility group box family member 4 OS=Homo sapiens OX=9606 GN=TOX4	TOX4	0.941	0.579233
O94851	[F-actin]-monooxygenase MICAL2 OS=Homo sapiens OX=9606 GN=MICAL2 PE=1	MICAL2	0.993	0.9639
O94874	E3 UFM1-protein ligase 1 OS=Homo sapiens OX=9606 GN=UFL1 PE=1 SV=2	UFL1	0.922	0.159052
O94880	PHD finger protein 14 OS=Homo sapiens OX=9606 GN=PHF14 PE=1 SV=2	PHF14	0.88	0.111521

O94886	CSC1-like protein 1 OS=Homo sapiens OX=9606 GN=TMEM63A PE=1 SV=3	TMEM63A	1000	0.001
O94888	UBX domain-containing protein 7 OS=Homo sapiens OX=9606 GN=UBXN7 PE=1 SV=2	UBXN7	0.939	0.512302
O94901	SUN domain-containing protein 1 OS=Homo sapiens OX=9606 GN=SUN1 PE=1 SV=4	SUN1	1.024	0.755966
O94903	Pyridoxal phosphate homeostasis protein OS=Homo sapiens OX=9606 GN=PLPBP PE=1	PLPBP	1.102	
O94905	Erlin-2 OS=Homo sapiens OX=9606 GN=ERLIN2 PE=1	ERLIN2	1.007	0.906901
O94906	Pre-mRNA-processing factor 6 OS=Homo sapiens OX=9606 GN=PRPF6 PE=1 SV=1	PRPF6	0.932	0.50506
O94915	Protein furry homolog-like OS=Homo sapiens OX=9606 GN=FRYL PE=1 SV=2	FRYL	1.087	0.388926
O94919	Endonuclease domain-containing 1 protein OS=Homo sapiens OX=9606 GN=ENDOD1 PE=1	ENDOD1	1.043	0.675102
O94925	Glutaminase kidney isoform, mitochondrial OS=Homo sapiens OX=9606 GN=GLS PE=1 SV=1	GLS	1.012	0.66777
O94929	Actin-binding LIM protein 3 OS=Homo sapiens OX=9606 GN=ABLIM3 PE=1 SV=3	ABLIM3	0.999	0.964175
O94955	Rho-related BTB domain-containing protein 3 OS=Homo sapiens OX=9606 GN=RHOBTB3 PE=1 SV=2	RHOBTB3	0.821	0.004628
O94973	AP-2 complex subunit alpha-2 OS=Homo sapiens OX=9606 GN=AP2A2 PE=1 SV=2	AP2A2	1.042	0.330742
O94979	Protein transport protein Sec31A OS=Homo sapiens OX=9606 GN=SEC31A PE=1 SV=3	SEC31A	1.095	0.517975
O94992	Protein HEXIM1 OS=Homo sapiens OX=9606 GN=HEXIM1 PE=1 SV=1	HEXIM1	0.782	0.145495
O95070	Protein YIF1A OS=Homo sapiens OX=9606 GN=YIF1A PE=1 SV=2	YIF1A	0.894	0.217532
O95071	E3 ubiquitin-protein ligase UBR5 OS=Homo sapiens OX=9606 GN=UBR5 PE=1 SV=2	UBR5	0.957	0.276983
O95104	SR-related and CTD-associated factor 4 OS=Homo sapiens OX=9606 GN=SCAF4 PE=1	SCAF4	0.947	0.221448

O95139	NADH dehydrogenase [ubiquinone] 1 beta subcomplex subunit 6 OS=Homo sapiens OX=9606 GN=NDUFB6 PE=1	NDUFB6	1.093	0.204412
O95140	Mitofusin-2 OS=Homo sapiens OX=9606 GN=MFN2 PE=1 SV=3	MFN2	1.047	0.604273
O95155	Ubiquitin conjugation factor E4 B OS=Homo sapiens OX=9606 GN=UBE4B PE=1 SV=1	UBE4B	0.467	
O95159	Zinc finger protein-like 1 OS=Homo sapiens OX=9606 GN=ZFPL1 PE=1 SV=2	ZFPL1	1.056	0.314374
O95167	NADH dehydrogenase [ubiquinone] 1 alpha subcomplex subunit 3 OS=Homo sapiens OX=9606 GN=NDUFA3 PE=1	NDUFA3	0.946	0.667562
O95168	NADH dehydrogenase [ubiquinone] 1 beta subcomplex subunit 4 OS=Homo sapiens OX=9606 GN=NDUFB4 PE=1	NDUFB4	0.904	0.421776
O95169	NADH dehydrogenase [ubiquinone] 1 beta subcomplex subunit 8, mitochondrial OS=Homo sapiens OX=9606 GN=NDUFB8 PE=1 SV=1	NDUFB8	1	0.969801
O95171	Sciellin OS=Homo sapiens OX=9606 GN=SCEL PE=1 SV=2	SCEL	0.94	0.583492
O95182	NADH dehydrogenase [ubiquinone] 1 alpha subcomplex subunit 7 OS=Homo sapiens OX=9606 GN=NDUFA7 PE=1	NDUFA7	0.885	0.415898
O95197	Reticulon-3 OS=Homo sapiens OX=9606 GN=RTN3 PE=1 SV=2	RTN3	0.897	0.456104
O95202	Mitochondrial proton/calcium exchanger protein OS=Homo sapiens OX=9606 GN=LETM1 PE=1 SV=1	LETM1	1.081	0.343349
O95218	Zinc finger Ran-binding domain-containing protein 2 OS=Homo sapiens OX=9606 GN=ZRANB2 PE=1 SV=2	ZRANB2	0.916	0.137572
O95219	Sorting nexin-4 OS=Homo sapiens OX=9606 GN=SNX4 PE=1 SV=1	SNX4	1.164	
O95232	Luc7-like protein 3 OS=Homo sapiens OX=9606 GN=LUC7L3 PE=1 SV=2	LUC7L3	1.023	0.560868
O95235	Kinesin-like protein KIF20A OS=Homo sapiens OX=9606 GN=KIF20A PE=1 SV=1	KIF20A	1.009	0.86352
O95239	Chromosome-associated kinesin KIF4A OS=Homo sapiens OX=9606 GN=KIF4A PE=1 SV=3	KIF4A	0.934	0.303088

O95249	Golgi SNAP receptor complex member 1 OS=Homo sapiens OX=9606 GN=GOSR1 PE=1	GOSR1	0.939	0.716342
O95251	Histone acetyltransferase KAT7 OS=Homo sapiens OX=9606 GN=KAT7 PE=1 SV=1	KAT7	0.946	0.622577
O95273	Cyclin-D1-binding protein 1 OS=Homo sapiens OX=9606 GN=CCNDBP1 PE=1 SV=2	CCNDBP1	1000	0.001
O95274	Ly6/PLAUR domain-containing protein 3 OS=Homo sapiens OX=9606 GN=LYPD3 PE=1	LYPD3	0.96	0.918171
O95292	Vesicle-associated membrane protein-associated protein B/C OS=Homo sapiens OX=9606 GN=VAPB PE=1 SV=3	VAPB	1.156	0.118799
O95297	Myelin protein zero-like protein 1 OS=Homo sapiens OX=9606 GN=MPZL1 PE=1 SV=1	MPZL1	0.947	0.505733
O95298	NADH dehydrogenase [ubiquinone] 1 subunit C2 OS=Homo sapiens OX=9606 GN=NDUFC2 PE=1 SV=1	NDUFC2	1.194	0.20847
O95299	NADH dehydrogenase [ubiquinone] 1 alpha subcomplex subunit 10, mitochondrial OS=Homo sapiens OX=9606 GN=NDUFA10 PE=1 SV=1	NDUFA10	0.969	0.665902
O95302	Peptidyl-prolyl cis-trans isomerase FKBP9 OS=Homo sapiens OX=9606 GN=FKBP9 PE=1	FKBP9	0.952	0.407959
O95336	6-phosphogluconolactonase OS=Homo sapiens OX=9606 GN=PGLS PE=1 SV=2	PGLS	1.102	0.49951
O95347	Structural maintenance of chromosomes protein 2 OS=Homo sapiens OX=9606 GN=SMC2 PE=1 SV=2	SMC2	0.975	0.815103
O95361	Tripartite motif-containing protein 16 OS=Homo sapiens OX=9606 GN=TRIM16 PE=1 SV=3	TRIM16	1.165	0.120482
O95365	Zinc finger and BTB domain-containing protein 7A OS=Homo sapiens OX=9606 GN=ZBTB7A PE=1 SV=1	ZBTB7A	0.886	0.103782
O95372	Acyl-protein thioesterase 2 OS=Homo sapiens OX=9606 GN=LYPLA2 PE=1 SV=1	LYPLA2	1.58	0.007719
O95373	Importin-7 OS=Homo sapiens OX=9606 GN=IPO7 PE=1 SV=1	IPO7	1.197	0.109581
O95394	Phosphoacetylglucosamine mutase OS=Homo sapiens OX=9606 GN=PGM3 PE=1 SV=1	PGM3	1.145	0.385546

O95400	CD2 antigen cytoplasmic tail-binding protein 2 OS=Homo sapiens OX=9606 GN=CD2BP2 PE=1 SV=1	CD2BP2	0.93	0.036912
O95425	Supervillin OS=Homo sapiens OX=9606 GN=SVIL PE=1 SV=2	SVIL	0.85	0.092584
O95433	Activator of 90 kDa heat shock protein ATPase homolog 1 OS=Homo sapiens OX=9606 GN=AHSA1 PE=1 SV=1	AHSA1	1.106	0.377246
O95453	Poly(A)-specific ribonuclease PARN OS=Homo sapiens OX=9606 GN=PARN PE=1 SV=1	PARN	0.975	0.786084
O95456	Proteasome assembly chaperone 1 OS=Homo sapiens OX=9606 GN=PSMG1 PE=1 SV=1	PSMG1	1.037	0.774223
O95470	Sphingosine-1-phosphate lyase 1 OS=Homo sapiens OX=9606 GN=SGPL1 PE=1 SV=3	SGPL1	1.026	0.60149
O95478	Ribosome biogenesis protein NSA2 homolog OS=Homo sapiens OX=9606 GN=NSA2 PE=1 SV=1	NSA2	1.171	0.097056
O95486	Protein transport protein Sec24A OS=Homo sapiens OX=9606 GN=SEC24A PE=1 SV=2	SEC24A	1.139	0.618433
O95563	Mitochondrial pyruvate carrier 2 OS=Homo sapiens OX=9606 GN=MPC2 PE=1 SV=1	MPC2	0.999	0.944972
O95571	Persulfide dioxygenase ETHE1, mitochondrial OS=Homo sapiens OX=9606 GN=ETHE1 PE=1	ETHE1	1.025	0.691647
O95573	Fatty acid CoA ligase Acsl3 OS=Homo sapiens OX=9606 GN=ACSL3 PE=1 SV=3	ACSL3	0.936	0.371929
O95602	DNA-directed RNA polymerase I subunit RPA1 OS=Homo sapiens OX=9606 GN=POLR1A PE=1 SV=2	POLR1A	0.933	0.20508
O95619	YEATS domain-containing protein 4 OS=Homo sapiens OX=9606 GN=YEATS4 PE=1 SV=1	YEATS4	0.937	
O95639	Cleavage and polyadenylation specificity factor subunit 4 OS=Homo sapiens OX=9606 GN=CPSF4 PE=1 SV=1	CPSF4	1.055	0.418773
O95674	Phosphatidate cytidylyltransferase 2 OS=Homo sapiens OX=9606 GN=CDS2 PE=1 SV=1	CDS2	1.198	0.053457
O95721	Synaptosomal-associated protein 29 OS=Homo sapiens OX=9606 GN=SNAP29 PE=1 SV=1	SNAP29	0.938	0.525506

O95747	Serine/threonine-protein kinase OSR1 OS=Homo sapiens OX=9606 GN=OXSR1 PE=1	OXSR1	1.052	0.576106
O95757	Heat shock 70 kDa protein 4L OS=Homo sapiens OX=9606 GN=HSPA4L PE=1 SV=3	HSPA4L	1.017	0.790118
O95758	Polypyrimidine tract-binding protein 3 OS=Homo sapiens OX=9606 GN=PTBP3 PE=1	PTBP3	1.088	0.205001
O95772	STARD3 N-terminal-like protein OS=Homo sapiens OX=9606 GN=STARD3NL PE=1 SV=1	STARD3NL	1.059	0.680862
O95777	U6 snRNA-associated Sm-like protein LSm8 OS=Homo sapiens OX=9606 GN=LSM8 PE=1 SV=3	LSM8	0.895	0.005567
O95782	AP-2 complex subunit alpha-1 OS=Homo sapiens OX=9606 GN=AP2A1 PE=1 SV=3	AP2A1	1.016	0.514719
O95785	Protein Wiz OS=Homo sapiens OX=9606 GN=WIZ PE=1 SV=2	WIZ	0.999	0.944181
O95793	Double-stranded RNA-binding protein Staufen homolog 1 OS=Homo sapiens OX=9606 GN=STAU1 PE=1 SV=2	STAU1	0.971	0.608887
O95816	BAG family molecular chaperone regulator 2 OS=Homo sapiens OX=9606 GN=BAG2 PE=1 SV=1	BAG2	1.118	0.039786
O95817	BAG family molecular chaperone regulator 3 OS=Homo sapiens OX=9606 GN=BAG3 PE=1 SV=3	BAG3	1.012	0.871189
O95819	Mitogen-activated protein kinase kinase kinase kinase 4 OS=Homo sapiens OX=9606 GN=MAP4K4 PE=1 SV=2	MAP4K4	1.291	0.282473
O95831	Apoptosis-inducing factor 1, mitochondrial OS=Homo sapiens OX=9606 GN=AIFM1 PE=1	AIFM1	0.994	0.853552
O95837	Guanine nucleotide-binding protein subunit alpha-14 OS=Homo sapiens OX=9606	GNA14	0.806	
O95861	3'(2'),5'-bisphosphate nucleotidase 1 OS=Homo sapiens OX=9606 GN=BPNT1 PE=1 SV=1	BPNT1	1.045	0.782067
O95870	Phosphatidylserine lipase ABHD16A OS=Homo sapiens OX=9606 GN=ABHD16A PE=1 SV=3	ABHD16A	1.24	
O95881	Thioredoxin domain-containing protein 12 OS=Homo sapiens OX=9606 GN=TXNDC12 PE=1 SV=1	TXNDC12	1.006	0.867108

O95926	Pre-mRNA-splicing factor SYF2 OS=Homo sapiens OX=9606 GN=SYF2 PE=1 SV=1	SYF2	1.038	
O95983	Methyl-CpG-binding domain protein 3 OS=Homo sapiens OX=9606 GN=MBD3 PE=1 SV=1	MBD3	0.868	0.325153
O95994	Anterior gradient protein 2 homolog OS=Homo sapiens OX=9606 GN=AGR2 PE=1 SV=1	AGR2	1.14	0.078831
O96000	NADH dehydrogenase [ubiquinone] 1 beta subcomplex subunit 10 OS=Homo sapiens OX=9606 GN=NDUFB10 PE=1	NDUFB10	1.021	0.621219
O96005	Cleft lip and palate transmembrane protein 1 OS=Homo sapiens OX=9606 GN=CLPTM1 PE=1 SV=1	CLPTM1	1.003	0.916167
O96008	Mitochondrial import receptor subunit TOM40 homolog OS=Homo sapiens OX=9606 GN=TOMM40 PE=1 SV=1	TOMM40	1.015	0.73944
O96019	Actin-like protein 6A OS=Homo sapiens OX=9606 GN=ACTL6A PE=1 SV=1	ACTL6A	0.918	0.019544
O96028	Histone-lysine N-methyltransferase NSD2 OS=Homo sapiens OX=9606 GN=NSD2 PE=1 SV=1	NSD2	0.892	
P00167	Cytochrome b5 OS=Homo sapiens OX=9606 GN=CYB5A PE=1	CYB5A	0.952	0.473229
P00338	L-lactate dehydrogenase A chain OS=Homo sapiens OX=9606 GN=LDHA PE=1 SV=2	LDHA	1.031	0.883108
P00367	Glutamate dehydrogenase 1, mitochondrial OS=Homo sapiens OX=9606 GN=GLUD1 PE=1	GLUD1	1.062	0.096887
P00387	NADH-cytochrome b5 reductase 3 OS=Homo sapiens OX=9606 GN=CYB5R3 PE=1 SV=3	CYB5R3	1.006	0.918587
P00390	Glutathione reductase, mitochondrial OS=Homo sapiens OX=9606 GN=GSR PE=1 SV=2	GSR	0.919	0.401291
P00395	Cytochrome c oxidase subunit 1 OS=Homo sapiens OX=9606 GN=MT-CO1 PE=1 SV=1	MT-CO1	0.944	0.652276
P00403	Cytochrome c oxidase subunit 2 OS=Homo sapiens OX=9606 GN=MT-CO2 PE=1 SV=1	MT-CO2	1.052	0.605442
P00414	Cytochrome c oxidase subunit 3 OS=Homo sapiens OX=9606 GN=MT-CO3 PE=1 SV=2	MT-CO3	0.821	0.147368
P00441	Superoxide dismutase [Cu-Zn] OS=Homo sapiens OX=9606 GN=SOD1 PE=1 SV=2	SOD1	1.09	0.331036

P00491	Purine nucleoside phosphorylase OS=Homo sapiens OX=9606 GN=PNP PE=1 SV=2	PNP	1.029	0.909713
P00492	Hypoxanthine-guanine phosphoribosyltransferase OS=Homo sapiens OX=9606 GN=HPRT1 PE=1 SV=2	HPRT1	1.057	0.715276
P00505	Aspartate aminotransferase, mitochondrial OS=Homo sapiens OX=9606 GN=GOT2 PE=1 SV=3	GOT2	1.066	0.106424
P00533	Epidermal growth factor receptor OS=Homo sapiens OX=9606 GN=EGFR PE=1 SV=2	EGFR	0.985	0.803847
P00558	Phosphoglycerate kinase 1 OS=Homo sapiens OX=9606 GN=PGK1 PE=1 SV=3	PGK1	0.942	0.569378
P00568	Adenylate kinase isoenzyme 1 OS=Homo sapiens OX=9606 GN=AK1 PE=1 SV=3	AK1	0.976	0.576415
P00749	Urokinase-type plasminogen activator OS=Homo sapiens OX=9606 GN=PLAU PE=1 SV=2	PLAU	0.957	0.671172
P01033	Metalloproteinase inhibitor 1 OS=Homo sapiens OX=9606 GN=TIMP1 PE=1 SV=1	TIMP1	1.019	0.902133
P01034	Cystatin-C OS=Homo sapiens OX=9606 GN=CST3 PE=1 SV=1	CST3	0.915	0.616464
P01111	GTPase NRas OS=Homo sapiens OX=9606 GN=NRAS PE=1 SV=1	NRAS	1.652	
P01112	GTPase HRas OS=Homo sapiens OX=9606 GN=HRAS PE=1 SV=1	HRAS	0.962	0.664971
P01116	GTPase KRas OS=Homo sapiens OX=9606 GN=KRAS PE=1 SV=1	KRAS	0.946	0.834339
P01130	Low-density lipoprotein receptor OS=Homo sapiens OX=9606 GN=LDLR PE=1 SV=1	LDLR	1.189	0.113157
P01137	Transforming growth factor beta-1 proprotein OS=Homo sapiens OX=9606 GN=TGFB1 PE=1	TGFB1	0.916	0.634587
P01889	HLA class I histocompatibility antigen, B alpha chain OS=Homo sapiens OX=9606 GN=HLA-B PE=1 SV=3	HLA-B	1.132	0.510516
P01893	Putative HLA class I histocompatibility antigen, alpha chain H OS=Homo sapiens OX=9606 GN=HLA-H PE=5	HLA-H	0.893	0.142366
P02545	Prelamin-A/C OS=Homo sapiens OX=9606 GN=LMNA PE=1	LMNA	1.035	0.54734
P02656	Apolipoprotein C-III OS=Homo sapiens OX=9606 GN=APOC3 PE=1 SV=1	APOC3	1.265	0.471059

P02751	Fibronectin OS=Homo sapiens OX=9606 GN=FN1 PE=1 SV=5	FN1	1.835	0.016963
P02786	Transferrin receptor protein 1 OS=Homo sapiens OX=9606 GN=TFRC PE=1 SV=2	TFRC	1.117	0.0651
P02792	Ferritin light chain OS=Homo sapiens OX=9606 GN=FTL PE=1 SV=2	FTL	1.057	0.570163
P02794	Ferritin heavy chain OS=Homo sapiens OX=9606 GN=FTH1 PE=1 SV=2	FTH1	1.077	0.496224
P03905	NADH-ubiquinone oxidoreductase chain 4 OS=Homo sapiens OX=9606 GN=MT-ND4 PE=1 SV=1	MT-ND4	1.006	0.932886
P03915	NADH-ubiquinone oxidoreductase chain 5 OS=Homo sapiens OX=9606 GN=MT-ND5 PE=1 SV=2	MT-ND5	0.846	0.335949
P03928	ATP synthase protein 8 OS=Homo sapiens OX=9606 GN=MT-ATP8 PE=1 SV=1	MT-ATP8	1.149	0.34368
P04035	3-hydroxy-3-methylglutaryl-coenzyme A reductase OS=Homo sapiens OX=9606 GN=HMGCR PE=1 SV=1	HMGCR	1.024	0.783904
P04040	Catalase OS=Homo sapiens OX=9606 GN=CAT PE=1 SV=3	CAT	0.985	0.939156
P04062	Lysosomal acid glucosylceramidase OS=Homo sapiens OX=9606 GN=GBA PE=1	GBA	1.127	0.045112
P04075	Fructose-bisphosphate aldolase A OS=Homo sapiens OX=9606 GN=ALDOA PE=1 SV=2	ALDOA	0.978	0.78431
P04080	Cystatin-B OS=Homo sapiens OX=9606 GN=CSTB PE=1 SV=2	CSTB	1.009	0.946683
P04083	Annexin A1 OS=Homo sapiens OX=9606 GN=ANXA1 PE=1	ANXA1	1	0.974316
P04114	Apolipoprotein B-100 OS=Homo sapiens OX=9606 GN=APOB PE=1 SV=2	APOB	1.208	0.100656
P04156	Major prion protein OS=Homo sapiens OX=9606 GN=PRNP PE=1 SV=1	PRNP	1.119	0.206002
P04179	Superoxide dismutase [Mn], mitochondrial OS=Homo sapiens OX=9606 GN=SOD2 PE=1 SV=3	SOD2	1.137	0.291046
P04181	Ornithine aminotransferase, mitochondrial OS=Homo sapiens OX=9606 GN=OAT PE=1 SV=1	OAT	0.956	0.826326
P04183	Thymidine kinase, cytosolic OS=Homo sapiens OX=9606 GN=TK1 PE=1 SV=2	TK1	0.95	0.621671

P04350	Tubulin beta-4A chain OS=Homo sapiens OX=9606 GN=TUBB4A PE=1 SV=2	TUBB4A	1.335	0.107098
P04406	Glyceraldehyde-3-phosphate dehydrogenase OS=Homo sapiens OX=9606 GN=GAPDH PE=1	GAPDH	1.067	0.59992
P04439	HLA class I histocompatibility antigen, A alpha chain OS=Homo sapiens OX=9606 GN=HLA-A PE=1 SV=2	HLA-A	1.109	0.132308
P04626	Receptor tyrosine-protein kinase erbB-2 OS=Homo sapiens OX=9606 GN=ERBB2 PE=1	ERBB2	0.935	0.189318
P04632	Calpain small subunit 1 OS=Homo sapiens OX=9606 GN=CAPNS1 PE=1 SV=1	CAPNS1	1.255	0.082668
P04637	Cellular tumor antigen p53 OS=Homo sapiens OX=9606 GN=TP53 PE=1 SV=4	TP53	1.016	
P04792	Heat shock protein beta-1 OS=Homo sapiens OX=9606 GN=HSPB1 PE=1 SV=2	HSPB1	1.3	0.071573
P04843	Dolichyl-diphosphooligosaccharide--protein glycosyltransferase subunit 1 OS=Homo sapiens OX=9606	RPN1	1.001	0.977553
P04844	Dolichyl-diphosphooligosaccharide--protein glycosyltransferase subunit 2 OS=Homo sapiens OX=9606	RPN2	0.996	0.878535
P04899	Guanine nucleotide-binding protein G(i) subunit alpha-2 OS=Homo sapiens OX=9606 GN=GNAI2 PE=1 SV=3	GNAI2	1.021	0.690574
P04920	Anion exchange protein 2 OS=Homo sapiens OX=9606 GN=SLC4A2 PE=1 SV=4	SLC4A2	1.176	0.260169
P05023	Sodium/potassium-transporting ATPase subunit alpha-1 OS=Homo sapiens OX=9606 GN=ATP1A1 PE=1 SV=1	ATP1A1	0.988	0.732822
P05026	Sodium/potassium-transporting ATPase subunit beta-1 OS=Homo sapiens OX=9606 GN=ATP1B1 PE=1 SV=1	ATP1B1	1.013	0.707625
P05067	Amyloid-beta precursor protein OS=Homo sapiens OX=9606 GN=APP PE=1 SV=3	APP	1.087	0.473635
P05114	Non-histone chromosomal protein HMG-14 OS=Homo sapiens OX=9606 GN=HMGN1 PE=1	HMGN1	0.977	0.919601

P05120	Plasminogen activator inhibitor 2 OS=Homo sapiens OX=9606 GN=SERPINB2 PE=1 SV=2	SERPINB2	0.903	0.079895
P05121	Plasminogen activator inhibitor 1 OS=Homo sapiens OX=9606 GN=SERPINE1 PE=1 SV=1	SERPINE1	1.089	0.508241
P05141	ADP/ATP translocase 2 OS=Homo sapiens OX=9606 GN=SLC25A5 PE=1 SV=7	SLC25A5	1.041	0.47304
P05166	Propionyl-CoA carboxylase beta chain, mitochondrial OS=Homo sapiens OX=9606 GN=PCCB PE=1 SV=3	PCCB	1.146	0.230841
P05198	Eukaryotic translation initiation factor 2 subunit 1 OS=Homo sapiens OX=9606 GN=EIF2S1	EIF2S1	1.084	0.306969
P05204	Non-histone chromosomal protein HMG-17 OS=Homo sapiens OX=9606 GN=HMGN2 PE=1	HMGN2	0.217	
P05386	60S acidic ribosomal protein P1 OS=Homo sapiens OX=9606 GN=RPLP1 PE=1 SV=1	RPLP1	1.033	0.666099
P05387	60S acidic ribosomal protein P2 OS=Homo sapiens OX=9606 GN=RPLP2 PE=1 SV=1	RPLP2	0.911	0.481115
P05388	60S acidic ribosomal protein P0 OS=Homo sapiens OX=9606 GN=RPLP0 PE=1 SV=1	RPLP0	1.021	0.598983
P05455	Lupus La protein OS=Homo sapiens OX=9606 GN=SSB PE=1	SSB	0.939	0.405772
P05556	Integrin beta-1 OS=Homo sapiens OX=9606 GN=ITGB1 PE=1 SV=2	ITGB1	0.96	0.68435
P05783	Keratin, type I cytoskeletal 18 OS=Homo sapiens OX=9606 GN=KRT18 PE=1 SV=2	KRT18	1.069	0.294819
P06132	Uroporphyrinogen decarboxylase OS=Homo sapiens OX=9606 GN=UROD PE=1 SV=2	UROD	0.804	0.250404
P06280	Alpha-galactosidase A OS=Homo sapiens OX=9606 GN=GLA PE=1 SV=1	GLA	0.991	0.837275
P06396	Gelsolin OS=Homo sapiens OX=9606 GN=GSN PE=1 SV=1	GSN	1.349	0.079641
P06400	Retinoblastoma-associated protein OS=Homo sapiens OX=9606 GN=RB1 PE=1 SV=2	RB1	0.768	
P06454	Prothymosin alpha OS=Homo sapiens OX=9606 GN=PTMA PE=1 SV=2	PTMA	0.859	0.452983
P06493	Cyclin-dependent kinase 1 OS=Homo sapiens OX=9606 GN=CDK1 PE=1 SV=3	CDK1	1.157	0.468034

P06576	ATP synthase subunit beta, mitochondrial OS=Homo sapiens OX=9606 GN=ATP5F1B PE=1 SV=3	ATP5F1B	1.007	0.867388
P06703	Protein S100-A6 OS=Homo sapiens OX=9606 GN=S100A6	S100A6	1.123	0.577688
P06730	Eukaryotic translation initiation factor 4E OS=Homo sapiens OX=9606 GN=EIF4E PE=1 SV=2	EIF4E	1.171	0.132279
P06733	Alpha-enolase OS=Homo sapiens OX=9606 GN=ENO1 PE=1 SV=2	ENO1	0.965	0.721497
P06737	Glycogen phosphorylase, liver form OS=Homo sapiens OX=9606 GN=PYGL PE=1 SV=4	PYGL	1.075	0.666733
P06744	Glucose-6-phosphate isomerase OS=Homo sapiens OX=9606 GN=GPI PE=1 SV=4	GPI	0.994	0.88716
P06748	Nucleophosmin OS=Homo sapiens OX=9606 GN=NPM1 PE=1 SV=2	NPM1	0.86	0.118205
P06753	Tropomyosin alpha-3 chain OS=Homo sapiens OX=9606 GN=TPM3 PE=1 SV=2	TPM3	0.902	0.070838
P06756	Integrin alpha-V OS=Homo sapiens OX=9606 GN=ITGAV	ITGAV	0.955	0.575789
P06865	Beta-hexosaminidase subunit alpha OS=Homo sapiens OX=9606 GN=HEXA PE=1 SV=2	HEXA	1.111	0.218282
P07099	Epoxide hydrolase 1 OS=Homo sapiens OX=9606 GN=EPHX1 PE=1 SV=1	EPHX1	1.012	0.769484
P07108	Acyl-CoA-binding protein OS=Homo sapiens OX=9606 GN=DBI PE=1 SV=2	DBI	0.935	0.67916
P07195	L-lactate dehydrogenase B chain OS=Homo sapiens OX=9606 GN=LDHB PE=1 SV=2	LDHB	0.963	0.659186
P07237	Protein disulfide-isomerase OS=Homo sapiens OX=9606 GN=P4HB PE=1 SV=3	P4HB	1.021	0.489462
P07339	Cathepsin D OS=Homo sapiens OX=9606 GN=CTSD PE=1 SV=1	CTSD	0.998	0.926813
P07355	Annexin A2 OS=Homo sapiens OX=9606 GN=ANXA2 PE=1	ANXA2	0.917	0.410004
P07384	Calpain-1 catalytic subunit OS=Homo sapiens OX=9606 GN=CAPN1 PE=1 SV=1	CAPN1	1.211	0.044265
P07437	Tubulin beta chain OS=Homo sapiens OX=9606 GN=TUBB PE=1 SV=2	TUBB	1.112	0.544378
P07602	Prosaposin OS=Homo sapiens OX=9606 GN=PSAP PE=1 SV=2	PSAP	1.142	0.068533

P07686	Beta-hexosaminidase subunit beta OS=Homo sapiens OX=9606 GN=HEXB PE=1 SV=3	HEXB	0.981	0.810029
P07711	Procathepsin L OS=Homo sapiens OX=9606 GN=CTSL PE=1 SV=2	CTSL	0.934	0.656248
P07741	Adenine phosphoribosyltransferase OS=Homo sapiens OX=9606 GN=APRT PE=1 SV=2	APRT	0.919	0.617371
P07814	Bifunctional glutamate/proline--tRNA ligase OS=Homo sapiens OX=9606 GN=EPRS1 PE=1	EPRS1	0.949	0.436536
P07858	Cathepsin B OS=Homo sapiens OX=9606 GN=CTSB PE=1 SV=3	CTSB	1.118	0.266742
P07900	Heat shock protein HSP 90-alpha OS=Homo sapiens OX=9606 GN=HSP90AA1 PE=1 SV=5	HSP90AA1	1	0.998049
P07910	Heterogeneous nuclear ribonucleoproteins C1/C2 OS=Homo sapiens OX=9606 GN=HNRNPC PE=1 SV=4	HNRNPC	0.943	0.371853
P07919	Cytochrome b-c1 complex subunit 6, mitochondrial OS=Homo sapiens OX=9606 GN=UQCRH	UQCRH	1.209	0.023891
P07942	Laminin subunit beta-1 OS=Homo sapiens OX=9606 GN=LAMB1 PE=1 SV=2	LAMB1	0.992	0.823431
P07947	Tyrosine-protein kinase Yes OS=Homo sapiens OX=9606 GN=YES1 PE=1 SV=3	YES1	0.971	0.452117
P07948	Tyrosine-protein kinase Lyn OS=Homo sapiens OX=9606 GN=LYN PE=1 SV=3	LYN	0.938	0.463465
P07954	Fumarate hydratase, mitochondrial OS=Homo sapiens OX=9606 GN=FH PE=1 SV=3	FH	0.977	0.683214
P07996	Thrombospondin-1 OS=Homo sapiens OX=9606 GN=THBS1 PE=1 SV=2	THBS1	1.006	0.882642
P08047	Transcription factor Sp1 OS=Homo sapiens OX=9606 GN=SP1 PE=1 SV=3	SP1	0.908	0.450513
P08069	Insulin-like growth factor 1 receptor OS=Homo sapiens OX=9606 GN=IGF1R PE=1 SV=1	IGF1R	1.007	0.956507
P08134	Rho-related GTP-binding protein RhoC OS=Homo sapiens OX=9606 GN=RHOC PE=1 SV=1	RHOC	1.452	
P08174	Complement decay-accelerating factor OS=Homo sapiens OX=9606 GN=CD55 PE=1 SV=4	CD55	1.091	0.529432
P08195	4F2 cell-surface antigen heavy chain OS=Homo sapiens OX=9606 GN=SLC3A2 PE=1	SLC3A2	1.026	0.550625

P08236	Beta-glucuronidase OS=Homo sapiens OX=9606 GN=GUSB PE=1 SV=2	GUSB	0.888	0.573975
P08237	ATP-dependent 6-phosphofructokinase, muscle type OS=Homo sapiens OX=9606 GN=PFKM PE=1 SV=2	PFKM	1.018	
P08238	Heat shock protein HSP 90-beta OS=Homo sapiens OX=9606 GN=HSP90AB1 PE=1 SV=4	HSP90AB1	1.06	0.568174
P08240	Signal recognition particle receptor subunit alpha OS=Homo sapiens OX=9606 GN=SRPRA PE=1	SRPRA	0.896	0.234118
P08243	Asparagine synthetase [glutamine-hydrolyzing] OS=Homo sapiens OX=9606 GN=ASNS PE=1 SV=4	ASNS	1.168	0.513757
P08397	Porphobilinogen deaminase OS=Homo sapiens OX=9606 GN=HMBS PE=1 SV=2	HMBS	0.694	0.319978
P08559	Pyruvate dehydrogenase E1 component subunit alpha, somatic form, mitochondrial OS=Homo sapiens OX=9606 GN=PDHA1 PE=1 SV=3	PDHA1	1.046	0.576489
P08574	Cytochrome c1, heme protein, mitochondrial OS=Homo sapiens OX=9606 GN=CYC1 PE=1 SV=3	CYC1	0.928	0.147136
P08579	U2 small nuclear ribonucleoprotein B" OS=Homo sapiens OX=9606 GN=SNRPB2 PE=1 SV=1	SNRPB2	0.882	0.13566
P08581	Hepatocyte growth factor receptor OS=Homo sapiens OX=9606 GN=MET PE=1 SV=4	MET	1.016	0.763735
P08621	U1 small nuclear ribonucleoprotein 70 kDa OS=Homo sapiens OX=9606 GN=SNRNP70 PE=1 SV=2	SNRNP70	0.956	0.353115
P08651	Nuclear factor 1 C-type OS=Homo sapiens OX=9606 GN=NFIC PE=1 SV=2	NFIC	0.839	
P08708	40S ribosomal protein S17 OS=Homo sapiens OX=9606 GN=RPS17 PE=1 SV=2	RPS17	0.984	0.808344
P08754	Guanine nucleotide-binding protein G(i) subunit alpha-3 OS=Homo sapiens OX=9606 GN=GNAI3 PE=1 SV=3	GNAI3	0.955	0.525916
P08758	Annexin A5 OS=Homo sapiens OX=9606 GN=ANXA5 PE=1	ANXA5	1.032	0.883303
P08865	40S ribosomal protein SA OS=Homo sapiens OX=9606 GN=RPSA PE=1 SV=4	RPSA	0.865	0.22196

P08962	CD63 antigen OS=Homo sapiens OX=9606 GN=CD63 PE=1 SV=2	CD63	1.014	0.951665
P09001	39S ribosomal protein L3, mitochondrial OS=Homo sapiens OX=9606 GN=MRPL3 PE=1	MRPL3	1.016	0.953519
P09012	U1 small nuclear ribonucleoprotein A OS=Homo sapiens OX=9606 GN=SNRPA PE=1 SV=3	SNRPA	0.873	0.059716
P09110	3-ketoacyl-CoA thiolase, peroxisomal OS=Homo sapiens OX=9606 GN=ACAA1 PE=1	ACAA1	1.029	0.641433
P09132	Signal recognition particle 19 kDa protein OS=Homo sapiens OX=9606 GN=SRP19 PE=1 SV=3	SRP19	1.33	0.527005
P09234	U1 small nuclear ribonucleoprotein C OS=Homo sapiens OX=9606 GN=SNRPC PE=1 SV=1	SNRPC	0.983	0.878312
P09382	Galectin-1 OS=Homo sapiens OX=9606 GN=LGALS1 PE=1 SV=2	LGALS1	0.906	0.492207
P09429	High mobility group protein B1 OS=Homo sapiens OX=9606 GN=HMGB1 PE=1 SV=3	HMGB1	0.827	0.23536
P09455	Retinol-binding protein 1 OS=Homo sapiens OX=9606 GN=RBP1 PE=1 SV=2	RBP1	1.103	0.541091
P09493	Tropomyosin alpha-1 chain OS=Homo sapiens OX=9606 GN=TPM1 PE=1 SV=2	TPM1	0.947	0.677985
P09496	Clathrin light chain A OS=Homo sapiens OX=9606 GN=CLTA PE=1 SV=1	CLTA	1.002	0.984185
P09497	Clathrin light chain B OS=Homo sapiens OX=9606 GN=CLTB PE=1 SV=1	CLTB	1.318	0.038073
P09525	Annexin A4 OS=Homo sapiens OX=9606 GN=ANXA4 PE=1	ANXA4	1.067	0.78118
P09543	2',3'-cyclic-nucleotide 3'-phosphodiesterase OS=Homo sapiens OX=9606 GN=CNP PE=1 SV=2	CNP	0.96	0.500484
P09601	Heme oxygenase 1 OS=Homo sapiens OX=9606 GN=HMOX1 PE=1 SV=1	HMOX1	1.142	0.549764
P09622	Dihydrolipoyl dehydrogenase, mitochondrial OS=Homo sapiens OX=9606 GN=DLD PE=1 SV=2	DLD	0.963	0.492791
P09651	Heterogeneous nuclear ribonucleoprotein A1 OS=Homo sapiens OX=9606 GN=HNRNPA1 PE=1 SV=5	HNRNPA1	0.938	0.134842

P09661	U2 small nuclear ribonucleoprotein A' OS=Homo sapiens OX=9606 GN=SNRPA1 PE=1 SV=2	SNRPA1	1.018	0.775195
P09668	Pro-cathepsin H OS=Homo sapiens OX=9606 GN=CTSH	CTSH	1.08	0.242747
P09669	Cytochrome c oxidase subunit 6C OS=Homo sapiens OX=9606 GN=COX6C PE=1 SV=2	COX6C	1.011	0.85597
P09758	Tumor-associated calcium signal transducer 2 OS=Homo sapiens OX=9606 GN=TACSTD2 PE=1 SV=3	TACSTD2	0.929	0.329622
P09874	Poly [ADP-ribose] polymerase 1 OS=Homo sapiens OX=9606 GN=PARP1 PE=1 SV=4	PARP1	0.825	0.142412
P09960	Leukotriene A-4 hydrolase OS=Homo sapiens OX=9606 GN=LTA4H PE=1 SV=2	LTA4H	0.931	0.509346
P09972	Fructose-bisphosphate aldolase C OS=Homo sapiens OX=9606 GN=ALDOC PE=1 SV=2	ALDOC	0.794	0.508781
Q71UI9	Histone H2A.V OS=Homo sapiens OX=9606 GN=H2AZ2 PE=1	H2AZ2	0.984	0.829716
P0CG12	Decreased expression in renal and prostate cancer protein OS=Homo sapiens OX=9606 GN=DERPC PE=1 SV=1	DERPC	0.84	0.349043
P0DI83	Ras-related protein Rab-34, isoform NARR OS=Homo sapiens OX=9606 GN=RAB34 PE=1	RAB34	0.987	0.80416
Q5DJT8	Cancer/testis antigen family 45 member A2 OS=Homo sapiens OX=9606 GN=CT45A2 PE=2	CT45A2	0.921	0.64541
P0DMV9	Heat shock 70 kDa protein 1B OS=Homo sapiens OX=9606 GN=HSPA1B PE=1 SV=1	HSPA1B	0.962	0.653402
P0DP25	Calmodulin-3 OS=Homo sapiens OX=9606 GN=CALM3 PE=1	CALM3	0.892	0.050725
P0DPB6	DNA-directed RNA polymerases I and III subunit RPAC2 OS=Homo sapiens OX=9606 GN=POLR1D PE=1 SV=1	POLR1D	1.091	0.474696
P10109	Adrenodoxin, mitochondrial OS=Homo sapiens OX=9606 GN=FDX1 PE=1 SV=1	FDX1	1.024	0.901851
P10155	60 kDa SS-A/Ro ribonucleoprotein OS=Homo sapiens OX=9606 GN=RO60 PE=1 SV=2	RO60	1.076	0.498433
P10301	Ras-related protein R-Ras OS=Homo sapiens OX=9606 GN=RRAS PE=1 SV=1	RRAS	0.961	0.518495

P10321	HLA class I histocompatibility antigen, C alpha chain OS=Homo sapiens OX=9606 GN=HLA-C PE=1 SV=3	HLA-C	0.986	0.846183
P10515	Dihydrolipoyllysine-residue acetyltransferase component of pyruvate dehydrogenase complex, mitochondrial OS=Homo sapiens OX=9606 GN=DLAT PE=1 SV=3	DLAT	0.961	0.509207
P10586	Receptor-type tyrosine-protein phosphatase F OS=Homo sapiens OX=9606 GN=PTPRF PE=1	PTPRF	0.955	0.045833
P10599	Thioredoxin OS=Homo sapiens OX=9606 GN=TXN PE=1 SV=3	TXN	0.978	0.867321
P10606	Cytochrome c oxidase subunit 5B, mitochondrial OS=Homo sapiens OX=9606 GN=COX5B PE=1	COX5B	1.015	0.811428
P10619	Lysosomal protective protein OS=Homo sapiens OX=9606 GN=CTSA PE=1 SV=2	CTSA	1.107	0.03945
P10644	cAMP-dependent protein kinase type I-alpha regulatory subunit OS=Homo sapiens OX=9606 GN=PRKAR1A PE=1 SV=1	PRKAR1A	1.096	0.175046
P10768	S-formylglutathione hydrolase OS=Homo sapiens OX=9606 GN=ESD PE=1 SV=2	ESD	0.943	0.590724
P10809	60 kDa heat shock protein, mitochondrial OS=Homo sapiens OX=9606 GN=HSPD1 PE=1	HSPD1	1.068	0.354028
P10909	Clusterin OS=Homo sapiens OX=9606 GN=CLU PE=1 SV=1	CLU	1.064	0.459721
P11021	Endoplasmic reticulum chaperone BiP OS=Homo sapiens OX=9606 GN=HSPA5 PE=1 SV=2	HSPA5	1.008	0.934156
P11047	Laminin subunit gamma-1 OS=Homo sapiens OX=9606 GN=LAMC1 PE=1 SV=3	LAMC1	0.94	0.591149
P11117	Lysosomal acid phosphatase OS=Homo sapiens OX=9606 GN=ACP2 PE=1 SV=3	ACP2	1.135	0.188646
P11142	Heat shock cognate 71 kDa protein OS=Homo sapiens OX=9606 GN=HSPA8 PE=1 SV=1	HSPA8	0.97	0.671631
P11166	Solute carrier family 2, facilitated glucose transporter member 1 OS=Homo sapiens OX=9606 GN=SLC2A1 PE=1 SV=2	SLC2A1	1.001	0.95578
P11172	Uridine 5'-monophosphate synthase OS=Homo sapiens OX=9606 GN=UMPS PE=1 SV=1	UMPS	1.015	0.805411

P11177	Pyruvate dehydrogenase E1 component subunit beta, mitochondrial OS=Homo sapiens OX=9606 GN=PDHB PE=1 SV=3	PDHB	1.029	0.567994
P11182	Lipoamide acyltransferase component of branched-chain alpha-keto acid dehydrogenase complex, mitochondrial OS=Homo sapiens OX=9606 GN=DBT PE=1	DBT	1.049	0.495379
P11216	Glycogen phosphorylase, brain form OS=Homo sapiens OX=9606 GN=PYGB PE=1 SV=5	PYGB	1.229	0.074747
P11233	Ras-related protein Ral-A OS=Homo sapiens OX=9606 GN=RALA PE=1 SV=1	RALA	0.941	0.289833
P11234	Ras-related protein Ral-B OS=Homo sapiens OX=9606 GN=RALB PE=1 SV=1	RALB	0.935	0.462759
P11279	Lysosome-associated membrane glycoprotein 1 OS=Homo sapiens OX=9606 GN=LAMP1 PE=1	LAMP1	1.125	0.193407
P11310	Medium-chain specific acyl-CoA dehydrogenase, mitochondrial OS=Homo sapiens OX=9606 GN=ACADM PE=1 SV=1	ACADM	0.936	0.085363
P11387	DNA topoisomerase 1 OS=Homo sapiens OX=9606 GN=TOP1 PE=1 SV=2	TOP1	0.932	0.586412
P11388	DNA topoisomerase 2-alpha OS=Homo sapiens OX=9606 GN=TOP2A PE=1 SV=3	TOP2A	0.988	0.842786
P11413	Glucose-6-phosphate 1-dehydrogenase OS=Homo sapiens OX=9606 GN=G6PD PE=1 SV=4	G6PD	1.11	0.148475
P11498	Pyruvate carboxylase, mitochondrial OS=Homo sapiens OX=9606 GN=PC PE=1 SV=2	PC	1.129	0.203125
P11586	C-1-tetrahydrofolate synthase, cytoplasmic OS=Homo sapiens OX=9606 GN=MTHFD1 PE=1 SV=4	MTHFD1	1.09	0.405982
P11717	Cation-independent mannose-6-phosphate receptor OS=Homo sapiens OX=9606 GN=IGF2R PE=1 SV=3	IGF2R	1.065	0.488032
P11766	Alcohol dehydrogenase class-3 OS=Homo sapiens OX=9606 GN=ADH5 PE=1 SV=4	ADH5	0.933	0.577003
P11908	Ribose-phosphate pyrophosphokinase 2 OS=Homo sapiens OX=9606 GN=PRPS2 PE=1 SV=2	PRPS2	1.058	0.642055

P11940	Polyadenylate-binding protein 1 OS=Homo sapiens OX=9606 GN=PABPC1 PE=1 SV=2	PABPC1	1.022	0.69148
P12004	Proliferating cell nuclear antigen OS=Homo sapiens OX=9606 GN=PCNA PE=1 SV=1	PCNA	0.819	0.135317
P12074	Cytochrome c oxidase subunit 6A1, mitochondrial OS=Homo sapiens OX=9606 GN=COX6A1 PE=1 SV=4	COX6A1	1.003	0.944449
P12081	Histidine--tRNA ligase, cytoplasmic OS=Homo sapiens OX=9606 GN=HARS1 PE=1	HARS1	1.078	0.581674
P12235	ADP/ATP translocase 1 OS=Homo sapiens OX=9606 GN=SLC25A4 PE=1 SV=4	SLC25A4	1.014	0.865448
P12236	ADP/ATP translocase 3 OS=Homo sapiens OX=9606 GN=SLC25A6 PE=1 SV=4	SLC25A6	1.042	0.672797
P12268	Inosine-5'-monophosphate dehydrogenase 2 OS=Homo sapiens OX=9606 GN=IMPDH2 PE=1 SV=2	IMPDH2	1.215	0.06866
P12270	Nucleoprotein TPR OS=Homo sapiens OX=9606 GN=TPR PE=1 SV=3	TPR	0.948	0.465121
P12429	Annexin A3 OS=Homo sapiens OX=9606 GN=ANXA3 PE=1	ANXA3	1.097	0.419432
P12532	Creatine kinase U-type, mitochondrial OS=Homo sapiens OX=9606 GN=CKMT1A PE=1 SV=1	CKMT1A	0.933	0.345094
P12814	Alpha-actinin-1 OS=Homo sapiens OX=9606 GN=ACTN1 PE=1	ACTN1	1.162	0.315087
P12830	Cadherin-1 OS=Homo sapiens OX=9606 GN=CDH1 PE=1 SV=3	CDH1	0.925	0.272715
P12931	Proto-oncogene tyrosine-protein kinase Src OS=Homo sapiens OX=9606 GN=SRC PE=1 SV=3	SRC	1.04	0.541295
P12955	Xaa-Pro dipeptidase OS=Homo sapiens OX=9606 GN=PEPD PE=1 SV=3	PEPD	0.993	0.890146
P12956	X-ray repair cross-complementing protein 6 OS=Homo sapiens OX=9606 GN=XRCC6 PE=1	XRCC6	0.931	0.135133
P13010	X-ray repair cross-complementing protein 5 OS=Homo sapiens OX=9606 GN=XRCC5 PE=1	XRCC5	0.95	0.048641
P13073	Cytochrome c oxidase subunit 4 isoform 1, mitochondrial OS=Homo sapiens OX=9606 GN=COX4I1 PE=1 SV=1	COX4I1	0.968	0.61953

P13473	Lysosome-associated membrane glycoprotein 2 OS=Homo sapiens OX=9606 GN=LAMP2 PE=1	LAMP2	1.021	0.824151
P13489	Ribonuclease inhibitor OS=Homo sapiens OX=9606 GN=RNH1 PE=1 SV=2	RNH1	1.072	0.345852
P13639	Elongation factor 2 OS=Homo sapiens OX=9606 GN=EEF2 PE=1 SV=4	EEF2	1.078	0.613332
P13667	Protein disulfide-isomerase A4 OS=Homo sapiens OX=9606 GN=PDIA4 PE=1 SV=2	PDIA4	1.016	0.724828
P13674	Prolyl 4-hydroxylase subunit alpha-1 OS=Homo sapiens OX=9606 GN=P4HA1 PE=1	P4HA1	1.063	0.131597
P13693	Translationally-controlled tumor protein OS=Homo sapiens OX=9606 GN=TPT1 PE=1 SV=1	TPT1	1.032	0.873121
P13726	Tissue factor OS=Homo sapiens OX=9606 GN=F3 PE=1 SV=1	F3	1.136	0.275528
P13796	Plastin-2 OS=Homo sapiens OX=9606 GN=LCP1 PE=1 SV=6	LCP1	1.133	0.343465
P13797	Plastin-3 OS=Homo sapiens OX=9606 GN=PLS3 PE=1 SV=4	PLS3	1.097	0.436708
P13804	Electron transfer flavoprotein subunit alpha, mitochondrial OS=Homo sapiens OX=9606 GN=ETFA PE=1 SV=1	ETFA	1	0.986299
P13807	Glycogen [starch] synthase, muscle OS=Homo sapiens OX=9606 GN=GYS1 PE=1 SV=2	GYS1	1.126	
P13861	cAMP-dependent protein kinase type II-alpha regulatory subunit OS=Homo sapiens OX=9606 GN=PRKAR2A PE=1 SV=2	PRKAR2A	1.044	0.367174
P13928	Annexin A8 OS=Homo sapiens OX=9606 GN=ANXA8 PE=1	ANXA8	0.976	0.793187
P13984	General transcription factor IIF subunit 2 OS=Homo sapiens OX=9606 GN=GTF2F2 PE=1	GTF2F2	0.916	0.491435
P13987	CD59 glycoprotein OS=Homo sapiens OX=9606 GN=CD59 PE=1 SV=1	CD59	1.184	0.168741
P13995	Bifunctional methylenetetrahydrofolate dehydrogenase/cyclohydrolase, mitochondrial OS=Homo sapiens OX=9606 GN=MTHFD2 PE=1 SV=2	MTHFD2	1.037	0.502516
P14174	Macrophage migration inhibitory factor OS=Homo sapiens OX=9606 GN=MIF PE=1 SV=4	MIF	1.024	0.942168

P14209	CD99 antigen OS=Homo sapiens OX=9606 GN=CD99 PE=1 SV=1	CD99	0.904	0.537726
P14314	Glucosidase 2 subunit beta OS=Homo sapiens OX=9606 GN=PRKCSH PE=1 SV=2	PRKCSH	1.006	0.911458
P14406	Cytochrome c oxidase subunit 7A2, mitochondrial OS=Homo sapiens OX=9606 GN=COX7A2 PE=1 SV=1	COX7A2	0.993	
P14550	Aldo-keto reductase family 1 member A1 OS=Homo sapiens OX=9606 GN=AKR1A1 PE=1 SV=3	AKR1A1	1.023	0.860725
P14618	Pyruvate kinase PKM OS=Homo sapiens OX=9606 GN=PKM PE=1 SV=4	PKM	1.057	0.737648
P14625	Endoplasmin OS=Homo sapiens OX=9606 GN=HSP90B1 PE=1 SV=1	HSP90B1	1.059	0.322594
P14678	Small nuclear ribonucleoprotein-associated proteins B and B' OS=Homo sapiens OX=9606 GN=SNRPB PE=1 SV=2	SNRPB	0.925	0.183759
P14735	Insulin-degrading enzyme OS=Homo sapiens OX=9606 GN=IDE PE=1 SV=4	IDE	1.024	0.921745
P14854	Cytochrome c oxidase subunit 6B1 OS=Homo sapiens OX=9606 GN=COX6B1 PE=1 SV=2	COX6B1	1.086	0.016166
P14859	POU domain, class 2, transcription factor 1 OS=Homo sapiens OX=9606 GN=POU2F1 PE=1	POU2F1	0.957	
P14866	Heterogeneous nuclear ribonucleoprotein L OS=Homo sapiens OX=9606 GN=HNRNPL PE=1 SV=2	HNRNPL	0.934	0.014989
P14868	Aspartate--tRNA ligase, cytoplasmic OS=Homo sapiens OX=9606 GN=DARS1 PE=1	DARS1	0.954	0.617857
P14923	Junction plakoglobin OS=Homo sapiens OX=9606 GN=JUP PE=1 SV=3	JUP	0.96	0.257386
P14927	Cytochrome b-c1 complex subunit 7 OS=Homo sapiens OX=9606 GN=UQCRB PE=1 SV=2	UQCRB	1.035	0.576741
P15121	Aldo-keto reductase family 1 member B1 OS=Homo sapiens OX=9606 GN=AKR1B1 PE=1 SV=3	AKR1B1	0.933	0.491942
P15151	Poliovirus receptor OS=Homo sapiens OX=9606 GN=PVR PE=1 SV=2	PVR	1.173	0.162349

P15153	Ras-related C3 botulinum toxin substrate 2 OS=Homo sapiens OX=9606 GN=RAC2 PE=1 SV=1	RAC2	0.961	0.620384
P15170	Eukaryotic peptide chain release factor GTP-binding subunit ERF3A OS=Homo sapiens OX=9606 GN=GSPT1 PE=1	GSPT1	1.105	0.145021
P15260	Interferon gamma receptor 1 OS=Homo sapiens OX=9606 GN=IFNGR1 PE=1 SV=1	IFNGR1	0.92	0.306363
P15291	Beta-1,4-galactosyltransferase 1 OS=Homo sapiens OX=9606 GN=B4GALT1 PE=1 SV=5	B4GALT1	1.102	0.052449
P15311	Ezrin OS=Homo sapiens OX=9606 GN=EZR PE=1 SV=4	EZR	1.097	0.300538
P15374	Ubiquitin carboxyl-terminal hydrolase isozyme L3 OS=Homo sapiens OX=9606 GN=UCHL3 PE=1 SV=1	UCHL3	1.066	0.641966
P15529	Membrane cofactor protein OS=Homo sapiens OX=9606 GN=CD46 PE=1 SV=3	CD46	1.044	0.593346
P15531	Nucleoside diphosphate kinase A OS=Homo sapiens OX=9606 GN=NME1 PE=1 SV=1	NME1	0.961	0.694794
P15559	NAD(P)H dehydrogenase [quinone] 1 OS=Homo sapiens OX=9606 GN=NQO1 PE=1 SV=1	NQO1	0.971	0.802698
P15586	N-acetylglucosamine-6-sulfatase OS=Homo sapiens OX=9606 GN=GNS PE=1 SV=3	GNS	1.059	0.495362
P15880	40S ribosomal protein S2 OS=Homo sapiens OX=9606 GN=RPS2 PE=1 SV=2	RPS2	1.116	0.324163
P15924	Desmoplakin OS=Homo sapiens OX=9606 GN=DSP PE=1 SV=3	DSP	0.991	0.839371
P15927	Replication protein A 32 kDa subunit OS=Homo sapiens OX=9606 GN=RPA2 PE=1 SV=1	RPA2	0.974	0.881355
P15954	Cytochrome c oxidase subunit 7C, mitochondrial OS=Homo sapiens OX=9606 GN=COX7C PE=1	COX7C	1.92	0.225918
P16070	CD44 antigen OS=Homo sapiens OX=9606 GN=CD44 PE=1 SV=3	CD44	1.025	0.62393
P16104	Histone H2AX OS=Homo sapiens OX=9606 GN=H2AX PE=1 SV=2	H2AX	1.205	0.428852
P16144	Integrin beta-4 OS=Homo sapiens OX=9606 GN=ITGB4 PE=1 SV=5	ITGB4	1.013	0.798727
P16152	Carbonyl reductase [NADPH] 1 OS=Homo sapiens OX=9606 GN=CBR1 PE=1 SV=3	CBR1	0.899	0.49565

P18846	Cyclic AMP-dependent transcription factor ATF-1 OS=Homo sapiens OX=9606	ATF1	0.954	
P16278	Beta-galactosidase OS=Homo sapiens OX=9606 GN=GLB1 PE=1 SV=2	GLB1	1.112	0.188707
P16401	Histone H1.5 OS=Homo sapiens OX=9606 GN=H1-5 PE=1 SV=3	H1-5	1.207	0.715915
P16403	Histone H1.2 OS=Homo sapiens OX=9606 GN=H1-2 PE=1 SV=2	H1-2	1.258	0.648095
P16422	Epithelial cell adhesion molecule OS=Homo sapiens OX=9606 GN=EPCAM PE=1 SV=2	EPCAM	1.039	0.577415
P16435	NADPH--cytochrome P450 reductase OS=Homo sapiens OX=9606 GN=POR PE=1 SV=2	POR	1.034	0.52174
P16615	Sarcoplasmic/endoplasmic reticulum calcium ATPase 2 OS=Homo sapiens OX=9606 GN=ATP2A2 PE=1 SV=1	ATP2A2	0.963	0.480804
P16949	Stathmin OS=Homo sapiens OX=9606 GN=STMN1 PE=1	STMN1	0.975	0.795421
P16989	Y-box-binding protein 3 OS=Homo sapiens OX=9606 GN=YBX3 PE=1 SV=4	YBX3	0.906	0.41073
P17096	High mobility group protein HMG-I/HMG-Y OS=Homo sapiens OX=9606 GN=HMGA1 PE=1	HMGA1	0.893	0.224523
P17152	Transmembrane protein 11, mitochondrial OS=Homo sapiens OX=9606 GN=TMEM11 PE=1 SV=1	TMEM11	1.089	0.476615
P17174	Aspartate aminotransferase, cytoplasmic OS=Homo sapiens OX=9606 GN=GOT1 PE=1 SV=3	GOT1	0.989	0.971392
P17252	Protein kinase C alpha type OS=Homo sapiens OX=9606 GN=PRKCA PE=1 SV=4	PRKCA	0.857	0.007205
P17275	Transcription factor jun-B OS=Homo sapiens OX=9606 GN=JUNB PE=1 SV=1	JUNB	1.088	0.277045
P17301	Integrin alpha-2 OS=Homo sapiens OX=9606 GN=ITGA2 PE=1 SV=1	ITGA2	0.979	0.798086
P17480	Nucleolar transcription factor 1 OS=Homo sapiens OX=9606 GN=UBTF PE=1 SV=1	UBTF	0.857	0.333454
P17568	NADH dehydrogenase [ubiquinone] 1 beta subcomplex subunit 7 OS=Homo sapiens OX=9606 GN=NDUFB7 PE=1	NDUFB7	0.97	0.988505

P17612	cAMP-dependent protein kinase catalytic subunit alpha OS=Homo sapiens OX=9606 GN=PRKACA PE=1 SV=2	PRKACA	1.116	0.084131
P17655	Calpain-2 catalytic subunit OS=Homo sapiens OX=9606 GN=CAPN2 PE=1 SV=6	CAPN2	1.208	0.112832
P17676	CCAAT/enhancer-binding protein beta OS=Homo sapiens OX=9606 GN=CEBPB PE=1 SV=2	CEBPB	1.04	0.721438
P17706	Tyrosine-protein phosphatase non-receptor type 2 OS=Homo sapiens OX=9606 GN=PTPN2 PE=1	PTPN2	1.038	0.52749
P17812	CTP synthase 1 OS=Homo sapiens OX=9606 GN=CTPS1 PE=1	CTPS1	1.195	0.027389
P17844	Probable ATP-dependent RNA helicase DDX5 OS=Homo sapiens OX=9606 GN=DDX5 PE=1 SV=1	DDX5	0.877	0.001243
P17900	Ganglioside GM2 activator OS=Homo sapiens OX=9606 GN=GM2A PE=1 SV=4	GM2A	1.251	
P17931	Galectin-3 OS=Homo sapiens OX=9606 GN=LGALS3 PE=1 SV=5	LGALS3	0.885	0.307917
P17980	26S proteasome regulatory subunit 6A OS=Homo sapiens OX=9606 GN=PSMC3 PE=1 SV=3	PSMC3	1.027	0.647541
P17987	T-complex protein 1 subunit alpha OS=Homo sapiens OX=9606 GN=TCP1 PE=1 SV=1	TCP1	0.933	0.403935
P18031	Tyrosine-protein phosphatase non-receptor type 1 OS=Homo sapiens OX=9606 GN=PTPN1 PE=1	PTPN1	0.999	0.973188
P18077	60S ribosomal protein L35a OS=Homo sapiens OX=9606 GN=RPL35A PE=1 SV=2	RPL35A	1.375	0.015696
P18084	Integrin beta-5 OS=Homo sapiens OX=9606 GN=ITGB5 PE=1 SV=1	ITGB5	1.026	0.797557
P18085	ADP-ribosylation factor 4 OS=Homo sapiens OX=9606 GN=ARF4 PE=1 SV=3	ARF4	0.867	0.292479
P18124	60S ribosomal protein L7 OS=Homo sapiens OX=9606 GN=RPL7 PE=1 SV=1	RPL7	1.128	0.740991
P18206	Vinculin OS=Homo sapiens OX=9606 GN=VCL PE=1 SV=4	VCL	1.191	0.129599
P18564	Integrin beta-6 OS=Homo sapiens OX=9606 GN=ITGB6 PE=1 SV=2	ITGB6	0.961	0.703471
P18583	Protein SON OS=Homo sapiens OX=9606 GN=SON PE=1 SV=4	SON	1.017	0.834981
P18615	Negative elongation factor E OS=Homo sapiens OX=9606 GN=NELFE PE=1 SV=3	NELFE	0.906	0.445385

P18621	60S ribosomal protein L17 OS=Homo sapiens OX=9606 GN=RPL17 PE=1 SV=3	RPL17	1.036	0.821813
P18669	Phosphoglycerate mutase 1 OS=Homo sapiens OX=9606 GN=PGAM1 PE=1 SV=2	PGAM1	1.02	0.895835
P18754	Regulator of chromosome condensation OS=Homo sapiens OX=9606 GN=RCC1 PE=1 SV=1	RCC1	0.953	0.312158
P18827	Syndecan-1 OS=Homo sapiens OX=9606 GN=SDC1 PE=1 SV=3	SDC1	0.978	0.854796
P18858	DNA ligase 1 OS=Homo sapiens OX=9606 GN=LIG1 PE=1 SV=1	LIG1	1.088	0.707192
P18859	ATP synthase-coupling factor 6, mitochondrial OS=Homo sapiens OX=9606 GN=ATP5PF PE=1	ATP5PF	0.96	0.650555
P18887	DNA repair protein XRCC1 OS=Homo sapiens OX=9606 GN=XRCC1 PE=1 SV=2	XRCC1	0.843	0.06616
P19022	Cadherin-2 OS=Homo sapiens OX=9606 GN=CDH2 PE=1 SV=4	CDH2	1.008	0.899648
P19224	UDP-glucuronosyltransferase 1-6 OS=Homo sapiens OX=9606 GN=UGT1A6 PE=1 SV=2	UGT1A6	0.967	0.573555
P19338	Nucleolin OS=Homo sapiens OX=9606 GN=NCL PE=1 SV=3	NCL	0.9	0.050316
P19367	Hexokinase-1 OS=Homo sapiens OX=9606 GN=HK1 PE=1 SV=3	HK1	0.807	0.106415
P19387	DNA-directed RNA polymerase II subunit RPB3 OS=Homo sapiens OX=9606 GN=POLR2C PE=1 SV=2	POLR2C	1.067	0.617092
P19388	DNA-directed RNA polymerases I, II, and III subunit RPABC1 OS=Homo sapiens OX=9606 GN=POLR2E PE=1 SV=4	POLR2E	0.903	0.369434
P19404	NADH dehydrogenase [ubiquinone] flavoprotein 2, mitochondrial OS=Homo sapiens OX=9606 GN=NDUFV2 PE=1	NDUFV2	0.935	0.417243
P19438	Tumor necrosis factor receptor superfamily member 1A OS=Homo sapiens OX=9606 GN=TNFRSF1A PE=1 SV=1	TNFRSF1A	0.876	0.272249
P19447	General transcription and DNA repair factor IIH helicase subunit XPB OS=Homo sapiens OX=9606 GN=ERCC3 PE=1 SV=1	ERCC3	1.008	0.912478
P19525	Interferon-induced, double-stranded RNA-activated protein kinase OS=Homo sapiens OX=9606 GN=EIF2AK2 PE=1	EIF2AK2	1.002	0.945173

P19623	Spermidine synthase OS=Homo sapiens OX=9606 GN=SRM PE=1 SV=1	SRM	0.883	0.163448
P19634	Sodium/hydrogen exchanger 1 OS=Homo sapiens OX=9606 GN=SLC9A1 PE=1 SV=2	SLC9A1	0.967	0.531603
P19784	Casein kinase II subunit alpha' OS=Homo sapiens OX=9606 GN=CSNK2A2 PE=1 SV=1	CSNK2A2	0.917	0.255886
P19793	Retinoic acid receptor RXR-alpha OS=Homo sapiens OX=9606 GN=RXRA PE=1 SV=1	RXRA	0.604	0.000343
P20020	Plasma membrane calcium-transporting ATPase 1 OS=Homo sapiens OX=9606 GN=ATP2B1 PE=1 SV=4	ATP2B1	1.009	0.83444
P20042	Eukaryotic translation initiation factor 2 subunit 2 OS=Homo sapiens OX=9606 GN=EIF2S2	EIF2S2	1.047	0.575533
P20073	Annexin A7 OS=Homo sapiens OX=9606 GN=ANXA7 PE=1	ANXA7	0.974	0.658363
P20290	Transcription factor BTF3 OS=Homo sapiens OX=9606 GN=BTF3 PE=1 SV=1	BTF3	0.891	0.447062
P20339	Ras-related protein Rab-5A OS=Homo sapiens OX=9606 GN=RAB5A PE=1 SV=2	RAB5A	0.925	0.366229
P20340	Ras-related protein Rab-6A OS=Homo sapiens OX=9606 GN=RAB6A PE=1 SV=3	RAB6A	1.036	0.309874
P20585	DNA mismatch repair protein Msh3 OS=Homo sapiens OX=9606 GN=MSH3 PE=1 SV=4	MSH3	0.971	
P20618	Proteasome subunit beta type-1 OS=Homo sapiens OX=9606 GN=PSMB1 PE=1 SV=2	PSMB1	0.993	0.881475
P20645	Cation-dependent mannose-6-phosphate receptor OS=Homo sapiens OX=9606 GN=M6PR PE=1 SV=1	M6PR	1.063	0.331931
P20674	Cytochrome c oxidase subunit 5A, mitochondrial OS=Homo sapiens OX=9606 GN=COX5A PE=1	COX5A	1.068	0.372727
P20700	Lamin-B1 OS=Homo sapiens OX=9606 GN=LMNB1 PE=1	LMNB1	0.983	0.708085
P20810	Calpastatin OS=Homo sapiens OX=9606 GN=CAST PE=1 SV=4	CAST	1.142	0.033228
P20839	Inosine-5'-monophosphate dehydrogenase 1 OS=Homo sapiens OX=9606 GN=IMPDH1 PE=1 SV=2	IMPDH1	1.44	0.096035

P20908	Collagen alpha-1(V) chain OS=Homo sapiens OX=9606 GN=COL5A1 PE=1 SV=3	COL5A1	1.119	0.635032
P20962	Parathymosin OS=Homo sapiens OX=9606 GN=PTMS PE=1 SV=2	PTMS	1.093	0.715373
P21127	Cyclin-dependent kinase 11B OS=Homo sapiens OX=9606 GN=CDK11B PE=1 SV=4	CDK11B	0.946	0.398814
P21281	V-type proton ATPase subunit B, brain isoform OS=Homo sapiens OX=9606 GN=ATP6V1B2 PE=1 SV=3	ATP6V1B 2	1.137	0.009499
P21283	V-type proton ATPase subunit C 1 OS=Homo sapiens OX=9606 GN=ATP6V1C1 PE=1 SV=4	ATP6V1C 1	0.994	0.905207
P21291	Cysteine and glycine-rich protein 1 OS=Homo sapiens OX=9606 GN=CSRP1 PE=1 SV=3	CSRP1	0.972	0.751319
P21333	Filamin-A OS=Homo sapiens OX=9606 GN=FLNA PE=1 SV=4	FLNA	0.972	0.6411
P21397	Amine oxidase [flavin-containing] A OS=Homo sapiens OX=9606 GN=MAOA PE=1 SV=1	MAOA	0.989	0.905825
P21399	Cytoplasmic aconitate hydratase OS=Homo sapiens OX=9606 GN=ACO1 PE=1 SV=3	ACO1	1.14	0.220766
P21589	5'-nucleotidase OS=Homo sapiens OX=9606 GN=NT5E PE=1 SV=1	NT5E	1.081	0.219159
P21741	Midkine OS=Homo sapiens OX=9606 GN=MDK PE=1 SV=1	MDK	0.877	0.628083
P21796	Voltage-dependent anion-selective channel protein 1 OS=Homo sapiens OX=9606 GN=VDAC1	VDAC1	0.966	0.6295
P21912	Succinate dehydrogenase [ubiquinone] iron-sulfur subunit, mitochondrial OS=Homo sapiens OX=9606 GN=SDHB PE=1 SV=3	SDHB	0.972	0.758285
P21926	CD9 antigen OS=Homo sapiens OX=9606 GN=CD9 PE=1 SV=4	CD9	0.908	0.408201
P21964	Catechol O-methyltransferase OS=Homo sapiens OX=9606 GN=COMT PE=1 SV=2	COMT	1.048	0.277781
P21980	Protein-glutamine gamma-glutamyltransferase 2 OS=Homo sapiens OX=9606 GN=TGM2 PE=1 SV=2	TGM2	1.168	0.490895
P22033	Methylmalonyl-CoA mutase, mitochondrial OS=Homo sapiens OX=9606 GN=MMUT PE=1	MMUT	0.934	0.535436
P22059	Oxysterol-binding protein 1 OS=Homo sapiens OX=9606 GN=OSBP PE=1 SV=1	OSBP	1.254	0.109038

P22061	Protein-L-isoaspartate(D-aspartate) O-methyltransferase OS=Homo sapiens OX=9606 GN=PCMT1 PE=1 SV=4	PCMT1	1.008	0.906402
P22087	rRNA 2'-O-methyltransferase fibrillarin OS=Homo sapiens OX=9606 GN=FBL PE=1 SV=2	FBL	0.917	0.49201
P22102	Trifunctional purine biosynthetic protein adenosine-3 OS=Homo sapiens OX=9606 GN=GART PE=1 SV=1	GART	1.091	0.306262
P22223	Cadherin-3 OS=Homo sapiens OX=9606 GN=CDH3 PE=1 SV=2	CDH3	1.115	0.140039
P22234	Multifunctional protein ADE2 OS=Homo sapiens OX=9606 GN=PAICS PE=1 SV=3	PAICS	1.156	0.119405
P22307	Sterol carrier protein 2 OS=Homo sapiens OX=9606 GN=SCP2 PE=1 SV=2	SCP2	1.035	0.610997
P22314	Ubiquitin-like modifier-activating enzyme 1 OS=Homo sapiens OX=9606 GN=UBA1 PE=1 SV=3	UBA1	0.992	0.930442
P22392	Nucleoside diphosphate kinase B OS=Homo sapiens OX=9606 GN=NME2 PE=1 SV=1	NME2	1.004	0.97723
P22570	NADPH:adrenodoxin oxidoreductase, mitochondrial OS=Homo sapiens OX=9606 GN=FDXR PE=1 SV=3	FDXR	1.041	0.245818
P22626	Heterogeneous nuclear ribonucleoproteins A2/B1 OS=Homo sapiens OX=9606 GN=HNRNPA2B1 PE=1 SV=2	HNRNPA2B1	0.961	0.257412
P22695	Cytochrome b-c1 complex subunit 2, mitochondrial OS=Homo sapiens OX=9606 GN=UQCRC2 PE=1 SV=3	UQCRC2	0.997	0.994518
P22830	Ferrochelatase, mitochondrial OS=Homo sapiens OX=9606 GN=FECH PE=1 SV=2	FECH	1.025	
P23193	Transcription elongation factor A protein 1 OS=Homo sapiens OX=9606 GN=TCEA1 PE=1	TCEA1	0.811	0.221523
P23229	Integrin alpha-6 OS=Homo sapiens OX=9606 GN=ITGA6 PE=1 SV=5	ITGA6	1.018	0.64544
P23246	Splicing factor, proline- and glutamine-rich OS=Homo sapiens OX=9606 GN=SFPQ PE=1 SV=2	SFPQ	0.956	0.444802
P23258	Tubulin gamma-1 chain OS=Homo sapiens OX=9606 GN=TUBG1 PE=1 SV=2	TUBG1	1.05	0.620722

P23284	Peptidyl-prolyl cis-trans isomerase B OS=Homo sapiens OX=9606 GN=PPIB PE=1 SV=2	PPIB	0.894	0.173335
P23368	NAD-dependent malic enzyme, mitochondrial OS=Homo sapiens OX=9606 GN=ME2 PE=1 SV=1	ME2	0.981	0.828355
P23381	Tryptophan--tRNA ligase, cytoplasmic OS=Homo sapiens OX=9606 GN=WARS1 PE=1	WARS1	1.088	0.412291
P23396	40S ribosomal protein S3 OS=Homo sapiens OX=9606 GN=RPS3 PE=1 SV=2	RPS3	0.954	0.361539
P23434	Glycine cleavage system H protein, mitochondrial OS=Homo sapiens OX=9606 GN=GCSH PE=1 SV=2	GCSH	0.934	0.517566
P23458	Tyrosine-protein kinase JAK1 OS=Homo sapiens OX=9606 GN=JAK1 PE=1 SV=2	JAK1	1.016	0.923859
P23526	Adenosylhomocysteinase OS=Homo sapiens OX=9606 GN=AHCY PE=1 SV=4	AHCY	1.074	0.600252
Q16778	Histone H2B type 2-E OS=Homo sapiens OX=9606 GN=H2BC21 PE=1 SV=3	H2BC21	1.032	0.841066
P23528	Cofilin-1 OS=Homo sapiens OX=9606 GN=CFL1 PE=1 SV=3	CFL1	1.011	0.953559
P23588	Eukaryotic translation initiation factor 4B OS=Homo sapiens OX=9606 GN=EIF4B PE=1 SV=2	EIF4B	1.205	0.20633
P23771	Trans-acting T-cell-specific transcription factor GATA-3 OS=Homo sapiens OX=9606 GN=GATA3 PE=1 SV=1	GATA3	0.888	0.47423
P23786	Carnitine O-palmitoyltransferase 2, mitochondrial OS=Homo sapiens OX=9606 GN=CPT2 PE=1 SV=2	CPT2	0.997	0.95445
P23919	Thymidylate kinase OS=Homo sapiens OX=9606 GN=DTYMK PE=1 SV=4	DTYMK	0.989	0.875188
P24390	ER lumen protein-retaining receptor 1 OS=Homo sapiens OX=9606 GN=KDELR1 PE=1	KDELR1	1.074	0.670195
P24468	COUP transcription factor 2 OS=Homo sapiens OX=9606 GN=NR2F2 PE=1 SV=1	NR2F2	0.97	0.807209
P24534	Elongation factor 1-beta OS=Homo sapiens OX=9606 GN=EEF1B2 PE=1 SV=3	EEF1B2	1.074	0.400219
P24539	ATP synthase F(0) complex subunit B1, mitochondrial OS=Homo sapiens OX=9606 GN=ATP5PB PE=1 SV=2	ATP5PB	1.038	0.592844

P24666	Low molecular weight phosphotyrosine protein phosphatase OS=Homo sapiens OX=9606 GN=ACP1 PE=1 SV=3	ACP1	1.089	0.384765
P24752	Acetyl-CoA acetyltransferase, mitochondrial OS=Homo sapiens OX=9606 GN=ACAT1 PE=1	ACAT1	0.998	0.946063
P24821	Tenascin OS=Homo sapiens OX=9606 GN=TNC PE=1 SV=3	TNC	1.076	0.592199
P24928	DNA-directed RNA polymerase II subunit RPB1 OS=Homo sapiens OX=9606 GN=POLR2A PE=1 SV=2	POLR2A	0.985	0.562604
P24941	Cyclin-dependent kinase 2 OS=Homo sapiens OX=9606 GN=CDK2 PE=1 SV=2	CDK2	1.006	0.960869
P25205	DNA replication licensing factor MCM3 OS=Homo sapiens OX=9606 GN=MCM3 PE=1	MCM3	0.833	0.086108
P25398	40S ribosomal protein S12 OS=Homo sapiens OX=9606 GN=RPS12 PE=1 SV=3	RPS12	1.081	0.106885
P25440	Bromodomain-containing protein 2 OS=Homo sapiens OX=9606 GN=BRD2 PE=1 SV=2	BRD2	0.811	
P25490	Transcriptional repressor protein YY1 OS=Homo sapiens OX=9606 GN=YY1 PE=1 SV=2	YY1	0.957	0.378865
P25685	DnaJ homolog subfamily B member 1 OS=Homo sapiens OX=9606 GN=DNAJB1 PE=1	DNAJB1	1.068	0.334282
P25705	ATP synthase subunit alpha, mitochondrial OS=Homo sapiens OX=9606 GN=ATP5F1A PE=1 SV=1	ATP5F1A	0.956	0.350673
P25786	Proteasome subunit alpha type-1 OS=Homo sapiens OX=9606 GN=PSMA1 PE=1 SV=1	PSMA1	0.942	0.230208
P25787	Proteasome subunit alpha type-2 OS=Homo sapiens OX=9606 GN=PSMA2 PE=1 SV=2	PSMA2	0.968	0.671728
P25788	Proteasome subunit alpha type-3 OS=Homo sapiens OX=9606 GN=PSMA3 PE=1 SV=2	PSMA3	1.071	0.310317
P25789	Proteasome subunit alpha type-4 OS=Homo sapiens OX=9606 GN=PSMA4 PE=1 SV=1	PSMA4	1.061	0.646561
P25815	Protein S100-P OS=Homo sapiens OX=9606 GN=S100P PE=1 SV=2	S100P	0.983	0.931062
P26006	Integrin alpha-3 OS=Homo sapiens OX=9606 GN=ITGA3 PE=1 SV=5	ITGA3	1.016	0.728232

P26196	Probable ATP-dependent RNA helicase DDX6 OS=Homo sapiens OX=9606 GN=DDX6 PE=1 SV=2	DDX6	1.076	0.228803
P26232	Catenin alpha-2 OS=Homo sapiens OX=9606 GN=CTNNA2 PE=1 SV=5	CTNNA2	0.728	0.000766
P26358	DNA (cytosine-5)-methyltransferase 1 OS=Homo sapiens OX=9606 GN=DNMT1	DNMT1	0.872	0.04659
P26368	Splicing factor U2AF 65 kDa subunit OS=Homo sapiens OX=9606 GN=U2AF2 PE=1	U2AF2	0.879	0.041682
P26373	60S ribosomal protein L13 OS=Homo sapiens OX=9606 GN=RPL13 PE=1 SV=4	RPL13	1.338	0.251373
P26440	Isovaleryl-CoA dehydrogenase, mitochondrial OS=Homo sapiens OX=9606 GN=IVD PE=1 SV=2	IVD	1.011	0.804966
P26572	Alpha-1,3-mannosyl-glycoprotein 2-beta-N-acetylglucosaminyltransferase OS=Homo sapiens OX=9606 GN=MGAT1 PE=1 SV=2	MGAT1	0.868	0.38415
P26583	High mobility group protein B2 OS=Homo sapiens OX=9606 GN=HMGB2 PE=1 SV=2	HMGB2	0.804	0.294466
P26599	Polypyrimidine tract-binding protein 1 OS=Homo sapiens OX=9606 GN=PTBP1 PE=1	PTBP1	1.027	0.654514
P26639	Threonine--tRNA ligase 1, cytoplasmic OS=Homo sapiens OX=9606 GN=TARS1 PE=1	TARS1	1.101	0.554789
P26640	Valine--tRNA ligase OS=Homo sapiens OX=9606 GN=VARS1 PE=1 SV=4	VARS1	1.049	0.539798
P26641	Elongation factor 1-gamma OS=Homo sapiens OX=9606 GN=EEF1G PE=1 SV=3	EEF1G	1.248	0.049853
P26885	Peptidyl-prolyl cis-trans isomerase FKBP2 OS=Homo sapiens OX=9606 GN=FKBP2 PE=1	FKBP2	1.039	0.497863
P27105	Stomatin OS=Homo sapiens OX=9606 GN=STOM PE=1 SV=3	STOM	0.984	0.757027
P27144	Adenylate kinase 4, mitochondrial OS=Homo sapiens OX=9606 GN=AK4 PE=1 SV=1	AK4	1.013	0.874084
P27348	14-3-3 protein theta OS=Homo sapiens OX=9606 GN=YWHAQ PE=1 SV=1	YWHAQ	1.119	0.342672
P27482	Calmodulin-like protein 3 OS=Homo sapiens OX=9606 GN=CALML3 PE=1 SV=2	CALML3	0.868	0.210792

P27635	60S ribosomal protein L10 OS=Homo sapiens OX=9606 GN=RPL10 PE=1 SV=4	RPL10	1.121	0.351027
P27694	Replication protein A 70 kDa DNA-binding subunit OS=Homo sapiens OX=9606 GN=RPA1	RPA1	0.859	0.16862
P27695	DNA-(apurinic or apyrimidinic site) endonuclease OS=Homo sapiens OX=9606 GN=APEX1	APEX1	0.774	0.021133
P27708	CAD protein OS=Homo sapiens OX=9606 GN=CAD PE=1 SV=3	CAD	1.266	0.083605
P27797	Calreticulin OS=Homo sapiens OX=9606 GN=CALR PE=1 SV=1	CALR	1.108	0.044792
P27816	Microtubule-associated protein 4 OS=Homo sapiens OX=9606 GN=MAP4 PE=1 SV=3	MAP4	1.082	0.312311
P27824	Calnexin OS=Homo sapiens OX=9606 GN=CANX PE=1 SV=2	CANX	1.1	0.062416
P28062	Proteasome subunit beta type-8 OS=Homo sapiens OX=9606 GN=PSMB8 PE=1 SV=3	PSMB8	0.948	0.359368
P28066	Proteasome subunit alpha type-5 OS=Homo sapiens OX=9606 GN=PSMA5 PE=1 SV=3	PSMA5	0.966	0.652196
P28070	Proteasome subunit beta type-4 OS=Homo sapiens OX=9606 GN=PSMB4 PE=1 SV=4	PSMB4	1.069	0.323964
P28072	Proteasome subunit beta type-6 OS=Homo sapiens OX=9606 GN=PSMB6 PE=1 SV=4	PSMB6	1.142	0.030321
P28074	Proteasome subunit beta type-5 OS=Homo sapiens OX=9606 GN=PSMB5 PE=1 SV=3	PSMB5	0.958	0.427868
P28288	ATP-binding cassette sub-family D member 3 OS=Homo sapiens OX=9606 GN=ABCD3 PE=1	ABCD3	1.034	0.550099
P28290	Protein ITPRID2 OS=Homo sapiens OX=9606 GN=ITPRID2 PE=1 SV=3	ITPRID2	1.145	0.172553
P28331	NADH-ubiquinone oxidoreductase 75 kDa subunit, mitochondrial OS=Homo sapiens OX=9606 GN=NDUFS1 PE=1 SV=3	NDUFS1	0.984	0.689624
P28340	DNA polymerase delta catalytic subunit OS=Homo sapiens OX=9606 GN=POLD1 PE=1	POLD1	0.918	0.408513
P28347	Transcriptional enhancer factor TEF-1 OS=Homo sapiens OX=9606 GN=TEAD1 PE=1	TEAD1	1.035	
P28482	Mitogen-activated protein kinase 1 OS=Homo sapiens OX=9606 GN=MAPK1 PE=1 SV=3	MAPK1	1.035	0.681189

P28715	DNA excision repair protein ERCC-5 OS=Homo sapiens OX=9606 GN=ERCC5 PE=1	ERCC5	0.759	
P28799	Progranulin OS=Homo sapiens OX=9606 GN=GRN PE=1 SV=2	GRN	1.202	0.012931
P28838	Cytosol aminopeptidase OS=Homo sapiens OX=9606 GN=LAP3 PE=1 SV=3	LAP3	0.945	0.47744
P29034	Protein S100-A2 OS=Homo sapiens OX=9606 GN=S100A2	S100A2	1.036	0.912962
P29083	General transcription factor IIE subunit 1 OS=Homo sapiens OX=9606 GN=GTF2E1 PE=1	GTF2E1	0.864	
P29084	Transcription initiation factor IIE subunit beta OS=Homo sapiens OX=9606 GN=GTF2E2 PE=1	GTF2E2	1.086	0.663702
P29144	Tripeptidyl-peptidase 2 OS=Homo sapiens OX=9606 GN=TPP2 PE=1 SV=4	TPP2	0.918	0.30399
P29279	CCN family member 2 OS=Homo sapiens OX=9606 GN=CCN2 PE=1 SV=2	CCN2	1.023	0.868843
P29317	Ephrin type-A receptor 2 OS=Homo sapiens OX=9606 GN=EPHA2 PE=1 SV=2	EPHA2	1.036	0.431796
P29323	Ephrin type-B receptor 2 OS=Homo sapiens OX=9606 GN=EPHB2 PE=1 SV=5	EPHB2	1.063	0.478714
P29373	Cellular retinoic acid-binding protein 2 OS=Homo sapiens OX=9606 GN=CRABP2 PE=1	CRABP2	1.134	0.248171
P29401	Transketolase OS=Homo sapiens OX=9606 GN=TKT PE=1 SV=3	TKT	0.877	0.324385
P29466	Caspase-1 OS=Homo sapiens OX=9606 GN=CASP1 PE=1	CASP1	1.182	0.386346
P29590	Protein PML OS=Homo sapiens OX=9606 GN=PML PE=1 SV=3	PML	0.948	0.201825
P29692	Elongation factor 1-delta OS=Homo sapiens OX=9606 GN=EEF1D PE=1 SV=5	EEF1D	1.012	0.946368
P29966	Myristoylated alanine-rich C-kinase substrate OS=Homo sapiens OX=9606 GN=MARCKS PE=1 SV=4	MARCKS	0.92	0.489036
P29992	Guanine nucleotide-binding protein subunit alpha-11 OS=Homo sapiens OX=9606	GNA11	1.089	0.450733
P30038	Delta-1-pyrroline-5-carboxylate dehydrogenase, mitochondrial OS=Homo sapiens OX=9606 GN=ALDH4A1 PE=1 SV=3	ALDH4A1	1.178	

P30040	Endoplasmic reticulum resident protein 29 OS=Homo sapiens OX=9606 GN=ERP29 PE=1 SV=4	ERP29	1.012	0.831548
P30041	Peroxiredoxin-6 OS=Homo sapiens OX=9606 GN=PRDX6	PRDX6	0.935	0.585431
P30043	Flavin reductase (NADPH) OS=Homo sapiens OX=9606 GN=BLVRB PE=1 SV=3	BLVRB	1.049	0.640715
P30044	Peroxiredoxin-5, mitochondrial OS=Homo sapiens OX=9606 GN=PRDX5 PE=1 SV=4	PRDX5	1.113	0.020302
P30046	D-dopachrome decarboxylase OS=Homo sapiens OX=9606 GN=DDT PE=1 SV=3	DDT	0.812	0.302741
P30048	Thioredoxin-dependent peroxide reductase, mitochondrial OS=Homo sapiens OX=9606 GN=PRDX3 PE=1 SV=3	PRDX3	0.916	0.456667
P30049	ATP synthase subunit delta, mitochondrial OS=Homo sapiens OX=9606 GN=ATP5F1D PE=1 SV=2	ATP5F1D	0.978	0.858133
P30050	60S ribosomal protein L12 OS=Homo sapiens OX=9606 GN=RPL12 PE=1 SV=1	RPL12	0.935	0.21066
P30084	Enoyl-CoA hydratase, mitochondrial OS=Homo sapiens OX=9606 GN=ECHS1 PE=1	ECHS1	1.03	0.713035
P30085	UMP-CMP kinase OS=Homo sapiens OX=9606 GN=CMPK1 PE=1 SV=3	CMPK1	0.968	0.755706
P30101	Protein disulfide-isomerase A3 OS=Homo sapiens OX=9606 GN=PDIA3 PE=1 SV=4	PDIA3	0.983	0.66883
P30153	Serine/threonine-protein phosphatase 2A 65 kDa regulatory subunit A alpha isoform OS=Homo sapiens OX=9606 GN=PPP2R1A PE=1 SV=4	PPP2R1A	1.069	0.363993
P30260	Cell division cycle protein 27 homolog OS=Homo sapiens OX=9606 GN=CDC27 PE=1	CDC27	0.914	0.466942
P30405	Peptidyl-prolyl cis-trans isomerase F, mitochondrial OS=Homo sapiens OX=9606 GN=PPIF PE=1	PPIF	1.005	0.954504
P30419	Glycylpeptide N-tetradecanoyltransferase 1 OS=Homo sapiens OX=9606 GN=NMT1 PE=1 SV=2	NMT1	0.936	0.514506
P30519	Heme oxygenase 2 OS=Homo sapiens OX=9606 GN=HMOX2 PE=1 SV=2	HMOX2	1.108	0.013708

P30520	Adenylosuccinate synthetase isozyme 2 OS=Homo sapiens OX=9606 GN=ADSS2 PE=1	ADSS2	0.979	0.789818
P30533	Alpha-2-macroglobulin receptor-associated protein OS=Homo sapiens OX=9606 GN=LRPAP1 PE=1 SV=1	LRPAP1	1.055	0.591078
P30536	Translocator protein OS=Homo sapiens OX=9606 GN=TSPO PE=1 SV=3	TSPO	1.117	0.33279
P30566	Adenylosuccinate lyase OS=Homo sapiens OX=9606 GN=ADSL PE=1 SV=2	ADSL	0.991	0.865469
P30622	CAP-Gly domain-containing linker protein 1 OS=Homo sapiens OX=9606 GN=CLIP1 PE=1 SV=2	CLIP1	1.078	0.447608
P30626	Sorcin OS=Homo sapiens OX=9606 GN=SRI PE=1 SV=1	SRI	1.013	0.940138
P30825	High affinity cationic amino acid transporter 1 OS=Homo sapiens OX=9606 GN=SLC7A1 PE=1	SLC7A1	0.96	0.536633
P30837	Aldehyde dehydrogenase X, mitochondrial OS=Homo sapiens OX=9606 GN=ALDH1B1 PE=1 SV=3	ALDH1B1	0.987	0.796283
P30838	Aldehyde dehydrogenase, dimeric NADP-preferring OS=Homo sapiens OX=9606 GN=ALDH3A1 PE=1 SV=3	ALDH3A1	1.106	0.074541
P30876	DNA-directed RNA polymerase II subunit RPB2 OS=Homo sapiens OX=9606 GN=POLR2B PE=1 SV=1	POLR2B	0.972	0.568007
P31040	Succinate dehydrogenase [ubiquinone] flavoprotein subunit, mitochondrial OS=Homo sapiens OX=9606 GN=SDHA PE=1 SV=2	SDHA	0.922	0.142435
P31150	Rab GDP dissociation inhibitor alpha OS=Homo sapiens OX=9606 GN=GDI1 PE=1 SV=2	GDI1	1.022	0.93835
P31153	S-adenosylmethionine synthase isoform type-2 OS=Homo sapiens OX=9606 GN=MAT2A PE=1	MAT2A	0.879	0.326132
P31350	Ribonucleoside-diphosphate reductase subunit M2 OS=Homo sapiens OX=9606 GN=RRM2 PE=1 SV=1	RRM2	1.282	0.092653
P31431	Syndecan-4 OS=Homo sapiens OX=9606 GN=SDC4 PE=1 SV=2	SDC4	1.003	0.998258
P31483	Nucleolysin TIA-1 isoform p40 OS=Homo sapiens OX=9606 GN=TIA1 PE=1 SV=3	TIA1	0.887	0.304785

P31641	Sodium- and chloride-dependent taurine transporter OS=Homo sapiens OX=9606 GN=SLC6A6 PE=1 SV=2	SLC6A6	0.747	0.251937
P31689	DnaJ homolog subfamily A member 1 OS=Homo sapiens OX=9606 GN=DNAJA1 PE=1	DNAJA1	1.017	0.754818
P31930	Cytochrome b-c1 complex subunit 1, mitochondrial OS=Homo sapiens OX=9606 GN=UQCRC1 PE=1 SV=3	UQCRC1	1.025	0.526662
P31937	3-hydroxyisobutyrate dehydrogenase, mitochondrial OS=Homo sapiens OX=9606 GN=HIBADH PE=1 SV=2	HIBADH	0.977	0.577401
P31939	Bifunctional purine biosynthesis protein ATIC OS=Homo sapiens OX=9606 GN=ATIC PE=1 SV=3	ATIC	1.072	0.68253
P31942	Heterogeneous nuclear ribonucleoprotein H3 OS=Homo sapiens OX=9606 GN=HNRNPH3 PE=1 SV=2	HNRNPH3	0.927	0.136069
P31943	Heterogeneous nuclear ribonucleoprotein H OS=Homo sapiens OX=9606 GN=HNRNPH1 PE=1 SV=4	HNRNPH1	0.868	0.095138
P31946	14-3-3 protein beta/alpha OS=Homo sapiens OX=9606 GN=YWHAB PE=1 SV=3	YWHAB	1.023	0.873667
P31947	14-3-3 protein sigma OS=Homo sapiens OX=9606 GN=SFN PE=1 SV=1	SFN	1.155	0.13122
P31948	Stress-induced-phosphoprotein 1 OS=Homo sapiens OX=9606 GN=STIP1 PE=1 SV=1	STIP1	1.021	0.832333
P31949	Protein S100-A11 OS=Homo sapiens OX=9606 GN=S100A11 PE=1 SV=2	S100A11	0.927	0.488969
P32119	Peroxiredoxin-2 OS=Homo sapiens OX=9606 GN=PRDX2	PRDX2	1.016	0.930006
P32321	Deoxycytidylate deaminase OS=Homo sapiens OX=9606 GN=DCTD PE=1 SV=2	DCTD	0.808	0.33418
P32322	Pyrroline-5-carboxylate reductase 1, mitochondrial OS=Homo sapiens OX=9606 GN=PYCR1	PYCR1	0.958	0.751201
P32519	ETS-related transcription factor Elf-1 OS=Homo sapiens OX=9606 GN=ELF1 PE=1 SV=2	ELF1	0.801	
P32780	General transcription factor IIH subunit 1 OS=Homo sapiens OX=9606 GN=GTF2H1 PE=1 SV=1	GTF2H1	1.004	0.974573

P32969	60S ribosomal protein L9 OS=Homo sapiens OX=9606 GN=RPL9 PE=1 SV=1	RPL9	0.939	0.658934
P33121	Long-chain-fatty-acid--CoA ligase 1 OS=Homo sapiens OX=9606 GN=ACSL1 PE=1 SV=1	ACSL1	0.986	0.820296
P33176	Kinesin-1 heavy chain OS=Homo sapiens OX=9606 GN=KIF5B PE=1 SV=1	KIF5B	1.119	0.218027
P33240	Cleavage stimulation factor subunit 2 OS=Homo sapiens OX=9606 GN=CSTF2 PE=1	CSTF2	0.91	0.204707
P33316	Deoxyuridine 5'-triphosphate nucleotidohydrolase, mitochondrial OS=Homo sapiens OX=9606 GN=DUT PE=1 SV=4	DUT	0.885	0.324683
P33527	Multidrug resistance-associated protein 1 OS=Homo sapiens OX=9606 GN=ABCC1 PE=1	ABCC1	0.989	0.795335
P33897	ATP-binding cassette sub-family D member 1 OS=Homo sapiens OX=9606 GN=ABCD1 PE=1	ABCD1	1.142	0.529558
P33947	ER lumen protein-retaining receptor 2 OS=Homo sapiens OX=9606 GN=KDELR2 PE=1	KDELR2	1.002	0.947633
P33991	DNA replication licensing factor MCM4 OS=Homo sapiens OX=9606 GN=MCM4 PE=1	MCM4	0.859	0.109925
P33992	DNA replication licensing factor MCM5 OS=Homo sapiens OX=9606 GN=MCM5 PE=1	MCM5	0.809	0.075983
P33993	DNA replication licensing factor MCM7 OS=Homo sapiens OX=9606 GN=MCM7 PE=1	MCM7	0.824	0.079081
P34897	Serine hydroxymethyltransferase, mitochondrial OS=Homo sapiens OX=9606 GN=SHMT2 PE=1	SHMT2	1.028	0.689897
P34932	Heat shock 70 kDa protein 4 OS=Homo sapiens OX=9606 GN=HSPA4 PE=1 SV=4	HSPA4	0.98	0.836568
P35052	Glypican-1 OS=Homo sapiens OX=9606 GN=GPC1 PE=1 SV=2	GPC1	0.867	0.04031
P35221	Catenin alpha-1 OS=Homo sapiens OX=9606 GN=CTNNA1 PE=1 SV=1	CTNNA1	0.912	0.025619
P35222	Catenin beta-1 OS=Homo sapiens OX=9606 GN=CTNNB1 PE=1 SV=1	CTNNB1	1.031	0.671106
P35226	Polycomb complex protein BMI-1 OS=Homo sapiens OX=9606 GN=BMI1 PE=1 SV=2	BMI1	0.907	0.18847
P35232	Prohibitin OS=Homo sapiens OX=9606 GN=PHB PE=1 SV=1	PHB	1.031	0.411925

P35237	Serpin B6 OS=Homo sapiens OX=9606 GN=SERPINB6 PE=1 SV=3	SERPINB6	0.945	0.487608
P35241	Radixin OS=Homo sapiens OX=9606 GN=RDX PE=1 SV=1	RDX	1.096	0.539265
P35244	Replication protein A 14 kDa subunit OS=Homo sapiens OX=9606 GN=RPA3 PE=1 SV=1	RPA3	0.848	0.160709
P35249	Replication factor C subunit 4 OS=Homo sapiens OX=9606 GN=RFC4 PE=1 SV=2	RFC4	0.901	0.261347
P35250	Replication factor C subunit 2 OS=Homo sapiens OX=9606 GN=RFC2 PE=1 SV=3	RFC2	1.055	0.059072
P35268	60S ribosomal protein L22 OS=Homo sapiens OX=9606 GN=RPL22 PE=1 SV=2	RPL22	1.086	0.118034
P35269	General transcription factor IIF subunit 1 OS=Homo sapiens OX=9606 GN=GTF2F1 PE=1	GTF2F1	0.798	0.122864
P35270	Sepiapterin reductase OS=Homo sapiens OX=9606 GN=SPR PE=1 SV=1	SPR	1.123	0.225269
P35579	Myosin-9 OS=Homo sapiens OX=9606 GN=MYH9 PE=1 SV=4	MYH9	1.248	0.069857
P35580	Myosin-10 OS=Homo sapiens OX=9606 GN=MYH10 PE=1	MYH10	1.127	0.103265
P35606	Coatomer subunit beta' OS=Homo sapiens OX=9606 GN=COPB2 PE=1 SV=2	COPB2	1.008	0.903027
P35611	Alpha-adducin OS=Homo sapiens OX=9606 GN=ADD1 PE=1 SV=2	ADD1	0.857	0.096744
P35613	Basigin OS=Homo sapiens OX=9606 GN=BSG PE=1 SV=2	BSG	0.998	0.960187
P35625	Metalloproteinase inhibitor 3 OS=Homo sapiens OX=9606 GN=TIMP3 PE=1 SV=2	TIMP3	1.13	0.651587
P35637	RNA-binding protein FUS OS=Homo sapiens OX=9606 GN=FUS PE=1 SV=1	FUS	0.799	0.158149
P35658	Nuclear pore complex protein Nup214 OS=Homo sapiens OX=9606 GN=NUP214 PE=1	NUP214	0.919	0.154484
P35659	Protein DEK OS=Homo sapiens OX=9606 GN=DEK PE=1 SV=1	DEK	0.774	0.042164
P35914	Hydroxymethylglutaryl-CoA lyase, mitochondrial OS=Homo sapiens OX=9606 GN=HMGCL PE=1 SV=2	HMGCL	0.861	0.241778
P35998	26S proteasome regulatory subunit 7 OS=Homo sapiens OX=9606 GN=PSMC2 PE=1 SV=3	PSMC2	1.01	0.92589

P36542	ATP synthase subunit gamma, mitochondrial OS=Homo sapiens OX=9606 GN=ATP5F1C PE=1 SV=1	ATP5F1C	1.11	0.039707
P36543	V-type proton ATPase subunit E 1 OS=Homo sapiens OX=9606 GN=ATP6V1E1 PE=1 SV=1	ATP6V1E1	1.072	0.483574
P36551	Oxygen-dependent coproporphyrinogen-III oxidase, mitochondrial OS=Homo sapiens OX=9606 GN=CPOX PE=1 SV=3	CPOX	1.032	0.525088
P36578	60S ribosomal protein L4 OS=Homo sapiens OX=9606 GN=RPL4 PE=1 SV=5	RPL4	1.459	0.242139
P36776	Lon protease homolog, mitochondrial OS=Homo sapiens OX=9606 GN=LONP1 PE=1	LONP1	0.994	0.892562
P36871	Phosphoglucomutase-1 OS=Homo sapiens OX=9606 GN=PGM1 PE=1 SV=3	PGM1	0.929	0.544794
P36873	Serine/threonine-protein phosphatase PP1-gamma catalytic subunit OS=Homo sapiens OX=9606 GN=PPP1CC PE=1 SV=1	PPP1CC	0.942	0.562143
P36941	Tumor necrosis factor receptor superfamily member 3 OS=Homo sapiens OX=9606 GN=LTBR PE=1 SV=1	LTBR	1.213	0.283393
P36952	Serpin B5 OS=Homo sapiens OX=9606 GN=SERPINB5 PE=1 SV=2	SERPINB5	1.063	0.691103
P36954	DNA-directed RNA polymerase II subunit RPB9 OS=Homo sapiens OX=9606 GN=POLR2I PE=1	POLR2I	0.875	0.486135
P36957	Dihydrolipoyllysine-residue succinyltransferase component of 2-oxoglutarate dehydrogenase complex, mitochondrial OS=Homo sapiens OX=9606 GN=DLST PE=1 SV=4	DLST	1.021	0.778946
P37108	Signal recognition particle 14 kDa protein OS=Homo sapiens OX=9606 GN=SRP14 PE=1 SV=2	SRP14	0.855	0.125309
P37173	TGF-beta receptor type-2 OS=Homo sapiens OX=9606 GN=TGFBR2 PE=1 SV=2	TGFBR2	0.967	0.726232
P37198	Nuclear pore glycoprotein p62 OS=Homo sapiens OX=9606 GN=NUP62 PE=1 SV=3	NUP62	0.927	0.332469
P37235	Hippocalcin-like protein 1 OS=Homo sapiens OX=9606 GN=HPCAL1 PE=1 SV=3	HPCAL1	0.922	0.611862

P37268	Squalene synthase OS=Homo sapiens OX=9606 GN=FDFT1 PE=1 SV=1	FDFT1	1.008	0.950585
P37802	Transgelin-2 OS=Homo sapiens OX=9606 GN=TAGLN2 PE=1 SV=3	TAGLN2	1.142	0.273896
P37837	Transaldolase OS=Homo sapiens OX=9606 GN=TALDO1 PE=1 SV=2	TALDO1	0.857	0.187755
P37840	Alpha-synuclein OS=Homo sapiens OX=9606 GN=SNCA	SNCA	1.023	0.595989
P38117	Electron transfer flavoprotein subunit beta OS=Homo sapiens OX=9606 GN=ETFB PE=1 SV=3	ETFB	0.883	0.144369
P38159	RNA-binding motif protein, X chromosome OS=Homo sapiens OX=9606 GN=RBMX PE=1	RBMX	1.015	0.701658
P38432	Coilin OS=Homo sapiens OX=9606 GN=COIL PE=1 SV=1	COIL	0.891	0.376744
P38435	Vitamin K-dependent gamma-carboxylase OS=Homo sapiens OX=9606 GN=GGCX PE=1 SV=2	GGCX	1.044	0.814531
P38571	Lysosomal acid lipase/cholesteryl ester hydrolase OS=Homo sapiens OX=9606 GN=LIPA PE=1 SV=2	LIPA	0.94	0.673031
P38606	V-type proton ATPase catalytic subunit A OS=Homo sapiens OX=9606 GN=ATP6V1A PE=1 SV=2	ATP6V1A	1.081	0.088687
P38646	Stress-70 protein, mitochondrial OS=Homo sapiens OX=9606 GN=HSPA9 PE=1 SV=2	HSPA9	1.001	0.942402
P38919	Eukaryotic initiation factor 4A-III OS=Homo sapiens OX=9606 GN=EIF4A3 PE=1 SV=4	EIF4A3	1.065	0.053454
P39019	40S ribosomal protein S19 OS=Homo sapiens OX=9606 GN=RPS19 PE=1 SV=2	RPS19	0.962	0.633097
P39023	60S ribosomal protein L3 OS=Homo sapiens OX=9606 GN=RPL3 PE=1 SV=2	RPL3	1.198	0.193026
P39060	Collagen alpha-1(XVIII) chain OS=Homo sapiens OX=9606 GN=COL18A1 PE=1 SV=5	COL18A1	0.82	0.340935
P39656	Dolichyl-diphosphooligosaccharide--protein glycosyltransferase 48 kDa subunit OS=Homo sapiens OX=9606	DDOST	1.053	0.426002
P39687	Acidic leucine-rich nuclear phosphoprotein 32 family member A OS=Homo sapiens OX=9606 GN=ANP32A PE=1 SV=1	ANP32A	0.789	0.196163

P39748	Flap endonuclease 1 OS=Homo sapiens OX=9606 GN=FEN1 PE=1 SV=1	FEN1	0.821	0.304904
P39880	Homeobox protein cut-like 1 OS=Homo sapiens OX=9606 GN=CUX1 PE=1 SV=3	CUX1	0.932	0.120486
P40121	Macrophage-capping protein OS=Homo sapiens OX=9606 GN=CAPG PE=1 SV=2	CAPG	1.171	0.065106
P40189	Interleukin-6 receptor subunit beta OS=Homo sapiens OX=9606 GN=IL6ST PE=1 SV=2	IL6ST	1.23	0.206714
P40222	Alpha-taxilin OS=Homo sapiens OX=9606 GN=TXLNA PE=1	TXLNA	0.971	0.742379
P40227	T-complex protein 1 subunit zeta OS=Homo sapiens OX=9606 GN=CCT6A PE=1 SV=3	CCT6A	0.927	0.521144
P40429	60S ribosomal protein L13a OS=Homo sapiens OX=9606 GN=RPL13A PE=1 SV=2	RPL13A	1.234	0.276866
P40616	ADP-ribosylation factor-like protein 1 OS=Homo sapiens OX=9606 GN=ARL1 PE=1 SV=1	ARL1	0.829	0.051692
P40692	DNA mismatch repair protein Mlh1 OS=Homo sapiens OX=9606 GN=MLH1 PE=1 SV=1	MLH1	0.816	0.08698
P40763	Signal transducer and activator of transcription 3 OS=Homo sapiens OX=9606 GN=STAT3 PE=1	STAT3	1.124	0.849566
P40925	Malate dehydrogenase, cytoplasmic OS=Homo sapiens OX=9606 GN=MDH1 PE=1 SV=4	MDH1	0.897	0.264995
P40926	Malate dehydrogenase, mitochondrial OS=Homo sapiens OX=9606 GN=MDH2 PE=1 SV=3	MDH2	1.042	0.574223
P40937	Replication factor C subunit 5 OS=Homo sapiens OX=9606 GN=RFC5 PE=1 SV=1	RFC5	0.96	0.5945
P40938	Replication factor C subunit 3 OS=Homo sapiens OX=9606 GN=RFC3 PE=1 SV=2	RFC3	0.849	0.104503
P40939	Trifunctional enzyme subunit alpha, mitochondrial OS=Homo sapiens OX=9606 GN=HADHA PE=1 SV=2	HADHA	1.021	0.662135
P41091	Eukaryotic translation initiation factor 2 subunit 3 OS=Homo sapiens OX=9606 GN=EIF2S3	EIF2S3	1.03	0.762543
P41134	DNA-binding protein inhibitor ID-1 OS=Homo sapiens OX=9606 GN=ID1 PE=1 SV=3	ID1	1.013	0.927277
P41208	Centrin-2 OS=Homo sapiens OX=9606 GN=CETN2 PE=1	CETN2	0.861	0.350459

P41212	Transcription factor ETV6 OS=Homo sapiens OX=9606 GN=ETV6 PE=1 SV=1	ETV6	1.053	0.455452
P41223	Protein BUD31 homolog OS=Homo sapiens OX=9606 GN=BUD31 PE=1 SV=2	BUD31	0.858	0.371336
P41227	N-alpha-acetyltransferase 10 OS=Homo sapiens OX=9606 GN=NAA10 PE=1 SV=1	NAA10	0.863	0.699243
P41250	Glycine--tRNA ligase OS=Homo sapiens OX=9606 GN=GARS1 PE=1 SV=3	GARS1	1.083	0.161584
P41252	Isoleucine--tRNA ligase, cytoplasmic OS=Homo sapiens OX=9606 GN=IARS1 PE=1 SV=2	IARS1	0.972	0.707231
P41567	Eukaryotic translation initiation factor 1 OS=Homo sapiens OX=9606 GN=EIF1 PE=1 SV=1	EIF1	0.916	0.41064
P41743	Protein kinase C iota type OS=Homo sapiens OX=9606 GN=PRKCI PE=1 SV=2	PRKCI	1.12	0.483746
P42126	Enoyl-CoA delta isomerase 1, mitochondrial OS=Homo sapiens OX=9606 GN=ECI1 PE=1 SV=1	ECI1	0.989	0.810646
P42166	Lamina-associated polypeptide 2, isoform alpha OS=Homo sapiens OX=9606 GN=TMPO PE=1 SV=2	TMPO	0.888	0.001648
P42167	Lamina-associated polypeptide 2, isoforms beta/gamma OS=Homo sapiens OX=9606 GN=TMPO PE=1 SV=2	TMPO	0.968	0.633793
P42224	Signal transducer and activator of transcription 1-alpha/beta OS=Homo sapiens OX=9606 GN=STAT1 PE=1 SV=2	STAT1	1.131	0.282275
P42285	Exosome RNA helicase MTR4 OS=Homo sapiens OX=9606 GN=MTREX PE=1 SV=3	MTREX	0.935	0.126617
P42330	Aldo-keto reductase family 1 member C3 OS=Homo sapiens OX=9606 GN=AKR1C3 PE=1 SV=4	AKR1C3	1.014	0.990913
P42356	Phosphatidylinositol 4-kinase alpha OS=Homo sapiens OX=9606 GN=PI4KA PE=1 SV=4	PI4KA	1.022	0.928659
P42574	Caspase-3 OS=Homo sapiens OX=9606 GN=CASP3 PE=1	CASP3	1.011	0.985564
P42677	40S ribosomal protein S27 OS=Homo sapiens OX=9606 GN=RPS27 PE=1 SV=3	RPS27	1.019	0.620449
P42695	Condensin-2 complex subunit D3 OS=Homo sapiens OX=9606 GN=NCAPD3 PE=1 SV=2	NCAPD3	0.969	0.706983

P42696	RNA-binding protein 34 OS=Homo sapiens OX=9606 GN=RBM34 PE=1 SV=2	RBM34	1.322	0.732275
P42704	Leucine-rich PPR motif-containing protein, mitochondrial OS=Homo sapiens OX=9606 GN=LRPPRC PE=1 SV=3	LRPPRC	0.967	0.312976
P42765	3-ketoacyl-CoA thiolase, mitochondrial OS=Homo sapiens OX=9606 GN=ACAA2 PE=1	ACAA2	0.948	0.589757
P42785	Lysosomal Pro-X carboxypeptidase OS=Homo sapiens OX=9606 GN=PRCP	PRCP	1.074	0.482685
P42892	Endothelin-converting enzyme 1 OS=Homo sapiens OX=9606 GN=ECE1 PE=1 SV=2	ECE1	1.035	0.607441
P43003	Excitatory amino acid transporter 1 OS=Homo sapiens OX=9606 GN=SLC1A3 PE=1 SV=1	SLC1A3	0.901	0.343894
P43034	Platelet-activating factor acetylhydrolase IB subunit beta OS=Homo sapiens OX=9606 GN=PAFAH1B1 PE=1 SV=2	PAFAH1B 1	1.078	0.32102
P43121	Cell surface glycoprotein MUC18 OS=Homo sapiens OX=9606 GN=MCAM PE=1 SV=2	MCAM	1.053	0.302633
P43243	Matrin-3 OS=Homo sapiens OX=9606 GN=MATR3 PE=1	MATR3	0.989	0.789865
P43246	DNA mismatch repair protein Msh2 OS=Homo sapiens OX=9606 GN=MSH2 PE=1 SV=1	MSH2	0.85	0.213191
P43304	Glycerol-3-phosphate dehydrogenase, mitochondrial OS=Homo sapiens OX=9606 GN=GPD2 PE=1 SV=3	GPD2	1.057	0.083832
P43307	Translocon-associated protein subunit alpha OS=Homo sapiens OX=9606 GN=SSR1 PE=1 SV=3	SSR1	1.08	0.45717
P43358	Melanoma-associated antigen 4 OS=Homo sapiens OX=9606 GN=MAGEA4 PE=1 SV=2	MAGEA4	1.017	0.881064
P43363	Melanoma-associated antigen 10 OS=Homo sapiens OX=9606 GN=MAGEA10 PE=2 SV=2	MAGEA10	0.882	0.386703
P43378	Tyrosine-protein phosphatase non-receptor type 9 OS=Homo sapiens OX=9606 GN=PTPN9 PE=1	PTPN9	0.961	0.772703
P43487	Ran-specific GTPase-activating protein OS=Homo sapiens OX=9606 GN=RANBP1 PE=1	RANBP1	1.026	0.948683

P43490	Nicotinamide phosphoribosyltransferase OS=Homo sapiens OX=9606 GN=NAMPT PE=1 SV=1	NAMPT	0.974	0.747216
P43686	26S proteasome regulatory subunit 6B OS=Homo sapiens OX=9606 GN=PSMC4 PE=1 SV=2	PSMC4	1.117	0.11563
P43897	Elongation factor Ts, mitochondrial OS=Homo sapiens OX=9606 GN=TSFM PE=1 SV=2	TSFM	0.9	0.216484
P45877	Peptidyl-prolyl cis-trans isomerase C OS=Homo sapiens OX=9606 GN=PPIC PE=1 SV=1	PPIC	1.037	0.488326
P45880	Voltage-dependent anion-selective channel protein 2 OS=Homo sapiens OX=9606 GN=VDAC2	VDAC2	1.069	0.2327
P45973	Chromobox protein homolog 5 OS=Homo sapiens OX=9606 GN=CBX5 PE=1 SV=1	CBX5	0.934	0.448886
P45974	Ubiquitin carboxyl-terminal hydrolase 5 OS=Homo sapiens OX=9606 GN=USP5 PE=1 SV=2	USP5	1.038	0.762698
P46013	Proliferation marker protein Ki-67 OS=Homo sapiens OX=9606 GN=MKI67 PE=1 SV=2	MKI67	1.058	0.493565
P46060	Ran GTPase-activating protein 1 OS=Homo sapiens OX=9606 GN=RANGAP1 PE=1 SV=1	RANGAP1	1.024	0.737457
P46063	ATP-dependent DNA helicase Q1 OS=Homo sapiens OX=9606 GN=RECQL PE=1 SV=3	RECQL	0.896	0.329056
P46087	Probable 28S rRNA (cytosine(4447)-C(5))-methyltransferase OS=Homo sapiens OX=9606 GN=NOP2	NOP2	1.266	0.216432
P46100	Transcriptional regulator ATRX OS=Homo sapiens OX=9606 GN=ATRX PE=1 SV=5	ATRX	0.848	0.093046
P46108	Adapter molecule crk OS=Homo sapiens OX=9606 GN=CRK PE=1 SV=2	CRK	1.134	0.460348
P46109	Crk-like protein OS=Homo sapiens OX=9606 GN=CRKL	CRKL	1.045	0.64141
P46199	Translation initiation factor IF-2, mitochondrial OS=Homo sapiens OX=9606 GN=MTIF2 PE=1 SV=2	MTIF2	0.98	0.715771
P46379	Large proline-rich protein BAG6 OS=Homo sapiens OX=9606 GN=BAG6 PE=1 SV=2	BAG6	1.084	0.419428
P46459	Vesicle-fusing ATPase OS=Homo sapiens OX=9606 GN=NSF PE=1 SV=3	NSF	1.238	0.002682

P46734	Dual specificity mitogen-activated protein kinase kinase 3 OS=Homo sapiens OX=9606 GN=MAP2K3 PE=1 SV=2	MAP2K3	0.896	0.480194
P46776	60S ribosomal protein L27a OS=Homo sapiens OX=9606 GN=RPL27A PE=1 SV=2	RPL27A	0.937	0.632475
P46777	60S ribosomal protein L5 OS=Homo sapiens OX=9606 GN=RPL5 PE=1 SV=3	RPL5	0.991	0.979536
P46778	60S ribosomal protein L21 OS=Homo sapiens OX=9606 GN=RPL21 PE=1 SV=2	RPL21	1.25	0.232556
P46779	60S ribosomal protein L28 OS=Homo sapiens OX=9606 GN=RPL28 PE=1 SV=3	RPL28	1.219	0.27893
P46781	40S ribosomal protein S9 OS=Homo sapiens OX=9606 GN=RPS9 PE=1 SV=3	RPS9	1.16	0.334575
P46782	40S ribosomal protein S5 OS=Homo sapiens OX=9606 GN=RPS5 PE=1 SV=4	RPS5	0.903	0.481757
P46783	40S ribosomal protein S10 OS=Homo sapiens OX=9606 GN=RPS10 PE=1 SV=1	RPS10	0.904	0.535053
P46926	Glucosamine-6-phosphate isomerase 1 OS=Homo sapiens OX=9606 GN=GNPDA1 PE=1	GNPDA1	1.102	0.047324
P46937	Transcriptional coactivator YAP1 OS=Homo sapiens OX=9606 GN=YAP1 PE=1 SV=2	YAP1	1.026	0.930198
P46939	Utrophin OS=Homo sapiens OX=9606 GN=UTRN PE=1 SV=2	UTRN	1.03	0.758988
P46940	Ras GTPase-activating-like protein IQGAP1 OS=Homo sapiens OX=9606 GN=IQGAP1 PE=1 SV=1	IQGAP1	1.082	0.309038
P46977	Dolichyl-diphosphooligosaccharide--protein glycosyltransferase subunit STT3A OS=Homo sapiens OX=9606	STT3A	1.08	0.539316
P47755	F-actin-capping protein subunit alpha-2 OS=Homo sapiens OX=9606 GN=CAPZA2 PE=1 SV=3	CAPZA2	0.965	0.546904
P47756	F-actin-capping protein subunit beta OS=Homo sapiens OX=9606 GN=CAPZB PE=1 SV=4	CAPZB	1.067	0.182367
P47813	Eukaryotic translation initiation factor 1A, X-chromosomal OS=Homo sapiens OX=9606 GN=EIF1AX PE=1 SV=2	EIF1AX	1.187	0.212591

P47895	Aldehyde dehydrogenase family 1 member A3 OS=Homo sapiens OX=9606 GN=ALDH1A3 PE=1 SV=2	ALDH1A3	1.032	0.79371
P47897	Glutamine--tRNA ligase OS=Homo sapiens OX=9606 GN=QARS1 PE=1 SV=1	QARS1	0.956	0.602114
P47914	60S ribosomal protein L29 OS=Homo sapiens OX=9606 GN=RPL29 PE=1 SV=2	RPL29	1.136	0.477594
P47985	Cytochrome b-c1 complex subunit Rieske, mitochondrial OS=Homo sapiens OX=9606 GN=UQCRFS1 PE=1 SV=2	UQCRFS1	0.924	0.117481
P48047	ATP synthase subunit O, mitochondrial OS=Homo sapiens OX=9606 GN=ATP5PO PE=1 SV=1	ATP5PO	0.975	0.788759
P48059	LIM and senescent cell antigen-like-containing domain protein 1 OS=Homo sapiens OX=9606 GN=LIMS1 PE=1 SV=4	LIMS1	0.989	0.896761
P48060	Glioma pathogenesis-related protein 1 OS=Homo sapiens OX=9606 GN=GLIPR1 PE=1	GLIPR1	1.104	0.25763
P48147	Prolyl endopeptidase OS=Homo sapiens OX=9606 GN=PREP PE=1 SV=2	PREP	0.819	0.142021
P48163	NADP-dependent malic enzyme OS=Homo sapiens OX=9606 GN=ME1 PE=1 SV=1	ME1	1.064	0.536786
P48436	Transcription factor SOX-9 OS=Homo sapiens OX=9606 GN=SOX9 PE=1 SV=1	SOX9	1.292	
P48444	Coatomer subunit delta OS=Homo sapiens OX=9606 GN=ARCN1 PE=1 SV=1	ARCN1	1.055	0.570839
P48449	Lanosterol synthase OS=Homo sapiens OX=9606 GN=LSS PE=1 SV=1	LSS	1.063	0.369808
P48507	Glutamate--cysteine ligase regulatory subunit OS=Homo sapiens OX=9606 GN=GCLM PE=1 SV=1	GCLM	0.869	0.231586
P48509	CD151 antigen OS=Homo sapiens OX=9606 GN=CD151 PE=1	CD151	0.989	0.974303
P48556	26S proteasome non-ATPase regulatory subunit 8 OS=Homo sapiens OX=9606 GN=PSMD8 PE=1 SV=2	PSMD8	1.019	0.740572
P48634	Protein PRRC2A OS=Homo sapiens OX=9606 GN=PRRC2A PE=1 SV=3	PRRC2A	1.008	0.857149

P48643	T-complex protein 1 subunit epsilon OS=Homo sapiens OX=9606 GN=CCT5 PE=1 SV=1	CCT5	0.91	0.390483
P48651	Phosphatidylserine synthase 1 OS=Homo sapiens OX=9606 GN=PTDSS1 PE=1 SV=1	PTDSS1	0.975	0.866251
P48723	Heat shock 70 kDa protein 13 OS=Homo sapiens OX=9606 GN=HSPA13 PE=1 SV=1	HSPA13	1.091	0.208996
P48729	Casein kinase I isoform alpha OS=Homo sapiens OX=9606 GN=CSNK1A1 PE=1 SV=2	CSNK1A1	0.944	0.5906
P48730	Casein kinase I isoform delta OS=Homo sapiens OX=9606 GN=CSNK1D PE=1 SV=2	CSNK1D	1.201	0.136183
P48735	Isocitrate dehydrogenase [NADP], mitochondrial OS=Homo sapiens OX=9606 GN=IDH2 PE=1 SV=2	IDH2	1.019	0.726135
P48739	Phosphatidylinositol transfer protein beta isoform OS=Homo sapiens OX=9606 GN=PITPNB	PITPNB	1.118	0.174031
P48960	Adhesion G protein-coupled receptor E5 OS=Homo sapiens OX=9606 GN=ADGRE5 PE=1 SV=4	ADGRE5	1.064	0.477144
P49005	DNA polymerase delta subunit 2 OS=Homo sapiens OX=9606 GN=POLD2 PE=1 SV=1	POLD2	0.852	0.174835
P49006	MARCKS-related protein OS=Homo sapiens OX=9606 GN=MARCKSL1 PE=1 SV=2	MARCKSL1	1.022	0.859843
P49023	Paxillin OS=Homo sapiens OX=9606 GN=PXN PE=1 SV=3	PXN	1.587	0.027711
P49184	Deoxyribonuclease-1-like 1 OS=Homo sapiens OX=9606 GN=DNASE1L1 PE=1 SV=1	DNASE1L1	1.101	0.502928
P49189	4-trimethylaminobutyraldehyde dehydrogenase OS=Homo sapiens OX=9606 GN=ALDH9A1 PE=1 SV=3	ALDH9A1	0.964	0.752909
P49207	60S ribosomal protein L34 OS=Homo sapiens OX=9606 GN=RPL34 PE=1 SV=3	RPL34	1.627	0.105447
P49257	Protein ERGIC-53 OS=Homo sapiens OX=9606 GN=LMAN1 PE=1 SV=2	LMAN1	0.969	0.574692
P49321	Nuclear autoantigenic sperm protein OS=Homo sapiens OX=9606 GN=NASP PE=1 SV=2	NASP	0.862	0.282683
P49327	Fatty acid synthase OS=Homo sapiens OX=9606 GN=FASN PE=1 SV=3	FASN	1.099	0.419862

P49368	T-complex protein 1 subunit gamma OS=Homo sapiens OX=9606 GN=CCT3 PE=1 SV=4	CCT3	0.986	0.901477
P49406	39S ribosomal protein L19, mitochondrial OS=Homo sapiens OX=9606 GN=MRPL19 PE=1 SV=2	MRPL19	0.951	0.635302
P49411	Elongation factor Tu, mitochondrial OS=Homo sapiens OX=9606 GN=TUFM PE=1 SV=2	TUFM	1.031	0.72449
P49454	Centromere protein F OS=Homo sapiens OX=9606 GN=CENPF PE=1 SV=3	CENPF	1.015	0.939386
P49458	Signal recognition particle 9 kDa protein OS=Homo sapiens OX=9606 GN=SRP9 PE=1 SV=2	SRP9	0.901	0.216298
P49585	Choline-phosphate cytidylyltransferase A OS=Homo sapiens OX=9606 GN=PCYT1A PE=1 SV=2	PCYT1A	0.943	0.648869
P49588	Alanine--tRNA ligase, cytoplasmic OS=Homo sapiens OX=9606 GN=AARS1 PE=1 SV=2	AARS1	1.051	0.639147
P49589	Cysteine--tRNA ligase, cytoplasmic OS=Homo sapiens OX=9606 GN=CARS1 PE=1	CARS1	0.99	
P49590	Histidine--tRNA ligase, mitochondrial OS=Homo sapiens OX=9606 GN=HARS2 PE=1	HARS2	1.031	0.537993
P49591	Serine--tRNA ligase, cytoplasmic OS=Homo sapiens OX=9606 GN=SARS1 PE=1 SV=3	SARS1	1.255	0.096459
P49643	DNA primase large subunit OS=Homo sapiens OX=9606 GN=PRIM2 PE=1 SV=2	PRIM2	1.018	0.793252
P49662	Caspase-4 OS=Homo sapiens OX=9606 GN=CASP4 PE=1	CASP4	1000	0.001
P49711	Transcriptional repressor CTCF OS=Homo sapiens OX=9606 GN=CTCF PE=1 SV=1	CTCF	1.077	0.241485
P49720	Proteasome subunit beta type-3 OS=Homo sapiens OX=9606 GN=PSMB3 PE=1 SV=2	PSMB3	0.918	0.183416
P49721	Proteasome subunit beta type-2 OS=Homo sapiens OX=9606 GN=PSMB2 PE=1 SV=1	PSMB2	1.033	0.772007
P49736	DNA replication licensing factor MCM2 OS=Homo sapiens OX=9606 GN=MCM2 PE=1	MCM2	0.798	0.054183
P49748	Very long-chain specific acyl-CoA dehydrogenase, mitochondrial OS=Homo sapiens OX=9606 GN=ACADVL PE=1 SV=1	ACADVL	0.978	0.622233

P49750	YLP motif-containing protein 1 OS=Homo sapiens OX=9606 GN=YLPM1 PE=1 SV=4	YLPM1	0.929	0.252226
P49753	Acyl-coenzyme A thioesterase 2, mitochondrial OS=Homo sapiens OX=9606 GN=ACOT2 PE=1	ACOT2	1.124	0.49722
P49755	Transmembrane emp24 domain-containing protein 10 OS=Homo sapiens OX=9606 GN=TMED10 PE=1 SV=2	TMED10	1.028	0.727837
P49756	RNA-binding protein 25 OS=Homo sapiens OX=9606 GN=RBM25 PE=1 SV=3	RBM25	0.915	0.088567
P49757	Protein numb homolog OS=Homo sapiens OX=9606 GN=NUMB PE=1 SV=2	NUMB	1.077	0.663288
P49773	Adenosine 5'-monophosphoramidase HINT1 OS=Homo sapiens OX=9606	HINT1	0.884	0.403332
P49790	Nuclear pore complex protein Nup153 OS=Homo sapiens OX=9606 GN=NUP153 PE=1	NUP153	0.834	0.172876
P49792	E3 SUMO-protein ligase RanBP2 OS=Homo sapiens OX=9606 GN=RANBP2 PE=1 SV=2	RANBP2	0.996	0.91065
P49821	NADH dehydrogenase [ubiquinone] flavoprotein 1, mitochondrial OS=Homo sapiens OX=9606 GN=NDUFV1 PE=1	NDUFV1	0.982	0.758269
P49902	Cytosolic purine 5'-nucleotidase OS=Homo sapiens OX=9606 GN=NT5C2 PE=1 SV=1	NT5C2	1.2	0.305856
P49903	Selenide, water dikinase 1 OS=Homo sapiens OX=9606 GN=SEPHS1 PE=1 SV=2	SEPHS1	0.808	0.158511
P49914	5-formyltetrahydrofolate cyclo-ligase OS=Homo sapiens OX=9606 GN=MTHFS PE=1	MTHFS	1.111	0.183654
P49915	GMP synthase [glutamine-hydrolyzing] OS=Homo sapiens OX=9606 GN=GMPS PE=1 SV=1	GMPS	0.87	0.277015
P49916	DNA ligase 3 OS=Homo sapiens OX=9606 GN=LIG3 PE=1 SV=2	LIG3	0.831	0.129736
P49959	Double-strand break repair protein MRE11 OS=Homo sapiens OX=9606 GN=MRE11 PE=1	MRE11	1.006	0.894185
P50148	Guanine nucleotide-binding protein G(q) subunit alpha OS=Homo sapiens OX=9606 GN=GNAQ PE=1 SV=4	GNAQ	1.126	0.214588

P50213	Isocitrate dehydrogenase [NAD] subunit alpha, mitochondrial OS=Homo sapiens OX=9606 GN=IDH3A PE=1 SV=1	IDH3A	0.994	0.894872
P50281	Matrix metalloproteinase-14 OS=Homo sapiens OX=9606 GN=MMP14 PE=1 SV=3	MMP14	0.98	0.742124
P50395	Rab GDP dissociation inhibitor beta OS=Homo sapiens OX=9606 GN=GDI2 PE=1 SV=2	GDI2	1.134	0.159746
P50402	Emerin OS=Homo sapiens OX=9606 GN=EMD PE=1 SV=1	EMD	1.03	0.261004
P50443	Sulfate transporter OS=Homo sapiens OX=9606 GN=SLC26A2 PE=1 SV=2	SLC26A2	1.093	0.459394
P50454	Serpin H1 OS=Homo sapiens OX=9606 GN=SERPINH1 PE=1 SV=2	SERPINH1	1.025	0.60584
P50502	Hsc70-interacting protein OS=Homo sapiens OX=9606 GN=ST13 PE=1 SV=2	ST13	1.004	0.99038
P50552	Vasodilator-stimulated phosphoprotein OS=Homo sapiens OX=9606 GN=VASP PE=1 SV=3	VASP	1.102	0.063142
P50570	Dynamin-2 OS=Homo sapiens OX=9606 GN=DNM2 PE=1 SV=2	DNM2	1.204	0.080708
P50579	Methionine aminopeptidase 2 OS=Homo sapiens OX=9606 GN=METAP2 PE=1 SV=1	METAP2	1.047	0.677949
P50613	Cyclin-dependent kinase 7 OS=Homo sapiens OX=9606 GN=CDK7 PE=1 SV=1	CDK7	0.926	0.634212
P50750	Cyclin-dependent kinase 9 OS=Homo sapiens OX=9606 GN=CDK9 PE=1 SV=3	CDK9	0.825	0.059644
P50895	Basal cell adhesion molecule OS=Homo sapiens OX=9606 GN=BCAM PE=1 SV=2	BCAM	0.914	0.172313
P50897	Palmitoyl-protein thioesterase 1 OS=Homo sapiens OX=9606 GN=PPT1 PE=1 SV=1	PPT1	0.918	0.152717
P50914	60S ribosomal protein L14 OS=Homo sapiens OX=9606 GN=RPL14 PE=1 SV=4	RPL14	1.31	0.418928
P50990	T-complex protein 1 subunit theta OS=Homo sapiens OX=9606 GN=CCT8 PE=1 SV=4	CCT8	0.935	0.403703
P50991	T-complex protein 1 subunit delta OS=Homo sapiens OX=9606 GN=CCT4 PE=1 SV=4	CCT4	0.931	0.482028
P50995	Annexin A11 OS=Homo sapiens OX=9606 GN=ANXA11 PE=1 SV=1	ANXA11	1.026	0.662044

P51003	Poly(A) polymerase alpha OS=Homo sapiens OX=9606 GN=PAPOLA PE=1 SV=4	PAPOLA	0.874	0.058319
P51114	Fragile X mental retardation syndrome-related protein 1 OS=Homo sapiens OX=9606 GN=FXR1 PE=1 SV=3	FXR1	1.044	0.229774
P51116	Fragile X mental retardation syndrome-related protein 2 OS=Homo sapiens OX=9606 GN=FXR2 PE=1 SV=2	FXR2	1.101	0.067729
P51148	Ras-related protein Rab-5C OS=Homo sapiens OX=9606 GN=RAB5C PE=1 SV=2	RAB5C	0.891	0.077894
P51149	Ras-related protein Rab-7a OS=Homo sapiens OX=9606 GN=RAB7A PE=1 SV=1	RAB7A	0.99	0.775734
P51151	Ras-related protein Rab-9A OS=Homo sapiens OX=9606 GN=RAB9A PE=1 SV=1	RAB9A	1.025	0.798958
P51153	Ras-related protein Rab-13 OS=Homo sapiens OX=9606 GN=RAB13 PE=1 SV=1	RAB13	1.011	0.962921
P51398	28S ribosomal protein S29, mitochondrial OS=Homo sapiens OX=9606 GN=DAP3 PE=1 SV=1	DAP3	0.945	0.450382
P51532	Transcription activator BRG1 OS=Homo sapiens OX=9606 GN=SMARCA4 PE=1 SV=2	SMARCA4	0.9	0.180689
P51553	Isocitrate dehydrogenase [NAD] subunit gamma, mitochondrial OS=Homo sapiens OX=9606 GN=IDH3G PE=1 SV=1	IDH3G	1.021	0.740706
P51571	Translocon-associated protein subunit delta OS=Homo sapiens OX=9606 GN=SSR4 PE=1 SV=1	SSR4	1.057	0.528273
P51572	B-cell receptor-associated protein 31 OS=Homo sapiens OX=9606 GN=BCAP31 PE=1 SV=3	BCAP31	1.096	0.259276
P51608	Methyl-CpG-binding protein 2 OS=Homo sapiens OX=9606 GN=MECP2 PE=1 SV=1	MECP2	0.923	
P51610	Host cell factor 1 OS=Homo sapiens OX=9606 GN=HCFC1	HCFC1	0.877	0.015539
P51636	Caveolin-2 OS=Homo sapiens OX=9606 GN=CAV2 PE=1 SV=2	CAV2	1.113	0.2041
P51648	Aldehyde dehydrogenase family 3 member A2 OS=Homo sapiens OX=9606 GN=ALDH3A2 PE=1 SV=1	ALDH3A2	0.956	0.54804

P51649	Succinate-semialdehyde dehydrogenase, mitochondrial OS=Homo sapiens OX=9606 GN=ALDH5A1 PE=1 SV=2	ALDH5A1	1.072	0.522307
P51659	Peroxisomal multifunctional enzyme type 2 OS=Homo sapiens OX=9606 GN=HSD17B4 PE=1	HSD17B4	0.973	0.551295
P51665	26S proteasome non-ATPase regulatory subunit 7 OS=Homo sapiens OX=9606 GN=PSMD7 PE=1 SV=2	PSMD7	0.986	0.858196
P51784	Ubiquitin carboxyl-terminal hydrolase 11 OS=Homo sapiens OX=9606 GN=USP11 PE=1 SV=3	USP11	0.886	0.494579
P51798	H(+)/Cl(-) exchange transporter 7 OS=Homo sapiens OX=9606 GN=CLCN7 PE=1 SV=2	CLCN7	0.875	
P51808	Dynein light chain Tctex-type 3 OS=Homo sapiens OX=9606 GN=DYNLT3 PE=1 SV=1	DYNLT3	1.206	
P51809	Vesicle-associated membrane protein 7 OS=Homo sapiens OX=9606 GN=VAMP7 PE=1	VAMP7	0.919	0.082576
P51858	Hepatoma-derived growth factor OS=Homo sapiens OX=9606 GN=HDGF PE=1 SV=1	HDGF	0.86	0.15239
P51948	CDK-activating kinase assembly factor MAT1 OS=Homo sapiens OX=9606 GN=MNAT1 PE=1	MNAT1	0.881	0.214272
P51965	Ubiquitin-conjugating enzyme E2 E1 OS=Homo sapiens OX=9606 GN=UBE2E1 PE=1 SV=1	UBE2E1	1.027	
P51970	NADH dehydrogenase [ubiquinone] 1 alpha subcomplex subunit 8 OS=Homo sapiens OX=9606 GN=NDUFA8 PE=1	NDUFA8	1.001	0.985249
P51991	Heterogeneous nuclear ribonucleoprotein A3 OS=Homo sapiens OX=9606 GN=HNRNPA3 PE=1 SV=2	HNRNPA3	0.941	0.02848
P52209	6-phosphogluconate dehydrogenase, decarboxylating OS=Homo sapiens OX=9606	PGD	0.95	0.555452
P52272	Heterogeneous nuclear ribonucleoprotein M OS=Homo sapiens OX=9606 GN=HNRNPM PE=1 SV=3	HNRNPM	0.964	0.378207
P52292	Importin subunit alpha-1 OS=Homo sapiens OX=9606 GN=KPNA2 PE=1 SV=1	KPNA2	1.132	0.198587
P52294	Importin subunit alpha-5 OS=Homo sapiens OX=9606 GN=KPNA1 PE=1 SV=3	KPNA1	1.22	0.192503

P52298	Nuclear cap-binding protein subunit 2 OS=Homo sapiens OX=9606 GN=NCBP2 PE=1	NCBP2	1.023	0.766858
P52434	DNA-directed RNA polymerases I, II, and III subunit RPABC3 OS=Homo sapiens OX=9606 GN=POLR2H PE=1 SV=4	POLR2H	0.923	0.389453
P52565	Rho GDP-dissociation inhibitor 1 OS=Homo sapiens OX=9606 GN=ARHGDIA PE=1 SV=3	ARHGDIA	1.038	0.725842
P52566	Rho GDP-dissociation inhibitor 2 OS=Homo sapiens OX=9606 GN=ARHGDIB PE=1 SV=3	ARHGDIB	1.017	0.931913
P52594	Arf-GAP domain and FG repeat-containing protein 1 OS=Homo sapiens OX=9606 GN=AGFG1 PE=1 SV=2	AGFG1	0.981	0.716496
P52597	Heterogeneous nuclear ribonucleoprotein F OS=Homo sapiens OX=9606 GN=HNRNPF PE=1 SV=3	HNRNPF	0.814	0.031567
P52701	DNA mismatch repair protein Msh6 OS=Homo sapiens OX=9606 GN=MSH6 PE=1 SV=2	MSH6	0.888	0.387982
P52735	Guanine nucleotide exchange factor VAV2 OS=Homo sapiens OX=9606 GN=VAV2 PE=1 SV=2	VAV2	0.924	0.186422
P52756	RNA-binding protein 5 OS=Homo sapiens OX=9606 GN=RBM5 PE=1 SV=2	RBM5	0.874	0.44152
P52758	2-iminobutanoate/2-iminopropanoate deaminase OS=Homo sapiens OX=9606 GN=RIDA PE=1 SV=1	RIDA	1.017	0.91239
P52788	Spermine synthase OS=Homo sapiens OX=9606 GN=SMS PE=1 SV=2	SMS	1.009	0.898639
P52789	Hexokinase-2 OS=Homo sapiens OX=9606 GN=HK2 PE=1 SV=2	HK2	0.941	0.844168
P52799	Ephrin-B2 OS=Homo sapiens OX=9606 GN=EFNB2 PE=1	EFNB2	0.977	0.827559
P52815	39S ribosomal protein L12, mitochondrial OS=Homo sapiens OX=9606 GN=MRPL12 PE=1 SV=2	MRPL12	0.912	0.244236
P52848	Bifunctional heparan sulfate N-deacetylase/N-sulfotransferase 1 OS=Homo sapiens OX=9606 GN=NDST1 PE=1 SV=1	NDST1	1.029	
P52895	Aldo-keto reductase family 1 member C2 OS=Homo sapiens OX=9606 GN=AKR1C2 PE=1 SV=3	AKR1C2	0.979	0.826536

P52907	F-actin-capping protein subunit alpha-1 OS=Homo sapiens OX=9606 GN=CAPZA1 PE=1 SV=3	CAPZA1	1.04	0.738637
P52926	High mobility group protein HMGI-C OS=Homo sapiens OX=9606 GN=HMGA2 PE=1	HMGA2	0.99	0.973475
P52948	Nuclear pore complex protein Nup98-Nup96 OS=Homo sapiens OX=9606 GN=NUP98 PE=1	NUP98	0.991	0.875735
P53007	Tricarboxylate transport protein, mitochondrial OS=Homo sapiens OX=9606 GN=SLC25A1 PE=1 SV=2	SLC25A1	1.008	0.931625
P53041	Serine/threonine-protein phosphatase 5 OS=Homo sapiens OX=9606 GN=PPP5C PE=1 SV=1	PPP5C	1.254	
P53365	Arfaptin-2 OS=Homo sapiens OX=9606 GN=ARFIP2 PE=1	ARFIP2	0.775	0.043379
P53367	Arfaptin-1 OS=Homo sapiens OX=9606 GN=ARFIP1 PE=1	ARFIP1	0.768	0.14987
P53396	ATP-citrate synthase OS=Homo sapiens OX=9606 GN=ACLY PE=1 SV=3	ACLY	0.99	0.846506
P53582	Methionine aminopeptidase 1 OS=Homo sapiens OX=9606 GN=METAP1 PE=1 SV=2	METAP1	1.058	0.165028
P53597	Succinate--CoA ligase [ADP/GDP-forming] subunit alpha, mitochondrial OS=Homo sapiens OX=9606 GN=SUCLG1 PE=1 SV=4	SUCLG1	1.066	0.301217
P53618	Coatomer subunit beta OS=Homo sapiens OX=9606 GN=COPB1 PE=1 SV=3	COPB1	1.025	0.72218
P53621	Coatomer subunit alpha OS=Homo sapiens OX=9606 GN=COPA PE=1 SV=2	COPA	0.979	0.832088
P53634	Dipeptidyl peptidase 1 OS=Homo sapiens OX=9606 GN=CTSC PE=1 SV=2	CTSC	0.874	0.246935
P53680	AP-2 complex subunit sigma OS=Homo sapiens OX=9606 GN=AP2S1 PE=1 SV=2	AP2S1	1.038	0.663502
P53701	Holocytochrome c-type synthase OS=Homo sapiens OX=9606 GN=HCCS PE=1 SV=1	HCCS	1.063	0.474316
P53985	Monocarboxylate transporter 1 OS=Homo sapiens OX=9606 GN=SLC16A1 PE=1 SV=3	SLC16A1	0.834	0.116821
P53990	IST1 homolog OS=Homo sapiens OX=9606 GN=IST1 PE=1 SV=1	IST1	1.261	0.010264

P53992	Protein transport protein Sec24C OS=Homo sapiens OX=9606 GN=SEC24C PE=1 SV=3	SEC24C	1.255	0.077261
P53999	Activated RNA polymerase II transcriptional coactivator p15 OS=Homo sapiens OX=9606 GN=SUB1 PE=1 SV=3	SUB1	0.883	0.35175
P54136	Arginine--tRNA ligase, cytoplasmic OS=Homo sapiens OX=9606 GN=RARS1 PE=1	RARS1	0.981	0.787231
P54289	Voltage-dependent calcium channel subunit alpha-2/delta-1 OS=Homo sapiens OX=9606 GN=CACNA2D1 PE=1 SV=3	CACNA2D1	1.12	0.182975
P54577	Tyrosine--tRNA ligase, cytoplasmic OS=Homo sapiens OX=9606 GN=YARS1 PE=1	YARS1	0.925	0.357372
P54578	Ubiquitin carboxyl-terminal hydrolase 14 OS=Homo sapiens OX=9606 GN=USP14 PE=1 SV=3	USP14	1.017	0.834809
P54709	Sodium/potassium-transporting ATPase subunit beta-3 OS=Homo sapiens OX=9606 GN=ATP1B3 PE=1 SV=1	ATP1B3	1.033	0.464612
P54725	UV excision repair protein RAD23 homolog A OS=Homo sapiens OX=9606 GN=RAD23A PE=1 SV=1	RAD23A	0.781	0.181404
P54727	UV excision repair protein RAD23 homolog B OS=Homo sapiens OX=9606 GN=RAD23B PE=1 SV=1	RAD23B	0.88	0.07981
P54760	Ephrin type-B receptor 4 OS=Homo sapiens OX=9606 GN=EPHB4 PE=1 SV=2	EPHB4	0.963	0.518884
P54764	Ephrin type-A receptor 4 OS=Homo sapiens OX=9606 GN=EPHA4 PE=1 SV=1	EPHA4	0.951	0.703625
P54819	Adenylate kinase 2, mitochondrial OS=Homo sapiens OX=9606 GN=AK2 PE=1 SV=2	AK2	0.99	0.837528
P54886	Delta-1-pyrroline-5-carboxylate synthase OS=Homo sapiens OX=9606 GN=ALDH18A1 PE=1 SV=2	ALDH18A1	0.994	0.942686
P54920	Alpha-soluble NSF attachment protein OS=Homo sapiens OX=9606 GN=NAPA PE=1 SV=3	NAPA	1.252	0.002444
P55010	Eukaryotic translation initiation factor 5 OS=Homo sapiens OX=9606 GN=EIF5 PE=1 SV=2	EIF5	1.085	0.484716

P55011	Solute carrier family 12 member 2 OS=Homo sapiens OX=9606 GN=SLC12A2 PE=1 SV=1	SLC12A2	0.973	0.347622
P55036	26S proteasome non-ATPase regulatory subunit 4 OS=Homo sapiens OX=9606 GN=PSMD4 PE=1 SV=1	PSMD4	1.013	0.831042
P55060	Exportin-2 OS=Homo sapiens OX=9606 GN=CSE1L PE=1	CSE1L	1.113	0.335035
P55061	Bax inhibitor 1 OS=Homo sapiens OX=9606 GN=TMBIM6 PE=1 SV=2	TMBIM6	1.131	0.277693
P55072	Transitional endoplasmic reticulum ATPase OS=Homo sapiens OX=9606 GN=VCP PE=1	VCP	1.033	0.689361
P55081	Microfibrillar-associated protein 1 OS=Homo sapiens OX=9606 GN=MFAP1 PE=1 SV=2	MFAP1	0.967	0.783423
P55084	Trifunctional enzyme subunit beta, mitochondrial OS=Homo sapiens OX=9606 GN=HADHB PE=1	HADHB	1.006	0.957596
P55145	Mesencephalic astrocyte-derived neurotrophic factor OS=Homo sapiens OX=9606 GN=MANF PE=1 SV=3	MANF	0.948	0.55729
P55209	Nucleosome assembly protein 1-like 1 OS=Homo sapiens OX=9606 GN=NAP1L1 PE=1	NAP1L1	1.207	0.053363
P55263	Adenosine kinase OS=Homo sapiens OX=9606 GN=ADK PE=1	ADK	0.978	0.836869
P55265	Double-stranded RNA-specific adenosine deaminase OS=Homo sapiens OX=9606 GN=ADAR PE=1 SV=4	ADAR	0.942	0.382344
P55317	Hepatocyte nuclear factor 3-alpha OS=Homo sapiens OX=9606 GN=FOXA1 PE=1 SV=2	FOXA1	0.782	
P55327	Tumor protein D52 OS=Homo sapiens OX=9606 GN=TPD52 PE=1 SV=2	TPD52	1.229	0.029027
P55735	Protein SEC13 homolog OS=Homo sapiens OX=9606 GN=SEC13 PE=1 SV=3	SEC13	1.013	0.883236
P55769	NHP2-like protein 1 OS=Homo sapiens OX=9606 GN=SNU13 PE=1 SV=3	SNU13	0.979	0.751015
P55786	Puromycin-sensitive aminopeptidase OS=Homo sapiens OX=9606 GN=NPEPPS PE=1	NPEPPS	1.011	0.887624
P55789	FAD-linked sulfhydryl oxidase ALR OS=Homo sapiens OX=9606 GN=GFER PE=1 SV=2	GFER	0.942	0.630527

P55795	Heterogeneous nuclear ribonucleoprotein H2 OS=Homo sapiens OX=9606 GN=HNRNPH2 PE=1 SV=1	HNRNPH2	0.948	0.407949
P55809	Succinyl-CoA:3-ketoacid coenzyme A transferase 1, mitochondrial OS=Homo sapiens OX=9606 GN=OXCT1 PE=1	OXCT1	1.002	0.956879
P55884	Eukaryotic translation initiation factor 3 subunit B OS=Homo sapiens OX=9606 GN=EIF3B PE=1 SV=3	EIF3B	1.046	0.589123
P55957	BH3-interacting domain death agonist OS=Homo sapiens OX=9606 GN=BID PE=1 SV=1	BID	1.188	0.083888
P56134	ATP synthase subunit f, mitochondrial OS=Homo sapiens OX=9606 GN=ATP5MF PE=1 SV=3	ATP5MF	0.895	0.026206
P56182	Ribosomal RNA processing protein 1 homolog A OS=Homo sapiens OX=9606 GN=RRP1	RRP1	0.987	0.844401
P56192	Methionine--tRNA ligase, cytoplasmic OS=Homo sapiens OX=9606 GN=MARS1 PE=1	MARS1	0.88	0.077347
P56270	Myc-associated zinc finger protein OS=Homo sapiens OX=9606 GN=MAZ PE=1 SV=1	MAZ	0.947	0.663744
P56378	ATP synthase subunit ATP5MJ, mitochondrial OS=Homo sapiens OX=9606 GN=ATP5MJ PE=1 SV=1	ATP5MJ	1.022	0.902679
P56385	ATP synthase subunit e, mitochondrial OS=Homo sapiens OX=9606 GN=ATP5ME PE=1 SV=2	ATP5ME	0.896	0.243872
P56537	Eukaryotic translation initiation factor 6 OS=Homo sapiens OX=9606 GN=EIF6 PE=1 SV=1	EIF6	0.821	0.082668
P56545	C-terminal-binding protein 2 OS=Homo sapiens OX=9606 GN=CTBP2 PE=1 SV=1	CTBP2	0.817	0.003406
P56556	NADH dehydrogenase [ubiquinone] 1 alpha subcomplex subunit 6 OS=Homo sapiens OX=9606 GN=NDUFA6 PE=1	NDUFA6	0.94	0.568847
P56962	Syntaxin-17 OS=Homo sapiens OX=9606 GN=STX17 PE=1	STX17	1.1	0.499098
P57088	Transmembrane protein 33 OS=Homo sapiens OX=9606 GN=TMEM33 PE=1 SV=2	TMEM33	0.975	0.932643

P57105	Synaptojanin-2-binding protein OS=Homo sapiens OX=9606 GN=SYNJ2BP PE=1 SV=2	SYNJ2BP	1.173	0.026567
P57735	Ras-related protein Rab-25 OS=Homo sapiens OX=9606 GN=RAB25 PE=1 SV=2	RAB25	1.122	0.279263
P57740	Nuclear pore complex protein Nup107 OS=Homo sapiens OX=9606 GN=NUP107 PE=1	NUP107	1.013	0.892847
P57764	Gasdermin-D OS=Homo sapiens OX=9606 GN=GSDMD PE=1	GSDMD	1.132	
P58107	Epiplakin OS=Homo sapiens OX=9606 GN=EPPK1 PE=1	EPPK1	1.101	0.389169
P58335	Anthrax toxin receptor 2 OS=Homo sapiens OX=9606 GN=ANTXR2 PE=1 SV=5	ANTXR2	1.32	0.018047
P58546	Myotrophin OS=Homo sapiens OX=9606 GN=MTPN PE=1 SV=2	MTPN	1.127	0.736178
Q99879	Histone H2B type 1-M OS=Homo sapiens OX=9606 GN=H2BC14 PE=1 SV=3	H2BC14	1.107	0.459542
P59998	Actin-related protein 2/3 complex subunit 4 OS=Homo sapiens OX=9606 GN=ARPC4 PE=1	ARPC4	1.154	0.042613
P60033	CD81 antigen OS=Homo sapiens OX=9606 GN=CD81 PE=1 SV=1	CD81	1.023	0.700127
P60174	Triosephosphate isomerase OS=Homo sapiens OX=9606 GN=TPI1 PE=1 SV=4	TPI1	0.903	0.423337
P60228	Eukaryotic translation initiation factor 3 subunit E OS=Homo sapiens OX=9606 GN=EIF3E PE=1 SV=1	EIF3E	1.105	0.291319
P60510	Serine/threonine-protein phosphatase 4 catalytic subunit OS=Homo sapiens OX=9606 GN=PPP4C PE=1 SV=1	PPP4C	0.957	0.731478
P60520	Gamma-aminobutyric acid receptor-associated protein-like 2 OS=Homo sapiens OX=9606 GN=GABARAPL2 PE=1 SV=1	GABARAPL2	0.985	0.599887
P60602	Reactive oxygen species modulator 1 OS=Homo sapiens OX=9606 GN=ROMO1 PE=1 SV=1	ROMO1	0.996	0.874068
P60604	Ubiquitin-conjugating enzyme E2 G2 OS=Homo sapiens OX=9606 GN=UBE2G2 PE=1 SV=1	UBE2G2	1.052	0.274023
P60660	Myosin light polypeptide 6 OS=Homo sapiens OX=9606 GN=MYL6 PE=1 SV=2	MYL6	1.192	0.249126
P60842	Eukaryotic initiation factor 4A-I OS=Homo sapiens OX=9606 GN=EIF4A1 PE=1 SV=1	EIF4A1	1.119	0.334239

P60866	40S ribosomal protein S20 OS=Homo sapiens OX=9606 GN=RPS20 PE=1 SV=1	RPS20	0.985	0.83453
P60900	Proteasome subunit alpha type-6 OS=Homo sapiens OX=9606 GN=PSMA6 PE=1 SV=1	PSMA6	0.973	0.71312
P60903	Protein S100-A10 OS=Homo sapiens OX=9606 GN=S100A10 PE=1 SV=2	S100A10	0.98	0.9703
P60953	Cell division control protein 42 homolog OS=Homo sapiens OX=9606 GN=CDC42 PE=1	CDC42	1.029	0.562369
P60981	Destrin OS=Homo sapiens OX=9606 GN=DSTN PE=1 SV=3	DSTN	1.165	0.425409
P60983	Glia maturation factor beta OS=Homo sapiens OX=9606 GN=GMFB PE=1 SV=2	GMFB	0.913	0.346936
P61006	Ras-related protein Rab-8A OS=Homo sapiens OX=9606 GN=RAB8A PE=1 SV=1	RAB8A	1.16	0.005289
P61011	Signal recognition particle 54 kDa protein OS=Homo sapiens OX=9606 GN=SRP54 PE=1 SV=1	SRP54	1.017	0.711831
P61018	Ras-related protein Rab-4B OS=Homo sapiens OX=9606 GN=RAB4B PE=1 SV=1	RAB4B	0.884	0.300653
P61019	Ras-related protein Rab-2A OS=Homo sapiens OX=9606 GN=RAB2A PE=1 SV=1	RAB2A	0.951	0.415795
P61020	Ras-related protein Rab-5B OS=Homo sapiens OX=9606 GN=RAB5B PE=1 SV=1	RAB5B	1.058	0.467622
P61026	Ras-related protein Rab-10 OS=Homo sapiens OX=9606 GN=RAB10 PE=1 SV=1	RAB10	1.032	0.534948
P62837	Ubiquitin-conjugating enzyme E2 D2 OS=Homo sapiens OX=9606 GN=UBE2D2 PE=1 SV=1	UBE2D2	1.18	0.17916
P61081	NEDD8-conjugating enzyme Ubc12 OS=Homo sapiens OX=9606 GN=UBE2M PE=1	UBE2M	0.918	0.504386
P61086	Ubiquitin-conjugating enzyme E2 K OS=Homo sapiens OX=9606 GN=UBE2K PE=1 SV=3	UBE2K	0.856	0.196114
P61088	Ubiquitin-conjugating enzyme E2 N OS=Homo sapiens OX=9606 GN=UBE2N PE=1 SV=1	UBE2N	0.998	0.999684
P61106	Ras-related protein Rab-14 OS=Homo sapiens OX=9606 GN=RAB14 PE=1 SV=4	RAB14	1.033	0.435288
P61158	Actin-related protein 3 OS=Homo sapiens OX=9606 GN=ACTR3 PE=1 SV=3	ACTR3	1.002	0.946949

P61160	Actin-related protein 2 OS=Homo sapiens OX=9606 GN=ACTR2 PE=1 SV=1	ACTR2	1.056	0.195544
P61163	Alpha-centractin OS=Homo sapiens OX=9606 GN=ACTR1A PE=1 SV=1	ACTR1A	1.118	0.151428
P61201	COP9 signalosome complex subunit 2 OS=Homo sapiens OX=9606 GN=COPS2 PE=1	COPS2	1.131	0.208218
P84077	ADP-ribosylation factor 1 OS=Homo sapiens OX=9606 GN=ARF1 PE=1 SV=2	ARF1	0.862	0.306389
P61221	ATP-binding cassette sub-family E member 1 OS=Homo sapiens OX=9606 GN=ABCE1 PE=1	ABCE1	1.023	0.693434
P61224	Ras-related protein Rap-1b OS=Homo sapiens OX=9606 GN=RAP1B PE=1 SV=1	RAP1B	0.983	0.676065
P61225	Ras-related protein Rap-2b OS=Homo sapiens OX=9606 GN=RAP2B PE=1 SV=1	RAP2B	0.982	0.726811
P61247	40S ribosomal protein S3a OS=Homo sapiens OX=9606 GN=RPS3A PE=1 SV=2	RPS3A	1.068	0.431694
P61254	60S ribosomal protein L26 OS=Homo sapiens OX=9606 GN=RPL26 PE=1 SV=1	RPL26	1.181	0.235993
P61289	Proteasome activator complex subunit 3 OS=Homo sapiens OX=9606 GN=PSME3 PE=1	PSME3	0.878	0.028657
P61313	60S ribosomal protein L15 OS=Homo sapiens OX=9606 GN=RPL15 PE=1 SV=2	RPL15	1.17	0.333913
P61353	60S ribosomal protein L27 OS=Homo sapiens OX=9606 GN=RPL27 PE=1 SV=2	RPL27	1.116	0.374242
P61421	V-type proton ATPase subunit d 1 OS=Homo sapiens OX=9606 GN=ATP6V0D1 PE=1 SV=1	ATP6V0D 1	1.029	0.702559
P61513	60S ribosomal protein L37a OS=Homo sapiens OX=9606 GN=RPL37A PE=1 SV=2	RPL37A	1.018	0.926542
P61586	Transforming protein RhoA OS=Homo sapiens OX=9606 GN=RHOA PE=1 SV=1	RHOA	0.992	0.856634
P61604	10 kDa heat shock protein, mitochondrial OS=Homo sapiens OX=9606 GN=HSPE1 PE=1	HSPE1	0.966	0.624855
P61619	Protein transport protein Sec61 subunit alpha isoform 1 OS=Homo sapiens OX=9606 GN=SEC61A1 PE=1 SV=2	SEC61A1	0.935	0.508286

P61758	Prefoldin subunit 3 OS=Homo sapiens OX=9606 GN=VBP1 PE=1 SV=4	VBP1	1.04	0.764562
P61769	Beta-2-microglobulin OS=Homo sapiens OX=9606 GN=B2M PE=1 SV=1	B2M	1.202	0.030759
P61803	Dolichyl-diphosphooligosaccharide--protein glycosyltransferase subunit DAD1 OS=Homo sapiens OX=9606	DAD1	0.971	0.705235
P61916	NPC intracellular cholesterol transporter 2 OS=Homo sapiens OX=9606 GN=NPC2 PE=1 SV=1	NPC2	0.978	0.906694
P61923	Coatomer subunit zeta-1 OS=Homo sapiens OX=9606 GN=COPZ1 PE=1 SV=1	COPZ1	0.992	0.895966
P61960	Ubiquitin-fold modifier 1 OS=Homo sapiens OX=9606 GN=UFM1 PE=1 SV=1	UFM1	0.932	0.35747
P61964	WD repeat-containing protein 5 OS=Homo sapiens OX=9606 GN=WDR5 PE=1 SV=1	WDR5	0.985	0.864088
P61966	AP-1 complex subunit sigma-1A OS=Homo sapiens OX=9606 GN=AP1S1 PE=1 SV=1	AP1S1	0.877	0.231152
P61970	Nuclear transport factor 2 OS=Homo sapiens OX=9606 GN=NUTF2 PE=1 SV=1	NUTF2	1.117	0.461092
P61978	Heterogeneous nuclear ribonucleoprotein K OS=Homo sapiens OX=9606 GN=HNRNPK PE=1 SV=1	HNRNPK	0.828	0.046701
P61981	14-3-3 protein gamma OS=Homo sapiens OX=9606 GN=YWHAG PE=1 SV=2	YWHAG	1.027	0.77342
P62070	Ras-related protein R-Ras2 OS=Homo sapiens OX=9606 GN=RRAS2 PE=1 SV=1	RRAS2	0.834	0.040334
P62072	Mitochondrial import inner membrane translocase subunit Tim10 OS=Homo sapiens OX=9606 GN=TIMM10 PE=1	TIMM10	1.241	0.063297
P62081	40S ribosomal protein S7 OS=Homo sapiens OX=9606 GN=RPS7 PE=1 SV=1	RPS7	1.108	0.048117
P62136	Serine/threonine-protein phosphatase PP1-alpha catalytic subunit OS=Homo sapiens OX=9606 GN=PPP1CA PE=1 SV=1	PPP1CA	1.035	0.358404

P62140	Serine/threonine-protein phosphatase PP1-beta catalytic subunit OS=Homo sapiens OX=9606 GN=PPP1CB PE=1 SV=3	PPP1CB	1.019	0.616661
P62191	26S proteasome regulatory subunit 4 OS=Homo sapiens OX=9606 GN=PSMC1 PE=1 SV=1	PSMC1	1.015	0.834468
P62195	26S proteasome regulatory subunit 8 OS=Homo sapiens OX=9606 GN=PSMC5 PE=1 SV=1	PSMC5	1.025	0.536953
P62241	40S ribosomal protein S8 OS=Homo sapiens OX=9606 GN=RPS8 PE=1 SV=2	RPS8	1.218	0.134882
P62244	40S ribosomal protein S15a OS=Homo sapiens OX=9606 GN=RPS15A PE=1 SV=2	RPS15A	0.933	0.428917
P62249	40S ribosomal protein S16 OS=Homo sapiens OX=9606 GN=RPS16 PE=1 SV=2	RPS16	1.035	0.806263
P62258	14-3-3 protein epsilon OS=Homo sapiens OX=9606 GN=YWHAE PE=1 SV=1	YWHAE	1.004	0.992107
P62263	40S ribosomal protein S14 OS=Homo sapiens OX=9606 GN=RPS14 PE=1 SV=3	RPS14	1.073	0.276591
P62266	40S ribosomal protein S23 OS=Homo sapiens OX=9606 GN=RPS23 PE=1 SV=3	RPS23	1.151	0.308919
P62269	40S ribosomal protein S18 OS=Homo sapiens OX=9606 GN=RPS18 PE=1 SV=3	RPS18	1.002	0.926481
P62273	40S ribosomal protein S29 OS=Homo sapiens OX=9606 GN=RPS29 PE=1 SV=2	RPS29	1.47	0.236583
P62277	40S ribosomal protein S13 OS=Homo sapiens OX=9606 GN=RPS13 PE=1 SV=2	RPS13	1.101	0.60122
P62280	40S ribosomal protein S11 OS=Homo sapiens OX=9606 GN=RPS11 PE=1 SV=3	RPS11	0.959	0.921213
P62304	Small nuclear ribonucleoprotein E OS=Homo sapiens OX=9606 GN=SNRPE PE=1 SV=1	SNRPE	0.967	0.56712
P62306	Small nuclear ribonucleoprotein F OS=Homo sapiens OX=9606 GN=SNRPF PE=1 SV=1	SNRPF	0.998	0.974163
P62310	U6 snRNA-associated Sm-like protein LSm3 OS=Homo sapiens OX=9606 GN=LSM3 PE=1 SV=2	LSM3	0.845	0.133106
P62312	U6 snRNA-associated Sm-like protein LSm6 OS=Homo sapiens OX=9606 GN=LSM6 PE=1 SV=1	LSM6	1.041	0.709147

P62314	Small nuclear ribonucleoprotein Sm D1 OS=Homo sapiens OX=9606 GN=SNRPD1 PE=1	SNRPD1	0.933	0.368463
P62316	Small nuclear ribonucleoprotein Sm D2 OS=Homo sapiens OX=9606 GN=SNRPD2 PE=1	SNRPD2	0.842	0.03964
P62318	Small nuclear ribonucleoprotein Sm D3 OS=Homo sapiens OX=9606 GN=SNRPD3 PE=1	SNRPD3	1.014	0.880384
P62328	Thymosin beta-4 OS=Homo sapiens OX=9606 GN=TMSB4X PE=1 SV=2	TMSB4X	0.916	0.606272
P62330	ADP-ribosylation factor 6 OS=Homo sapiens OX=9606 GN=ARF6 PE=1 SV=2	ARF6	0.925	0.411327
P62333	26S proteasome regulatory subunit 10B OS=Homo sapiens OX=9606 GN=PSMC6 PE=1 SV=1	PSMC6	1.106	0.145056
P62341	Thioredoxin reductase-like selenoprotein T OS=Homo sapiens OX=9606 GN=SELENOT PE=1 SV=2	SELENOT	0.857	
P62424	60S ribosomal protein L7a OS=Homo sapiens OX=9606 GN=RPL7A PE=1 SV=2	RPL7A	1.368	0.343705
P62487	DNA-directed RNA polymerase II subunit RPB7 OS=Homo sapiens OX=9606 GN=POLR2G PE=1 SV=1	POLR2G	1.134	0.344878
P62495	Eukaryotic peptide chain release factor subunit 1 OS=Homo sapiens OX=9606 GN=ETF1 PE=1 SV=3	ETF1	1.128	0.125051
P62633	CCHC-type zinc finger nucleic acid binding protein OS=Homo sapiens OX=9606 GN=CNBP	CNBP	1.062	0.592008
P62701	40S ribosomal protein S4, X isoform OS=Homo sapiens OX=9606 GN=RPS4X PE=1	RPS4X	1.105	0.533824
P62750	60S ribosomal protein L23a OS=Homo sapiens OX=9606 GN=RPL23A PE=1 SV=1	RPL23A	1.058	0.131737
P62753	40S ribosomal protein S6 OS=Homo sapiens OX=9606 GN=RPS6 PE=1 SV=1	RPS6	1.129	0.150188
P62805	Histone H4 OS=Homo sapiens OX=9606 GN=H4C1 PE=1 SV=2	H4C1	1.088	0.811701
P62820	Ras-related protein Rab-1A OS=Homo sapiens OX=9606 GN=RAB1A PE=1 SV=3	RAB1A	1.02	0.750953
P62826	GTP-binding nuclear protein Ran OS=Homo sapiens OX=9606 GN=RAN PE=1 SV=3	RAN	0.954	0.657034

P62829	60S ribosomal protein L23 OS=Homo sapiens OX=9606 GN=RPL23 PE=1 SV=1	RPL23	1.082	0.318866
P62834	Ras-related protein Rap-1A OS=Homo sapiens OX=9606 GN=RAP1A PE=1 SV=1	RAP1A	0.889	0.326535
P62841	40S ribosomal protein S15 OS=Homo sapiens OX=9606 GN=RPS15 PE=1 SV=2	RPS15	0.988	0.845421
P62847	40S ribosomal protein S24 OS=Homo sapiens OX=9606 GN=RPS24 PE=1 SV=1	RPS24	1.06	0.578507
P62851	40S ribosomal protein S25 OS=Homo sapiens OX=9606 GN=RPS25 PE=1 SV=1	RPS25	1.047	0.600569
P62854	40S ribosomal protein S26 OS=Homo sapiens OX=9606 GN=RPS26 PE=1 SV=3	RPS26	0.994	0.942075
P62857	40S ribosomal protein S28 OS=Homo sapiens OX=9606 GN=RPS28 PE=1 SV=1	RPS28	0.904	0.439893
P62873	Guanine nucleotide-binding protein G(I)/G(S)/G(T) subunit beta-1 OS=Homo sapiens OX=9606 GN=GNB1 PE=1 SV=3	GNB1	0.972	0.33747
P62875	DNA-directed RNA polymerases I, II, and III subunit RPABC5 OS=Homo sapiens OX=9606 GN=POLR2L PE=1 SV=1	POLR2L	1.109	0.653858
P62877	E3 ubiquitin-protein ligase RBX1 OS=Homo sapiens OX=9606 GN=RBX1 PE=1 SV=1	RBX1	0.927	0.799245
P62879	Guanine nucleotide-binding protein G(I)/G(S)/G(T) subunit beta-2 OS=Homo sapiens OX=9606 GN=GNB2 PE=1 SV=3	GNB2	1.006	0.8964
P62888	60S ribosomal protein L30 OS=Homo sapiens OX=9606 GN=RPL30 PE=1 SV=2	RPL30	1.038	0.679752
Q59GN2	Putative 60S ribosomal protein L39-like 5 OS=Homo sapiens OX=9606 GN=RPL39P5 PE=5	RPL39P5	1.013	0.911317
P62899	60S ribosomal protein L31 OS=Homo sapiens OX=9606 GN=RPL31 PE=1 SV=1	RPL31	0.95	0.429913
P62906	60S ribosomal protein L10a OS=Homo sapiens OX=9606 GN=RPL10A PE=1 SV=2	RPL10A	1.173	0.266475
P62910	60S ribosomal protein L32 OS=Homo sapiens OX=9606 GN=RPL32 PE=1 SV=2	RPL32	1.045	0.580381

P62913	60S ribosomal protein L11 OS=Homo sapiens OX=9606 GN=RPL11 PE=1 SV=2	RPL11	0.926	0.357418
P62917	60S ribosomal protein L8 OS=Homo sapiens OX=9606 GN=RPL8 PE=1 SV=2	RPL8	1.273	0.366836
P62937	Peptidyl-prolyl cis-trans isomerase A OS=Homo sapiens OX=9606 GN=PPIA PE=1 SV=2	PPIA	0.976	0.790372
P62942	Peptidyl-prolyl cis-trans isomerase FKBP1A OS=Homo sapiens OX=9606 GN=FKBP1A PE=1 SV=2	FKBP1A	1.097	0.463489
P62979	Ubiquitin-40S ribosomal protein S27a OS=Homo sapiens OX=9606 GN=RPS27A PE=1 SV=2	RPS27A	0.843	0.080747
P62993	Growth factor receptor-bound protein 2 OS=Homo sapiens OX=9606 GN=GRB2 PE=1 SV=1	GRB2	0.924	0.566809
P62995	Transformer-2 protein homolog beta OS=Homo sapiens OX=9606 GN=TRA2B PE=1 SV=1	TRA2B	0.996	0.853457
P63000	Ras-related C3 botulinum toxin substrate 1 OS=Homo sapiens OX=9606 GN=RAC1 PE=1 SV=1	RAC1	0.969	0.417613
P63010	AP-2 complex subunit beta OS=Homo sapiens OX=9606 GN=AP2B1 PE=1 SV=1	AP2B1	1.022	0.64011
Q5JWF2	Guanine nucleotide-binding protein G(s) subunit alpha isoforms XLas OS=Homo sapiens OX=9606 GN=GNAS PE=1 SV=2	GNAS	0.953	0.406826
P63096	Guanine nucleotide-binding protein G(i) subunit alpha-1 OS=Homo sapiens OX=9606 GN=GNAI1 PE=1 SV=2	GNAI1	1.097	0.066954
P63098	Calcineurin subunit B type 1 OS=Homo sapiens OX=9606 GN=PPP3R1 PE=1 SV=2	PPP3R1	0.948	0.533011
P63104	14-3-3 protein zeta/delta OS=Homo sapiens OX=9606 GN=YWHAZ PE=1 SV=1	YWHAZ	1.028	0.838589
P63151	Serine/threonine-protein phosphatase 2A 55 kDa regulatory subunit B alpha isoform OS=Homo sapiens OX=9606 GN=PPP2R2A PE=1 SV=1	PPP2R2A	1.103	0.515573
P63165	Small ubiquitin-related modifier 1 OS=Homo sapiens OX=9606 GN=SUMO1 PE=1 SV=1	SUMO1	0.888	0.251277
P63167	Dynein light chain 1, cytoplasmic OS=Homo sapiens OX=9606 GN=DYNLL1 PE=1 SV=1	DYNLL1	1.017	0.77544

P63173	60S ribosomal protein L38 OS=Homo sapiens OX=9606 GN=RPL38 PE=1 SV=2	RPL38	1.173	0.069342
P63208	S-phase kinase-associated protein 1 OS=Homo sapiens OX=9606 GN=SKP1 PE=1 SV=2	SKP1	0.829	0.331585
P63218	Guanine nucleotide-binding protein G(I)/G(S)/G(O) subunit gamma-5 OS=Homo sapiens OX=9606 GN=GNG5 PE=1 SV=3	GNG5	0.91	0.339702
P63220	40S ribosomal protein S21 OS=Homo sapiens OX=9606 GN=RPS21 PE=1 SV=1	RPS21	0.852	0.261731
P63241	Eukaryotic translation initiation factor 5A-1 OS=Homo sapiens OX=9606 GN=EIF5A PE=1 SV=2	EIF5A	0.991	0.881526
P63244	Receptor of activated protein C kinase 1 OS=Homo sapiens OX=9606 GN=RACK1 PE=1	RACK1	0.891	0.297825
P63261	Actin, cytoplasmic 2 OS=Homo sapiens OX=9606 GN=ACTG1 PE=1 SV=1	ACTG1	0.977	0.69445
P63279	SUMO-conjugating enzyme UBC9 OS=Homo sapiens OX=9606 GN=UBE2I PE=1 SV=1	UBE2I	0.867	0.21163
P63313	Thymosin beta-10 OS=Homo sapiens OX=9606 GN=TMSB10 PE=1 SV=2	TMSB10	1.014	0.991397
P67775	Serine/threonine-protein phosphatase 2A catalytic subunit alpha isoform OS=Homo sapiens OX=9606 GN=PPP2CA PE=1 SV=1	PPP2CA	1.024	0.819727
P67809	Y-box-binding protein 1 OS=Homo sapiens OX=9606 GN=YBX1 PE=1 SV=3	YBX1	0.982	0.782893
P67812	Signal peptidase complex catalytic subunit SEC11A OS=Homo sapiens OX=9606 GN=SEC11A	SEC11A	1.11	0.152337
P67870	Casein kinase II subunit beta OS=Homo sapiens OX=9606 GN=CSNK2B PE=1 SV=1	CSNK2B	0.993	0.958872
P67936	Tropomyosin alpha-4 chain OS=Homo sapiens OX=9606 GN=TPM4 PE=1 SV=3	TPM4	0.939	0.49323
P68032	Actin, alpha cardiac muscle 1 OS=Homo sapiens OX=9606 GN=ACTC1 PE=1 SV=1	ACTC1	1.063	0.93666
P68036	Ubiquitin-conjugating enzyme E2 L3 OS=Homo sapiens OX=9606 GN=UBE2L3 PE=1 SV=1	UBE2L3	0.911	0.468175

P68104	Elongation factor 1-alpha 1 OS=Homo sapiens OX=9606 GN=EEF1A1 PE=1 SV=1	EEF1A1	1.003	0.979022
P68363	Tubulin alpha-1B chain OS=Homo sapiens OX=9606 GN=TUBA1B PE=1 SV=1	TUBA1B	1.14	0.388382
P68366	Tubulin alpha-4A chain OS=Homo sapiens OX=9606 GN=TUBA4A PE=1 SV=1	TUBA4A	1.061	0.752453
P68371	Tubulin beta-4B chain OS=Homo sapiens OX=9606 GN=TUBB4B PE=1 SV=1	TUBB4B	1.081	0.692825
P68400	Casein kinase II subunit alpha OS=Homo sapiens OX=9606 GN=CSNK2A1 PE=1 SV=1	CSNK2A1	0.957	0.577481
P68402	Platelet-activating factor acetylhydrolase IB subunit alpha2 OS=Homo sapiens OX=9606 GN=PAFAH1B2 PE=1 SV=1	PAFAH1B 2	1.024	0.787561
P78310	Coxsackievirus and adenovirus receptor OS=Homo sapiens OX=9606 GN=CXADR PE=1	CXADR	0.898	0.283042
P78316	Nucleolar protein 14 OS=Homo sapiens OX=9606 GN=NOP14 PE=1 SV=3	NOP14	0.989	0.845106
P78332	RNA-binding protein 6 OS=Homo sapiens OX=9606 GN=RBM6 PE=1 SV=5	RBM6	0.919	0.484151
P78344	Eukaryotic translation initiation factor 4 gamma 2 OS=Homo sapiens OX=9606 GN=EIF4G2	EIF4G2	1.067	0.170986
P78346	Ribonuclease P protein subunit p30 OS=Homo sapiens OX=9606 GN=RPP30 PE=1 SV=1	RPP30	0.988	0.853355
P78347	General transcription factor II-I OS=Homo sapiens OX=9606 GN=GTF2I PE=1 SV=2	GTF2I	0.921	0.075948
P78362	SRSF protein kinase 2 OS=Homo sapiens OX=9606 GN=SRPK2 PE=1 SV=3	SRPK2	0.95	0.281286
P78368	Casein kinase I isoform gamma-2 OS=Homo sapiens OX=9606 GN=CSNK1G2 PE=1 SV=1	CSNK1G2	0.981	0.822203
P78371	T-complex protein 1 subunit beta OS=Homo sapiens OX=9606 GN=CCT2 PE=1 SV=4	CCT2	0.911	0.302293
P78406	mRNA export factor OS=Homo sapiens OX=9606 GN=RAE1 PE=1 SV=1	RAE1	0.957	0.413711
P78417	Glutathione S-transferase omega-1 OS=Homo sapiens OX=9606 GN=GSTO1 PE=1 SV=2	GSTO1	1.04	0.878328

P78504	Protein jagged-1 OS=Homo sapiens OX=9606 GN=JAG1	JAG1	1.108	0.158986
P78527	DNA-dependent protein kinase catalytic subunit OS=Homo sapiens OX=9606 GN=PRKDC	PRKDC	0.918	0.267829
P78536	Disintegrin and metalloproteinase domain-containing protein 17 OS=Homo sapiens OX=9606 GN=ADAM17 PE=1 SV=1	ADAM17	0.875	0.070474
P78552	Interleukin-13 receptor subunit alpha-1 OS=Homo sapiens OX=9606 GN=IL13RA1 PE=1 SV=1	IL13RA1	1.181	
P79522	Proline-rich protein 3 OS=Homo sapiens OX=9606 GN=PRR3 PE=1 SV=2	PRR3	1.015	0.832426
P80188	Neutrophil gelatinase-associated lipocalin OS=Homo sapiens OX=9606 GN=LCN2 PE=1 SV=2	LCN2	1.313	0.252312
P80303	Nucleobindin-2 OS=Homo sapiens OX=9606 GN=NUCB2 PE=1	NUCB2	0.895	0.03113
P80404	4-aminobutyrate aminotransferase, mitochondrial OS=Homo sapiens OX=9606 GN=ABAT PE=1 SV=3	ABAT	1.201	0.304551
P80723	Brain acid soluble protein 1 OS=Homo sapiens OX=9606 GN=BASP1 PE=1 SV=2	BASP1	1.147	0.442557
P82094	TATA element modulatory factor OS=Homo sapiens OX=9606 GN=TMF1 PE=1 SV=2	TMF1	1.089	
P82650	28S ribosomal protein S22, mitochondrial OS=Homo sapiens OX=9606 GN=MRPS22 PE=1 SV=1	MRPS22	0.934	0.357203
P82663	28S ribosomal protein S25, mitochondrial OS=Homo sapiens OX=9606 GN=MRPS25 PE=1 SV=1	MRPS25	0.97	0.757864
P82664	28S ribosomal protein S10, mitochondrial OS=Homo sapiens OX=9606 GN=MRPS10 PE=1 SV=2	MRPS10	0.919	0.23101
P82673	28S ribosomal protein S35, mitochondrial OS=Homo sapiens OX=9606 GN=MRPS35 PE=1 SV=1	MRPS35	0.952	0.44562
P82675	28S ribosomal protein S5, mitochondrial OS=Homo sapiens OX=9606 GN=MRPS5 PE=1	MRPS5	0.94	0.371525
P82909	28S ribosomal protein S36, mitochondrial OS=Homo sapiens OX=9606 GN=MRPS36 PE=1 SV=2	MRPS36	0.882	0.454217

P82912	28S ribosomal protein S11, mitochondrial OS=Homo sapiens OX=9606 GN=MRPS11 PE=1 SV=2	MRPS11	1.046	0.273708
P82914	28S ribosomal protein S15, mitochondrial OS=Homo sapiens OX=9606 GN=MRPS15 PE=1 SV=1	MRPS15	1.081	0.439614
P82930	28S ribosomal protein S34, mitochondrial OS=Homo sapiens OX=9606 GN=MRPS34 PE=1 SV=2	MRPS34	0.997	0.915845
P82932	28S ribosomal protein S6, mitochondrial OS=Homo sapiens OX=9606 GN=MRPS6 PE=1	MRPS6	0.849	0.021025
P82933	28S ribosomal protein S9, mitochondrial OS=Homo sapiens OX=9606 GN=MRPS9 PE=1	MRPS9	0.959	0.688156
P82970	High mobility group nucleosome-binding domain-containing protein 5 OS=Homo sapiens OX=9606 GN=HMGN5 PE=1 SV=1	HMGN5	0.759	0.048908
P82979	SAP domain-containing ribonucleoprotein OS=Homo sapiens OX=9606 GN=SARNP	SARNP	0.76	0.00873
P83111	Serine beta-lactamase-like protein LACTB, mitochondrial OS=Homo sapiens OX=9606 GN=LACTB PE=1 SV=2	LACTB	1.116	0.185401
P83436	Conserved oligomeric Golgi complex subunit 7 OS=Homo sapiens OX=9606 GN=COG7 PE=1 SV=1	COG7	0.879	
P83731	60S ribosomal protein L24 OS=Homo sapiens OX=9606 GN=RPL24 PE=1 SV=1	RPL24	1.282	0.429353
P83916	Chromobox protein homolog 1 OS=Homo sapiens OX=9606 GN=CBX1 PE=1 SV=1	CBX1	0.844	0.002431
P84022	Mothers against decapentaplegic homolog 3 OS=Homo sapiens OX=9606 GN=SMAD3 PE=1	SMAD3	0.778	0.023372
P84085	ADP-ribosylation factor 5 OS=Homo sapiens OX=9606 GN=ARF5 PE=1 SV=2	ARF5	0.944	0.643081
P84090	Enhancer of rudimentary homolog OS=Homo sapiens OX=9606 GN=ERH PE=1 SV=1	ERH	1.049	0.338502
P84095	Rho-related GTP-binding protein RhoG OS=Homo sapiens OX=9606 GN=RHOG PE=1 SV=1	RHOG	0.937	0.213628

P84098	60S ribosomal protein L19 OS=Homo sapiens OX=9606 GN=RPL19 PE=1 SV=1	RPL19	0.968	0.858735
P84103	Serine/arginine-rich splicing factor 3 OS=Homo sapiens OX=9606 GN=SRSF3 PE=1 SV=1	SRSF3	0.909	0.225694
P84243	Histone H3.3 OS=Homo sapiens OX=9606 GN=H3-3A PE=1 SV=2	H3-3A	1.46	0.351548
P85037	Forkhead box protein K1 OS=Homo sapiens OX=9606 GN=FOXK1 PE=1 SV=1	FOXK1	0.974	0.328289
P98153	Integral membrane protein DGCR2/IDD OS=Homo sapiens OX=9606 GN=DGCR2 PE=1	DGCR2	0.783	
P98175	RNA-binding protein 10 OS=Homo sapiens OX=9606 GN=RBM10 PE=1 SV=3	RBM10	0.893	0.049549
P98179	RNA-binding protein 3 OS=Homo sapiens OX=9606 GN=RBM3 PE=1 SV=1	RBM3	0.925	0.313022
P98194	Calcium-transporting ATPase type 2C member 1 OS=Homo sapiens OX=9606 GN=ATP2C1 PE=1 SV=3	ATP2C1	1.006	0.888052
P99999	Cytochrome c OS=Homo sapiens OX=9606 GN=CYCS PE=1 SV=2	CYCS	0.574	0.018606
Q00059	Transcription factor A, mitochondrial OS=Homo sapiens OX=9606 GN=TFAM PE=1 SV=1	TFAM	0.998	0.916009
Q00169	Phosphatidylinositol transfer protein alpha isoform OS=Homo sapiens OX=9606 GN=PITPNA	PITPNA	1.454	0.320338
Q00325	Phosphate carrier protein, mitochondrial OS=Homo sapiens OX=9606 GN=SLC25A3 PE=1 SV=2	SLC25A3	0.98	0.760801
Q00341	Vigilin OS=Homo sapiens OX=9606 GN=HDLBP PE=1	HDLBP	0.734	0.015962
Q00403	Transcription initiation factor IIB OS=Homo sapiens OX=9606 GN=GTF2B PE=1 SV=1	GTF2B	0.92	0.13938
Q00577	Transcriptional activator protein Pur-alpha OS=Homo sapiens OX=9606 GN=PURA PE=1 SV=2	PURA	1.055	0.39343
Q00587	Cdc42 effector protein 1 OS=Homo sapiens OX=9606 GN=CDC42EP1 PE=1 SV=1	CDC42EP1	1.113	0.525589
Q00610	Clathrin heavy chain 1 OS=Homo sapiens OX=9606 GN=CLTC PE=1 SV=5	CLTC	1.109	0.071625
Q00613	Heat shock factor protein 1 OS=Homo sapiens OX=9606 GN=HSF1 PE=1 SV=1	HSF1	0.826	0.057111

Q00688	Peptidyl-prolyl cis-trans isomerase FKBP3 OS=Homo sapiens OX=9606 GN=FKBP3 PE=1	FKBP3	0.907	0.494272
Q00765	Receptor expression-enhancing protein 5 OS=Homo sapiens OX=9606 GN=REEP5 PE=1	REEP5	1.133	0.159673
Q00796	Sorbitol dehydrogenase OS=Homo sapiens OX=9606 GN=SORD PE=1 SV=4	SORD	0.971	0.849523
Q00839	Heterogeneous nuclear ribonucleoprotein U OS=Homo sapiens OX=9606 GN=HNRNPU PE=1 SV=6	HNRNPU	0.945	0.096264
Q01081	Splicing factor U2AF 35 kDa subunit OS=Homo sapiens OX=9606 GN=U2AF1 PE=1	U2AF1	1.018	0.872485
Q01082	Spectrin beta chain, non-erythrocytic 1 OS=Homo sapiens OX=9606 GN=SPTBN1 PE=1 SV=2	SPTBN1	1.003	0.910884
Q01085	Nucleolysin TIAR OS=Homo sapiens OX=9606 GN=TIAL1 PE=1 SV=1	TIAL1	0.986	0.869555
Q01105	Protein SET OS=Homo sapiens OX=9606 GN=SET PE=1 SV=3	SET	0.98	0.965825
Q01130	Serine/arginine-rich splicing factor 2 OS=Homo sapiens OX=9606 GN=SRSF2 PE=1 SV=4	SRSF2	0.874	0.236512
Q01196	Runt-related transcription factor 1 OS=Homo sapiens OX=9606 GN=RUNX1 PE=1 SV=3	RUNX1	0.93	0.043033
Q01469	Fatty acid-binding protein 5 OS=Homo sapiens OX=9606 GN=FABP5 PE=1 SV=3	FABP5	0.943	0.623946
Q01518	Adenylyl cyclase-associated protein 1 OS=Homo sapiens OX=9606 GN=CAP1 PE=1 SV=5	CAP1	1.158	0.208557
Q01581	Hydroxymethylglutaryl-CoA synthase, cytoplasmic OS=Homo sapiens OX=9606 GN=HMGCS1 PE=1 SV=2	HMGCS1	1.133	0.675617
Q01628	Interferon-induced transmembrane protein 3 OS=Homo sapiens OX=9606 GN=IFITM3 PE=1	IFITM3	0.846	0.520473
Q01650	Large neutral amino acids transporter small subunit 1 OS=Homo sapiens OX=9606 GN=SLC7A5 PE=1 SV=2	SLC7A5	1.019	0.773844
Q01658	Protein Dr1 OS=Homo sapiens OX=9606 GN=DR1 PE=1 SV=1	DR1	1.007	
Q01780	Exosome component 10 OS=Homo sapiens OX=9606 GN=EXOSC10 PE=1 SV=2	EXOSC10	0.918	0.238404

Q01813	ATP-dependent 6-phosphofructokinase, platelet type OS=Homo sapiens OX=9606 GN=PFKP PE=1 SV=2	PFKP	1.061	0.414417
Q01844	RNA-binding protein EWS OS=Homo sapiens OX=9606 GN=EWSR1 PE=1 SV=1	EWSR1	0.894	0.046905
Q01970	1-phosphatidylinositol 4,5-bisphosphate phosphodiesterase beta-3 OS=Homo sapiens OX=9606 GN=PLCB3 PE=1	PLCB3	1.472	
Q02218	2-oxoglutarate dehydrogenase, mitochondrial OS=Homo sapiens OX=9606 GN=OGDH PE=1	OGDH	0.987	0.824945
Q02241	Kinesin-like protein KIF23 OS=Homo sapiens OX=9606 GN=KIF23 PE=1 SV=3	KIF23	0.927	0.247529
Q02297	Pro-neuregulin-1, membrane-bound isoform OS=Homo sapiens OX=9606 GN=NRG1 PE=1 SV=3	NRG1	1.145	
Q02388	Collagen alpha-1(VII) chain OS=Homo sapiens OX=9606 GN=COL7A1 PE=1 SV=2	COL7A1	0.745	0.031967
Q02487	Desmocollin-2 OS=Homo sapiens OX=9606 GN=DSC2 PE=1 SV=1	DSC2	1.027	0.862744
Q02539	Histone H1.1 OS=Homo sapiens OX=9606 GN=H1-1 PE=1 SV=3	H1-1	1.316	0.606871
Q02543	60S ribosomal protein L18a OS=Homo sapiens OX=9606 GN=RPL18A PE=1 SV=2	RPL18A	1.251	0.30754
Q02750	Dual specificity mitogen-activated protein kinase kinase 1 OS=Homo sapiens OX=9606 GN=MAP2K1 PE=1 SV=2	MAP2K1	1.17	0.358373
Q02790	Peptidyl-prolyl cis-trans isomerase FKBP4 OS=Homo sapiens OX=9606 GN=FKBP4 PE=1	FKBP4	1.046	0.610869
Q02809	Procollagen-lysine,2-oxoglutarate 5-dioxygenase 1 OS=Homo sapiens OX=9606 GN=PLOD1	PLOD1	0.921	0.44819
Q02818	Nucleobindin-1 OS=Homo sapiens OX=9606 GN=NUCB1 PE=1	NUCB1	0.89	0.260404
Q02878	60S ribosomal protein L6 OS=Homo sapiens OX=9606 GN=RPL6 PE=1 SV=3	RPL6	1.137	0.654542
Q02880	DNA topoisomerase 2-beta OS=Homo sapiens OX=9606 GN=TOP2B PE=1 SV=3	TOP2B	0.909	0.432874
Q02952	A-kinase anchor protein 12 OS=Homo sapiens OX=9606 GN=AKAP12 PE=1 SV=4	AKAP12	0.933	0.320677

Q02978	Mitochondrial 2-oxoglutarate/malate carrier protein OS=Homo sapiens OX=9606 GN=SLC25A11 PE=1 SV=3	SLC25A11	1.024	0.480562
Q03112	Histone-lysine N-methyltransferase MECOM OS=Homo sapiens OX=9606 GN=MECOM PE=1 SV=3	MECOM	1.039	0.714477
Q03135	Caveolin-1 OS=Homo sapiens OX=9606 GN=CAV1 PE=1 SV=4	CAV1	0.97	0.511936
Q03169	Tumor necrosis factor alpha-induced protein 2 OS=Homo sapiens OX=9606 GN=TNFAIP2 PE=1 SV=2	TNFAIP2	0.929	0.571648
Q03252	Lamin-B2 OS=Homo sapiens OX=9606 GN=LMNB2 PE=1	LMNB2	1.031	0.570738
Q03405	Urokinase plasminogen activator surface receptor OS=Homo sapiens OX=9606 GN=PLAUR PE=1	PLAUR	1.078	0.142113
Q03518	Antigen peptide transporter 1 OS=Homo sapiens OX=9606 GN=TAP1 PE=1 SV=2	TAP1	1.034	0.726113
Q03519	Antigen peptide transporter 2 OS=Homo sapiens OX=9606 GN=TAP2 PE=1 SV=1	TAP2	0.946	0.62452
Q03701	CCAAT/enhancer-binding protein zeta OS=Homo sapiens OX=9606 GN=CEBPZ PE=1 SV=3	CEBPZ	1.027	0.750047
Q04446	1,4-alpha-glucan-branching enzyme OS=Homo sapiens OX=9606 GN=GBE1 PE=1 SV=3	GBE1	1.49	0.068945
Q04637	Eukaryotic translation initiation factor 4 gamma 1 OS=Homo sapiens OX=9606 GN=EIF4G1	EIF4G1	1.115	0.050424
Q04721	Neurogenic locus notch homolog protein 2 OS=Homo sapiens OX=9606 GN=NOTCH2 PE=1 SV=3	NOTCH2	1.135	0.085752
Q04726	Transducin-like enhancer protein 3 OS=Homo sapiens OX=9606 GN=TLE3 PE=1 SV=2	TLE3	1.035	
Q04760	Lactoylglutathione lyase OS=Homo sapiens OX=9606 GN=GLO1 PE=1 SV=4	GLO1	1.048	0.868824
Q04828	Aldo-keto reductase family 1 member C1 OS=Homo sapiens OX=9606 GN=AKR1C1 PE=1 SV=1	AKR1C1	1.095	0.60058
Q04837	Single-stranded DNA-binding protein, mitochondrial OS=Homo sapiens OX=9606 GN=SSBP1 PE=1 SV=1	SSBP1	1.301	0.071646

Q04917	14-3-3 protein eta OS=Homo sapiens OX=9606 GN=YWHAH PE=1 SV=4	YWHAH	0.983	0.836653
Q04941	Proteolipid protein 2 OS=Homo sapiens OX=9606 GN=PLP2 PE=1 SV=1	PLP2	0.949	0.445325
Q05048	Cleavage stimulation factor subunit 1 OS=Homo sapiens OX=9606 GN=CSTF1 PE=1	CSTF1	0.994	0.907084
Q05209	Tyrosine-protein phosphatase non-receptor type 12 OS=Homo sapiens OX=9606 GN=PTPN12	PTPN12	1.255	0.290612
Q05397	Focal adhesion kinase 1 OS=Homo sapiens OX=9606 GN=PTK2 PE=1 SV=2	PTK2	1.142	0.185063
Q05519	Serine/arginine-rich splicing factor 11 OS=Homo sapiens OX=9606 GN=SRSF11 PE=1 SV=1	SRSF11	0.84	0.112084
Q05639	Elongation factor 1-alpha 2 OS=Homo sapiens OX=9606 GN=EEF1A2 PE=1 SV=1	EEF1A2	1.032	0.821636
Q05655	Protein kinase C delta type OS=Homo sapiens OX=9606 GN=PRKCD PE=1 SV=2	PRKCD	0.683	0.013784
Q05682	Caldesmon OS=Homo sapiens OX=9606 GN=CALD1 PE=1	CALD1	0.878	0.379109
Q05D32	CTD small phosphatase-like protein 2 OS=Homo sapiens OX=9606 GN=CTDSPL2 PE=1	CTDSPL2	0.942	0.36712
Q06124	Tyrosine-protein phosphatase non-receptor type 11 OS=Homo sapiens OX=9606 GN=PTPN11	PTPN11	0.993	0.944463
Q06136	3-ketodihydrosphingosine reductase OS=Homo sapiens OX=9606 GN=KDSR PE=1 SV=1	KDSR	1.048	0.391803
Q06210	Glutamine--fructose-6-phosphate aminotransferase [isomerizing] 1 OS=Homo sapiens OX=9606 GN=GFPT1 PE=1 SV=3	GFPT1	1.269	0.02394
Q06265	Exosome complex component RRP45 OS=Homo sapiens OX=9606 GN=EXOSC9 PE=1 SV=3	EXOSC9	1.076	0.593121
Q06323	Proteasome activator complex subunit 1 OS=Homo sapiens OX=9606 GN=PSME1 PE=1	PSME1	0.931	0.518187
Q06481	Amyloid beta precursor like protein 2 OS=Homo sapiens OX=9606 GN=APLP2 PE=1	APLP2	1.103	0.212889
Q06587	E3 ubiquitin-protein ligase RING1 OS=Homo sapiens OX=9606 GN=RING1 PE=1 SV=2	RING1	0.907	0.204146

Q06787	Synaptic functional regulator FMR1 OS=Homo sapiens OX=9606 GN=FMR1 PE=1 SV=1	FMR1	0.919	0.444804
Q06830	Peroxiredoxin-1 OS=Homo sapiens OX=9606 GN=PRDX1	PRDX1	1.017	0.941946
Q07020	60S ribosomal protein L18 OS=Homo sapiens OX=9606 GN=RPL18 PE=1 SV=2	RPL18	1.596	0.238586
Q07021	Complement component 1 Q subcomponent-binding protein, mitochondrial OS=Homo sapiens OX=9606 GN=C1QBP PE=1	C1QBP	1.017	0.83576
Q07065	Cytoskeleton-associated protein 4 OS=Homo sapiens OX=9606 GN=CKAP4 PE=1 SV=2	CKAP4	0.982	0.670486
Q07157	Tight junction protein ZO-1 OS=Homo sapiens OX=9606 GN=TJP1 PE=1 SV=3	TJP1	1.036	0.529009
Q07617	Sperm-associated antigen 1 OS=Homo sapiens OX=9606 GN=SPAG1 PE=1 SV=3	SPAG1	0.939	0.292757
Q07666	KH domain-containing, RNA-binding, signal transduction-associated protein 1 OS=Homo sapiens OX=9606 GN=KHDRBS1 PE=1 SV=1	KHDRBS1	0.933	0.266477
Q07817	Bcl-2-like protein 1 OS=Homo sapiens OX=9606 GN=BCL2L1 PE=1 SV=1	BCL2L1	0.943	0.378141
Q07866	Kinesin light chain 1 OS=Homo sapiens OX=9606 GN=KLC1 PE=1 SV=2	KLC1	1.101	0.360286
Q07955	Serine/arginine-rich splicing factor 1 OS=Homo sapiens OX=9606 GN=SRSF1 PE=1 SV=2	SRSF1	0.903	0.06208
Q07960	Rho GTPase-activating protein 1 OS=Homo sapiens OX=9606 GN=ARHGAP1 PE=1 SV=1	ARHGAP1	1.173	0.019894
Q08170	Serine/arginine-rich splicing factor 4 OS=Homo sapiens OX=9606 GN=SRSF4 PE=1 SV=2	SRSF4	0.975	0.96958
Q08174	Protocadherin-1 OS=Homo sapiens OX=9606 GN=PCDH1	PCDH1	0.951	0.50172
Q08209	Serine/threonine-protein phosphatase 2B catalytic subunit alpha isoform OS=Homo sapiens OX=9606 GN=PPP3CA PE=1 SV=1	PPP3CA	1.201	0.373078
Q08211	ATP-dependent RNA helicase A OS=Homo sapiens OX=9606 GN=DHX9 PE=1 SV=4	DHX9	0.925	0.236664

Q08257	Quinone oxidoreductase OS=Homo sapiens OX=9606 GN=CRYZ PE=1 SV=1	CRYZ	1.036	0.381664
Q08345	Epithelial discoidin domain-containing receptor 1 OS=Homo sapiens OX=9606 GN=DDR1 PE=1 SV=1	DDR1	1.005	0.990109
Q08357	Sodium-dependent phosphate transporter 2 OS=Homo sapiens OX=9606 GN=SLC20A2 PE=1 SV=1	SLC20A2	0.881	0.499931
Q08378	Golgin subfamily A member 3 OS=Homo sapiens OX=9606 GN=GOLGA3 PE=1 SV=2	GOLGA3	1.091	
Q08379	Golgin subfamily A member 2 OS=Homo sapiens OX=9606 GN=GOLGA2 PE=1 SV=3	GOLGA2	0.994	0.906984
Q08380	Galectin-3-binding protein OS=Homo sapiens OX=9606 GN=LGALS3BP PE=1 SV=1	LGALS3BP	1.053	0.268059
Q08426	Peroxisomal bifunctional enzyme OS=Homo sapiens OX=9606 GN=EHHADH PE=1 SV=3	EHHADH	1.018	0.916617
Q08752	Peptidyl-prolyl cis-trans isomerase D OS=Homo sapiens OX=9606 GN=PPID PE=1 SV=3	PPID	0.893	0.419281
Q08945	FACT complex subunit SSRP1 OS=Homo sapiens OX=9606 GN=SSRP1 PE=1 SV=1	SSRP1	0.876	0.123353
Q08AF3	Schlafen family member 5 OS=Homo sapiens OX=9606 GN=SLFN5 PE=1 SV=1	SLFN5	1.022	0.813255
Q08AM6	Protein VAC14 homolog OS=Homo sapiens OX=9606 GN=VAC14 PE=1 SV=1	VAC14	1.063	0.670694
Q08J23	RNA cytosine C(5)-methyltransferase NSUN2 OS=Homo sapiens OX=9606 GN=NSUN2 PE=1 SV=2	NSUN2	0.932	0.392919
Q09028	Histone-binding protein RBBP4 OS=Homo sapiens OX=9606 GN=RBBP4 PE=1 SV=3	RBBP4	0.882	0.029169
Q09161	Nuclear cap-binding protein subunit 1 OS=Homo sapiens OX=9606 GN=NCBP1 PE=1	NCBP1	0.957	0.55067
Q09328	Alpha-1,6-mannosylglycoprotein 6-beta-N-acetylglucosaminyltransferase A OS=Homo sapiens OX=9606 GN=MGAT5 PE=1 SV=1	MGAT5	1000	0.001
Q09472	Histone acetyltransferase p300 OS=Homo sapiens OX=9606 GN=EP300 PE=1 SV=2	EP300	0.924	

Q09666	Neuroblast differentiation-associated protein AHNAK OS=Homo sapiens OX=9606 GN=AHNAK PE=1 SV=2	AHNAK	0.952	0.381011
Q0VDF9	Heat shock 70 kDa protein 14 OS=Homo sapiens OX=9606 GN=HSPA14 PE=1 SV=1	HSPA14	1.159	0.274639
Q0ZGT2	Nexilin OS=Homo sapiens OX=9606 GN=NEXN PE=1 SV=1	NEXN	0.952	0.776451
Q10469	Alpha-1,6-mannosyl-glycoprotein 2-beta-N-acetylglucosaminyltransferase OS=Homo sapiens OX=9606 GN=MGAT2 PE=1 SV=1	MGAT2	1.072	
Q10471	Polypeptide N-acetylgalactosaminyltransferase 2 OS=Homo sapiens OX=9606 GN=GALNT2 PE=1 SV=1	GALNT2	0.997	0.983346
Q10472	Polypeptide N-acetylgalactosaminyltransferase 1 OS=Homo sapiens OX=9606 GN=GALNT1 PE=1 SV=1	GALNT1	0.947	0.467764
Q10567	AP-1 complex subunit beta-1 OS=Homo sapiens OX=9606 GN=AP1B1 PE=1 SV=2	AP1B1	1.041	0.554646
Q10570	Cleavage and polyadenylation specificity factor subunit 1 OS=Homo sapiens OX=9606 GN=CPSF1 PE=1 SV=2	CPSF1	0.938	0.09145
Q10589	Bone marrow stromal antigen 2 OS=Homo sapiens OX=9606 GN=BST2 PE=1 SV=1	BST2	1.073	0.610591
Q10713	Mitochondrial-processing peptidase subunit alpha OS=Homo sapiens OX=9606 GN=PMPCA	PMPCA	0.942	0.361961
Q11201	CMP-N-acetylneuraminate-beta-galactosamide-alpha-2,3-sialyltransferase 1 OS=Homo sapiens OX=9606 GN=ST3GAL1 PE=1 SV=1	ST3GAL1	1.054	0.364673
Q12765	Secernin-1 OS=Homo sapiens OX=9606 GN=SCRN1 PE=1	SCRN1	1.269	0.631365
Q12768	WASH complex subunit 5 OS=Homo sapiens OX=9606 GN=WASHC5 PE=1 SV=1	WASHC5	1.117	0.467673
Q12769	Nuclear pore complex protein Nup160 OS=Homo sapiens OX=9606 GN=NUP160 PE=1	NUP160	1.071	0.246063
Q12788	Transducin beta-like protein 3 OS=Homo sapiens OX=9606 GN=TBL3 PE=1 SV=2	TBL3	0.972	0.595012

Q12789	General transcription factor 3C polypeptide 1 OS=Homo sapiens OX=9606 GN=GTF3C1 PE=1	GTF3C1	0.958	0.521234
Q12792	Twinfilin-1 OS=Homo sapiens OX=9606 GN=TWF1 PE=1 SV=3	TWF1	1.091	0.016072
Q12797	Aspartyl/asparaginyl beta-hydroxylase OS=Homo sapiens OX=9606 GN=ASPH PE=1 SV=3	ASPH	1.016	0.841507
Q12800	Alpha-globin transcription factor CP2 OS=Homo sapiens OX=9606 GN=TFCP2 PE=1 SV=2	TFCP2	0.962	0.665323
Q12805	EGF-containing fibulin-like extracellular matrix protein 1 OS=Homo sapiens OX=9606 GN=EFEMP1 PE=1 SV=2	EFEMP1	1.168	0.123022
Q12824	SWI/SNF-related matrix-associated actin-dependent regulator of chromatin subfamily B member 1 OS=Homo sapiens OX=9606 GN=SMARCB1 PE=1	SMARCB1	0.921	0.231431
Q12834	Cell division cycle protein 20 homolog OS=Homo sapiens OX=9606 GN=CDC20 PE=1	CDC20	0.951	0.885408
Q12846	Syntaxin-4 OS=Homo sapiens OX=9606 GN=STX4 PE=1 SV=2	STX4	1.051	0.610969
Q12849	G-rich sequence factor 1 OS=Homo sapiens OX=9606 GN=GRSF1 PE=1 SV=3	GRSF1	1.045	0.376002
Q12860	Contactin-1 OS=Homo sapiens OX=9606 GN=CNTN1 PE=1	CNTN1	1.018	0.955517
Q12872	Splicing factor, suppressor of white-apricot homolog OS=Homo sapiens OX=9606 GN=SFSWAP PE=1 SV=3	SFSWAP	0.977	0.849546
Q12873	Chromodomain-helicase-DNA-binding protein 3 OS=Homo sapiens OX=9606 GN=CHD3	CHD3	1.018	0.692145
Q12874	Splicing factor 3A subunit 3 OS=Homo sapiens OX=9606 GN=SF3A3 PE=1 SV=1	SF3A3	0.905	0.053204
Q12888	TP53-binding protein 1 OS=Homo sapiens OX=9606 GN=TP53BP1 PE=1 SV=2	TP53BP1	0.917	0.184183
Q12904	Aminoacyl tRNA synthase complex-interacting multifunctional protein 1 OS=Homo sapiens OX=9606	AIMP1	0.926	0.399118
Q12905	Interleukin enhancer-binding factor 2 OS=Homo sapiens OX=9606 GN=ILF2 PE=1 SV=2	ILF2	0.989	0.785533
Q12906	Interleukin enhancer-binding factor 3 OS=Homo sapiens OX=9606 GN=ILF3 PE=1 SV=3	ILF3	0.917	0.046908

Q12907	Vesicular integral-membrane protein VIP36 OS=Homo sapiens OX=9606 GN=LMAN2 PE=1	LMAN2	1.04	0.418211
Q12929	Epidermal growth factor receptor kinase substrate 8 OS=Homo sapiens OX=9606 GN=EPS8 PE=1	EPS8	1.051	0.268926
Q12931	Heat shock protein 75 kDa, mitochondrial OS=Homo sapiens OX=9606 GN=TRAP1 PE=1	TRAP1	1.004	0.935229
Q12955	Ankyrin-3 OS=Homo sapiens OX=9606 GN=ANK3 PE=1 SV=3	ANK3	1.038	0.688234
Q12959	Disks large homolog 1 OS=Homo sapiens OX=9606 GN=DLG1 PE=1 SV=2	DLG1	0.915	0.013534
Q12972	Nuclear inhibitor of protein phosphatase 1 OS=Homo sapiens OX=9606 GN=PPP1R8 PE=1	PPP1R8	0.816	0.085554
Q12974	Protein tyrosine phosphatase type IVA 2 OS=Homo sapiens OX=9606 GN=PTP4A2 PE=1	PTP4A2	1.18	0.351347
Q12996	Cleavage stimulation factor subunit 3 OS=Homo sapiens OX=9606 GN=CSTF3 PE=1	CSTF3	0.988	0.776516
Q13011	Delta(3,5)-Delta(2,4)-dienoyl-CoA isomerase, mitochondrial OS=Homo sapiens OX=9606 GN=ECH1 PE=1 SV=2	ECH1	1.028	0.74331
Q13033	Striatin-3 OS=Homo sapiens OX=9606 GN=STRN3 PE=1	STRN3	0.89	
Q13045	Protein flightless-1 homolog OS=Homo sapiens OX=9606 GN=FLII PE=1 SV=2	FLII	1.029	0.645178
Q13057	Bifunctional coenzyme A synthase OS=Homo sapiens OX=9606 GN=COASY PE=1 SV=4	COASY	0.989	0.96732
Q13084	39S ribosomal protein L28, mitochondrial OS=Homo sapiens OX=9606 GN=MRPL28 PE=1 SV=4	MRPL28	1.182	0.351213
Q13085	Acetyl-CoA carboxylase 1 OS=Homo sapiens OX=9606 GN=ACACA PE=1 SV=2	ACACA	1.042	0.501207
Q13098	COP9 signalosome complex subunit 1 OS=Homo sapiens OX=9606 GN=GPS1 PE=1 SV=4	GPS1	0.966	0.617659
Q13111	Chromatin assembly factor 1 subunit A OS=Homo sapiens OX=9606 GN=CHAF1A PE=1	CHAF1A	1.088	0.470407
Q13123	Protein Red OS=Homo sapiens OX=9606 GN=IK PE=1 SV=3	IK	0.984	0.912062
Q13126	S-methyl-5'-thioadenosine phosphorylase OS=Homo sapiens OX=9606 GN=MTAP PE=1 SV=2	MTAP	1.004	0.950692

Q13136	Liprin-alpha-1 OS=Homo sapiens OX=9606 GN=PPFIA1 PE=1	PPFIA1	1.092	0.00891
Q13148	TAR DNA-binding protein 43 OS=Homo sapiens OX=9606 GN=TARDBP PE=1 SV=1	TARDBP	0.918	0.03908
Q13151	Heterogeneous nuclear ribonucleoprotein A0 OS=Homo sapiens OX=9606 GN=HNRNPA0 PE=1 SV=1	HNRNPA0	0.896	0.081516
Q13155	Aminoacyl tRNA synthase complex-interacting multifunctional protein 2 OS=Homo sapiens OX=9606	AIMP2	1.04	0.558691
Q13158	FAS-associated death domain protein OS=Homo sapiens OX=9606 GN=FADD PE=1 SV=1	FADD	0.876	0.356284
Q13162	Peroxiredoxin-4 OS=Homo sapiens OX=9606 GN=PRDX4	PRDX4	0.963	0.490371
Q13177	Serine/threonine-protein kinase PAK 2 OS=Homo sapiens OX=9606 GN=PAK2 PE=1 SV=3	PAK2	1.051	0.591525
Q13185	Chromobox protein homolog 3 OS=Homo sapiens OX=9606 GN=CBX3 PE=1 SV=4	CBX3	0.804	0.028319
Q13190	Syntaxin-5 OS=Homo sapiens OX=9606 GN=STX5 PE=1 SV=2	STX5	0.875	0.032685
Q13200	26S proteasome non-ATPase regulatory subunit 2 OS=Homo sapiens OX=9606 GN=PSMD2 PE=1 SV=3	PSMD2	1.031	0.526594
Q13206	Probable ATP-dependent RNA helicase DDX10 OS=Homo sapiens OX=9606 GN=DDX10	DDX10	1.022	0.922446
Q13217	DnaJ homolog subfamily C member 3 OS=Homo sapiens OX=9606 GN=DNAJC3 PE=1	DNAJC3	0.989	0.821556
Q13232	Nucleoside diphosphate kinase 3 OS=Homo sapiens OX=9606 GN=NME3 PE=1 SV=2	NME3	0.914	0.267786
Q13242	Serine/arginine-rich splicing factor 9 OS=Homo sapiens OX=9606 GN=SRSF9 PE=1 SV=1	SRSF9	1.058	0.447557
Q13243	Serine/arginine-rich splicing factor 5 OS=Homo sapiens OX=9606 GN=SRSF5 PE=1 SV=1	SRSF5	0.794	0.014378
Q13247	Serine/arginine-rich splicing factor 6 OS=Homo sapiens OX=9606 GN=SRSF6 PE=1 SV=2	SRSF6	0.894	0.015349
Q13257	Mitotic spindle assembly checkpoint protein MAD2A OS=Homo sapiens OX=9606 GN=MAD2L1 PE=1 SV=1	MAD2L1	2.48	

Q13263	Transcription intermediary factor 1-beta OS=Homo sapiens OX=9606 GN=TRIM28 PE=1	TRIM28	0.921	0.10255
Q13277	Syntaxin-3 OS=Homo sapiens OX=9606 GN=STX3 PE=1 SV=3	STX3	1.048	0.280724
Q13283	Ras GTPase-activating protein-binding protein 1 OS=Homo sapiens OX=9606 GN=G3BP1	G3BP1	0.982	0.773215
Q13308	Inactive tyrosine-protein kinase 7 OS=Homo sapiens OX=9606 GN=PTK7 PE=1 SV=2	PTK7	0.889	0.086423
Q13310	Polyadenylate-binding protein 4 OS=Homo sapiens OX=9606 GN=PABPC4 PE=1 SV=1	PABPC4	0.973	0.769524
Q13315	Serine-protein kinase ATM OS=Homo sapiens OX=9606 GN=ATM PE=1 SV=4	ATM	0.001	0.001
Q13330	Metastasis-associated protein MTA1 OS=Homo sapiens OX=9606 GN=MTA1 PE=1 SV=2	MTA1	0.916	0.436543
Q13332	Receptor-type tyrosine-protein phosphatase S OS=Homo sapiens OX=9606 GN=PTPRS PE=1	PTPRS	0.927	0.5082
Q13347	Eukaryotic translation initiation factor 3 subunit I OS=Homo sapiens OX=9606 GN=EIF3I	EIF3I	1.041	0.630845
Q13363	C-terminal-binding protein 1 OS=Homo sapiens OX=9606 GN=CTBP1 PE=1 SV=2	CTBP1	1.076	0.113546
Q13404	Ubiquitin-conjugating enzyme E2 variant 1 OS=Homo sapiens OX=9606 GN=UBE2V1 PE=1 SV=2	UBE2V1	1.158	0.237743
Q13405	39S ribosomal protein L49, mitochondrial OS=Homo sapiens OX=9606 GN=MRPL49 PE=1 SV=1	MRPL49	0.936	0.438884
Q13409	Cytoplasmic dynein 1 intermediate chain 2 OS=Homo sapiens OX=9606 GN=DYNC1I2 PE=1 SV=3	DYNC1I2	1.146	0.40507
Q13416	Origin recognition complex subunit 2 OS=Homo sapiens OX=9606 GN=ORC2 PE=1 SV=2	ORC2	1.053	
Q13418	Integrin-linked protein kinase OS=Homo sapiens OX=9606 GN=ILK PE=1 SV=2	ILK	1.007	0.969659
Q13423	NAD(P) transhydrogenase, mitochondrial OS=Homo sapiens OX=9606 GN=NNT PE=1 SV=3	NNT	1.053	0.190432
Q13425	Beta-2-syntrophin OS=Homo sapiens OX=9606 GN=SNTB2 PE=1 SV=1	SNTB2	1.049	0.185973

Q13427	Peptidyl-prolyl cis-trans isomerase G OS=Homo sapiens OX=9606 GN=PPIG PE=1 SV=2	PPIG	0.903	0.142653
Q13428	Treacle protein OS=Homo sapiens OX=9606 GN=TCOF1 PE=1	TCOF1	0.906	0.480286
Q13435	Splicing factor 3B subunit 2 OS=Homo sapiens OX=9606 GN=SF3B2 PE=1 SV=2	SF3B2	0.927	0.210473
Q13438	Protein OS-9 OS=Homo sapiens OX=9606 GN=OS9 PE=1 SV=1	OS9	1.1	0.25346
Q13439	Golgin subfamily A member 4 OS=Homo sapiens OX=9606 GN=GOLGA4 PE=1 SV=1	GOLGA4	0.939	0.148835
Q13442	28 kDa heat- and acid-stable phosphoprotein OS=Homo sapiens OX=9606 GN=PDAP1 PE=1	PDAP1	1.042	0.801226
Q13443	Disintegrin and metalloproteinase domain-containing protein 9 OS=Homo sapiens OX=9606 GN=ADAM9 PE=1 SV=1	ADAM9	1.055	0.486453
Q13445	Transmembrane emp24 domain-containing protein 1 OS=Homo sapiens OX=9606 GN=TMED1 PE=1 SV=1	TMED1	0.982	0.673418
Q13454	Tumor suppressor candidate 3 OS=Homo sapiens OX=9606 GN=TUSC3 PE=1 SV=1	TUSC3	1	0.967688
Q13492	Phosphatidylinositol-binding clathrin assembly protein OS=Homo sapiens OX=9606 GN=PICALM PE=1 SV=2	PICALM	1.13	0.057595
Q13501	Sequestosome-1 OS=Homo sapiens OX=9606 GN=SQSTM1 PE=1 SV=1	SQSTM1	1.098	0.433804
Q13505	Metaxin-1 OS=Homo sapiens OX=9606 GN=MTX1 PE=1 SV=3	MTX1	1.086	0.131871
Q13509	Tubulin beta-3 chain OS=Homo sapiens OX=9606 GN=TUBB3 PE=1 SV=2	TUBB3	1.138	0.468392
Q13510	Acid ceramidase OS=Homo sapiens OX=9606 GN=ASAH1	ASAH1	1.163	0.126177
Q13523	Serine/threonine-protein kinase PRP4 homolog OS=Homo sapiens OX=9606 GN=PRPF4B PE=1 SV=3	PRPF4B	0.944	0.262984
Q13530	Serine incorporator 3 OS=Homo sapiens OX=9606 GN=SERINC3 PE=1 SV=2	SERINC3	1.027	0.837179
Q13547	Histone deacetylase 1 OS=Homo sapiens OX=9606 GN=HDAC1 PE=1 SV=1	HDAC1	0.894	0.016182

Q13555	Calcium/calmodulin-dependent protein kinase type II subunit gamma OS=Homo sapiens OX=9606 GN=CAMK2G PE=1	CAMK2G	1.352	0.11321
Q13561	Dynactin subunit 2 OS=Homo sapiens OX=9606 GN=DCTN2 PE=1 SV=4	DCTN2	1.146	0.330891
Q13564	NEDD8-activating enzyme E1 regulatory subunit OS=Homo sapiens OX=9606 GN=NAE1 PE=1 SV=1	NAE1	0.985	0.857114
Q13571	Lysosomal-associated transmembrane protein 5 OS=Homo sapiens OX=9606 GN=LAPTM5 PE=1 SV=1	LAPTM5	0.93	0.351698
Q13573	SNW domain-containing protein 1 OS=Homo sapiens OX=9606 GN=SNW1 PE=1 SV=1	SNW1	0.9	0.07503
Q13586	Stromal interaction molecule 1 OS=Homo sapiens OX=9606 GN=STIM1 PE=1 SV=3	STIM1	1.006	0.841718
Q13595	Transformer-2 protein homolog alpha OS=Homo sapiens OX=9606 GN=TRA2A PE=1	TRA2A	0.961	0.621152
Q13596	Sorting nexin-1 OS=Homo sapiens OX=9606 GN=SNX1 PE=1 SV=3	SNX1	0.928	0.601502
Q13601	KRR1 small subunit processome component homolog OS=Homo sapiens OX=9606 GN=KRR1 PE=1 SV=4	KRR1	1.118	0.712101
Q13610	Periodic tryptophan protein 1 homolog OS=Homo sapiens OX=9606 GN=PWP1 PE=1 SV=1	PWP1	1.019	0.698116
Q13616	Cullin-1 OS=Homo sapiens OX=9606 GN=CUL1 PE=1 SV=2	CUL1	0.857	0.12546
Q13617	Cullin-2 OS=Homo sapiens OX=9606 GN=CUL2 PE=1 SV=2	CUL2	0.947	0.64001
Q13618	Cullin-3 OS=Homo sapiens OX=9606 GN=CUL3 PE=1 SV=2	CUL3	1	0.998707
Q13619	Cullin-4A OS=Homo sapiens OX=9606 GN=CUL4A PE=1	CUL4A	0.973	0.410222
Q13620	Cullin-4B OS=Homo sapiens OX=9606 GN=CUL4B PE=1	CUL4B	0.994	0.951387
Q13637	Ras-related protein Rab-32 OS=Homo sapiens OX=9606 GN=RAB32 PE=1 SV=3	RAB32	1.057	0.485539
Q13641	Trophoblast glycoprotein OS=Homo sapiens OX=9606 GN=TPBG PE=1 SV=1	TPBG	1.047	0.664392
Q13724	Mannosyl-oligosaccharide glucosidase OS=Homo sapiens OX=9606 GN=MOGS PE=1	MOGS	1	0.979379

Q13740	CD166 antigen OS=Homo sapiens OX=9606 GN=ALCAM PE=1 SV=2	ALCAM	0.942	0.128882
Q13751	Laminin subunit beta-3 OS=Homo sapiens OX=9606 GN=LAMB3 PE=1 SV=1	LAMB3	1.153	0.037114
Q13753	Laminin subunit gamma-2 OS=Homo sapiens OX=9606 GN=LAMC2 PE=1 SV=2	LAMC2	1.208	0.010333
Q13769	THO complex subunit 5 homolog OS=Homo sapiens OX=9606 GN=THOC5 PE=1 SV=2	THOC5	0.994	0.889551
Q13772	Nuclear receptor coactivator 4 OS=Homo sapiens OX=9606 GN=NCOA4 PE=1 SV=1	NCOA4	1.127	0.368451
Q13795	ADP-ribosylation factor-related protein 1 OS=Homo sapiens OX=9606 GN=ARFRP1 PE=1 SV=1	ARFRP1	1.091	0.259449
Q13813	Spectrin alpha chain, non-erythrocytic 1 OS=Homo sapiens OX=9606 GN=SPTAN1 PE=1 SV=3	SPTAN1	1.033	0.502831
Q13823	Nucleolar GTP-binding protein 2 OS=Homo sapiens OX=9606 GN=GNL2 PE=1 SV=1	GNL2	1.036	0.768153
Q13838	Spliceosome RNA helicase DDX39B OS=Homo sapiens OX=9606 GN=DDX39B PE=1 SV=1	DDX39B	0.885	0.015976
Q13868	Exosome complex component RRP4 OS=Homo sapiens OX=9606 GN=EXOSC2 PE=1	EXOSC2	0.996	0.921736
Q13873	Bone morphogenetic protein receptor type-2 OS=Homo sapiens OX=9606 GN=BMPR2 PE=1	BMPR2	0.806	
Q13895	Bystin OS=Homo sapiens OX=9606 GN=BYSL PE=1 SV=3	BYSL	0.926	0.534775
Q13907	Isopentenyl-diphosphate Delta-isomerase 1 OS=Homo sapiens OX=9606 GN=IDI1 PE=1 SV=2	IDI1	0.959	0.759806
Q13948	Protein CASP OS=Homo sapiens OX=9606 GN=CUX1 PE=1 SV=2	CUX1	1.003	0.944106
Q13951	Core-binding factor subunit beta OS=Homo sapiens OX=9606 GN=CBFB PE=1 SV=2	CBFB	0.937	0.293278
Q13952	Nuclear transcription factor Y subunit gamma OS=Homo sapiens OX=9606 GN=NFYC PE=1 SV=3	NFYC	0.947	0.621932
Q14004	Cyclin-dependent kinase 13 OS=Homo sapiens OX=9606 GN=CDK13 PE=1 SV=2	CDK13	0.918	0.444077

Q14008	Cytoskeleton-associated protein 5 OS=Homo sapiens OX=9606 GN=CKAP5 PE=1 SV=3	CKAP5	1.06	0.501585
Q14011	Cold-inducible RNA-binding protein OS=Homo sapiens OX=9606 GN=CIRBP PE=1	CIRBP	0.868	0.193235
Q14019	Coactosin-like protein OS=Homo sapiens OX=9606 GN=COTL1 PE=1 SV=3	COTL1	1.101	0.413824
Q14061	Cytochrome c oxidase copper chaperone OS=Homo sapiens OX=9606 GN=COX17 PE=1	COX17	1.04	0.837703
Q14103	Heterogeneous nuclear ribonucleoprotein D0 OS=Homo sapiens OX=9606 GN=HNRNPD PE=1 SV=1	HNRNPD	0.928	0.014581
Q14108	Lysosome membrane protein 2 OS=Homo sapiens OX=9606 GN=SCARB2 PE=1 SV=2	SCARB2	1.09	0.137378
Q14116	Interleukin-18 OS=Homo sapiens OX=9606 GN=IL18 PE=1 SV=1	IL18	0.979	0.795729
Q14118	Dystroglycan 1 OS=Homo sapiens OX=9606 GN=DAG1 PE=1 SV=2	DAG1	0.811	0.060517
Q14119	Vascular endothelial zinc finger 1 OS=Homo sapiens OX=9606 GN=VEZF1 PE=1 SV=2	VEZF1	0.942	0.52394
Q14126	Desmoglein-2 OS=Homo sapiens OX=9606 GN=DSG2 PE=1 SV=2	DSG2	0.97	0.635768
Q14134	Tripartite motif-containing protein 29 OS=Homo sapiens OX=9606 GN=TRIM29 PE=1 SV=2	TRIM29	0.903	0.093141
Q14137	Ribosome biogenesis protein BOP1 OS=Homo sapiens OX=9606 GN=BOP1 PE=1 SV=2	BOP1	1.062	0.202584
Q14146	Unhealthy ribosome biogenesis protein 2 homolog OS=Homo sapiens OX=9606 GN=URB2 PE=1 SV=2	URB2	1.097	0.503212
Q14151	Scaffold attachment factor B2 OS=Homo sapiens OX=9606 GN=SAFB2 PE=1 SV=1	SAFB2	0.929	0.387425
Q14152	Eukaryotic translation initiation factor 3 subunit A OS=Homo sapiens OX=9606 GN=EIF3A PE=1 SV=1	EIF3A	1.02	0.726459
Q14156	Protein EFR3 homolog A OS=Homo sapiens OX=9606 GN=EFR3A PE=1 SV=2	EFR3A	0.909	0.246462
Q14157	Ubiquitin-associated protein 2-like OS=Homo sapiens OX=9606 GN=UBAP2L PE=1 SV=2	UBAP2L	0.826	0.170456

Q14160	Protein scribble homolog OS=Homo sapiens OX=9606 GN=SCRIB PE=1 SV=4	SCRIB	1.034	0.676226
Q14165	Malectin OS=Homo sapiens OX=9606 GN=MLEC PE=1 SV=1	MLEC	0.981	0.68228
Q14166	Tubulin--tyrosine ligase-like protein 12 OS=Homo sapiens OX=9606 GN=TTLL12 PE=1	TTLL12	1.018	0.870371
Q14192	Four and a half LIM domains protein 2 OS=Homo sapiens OX=9606 GN=FHL2 PE=1 SV=3	FHL2	1.077	0.560685
Q14195	Dihydropyrimidinase-related protein 3 OS=Homo sapiens OX=9606 GN=DPYSL3 PE=1	DPYSL3	1.212	0.157884
Q14197	Peptidyl-tRNA hydrolase ICT1, mitochondrial OS=Homo sapiens OX=9606 GN=MRPL58 PE=1 SV=1	MRPL58	0.981	0.779306
Q14202	Zinc finger MYM-type protein 3 OS=Homo sapiens OX=9606 GN=ZMYM3 PE=1 SV=2	ZMYM3	1.162	0.110068
Q14203	Dynactin subunit 1 OS=Homo sapiens OX=9606 GN=DCTN1 PE=1 SV=3	DCTN1	1.127	0.23169
Q14204	Cytoplasmic dynein 1 heavy chain 1 OS=Homo sapiens OX=9606 GN=DYNC1H1 PE=1 SV=5	DYNC1H1	1.191	0.073371
Q14232	Translation initiation factor eIF-2B subunit alpha OS=Homo sapiens OX=9606 GN=EIF2B1 PE=1	EIF2B1	1.118	0.630314
Q14240	Eukaryotic initiation factor 4A-II OS=Homo sapiens OX=9606 GN=EIF4A2 PE=1 SV=2	EIF4A2	1.126	0.327987
Q14244	Ensconsin OS=Homo sapiens OX=9606 GN=MAP7 PE=1 SV=1	MAP7	1.144	0.293492
Q14247	Src substrate cortactin OS=Homo sapiens OX=9606 GN=CTTN PE=1 SV=2	CTTN	1.104	0.046736
Q14254	Flotillin-2 OS=Homo sapiens OX=9606 GN=FLOT2 PE=1	FLOT2	0.992	0.91612
Q14257	Reticulocalbin-2 OS=Homo sapiens OX=9606 GN=RCN2	RCN2	1.025	0.812154
Q14258	E3 ubiquitin/ISG15 ligase TRIM25 OS=Homo sapiens OX=9606 GN=TRIM25 PE=1 SV=2	TRIM25	1.166	0.044026
Q14318	Peptidyl-prolyl cis-trans isomerase FKBP8 OS=Homo sapiens OX=9606 GN=FKBP8 PE=1	FKBP8	0.866	0.123818
Q14320	Protein FAM50A OS=Homo sapiens OX=9606 GN=FAM50A PE=1 SV=2	FAM50A	0.892	0.582985
Q14331	Protein FRG1 OS=Homo sapiens OX=9606 GN=FRG1 PE=1 SV=1	FRG1	0.892	0.297822

Q14344	Guanine nucleotide-binding protein subunit alpha-13 OS=Homo sapiens OX=9606	GNA13	0.972	0.76781
Q14376	UDP-glucose 4-epimerase OS=Homo sapiens OX=9606 GN=GALE PE=1 SV=2	GALE	1.192	0.286784
Q14435	Polypeptide N-acetylgalactosaminyltransferase 3 OS=Homo sapiens OX=9606 GN=GALNT3 PE=1 SV=2	GALNT3	0.984	0.492507
Q14444	Caprin-1 OS=Homo sapiens OX=9606 GN=CAPRIN1 PE=1 SV=2	CAPRIN1	0.921	0.484152
Q14451	Growth factor receptor-bound protein 7 OS=Homo sapiens OX=9606 GN=GRB7 PE=1 SV=2	GRB7	1.011	0.868555
Q14498	RNA-binding protein 39 OS=Homo sapiens OX=9606 GN=RBM39 PE=1 SV=2	RBM39	0.939	0.17638
Q14517	Protocadherin Fat 1 OS=Homo sapiens OX=9606 GN=FAT1 PE=1 SV=2	FAT1	1.035	0.489662
Q14534	Squalene monooxygenase OS=Homo sapiens OX=9606 GN=SQLE PE=1 SV=3	SQLE	1.063	0.558573
Q14554	Protein disulfide-isomerase A5 OS=Homo sapiens OX=9606 GN=PDIA5 PE=1 SV=1	PDIA5	1.056	0.234144
Q14562	ATP-dependent RNA helicase DHX8 OS=Homo sapiens OX=9606 GN=DHX8 PE=1 SV=1	DHX8	0.984	0.851015
Q14563	Semaphorin-3A OS=Homo sapiens OX=9606 GN=SEMA3A PE=1 SV=1	SEMA3A	0.92	0.415462
Q14566	DNA replication licensing factor MCM6 OS=Homo sapiens OX=9606 GN=MCM6 PE=1	MCM6	0.825	0.146098
Q14571	Inositol 1,4,5-trisphosphate receptor type 2 OS=Homo sapiens OX=9606 GN=ITPR2 PE=1 SV=2	ITPR2	1.204	
Q14573	Inositol 1,4,5-trisphosphate receptor type 3 OS=Homo sapiens OX=9606 GN=ITPR3 PE=1 SV=2	ITPR3	1.063	0.223088
Q14574	Desmocollin-3 OS=Homo sapiens OX=9606 GN=DSC3 PE=1 SV=3	DSC3	0.974	0.748743
Q14592	Zinc finger protein 460 OS=Homo sapiens OX=9606 GN=ZNF460 PE=1 SV=2	ZNF460	1.035	0.77097
Q14651	Plastin-1 OS=Homo sapiens OX=9606 GN=PLS1 PE=1 SV=2	PLS1	1.115	0.472813
Q14671	Pumilio homolog 1 OS=Homo sapiens OX=9606 GN=PUM1 PE=1 SV=3	PUM1	1.079	0.396347

Q14676	Mediator of DNA damage checkpoint protein 1 OS=Homo sapiens OX=9606 GN=MDC1 PE=1 SV=3	MDC1	0.986	0.781408
Q14677	Clathrin interactor 1 OS=Homo sapiens OX=9606 GN=CLINT1 PE=1 SV=1	CLINT1	1.027	0.707865
Q14683	Structural maintenance of chromosomes protein 1A OS=Homo sapiens OX=9606 GN=SMC1A PE=1 SV=2	SMC1A	0.897	0.043986
Q14684	Ribosomal RNA processing protein 1 homolog B OS=Homo sapiens OX=9606 GN=RRP1B	RRP1B	1.066	0.48505
Q14690	Protein RRP5 homolog OS=Homo sapiens OX=9606 GN=PDCD11 PE=1 SV=3	PDCD11	0.969	0.756248
Q14692	Ribosome biogenesis protein BMS1 homolog OS=Homo sapiens OX=9606 GN=BMS1	BMS1	0.994	0.903358
Q14694	Ubiquitin carboxyl-terminal hydrolase 10 OS=Homo sapiens OX=9606 GN=USP10 PE=1 SV=2	USP10	1.061	0.396251
Q14696	LRP chaperone MESD OS=Homo sapiens OX=9606 GN=MESD PE=1 SV=2	MESD	1.01	0.849142
Q14697	Neutral alpha-glucosidase AB OS=Homo sapiens OX=9606 GN=GANAB PE=1 SV=3	GANAB	1.019	0.517327
Q14728	Major facilitator superfamily domain-containing protein 10 OS=Homo sapiens OX=9606 GN=MFSD10 PE=1 SV=1	MFSD10	1.103	0.190354
Q14738	Serine/threonine-protein phosphatase 2A 56 kDa regulatory subunit delta isoform OS=Homo sapiens OX=9606 GN=PPP2R5D PE=1 SV=1	PPP2R5D	1.085	0.269471
Q14739	Delta(14)-sterol reductase LBR OS=Homo sapiens OX=9606 GN=LBR PE=1 SV=2	LBR	0.899	0.136097
Q14746	Conserved oligomeric Golgi complex subunit 2 OS=Homo sapiens OX=9606 GN=COG2 PE=1 SV=1	COG2	1.023	0.853807
Q14789	Golgin subfamily B member 1 OS=Homo sapiens OX=9606 GN=GOLGB1 PE=1 SV=2	GOLGB1	0.982	0.58358
Q14802	FXYD domain-containing ion transport regulator 3 OS=Homo sapiens OX=9606 GN=FXYD3 PE=1 SV=1	FXYD3	1.011	0.879257

Q14807	Kinesin-like protein KIF22 OS=Homo sapiens OX=9606 GN=KIF22 PE=1 SV=5	KIF22	0.984	0.880647
Q14839	Chromodomain-helicase-DNA-binding protein 4 OS=Homo sapiens OX=9606 GN=CHD4	CHD4	0.927	0.246678
Q14847	LIM and SH3 domain protein 1 OS=Homo sapiens OX=9606 GN=LASP1 PE=1 SV=2	LASP1	1.124	0.458631
Q14849	StAR-related lipid transfer protein 3 OS=Homo sapiens OX=9606 GN=STARD3 PE=1 SV=2	STARD3	1.051	0.337794
Q14919	Dr1-associated corepressor OS=Homo sapiens OX=9606 GN=DRAP1 PE=1 SV=3	DRAP1	0.739	0.068752
Q14956	Transmembrane glycoprotein NMB OS=Homo sapiens OX=9606 GN=GPNMB PE=1	GPNMB	1.758	0.007767
Q14966	Zinc finger protein 638 OS=Homo sapiens OX=9606 GN=ZNF638 PE=1 SV=2	ZNF638	0.935	0.431208
Q14974	Importin subunit beta-1 OS=Homo sapiens OX=9606 GN=KPNB1 PE=1 SV=2	KPNB1	1.083	0.208942
Q14978	Nucleolar and coiled-body phosphoprotein 1 OS=Homo sapiens OX=9606 GN=NOLC1	NOLC1	0.759	0.171916
Q14980	Nuclear mitotic apparatus protein 1 OS=Homo sapiens OX=9606 GN=NUMA1 PE=1 SV=2	NUMA1	0.976	0.473237
Q14997	Proteasome activator complex subunit 4 OS=Homo sapiens OX=9606 GN=PSME4 PE=1	PSME4	0.79	0.072326
Q14BN4	Sarcolemmal membrane-associated protein OS=Homo sapiens OX=9606 GN=SLMAP PE=1	SLMAP	1.008	0.925996
Q15003	Condensin complex subunit 2 OS=Homo sapiens OX=9606 GN=NCAPH PE=1 SV=3	NCAPH	1.099	0.181505
Q15005	Signal peptidase complex subunit 2 OS=Homo sapiens OX=9606 GN=SPCS2 PE=1 SV=3	SPCS2	0.987	0.796562
Q15006	ER membrane protein complex subunit 2 OS=Homo sapiens OX=9606 GN=EMC2 PE=1 SV=1	EMC2	1.061	0.131628
Q15007	Pre-mRNA-splicing regulator WTAP OS=Homo sapiens OX=9606 GN=WTAP PE=1 SV=2	WTAP	0.961	0.373574
Q15008	26S proteasome non-ATPase regulatory subunit 6 OS=Homo sapiens OX=9606 GN=PSMD6 PE=1 SV=1	PSMD6	1.081	0.099857

Q15012	Lysosomal-associated transmembrane protein 4A OS=Homo sapiens OX=9606 GN=LAPTM4A PE=1 SV=1	LAPTM4A	0.527	
Q15014	Mortality factor 4-like protein 2 OS=Homo sapiens OX=9606 GN=MORF4L2 PE=1 SV=1	MORF4L2	0.928	0.532379
Q15019	Septin-2 OS=Homo sapiens OX=9606 GN=SEPTIN2 PE=1 SV=1	SEPTIN2	1.108	0.288303
Q15020	Squamous cell carcinoma antigen recognized by T-cells 3 OS=Homo sapiens OX=9606 GN=SART3 PE=1 SV=1	SART3	0.909	0.28837
Q15021	Condensin complex subunit 1 OS=Homo sapiens OX=9606 GN=NCAPD2 PE=1 SV=3	NCAPD2	1.004	0.964265
Q15022	Polycomb protein SUZ12 OS=Homo sapiens OX=9606 GN=SUZ12 PE=1 SV=3	SUZ12	0.889	0.002096
Q15024	Exosome complex component RRP42 OS=Homo sapiens OX=9606 GN=EXOSC7 PE=1 SV=3	EXOSC7	0.889	0.24725
Q15029	116 kDa U5 small nuclear ribonucleoprotein component OS=Homo sapiens OX=9606 GN=EFTUD2 PE=1 SV=1	EFTUD2	0.995	0.817953
Q15031	Probable leucine--tRNA ligase, mitochondrial OS=Homo sapiens OX=9606 GN=LARS2 PE=1	LARS2	1.005	0.940205
Q15041	ADP-ribosylation factor-like protein 6-interacting protein 1 OS=Homo sapiens OX=9606 GN=ARL6IP1 PE=1 SV=2	ARL6IP1	1.069	0.652804
Q15043	Metal cation symporter ZIP14 OS=Homo sapiens OX=9606 GN=SLC39A14 PE=1 SV=3	SLC39A14	1.055	0.463807
Q15046	Lysine--tRNA ligase OS=Homo sapiens OX=9606 GN=KARS1 PE=1 SV=3	KARS1	0.961	0.624723
Q15050	Ribosome biogenesis regulatory protein homolog OS=Homo sapiens OX=9606 GN=RRS1	RRS1	0.927	0.203983
Q15056	Eukaryotic translation initiation factor 4H OS=Homo sapiens OX=9606 GN=EIF4H PE=1 SV=5	EIF4H	0.994	0.887012
Q15061	WD repeat-containing protein 43 OS=Homo sapiens OX=9606 GN=WDR43 PE=1 SV=3	WDR43	0.979	0.787969
Q15067	Peroxisomal acyl-coenzyme A oxidase 1 OS=Homo sapiens OX=9606 GN=ACOX1 PE=1	ACOX1	1	0.947449

Q15070	Mitochondrial inner membrane protein OXA1L OS=Homo sapiens OX=9606 GN=OXA1L PE=1	OXA1L	0.909	0.362087
Q15075	Early endosome antigen 1 OS=Homo sapiens OX=9606 GN=EEA1 PE=1 SV=2	EEA1	0.889	0.025546
Q15084	Protein disulfide-isomerase A6 OS=Homo sapiens OX=9606 GN=PDIA6 PE=1 SV=1	PDIA6	1.088	0.045509
Q15102	Platelet-activating factor acetylhydrolase IB subunit alpha1 OS=Homo sapiens OX=9606 GN=PAFAH1B3 PE=1 SV=1	PAFAH1B 3	1.072	0.238607
Q15120	[Pyruvate dehydrogenase (acetyl-transferring)] kinase isozyme 3, mitochondrial OS=Homo sapiens OX=9606 GN=PDK3 PE=1 SV=1	PDK3	0.898	0.444506
Q15121	Astrocytic phosphoprotein PEA-15 OS=Homo sapiens OX=9606 GN=PEA15 PE=1 SV=2	PEA15	1.161	0.344892
Q15125	3-beta-hydroxysteroid-Delta(8),Delta(7)-isomerase OS=Homo sapiens OX=9606 GN=EBP PE=1 SV=3	EBP	0.575	0.070335
Q15149	Plectin OS=Homo sapiens OX=9606 GN=PLEC PE=1 SV=3	PLEC	1.094	0.124009
Q15154	Pericentriolar material 1 protein OS=Homo sapiens OX=9606 GN=PCM1 PE=1 SV=5	PCM1	1.012	0.861236
Q15155	Nodal modulator 1 OS=Homo sapiens OX=9606 GN=NOMO1 PE=1 SV=5	NOMO1	1.087	0.120953
Q15165	Serum paraoxonase/arylesterase 2 OS=Homo sapiens OX=9606 GN=PON2 PE=1 SV=4	PON2	0.978	0.804766
Q15181	Inorganic pyrophosphatase OS=Homo sapiens OX=9606 GN=PPA1 PE=1 SV=2	PPA1	1.025	0.813958
Q15185	Prostaglandin E synthase 3 OS=Homo sapiens OX=9606 GN=PTGES3 PE=1 SV=1	PTGES3	1.049	0.656694
Q15223	Nectin-1 OS=Homo sapiens OX=9606 GN=NECTIN1 PE=1 SV=3	NECTIN1	0.883	0.306341
Q15233	Non-POU domain-containing octamer-binding protein OS=Homo sapiens OX=9606 GN=NONO PE=1 SV=4	NONO	0.986	0.743565
Q15257	Serine/threonine-protein phosphatase 2A activator OS=Homo sapiens OX=9606 GN=PTPA PE=1 SV=3	PTPA	1.27	0.257246

Q15262	Receptor-type tyrosine-protein phosphatase kappa OS=Homo sapiens OX=9606 GN=PTPRK PE=1 SV=2	PTPRK	1.033	0.697871
Q15269	Periodic tryptophan protein 2 homolog OS=Homo sapiens OX=9606 GN=PWP2 PE=2 SV=2	PWP2	0.947	0.626828
Q15286	Ras-related protein Rab-35 OS=Homo sapiens OX=9606 GN=RAB35 PE=1 SV=1	RAB35	0.953	0.695929
Q15287	RNA-binding protein with serine-rich domain 1 OS=Homo sapiens OX=9606 GN=RNPS1 PE=1	RNPS1	0.905	0.411494
Q15291	Retinoblastoma-binding protein 5 OS=Homo sapiens OX=9606 GN=RBBP5 PE=1 SV=2	RBBP5	0.876	0.037338
Q15293	Reticulocalbin-1 OS=Homo sapiens OX=9606 GN=RCN1	RCN1	1.067	0.2111
Q15363	Transmembrane emp24 domain-containing protein 2 OS=Homo sapiens OX=9606 GN=TMED2 PE=1 SV=1	TMED2	1.128	0.299516
Q15365	Poly(rC)-binding protein 1 OS=Homo sapiens OX=9606 GN=PCBP1 PE=1 SV=2	PCBP1	0.916	0.299253
Q15366	Poly(rC)-binding protein 2 OS=Homo sapiens OX=9606 GN=PCBP2 PE=1 SV=1	PCBP2	0.98	0.782125
Q15369	Elongin-C OS=Homo sapiens OX=9606 GN=ELOC PE=1 SV=1	ELOC	0.895	0.209806
Q15370	Elongin-B OS=Homo sapiens OX=9606 GN=ELOB PE=1 SV=1	ELOB	1.002	0.957174
Q15382	GTP-binding protein Rheb OS=Homo sapiens OX=9606 GN=RHEB PE=1 SV=1	RHEB	1.16	0.304554
Q15388	Mitochondrial import receptor subunit TOM20 homolog OS=Homo sapiens OX=9606 GN=TOMM20 PE=1 SV=1	TOMM20	1.159	0.000905
Q15392	Delta(24)-sterol reductase OS=Homo sapiens OX=9606 GN=DHCR24 PE=1 SV=2	DHCR24	1.025	0.7795
Q15393	Splicing factor 3B subunit 3 OS=Homo sapiens OX=9606 GN=SF3B3 PE=1 SV=4	SF3B3	0.982	0.475898
Q15397	Pumilio homolog 3 OS=Homo sapiens OX=9606 GN=PUM3 PE=1 SV=3	PUM3	0.972	0.807681
Q15404	Ras suppressor protein 1 OS=Homo sapiens OX=9606 GN=RSU1 PE=1 SV=3	RSU1	1.405	0.035112
Q15417	Calponin-3 OS=Homo sapiens OX=9606 GN=CNN3 PE=1 SV=1	CNN3	1.002	0.951398

Q15424	Scaffold attachment factor B1 OS=Homo sapiens OX=9606 GN=SAFB PE=1 SV=4	SAFB	1.033	0.482084
Q15427	Splicing factor 3B subunit 4 OS=Homo sapiens OX=9606 GN=SF3B4 PE=1 SV=1	SF3B4	0.947	0.34068
Q15428	Splicing factor 3A subunit 2 OS=Homo sapiens OX=9606 GN=SF3A2 PE=1 SV=2	SF3A2	0.919	0.541304
Q15435	Protein phosphatase 1 regulatory subunit 7 OS=Homo sapiens OX=9606 GN=PPP1R7 PE=1	PPP1R7	1.025	
Q15437	Protein transport protein Sec23B OS=Homo sapiens OX=9606 GN=SEC23B PE=1 SV=2	SEC23B	1.086	0.36352
Q15459	Splicing factor 3A subunit 1 OS=Homo sapiens OX=9606 GN=SF3A1 PE=1 SV=1	SF3A1	0.927	0.021097
Q15527	Surfeit locus protein 2 OS=Homo sapiens OX=9606 GN=SURF2 PE=1 SV=3	SURF2	0.931	0.583205
Q15555	Microtubule-associated protein RP/EB family member 2 OS=Homo sapiens OX=9606 GN=MAPRE2 PE=1 SV=1	MAPRE2	1.126	
Q15582	Transforming growth factor-beta-induced protein ig-h3 OS=Homo sapiens OX=9606 GN=TGFBI PE=1 SV=1	TGFBI	1.071	0.540563
Q15599	Na(+)/H(+) exchange regulatory cofactor NHE-RF2 OS=Homo sapiens OX=9606 GN=SLC9A3R2 PE=1 SV=2	SLC9A3R2	1.157	0.366983
Q15629	Translocating chain-associated membrane protein 1 OS=Homo sapiens OX=9606 GN=TRAM1 PE=1 SV=3	TRAM1	1.077	0.382209
Q15631	Translin OS=Homo sapiens OX=9606 GN=TSN PE=1 SV=1	TSN	0.935	0.439532
Q15637	Splicing factor 1 OS=Homo sapiens OX=9606 GN=SF1 PE=1	SF1	0.944	0.424651
Q15643	Thyroid receptor-interacting protein 11 OS=Homo sapiens OX=9606 GN=TRIP11 PE=1	TRIP11	1.008	0.893322
Q15648	Mediator of RNA polymerase II transcription subunit 1 OS=Homo sapiens OX=9606 GN=MED1 PE=1 SV=4	MED1	0.921	0.15911
Q15654	Thyroid receptor-interacting protein 6 OS=Homo sapiens OX=9606 GN=TRIP6 PE=1 SV=3	TRIP6	1.068	0.225473

Q15691	Microtubule-associated protein RP/EB family member 1 OS=Homo sapiens OX=9606 GN=MAPRE1 PE=1 SV=3	MAPRE1	1.096	0.440168
Q15717	ELAV-like protein 1 OS=Homo sapiens OX=9606 GN=ELAVL1 PE=1 SV=2	ELAVL1	1.002	0.988854
Q15738	Sterol-4-alpha-carboxylate 3-dehydrogenase, decarboxylating OS=Homo sapiens OX=9606 GN=NSDHL PE=1 SV=2	NSDHL	0.968	0.682029
Q15742	NGFI-A-binding protein 2 OS=Homo sapiens OX=9606 GN=NAB2 PE=1 SV=1	NAB2	1.21	0.148373
Q15758	Neutral amino acid transporter B(0) OS=Homo sapiens OX=9606 GN=SLC1A5 PE=1 SV=2	SLC1A5	0.991	0.882076
Q15785	Mitochondrial import receptor subunit TOM34 OS=Homo sapiens OX=9606 GN=TOMM34 PE=1 SV=2	TOMM34	1.132	0.226452
Q15800	Methylsterol monooxygenase 1 OS=Homo sapiens OX=9606 GN=MSMO1 PE=1 SV=1	MSMO1	0.995	0.951503
Q15819	Ubiquitin-conjugating enzyme E2 variant 2 OS=Homo sapiens OX=9606 GN=UBE2V2 PE=1 SV=4	UBE2V2	0.983	0.829256
Q15833	Syntaxin-binding protein 2 OS=Homo sapiens OX=9606 GN=STXBP2 PE=1 SV=2	STXBP2	0.912	0.229367
Q15836	Vesicle-associated membrane protein 3 OS=Homo sapiens OX=9606 GN=VAMP3 PE=1	VAMP3	0.936	0.296459
Q15843	NEDD8 OS=Homo sapiens OX=9606 GN=NEDD8 PE=1	NEDD8	0.858	0.261564
Q15904	V-type proton ATPase subunit S1 OS=Homo sapiens OX=9606 GN=ATP6AP1 PE=1 SV=2	ATP6AP1	0.929	0.051559
Q15906	Vacuolar protein sorting-associated protein 72 homolog OS=Homo sapiens OX=9606 GN=VPS72 PE=1 SV=1	VPS72	0.977	0.435916
Q15907	Ras-related protein Rab-11B OS=Homo sapiens OX=9606 GN=RAB11B PE=1 SV=4	RAB11B	0.953	0.305802
Q15910	Histone-lysine N-methyltransferase EZH2 OS=Homo sapiens OX=9606 GN=EZH2 PE=1 SV=2	EZH2	0.99	0.9148
Q15942	Zyxin OS=Homo sapiens OX=9606 GN=ZYX PE=1 SV=1	ZYX	1.227	0.051668

Q16181	Septin-7 OS=Homo sapiens OX=9606 GN=SEPTIN7 PE=1 SV=2	SEPTIN7	1.03	0.522447
Q16186	Proteasomal ubiquitin receptor ADRM1 OS=Homo sapiens OX=9606 GN=ADRM1 PE=1	ADRM1	1.064	0.049447
Q16204	Coiled-coil domain-containing protein 6 OS=Homo sapiens OX=9606 GN=CCDC6 PE=1	CCDC6	0.986	0.858092
Q16222	UDP-N-acetylhexosamine pyrophosphorylase OS=Homo sapiens OX=9606 GN=UAP1 PE=1 SV=3	UAP1	1.272	0.152292
Q16363	Laminin subunit alpha-4 OS=Homo sapiens OX=9606 GN=LAMA4 PE=1 SV=4	LAMA4	1.105	0.247285
Q16401	26S proteasome non-ATPase regulatory subunit 5 OS=Homo sapiens OX=9606 GN=PSMD5 PE=1 SV=3	PSMD5	1.036	0.720523
Q16513	Serine/threonine-protein kinase N2 OS=Homo sapiens OX=9606 GN=PKN2 PE=1 SV=1	PKN2	0.931	0.498645
Q16527	Cysteine and glycine-rich protein 2 OS=Homo sapiens OX=9606 GN=CSRP2 PE=1 SV=3	CSRP2	0.969	0.749601
Q16531	DNA damage-binding protein 1 OS=Homo sapiens OX=9606 GN=DDB1 PE=1 SV=1	DDB1	0.933	0.143662
Q16537	Serine/threonine-protein phosphatase 2A 56 kDa regulatory subunit epsilon isoform OS=Homo sapiens OX=9606 GN=PPP2R5E PE=1 SV=1	PPP2R5E	1.309	0.382121
Q16540	39S ribosomal protein L23, mitochondrial OS=Homo sapiens OX=9606 GN=MRPL23 PE=1 SV=1	MRPL23	0.929	0.32196
Q16543	Hsp90 co-chaperone Cdc37 OS=Homo sapiens OX=9606 GN=CDC37 PE=1 SV=1	CDC37	1.032	0.77301
Q16555	Dihydropyrimidinase-related protein 2 OS=Homo sapiens OX=9606 GN=DPYSL2 PE=1	DPYSL2	1.175	0.144682
Q16563	Synaptophysin-like protein 1 OS=Homo sapiens OX=9606 GN=SYPL1 PE=1 SV=1	SYPL1	0.89	0.208191
Q16576	Histone-binding protein RBBP7 OS=Homo sapiens OX=9606 GN=RBBP7 PE=1 SV=1	RBBP7	0.843	0.020322
Q16595	Frataxin, mitochondrial OS=Homo sapiens OX=9606 GN=FXN PE=1 SV=2	FXN	0.899	0.09021

Q16611	Bcl-2 homologous antagonist/killer OS=Homo sapiens OX=9606 GN=BAK1 PE=1 SV=1	BAK1	0.991	
Q16625	Occludin OS=Homo sapiens OX=9606 GN=OCLN PE=1 SV=1	OCLN	0.978	0.977257
Q16629	Serine/arginine-rich splicing factor 7 OS=Homo sapiens OX=9606 GN=SRSF7 PE=1 SV=1	SRSF7	0.93	0.011961
Q16630	Cleavage and polyadenylation specificity factor subunit 6 OS=Homo sapiens OX=9606 GN=CPSF6 PE=1 SV=2	CPSF6	0.934	0.084067
Q16637	Survival motor neuron protein OS=Homo sapiens OX=9606 GN=SMN1 PE=1 SV=1	SMN1	1.147	0.180284
Q16643	Drebrin OS=Homo sapiens OX=9606 GN=DBN1 PE=1 SV=4	DBN1	1.064	0.251044
Q16658	Fascin OS=Homo sapiens OX=9606 GN=FSCN1 PE=1	FSCN1	1.026	0.974205
Q16666	Gamma-interferon-inducible protein 16 OS=Homo sapiens OX=9606 GN=IFI16 PE=1 SV=3	IFI16	0.968	0.778719
Q16698	2,4-dienoyl-CoA reductase [(3E)-enoyl-CoA-producing], mitochondrial OS=Homo sapiens OX=9606 GN=DECR1 PE=1	DECR1	0.996	0.948268
Q16706	Alpha-mannosidase 2 OS=Homo sapiens OX=9606 GN=MAN2A1 PE=1 SV=2	MAN2A1	1.104	0.208739
Q16718	NADH dehydrogenase [ubiquinone] 1 alpha subcomplex subunit 5 OS=Homo sapiens OX=9606 GN=NDUFA5 PE=1	NDUFA5	1.003	0.961843
Q16739	Ceramide glucosyltransferase OS=Homo sapiens OX=9606 GN=UGCG PE=1 SV=1	UGCG	0.001	0.001
Q16740	ATP-dependent Clp protease proteolytic subunit, mitochondrial OS=Homo sapiens OX=9606 GN=CLPP PE=1 SV=1	CLPP	0.89	0.528182
Q16762	Thiosulfate sulfurtransferase OS=Homo sapiens OX=9606 GN=TST PE=1 SV=4	TST	0.987	0.843277
Q16763	Ubiquitin-conjugating enzyme E2 S OS=Homo sapiens OX=9606 GN=UBE2S PE=1 SV=2	UBE2S	1.041	0.651433
Q16774	Guanylate kinase OS=Homo sapiens OX=9606 GN=GUK1	GUK1	1.008	0.950782
Q16787	Laminin subunit alpha-3 OS=Homo sapiens OX=9606 GN=LAMA3 PE=1 SV=2	LAMA3	1.051	0.294479

Q16795	NADH dehydrogenase [ubiquinone] 1 alpha subcomplex subunit 9, mitochondrial OS=Homo sapiens OX=9606 GN=NDUFA9 PE=1 SV=2	NDUFA9	1.005	0.997941
Q16798	NADP-dependent malic enzyme, mitochondrial OS=Homo sapiens OX=9606 GN=ME3 PE=2 SV=2	ME3	1.065	0.601961
Q16799	Reticulon-1 OS=Homo sapiens OX=9606 GN=RTN1 PE=1 SV=1	RTN1	1.17	0.237935
Q16822	Phosphoenolpyruvate carboxykinase [GTP], mitochondrial OS=Homo sapiens OX=9606 GN=PCK2 PE=1 SV=4	PCK2	1.026	0.891089
Q16831	Uridine phosphorylase 1 OS=Homo sapiens OX=9606 GN=UPP1 PE=1 SV=1	UPP1	0.865	0.364291
Q16836	Hydroxyacyl-coenzyme A dehydrogenase, mitochondrial OS=Homo sapiens OX=9606 GN=HADH PE=1 SV=3	HADH	0.987	0.690611
Q16850	Lanosterol 14-alpha demethylase OS=Homo sapiens OX=9606 GN=CYP51A1 PE=1 SV=4	CYP51A1	1.049	0.675514
Q16851	UTP--glucose-1-phosphate uridylyltransferase OS=Homo sapiens OX=9606 GN=UGP2 PE=1 SV=5	UGP2	1.103	0.357625
Q16864	V-type proton ATPase subunit F OS=Homo sapiens OX=9606 GN=ATP6V1F PE=1 SV=2	ATP6V1F	1.008	0.863672
Q16880	2-hydroxyacylsphingosine 1-beta-galactosyltransferase OS=Homo sapiens OX=9606 GN=UGT8 PE=1 SV=2	UGT8	1.101	0.659759
Q16881	Thioredoxin reductase 1, cytoplasmic OS=Homo sapiens OX=9606 GN=TXNRD1 PE=1 SV=3	TXNRD1	1.036	0.71455
Q16890	Tumor protein D53 OS=Homo sapiens OX=9606 GN=TPD52L1 PE=1 SV=1	TPD52L1	1.049	0.532563
Q16891	MICOS complex subunit MIC60 OS=Homo sapiens OX=9606 GN=IMMT PE=1 SV=1	IMMT	0.974	0.47153
Q17RY6	Lymphocyte antigen 6K OS=Homo sapiens OX=9606 GN=LY6K PE=1 SV=2	LY6K	0.997	0.934283
Q1ED39	Lysine-rich nucleolar protein 1 OS=Homo sapiens OX=9606 GN=KNOP1 PE=1 SV=1	KNOP1	0.897	0.426116

Q1KMD3	Heterogeneous nuclear ribonucleoprotein U-like protein 2 OS=Homo sapiens OX=9606 GN=HNRNPUL2 PE=1 SV=1	HNRNPUL 2	1.01	0.690709
Q24JP5	Transmembrane protein 132A OS=Homo sapiens OX=9606 GN=TMEM132A PE=1 SV=1	TMEM132 A	0.982	0.838098
Q27J81	Inverted formin-2 OS=Homo sapiens OX=9606 GN=INF2 PE=1	INF2	1.028	0.720007
Q29RF7	Sister chromatid cohesion protein PDS5 homolog A OS=Homo sapiens OX=9606 GN=PDS5A	PDS5A	0.881	0.192407
Q2KHR3	Glutamine and serine-rich protein 1 OS=Homo sapiens OX=9606 GN=QSER1 PE=1 SV=3	QSER1	0.639	
Q2NL82	Pre-rRNA-processing protein TSR1 homolog OS=Homo sapiens OX=9606 GN=TSR1 PE=1 SV=1	TSR1	0.769	0.149772
Q2PZI1	Probable C-mannosyltransferase DPY19L1 OS=Homo sapiens OX=9606 GN=DPY19L1 PE=2 SV=1	DPY19L1	1.167	0.315408
Q2TAL8	Transcriptional regulator QRICH1 OS=Homo sapiens OX=9606 GN=QRICH1 PE=1 SV=1	QRICH1	0.845	0.234204
Q2TAY7	WD40 repeat-containing protein SMU1 OS=Homo sapiens OX=9606 GN=SMU1 PE=1 SV=2	SMU1	0.941	0.297503
Q32MZ4	Leucine-rich repeat flightless-interacting protein 1 OS=Homo sapiens OX=9606 GN=LRRFIP1 PE=1 SV=2	LRRFIP1	1.102	0.173833
Q32P28	Prolyl 3-hydroxylase 1 OS=Homo sapiens OX=9606 GN=P3H1 PE=1 SV=2	P3H1	1.148	0.401776
Q3B726	DNA-directed RNA polymerase I subunit RPA43 OS=Homo sapiens OX=9606 GN=POLR1F PE=1 SV=1	POLR1F	1.093	0.144172
Q3KQU3	MAP7 domain-containing protein 1 OS=Homo sapiens OX=9606 GN=MAP7D1 PE=1 SV=1	MAP7D1	1.063	
Q3MHD2	Protein LSM12 homolog OS=Homo sapiens OX=9606 GN=LSM12 PE=1 SV=2	LSM12	1.029	0.29821
Q3SXM5	Inactive hydroxysteroid dehydrogenase-like protein 1 OS=Homo sapiens OX=9606 GN=HSDL1 PE=1 SV=3	HSDL1	0.893	0.592484
Q3ZCM7	Tubulin beta-8 chain OS=Homo sapiens OX=9606 GN=TUBB8 PE=1 SV=2	TUBB8	0.88	0.3905

Q3ZCQ8	Mitochondrial import inner membrane translocase subunit TIM50 OS=Homo sapiens OX=9606 GN=TIMM50 PE=1	TIMM50	1.041	0.574562
Q49A26	Putative oxidoreductase GLYR1 OS=Homo sapiens OX=9606 GN=GLYR1 PE=1 SV=4	GLYR1	1.101	0.229683
Q4G0J3	La-related protein 7 OS=Homo sapiens OX=9606 GN=LARP7 PE=1 SV=1	LARP7	1	0.970772
Q4G0N4	NAD kinase 2, mitochondrial OS=Homo sapiens OX=9606 GN=NADK2 PE=1 SV=2	NADK2	1.056	0.125924
Q4G148	Glucoside xylosyltransferase 1 OS=Homo sapiens OX=9606 GN=GXYLT1 PE=1 SV=2	GXYLT1	1.227	0.008417
Q4KMQ2	Anoctamin-6 OS=Homo sapiens OX=9606 GN=ANO6 PE=1 SV=2	ANO6	1.112	0.422225
Q4KWH8	1-phosphatidylinositol 4,5-bisphosphate phosphodiesterase eta-1 OS=Homo sapiens OX=9606 GN=PLCH1 PE=1 SV=1	PLCH1	1.088	
Q4LE39	AT-rich interactive domain-containing protein 4B OS=Homo sapiens OX=9606 GN=ARID4B PE=1 SV=2	ARID4B	0.952	0.569964
Q4VC31	Protein MIX23 OS=Homo sapiens OX=9606 GN=MIX23 PE=1	MIX23	1.003	0.979091
Q52LJ0	Protein FAM98B OS=Homo sapiens OX=9606 GN=FAM98B PE=1 SV=2	FAM98B	1.009	0.966646
Q53EP0	Fibronectin type III domain-containing protein 3B OS=Homo sapiens OX=9606 GN=FNDC3B PE=1 SV=2	FNDC3B	0.934	0.00281
Q53EU6	Glycerol-3-phosphate acyltransferase 3 OS=Homo sapiens OX=9606 GN=GPAT3	GPAT3	1.009	0.778545
Q53F19	Nuclear cap-binding protein subunit 3 OS=Homo sapiens OX=9606 GN=NCBP3 PE=1	NCBP3	1.076	0.424763
Q53FV1	ORM1-like protein 2 OS=Homo sapiens OX=9606 GN=ORMDL2 PE=1 SV=2	ORMDL2	1.117	0.591416
Q53GA4	Pleckstrin homology-like domain family A member 2 OS=Homo sapiens OX=9606 GN=PHLDA2 PE=1 SV=2	PHLDA2	0.831	0.061464
Q53GQ0	Very-long-chain 3-oxoacyl-CoA reductase OS=Homo sapiens OX=9606 GN=HSD17B12 PE=1 SV=2	HSD17B12	0.971	0.168487

Q53GS9	U4/U6.U5 tri-snRNP-associated protein 2 OS=Homo sapiens OX=9606 GN=USP39 PE=1 SV=2	USP39	0.834	0.20251
Q53H12	Acylglycerol kinase, mitochondrial OS=Homo sapiens OX=9606 GN=AGK PE=1 SV=2	AGK	1.083	0.047266
Q53H82	Endoribonuclease LACTB2 OS=Homo sapiens OX=9606 GN=LACTB2 PE=1 SV=2	LACTB2	1.073	0.454524
Q53H96	Pyrroline-5-carboxylate reductase 3 OS=Homo sapiens OX=9606 GN=PYCR3 PE=1 SV=3	PYCR3	1.349	
Q53HL2	Borealin OS=Homo sapiens OX=9606 GN=CDCA8 PE=1	CDCA8	1.148	0.135192
Q53S33	BolA-like protein 3 OS=Homo sapiens OX=9606 GN=BOLA3 PE=1 SV=1	BOLA3	0.998	
Q53TN4	Plasma membrane ascorbate-dependent reductase CYBRD1 OS=Homo sapiens OX=9606 GN=CYBRD1 PE=1 SV=1	CYBRD1	0.823	0.471907
Q562R1	Beta-actin-like protein 2 OS=Homo sapiens OX=9606 GN=ACTBL2 PE=1 SV=2	ACTBL2	0.677	
Q56VL3	OCIA domain-containing protein 2 OS=Homo sapiens OX=9606 GN=OCIAD2 PE=1 SV=1	OCIAD2	0.995	0.957984
Q58FF8	Putative heat shock protein HSP 90-beta 2 OS=Homo sapiens OX=9606 GN=HSP90AB2P PE=1	HSP90AB2P	0.981	0.811387
Q5BJD5	Transmembrane protein 41B OS=Homo sapiens OX=9606 GN=TMEM41B PE=1 SV=1	TMEM41B	1.036	0.581604
Q5BJF2	Sigma intracellular receptor 2 OS=Homo sapiens OX=9606 GN=TMEM97 PE=1 SV=1	TMEM97	1.007	0.946751
Q5BJH7	Protein YIF1B OS=Homo sapiens OX=9606 GN=YIF1B PE=1 SV=1	YIF1B	0.954	0.650953
Q5I7T1	Putative Dol-P-Glc:Glc(2)Man(9)GlcNAc(2)-PP-Dol alpha-1,2-glucosyltransferase OS=Homo sapiens OX=9606 GN=ALG10B PE=1 SV=2	ALG10B	0.966	
Q5BKY9	Protein FAM133B OS=Homo sapiens OX=9606 GN=FAM133B PE=1 SV=1	FAM133B	0.999	
Q5BKZ1	DBIRD complex subunit ZNF326 OS=Homo sapiens OX=9606 GN=ZNF326 PE=1 SV=2	ZNF326	0.934	0.164651
Q5C9Z4	Nucleolar MIF4G domain-containing protein 1 OS=Homo sapiens OX=9606 GN=NOM1 PE=1 SV=1	NOM1	0.847	0.302175

Q5EB52	Mesoderm-specific transcript homolog protein OS=Homo sapiens OX=9606 GN=MEST	MEST	0.93	0.65027
Q5EBL8	PDZ domain-containing protein 11 OS=Homo sapiens OX=9606 GN=PDZD11 PE=1 SV=2	PDZD11	1.233	0.031606
Q5H9R7	Serine/threonine-protein phosphatase 6 regulatory subunit 3 OS=Homo sapiens OX=9606 GN=PPP6R3 PE=1 SV=2	PPP6R3	0.996	0.936686
Q5HYI7	Metaxin-3 OS=Homo sapiens OX=9606 GN=MTX3 PE=1 SV=2	MTX3	1.075	
Q5HYI8	Rab-like protein 3 OS=Homo sapiens OX=9606 GN=RABL3 PE=1 SV=1	RABL3	0.97	0.855453
Q5J8M3	ER membrane protein complex subunit 4 OS=Homo sapiens OX=9606 GN=EMC4 PE=1 SV=2	EMC4	1.021	0.668303
Q5JPH6	Probable glutamate--tRNA ligase, mitochondrial OS=Homo sapiens OX=9606 GN=EARS2 PE=1	EARS2	1.12	0.522551
Q5JRA6	Transport and Golgi organization protein 1 homolog OS=Homo sapiens OX=9606 GN=MIA3 PE=1 SV=1	MIA3	1.008	0.857711
Q5JRX3	Presequence protease, mitochondrial OS=Homo sapiens OX=9606 GN=PITRM1 PE=1	PITRM1	1.035	0.467899
Q5JSZ5	Protein PRRC2B OS=Homo sapiens OX=9606 GN=PRRC2B PE=1 SV=2	PRRC2B	0.929	0.416587
Q5JTH9	RRP12-like protein OS=Homo sapiens OX=9606 GN=RRP12 PE=1 SV=2	RRP12	0.983	0.829873
Q5JTJ3	Cytochrome c oxidase assembly factor 6 homolog OS=Homo sapiens OX=9606 GN=COA6	COA6	1.12	0.244337
Q5JTV8	Torsin-1A-interacting protein 1 OS=Homo sapiens OX=9606 GN=TOR1AIP1 PE=1 SV=2	TOR1AIP1	0.961	0.662228
Q5JTZ9	Alanine--tRNA ligase, mitochondrial OS=Homo sapiens OX=9606 GN=AARS2 PE=1	AARS2	0.975	0.826513
Q5JVF3	PCI domain-containing protein 2 OS=Homo sapiens OX=9606 GN=PCID2 PE=1 SV=2	PCID2	0.873	0.479903
Q5K4L6	Solute carrier family 27 member 3 OS=Homo sapiens OX=9606 GN=SLC27A3 PE=1 SV=4	SLC27A3	1.136	0.431708
Q5K651	Sterile alpha motif domain-containing protein 9 OS=Homo sapiens OX=9606 GN=SAMD9 PE=1 SV=1	SAMD9	0.962	0.803239

Q5M775	Cytospin-B OS=Homo sapiens OX=9606 GN=SPECC1 PE=1 SV=1	SPECC1	0.839	0.030502
Q5M9Q1	NKAP-like protein OS=Homo sapiens OX=9606 GN=NKAPL PE=1 SV=3	NKAPL	0.001	0.001
Q5NDL2	EGF domain-specific O-linked N-acetylglucosamine transferase OS=Homo sapiens OX=9606 GN=EOGT PE=1 SV=1	EOGT	0.928	0.178904
Q5PRF9	Protein Smaug homolog 2 OS=Homo sapiens OX=9606 GN=SAMD4B PE=1 SV=1	SAMD4B	0.884	0.480352
Q5QJE6	Deoxynucleotidyltransferase terminal-interacting protein 2 OS=Homo sapiens OX=9606 GN=DNTTIP2 PE=1 SV=2	DNTTIP2	0.982	0.736464
Q5RI15	Cytochrome c oxidase assembly protein COX20, mitochondrial OS=Homo sapiens OX=9606 GN=COX20 PE=1 SV=2	COX20	1.106	0.176251
Q5RKV6	Exosome complex component MTR3 OS=Homo sapiens OX=9606 GN=EXOSC6 PE=1	EXOSC6	0.991	0.881815
Q5SNT2	Transmembrane protein 201 OS=Homo sapiens OX=9606 GN=TMEM201 PE=1 SV=1	TMEM201	1.157	0.070192
Q5SRE5	Nucleoporin NUP188 OS=Homo sapiens OX=9606 GN=NUP188 PE=1 SV=1	NUP188	0.956	0.359225
Q5SSJ5	Heterochromatin protein 1-binding protein 3 OS=Homo sapiens OX=9606 GN=HP1BP3 PE=1 SV=1	HP1BP3	1.034	0.947793
Q5SWX8	Protein odr-4 homolog OS=Homo sapiens OX=9606 GN=ODR4 PE=1 SV=1	ODR4	0.849	0.432689
Q5SY16	Polynucleotide 5'-hydroxyl-kinase NOL9 OS=Homo sapiens OX=9606 GN=NOL9 PE=1 SV=1	NOL9	1.134	0.298563
Q5T160	Probable arginine--tRNA ligase, mitochondrial OS=Homo sapiens OX=9606 GN=RARS2 PE=1	RARS2	0.985	0.859737
Q5T200	Zinc finger CCCH domain-containing protein 13 OS=Homo sapiens OX=9606 GN=ZC3H13 PE=1 SV=1	ZC3H13	1.12	0.009334
Q5T280	Putative methyltransferase C9orf114 OS=Homo sapiens OX=9606 GN=SPOUT1 PE=1	SPOUT1	0.814	0.121917
Q5T3F8	CSC1-like protein 2 OS=Homo sapiens OX=9606 GN=TMEM63B PE=1 SV=1	TMEM63B	1.022	0.567806

Q5T3I0	G patch domain-containing protein 4 OS=Homo sapiens OX=9606 GN=GPATCH4 PE=1 SV=2	GPATCH4	0.943	0.138445
Q5T440	Putative transferase CAF17, mitochondrial OS=Homo sapiens OX=9606 GN=IBA57 PE=1 SV=1	IBA57	0.992	0.910002
Q5T4S7	E3 ubiquitin-protein ligase UBR4 OS=Homo sapiens OX=9606 GN=UBR4 PE=1 SV=1	UBR4	1.092	0.288784
Q5T5P2	Sickle tail protein homolog OS=Homo sapiens OX=9606 GN=KIAA1217 PE=1 SV=2	KIAA1217	0.963	0.543806
Q5T653	39S ribosomal protein L2, mitochondrial OS=Homo sapiens OX=9606 GN=MRPL2 PE=1	MRPL2	1.043	0.564164
Q5T8D3	Acyl-CoA-binding domain-containing protein 5 OS=Homo sapiens OX=9606 GN=ACBD5 PE=1 SV=1	ACBD5	1.001	0.960632
Q5T8P6	RNA-binding protein 26 OS=Homo sapiens OX=9606 GN=RBM26 PE=1 SV=3	RBM26	0.864	0.100674
Q5T9A4	ATPase family AAA domain-containing protein 3B OS=Homo sapiens OX=9606 GN=ATAD3B PE=1 SV=1	ATAD3B	1.025	0.659885
Q5T9L3	Protein wntless homolog OS=Homo sapiens OX=9606 GN=WLS PE=1 SV=2	WLS	0.868	0.200448
Q5TA45	Integrator complex subunit 11 OS=Homo sapiens OX=9606 GN=INTS11 PE=1 SV=2	INTS11	0.96	0.660916
Q5TC12	ATP synthase mitochondrial F1 complex assembly factor 1 OS=Homo sapiens OX=9606 GN=ATPAF1 PE=1 SV=1	ATPAF1	1.053	0.514128
Q5TDH0	Protein DDI1 homolog 2 OS=Homo sapiens OX=9606 GN=DDI2 PE=1 SV=1	DDI2	1.073	0.681705
Q5TEC6	Histone HIST2H3PS2 OS=Homo sapiens OX=9606 GN=H3-2 PE=1 SV=1	H3-2	1.017	0.922287
Q5TFE4	5'-nucleotidase domain-containing protein 1 OS=Homo sapiens OX=9606 GN=NT5DC1 PE=1 SV=1	NT5DC1	0.93	0.335125
Q5TGZ0	MICOS complex subunit MIC10 OS=Homo sapiens OX=9606 GN=MICOS10 PE=1 SV=1	MICOS10	1.25	0.356194
Q5TZA2	Rootletin OS=Homo sapiens OX=9606 GN=CROCC PE=1	CROCC	1.016	0.700874

Q5U5X0	Complex III assembly factor LYRM7 OS=Homo sapiens OX=9606 GN=LYRM7 PE=1	LYRM7	0.942	0.55729
Q5UIP0	Telomere-associated protein RIF1 OS=Homo sapiens OX=9606 GN=RIF1 PE=1 SV=2	RIF1	0.917	0.22279
Q5VST6	Alpha/beta hydrolase domain-containing protein 17B OS=Homo sapiens OX=9606 GN=ABHD17B PE=1 SV=1	ABHD17B	1.643	
Q5VT52	Regulation of nuclear pre-mRNA domain-containing protein 2 OS=Homo sapiens OX=9606 GN=RPRD2 PE=1 SV=1	RPRD2	0.939	0.159206
Q5VT66	Mitochondrial amidoxime-reducing component 1 OS=Homo sapiens OX=9606 GN=MTARC1 PE=1 SV=1	MTARC1	0.668	0.399487
Q5VTR2	E3 ubiquitin-protein ligase BRE1A OS=Homo sapiens OX=9606 GN=RNF20 PE=1 SV=2	RNF20	0.908	0.395648
Q5VV42	Threonylcarbamoyladenosine tRNA methylthiotransferase OS=Homo sapiens OX=9606 GN=CDKAL1 PE=1 SV=1	CDKAL1	0.879	0.057303
Q5VW32	BRO1 domain-containing protein BROX OS=Homo sapiens OX=9606 GN=BROX PE=1 SV=1	BROX	1.089	0.470168
Q5VW38	Protein GPR107 OS=Homo sapiens OX=9606 GN=GPR107	GPR107	1.112	0.625633
Q5VWZ2	Lysophospholipase-like protein 1 OS=Homo sapiens OX=9606 GN=LYPLAL1 PE=1 SV=3	LYPLAL1	1.068	0.722361
Q5VYK3	Proteasome adapter and scaffold protein ECM29 OS=Homo sapiens OX=9606 GN=ECPAS PE=1	ECPAS	0.945	0.630317
Q5VYY1	Ankyrin repeat domain-containing protein 22 OS=Homo sapiens OX=9606 GN=ANKRD22 PE=2 SV=1	ANKRD22	1.111	0.449343
Q5VZL5	Zinc finger MYM-type protein 4 OS=Homo sapiens OX=9606 GN=ZMYM4 PE=1 SV=1	ZMYM4	0.986	0.916048
Q5W0Z9	Palmitoyltransferase ZDHHC20 OS=Homo sapiens OX=9606 GN=ZDHHC20 PE=1 SV=1	ZDHHC20	1.095	0.640511
Q5W111	SPRY domain-containing protein 7 OS=Homo sapiens OX=9606 GN=SPRYD7 PE=1 SV=2	SPRYD7	0.931	0.537362
Q5XKP0	MICOS complex subunit MIC13 OS=Homo sapiens OX=9606 GN=MICOS13 PE=1 SV=1	MICOS13	1.089	0.29722

Q5ZPR3	CD276 antigen OS=Homo sapiens OX=9606 GN=CD276 PE=1	CD276	0.923	0.404405
Q63ZY3	KN motif and ankyrin repeat domain-containing protein 2 OS=Homo sapiens OX=9606 GN=KANK2 PE=1 SV=1	KANK2	0.903	0.418886
Q658P3	Metalloreductase STEAP3 OS=Homo sapiens OX=9606 GN=STEAP3 PE=1 SV=2	STEAP3	0.969	0.840838
Q658Y4	Protein FAM91A1 OS=Homo sapiens OX=9606 GN=FAM91A1 PE=1 SV=3	FAM91A1	1.093	0.317834
Q66K74	Microtubule-associated protein 1S OS=Homo sapiens OX=9606 GN=MAP1S PE=1 SV=2	MAP1S	0.921	
Q68CP9	AT-rich interactive domain-containing protein 2 OS=Homo sapiens OX=9606 GN=ARID2 PE=1 SV=2	ARID2	1.016	0.818372
Q68CQ4	U3 small nucleolar RNA-associated protein 25 homolog OS=Homo sapiens OX=9606 GN=UTP25 PE=1 SV=2	UTP25	0.93	0.388533
Q68CZ2	Tensin-3 OS=Homo sapiens OX=9606 GN=TNS3 PE=1 SV=2	TNS3	0.984	0.936726
Q68DK2	Zinc finger FYVE domain-containing protein 26 OS=Homo sapiens OX=9606 GN=ZFYVE26 PE=1 SV=3	ZFYVE26	1.145	0.133829
Q68E01	Integrator complex subunit 3 OS=Homo sapiens OX=9606 GN=INTS3 PE=1 SV=1	INTS3	0.894	0.10433
Q69YH5	Cell division cycle-associated protein 2 OS=Homo sapiens OX=9606 GN=CDCA2 PE=1	CDCA2	0.991	
Q69YN2	CWF19-like protein 1 OS=Homo sapiens OX=9606 GN=CWF19L1 PE=1 SV=2	CWF19L1	1.015	
Q69YN4	Protein virilizer homolog OS=Homo sapiens OX=9606 GN=VIRMA PE=1 SV=2	VIRMA	0.94	0.277142
Q6DD87	Zinc finger protein 787 OS=Homo sapiens OX=9606 GN=ZNF787 PE=1 SV=4	ZNF787	0.971	0.732637
Q6DD88	Atlastin-3 OS=Homo sapiens OX=9606 GN=ATL3 PE=1 SV=1	ATL3	1.011	0.871014
Q6DKI1	60S ribosomal protein L7-like 1 OS=Homo sapiens OX=9606 GN=RPL7L1 PE=1 SV=2	RPL7L1	1.312	0.237051
Q6DKJ4	Nucleoredoxin OS=Homo sapiens OX=9606 GN=NXN PE=1 SV=2	NXN	0.967	0.71725

Q6FI81	Anamorsin OS=Homo sapiens OX=9606 GN=CIAPIN1 PE=1 SV=2	CIAPIN1	1.251	0.004808
Q6GMV3	Putative peptidyl-tRNA hydrolase PTRHD1 OS=Homo sapiens OX=9606 GN=PTRHD1 PE=1 SV=1	PTRHD1	1.04	0.659841
Q6I9Y2	THO complex subunit 7 homolog OS=Homo sapiens OX=9606 GN=THOC7 PE=1 SV=3	THOC7	0.943	0.734772
Q6IAA8	Ragulator complex protein LAMTOR1 OS=Homo sapiens OX=9606 GN=LAMTOR1 PE=1 SV=2	LAMTOR1	1.006	0.926758
Q6IBS0	Twinfilin-2 OS=Homo sapiens OX=9606 GN=TWF2 PE=1 SV=2	TWF2	1.087	0.38371
Q6IBW4	Condensin-2 complex subunit H2 OS=Homo sapiens OX=9606 GN=NCAPH2 PE=1 SV=1	NCAPH2	1.348	
Q6IN84	rRNA methyltransferase 1, mitochondrial OS=Homo sapiens OX=9606 GN=MRM1 PE=1	MRM1	0.747	
Q6IN85	Serine/threonine-protein phosphatase 4 regulatory subunit 3A OS=Homo sapiens OX=9606 GN=PPP4R3A PE=1 SV=1	PPP4R3A	0.851	0.046922
Q6IQ22	Ras-related protein Rab-12 OS=Homo sapiens OX=9606 GN=RAB12 PE=1 SV=3	RAB12	0.982	0.791306
Q6IQ23	Pleckstrin homology domain-containing family A member 7 OS=Homo sapiens OX=9606 GN=PLEKHA7 PE=1 SV=2	PLEKHA7	1.217	
Q6ISB3	Grainyhead-like protein 2 homolog OS=Homo sapiens OX=9606 GN=GRHL2 PE=1 SV=1	GRHL2	1.004	0.952019
Q6KC79	Nipped-B-like protein OS=Homo sapiens OX=9606 GN=NIPBL PE=1 SV=2	NIPBL	0.976	0.849352
Q6L8Q7	2',5'-phosphodiesterase 12 OS=Homo sapiens OX=9606 GN=PDE12 PE=1 SV=2	PDE12	0.977	0.71835
Q6N075	Molybdate-anion transporter OS=Homo sapiens OX=9606 GN=MFSD5 PE=1 SV=2	MFSD5	1000	0.001
Q6NTF9	Rhomboid domain-containing protein 2 OS=Homo sapiens OX=9606 GN=RHBDD2 PE=1 SV=2	RHBDD2	0.857	0.196851
Q6NUK1	Calcium-binding mitochondrial carrier protein SCaMC-1 OS=Homo sapiens OX=9606 GN=SLC25A24 PE=1 SV=2	SLC25A24	1.064	0.402254

Q6NUK4	Receptor expression-enhancing protein 3 OS=Homo sapiens OX=9606 GN=REEP3 PE=1	REEP3	0.843	
Q6NUM9	All-trans-retinol 13,14-reductase OS=Homo sapiens OX=9606 GN=RETSAT PE=1 SV=2	RETSAT	1.123	0.14374
Q6NUQ4	Transmembrane protein 214 OS=Homo sapiens OX=9606 GN=TMEM214 PE=1 SV=2	TMEM214	1.031	0.709128
Q6NVY1	3-hydroxyisobutyryl-CoA hydrolase, mitochondrial OS=Homo sapiens OX=9606	HIBCH	0.964	0.762289
Q6NXG1	Epithelial splicing regulatory protein 1 OS=Homo sapiens OX=9606 GN=ESRP1 PE=1	ESRP1	0.854	0.023101
Q6NYC1	Bifunctional arginine demethylase and lysyl-hydroxylase JMJD6 OS=Homo sapiens OX=9606 GN=JMJD6 PE=1 SV=1	JMJD6	0.988	0.774184
Q6NYC8	Phostensin OS=Homo sapiens OX=9606 GN=PPP1R18 PE=1 SV=1	PPP1R18	0.808	0.271112
Q6NZI2	Caveolae-associated protein 1 OS=Homo sapiens OX=9606 GN=CAVIN1 PE=1 SV=1	CAVIN1	0.971	0.646844
Q6NZY4	Zinc finger CCHC domain-containing protein 8 OS=Homo sapiens OX=9606 GN=ZCCHC8 PE=1 SV=2	ZCCHC8	0.811	0.11663
Q6P161	39S ribosomal protein L54, mitochondrial OS=Homo sapiens OX=9606 GN=MRPL54 PE=1 SV=1	MRPL54	0.96	0.645283
Q6P179	Endoplasmic reticulum aminopeptidase 2 OS=Homo sapiens OX=9606 GN=ERAP2	ERAP2	0.951	0.346059
Q6P1A2	Lysophospholipid acyltransferase 5 OS=Homo sapiens OX=9606 GN=LPCAT3 PE=1 SV=1	LPCAT3	0.968	0.640641
Q6P1J9	Parafibromin OS=Homo sapiens OX=9606 GN=CDC73 PE=1	CDC73	0.884	0.207529
Q6P1K2	Polyamine-modulated factor 1 OS=Homo sapiens OX=9606 GN=PMF1 PE=1 SV=2	PMF1	0.996	
Q6P1L8	39S ribosomal protein L14, mitochondrial OS=Homo sapiens OX=9606 GN=MRPL14 PE=1 SV=1	MRPL14	0.964	0.601363
Q6P1M0	Long-chain fatty acid transport protein 4 OS=Homo sapiens OX=9606 GN=SLC27A4 PE=1 SV=1	SLC27A4	1.048	0.602192

Q6P1Q0	LETM1 domain-containing protein 1 OS=Homo sapiens OX=9606 GN=LETMD1 PE=1 SV=1	LETMD1	1.214	
Q6P1X6	UPF0598 protein C8orf82 OS=Homo sapiens OX=9606 GN=C8orf82 PE=1 SV=2	C8orf82	0.895	0.394453
Q6P2E9	Enhancer of mRNA-decapping protein 4 OS=Homo sapiens OX=9606 GN=EDC4 PE=1 SV=1	EDC4	1.046	0.433083
Q6P2Q9	Pre-mRNA-processing-splicing factor 8 OS=Homo sapiens OX=9606 GN=PRPF8 PE=1 SV=2	PRPF8	0.964	0.425582
Q6P4E1	Protein GOLM2 OS=Homo sapiens OX=9606 GN=GOLM2	GOLM2	1.003	0.943169
Q6P4Q7	Metal transporter CNNM4 OS=Homo sapiens OX=9606 GN=CNNM4 PE=1 SV=3	CNNM4	0.93	0.340276
Q6P587	Acylpyruvase FAHD1, mitochondrial OS=Homo sapiens OX=9606 GN=FAHD1 PE=1	FAHD1	0.985	0.817515
Q6P5Z2	Serine/threonine-protein kinase N3 OS=Homo sapiens OX=9606 GN=PKN3 PE=1 SV=1	PKN3	1000	0.001
Q6P996	Pyridoxal-dependent decarboxylase domain-containing protein 1 OS=Homo sapiens OX=9606 GN=PDXDC1 PE=1	PDXDC1	0.845	0.630212
Q6P9B6	MTOR-associated protein MEAK7 OS=Homo sapiens OX=9606 GN=MEAK7 PE=1 SV=2	MEAK7	0.924	0.452081
Q6P9B9	Integrator complex subunit 5 OS=Homo sapiens OX=9606 GN=INTS5 PE=1 SV=1	INTS5	1.004	0.908814
Q6PCB5	Lysine-specific demethylase RSBN1L OS=Homo sapiens OX=9606 GN=RSBN1L PE=1 SV=2	RSBN1L	0.97	0.519073
Q6PCB7	Long-chain fatty acid transport protein 1 OS=Homo sapiens OX=9606 GN=SLC27A1 PE=1 SV=1	SLC27A1	1000	0.001
Q6PCB8	Embigin OS=Homo sapiens OX=9606 GN=EMB PE=1 SV=1	EMB	0.985	0.842463
Q6PD62	RNA polymerase-associated protein CTR9 homolog OS=Homo sapiens OX=9606 GN=CTR9	CTR9	0.923	0.223903
Q6PI48	Aspartate--tRNA ligase, mitochondrial OS=Homo sapiens OX=9606 GN=DARS2 PE=1	DARS2	1.022	0.704723
Q6PI78	Transmembrane protein 65 OS=Homo sapiens OX=9606 GN=TMEM65 PE=1 SV=2	TMEM65	0.963	

Q6PIU2	Neutral cholesterol ester hydrolase 1 OS=Homo sapiens OX=9606 GN=NCEH1 PE=1 SV=3	NCEH1	1.056	0.392862
Q6PJG2	Mitotic deacetylase-associated SANT domain protein OS=Homo sapiens OX=9606 GN=MIDEAS PE=1 SV=2	MIDEAS	0.687	0.008514
Q6PJG6	BRCA1-associated ATM activator 1 OS=Homo sapiens OX=9606 GN=BRAT1 PE=1 SV=2	BRAT1	0.856	0.074813
Q6PJT7	Zinc finger CCCH domain-containing protein 14 OS=Homo sapiens OX=9606 GN=ZC3H14 PE=1 SV=1	ZC3H14	0.966	0.642042
Q6PKG0	La-related protein 1 OS=Homo sapiens OX=9606 GN=LARP1 PE=1 SV=2	LARP1	1.184	0.031168
Q6PL18	ATPase family AAA domain-containing protein 2 OS=Homo sapiens OX=9606 GN=ATAD2 PE=1 SV=1	ATAD2	0.984	0.83962
Q6RFH5	WD repeat-containing protein 74 OS=Homo sapiens OX=9606 GN=WDR74 PE=1 SV=1	WDR74	0.978	0.80647
Q6RW13	Type-1 angiotensin II receptor-associated protein OS=Homo sapiens OX=9606 GN=AGTRAP PE=1 SV=1	AGTRAP	1.068	
Q6UB35	Monofunctional C1-tetrahydrofolate synthase, mitochondrial OS=Homo sapiens OX=9606 GN=MTHFD1L PE=1	MTHFD1L	0.928	0.403481
Q6UN15	Pre-mRNA 3'-end-processing factor FIP1 OS=Homo sapiens OX=9606 GN=FIP1L1 PE=1	FIP1L1	1	0.995987
Q6UW02	Cytochrome P450 20A1 OS=Homo sapiens OX=9606 GN=CYP20A1 PE=1 SV=1	CYP20A1	1.133	0.407591
Q6UW68	Transmembrane protein 205 OS=Homo sapiens OX=9606 GN=TMEM205 PE=1 SV=1	TMEM205	0.933	0.704916
Q6UWP7	Lysocardiolipin acyltransferase 1 OS=Homo sapiens OX=9606 GN=LCLAT1 PE=1 SV=1	LCLAT1	1.04	0.638138
Q6UX07	Dehydrogenase/reductase SDR family member 13 OS=Homo sapiens OX=9606 GN=DHRS13 PE=2 SV=1	DHRS13	1.006	0.963056
Q6UXH1	Protein disulfide isomerase CRELD2 OS=Homo sapiens OX=9606 GN=CRELD2 PE=1 SV=1	CRELD2	1.143	0.204389

Q6UXN9	WD repeat-containing protein 82 OS=Homo sapiens OX=9606 GN=WDR82 PE=1 SV=1	WDR82	0.886	0.007512
Q6UXV4	MICOS complex subunit MIC27 OS=Homo sapiens OX=9606 GN=APOOL PE=1 SV=1	APOOL	0.989	0.926395
Q6WCQ1	Myosin phosphatase Rho-interacting protein OS=Homo sapiens OX=9606 GN=MPRIP	MPRIP	1.058	0.403647
Q6WKZ4	Rab11 family-interacting protein 1 OS=Homo sapiens OX=9606 GN=RAB11FIP1 PE=1 SV=3	RAB11FIP1	0.945	0.423981
Q6Y1H2	Very-long-chain (3R)-3-hydroxyacyl-CoA dehydratase 2 OS=Homo sapiens OX=9606 GN=HACD2 PE=1 SV=1	HACD2	0.827	0.348837
Q6Y288	Beta-1,3-glucosyltransferase OS=Homo sapiens OX=9606 GN=B3GLCT PE=1 SV=2	B3GLCT	1.004	0.942408
Q6YHK3	CD109 antigen OS=Homo sapiens OX=9606 GN=CD109 PE=1	CD109	0.935	0.413599
Q6YN16	Hydroxysteroid dehydrogenase-like protein 2 OS=Homo sapiens OX=9606 GN=HSDL2 PE=1	HSDL2	1.04	0.55613
Q6ZMG9	Ceramide synthase 6 OS=Homo sapiens OX=9606 GN=CERS6 PE=1 SV=1	CERS6	0.793	0.367463
Q6ZN55	Zinc finger protein 574 OS=Homo sapiens OX=9606 GN=ZNF574 PE=1 SV=2	ZNF574	1.021	0.939585
Q6ZNA5	Ferric-chelate reductase 1 OS=Homo sapiens OX=9606 GN=FRRS1 PE=2 SV=2	FRRS1	0.936	0.565816
Q6ZNB6	NF-X1-type zinc finger protein NFXL1 OS=Homo sapiens OX=9606 GN=NFXL1 PE=1	NFXL1	1.153	0.675429
Q6ZRP7	Sulfhydryl oxidase 2 OS=Homo sapiens OX=9606 GN=QSOX2 PE=1 SV=3	QSOX2	1.181	0.319181
Q6ZRS2	Helicase SRCAP OS=Homo sapiens OX=9606 GN=SRCAP	SRCAP	1.043	0.31234
Q6ZRV2	Protein FAM83H OS=Homo sapiens OX=9606 GN=FAM83H PE=1 SV=3	FAM83H	1.019	0.683982
Q6ZT21	Transmembrane protein with metallophosphoesterase domain OS=Homo sapiens OX=9606 GN=TMPPE PE=1 SV=2	TMPPE	1.034	0.725279
Q6ZXV5	Protein O-mannosyl-transferase TMTC3 OS=Homo sapiens OX=9606 GN=TMTC3 PE=1	TMTC3	0.936	0.325138

Q70J99	Protein unc-13 homolog D OS=Homo sapiens OX=9606 GN=UNC13D PE=1 SV=1	UNC13D	1.012	0.843139
Q70UQ0	Inhibitor of nuclear factor kappa-B kinase-interacting protein OS=Homo sapiens OX=9606 GN=IKBIP PE=1 SV=1	IKBIP	0.944	0.570351
Q71DI3	Histone H3.2 OS=Homo sapiens OX=9606 GN=H3C15 PE=1	H3C15	1.22	0.551686
Q71RC2	La-related protein 4 OS=Homo sapiens OX=9606 GN=LARP4 PE=1 SV=3	LARP4	0.912	0.43797
Q71U36	Tubulin alpha-1A chain OS=Homo sapiens OX=9606 GN=TUBA1A PE=1 SV=1	TUBA1A	1.168	0.241546
Q71UM5	40S ribosomal protein S27-like OS=Homo sapiens OX=9606 GN=RPS27L PE=1 SV=3	RPS27L	1.084	0.461845
Q75N03	E3 ubiquitin-protein ligase Hakai OS=Homo sapiens OX=9606 GN=CBLL1 PE=1 SV=1	CBLL1	0.7	
Q76FK4	Nucleolar protein 8 OS=Homo sapiens OX=9606 GN=NOL8 PE=1 SV=1	NOL8	1.105	
Q7KZ85	Transcription elongation factor SPT6 OS=Homo sapiens OX=9606 GN=SUPT6H PE=1	SUPT6H	0.905	0.177418
Q7KZF4	Staphylococcal nuclease domain-containing protein 1 OS=Homo sapiens OX=9606 GN=SND1 PE=1 SV=1	SND1	0.967	0.494969
Q7L014	Probable ATP-dependent RNA helicase DDX46 OS=Homo sapiens OX=9606 GN=DDX46	DDX46	0.92	0.17039
Q7L0Y3	tRNA methyltransferase 10 homolog C OS=Homo sapiens OX=9606 GN=TRMT10C PE=1	TRMT10C	0.979	0.7264
Q7L1Q6	Basic leucine zipper and W2 domain-containing protein 1 OS=Homo sapiens OX=9606 GN=BZW1 PE=1 SV=1	BZW1	1.133	0.195777
Q7L1W4	Volume-regulated anion channel subunit LRRC8D OS=Homo sapiens OX=9606 GN=LRRC8D PE=1 SV=1	LRRC8D	1.478	0.372982
Q7L2E3	ATP-dependent RNA helicase DHX30 OS=Homo sapiens OX=9606 GN=DHX30 PE=1	DHX30	0.933	0.458598
Q7L2H7	Eukaryotic translation initiation factor 3 subunit M OS=Homo sapiens OX=9606 GN=EIF3M PE=1 SV=1	EIF3M	1.085	0.211524

Q7L2J0	7SK snRNA methylphosphate capping enzyme OS=Homo sapiens OX=9606 GN=MEPCE	MEPCE	0.997	0.913424
Q7L576	Cytoplasmic FMR1-interacting protein 1 OS=Homo sapiens OX=9606 GN=CYFIP1 PE=1	CYFIP1	1.071	0.201618
Q7L5N1	COP9 signalosome complex subunit 6 OS=Homo sapiens OX=9606 GN=COPS6 PE=1	COPS6	1.064	0.538767
Q7L5N7	Lysophosphatidylcholine acyltransferase 2 OS=Homo sapiens OX=9606 GN=LPCAT2 PE=1 SV=1	LPCAT2	1.074	0.217251
Q7L8L6	FAST kinase domain-containing protein 5, mitochondrial OS=Homo sapiens OX=9606 GN=FASTKD5 PE=1 SV=1	FASTKD5	1.132	
Q9H8S9	MOB kinase activator 1A OS=Homo sapiens OX=9606 GN=MOB1A PE=1 SV=4	MOB1A	1.115	0.428357
Q7LBC6	Lysine-specific demethylase 3B OS=Homo sapiens OX=9606 GN=KDM3B PE=1 SV=2	KDM3B	0.916	0.579473
Q7LGA3	Heparan sulfate 2-O-sulfotransferase 1 OS=Homo sapiens OX=9606 GN=HS2ST1	HS2ST1	1.064	0.560732
Q7RTV0	PHD finger-like domain-containing protein 5A OS=Homo sapiens OX=9606 GN=PHF5A	PHF5A	1.042	0.671274
Q7Z2K6	Endoplasmic reticulum metallopeptidase 1 OS=Homo sapiens OX=9606 GN=ERMP1 PE=1 SV=2	ERMP1	1.064	0.386149
Q7Z2T5	TRMT1-like protein OS=Homo sapiens OX=9606 GN=TRMT1L PE=1 SV=2	TRMT1L	1.014	0.914145
Q7Z2W4	Zinc finger CCCH-type antiviral protein 1 OS=Homo sapiens OX=9606 GN=ZC3HAV1 PE=1 SV=3	ZC3HAV1	0.966	0.57693
Q7Z2W9	39S ribosomal protein L21, mitochondrial OS=Homo sapiens OX=9606 GN=MRPL21 PE=1 SV=2	MRPL21	0.878	0.076873
Q7Z3B4	Nucleoporin p54 OS=Homo sapiens OX=9606 GN=NUP54	NUP54	1.034	0.555718
Q7Z3K3	Pogo transposable element with ZNF domain OS=Homo sapiens OX=9606 GN=POGZ PE=1 SV=2	POGZ	0.918	0.412302
Q7Z406	Myosin-14 OS=Homo sapiens OX=9606 GN=MYH14 PE=1	MYH14	1.153	0.297797

Q7Z417	Nuclear fragile X mental retardation-interacting protein 2 OS=Homo sapiens OX=9606 GN=NUFIP2 PE=1 SV=1	NUFIP2	0.922	0.103882
Q7Z434	Mitochondrial antiviral-signaling protein OS=Homo sapiens OX=9606 GN=MAVS PE=1	MAVS	0.976	0.580406
Q7Z478	ATP-dependent RNA helicase DHX29 OS=Homo sapiens OX=9606 GN=DHX29 PE=1	DHX29	1.128	0.19397
Q7Z4F1	Low-density lipoprotein receptor-related protein 10 OS=Homo sapiens OX=9606 GN=LRP10	LRP10	0.99	0.774861
Q7Z4H8	Protein O-glucosyltransferase 3 OS=Homo sapiens OX=9606 GN=POGLUT3 PE=1 SV=2	POGLUT3	0.971	0.495741
Q7Z4Q2	HEAT repeat-containing protein 3 OS=Homo sapiens OX=9606 GN=HEATR3 PE=1 SV=2	HEATR3	1.057	0.752901
Q7Z4V5	Hepatoma-derived growth factor-related protein 2 OS=Homo sapiens OX=9606 GN=HDGFL2 PE=1 SV=1	HDGFL2	0.881	0.105744
Q7Z4W1	L-xylulose reductase OS=Homo sapiens OX=9606 GN=DCXR PE=1 SV=2	DCXR	1.155	0.27121
Q7Z5G4	Golgin subfamily A member 7 OS=Homo sapiens OX=9606 GN=GOLGA7 PE=1 SV=2	GOLGA7	1.035	0.632645
Q7Z5K2	Wings apart-like protein homolog OS=Homo sapiens OX=9606 GN=WAPL PE=1 SV=1	WAPL	0.899	0.187967
Q7Z5L9	Interferon regulatory factor 2-binding protein 2 OS=Homo sapiens OX=9606 GN=IRF2BP2 PE=1 SV=2	IRF2BP2	0.831	0.044703
Q7Z6E9	E3 ubiquitin-protein ligase RBBP6 OS=Homo sapiens OX=9606 GN=RBBP6 PE=1 SV=1	RBBP6	0.853	0.20736
Q7Z6Z7	E3 ubiquitin-protein ligase HUWE1 OS=Homo sapiens OX=9606 GN=HUWE1 PE=1	HUWE1	1.043	0.487612
Q7Z739	YTH domain-containing family protein 3 OS=Homo sapiens OX=9606 GN=YTHDF3 PE=1 SV=1	YTHDF3	1.007	0.867917
Q7Z7F7	39S ribosomal protein L55, mitochondrial OS=Homo sapiens OX=9606 GN=MRPL55 PE=1 SV=1	MRPL55	0.961	0.736158

Q7Z7H5	Transmembrane emp24 domain-containing protein 4 OS=Homo sapiens OX=9606 GN=TMED4 PE=1 SV=1	TMED4	0.985	0.831947
Q7Z7H8	39S ribosomal protein L10, mitochondrial OS=Homo sapiens OX=9606 GN=MRPL10 PE=1 SV=3	MRPL10	1.183	0.219864
Q7Z7K0	COX assembly mitochondrial protein homolog OS=Homo sapiens OX=9606 GN=CMC1	CMC1	0.915	0.536508
Q7Z7K6	Centromere protein V OS=Homo sapiens OX=9606 GN=CENPV PE=1 SV=1	CENPV	0.769	0.283737
Q86SF2	N-acetylgalactosaminyltransferase 7 OS=Homo sapiens OX=9606 GN=GALNT7 PE=1 SV=1	GALNT7	0.971	0.72355
Q86SJ2	Amphoterin-induced protein 2 OS=Homo sapiens OX=9606 GN=AMIGO2 PE=1 SV=1	AMIGO2	1.053	0.450822
Q86SQ0	Pleckstrin homology-like domain family B member 2 OS=Homo sapiens OX=9606 GN=PHLDB2 PE=1 SV=2	PHLDB2	1.257	0.067698
Q86SX6	Glutaredoxin-related protein 5, mitochondrial OS=Homo sapiens OX=9606 GN=GLRX5 PE=1	GLRX5	0.992	0.515063
Q86T03	Type 1 phosphatidylinositol 4,5-bisphosphate 4-phosphatase OS=Homo sapiens OX=9606 GN=PIP4P1 PE=1 SV=1	PIP4P1	1.057	0.557081
Q86TB9	Protein PAT1 homolog 1 OS=Homo sapiens OX=9606 GN=PATL1 PE=1 SV=2	PATL1	1.177	0.181269
Q86U38	Nucleolar protein 9 OS=Homo sapiens OX=9606 GN=NOP9 PE=1 SV=1	NOP9	0.93	0.579873
Q86U42	Polyadenylate-binding protein 2 OS=Homo sapiens OX=9606 GN=PABPN1 PE=1 SV=3	PABPN1	1.022	0.589454
Q86U44	N6-adenosine-methyltransferase catalytic subunit OS=Homo sapiens OX=9606 GN=METTL3 PE=1 SV=2	METTL3	1.361	
Q86U86	Protein polybromo-1 OS=Homo sapiens OX=9606 GN=PBRM1 PE=1 SV=1	PBRM1	0.979	0.710532
Q86UE4	Protein LYRIC OS=Homo sapiens OX=9606 GN=MTDH PE=1	MTDH	0.984	0.738306
Q86UL3	Glycerol-3-phosphate acyltransferase 4 OS=Homo sapiens OX=9606 GN=GPAT4	GPAT4	1.058	0.458784

Q86UP2	Kinectin OS=Homo sapiens OX=9606 GN=KTN1 PE=1 SV=1	KTN1	1.014	0.681722
Q86UY8	5'-nucleotidase domain-containing protein 3 OS=Homo sapiens OX=9606 GN=NT5DC3 PE=1 SV=1	NT5DC3	1.311	0.017755
Q86V48	Leucine zipper protein 1 OS=Homo sapiens OX=9606 GN=LUZP1 PE=1 SV=2	LUZP1	0.783	0.005041
Q86V81	THO complex subunit 4 OS=Homo sapiens OX=9606 GN=ALYREF PE=1 SV=3	ALYREF	0.897	0.014638
Q86V85	Integral membrane protein GPR180 OS=Homo sapiens OX=9606 GN=GPR180 PE=2	GPR180	1.152	0.557234
Q86VI3	Ras GTPase-activating-like protein IQGAP3 OS=Homo sapiens OX=9606 GN=IQGAP3 PE=1 SV=2	IQGAP3	1.079	0.552851
Q86VM9	Zinc finger CCCH domain-containing protein 18 OS=Homo sapiens OX=9606 GN=ZC3H18 PE=1 SV=2	ZC3H18	0.815	0.043886
Q86VP1	Tax1-binding protein 1 OS=Homo sapiens OX=9606 GN=TAX1BP1 PE=1 SV=2	TAX1BP1	1.017	0.920069
Q86VP6	Cullin-associated NEDD8-dissociated protein 1 OS=Homo sapiens OX=9606 GN=CAND1 PE=1 SV=2	CAND1	0.966	0.736058
Q86VR2	Reticulophagy regulator 3 OS=Homo sapiens OX=9606 GN=RETREG3 PE=1 SV=1	RETREG3	1.11	0.176157
Q86W42	THO complex subunit 6 homolog OS=Homo sapiens OX=9606 GN=THOC6 PE=1 SV=1	THOC6	0.947	0.323959
Q86W92	Liprin-beta-1 OS=Homo sapiens OX=9606 GN=PPFIBP1 PE=1 SV=2	PPFIBP1	0.998	0.966805
Q86WA6	Valacyclovir hydrolase OS=Homo sapiens OX=9606 GN=BPHL PE=1 SV=1	BPHL	1.031	0.89291
Q86WB0	Nuclear-interacting partner of ALK OS=Homo sapiens OX=9606 GN=ZC3HC1 PE=1 SV=1	ZC3HC1	1.008	0.798134
Q86WX3	Active regulator of SIRT1 OS=Homo sapiens OX=9606 GN=RPS19BP1 PE=1 SV=1	RPS19BP1	1.055	0.53089
Q86X29	Lipolysis-stimulated lipoprotein receptor OS=Homo sapiens OX=9606 GN=LSR PE=1 SV=4	LSR	1.057	0.516276

Q86XI2	Condensin-2 complex subunit G2 OS=Homo sapiens OX=9606 GN=NCAPG2 PE=1 SV=1	NCAPG2	1.024	0.971658
Q86XL3	Ankyrin repeat and LEM domain-containing protein 2 OS=Homo sapiens OX=9606 GN=ANKLE2 PE=1 SV=4	ANKLE2	1.062	0.283976
Q86XP3	ATP-dependent RNA helicase DDX42 OS=Homo sapiens OX=9606 GN=DDX42 PE=1	DDX42	0.85	0.048393
Q86XZ4	Spermatogenesis-associated serine-rich protein 2 OS=Homo sapiens OX=9606 GN=SPATS2 PE=1	SPATS2	1.407	0.016576
Q86Y07	Serine/threonine-protein kinase VRK2 OS=Homo sapiens OX=9606 GN=VRK2 PE=1 SV=3	VRK2	0.903	0.166648
Q86Y39	NADH dehydrogenase [ubiquinone] 1 alpha subcomplex subunit 11 OS=Homo sapiens OX=9606 GN=NDUFA11 PE=1	NDUFA11	1.113	0.531884
Q86Y79	Probable peptidyl-tRNA hydrolase OS=Homo sapiens OX=9606 GN=PTRH1 PE=1 SV=1	PTRH1	1.084	
Q86Y82	Syntaxin-12 OS=Homo sapiens OX=9606 GN=STX12 PE=1	STX12	0.933	0.569574
Q86YP4	Transcriptional repressor p66-alpha OS=Homo sapiens OX=9606 GN=GATAD2A PE=1	GATAD2A	0.906	0.359537
Q86YQ8	Copine-8 OS=Homo sapiens OX=9606 GN=CPNE8 PE=1	CPNE8	1.18	0.03288
Q86YS6	Ras-related protein Rab-43 OS=Homo sapiens OX=9606 GN=RAB43 PE=1 SV=1	RAB43	1.035	
Q8IUF8	Ribosomal oxygenase 2 OS=Homo sapiens OX=9606 GN=RIOX2 PE=1 SV=1	RIOX2	0.795	0.20692
Q8IUH4	Palmitoyltransferase ZDHHC13 OS=Homo sapiens OX=9606 GN=ZDHHC13 PE=1 SV=3	ZDHHC13	0.95	0.142252
Q8IV08	5'-3' exonuclease PLD3 OS=Homo sapiens OX=9606 GN=PLD3 PE=1 SV=1	PLD3	1.014	0.84485
Q8IVF2	Protein AHNAK2 OS=Homo sapiens OX=9606 GN=AHNAK2 PE=1 SV=2	AHNAK2	1.023	0.844961
Q8IVL5	Prolyl 3-hydroxylase 2 OS=Homo sapiens OX=9606 GN=P3H2 PE=1 SV=1	P3H2	1.037	0.722811
Q8IVS2	Malonyl-CoA-acyl carrier protein transacylase, mitochondrial OS=Homo sapiens OX=9606 GN=MCAT PE=1 SV=2	MCAT	0.963	0.732562

Q8IVT2	Mitotic interactor and substrate of PLK1 OS=Homo sapiens OX=9606 GN=MISP PE=1 SV=1	MISP	0.952	0.526506
Q8IWA0	WD repeat-containing protein 75 OS=Homo sapiens OX=9606 GN=WDR75 PE=1 SV=1	WDR75	0.998	0.939243
Q8IWA4	Mitofusin-1 OS=Homo sapiens OX=9606 GN=MFN1 PE=1 SV=3	MFN1	1.083	0.150923
Q8IWA5	Choline transporter-like protein 2 OS=Homo sapiens OX=9606 GN=SLC44A2 PE=1 SV=3	SLC44A2	1.043	0.701472
Q8IWB7	WD repeat and FYVE domain-containing protein 1 OS=Homo sapiens OX=9606 GN=WDFY1 PE=1 SV=1	WDFY1	0.881	0.314201
Q8IWS0	PHD finger protein 6 OS=Homo sapiens OX=9606 GN=PHF6 PE=1 SV=1	PHF6	0.895	0.119385
Q8IWT6	Volume-regulated anion channel subunit LRRC8A OS=Homo sapiens OX=9606 GN=LRRC8A PE=1 SV=1	LRRC8A	1.055	0.486913
Q8IWX8	Calcium homeostasis endoplasmic reticulum protein OS=Homo sapiens OX=9606 GN=CHERP	CHERP	0.96	0.23608
Q8IX12	Cell division cycle and apoptosis regulator protein 1 OS=Homo sapiens OX=9606 GN=CCAR1 PE=1 SV=2	CCAR1	0.94	0.192811
Q8IXB1	DnaJ homolog subfamily C member 10 OS=Homo sapiens OX=9606 GN=DNAJC10 PE=1	DNAJC10	0.995	0.872421
Q8IXH7	Negative elongation factor C/D OS=Homo sapiens OX=9606 GN=NELFCD PE=1 SV=2	NELFCD	0.842	0.06249
Q8IXI1	Mitochondrial Rho GTPase 2 OS=Homo sapiens OX=9606 GN=RHOT2 PE=1 SV=2	RHOT2	0.984	0.781815
Q8IXI2	Mitochondrial Rho GTPase 1 OS=Homo sapiens OX=9606 GN=RHOT1 PE=1 SV=2	RHOT1	0.96	0.572592
Q8IXK0	Polyhomeotic-like protein 2 OS=Homo sapiens OX=9606 GN=PHC2 PE=1 SV=1	PHC2	0.991	0.853297
Q8IXK2	Polypeptide N-acetylgalactosaminyltransferase 12 OS=Homo sapiens OX=9606 GN=GALNT12 PE=1 SV=3	GALNT12	1.104	0.189217
Q8IXM3	39S ribosomal protein L41, mitochondrial OS=Homo sapiens OX=9606 GN=MRPL41 PE=1 SV=1	MRPL41	0.985	0.889277

Q8IXM6	Nurim OS=Homo sapiens OX=9606 GN=NRM PE=1 SV=1	NRM	0.923	0.656017
Q8IXT5	RNA-binding protein 12B OS=Homo sapiens OX=9606 GN=RBM12B PE=1 SV=2	RBM12B	1.001	0.962471
Q8IXU6	Solute carrier family 35 member F2 OS=Homo sapiens OX=9606 GN=SLC35F2 PE=1 SV=1	SLC35F2	1.033	0.513316
Q8IY18	Structural maintenance of chromosomes protein 5 OS=Homo sapiens OX=9606 GN=SMC5 PE=1 SV=2	SMC5	0.855	
Q8IY37	Probable ATP-dependent RNA helicase DHX37 OS=Homo sapiens OX=9606 GN=DHX37	DHX37	1.017	0.955319
Q8IY57	YY1-associated factor 2 OS=Homo sapiens OX=9606 GN=YAF2 PE=1 SV=3	YAF2	0.001	0.001
Q8IY67	Ribonucleoprotein PTB-binding 1 OS=Homo sapiens OX=9606 GN=RAVER1 PE=1 SV=1	RAVER1	0.865	0.098059
Q8IY81	pre-rRNA 2'-O-ribose RNA methyltransferase FTSJ3 OS=Homo sapiens OX=9606 GN=FTSJ3 PE=1 SV=2	FTSJ3	0.97	0.707569
Q8IY95	Transmembrane protein 192 OS=Homo sapiens OX=9606 GN=TMEM192 PE=1 SV=1	TMEM192	0.928	0.418066
Q8IYB3	Serine/arginine repetitive matrix protein 1 OS=Homo sapiens OX=9606 GN=SRRM1 PE=1	SRRM1	0.886	0.340955
Q8IYB8	ATP-dependent RNA helicase SUPV3L1, mitochondrial OS=Homo sapiens OX=9606 GN=SUPV3L1 PE=1 SV=1	SUPV3L1	1.015	0.938701
Q8IYD1	Eukaryotic peptide chain release factor GTP-binding subunit ERF3B OS=Homo sapiens OX=9606 GN=GSPT2 PE=1	GSPT2	1.454	
Q8IYS2	Uncharacterized protein KIAA2013 OS=Homo sapiens OX=9606 GN=KIAA2013 PE=1	KIAA2013	1.033	0.771209
Q8IYU8	Calcium uptake protein 2, mitochondrial OS=Homo sapiens OX=9606 GN=MICU2 PE=1	MICU2	1.012	0.951611
Q8IZ52	Chondroitin sulfate synthase 2 OS=Homo sapiens OX=9606 GN=CHPF PE=1 SV=2	CHPF	0.73	
Q8IZ81	ELMO domain-containing protein 2 OS=Homo sapiens OX=9606 GN=ELMOD2 PE=1 SV=1	ELMOD2	0.95	0.699891

Q8IZ83	Aldehyde dehydrogenase family 16 member A1 OS=Homo sapiens OX=9606 GN=ALDH16A1 PE=1 SV=2	ALDH16A1	1.19	0.320899
Q8IZL8	Proline-, glutamic acid- and leucine-rich protein 1 OS=Homo sapiens OX=9606 GN=PELP1	PELP1	1.02	0.727981
Q8IZP0	Abl interactor 1 OS=Homo sapiens OX=9606 GN=ABI1 PE=1 SV=4	ABI1	1.042	0.580845
Q8IZV5	Retinol dehydrogenase 10 OS=Homo sapiens OX=9606 GN=RDH10 PE=1 SV=1	RDH10	0.963	
Q8N0U8	Vitamin K epoxide reductase complex subunit 1-like protein 1 OS=Homo sapiens OX=9606 GN=VKORC1L1 PE=1 SV=2	VKORC1L1	1	0.947523
Q8N0X7	Spartin OS=Homo sapiens OX=9606 GN=SPART PE=1	SPART	1.083	0.154389
Q8N128	Protein FAM177A1 OS=Homo sapiens OX=9606 GN=FAM177A1 PE=1 SV=1	FAM177A1	0.957	0.426823
Q8N138	ORM1-like protein 3 OS=Homo sapiens OX=9606 GN=ORMDL3 PE=1 SV=1	ORMDL3	1.008	0.911062
Q8N163	Cell cycle and apoptosis regulator protein 2 OS=Homo sapiens OX=9606 GN=CCAR2 PE=1	CCAR2	0.961	0.357558
Q8N183	NADH dehydrogenase [ubiquinone] 1 alpha subcomplex assembly factor 2 OS=Homo sapiens OX=9606 GN=NDUFAF2	NDUFAF2	1.145	0.143244
Q8N1F7	Nuclear pore complex protein Nup93 OS=Homo sapiens OX=9606 GN=NUP93 PE=1	NUP93	0.959	0.173206
Q8N1G0	Zinc finger protein 687 OS=Homo sapiens OX=9606 GN=ZNF687 PE=1 SV=1	ZNF687	0.901	0.223027
Q8N1G4	Leucine-rich repeat-containing protein 47 OS=Homo sapiens OX=9606 GN=LRRC47 PE=1 SV=1	LRRC47	1.241	0.100653
Q8N1P7	Beta/gamma crystallin domain-containing protein 2 OS=Homo sapiens OX=9606 GN=CRYBG2 PE=2 SV=2	CRYBG2	1.038	0.713389
Q8N201	Integrator complex subunit 1 OS=Homo sapiens OX=9606 GN=INTS1 PE=1 SV=2	INTS1	0.893	0.024526
Q8N2F6	Armadillo repeat-containing protein 10 OS=Homo sapiens OX=9606 GN=ARMC10 PE=1	ARMC10	1.017	0.774804

Q8N2K0	Lysophosphatidylserine lipase ABHD12 OS=Homo sapiens OX=9606 GN=ABHD12 PE=1 SV=2	ABHD12	1.011	0.902729
Q8N357	Solute carrier family 35 member F6 OS=Homo sapiens OX=9606 GN=SLC35F6 PE=1 SV=1	SLC35F6	0.781	0.377445
Q8N3C0	Activating signal cointegrator 1 complex subunit 3 OS=Homo sapiens OX=9606 GN=ASCC3 PE=1 SV=3	ASCC3	1.316	0.165471
Q8N3F8	MICAL-like protein 1 OS=Homo sapiens OX=9606 GN=MICALL1 PE=1 SV=2	MICALL1	1.115	0.278613
Q8N3R9	Protein PALS1 OS=Homo sapiens OX=9606 GN=PALS1 PE=1	PALS1	1.103	
Q8N3U4	Cohesin subunit SA-2 OS=Homo sapiens OX=9606 GN=STAG2 PE=1 SV=3	STAG2	0.933	0.131216
Q8N442	Translation factor GUF1, mitochondrial OS=Homo sapiens OX=9606 GN=GUF1 PE=1 SV=1	GUF1	1.439	
Q8N4H5	Mitochondrial import receptor subunit TOM5 homolog OS=Homo sapiens OX=9606 GN=TOMM5 PE=1 SV=1	TOMM5	0.8	0.169034
Q8N4Q1	Mitochondrial intermembrane space import and assembly protein 40 OS=Homo sapiens OX=9606 GN=CHCHD4 PE=1 SV=1	CHCHD4	0.972	0.800818
Q8N4V1	ER membrane protein complex subunit 5 OS=Homo sapiens OX=9606 GN=MMGT1 PE=1 SV=1	MMGT1	1.053	0.359363
Q8N4X5	Actin filament-associated protein 1-like 2 OS=Homo sapiens OX=9606 GN=AFAP1L2 PE=1	AFAP1L2	1.004	0.990429
Q8N511	Transmembrane protein 199 OS=Homo sapiens OX=9606 GN=TMEM199 PE=1 SV=1	TMEM199	0.993	
Q8N543	Prolyl 3-hydroxylase OGFOD1 OS=Homo sapiens OX=9606 GN=OGFOD1 PE=1 SV=1	OGFOD1	0.948	0.829185
Q8N5K1	CDGSH iron-sulfur domain-containing protein 2 OS=Homo sapiens OX=9606 GN=CISD2 PE=1 SV=1	CISD2	0.924	0.267806
Q8N5M1	ATP synthase mitochondrial F1 complex assembly factor 2 OS=Homo sapiens OX=9606 GN=ATPAF2 PE=1 SV=1	ATPAF2	1.089	0.05797

Q8N5M9	Protein jagunal homolog 1 OS=Homo sapiens OX=9606 GN=JAGN1 PE=1 SV=1	JAGN1	1.007	0.898636
Q8N5N7	39S ribosomal protein L50, mitochondrial OS=Homo sapiens OX=9606 GN=MRPL50 PE=1 SV=2	MRPL50	0.962	0.670701
Q8N684	Cleavage and polyadenylation specificity factor subunit 7 OS=Homo sapiens OX=9606 GN=CPSF7 PE=1 SV=1	CPSF7	0.962	0.528913
Q8N6S5	ADP-ribosylation factor-like protein 6-interacting protein 6 OS=Homo sapiens OX=9606 GN=ARL6IP6 PE=1 SV=1	ARL6IP6	1.01	0.89037
Q8N6T3	ADP-ribosylation factor GTPase-activating protein 1 OS=Homo sapiens OX=9606 GN=ARFGAP1 PE=1 SV=2	ARFGAP1	1.134	0.737377
Q8N726	Tumor suppressor ARF OS=Homo sapiens OX=9606 GN=CDKN2A PE=1 SV=2	CDKN2A	1.003	0.999918
Q8N766	ER membrane protein complex subunit 1 OS=Homo sapiens OX=9606 GN=EMC1 PE=1 SV=1	EMC1	1.038	0.413439
Q8N7H5	RNA polymerase II-associated factor 1 homolog OS=Homo sapiens OX=9606 GN=PAF1	PAF1	1.01	0.905259
Q8N806	Putative E3 ubiquitin-protein ligase UBR7 OS=Homo sapiens OX=9606 GN=UBR7 PE=1 SV=2	UBR7	0.897	
Q8N884	Cyclic GMP-AMP synthase OS=Homo sapiens OX=9606 GN=CGAS PE=1 SV=2	CGAS	0.802	0.04818
Q8N8A6	ATP-dependent RNA helicase DDX51 OS=Homo sapiens OX=9606 GN=DDX51 PE=1	DDX51	0.94	0.571499
Q8N8S7	Protein enabled homolog OS=Homo sapiens OX=9606 GN=ENAH PE=1 SV=2	ENAH	1.032	0.574166
Q8N8Z6	Discoidin, CUB and LCCL domain-containing protein 1 OS=Homo sapiens OX=9606 GN=DCBLD1 PE=1 SV=2	DCBLD1	1.011	0.881106
Q8N983	39S ribosomal protein L43, mitochondrial OS=Homo sapiens OX=9606 GN=MRPL43 PE=1 SV=1	MRPL43	0.937	0.44498
Q8N9T8	Protein KRI1 homolog OS=Homo sapiens OX=9606 GN=KRI1 PE=1 SV=3	KRI1	1.001	0.978045

Q8NAV1	Pre-mRNA-splicing factor 38A OS=Homo sapiens OX=9606 GN=PRPF38A PE=1 SV=1	PRPF38A	0.938	0.336522
Q8NB49	Phospholipid-transporting ATPase IG OS=Homo sapiens OX=9606 GN=ATP11C PE=1 SV=3	ATP11C	0.93	0.378108
Q8NBJ4	Golgi membrane protein 1 OS=Homo sapiens OX=9606 GN=GOLM1 PE=1 SV=1	GOLM1	0.903	0.445703
Q8NBJ5	Procollagen galactosyltransferase 1 OS=Homo sapiens OX=9606 GN=COLGALT1 PE=1 SV=1	COLGALT 1	1.042	0.363109
Q8NBJ7	Inactive C-alpha-formylglycine-generating enzyme 2 OS=Homo sapiens OX=9606 GN=SUMF2 PE=1 SV=2	SUMF2	0.939	0.220769
Q8NBM4	Ubiquitin-associated domain-containing protein 2 OS=Homo sapiens OX=9606 GN=UBAC2 PE=1 SV=1	UBAC2	1.037	0.540446
Q8NBM8	Prenylcysteine oxidase-like OS=Homo sapiens OX=9606 GN=PCYOX1L PE=1 SV=2	PCYOX1L	1.101	0.374624
Q8NBN3	Transmembrane protein 87A OS=Homo sapiens OX=9606 GN=TMEM87A PE=1 SV=3	TMEM87A	1.127	0.18852
Q8NBN7	Retinol dehydrogenase 13 OS=Homo sapiens OX=9606 GN=RDH13 PE=1 SV=2	RDH13	0.827	
Q8NBQ5	Estradiol 17-beta-dehydrogenase 11 OS=Homo sapiens OX=9606 GN=HSD17B11 PE=1 SV=3	HSD17B11	1.095	0.199085
Q8NBS9	Thioredoxin domain-containing protein 5 OS=Homo sapiens OX=9606 GN=TXNDC5 PE=1 SV=2	TXNDC5	1.098	0.122091
Q8NBU5	Outer mitochondrial transmembrane helix translocase OS=Homo sapiens OX=9606	ATAD1	0.944	0.155518
Q8NBX0	Saccharopine dehydrogenase-like oxidoreductase OS=Homo sapiens OX=9606 GN=SCCPDH PE=1 SV=1	SCCPDH	1.124	0.221901
Q8NBZ7	UDP-glucuronic acid decarboxylase 1 OS=Homo sapiens OX=9606 GN=UXS1	UXS1	1.125	0.691885
Q8NC42	E3 ubiquitin-protein ligase RNF149 OS=Homo sapiens OX=9606 GN=RNF149 PE=2	RNF149	1.059	0.392942
Q8NC51	Plasminogen activator inhibitor 1 RNA-binding protein OS=Homo sapiens OX=9606 GN=SERBP1 PE=1 SV=2	SERBP1	0.921	0.261685

Q8NC56	LEM domain-containing protein 2 OS=Homo sapiens OX=9606 GN=LEMD2 PE=1 SV=1	LEMD2	1.036	0.737505
Q8NCA5	Protein FAM98A OS=Homo sapiens OX=9606 GN=FAM98A PE=1 SV=2	FAM98A	1.04	0.747918
Q8NCG7	Diacylglycerol lipase-beta OS=Homo sapiens OX=9606 GN=DAGLB PE=1 SV=2	DAGLB	1.088	0.182152
Q8NCH0	Carbohydrate sulfotransferase 14 OS=Homo sapiens OX=9606 GN=CHST14 PE=1 SV=2	CHST14	1.135	0.102614
Q8NCN5	Pyruvate dehydrogenase phosphatase regulatory subunit, mitochondrial OS=Homo sapiens OX=9606 GN=PDPR PE=1 SV=2	PDPR	0.957	0.637957
Q8NCW5	NAD(P)H-hydrate epimerase OS=Homo sapiens OX=9606 GN=NAXE PE=1 SV=2	NAXE	0.928	0.272902
Q8ND56	Protein LSM14 homolog A OS=Homo sapiens OX=9606 GN=LSM14A PE=1 SV=3	LSM14A	1.202	0.031335
Q8ND76	Cyclin-Y OS=Homo sapiens OX=9606 GN=CCNY PE=1 SV=2	CCNY	0.953	0.459388
Q8ND82	Zinc finger protein 280C OS=Homo sapiens OX=9606 GN=ZNF280C PE=1 SV=1	ZNF280C	1.268	
Q8NDI1	EH domain-binding protein 1 OS=Homo sapiens OX=9606 GN=EHBP1 PE=1 SV=3	EHBP1	1.015	0.92403
Q8NDT2	Putative RNA-binding protein 15B OS=Homo sapiens OX=9606 GN=RBM15B PE=1 SV=3	RBM15B	1.093	0.552409
Q8NDV3	Structural maintenance of chromosomes protein 1B OS=Homo sapiens OX=9606 GN=SMC1B PE=2 SV=2	SMC1B	0.986	
Q8NDX5	Polyhomeotic-like protein 3 OS=Homo sapiens OX=9606 GN=PHC3 PE=1 SV=1	PHC3	0.87	0.261942
Q8NDZ4	Divergent protein kinase domain 2A OS=Homo sapiens OX=9606 GN=DIPK2A PE=1 SV=1	DIPK2A	0.869	0.395241
Q8NE71	ATP-binding cassette sub-family F member 1 OS=Homo sapiens OX=9606 GN=ABCF1 PE=1	ABCF1	1.054	0.524413
Q8NE86	Calcium uniporter protein, mitochondrial OS=Homo sapiens OX=9606 GN=MCU PE=1 SV=1	MCU	1.135	0.164742
Q8NEF9	Serum response factor-binding protein 1 OS=Homo sapiens OX=9606 GN=SRFBP1 PE=1	SRFBP1	1.175	

Q8NEJ9	Neuroguidin OS=Homo sapiens OX=9606 GN=NGDN PE=1	NGDN	0.89	0.284979
Q8NEM2	SHC SH2 domain-binding protein 1 OS=Homo sapiens OX=9606 GN=SHCBP1 PE=1 SV=3	SHCBP1	0.989	0.945298
Q8NEW0	Zinc transporter 7 OS=Homo sapiens OX=9606 GN=SLC30A7 PE=2 SV=1	SLC30A7	1.059	0.510669
Q8NEY8	Periphilin-1 OS=Homo sapiens OX=9606 GN=PPHLN1 PE=1 SV=2	PPHLN1	0.963	0.747945
Q8NF37	Lysophosphatidylcholine acyltransferase 1 OS=Homo sapiens OX=9606 GN=LPCAT1 PE=1 SV=2	LPCAT1	1.088	0.310372
Q8NFD5	AT-rich interactive domain-containing protein 1B OS=Homo sapiens OX=9606 GN=ARID1B PE=1 SV=2	ARID1B	0.971	0.789781
Q8NFF5	FAD synthase OS=Homo sapiens OX=9606 GN=FLAD1 PE=1	FLAD1	0.998	0.940941
Q8NFH3	Nucleoporin Nup43 OS=Homo sapiens OX=9606 GN=NUP43 PE=1 SV=1	NUP43	1.062	0.441754
Q8NFH4	Nucleoporin Nup37 OS=Homo sapiens OX=9606 GN=NUP37 PE=1 SV=1	NUP37	1.07	0.254116
Q8NFH5	Nucleoporin NUP35 OS=Homo sapiens OX=9606 GN=NUP35 PE=1 SV=1	NUP35	0.941	0.483845
Q8NFJ5	Retinoic acid-induced protein 3 OS=Homo sapiens OX=9606 GN=GPRC5A PE=1 SV=2	GPRC5A	1.206	0.059778
Q8NFQ8	Torsin-1A-interacting protein 2 OS=Homo sapiens OX=9606 GN=TOR1AIP2 PE=1 SV=1	TOR1AIP2	0.983	0.867688
Q8NFV4	Protein ABHD11 OS=Homo sapiens OX=9606 GN=ABHD11 PE=1 SV=1	ABHD11	1.054	0.06064
Q8NFW8	N-acylneuraminate cytidylyltransferase OS=Homo sapiens OX=9606 GN=CMAS PE=1 SV=2	CMAS	1.005	0.949762
Q8NG11	Tetraspanin-14 OS=Homo sapiens OX=9606 GN=TSPAN14 PE=1 SV=1	TSPAN14	0.857	0.106821
Q8NHH9	Atlastin-2 OS=Homo sapiens OX=9606 GN=ATL2 PE=1 SV=2	ATL2	1.002	0.995112
Q8NHP8	Putative phospholipase B-like 2 OS=Homo sapiens OX=9606 GN=PLBD2 PE=1 SV=2	PLBD2	0.818	0.074304

Q8NHY3	GAS2-like protein 2 OS=Homo sapiens OX=9606 GN=GAS2L2 PE=1 SV=1	GAS2L2	1.127	
Q8NI22	Multiple coagulation factor deficiency protein 2 OS=Homo sapiens OX=9606 GN=MCFD2 PE=1 SV=1	MCFD2	0.947	0.279007
Q8NI27	THO complex subunit 2 OS=Homo sapiens OX=9606 GN=THOC2 PE=1 SV=2	THOC2	0.933	0.506437
Q8NI36	WD repeat-containing protein 36 OS=Homo sapiens OX=9606 GN=WDR36 PE=1 SV=1	WDR36	0.972	0.644684
Q8TAE8	Growth arrest and DNA damage-inducible proteins-interacting protein 1 OS=Homo sapiens OX=9606 GN=GADD45GIP1	GADD45GIP1	1.107	0.392292
Q8TAF3	WD repeat-containing protein 48 OS=Homo sapiens OX=9606 GN=WDR48 PE=1 SV=1	WDR48	0.98	
Q8TAQ2	SWI/SNF complex subunit SMARCC2 OS=Homo sapiens OX=9606 GN=SMARCC2 PE=1 SV=1	SMARCC2	0.914	0.01632
Q8TAT6	Nuclear protein localization protein 4 homolog OS=Homo sapiens OX=9606 GN=NPLOC4 PE=1 SV=3	NPLOC4	1.072	0.477364
Q8TB36	Ganglioside-induced differentiation-associated protein 1 OS=Homo sapiens OX=9606 GN=GDAP1 PE=1 SV=3	GDAP1	1.103	0.201738
Q8TB37	Iron-sulfur protein NUBPL OS=Homo sapiens OX=9606 GN=NUBPL PE=1 SV=3	NUBPL	1.022	0.940831
Q8TB61	Adenosine 3'-phospho 5'-phosphosulfate transporter 1 OS=Homo sapiens OX=9606 GN=SLC35B2 PE=1 SV=1	SLC35B2	1.006	0.920748
Q8TB96	T-cell immunomodulatory protein OS=Homo sapiens OX=9606 GN=ITFG1 PE=1 SV=1	ITFG1	0.866	
Q8TBA6	Golgin subfamily A member 5 OS=Homo sapiens OX=9606 GN=GOLGA5 PE=1 SV=3	GOLGA5	1.04	
Q8TBC4	NEDD8-activating enzyme E1 catalytic subunit OS=Homo sapiens OX=9606 GN=UBA3	UBA3	0.935	0.688903
Q8TBP6	Solute carrier family 25 member 40 OS=Homo sapiens OX=9606 GN=SLC25A40 PE=1 SV=1	SLC25A40	1.006	0.991733

Q8TBQ9	Protein kish-A OS=Homo sapiens OX=9606 GN=TMEM167A PE=1 SV=1	TMEM167A	1.02	
Q8TBX8	Phosphatidylinositol 5-phosphate 4-kinase type-2 gamma OS=Homo sapiens OX=9606 GN=PIP4K2C PE=1 SV=3	PIP4K2C	1.069	0.504669
Q8TC12	Retinol dehydrogenase 11 OS=Homo sapiens OX=9606 GN=RDH11 PE=1 SV=2	RDH11	1.118	0.131954
Q8TCC3	39S ribosomal protein L30, mitochondrial OS=Homo sapiens OX=9606 GN=MRPL30 PE=1 SV=1	MRPL30	1.183	0.284086
Q8TCJ2	Dolichyl-diphosphooligosaccharide--protein glycosyltransferase subunit STT3B OS=Homo sapiens OX=9606	STT3B	1.068	0.424909
Q8TCS8	Polyribonucleotide nucleotidyltransferase 1, mitochondrial OS=Homo sapiens OX=9606 GN=PNPT1 PE=1	PNPT1	1.015	0.712629
Q8TCT8	Signal peptide peptidase-like 2A OS=Homo sapiens OX=9606 GN=SPPL2A PE=1 SV=2	SPPL2A	1.019	0.952321
Q8TCT9	Minor histocompatibility antigen H13 OS=Homo sapiens OX=9606 GN=HM13 PE=1 SV=1	HM13	0.981	0.809578
Q8TDD1	ATP-dependent RNA helicase DDX54 OS=Homo sapiens OX=9606 GN=DDX54 PE=1	DDX54	1.157	0.308324
Q8TDN6	Ribosome biogenesis protein BRX1 homolog OS=Homo sapiens OX=9606 GN=BRIX1 PE=1 SV=2	BRIX1	1.133	0.523844
Q8TDW0	Volume-regulated anion channel subunit LRRC8C OS=Homo sapiens OX=9606 GN=LRRC8C PE=1 SV=2	LRRC8C	1.099	0.40911
Q8TDX7	Serine/threonine-protein kinase Nek7 OS=Homo sapiens OX=9606 GN=NEK7 PE=1 SV=1	NEK7	1.191	0.122073
Q8TEA8	D-aminoacyl-tRNA deacylase 1 OS=Homo sapiens OX=9606 GN=DTD1 PE=1 SV=2	DTD1	1.003	0.938017
Q8TED0	U3 small nucleolar RNA-associated protein 15 homolog OS=Homo sapiens OX=9606 GN=UTP15 PE=1 SV=3	UTP15	0.938	0.189689
Q8TED1	Probable glutathione peroxidase 8 OS=Homo sapiens OX=9606 GN=GPX8 PE=1 SV=2	GPX8	1.018	0.677835

Q8TED4	Glucose-6-phosphate exchanger SLC37A2 OS=Homo sapiens OX=9606 GN=SLC37A2 PE=2 SV=2	SLC37A2	1.138	0.43887
Q8TEM1	Nuclear pore membrane glycoprotein 210 OS=Homo sapiens OX=9606 GN=NUP210	NUP210	0.871	0.007102
Q8TEQ6	Gem-associated protein 5 OS=Homo sapiens OX=9606 GN=GEMIN5 PE=1 SV=3	GEMIN5	1.172	0.133866
Q8TEX9	Importin-4 OS=Homo sapiens OX=9606 GN=IPO4 PE=1 SV=2	IPO4	1.012	0.866188
Q8TF05	Serine/threonine-protein phosphatase 4 regulatory subunit 1 OS=Homo sapiens OX=9606 GN=PPP4R1 PE=1 SV=1	PPP4R1	1.253	0.183736
Q8TF68	Zinc finger protein 384 OS=Homo sapiens OX=9606 GN=ZNF384 PE=1 SV=2	ZNF384	0.897	0.370926
Q8WTT2	Nucleolar complex protein 3 homolog OS=Homo sapiens OX=9606 GN=NOC3L PE=1	NOC3L	0.927	0.540108
Q8WTV0	Scavenger receptor class B member 1 OS=Homo sapiens OX=9606 GN=SCARB1 PE=1	SCARB1	1.06	0.39341
Q8WU90	Zinc finger CCCH domain-containing protein 15 OS=Homo sapiens OX=9606 GN=ZC3H15 PE=1 SV=1	ZC3H15	0.943	0.553488
Q8WUA2	Peptidyl-prolyl cis-trans isomerase-like 4 OS=Homo sapiens OX=9606 GN=PPIL4	PPIL4	0.994	0.992274
Q8WUA4	General transcription factor 3C polypeptide 2 OS=Homo sapiens OX=9606 GN=GTF3C2 PE=1	GTF3C2	1.166	0.211583
Q8WUD4	Coiled-coil domain-containing protein 12 OS=Homo sapiens OX=9606 GN=CCDC12 PE=1 SV=1	CCDC12	0.956	0.707145
Q8WUF5	RelA-associated inhibitor OS=Homo sapiens OX=9606 GN=PPP1R13L PE=1 SV=4	PPP1R13L	1.105	0.193159
Q8WUH6	Transmembrane protein 263 OS=Homo sapiens OX=9606 GN=TMEM263 PE=1 SV=1	TMEM263	1.09	0.358186
Q8WUK0	Phosphatidylglycerophosphatase and protein-tyrosine phosphatase 1 OS=Homo sapiens OX=9606 GN=PTPMT1 PE=1 SV=1	PTPMT1	0.963	0.461935
Q8WUM0	Nuclear pore complex protein Nup133 OS=Homo sapiens OX=9606 GN=NUP133 PE=1	NUP133	1.015	0.373294

Q8WUM4	Programmed cell death 6-interacting protein OS=Homo sapiens OX=9606 GN=PDCD6IP	PDCD6IP	1.119	0.082731
Q8WUM9	Sodium-dependent phosphate transporter 1 OS=Homo sapiens OX=9606 GN=SLC20A1 PE=1 SV=1	SLC20A1	0.958	
Q8WUP2	Filamin-binding LIM protein 1 OS=Homo sapiens OX=9606 GN=FBLIM1 PE=1 SV=2	FBLIM1	1.011	
Q8WUU5	GATA zinc finger domain-containing protein 1 OS=Homo sapiens OX=9606 GN=GATAD1 PE=1 SV=1	GATAD1	0.971	0.834598
Q8WUW1	Protein BRICK1 OS=Homo sapiens OX=9606 GN=BRK1	BRK1	1.323	0.164664
Q8WUY1	Protein THEM6 OS=Homo sapiens OX=9606 GN=THEM6	THEM6	0.927	0.558218
Q8WV24	Pleckstrin homology-like domain family A member 1 OS=Homo sapiens OX=9606 GN=PHLDA1 PE=1 SV=4	PHLDA1	0.912	0.354798
Q8WVB6	Chromosome transmission fidelity protein 18 homolog OS=Homo sapiens OX=9606 GN=CHTF18 PE=1 SV=1	CHTF18	0.681	0.080209
Q8WVC0	RNA polymerase-associated protein LEO1 OS=Homo sapiens OX=9606 GN=LEO1 PE=1 SV=1	LEO1	0.85	0.005462
Q8WVC6	Dephospho-CoA kinase domain-containing protein OS=Homo sapiens OX=9606 GN=DCAKD PE=1 SV=1	DCAKD	1.036	0.511828
Q8WVM7	Cohesin subunit SA-1 OS=Homo sapiens OX=9606 GN=STAG1 PE=1 SV=3	STAG1	0.961	0.838882
Q8WVM8	Sec1 family domain-containing protein 1 OS=Homo sapiens OX=9606 GN=SCFD1 PE=1	SCFD1	0.974	0.583731
Q8WVQ1	Soluble calcium-activated nucleotidase 1 OS=Homo sapiens OX=9606 GN=CANT1 PE=1	CANT1	1.028	0.834335
Q8WVV4	Protein POF1B OS=Homo sapiens OX=9606 GN=POF1B PE=1	POF1B	0.969	0.57123
Q8WVV9	Heterogeneous nuclear ribonucleoprotein L-like OS=Homo sapiens OX=9606 GN=HNRNPLL PE=1 SV=1	HNRNPLL	0.901	0.090348
Q8WVX9	Fatty acyl-CoA reductase 1 OS=Homo sapiens OX=9606 GN=FAR1 PE=1 SV=1	FAR1	0.901	0.492874

Q8WVY7	Ubiquitin-like domain-containing CTD phosphatase 1 OS=Homo sapiens OX=9606 GN=UBLCP1 PE=1 SV=2	UBLCP1	0.914	0.567776
Q8WW12	PEST proteolytic signal-containing nuclear protein OS=Homo sapiens OX=9606 GN=PCNP PE=1 SV=2	PCNP	0.734	0.065945
Q8WW59	SPRY domain-containing protein 4 OS=Homo sapiens OX=9606 GN=SPRYD4 PE=1 SV=2	SPRYD4	1.111	0.319775
Q8WWA1	Transmembrane protein 40 OS=Homo sapiens OX=9606 GN=TMEM40 PE=1 SV=2	TMEM40	0.985	0.835089
Q8WWC4	m-AAA protease-interacting protein 1, mitochondrial OS=Homo sapiens OX=9606	MAIP1	1.002	0.987141
Q8WWH5	Probable tRNA pseudouridine synthase 1 OS=Homo sapiens OX=9606 GN=TRUB1 PE=1	TRUB1	1.07	
Q8WWI1	LIM domain only protein 7 OS=Homo sapiens OX=9606 GN=LMO7 PE=1 SV=3	LMO7	0.968	0.594506
Q8WWI5	Choline transporter-like protein 1 OS=Homo sapiens OX=9606 GN=SLC44A1 PE=1 SV=1	SLC44A1	0.969	0.666973
Q8WWM7	Ataxin-2-like protein OS=Homo sapiens OX=9606 GN=ATXN2L PE=1 SV=2	ATXN2L	1.038	0.633192
Q8WWV3	Reticulon-4-interacting protein 1, mitochondrial OS=Homo sapiens OX=9606 GN=RTN4IP1 PE=1 SV=2	RTN4IP1	1.223	0.262735
Q8WWY3	U4/U6 small nuclear ribonucleoprotein Prp31 OS=Homo sapiens OX=9606 GN=PRPF31 PE=1 SV=2	PRPF31	0.99	0.889106
Q8WX92	Negative elongation factor B OS=Homo sapiens OX=9606 GN=NELFB PE=1 SV=1	NELFB	0.921	0.279617
Q8WX93	Palladin OS=Homo sapiens OX=9606 GN=PALLD PE=1	PALLD	1.037	0.559694
Q8WXA9	Splicing regulatory glutamine/lysine-rich protein 1 OS=Homo sapiens OX=9606	SREK1	0.914	
Q8WXE9	Stonin-2 OS=Homo sapiens OX=9606 GN=STON2 PE=1	STON2	1.134	0.026757
Q8WXF1	Paraspeckle component 1 OS=Homo sapiens OX=9606 GN=PSPC1 PE=1 SV=1	PSPC1	0.903	0.053088
Q8WXH0	Nesprin-2 OS=Homo sapiens OX=9606 GN=SYNE2 PE=1	SYNE2	0.89	0.28841

Q8WXI9	Transcriptional repressor p66-beta OS=Homo sapiens OX=9606 GN=GATAD2B PE=1 SV=1	GATAD2B	0.836	0.052025
Q8WXX5	DnaJ homolog subfamily C member 9 OS=Homo sapiens OX=9606 GN=DNAJC9 PE=1	DNAJC9	0.954	0.378409
Q8WY22	BRI3-binding protein OS=Homo sapiens OX=9606 GN=BRI3BP PE=1 SV=1	BRI3BP	0.949	0.236646
Q8WYA6	Beta-catenin-like protein 1 OS=Homo sapiens OX=9606 GN=CTNNBL1 PE=1 SV=1	CTNNBL1	1.001	0.986496
Q8WYH8	Inhibitor of growth protein 5 OS=Homo sapiens OX=9606 GN=ING5 PE=1 SV=1	ING5	0.952	0.712805
Q8WYP5	Protein ELYS OS=Homo sapiens OX=9606 GN=AHCTF1 PE=1 SV=3	AHCTF1	1.003	0.962234
Q8WZ42	Titin OS=Homo sapiens OX=9606 GN=TTN PE=1 SV=4	TTN	1.373	
Q92466	DNA damage-binding protein 2 OS=Homo sapiens OX=9606 GN=DDB2 PE=1 SV=1	DDB2	0.984	0.839422
Q92499	ATP-dependent RNA helicase DDX1 OS=Homo sapiens OX=9606 GN=DDX1 PE=1 SV=2	DDX1	1.026	0.679576
Q92504	Zinc transporter SLC39A7 OS=Homo sapiens OX=9606 GN=SLC39A7 PE=1 SV=2	SLC39A7	1.229	0.041878
Q92508	Piezo-type mechanosensitive ion channel component 1 OS=Homo sapiens OX=9606 GN=PIEZO1 PE=1 SV=4	PIEZO1	1.202	0.108447
Q92520	Protein FAM3C OS=Homo sapiens OX=9606 GN=FAM3C	FAM3C	1.015	0.865973
Q92522	Histone H1.10 OS=Homo sapiens OX=9606 GN=H1-10 PE=1 SV=1	H1-10	1.238	0.595875
Q92530	Proteasome inhibitor PI31 subunit OS=Homo sapiens OX=9606 GN=PSMF1 PE=1 SV=2	PSMF1	1.089	0.504653
Q92538	Golgi-specific brefeldin A-resistance guanine nucleotide exchange factor 1 OS=Homo sapiens OX=9606 GN=GBF1	GBF1	1.147	0.454469
Q92541	RNA polymerase-associated protein RTF1 homolog OS=Homo sapiens OX=9606 GN=RTF1	RTF1	0.777	0.042205
Q92542	Nicastrin OS=Homo sapiens OX=9606 GN=NCSTN PE=1	NCSTN	1.124	0.041636
Q92544	Transmembrane 9 superfamily member 4 OS=Homo sapiens OX=9606 GN=TM9SF4 PE=1	TM9SF4	0.961	0.697865

Q92552	28S ribosomal protein S27, mitochondrial OS=Homo sapiens OX=9606 GN=MRPS27 PE=1 SV=3	MRPS27	0.958	0.591838
Q92572	AP-3 complex subunit sigma-1 OS=Homo sapiens OX=9606 GN=AP3S1 PE=1 SV=1	AP3S1	0.863	0.54621
Q92575	UBX domain-containing protein 4 OS=Homo sapiens OX=9606 GN=UBXN4 PE=1 SV=2	UBXN4	0.824	0.062245
Q92576	PHD finger protein 3 OS=Homo sapiens OX=9606 GN=PHF3 PE=1 SV=3	PHF3	0.906	0.036058
Q92597	Protein NDRG1 OS=Homo sapiens OX=9606 GN=NDRG1	NDRG1	1.078	0.475111
Q92598	Heat shock protein 105 kDa OS=Homo sapiens OX=9606 GN=HSPH1 PE=1 SV=1	HSPH1	0.986	0.850077
Q92599	Septin-8 OS=Homo sapiens OX=9606 GN=SEPTIN8 PE=1 SV=4	SEPTIN8	0.989	0.848884
Q92600	CCR4-NOT transcription complex subunit 9 OS=Homo sapiens OX=9606 GN=CNOT9 PE=1	CNOT9	1.002	0.887888
Q92604	Acyl-CoA:lysophosphatidylglycerol acyltransferase 1 OS=Homo sapiens OX=9606 GN=LPGAT1	LPGAT1	1.179	0.401328
Q92609	TBC1 domain family member 5 OS=Homo sapiens OX=9606 GN=TBC1D5 PE=1 SV=1	TBC1D5	1000	0.001
Q92614	Unconventional myosin-XVIIIa OS=Homo sapiens OX=9606 GN=MYO18A PE=1 SV=3	MYO18A	1.004	0.928027
Q92615	La-related protein 4B OS=Homo sapiens OX=9606 GN=LARP4B PE=1 SV=3	LARP4B	0.962	0.286295
Q92616	eIF-2-alpha kinase activator GCN1 OS=Homo sapiens OX=9606 GN=GCN1 PE=1 SV=6	GCN1	1.159	0.146712
Q92620	Pre-mRNA-splicing factor ATP-dependent RNA helicase PRP16 OS=Homo sapiens OX=9606 GN=DHX38 PE=1 SV=2	DHX38	0.928	0.489981
Q92621	Nuclear pore complex protein Nup205 OS=Homo sapiens OX=9606 GN=NUP205 PE=1	NUP205	0.982	0.766803
Q92643	GPI-anchor transamidase OS=Homo sapiens OX=9606 GN=PIGK PE=1 SV=2	PIGK	0.947	0.677659

Q92665	28S ribosomal protein S31, mitochondrial OS=Homo sapiens OX=9606 GN=MRPS31 PE=1 SV=3	MRPS31	0.902	0.265504
Q92667	A-kinase anchor protein 1, mitochondrial OS=Homo sapiens OX=9606 GN=AKAP1 PE=1	AKAP1	1.109	0.431642
Q92673	Sortilin-related receptor OS=Homo sapiens OX=9606 GN=SORL1 PE=1 SV=2	SORL1	1.032	0.223284
Q92685	Dol-P-Man:Man(5)GlcNAc(2)-PP-Dol alpha-1,3-mannosyltransferase OS=Homo sapiens OX=9606 GN=ALG3 PE=1 SV=1	ALG3	0.968	
Q92688	Acidic leucine-rich nuclear phosphoprotein 32 family member B OS=Homo sapiens OX=9606 GN=ANP32B PE=1 SV=1	ANP32B	0.818	0.147742
Q92692	Nectin-2 OS=Homo sapiens OX=9606 GN=NECTIN2 PE=1 SV=1	NECTIN2	1.104	0.125974
Q92734	Protein TFG OS=Homo sapiens OX=9606 GN=TFG PE=1 SV=2	TFG	1.135	0.148418
Q92747	Actin-related protein 2/3 complex subunit 1A OS=Homo sapiens OX=9606 GN=ARPC1A PE=1 SV=2	ARPC1A	1.062	0.367275
Q92759	General transcription factor IIH subunit 4 OS=Homo sapiens OX=9606 GN=GTF2H4 PE=1 SV=1	GTF2H4	0.966	0.828956
Q92769	Histone deacetylase 2 OS=Homo sapiens OX=9606 GN=HDAC2 PE=1 SV=2	HDAC2	0.956	0.250957
Q92783	Signal transducing adapter molecule 1 OS=Homo sapiens OX=9606 GN=STAM PE=1 SV=3	STAM	0.868	0.315711
Q92785	Zinc finger protein ubi-d4 OS=Homo sapiens OX=9606 GN=DPF2 PE=1 SV=2	DPF2	0.94	0.31441
Q92791	Endoplasmic reticulum protein SC65 OS=Homo sapiens OX=9606 GN=P3H4 PE=1 SV=1	P3H4	1.217	0.563486
Q92796	Disks large homolog 3 OS=Homo sapiens OX=9606 GN=DLG3 PE=1 SV=2	DLG3	0.867	0.727929
Q92797	Symplekin OS=Homo sapiens OX=9606 GN=SYMPK PE=1	SYMPK	0.907	0.058881
Q92804	TATA-binding protein-associated factor 2N OS=Homo sapiens OX=9606 GN=TAF15 PE=1	TAF15	0.862	0.008526
Q92817	Envoplakin OS=Homo sapiens OX=9606 GN=EVPL PE=1 SV=3	EVPL	1.062	0.519723

Q92820	Gamma-glutamyl hydrolase OS=Homo sapiens OX=9606 GN=GGH PE=1 SV=2	GGH	1.104	0.51529
Q92828	Coronin-2A OS=Homo sapiens OX=9606 GN=CORO2A PE=1 SV=2	CORO2A	0.866	
Q92841	Probable ATP-dependent RNA helicase DDX17 OS=Homo sapiens OX=9606 GN=DDX17	DDX17	0.898	0.000587
Q92859	Neogenin OS=Homo sapiens OX=9606 GN=NEO1 PE=1 SV=2	NEO1	1.023	0.851902
Q92876	Kallikrein-6 OS=Homo sapiens OX=9606 GN=KLK6 PE=1 SV=1	KLK6	1.169	0.363251
Q92878	DNA repair protein RAD50 OS=Homo sapiens OX=9606 GN=RAD50 PE=1 SV=1	RAD50	0.895	0.000704
Q92879	CUGBP Elav-like family member 1 OS=Homo sapiens OX=9606 GN=CELF1 PE=1 SV=2	CELF1	0.902	0.027745
Q92882	Osteoclast-stimulating factor 1 OS=Homo sapiens OX=9606 GN=OSTF1 PE=1 SV=2	OSTF1	1.009	0.913362
Q92890	Ubiquitin recognition factor in ER-associated degradation protein 1 OS=Homo sapiens OX=9606 GN=UFD1 PE=1 SV=3	UFD1	1.141	0.146507
Q92896	Golgi apparatus protein 1 OS=Homo sapiens OX=9606 GN=GLG1 PE=1 SV=2	GLG1	1.067	0.118765
Q92900	Regulator of nonsense transcripts 1 OS=Homo sapiens OX=9606 GN=UPF1 PE=1 SV=2	UPF1	0.991	0.829942
Q92903	Phosphatidate cytidylyltransferase 1 OS=Homo sapiens OX=9606 GN=CDS1 PE=1 SV=2	CDS1	1.059	
Q92905	COP9 signalosome complex subunit 5 OS=Homo sapiens OX=9606 GN=COPS5 PE=1	COPS5	0.89	0.430377
Q92917	G-patch domain and KOW motifs-containing protein OS=Homo sapiens OX=9606 GN=GPKOW PE=1 SV=2	GPKOW	0.885	0.197536
Q92922	SWI/SNF complex subunit SMARCC1 OS=Homo sapiens OX=9606 GN=SMARCC1 PE=1 SV=3	SMARCC1	0.963	0.158087
Q92925	SWI/SNF-related matrix-associated actin-dependent regulator of chromatin subfamily D member 2 OS=Homo sapiens OX=9606 GN=SMARCD2 PE=1	SMARCD2	0.99	0.745339

Q92930	Ras-related protein Rab-8B OS=Homo sapiens OX=9606 GN=RAB8B PE=1 SV=2	RAB8B	0.925	0.437785
Q92945	Far upstream element-binding protein 2 OS=Homo sapiens OX=9606 GN=KHSRP PE=1	KHSRP	0.9	0.205656
Q92968	Peroxisomal membrane protein PEX13 OS=Homo sapiens OX=9606 GN=PEX13 PE=1	PEX13	0.767	
Q92973	Transportin-1 OS=Homo sapiens OX=9606 GN=TNPO1 PE=1	TNPO1	1.1	0.368484
Q92974	Rho guanine nucleotide exchange factor 2 OS=Homo sapiens OX=9606 GN=ARHGEF2 PE=1 SV=4	ARHGEF2	1.165	0.407093
Q92979	Ribosomal RNA small subunit methyltransferase NEP1 OS=Homo sapiens OX=9606 GN=EMG1 PE=1 SV=4	EMG1	0.897	0.116122
Q93009	Ubiquitin carboxyl-terminal hydrolase 7 OS=Homo sapiens OX=9606 GN=USP7 PE=1 SV=2	USP7	0.96	0.242365
Q93034	Cullin-5 OS=Homo sapiens OX=9606 GN=CUL5 PE=1 SV=4	CUL5	0.937	0.555232
Q93045	Stathmin-2 OS=Homo sapiens OX=9606 GN=STMN2 PE=1	STMN2	0.977	0.741843
Q93050	V-type proton ATPase 116 kDa subunit a1 OS=Homo sapiens OX=9606 GN=ATP6V0A1 PE=1 SV=3	ATP6V0A 1	0.991	0.868986
Q93052	Lipoma-preferred partner OS=Homo sapiens OX=9606 GN=LPP PE=1 SV=1	LPP	1.229	0.036971
Q93063	Exostosin-2 OS=Homo sapiens OX=9606 GN=EXT2 PE=1 SV=1	EXT2	1.302	
Q93096	Protein tyrosine phosphatase type IVA 1 OS=Homo sapiens OX=9606 GN=PTP4A1 PE=1	PTP4A1	0.992	0.903641
Q969E2	Secretory carrier-associated membrane protein 4 OS=Homo sapiens OX=9606 GN=SCAMP4 PE=1 SV=1	SCAMP4	0.974	0.660295
Q969G3	SWI/SNF-related matrix-associated actin-dependent regulator of chromatin subfamily E member 1 OS=Homo sapiens OX=9606 GN=SMARCE1 PE=1	SMARCE1	0.976	0.756778
Q969H8	Myeloid-derived growth factor OS=Homo sapiens OX=9606 GN=MYDGF PE=1 SV=1	MYDGF	1.01	0.950413
Q969M3	Protein YIPF5 OS=Homo sapiens OX=9606 GN=YIPF5 PE=1 SV=1	YIPF5	0.9	0.354901

Q969N2	GPI transamidase component PIG-T OS=Homo sapiens OX=9606 GN=PIGT PE=1 SV=1	PIGT	1.054	0.281684
Q969P0	Immunoglobulin superfamily member 8 OS=Homo sapiens OX=9606 GN=IGSF8 PE=1 SV=1	IGSF8	0.858	
Q969Q0	60S ribosomal protein L36a-like OS=Homo sapiens OX=9606 GN=RPL36AL PE=1 SV=3	RPL36AL	1.242	0.36734
Q969R2	Oxysterol-binding protein 2 OS=Homo sapiens OX=9606 GN=OSBP2 PE=1 SV=2	OSBP2	0.001	0.001
Q969S9	Ribosome-releasing factor 2, mitochondrial OS=Homo sapiens OX=9606 GN=GFM2 PE=1 SV=1	GFM2	1.117	0.244051
Q969T9	WW domain-binding protein 2 OS=Homo sapiens OX=9606 GN=WBP2 PE=1 SV=1	WBP2	0.853	0.178819
Q969V3	Nicalin OS=Homo sapiens OX=9606 GN=NCLN PE=1 SV=2	NCLN	1.065	0.255412
Q969W9	Protein TMEPAI OS=Homo sapiens OX=9606 GN=PMEPA1 PE=1 SV=1	PMEPA1	0.982	0.976164
Q969X1	Protein lifeguard 3 OS=Homo sapiens OX=9606 GN=TMBIM1 PE=1 SV=2	TMBIM1	0.917	0.549336
Q969X5	Endoplasmic reticulum-Golgi intermediate compartment protein 1 OS=Homo sapiens OX=9606 GN=ERGIC1 PE=1 SV=1	ERGIC1	0.959	0.259708
Q969X6	U3 small nucleolar RNA-associated protein 4 homolog OS=Homo sapiens OX=9606 GN=UTP4 PE=1 SV=1	UTP4	0.998	0.918256
Q969Y2	tRNA modification GTPase GTPBP3, mitochondrial OS=Homo sapiens OX=9606 GN=GTPBP3 PE=1 SV=2	GTPBP3	1.032	
Q969Z0	FAST kinase domain-containing protein 4 OS=Homo sapiens OX=9606 GN=TBRG4 PE=1	TBRG4	0.934	0.340394
Q96A26	Protein FAM162A OS=Homo sapiens OX=9606 GN=FAM162A PE=1 SV=2	FAM162A	0.925	0.236138
Q96A33	PAT complex subunit CCDC47 OS=Homo sapiens OX=9606 GN=CCDC47 PE=1 SV=1	CCDC47	0.991	0.846546
Q96A35	39S ribosomal protein L24, mitochondrial OS=Homo sapiens OX=9606 GN=MRPL24 PE=1 SV=1	MRPL24	1.138	0.214504

Q96A49	Synapse-associated protein 1 OS=Homo sapiens OX=9606 GN=SYAP1 PE=1 SV=1	SYAP1	0.997	0.95012
Q96A57	Transmembrane protein 230 OS=Homo sapiens OX=9606 GN=TMEM230 PE=1 SV=1	TMEM230	0.984	
Q96A72	Protein mago nashi homolog 2 OS=Homo sapiens OX=9606 GN=MAGOHB PE=1 SV=1	MAGOHB	0.973	0.382937
Q96AB3	Isochorismatase domain-containing protein 2 OS=Homo sapiens OX=9606 GN=ISOC2	ISOC2	1.197	0.037879
Q96AC1	Fermitin family homolog 2 OS=Homo sapiens OX=9606 GN=FERMT2 PE=1 SV=1	FERMT2	1.235	0.393805
Q96AE4	Far upstream element-binding protein 1 OS=Homo sapiens OX=9606 GN=FUBP1 PE=1	FUBP1	0.82	0.07841
Q96AG4	Leucine-rich repeat-containing protein 59 OS=Homo sapiens OX=9606 GN=LRRC59 PE=1 SV=1	LRRC59	1.037	0.693663
Q96AJ9	Vesicle transport through interaction with t-SNAREs homolog 1A OS=Homo sapiens OX=9606 GN=VTI1A PE=1 SV=2	VTI1A	1.227	0.112405
Q96AQ6	Pre-B-cell leukemia transcription factor-interacting protein 1 OS=Homo sapiens OX=9606 GN=PBXIP1 PE=1 SV=1	PBXIP1	0.95	0.640436
Q96AY3	Peptidyl-prolyl cis-trans isomerase FKBP10 OS=Homo sapiens OX=9606 GN=FKBP10 PE=1	FKBP10	1.017	0.758666
Q96B26	Exosome complex component RRP43 OS=Homo sapiens OX=9606 GN=EXOSC8 PE=1 SV=1	EXOSC8	0.937	0.348002
Q96B49	Mitochondrial import receptor subunit TOM6 homolog OS=Homo sapiens OX=9606 GN=TOMM6 PE=1 SV=1	TOMM6	1.031	
Q96BK5	PIN2/TERF1-interacting telomerase inhibitor 1 OS=Homo sapiens OX=9606 GN=PINX1	PINX1	0.891	0.426756
Q96BP3	Peptidylprolyl isomerase domain and WD repeat-containing protein 1 OS=Homo sapiens OX=9606 GN=PPWD1 PE=1 SV=1	PPWD1	0.93	0.501396
Q96BR5	Cytochrome c oxidase assembly factor 7 OS=Homo sapiens OX=9606 GN=COA7 PE=1 SV=2	COA7	1.02	0.894781

Q96BW9	Phosphatidate cytidylyltransferase, mitochondrial OS=Homo sapiens OX=9606 GN=TAMM41 PE=1 SV=2	TAMM41	0.936	0.345596
Q96C01	Protein FAM136A OS=Homo sapiens OX=9606 GN=FAM136A PE=1 SV=1	FAM136A	0.906	0.07471
Q96C19	EF-hand domain-containing protein D2 OS=Homo sapiens OX=9606 GN=EFHD2 PE=1	EFHD2	0.995	0.929015
Q96C36	Pyrroline-5-carboxylate reductase 2 OS=Homo sapiens OX=9606 GN=PYCR2 PE=1 SV=1	PYCR2	0.903	0.241763
Q96C57	Protein CUSTOS OS=Homo sapiens OX=9606 GN=CUSTOS PE=1 SV=2	CUSTOS	1	0.974245
Q96C86	m7GpppX diphosphatase OS=Homo sapiens OX=9606 GN=DCPS PE=1 SV=2	DCPS	0.926	0.569958
Q96CB9	5-methylcytosine rRNA methyltransferase NSUN4 OS=Homo sapiens OX=9606 GN=NSUN4 PE=1 SV=2	NSUN4	1.054	
Q96CM8	Medium-chain acyl-CoA ligase ACSF2, mitochondrial OS=Homo sapiens OX=9606 GN=ACSF2 PE=1 SV=2	ACSF2	1.088	0.294306
Q96CN7	Isochorismatase domain-containing protein 1 OS=Homo sapiens OX=9606 GN=ISOC1	ISOC1	1.109	0.070086
Q96CP2	FLYWCH family member 2 OS=Homo sapiens OX=9606 GN=FLYWCH2 PE=1 SV=1	FLYWCH2	0.001	0.001
Q96CP7	TLC domain-containing protein 1 OS=Homo sapiens OX=9606 GN=TLCD1 PE=1 SV=1	TLCD1	0.708	0.101624
Q96CS3	FAS-associated factor 2 OS=Homo sapiens OX=9606 GN=FAF2 PE=1 SV=2	FAF2	1.035	0.032607
Q96CT7	Coiled-coil domain-containing protein 124 OS=Homo sapiens OX=9606 GN=CCDC124 PE=1 SV=1	CCDC124	1.019	
Q96CU9	FAD-dependent oxidoreductase domain-containing protein 1 OS=Homo sapiens OX=9606 GN=FOXRED1 PE=1 SV=2	FOXRED1	0.987	0.86194
Q96CW1	AP-2 complex subunit mu OS=Homo sapiens OX=9606 GN=AP2M1 PE=1 SV=2	AP2M1	0.962	0.651895

Q96CX2	BTB/POZ domain-containing protein KCTD12 OS=Homo sapiens OX=9606 GN=KCTD12 PE=1 SV=1	KCTD12	0.872	
Q96D46	60S ribosomal export protein NMD3 OS=Homo sapiens OX=9606 GN=NMD3 PE=1 SV=1	NMD3	0.915	0.554652
Q96D53	Atypical kinase COQ8B, mitochondrial OS=Homo sapiens OX=9606 GN=COQ8B PE=1	COQ8B	1.015	0.949095
Q96DA6	Mitochondrial import inner membrane translocase subunit TIM14 OS=Homo sapiens OX=9606 GN=DNAJC19 PE=1	DNAJC19	1.013	0.850172
Q96DB5	Regulator of microtubule dynamics protein 1 OS=Homo sapiens OX=9606 GN=RMDN1 PE=1	RMDN1	1.02	0.479724
Q96DH6	RNA-binding protein Musashi homolog 2 OS=Homo sapiens OX=9606 GN=MSI2 PE=1 SV=1	MSI2	1.02	0.87247
Q96DI7	U5 small nuclear ribonucleoprotein 40 kDa protein OS=Homo sapiens OX=9606 GN=SNRNP40 PE=1 SV=1	SNRNP40	0.93	0.21072
Q96DV4	39S ribosomal protein L38, mitochondrial OS=Homo sapiens OX=9606 GN=MRPL38 PE=1 SV=2	MRPL38	0.946	0.425372
Q96DZ1	Endoplasmic reticulum lectin 1 OS=Homo sapiens OX=9606 GN=ERLEC1 PE=1 SV=1	ERLEC1	1.175	0.131861
Q96E11	Ribosome-recycling factor, mitochondrial OS=Homo sapiens OX=9606 GN=MRRF PE=1 SV=1	MRRF	1.085	0.694908
Q96E39	RNA binding motif protein, X-linked-like-1 OS=Homo sapiens OX=9606 GN=RBMXL1 PE=1 SV=1	RBMXL1	1.336	
Q96EC8	Protein YIPF6 OS=Homo sapiens OX=9606 GN=YIPF6 PE=1 SV=2	YIPF6	1.028	
Q96EE3	Nucleoporin SEH1 OS=Homo sapiens OX=9606 GN=SEH1L PE=1 SV=3	SEH1L	1.074	0.263841
Q96EH3	Mitochondrial assembly of ribosomal large subunit protein 1 OS=Homo sapiens OX=9606 GN=MALSU1 PE=1 SV=1	MALSU1	0.863	0.632734
Q96EK6	Glucosamine 6-phosphate N-acetyltransferase OS=Homo sapiens OX=9606 GN=GNPNAT1 PE=1 SV=1	GNPNAT1	1.007	0.996424

Q96EL2	28S ribosomal protein S24, mitochondrial OS=Homo sapiens OX=9606 GN=MRPS24 PE=1 SV=1	MRPS24	1.097	0.440569
Q96EL3	39S ribosomal protein L53, mitochondrial OS=Homo sapiens OX=9606 GN=MRPL53 PE=1 SV=1	MRPL53	1.053	0.569123
Q96EP5	DAZ-associated protein 1 OS=Homo sapiens OX=9606 GN=DAZAP1 PE=1 SV=1	DAZAP1	0.867	0.077825
Q96ER9	Mitochondrial potassium channel OS=Homo sapiens OX=9606 GN=CCDC51 PE=1 SV=2	CCDC51	1.162	0.278288
Q96ES7	SAGA-associated factor 29 OS=Homo sapiens OX=9606 GN=SGF29 PE=1 SV=1	SGF29	1.055	
Q96EU6	Ribosomal RNA processing protein 36 homolog OS=Homo sapiens OX=9606 GN=RRP36	RRP36	1.182	0.07142
Q96EU7	C1GALT1-specific chaperone 1 OS=Homo sapiens OX=9606 GN=C1GALT1C1 PE=1 SV=1	C1GALT1C1	0.952	0.557379
Q96EX1	Small integral membrane protein 12 OS=Homo sapiens OX=9606 GN=SMIM12 PE=1 SV=3	SMIM12	1.246	0.050027
Q96EY1	DnaJ homolog subfamily A member 3, mitochondrial OS=Homo sapiens OX=9606 GN=DNAJA3 PE=1 SV=2	DNAJA3	1.024	0.736763
Q96EY4	Translation machinery-associated protein 16 OS=Homo sapiens OX=9606 GN=TMA16 PE=1	TMA16	0.759	0.017421
Q96EY7	Pentatricopeptide repeat domain-containing protein 3, mitochondrial OS=Homo sapiens OX=9606 GN=PTCD3 PE=1 SV=3	PTCD3	1.011	0.765011
Q96EY8	Corrinoid adenosyltransferase OS=Homo sapiens OX=9606 GN=MMAB PE=1 SV=1	MMAB	1.34	0.055989
Q96FM1	Post-GPI attachment to proteins factor 3 OS=Homo sapiens OX=9606 GN=PGAP3 PE=1	PGAP3	0.853	0.355729
Q96FQ6	Protein S100-A16 OS=Homo sapiens OX=9606 GN=S100A16 PE=1 SV=1	S100A16	1.011	0.989108
Q96FV9	THO complex subunit 1 OS=Homo sapiens OX=9606 GN=THOC1 PE=1 SV=1	THOC1	0.927	0.288509
Q96FW1	Ubiquitin thioesterase OTUB1 OS=Homo sapiens OX=9606 GN=OTUB1 PE=1 SV=2	OTUB1	0.948	0.566182

Q96FX7	tRNA (adenine(58)-N(1))-methyltransferase catalytic subunit TRMT61A OS=Homo sapiens OX=9606 GN=TRMT61A PE=1 SV=1	TRMT61A	0.876	0.229946
Q96FX8	p53 apoptosis effector related to PMP-22 OS=Homo sapiens OX=9606 GN=PERP PE=1 SV=1	PERP	1.434	0.254683
Q96FZ2	Abasic site processing protein HMCES OS=Homo sapiens OX=9606 GN=HMCES PE=1	HMCES	0.899	
Q96FZ7	Charged multivesicular body protein 6 OS=Homo sapiens OX=9606 GN=CHMP6 PE=1	CHMP6	0.937	0.707981
Q96G03	Phosphoglucomutase-2 OS=Homo sapiens OX=9606 GN=PGM2 PE=1 SV=4	PGM2	0.953	0.675391
Q96G21	U3 small nucleolar ribonucleoprotein protein IMP4 OS=Homo sapiens OX=9606	IMP4	1.104	0.549726
Q96G23	Ceramide synthase 2 OS=Homo sapiens OX=9606 GN=CERS2 PE=1 SV=1	CERS2	1.194	0.326843
Q96G25	Mediator of RNA polymerase II transcription subunit 8 OS=Homo sapiens OX=9606 GN=MED8 PE=1 SV=2	MED8	1.063	0.068897
Q96GC5	39S ribosomal protein L48, mitochondrial OS=Homo sapiens OX=9606 GN=MRPL48 PE=1 SV=2	MRPL48	0.916	0.285508
Q96GC9	Vacuole membrane protein 1 OS=Homo sapiens OX=9606 GN=VMP1 PE=1 SV=1	VMP1	1.008	0.935632
Q96GD4	Aurora kinase B OS=Homo sapiens OX=9606 GN=AURKB	AURKB	0.897	0.143223
Q96GF1	E3 ubiquitin-protein ligase RNF185 OS=Homo sapiens OX=9606 GN=RNF185 PE=1	RNF185	0.784	0.225461
Q96GG9	DCN1-like protein 1 OS=Homo sapiens OX=9606 GN=DCUN1D1 PE=1 SV=1	DCUN1D1	0.948	0.760368
Q96GM5	SWI/SNF-related matrix-associated actin-dependent regulator of chromatin subfamily D member 1 OS=Homo sapiens OX=9606 GN=SMARCD1 PE=1	SMARCD1	0.998	0.953313
Q96GQ5	RUS family member 1 OS=Homo sapiens OX=9606 GN=RUSF1 PE=1 SV=2	RUSF1	1.106	
Q96GQ7	Probable ATP-dependent RNA helicase DDX27 OS=Homo sapiens OX=9606 GN=DDX27	DDX27	0.935	0.148653

Q96GX5	Serine/threonine-protein kinase greatwall OS=Homo sapiens OX=9606 GN=MASTL PE=1	MASTL	1.091	0.556409
Q96HA7	Tonsoku-like protein OS=Homo sapiens OX=9606 GN=TONSL PE=1 SV=2	TONSL	0.801	
Q96HC4	PDZ and LIM domain protein 5 OS=Homo sapiens OX=9606 GN=PDLIM5 PE=1 SV=5	PDLIM5	1.159	0.048624
Q96HE7	ERO1-like protein alpha OS=Homo sapiens OX=9606 GN=ERO1A PE=1 SV=2	ERO1A	1.045	0.381538
Q96HP0	Dedicator of cytokinesis protein 6 OS=Homo sapiens OX=9606 GN=DOCK6 PE=1 SV=3	DOCK6	0.956	0.714386
Q96HQ2	CDKN2AIP N-terminal-like protein OS=Homo sapiens OX=9606 GN=CDKN2AIPNL	CDKN2AIPNL	0.855	0.072911
Q96HS1	Serine/threonine-protein phosphatase PGAM5, mitochondrial OS=Homo sapiens OX=9606 GN=PGAM5 PE=1	PGAM5	1.036	0.525532
Q96HV5	Transmembrane protein 41A OS=Homo sapiens OX=9606 GN=TMEM41A PE=1 SV=1	TMEM41A	0.651	0.013512
Q96HW7	Integrator complex subunit 4 OS=Homo sapiens OX=9606 GN=INTS4 PE=1 SV=2	INTS4	1.167	0.153003
Q96HY6	DDRGK domain-containing protein 1 OS=Homo sapiens OX=9606 GN=DDRGK1 PE=1	DDRGK1	0.907	0.014762
Q96HY7	Probable 2-oxoglutarate dehydrogenase E1 component DHKTD1, mitochondrial OS=Homo sapiens OX=9606 GN=DHTKD1 PE=1 SV=2	DHTKD1	0.975	0.830642
Q96I24	Far upstream element-binding protein 3 OS=Homo sapiens OX=9606 GN=FUBP3 PE=1	FUBP3	0.918	0.043673
Q96I25	Splicing factor 45 OS=Homo sapiens OX=9606 GN=RBM17	RBM17	0.92	0.0628
Q96I51	RCC1-like G exchanging factor-like protein OS=Homo sapiens OX=9606 GN=RCC1L PE=1	RCC1L	0.972	0.756943
Q96I99	Succinate--CoA ligase [GDP-forming] subunit beta, mitochondrial OS=Homo sapiens OX=9606 GN=SUCLG2 PE=1	SUCLG2	1.073	0.216031
Q96IX5	ATP synthase membrane subunit K, mitochondrial OS=Homo sapiens OX=9606 GN=ATP5MK PE=1 SV=1	ATP5MK	1.013	0.934844

Q96IZ0	PRKC apoptosis WT1 regulator protein OS=Homo sapiens OX=9606 GN=PAWR PE=1	PAWR	1.017	0.872451
Q96J01	THO complex subunit 3 OS=Homo sapiens OX=9606 GN=THOC3 PE=1 SV=1	THOC3	1.02	0.799397
Q96J02	E3 ubiquitin-protein ligase Itchy homolog OS=Homo sapiens OX=9606 GN=ITCH PE=1 SV=2	ITCH	1.071	
Q96J42	Thioredoxin domain-containing protein 15 OS=Homo sapiens OX=9606 GN=TXNDC15 PE=1 SV=1	TXNDC15	1.091	0.881579
Q96JB2	Conserved oligomeric Golgi complex subunit 3 OS=Homo sapiens OX=9606 GN=COG3 PE=1 SV=3	COG3	0.85	0.159786
Q96JB5	CDK5 regulatory subunit-associated protein 3 OS=Homo sapiens OX=9606 GN=CDK5RAP3 PE=1 SV=2	CDK5RAP3	1.064	0.315444
Q96JJ7	Protein disulfide-isomerase TMX3 OS=Homo sapiens OX=9606 GN=TMX3 PE=1 SV=2	TMX3	1.032	0.619075
Q96JM3	Chromosome alignment-maintaining phosphoprotein 1 OS=Homo sapiens OX=9606 GN=CHAMP1 PE=1 SV=2	CHAMP1	1.059	0.49911
Q96JP5	E3 ubiquitin-protein ligase ZFP91 OS=Homo sapiens OX=9606 GN=ZFP91 PE=1 SV=1	ZFP91	0.829	0.088602
Q96K17	Transcription factor BTF3 homolog 4 OS=Homo sapiens OX=9606 GN=BTF3L4 PE=1	BTF3L4	0.888	0.217504
Q96K37	Solute carrier family 35 member E1 OS=Homo sapiens OX=9606 GN=SLC35E1 PE=1 SV=2	SLC35E1	0.979	0.817316
Q96K49	Transmembrane protein 87B OS=Homo sapiens OX=9606 GN=TMEM87B PE=1 SV=1	TMEM87B	1.051	0.606183
Q96KA5	Cleft lip and palate transmembrane protein 1-like protein OS=Homo sapiens OX=9606 GN=CLPTM1L PE=1 SV=1	CLPTM1L	0.882	0.114504
Q96KC8	DnaJ homolog subfamily C member 1 OS=Homo sapiens OX=9606 GN=DNAJC1 PE=1	DNAJC1	0.957	0.398074
Q96KP1	Exocyst complex component 2 OS=Homo sapiens OX=9606 GN=EXOC2 PE=1 SV=1	EXOC2	1.149	0.255562
Q96KP4	Cytosolic non-specific dipeptidase OS=Homo sapiens OX=9606 GN=CNDP2 PE=1 SV=2	CNDP2	0.989	0.847076

Q96KQ7	Histone-lysine N-methyltransferase EHMT2 OS=Homo sapiens OX=9606 GN=EHMT2 PE=1	EHMT2	0.95	0.316945
Q96KR1	Zinc finger RNA-binding protein OS=Homo sapiens OX=9606 GN=ZFR PE=1 SV=2	ZFR	0.859	0.107606
Q96L91	E1A-binding protein p400 OS=Homo sapiens OX=9606 GN=EP400 PE=1 SV=4	EP400	0.965	0.775862
Q96L92	Sorting nexin-27 OS=Homo sapiens OX=9606 GN=SNX27	SNX27	0.821	
Q96M27	Protein PRRC1 OS=Homo sapiens OX=9606 GN=PRRC1 PE=1	PRRC1	1.094	0.53504
Q96MU7	YTH domain-containing protein 1 OS=Homo sapiens OX=9606 GN=YTHDC1 PE=1 SV=3	YTHDC1	1.036	0.689789
Q96MW5	Conserved oligomeric Golgi complex subunit 8 OS=Homo sapiens OX=9606 GN=COG8 PE=1 SV=2	COG8	0.875	0.569222
Q96N66	Lysophospholipid acyltransferase 7 OS=Homo sapiens OX=9606 GN=MBOAT7 PE=1 SV=2	MBOAT7	1.077	0.495624
Q96N67	Dedicator of cytokinesis protein 7 OS=Homo sapiens OX=9606 GN=DOCK7 PE=1 SV=4	DOCK7	1.205	0.146063
Q96ND0	Protein FAM210A OS=Homo sapiens OX=9606 GN=FAM210A PE=1 SV=2	FAM210A	0.963	0.699915
Q96P16	Regulation of nuclear pre-mRNA domain-containing protein 1A OS=Homo sapiens OX=9606 GN=RPRD1A PE=1 SV=1	RPRD1A	0.987	0.881025
Q96P70	Importin-9 OS=Homo sapiens OX=9606 GN=IPO9 PE=1 SV=3	IPO9	1.202	
Q96PC5	Melanoma inhibitory activity protein 2 OS=Homo sapiens OX=9606 GN=MIA2 PE=1 SV=4	MIA2	0.98	0.718376
Q96PD2	Discoidin, CUB and LCCL domain-containing protein 2 OS=Homo sapiens OX=9606 GN=DCBLD2 PE=1 SV=1	DCBLD2	1.086	0.049199
Q96PK6	RNA-binding protein 14 OS=Homo sapiens OX=9606 GN=RBM14 PE=1 SV=2	RBM14	0.994	0.886355
Q96PU8	Protein quaking OS=Homo sapiens OX=9606 GN=QKI PE=1 SV=1	QKI	0.819	0.018534
Q96PV4	Paraneoplastic antigen-like protein 5 OS=Homo sapiens OX=9606 GN=PNMA5 PE=1 SV=2	PNMA5	0.955	0.154903
Q96PY5	Formin-like protein 2 OS=Homo sapiens OX=9606 GN=FMNL2 PE=1 SV=3	FMNL2	0.995	0.917686

Q96PZ0	Pseudouridylate synthase 7 homolog OS=Homo sapiens OX=9606 GN=PUS7 PE=1 SV=2	PUS7	0.926	0.527456
Q96Q11	CCA tRNA nucleotidyltransferase 1, mitochondrial OS=Homo sapiens OX=9606 GN=TRNT1	TRNT1	0.871	0.101837
Q96Q15	Serine/threonine-protein kinase SMG1 OS=Homo sapiens OX=9606 GN=SMG1 PE=1 SV=3	SMG1	1000	0.001
Q96QC0	Serine/threonine-protein phosphatase 1 regulatory subunit 10 OS=Homo sapiens OX=9606 GN=PPP1R10 PE=1 SV=1	PPP1R10	0.918	0.571118
Q96QD8	Sodium-coupled neutral amino acid transporter 2 OS=Homo sapiens OX=9606 GN=SLC38A2 PE=1 SV=2	SLC38A2	1.035	0.582511
Q96QD9	UAP56-interacting factor OS=Homo sapiens OX=9606 GN=FYTTD1 PE=1 SV=3	FYTTD1	1.52	0.562665
Q96QK1	Vacuolar protein sorting-associated protein 35 OS=Homo sapiens OX=9606 GN=VPS35 PE=1 SV=2	VPS35	0.996	0.997654
Q96QR8	Transcriptional activator protein Pur-beta OS=Homo sapiens OX=9606 GN=PURB PE=1 SV=3	PURB	1.005	0.879255
Q96QT6	PHD finger protein 12 OS=Homo sapiens OX=9606 GN=PHF12 PE=1 SV=2	PHF12	0.92	0.56228
Q96RD7	Pannexin-1 OS=Homo sapiens OX=9606 GN=PANX1 PE=1	PANX1	1.141	0.398282
Q96RL1	BRCA1-A complex subunit RAP80 OS=Homo sapiens OX=9606 GN=UIMC1 PE=1	UIMC1	0.847	0.122568
Q96RN5	Mediator of RNA polymerase II transcription subunit 15 OS=Homo sapiens OX=9606 GN=MED15 PE=1 SV=2	MED15	0.973	0.70405
Q96RP9	Elongation factor G, mitochondrial OS=Homo sapiens OX=9606 GN=GFM1 PE=1 SV=2	GFM1	0.967	0.559737
Q96RQ1	Endoplasmic reticulum-Golgi intermediate compartment protein 2 OS=Homo sapiens OX=9606 GN=ERGIC2 PE=1 SV=2	ERGIC2	1.014	0.830746
Q96RQ3	Methylcrotonoyl-CoA carboxylase subunit alpha, mitochondrial OS=Homo sapiens OX=9606 GN=MCCC1 PE=1 SV=3	MCCC1	0.92	0.073686
Q96RS6	NudC domain-containing protein 1 OS=Homo sapiens OX=9606 GN=NUDCD1 PE=1 SV=2	NUDCD1	1.015	0.747926

Q96RT1	Erbin OS=Homo sapiens OX=9606 GN=ERBIN PE=1	ERBIN	0.837	0.021728
Q96S52	GPI transamidase component PIG-S OS=Homo sapiens OX=9606 GN=PIGS PE=1 SV=3	PIGS	1.002	0.985498
Q96S55	ATPase WRNIP1 OS=Homo sapiens OX=9606 GN=WRNIP1 PE=1 SV=2	WRNIP1	1.002	
Q96S66	Chloride channel CLIC-like protein 1 OS=Homo sapiens OX=9606 GN=CLCC1 PE=1	CLCC1	1.011	0.936787
Q96S97	Myeloid-associated differentiation marker OS=Homo sapiens OX=9606 GN=MYADM PE=1	MYADM	0.958	0.634554
Q96SB4	SRSF protein kinase 1 OS=Homo sapiens OX=9606 GN=SRPK1 PE=1 SV=2	SRPK1	1.067	0.358081
Q96SB8	Structural maintenance of chromosomes protein 6 OS=Homo sapiens OX=9606 GN=SMC6 PE=1 SV=2	SMC6	0.83	0.111893
Q96SI9	Spermatid perinuclear RNA-binding protein OS=Homo sapiens OX=9606 GN=STRBP PE=1	STRBP	0.95	0.066726
Q96SK2	Transmembrane protein 209 OS=Homo sapiens OX=9606 GN=TMEM209 PE=1 SV=2	TMEM209	1.172	0.032632
Q96SQ9	Cytochrome P450 2S1 OS=Homo sapiens OX=9606 GN=CYP2S1 PE=1 SV=2	CYP2S1	1.026	0.725515
Q96ST2	Protein IWS1 homolog OS=Homo sapiens OX=9606 GN=IWS1 PE=1 SV=2	IWS1	0.956	0.618456
Q96ST3	Paired amphipathic helix protein Sin3a OS=Homo sapiens OX=9606 GN=SIN3A PE=1 SV=2	SIN3A	0.96	0.672465
Q96SU4	Oxysterol-binding protein-related protein 9 OS=Homo sapiens OX=9606 GN=OSBPL9 PE=1 SV=2	OSBPL9	1.337	0.015491
Q96SY0	Integrator complex subunit 14 OS=Homo sapiens OX=9606 GN=INTS14 PE=1 SV=2	INTS14	0.907	0.183404
Q96T23	Remodeling and spacing factor 1 OS=Homo sapiens OX=9606 GN=RSF1 PE=1 SV=2	RSF1	0.954	0.306441
Q96T37	RNA-binding protein 15 OS=Homo sapiens OX=9606 GN=RBM15 PE=1 SV=2	RBM15	0.937	0.37078
Q96T76	MMS19 nucleotide excision repair protein homolog OS=Homo sapiens OX=9606 GN=MMS19	MMS19	1.25	

Q96T88	E3 ubiquitin-protein ligase UHRF1 OS=Homo sapiens OX=9606 GN=UHRF1 PE=1 SV=1	UHRF1	1.014	0.849765
Q96TA1	Protein Niban 2 OS=Homo sapiens OX=9606 GN=NIBAN2 PE=1 SV=3	NIBAN2	1.111	0.547158
Q96TA2	ATP-dependent zinc metalloprotease YME1L1 OS=Homo sapiens OX=9606 GN=YME1L1 PE=1 SV=2	YME1L1	0.977	0.707213
Q96TC7	Regulator of microtubule dynamics protein 3 OS=Homo sapiens OX=9606 GN=RMDN3 PE=1	RMDN3	0.97	0.601535
Q99417	c-Myc-binding protein OS=Homo sapiens OX=9606 GN=MYCBP PE=1 SV=3	MYCBP	1.008	0.939111
Q99426	Tubulin-folding cofactor B OS=Homo sapiens OX=9606 GN=TBCB PE=1 SV=2	TBCB	0.844	0.231419
Q99436	Proteasome subunit beta type-7 OS=Homo sapiens OX=9606 GN=PSMB7 PE=1 SV=1	PSMB7	1.023	0.842988
Q99439	Calponin-2 OS=Homo sapiens OX=9606 GN=CNN2 PE=1 SV=4	CNN2	0.938	0.498364
Q99442	Translocation protein SEC62 OS=Homo sapiens OX=9606 GN=SEC62 PE=1 SV=1	SEC62	1.027	0.7171
Q99459	Cell division cycle 5-like protein OS=Homo sapiens OX=9606 GN=CDC5L PE=1 SV=2	CDC5L	0.973	0.627821
Q99460	26S proteasome non-ATPase regulatory subunit 1 OS=Homo sapiens OX=9606 GN=PSMD1 PE=1 SV=2	PSMD1	0.965	0.554157
Q99470	Stromal cell-derived factor 2 OS=Homo sapiens OX=9606 GN=SDF2 PE=1 SV=2	SDF2	1.145	0.130965
Q99471	Prefoldin subunit 5 OS=Homo sapiens OX=9606 GN=PFDN5 PE=1 SV=2	PFDN5	0.785	0.114809
Q99496	E3 ubiquitin-protein ligase RING2 OS=Homo sapiens OX=9606 GN=RNF2 PE=1 SV=1	RNF2	0.875	0.255739
Q99497	Parkinson disease protein 7 OS=Homo sapiens OX=9606 GN=PARK7 PE=1 SV=2	PARK7	0.901	0.382316
Q99519	Sialidase-1 OS=Homo sapiens OX=9606 GN=NEU1 PE=1 SV=1	NEU1	1.053	0.531706
Q99536	Synaptic vesicle membrane protein VAT-1 homolog OS=Homo sapiens OX=9606 GN=VAT1	VAT1	1.04	0.660812
Q99538	Legumain OS=Homo sapiens OX=9606 GN=LGMN PE=1	LGMN	0.968	0.402016

Q99547	M-phase phosphoprotein 6 OS=Homo sapiens OX=9606 GN=MPHOSPH6 PE=1 SV=2	MPHOSPH6	0.867	0.185331
Q99549	M-phase phosphoprotein 8 OS=Homo sapiens OX=9606 GN=MPHOSPH8 PE=1 SV=2	MPHOSPH8	1.006	0.978773
Q99567	Nuclear pore complex protein Nup88 OS=Homo sapiens OX=9606 GN=NUP88 PE=1	NUP88	1.032	0.62495
Q99575	Ribonucleases P/MRP protein subunit POP1 OS=Homo sapiens OX=9606 GN=POP1 PE=1 SV=2	POP1	0.823	0.23714
Q99584	Protein S100-A13 OS=Homo sapiens OX=9606 GN=S100A13 PE=1 SV=1	S100A13	0.746	0.116797
Q99590	Protein SCAF11 OS=Homo sapiens OX=9606 GN=SCAF11	SCAF11	1.036	0.277194
Q99613	Eukaryotic translation initiation factor 3 subunit C OS=Homo sapiens OX=9606 GN=EIF3C PE=1 SV=1	EIF3C	0.997	0.937007
Q99614	Tetratricopeptide repeat protein 1 OS=Homo sapiens OX=9606 GN=TTC1 PE=1 SV=1	TTC1	1.105	0.704626
Q99615	DnaJ homolog subfamily C member 7 OS=Homo sapiens OX=9606 GN=DNAJC7 PE=1	DNAJC7	0.914	0.273747
Q99623	Prohibitin-2 OS=Homo sapiens OX=9606 GN=PHB2 PE=1 SV=2	PHB2	1.078	0.072186
Q99627	COP9 signalosome complex subunit 8 OS=Homo sapiens OX=9606 GN=COPS8 PE=1	COPS8	1.006	0.947717
Q99640	Membrane-associated tyrosine- and threonine-specific cdc2-inhibitory kinase OS=Homo sapiens OX=9606 GN=PKMYT1	PKMYT1	1.044	0.611129
Q99643	Succinate dehydrogenase cytochrome b560 subunit, mitochondrial OS=Homo sapiens OX=9606 GN=SDHC PE=1 SV=1	SDHC	1.094	0.360358
Q99653	Calcineurin B homologous protein 1 OS=Homo sapiens OX=9606 GN=CHP1 PE=1 SV=3	CHP1	1.133	0.127096
Q99661	Kinesin-like protein KIF2C OS=Homo sapiens OX=9606 GN=KIF2C PE=1 SV=2	KIF2C	0.969	0.674349
Q99714	3-hydroxyacyl-CoA dehydrogenase type-2 OS=Homo sapiens OX=9606 GN=HSD17B10 PE=1	HSD17B10	0.972	0.602512
Q99715	Collagen alpha-1(XII) chain OS=Homo sapiens OX=9606 GN=COL12A1 PE=1 SV=2	COL12A1	0.83	0.13208

Q99720	Sigma non-opioid intracellular receptor 1 OS=Homo sapiens OX=9606 GN=SIGMAR1 PE=1 SV=1	SIGMAR1	0.973	0.680317
Q99729	Heterogeneous nuclear ribonucleoprotein A/B OS=Homo sapiens OX=9606 GN=HNRNPAB PE=1 SV=2	HNRNPAB	0.942	0.412113
Q99733	Nucleosome assembly protein 1-like 4 OS=Homo sapiens OX=9606 GN=NAP1L4 PE=1	NAP1L4	1.103	0.344536
Q99747	Gamma-soluble NSF attachment protein OS=Homo sapiens OX=9606 GN=NAPG PE=1 SV=1	NAPG	1.197	0.111451
Q99757	Thioredoxin, mitochondrial OS=Homo sapiens OX=9606 GN=TXN2 PE=1 SV=2	TXN2	1.006	0.889306
Q99797	Mitochondrial intermediate peptidase OS=Homo sapiens OX=9606 GN=MIPEP PE=1	MIPEP	0.895	0.409193
Q99798	Aconitate hydratase, mitochondrial OS=Homo sapiens OX=9606 GN=ACO2 PE=1 SV=2	ACO2	0.981	0.621664
Q99805	Transmembrane 9 superfamily member 2 OS=Homo sapiens OX=9606 GN=TM9SF2 PE=1	TM9SF2	1.093	0.034858
Q99808	Equilibrative nucleoside transporter 1 OS=Homo sapiens OX=9606 GN=SLC29A1 PE=1	SLC29A1	0.98	0.802293
Q99816	Tumor susceptibility gene 101 protein OS=Homo sapiens OX=9606 GN=TSG101 PE=1	TSG101	1.194	0.119842
Q99828	Calcium and integrin-binding protein 1 OS=Homo sapiens OX=9606 GN=CIB1 PE=1 SV=4	CIB1	0.944	0.368663
Q99829	Copine-1 OS=Homo sapiens OX=9606 GN=CPNE1 PE=1	CPNE1	1.134	0.500015
Q99832	T-complex protein 1 subunit eta OS=Homo sapiens OX=9606 GN=CCT7 PE=1 SV=2	CCT7	0.955	0.590083
Q99848	Probable rRNA-processing protein EBP2 OS=Homo sapiens OX=9606 GN=EBNA1BP2 PE=1	EBNA1BP2	0.918	0.075721
Q99873	Protein arginine N-methyltransferase 1 OS=Homo sapiens OX=9606 GN=PRMT1	PRMT1	0.92	0.27596
Q99942	E3 ubiquitin-protein ligase RNF5 OS=Homo sapiens OX=9606 GN=RNF5 PE=1 SV=1	RNF5	0.001	0.001
Q99943	1-acyl-sn-glycerol-3-phosphate acyltransferase alpha OS=Homo sapiens OX=9606 GN=AGPAT1 PE=1 SV=2	AGPAT1	1.048	0.808857

Q99959	Plakophilin-2 OS=Homo sapiens OX=9606 GN=PKP2 PE=1 SV=2	PKP2	0.984	0.791157
Q99961	Endophilin-A2 OS=Homo sapiens OX=9606 GN=SH3GL1 PE=1 SV=1	SH3GL1	1.187	0.084073
Q99985	Semaphorin-3C OS=Homo sapiens OX=9606 GN=SEMA3C PE=2 SV=2	SEMA3C	1.046	0.548042
Q99986	Serine/threonine-protein kinase VRK1 OS=Homo sapiens OX=9606 GN=VRK1 PE=1 SV=1	VRK1	0.943	0.596799
Q99996	A-kinase anchor protein 9 OS=Homo sapiens OX=9606 GN=AKAP9 PE=1 SV=4	AKAP9	1.214	0.091786
Q9BPW8	Protein NipSnap homolog 1 OS=Homo sapiens OX=9606 GN=NIPSNAP1 PE=1 SV=1	NIPSNAP1	1.202	0.170686
Q9BPX3	Condensin complex subunit 3 OS=Homo sapiens OX=9606 GN=NCAPG PE=1 SV=1	NCAPG	0.787	0.25386
Q9BPX5	Actin-related protein 2/3 complex subunit 5-like protein OS=Homo sapiens OX=9606 GN=ARPC5L PE=1 SV=1	ARPC5L	0.952	0.326283
Q9BQ39	ATP-dependent RNA helicase DDX50 OS=Homo sapiens OX=9606 GN=DDX50 PE=1	DDX50	0.834	0.168691
Q9BQ52	Zinc phosphodiesterase ELAC protein 2 OS=Homo sapiens OX=9606 GN=ELAC2 PE=1	ELAC2	0.872	0.122985
Q9BQ61	Telomerase RNA component interacting RNase OS=Homo sapiens OX=9606 GN=TRIR PE=1 SV=1	TRIR	1.006	0.941933
Q9BQ67	Glutamate-rich WD repeat-containing protein 1 OS=Homo sapiens OX=9606 GN=GRWD1 PE=1 SV=1	GRWD1	1.005	0.979595
Q9BQ75	Protein CMSS1 OS=Homo sapiens OX=9606 GN=CMSS1 PE=1	CMSS1	0.913	0.356207
Q9BQ95	Evolutionarily conserved signaling intermediate in Toll pathway, mitochondrial OS=Homo sapiens OX=9606 GN=ECSIT PE=1 SV=1	ECSIT	0.973	0.725691
Q9BQA1	Methylosome protein 50 OS=Homo sapiens OX=9606 GN=WDR77 PE=1 SV=1	WDR77	1.136	0.135897
Q9BQB6	Vitamin K epoxide reductase complex subunit 1 OS=Homo sapiens OX=9606 GN=VKORC1 PE=1 SV=1	VKORC1	1.001	0.995553

Q9BQC6	Ribosomal protein 63, mitochondrial OS=Homo sapiens OX=9606 GN=MRPL57 PE=1	MRPL57	1.067	0.616064
Q9BQE3	Tubulin alpha-1C chain OS=Homo sapiens OX=9606 GN=TUBA1C PE=1 SV=1	TUBA1C	1.098	0.452647
Q9BQE5	Apolipoprotein L2 OS=Homo sapiens OX=9606 GN=APOL2 PE=1 SV=1	APOL2	0.847	0.379151
Q9BQE9	B-cell CLL/lymphoma 7 protein family member B OS=Homo sapiens OX=9606 GN=BCL7B	BCL7B	0.916	0.285654
Q9BQG0	Myb-binding protein 1A OS=Homo sapiens OX=9606 GN=MYBBP1A PE=1 SV=2	MYBBP1A	1.012	0.983127
Q9BQL6	Fermitin family homolog 1 OS=Homo sapiens OX=9606 GN=FERMT1 PE=1 SV=1	FERMT1	1.013	
Q9BQP7	Mitochondrial genome maintenance exonuclease 1 OS=Homo sapiens OX=9606 GN=MGME1 PE=1 SV=1	MGME1	0.925	0.515377
Q9BR76	Coronin-1B OS=Homo sapiens OX=9606 GN=CORO1B PE=1 SV=1	CORO1B	0.93	0.344542
Q9BRA2	Thioredoxin domain-containing protein 17 OS=Homo sapiens OX=9606 GN=TXNDC17 PE=1 SV=1	TXNDC17	0.974	0.772529
Q9BRG1	Vacuolar protein-sorting-associated protein 25 OS=Homo sapiens OX=9606 GN=VPS25	VPS25	1.183	0.243673
Q9BRJ2	39S ribosomal protein L45, mitochondrial OS=Homo sapiens OX=9606 GN=MRPL45 PE=1 SV=2	MRPL45	0.926	0.212234
Q9BRJ6	Uncharacterized protein C7orf50 OS=Homo sapiens OX=9606 GN=C7orf50 PE=1 SV=1	C7orf50	0.775	0.0195
Q9BRK5	45 kDa calcium-binding protein OS=Homo sapiens OX=9606 GN=SDF4 PE=1 SV=1	SDF4	0.932	0.437157
Q9BRP8	Partner of Y14 and mago OS=Homo sapiens OX=9606 GN=PYM1 PE=1 SV=1	PYM1	1.027	0.726086
Q9BRR6	ADP-dependent glucokinase OS=Homo sapiens OX=9606 GN=ADPGK PE=1 SV=1	ADPGK	1.017	0.774358
Q9BRT3	Migration and invasion enhancer 1 OS=Homo sapiens OX=9606 GN=MIEN1 PE=1 SV=1	MIEN1	1.038	0.844

Q9BRT6	Protein LLP homolog OS=Homo sapiens OX=9606 GN=LLPH PE=1 SV=1	LLPH	0.901	0.138326
Q9BRT9	DNA replication complex GINS protein SLD5 OS=Homo sapiens OX=9606 GN=GINS4 PE=1 SV=1	GINS4	1.024	0.850734
Q9BRU9	rRNA-processing protein UTP23 homolog OS=Homo sapiens OX=9606 GN=UTP23 PE=1	UTP23	0.955	0.680836
Q9BRX5	DNA replication complex GINS protein PSF3 OS=Homo sapiens OX=9606 GN=GINS3 PE=1 SV=1	GINS3	1.445	
Q9BRX8	Peroxiredoxin-like 2A OS=Homo sapiens OX=9606 GN=PRXL2A PE=1 SV=3	PRXL2A	0.791	0.485138
Q9BS26	Endoplasmic reticulum resident protein 44 OS=Homo sapiens OX=9606 GN=ERP44 PE=1 SV=1	ERP44	0.996	0.968967
Q9BSC4	Nucleolar protein 10 OS=Homo sapiens OX=9606 GN=NOL10 PE=1 SV=1	NOL10	1.005	0.962736
Q9BSF4	Mitochondrial import inner membrane translocase subunit Tim29 OS=Homo sapiens OX=9606 GN=TIMM29 PE=1	TIMM29	1.098	
Q9BSH4	Translational activator of cytochrome c oxidase 1 OS=Homo sapiens OX=9606 GN=TACO1 PE=1 SV=1	TACO1	0.943	0.372122
Q9BSJ8	Extended synaptotagmin-1 OS=Homo sapiens OX=9606 GN=ESYT1 PE=1 SV=1	ESYT1	1.064	0.272188
Q9BSR8	Protein YIPF4 OS=Homo sapiens OX=9606 GN=YIPF4 PE=1 SV=1	YIPF4	0.971	0.332685
Q9BT09	Protein canopy homolog 3 OS=Homo sapiens OX=9606 GN=CNPY3 PE=1 SV=1	CNPY3	0.994	0.919195
Q9BT22	Chitobiosyldiphosphodolichol beta-mannosyltransferase OS=Homo sapiens OX=9606 GN=ALG1 PE=1 SV=2	ALG1	1.033	0.665672
Q9BT67	NEDD4 family-interacting protein 1 OS=Homo sapiens OX=9606 GN=NDFIP1 PE=1 SV=1	NDFIP1	1.126	0.232284
Q9BT78	COP9 signalosome complex subunit 4 OS=Homo sapiens OX=9606 GN=COPS4 PE=1	COPS4	1.027	0.779985
Q9BTA9	WW domain-containing adapter protein with coiled-coil OS=Homo sapiens OX=9606 GN=WAC PE=1 SV=3	WAC	0.895	0.325087

Q9BTC0	Death-inducer obliterator 1 OS=Homo sapiens OX=9606 GN=DIDO1 PE=1 SV=5	DIDO1	0.953	0.270773
Q9BTD8	RNA-binding protein 42 OS=Homo sapiens OX=9606 GN=RBM42 PE=1 SV=1	RBM42	0.915	0.077597
Q9BTE3	Mini-chromosome maintenance complex-binding protein OS=Homo sapiens OX=9606 GN=MCMBP PE=1 SV=2	MCMBP	0.779	0.234289
Q9BTE7	DCN1-like protein 5 OS=Homo sapiens OX=9606 GN=DCUN1D5 PE=1 SV=1	DCUN1D5	0.862	0.384128
Q9BTL3	RNA guanine-N7 methyltransferase activating subunit OS=Homo sapiens OX=9606 GN=RAMAC PE=1	RAMAC	0.848	0.01026
Q9BTT0	Acidic leucine-rich nuclear phosphoprotein 32 family member E OS=Homo sapiens OX=9606 GN=ANP32E PE=1 SV=1	ANP32E	0.891	0.344057
Q9BTT6	Leucine-rich repeat-containing protein 1 OS=Homo sapiens OX=9606 GN=LRRC1 PE=1	LRRC1	0.876	0.484959
Q9BTU6	Phosphatidylinositol 4-kinase type 2-alpha OS=Homo sapiens OX=9606 GN=PI4K2A PE=1	PI4K2A	1.115	0.194511
Q9BTV4	Transmembrane protein 43 OS=Homo sapiens OX=9606 GN=TMEM43 PE=1 SV=1	TMEM43	1.034	0.61937
Q9BTX1	Nucleoporin NDC1 OS=Homo sapiens OX=9606 GN=NDC1 PE=1 SV=2	NDC1	0.975	0.791669
Q9BU61	NADH dehydrogenase [ubiquinone] 1 alpha subcomplex assembly factor 3 OS=Homo sapiens OX=9606 GN=NDUFAF3	NDUFAF3	0.861	0.015686
Q9BU76	Multiple myeloma tumor-associated protein 2 OS=Homo sapiens OX=9606 GN=MMTAG2 PE=1 SV=1	MMTAG2	0.878	0.083747
Q9BUA3	Spindlin interactor and repressor of chromatin-binding protein OS=Homo sapiens OX=9606 GN=SPINDOC PE=1 SV=3	SPINDOC	0.86	0.513402
Q9BUB7	Transmembrane protein 70, mitochondrial OS=Homo sapiens OX=9606 GN=TMEM70 PE=1 SV=2	TMEM70	1.012	0.901422
Q9BUF5	Tubulin beta-6 chain OS=Homo sapiens OX=9606 GN=TUBB6 PE=1 SV=1	TUBB6	1.186	0.322182

Q9BUJ2	Heterogeneous nuclear ribonucleoprotein U-like protein 1 OS=Homo sapiens OX=9606 GN=HNRNPUL1 PE=1 SV=2	HNRNPUL1	0.999	0.966276
Q9BUL8	Programmed cell death protein 10 OS=Homo sapiens OX=9606 GN=PDCD10 PE=1 SV=1	PDCD10	1.005	0.95491
Q9BUN8	Derlin-1 OS=Homo sapiens OX=9606 GN=DERL1 PE=1	DERL1	1.046	0.817865
Q9BUP3	Oxidoreductase HTATIP2 OS=Homo sapiens OX=9606 GN=HTATIP2 PE=1 SV=2	HTATIP2	1.054	0.039468
Q9BUQ8	Probable ATP-dependent RNA helicase DDX23 OS=Homo sapiens OX=9606 GN=DDX23	DDX23	0.952	0.065941
Q9BUR5	MICOS complex subunit MIC26 OS=Homo sapiens OX=9606 GN=APOO PE=1 SV=1	APOO	1.101	0.577053
Q9BUZ4	TNF receptor-associated factor 4 OS=Homo sapiens OX=9606 GN=TRAF4 PE=1 SV=1	TRAF4	1.074	0.276476
Q9BV38	WD repeat-containing protein 18 OS=Homo sapiens OX=9606 GN=WDR18 PE=1 SV=2	WDR18	1.002	0.989198
Q9BV40	Vesicle-associated membrane protein 8 OS=Homo sapiens OX=9606 GN=VAMP8 PE=1	VAMP8	0.994	0.914924
Q9BVA1	Tubulin beta-2B chain OS=Homo sapiens OX=9606 GN=TUBB2B PE=1 SV=1	TUBB2B	1.002	0.934608
Q9BVC6	Transmembrane protein 109 OS=Homo sapiens OX=9606 GN=TMEM109 PE=1 SV=1	TMEM109	0.935	
Q9BVI4	Nucleolar complex protein 4 homolog OS=Homo sapiens OX=9606 GN=NOC4L PE=1	NOC4L	0.98	0.746073
Q9BVJ6	U3 small nucleolar RNA-associated protein 14 homolog A OS=Homo sapiens OX=9606 GN=UTP14A PE=1 SV=1	UTP14A	1.022	0.778593
Q9BVK6	Transmembrane emp24 domain-containing protein 9 OS=Homo sapiens OX=9606 GN=TMED9 PE=1 SV=2	TMED9	1.022	0.737962
Q9BVL2	Nucleoporin p58/p45 OS=Homo sapiens OX=9606 GN=NUP58 PE=1 SV=1	NUP58	1.049	0.438126
Q9BVP2	Guanine nucleotide-binding protein-like 3 OS=Homo sapiens OX=9606 GN=GNL3 PE=1 SV=2	GNL3	1.068	0.340924

Q9BVT8	Transmembrane and ubiquitin-like domain-containing protein 1 OS=Homo sapiens OX=9606 GN=TMUB1 PE=1 SV=1	TMUB1	0.892	0.537477
Q9BVV7	Mitochondrial import inner membrane translocase subunit Tim21 OS=Homo sapiens OX=9606 GN=TIMM21 PE=1	TIMM21	0.811	0.039093
Q9BVX2	Transmembrane protein 106C OS=Homo sapiens OX=9606 GN=TMEM106C PE=1 SV=1	TMEM106C	1.129	
Q9BW19	Kinesin-like protein KIFC1 OS=Homo sapiens OX=9606 GN=KIFC1 PE=1 SV=2	KIFC1	0.756	0.21194
Q9BW27	Nuclear pore complex protein Nup85 OS=Homo sapiens OX=9606 GN=NUP85 PE=1	NUP85	1.046	0.290524
Q9BW60	Elongation of very long chain fatty acids protein 1 OS=Homo sapiens OX=9606 GN=ELOVL1 PE=1 SV=1	ELOVL1	1.064	0.724525
Q9BW72	HIG1 domain family member 2A, mitochondrial OS=Homo sapiens OX=9606 GN=HIGD2A PE=1 SV=1	HIGD2A	1.089	0.671825
Q9BW92	Threonine--tRNA ligase, mitochondrial OS=Homo sapiens OX=9606 GN=TARS2 PE=1	TARS2	0.912	0.318532
Q9BWD1	Acetyl-CoA acetyltransferase, cytosolic OS=Homo sapiens OX=9606 GN=ACAT2 PE=1	ACAT2	1.015	0.940895
Q9BWF3	RNA-binding protein 4 OS=Homo sapiens OX=9606 GN=RBM4 PE=1 SV=1	RBM4	0.833	0.037983
Q9BWJ5	Splicing factor 3B subunit 5 OS=Homo sapiens OX=9606 GN=SF3B5 PE=1 SV=1	SF3B5	1.088	0.405197
Q9BWM7	Sideroflexin-3 OS=Homo sapiens OX=9606 GN=SFXN3 PE=1	SFXN3	1.103	0.312751
Q9BWS9	Chitinase domain-containing protein 1 OS=Homo sapiens OX=9606 GN=CHID1 PE=1	CHID1	1.148	0.170275
Q9BWU0	Kanadaptin OS=Homo sapiens OX=9606 GN=SLC4A1AP PE=1 SV=1	SLC4A1AP	0.863	0.350304
Q9BX40	Protein LSM14 homolog B OS=Homo sapiens OX=9606 GN=LSM14B PE=1 SV=1	LSM14B	1.024	0.839388
Q9BX59	Tapasin-related protein OS=Homo sapiens OX=9606 GN=TAPBPL PE=1 SV=2	TAPBPL	1.064	0.410415

Q9BX68	Adenosine 5'-monophosphoramidase HINT2 OS=Homo sapiens OX=9606	HINT2	0.866	0.010021
Q9BXJ9	N-alpha-acetyltransferase 15, NatA auxiliary subunit OS=Homo sapiens OX=9606 GN=NAA15	NAA15	1.07	0.57244
Q9BXK5	Bcl-2-like protein 13 OS=Homo sapiens OX=9606 GN=BCL2L13 PE=1 SV=1	BCL2L13	1.084	0.409141
Q9BXP2	Solute carrier family 12 member 9 OS=Homo sapiens OX=9606 GN=SLC12A9 PE=1 SV=1	SLC12A9	1.076	0.510477
Q9BXP5	Serrate RNA effector molecule homolog OS=Homo sapiens OX=9606 GN=SRRT PE=1 SV=1	SRRT	0.934	0.305716
Q9BXS4	Transmembrane protein 59 OS=Homo sapiens OX=9606 GN=TMEM59 PE=1 SV=1	TMEM59	1.024	0.908296
Q9BXS5	AP-1 complex subunit mu-1 OS=Homo sapiens OX=9606 GN=AP1M1 PE=1 SV=3	AP1M1	0.936	0.621177
Q9BXS6	Nucleolar and spindle-associated protein 1 OS=Homo sapiens OX=9606 GN=NUSAP1 PE=1 SV=1	NUSAP1	0.979	0.946831
Q9BXS9	Solute carrier family 26 member 6 OS=Homo sapiens OX=9606 GN=SLC26A6 PE=1 SV=1	SLC26A6	1.082	
Q9BXW7	Haloacid dehalogenase-like hydrolase domain-containing 5 OS=Homo sapiens OX=9606 GN=HDHD5 PE=1 SV=1	HDHD5	0.896	0.098325
Q9BXW9	Fanconi anemia group D2 protein OS=Homo sapiens OX=9606 GN=FANCD2 PE=1 SV=2	FANCD2	1.09	0.654506
Q9BXY0	Protein MAK16 homolog OS=Homo sapiens OX=9606 GN=MAK16 PE=1 SV=2	MAK16	0.966	0.691889
Q9BY42	Replication termination factor 2 OS=Homo sapiens OX=9606 GN=RTF2 PE=1 SV=3	RTF2	0.929	
Q9BY44	Eukaryotic translation initiation factor 2A OS=Homo sapiens OX=9606 GN=EIF2A PE=1 SV=3	EIF2A	0.995	0.959308
Q9BY50	Signal peptidase complex catalytic subunit SEC11C OS=Homo sapiens OX=9606 GN=SEC11C	SEC11C	1.114	0.450533
Q9BY77	Polymerase delta-interacting protein 3 OS=Homo sapiens OX=9606 GN=POLDIP3 PE=1	POLDIP3	0.947	0.135603
Q9BYC5	Alpha-(1,6)-fucosyltransferase OS=Homo sapiens OX=9606 GN=FUT8 PE=1 SV=2	FUT8	0.988	0.956847

Q9BYC9	39S ribosomal protein L20, mitochondrial OS=Homo sapiens OX=9606 GN=MRPL20 PE=1 SV=1	MRPL20	1.008	0.950137
Q9BYD1	39S ribosomal protein L13, mitochondrial OS=Homo sapiens OX=9606 GN=MRPL13 PE=1 SV=1	MRPL13	0.986	0.837583
Q9BYD2	39S ribosomal protein L9, mitochondrial OS=Homo sapiens OX=9606 GN=MRPL9 PE=1	MRPL9	1.043	0.663961
Q9BYD3	39S ribosomal protein L4, mitochondrial OS=Homo sapiens OX=9606 GN=MRPL4 PE=1	MRPL4	1.071	0.485277
Q9BYD6	39S ribosomal protein L1, mitochondrial OS=Homo sapiens OX=9606 GN=MRPL1 PE=1	MRPL1	0.958	0.594132
Q9BYG3	MKI67 FHA domain-interacting nucleolar phosphoprotein OS=Homo sapiens OX=9606 GN=NIFK PE=1 SV=1	NIFK	1.06	0.750386
Q9BYJ9	YTH domain-containing family protein 1 OS=Homo sapiens OX=9606 GN=YTHDF1 PE=1 SV=1	YTHDF1	1.109	0.189762
Q9BYN8	28S ribosomal protein S26, mitochondrial OS=Homo sapiens OX=9606 GN=MRPS26 PE=1 SV=1	MRPS26	1.027	0.674097
Q9BYT8	Neurolysin, mitochondrial OS=Homo sapiens OX=9606 GN=NLN PE=1 SV=1	NLN	1.074	0.221059
Q9BZA5	Putative gamma-taxilin 2 OS=Homo sapiens OX=9606 GN=TXLNGY PE=5 SV=3	TXLNGY	0.001	0.001
Q9BZE1	39S ribosomal protein L37, mitochondrial OS=Homo sapiens OX=9606 GN=MRPL37 PE=1 SV=2	MRPL37	0.946	0.178623
Q9BZE4	GTP-binding protein 4 OS=Homo sapiens OX=9606 GN=GTPBP4 PE=1 SV=3	GTPBP4	1.062	0.831931
Q9BZF1	Oxysterol-binding protein-related protein 8 OS=Homo sapiens OX=9606 GN=OSBPL8 PE=1 SV=3	OSBPL8	1.018	0.788567
Q9BZF9	Uveal autoantigen with coiled-coil domains and ankyrin repeats OS=Homo sapiens OX=9606 GN=UACA PE=1 SV=2	UACA	0.966	0.451693
Q9BZG1	Ras-related protein Rab-34 OS=Homo sapiens OX=9606 GN=RAB34 PE=1 SV=1	RAB34	0.954	0.166163

Q9BZH6	WD repeat-containing protein 11 OS=Homo sapiens OX=9606 GN=WDR11 PE=1 SV=1	WDR11	1.153	0.040667
Q9BZJ0	Crooked neck-like protein 1 OS=Homo sapiens OX=9606 GN=CRNKL1 PE=1 SV=4	CRNKL1	0.943	0.383012
Q9BZK7	F-box-like/WD repeat-containing protein TBL1XR1 OS=Homo sapiens OX=9606 GN=TBL1XR1 PE=1 SV=1	TBL1XR1	0.892	0.073423
Q9BZM5	UL16-binding protein 2 OS=Homo sapiens OX=9606 GN=ULBP2 PE=1 SV=1	ULBP2	0.831	0.052782
Q9BZZ5	Apoptosis inhibitor 5 OS=Homo sapiens OX=9606 GN=API5 PE=1 SV=3	API5	0.894	0.252551
Q9C005	Protein dpy-30 homolog OS=Homo sapiens OX=9606 GN=DPY30 PE=1 SV=1	DPY30	0.985	0.822969
Q9C0B1	Alpha-ketoglutarate-dependent dioxygenase FTO OS=Homo sapiens OX=9606 GN=FTO PE=1	FTO	0.815	0.360914
Q9C0B5	Palmitoyltransferase ZDHHC5 OS=Homo sapiens OX=9606 GN=ZDHHC5 PE=1 SV=2	ZDHHC5	0.945	0.695006
Q9C0C2	182 kDa tankyrase-1-binding protein OS=Homo sapiens OX=9606 GN=TNKS1BP1 PE=1	TNKS1BP 1	0.939	0.115144
Q9C0D9	Ethanolaminephosphotransferase 1 OS=Homo sapiens OX=9606 GN=SELENOI PE=1 SV=3	SELENOI	1.045	0.772237
Q9C0E8	Endoplasmic reticulum junction formation protein lunapark OS=Homo sapiens OX=9606 GN=LNPK PE=1 SV=2	LNPK	0.894	0.378284
Q9C0H2	Protein tweety homolog 3 OS=Homo sapiens OX=9606 GN=TTYH3 PE=1 SV=3	TTYH3	1.139	0.236725
Q9C0J8	pre-mRNA 3' end processing protein WDR33 OS=Homo sapiens OX=9606 GN=WDR33	WDR33	1.043	0.65104
Q9GZL7	Ribosome biogenesis protein WDR12 OS=Homo sapiens OX=9606 GN=WDR12 PE=1	WDR12	0.928	0.20497
Q9GZM5	Protein YIPF3 OS=Homo sapiens OX=9606 GN=YIPF3 PE=1 SV=1	YIPF3	0.925	0.5194
Q9GZM7	Tubulointerstitial nephritis antigen-like OS=Homo sapiens OX=9606 GN=TINAGL1 PE=1	TINAGL1	0.919	0.446802
Q9GZP8	Immortalization up-regulated protein OS=Homo sapiens OX=9606 GN=IMUP PE=1 SV=1	IMUP	0.848	0.165936

Q9GZP9	Derlin-2 OS=Homo sapiens OX=9606 GN=DERL2 PE=1	DERL2	0.872	0.240998
Q9GZR2	RNA exonuclease 4 OS=Homo sapiens OX=9606 GN=REXO4 PE=1 SV=2	REXO4	1.046	0.180323
Q9GZR7	ATP-dependent RNA helicase DDX24 OS=Homo sapiens OX=9606 GN=DDX24 PE=1	DDX24	1.029	0.904826
Q9GZS1	DNA-directed RNA polymerase I subunit RPA49 OS=Homo sapiens OX=9606 GN=POLR1E PE=1 SV=3	POLR1E	0.95	0.675583
Q9GZS3	WD repeat-containing protein 61 OS=Homo sapiens OX=9606 GN=WDR61 PE=1 SV=1	WDR61	1.007	0.994655
Q9GZT3	SRA stem-loop-interacting RNA-binding protein, mitochondrial OS=Homo sapiens OX=9606 GN=SLIRP PE=1 SV=1	SLIRP	0.993	0.898977
Q9GZT6	Coiled-coil domain-containing protein 90B, mitochondrial OS=Homo sapiens OX=9606 GN=CCDC90B PE=1 SV=2	CCDC90B	1.021	0.913461
Q9GZY8	Mitochondrial fission factor OS=Homo sapiens OX=9606 GN=MFF PE=1 SV=1	MFF	1.035	0.315541
Q9GZZ1	N-alpha-acetyltransferase 50 OS=Homo sapiens OX=9606 GN=NAA50 PE=1 SV=1	NAA50	1.321	0.096449
Q9H000	Probable E3 ubiquitin-protein ligase makorin-2 OS=Homo sapiens OX=9606 GN=MKRN2	MKRN2	0.883	
Q9H061	Transmembrane protein 126A OS=Homo sapiens OX=9606 GN=TMEM126A PE=1 SV=1	TMEM126A	1.003	0.983357
Q9H078	Caseinolytic peptidase B protein homolog OS=Homo sapiens OX=9606 GN=CLPB PE=1 SV=1	CLPB	1.017	0.801783
Q9H089	Large subunit GTPase 1 homolog OS=Homo sapiens OX=9606 GN=LSG1 PE=1 SV=2	LSG1	1.141	0.154181
Q9H0A0	RNA cytidine acetyltransferase OS=Homo sapiens OX=9606 GN=NAT10 PE=1 SV=2	NAT10	1.014	0.960062
Q9H0B6	Kinesin light chain 2 OS=Homo sapiens OX=9606 GN=KLC2 PE=1 SV=1	KLC2	1000	0.001
Q9H0C8	Integrin-linked kinase-associated serine/threonine phosphatase 2C OS=Homo sapiens OX=9606 GN=ILKAP PE=1 SV=1	ILKAP	0.877	0.064123

Q9H0D6	5'-3' exoribonuclease 2 OS=Homo sapiens OX=9606 GN=XRN2 PE=1 SV=1	XRN2	0.86	0.037874
Q9H0E2	Toll-interacting protein OS=Homo sapiens OX=9606 GN=TOLLIP PE=1 SV=1	TOLLIP	0.978	0.966999
Q9H0E3	Histone deacetylase complex subunit SAP130 OS=Homo sapiens OX=9606 GN=SAP130	SAP130	0.989	0.967036
Q9H0E9	Bromodomain-containing protein 8 OS=Homo sapiens OX=9606 GN=BRD8 PE=1 SV=2	BRD8	0.863	0.116756
Q9H0H0	Integrator complex subunit 2 OS=Homo sapiens OX=9606 GN=INTS2 PE=1 SV=2	INTS2	0.937	0.711057
Q9H0H5	Rac GTPase-activating protein 1 OS=Homo sapiens OX=9606 GN=RACGAP1 PE=1 SV=1	RACGAP1	0.944	0.358593
Q9H0P0	Cytosolic 5'-nucleotidase 3A OS=Homo sapiens OX=9606 GN=NT5C3A PE=1 SV=3	NT5C3A	1.098	0.158658
Q9H0R6	Glutamyl-tRNA(Gln) amidotransferase subunit A, mitochondrial OS=Homo sapiens OX=9606 GN=QRSL1 PE=1	QRSL1	1.118	0.072881
Q9H0S4	Probable ATP-dependent RNA helicase DDX47 OS=Homo sapiens OX=9606 GN=DDX47	DDX47	0.905	0.338613
Q9H0U3	Magnesium transporter protein 1 OS=Homo sapiens OX=9606 GN=MAGT1 PE=1 SV=1	MAGT1	1.002	0.952153
Q9H0U4	Ras-related protein Rab-1B OS=Homo sapiens OX=9606 GN=RAB1B PE=1 SV=1	RAB1B	0.942	0.334341
Q9H0U9	Testis-specific Y-encoded-like protein 1 OS=Homo sapiens OX=9606 GN=TSPYL1 PE=1	TSPYL1	1.026	0.727121
Q9H0V9	VIP36-like protein OS=Homo sapiens OX=9606 GN=LMAN2L PE=1 SV=1	LMAN2L	0.973	0.790019
Q9H147	Deoxynucleotidyltransferase terminal-interacting protein 1 OS=Homo sapiens OX=9606 GN=DNTTIP1 PE=1 SV=2	DNTTIP1	1.029	0.543684
Q9H173	Nucleotide exchange factor SIL1 OS=Homo sapiens OX=9606 GN=SIL1 PE=1 SV=1	SIL1	1.056	0.679401
Q9H1B7	Probable E3 ubiquitin-protein ligase IRF2BPL OS=Homo sapiens OX=9606 GN=IRF2BPL PE=1 SV=1	IRF2BPL	0.859	0.016499

Q9H1C4	Protein unc-93 homolog B1 OS=Homo sapiens OX=9606 GN=UNC93B1 PE=1 SV=2	UNC93B1	0.992	0.990239
Q9H1E3	Nuclear ubiquitous casein and cyclin-dependent kinase substrate 1 OS=Homo sapiens OX=9606 GN=NUCKS1 PE=1 SV=1	NUCKS1	0.829	0.288363
Q9H1E5	Thioredoxin-related transmembrane protein 4 OS=Homo sapiens OX=9606	TMX4	1.075	0.017124
Q9H1I8	Activating signal cointegrator 1 complex subunit 2 OS=Homo sapiens OX=9606 GN=ASCC2 PE=1 SV=3	ASCC2	1.237	0.06998
Q9H1K1	Iron-sulfur cluster assembly enzyme ISCU, mitochondrial OS=Homo sapiens OX=9606 GN=ISCU PE=1 SV=2	ISCU	0.923	0.398572
Q9H204	Mediator of RNA polymerase II transcription subunit 28 OS=Homo sapiens OX=9606 GN=MED28 PE=1 SV=1	MED28	0.697	
Q9H223	EH domain-containing protein 4 OS=Homo sapiens OX=9606 GN=EHD4 PE=1 SV=1	EHD4	1.084	0.171786
Q9H269	Vacuolar protein sorting-associated protein 16 homolog OS=Homo sapiens OX=9606 GN=VPS16 PE=1 SV=2	VPS16	0.976	
Q9H299	SH3 domain-binding glutamic acid-rich-like protein 3 OS=Homo sapiens OX=9606 GN=SH3BGRL3 PE=1 SV=1	SH3BGRL3	0.951	0.703542
Q9H2D1	Mitochondrial folate transporter/carrier OS=Homo sapiens OX=9606 GN=SLC25A32 PE=1 SV=2	SLC25A32	0.934	0.403798
Q9H2D6	TRIO and F-actin-binding protein OS=Homo sapiens OX=9606 GN=TRIOBP PE=1 SV=3	TRIOBP	0.896	0.097498
Q9H2H9	Sodium-coupled neutral amino acid transporter 1 OS=Homo sapiens OX=9606 GN=SLC38A1 PE=1 SV=1	SLC38A1	1.078	0.550161
Q9H2P0	Activity-dependent neuroprotector homeobox protein OS=Homo sapiens OX=9606 GN=ADNP PE=1 SV=1	ADNP	0.923	0.17333
Q9H2U1	ATP-dependent DNA/RNA helicase DHX36 OS=Homo sapiens OX=9606 GN=DHX36	DHX36	1.023	0.810314

Q9H2U2	Inorganic pyrophosphatase 2, mitochondrial OS=Homo sapiens OX=9606 GN=PPA2 PE=1 SV=2	PPA2	1.021	0.530797
Q9H2V7	Protein spinster homolog 1 OS=Homo sapiens OX=9606 GN=SPNS1 PE=1 SV=1	SPNS1	0.939	0.495806
Q9H2W6	39S ribosomal protein L46, mitochondrial OS=Homo sapiens OX=9606 GN=MRPL46 PE=1 SV=1	MRPL46	1.065	0.343283
Q9H300	Presenilins-associated rhomboid-like protein, mitochondrial OS=Homo sapiens OX=9606 GN=PARL PE=1 SV=2	PARL	1.115	0.335037
Q9H307	Pinin OS=Homo sapiens OX=9606 GN=PNN PE=1 SV=5	PNN	0.94	0.400806
Q9H3D4	Tumor protein 63 OS=Homo sapiens OX=9606 GN=TP63 PE=1	TP63	0.955	0.670403
Q9H3K2	Growth hormone-inducible transmembrane protein OS=Homo sapiens OX=9606 GN=GHITM PE=1 SV=2	GHITM	0.921	0.285931
Q9H3K6	BolA-like protein 2 OS=Homo sapiens OX=9606 GN=BOLA2 PE=1 SV=1	BOLA2	0.973	0.837581
Q9H3N1	Thioredoxin-related transmembrane protein 1 OS=Homo sapiens OX=9606	TMX1	0.949	0.467859
Q9H3P2	Negative elongation factor A OS=Homo sapiens OX=9606 GN=NELFA PE=1 SV=3	NELFA	0.929	0.578015
Q9H3P7	Golgi resident protein GCP60 OS=Homo sapiens OX=9606 GN=ACBD3 PE=1 SV=4	ACBD3	0.772	0.035764
Q9H3U1	Protein unc-45 homolog A OS=Homo sapiens OX=9606 GN=UNC45A PE=1 SV=1	UNC45A	1.152	0.302595
Q9H3Z4	DnaJ homolog subfamily C member 5 OS=Homo sapiens OX=9606 GN=DNAJC5 PE=1	DNAJC5	0.641	0.009217
Q9H444	Charged multivesicular body protein 4b OS=Homo sapiens OX=9606 GN=CHMP4B PE=1	CHMP4B	0.899	0.3196
Q9H488	GDP-fucose protein O-fucosyltransferase 1 OS=Homo sapiens OX=9606 GN=POFUT1 PE=1 SV=1	POFUT1	1.001	0.976112
Q9H490	Phosphatidylinositol glycan anchor biosynthesis class U protein OS=Homo sapiens OX=9606 GN=PIGU PE=1 SV=3	PIGU	1.016	0.803027

Q9H4A6	Golgi phosphoprotein 3 OS=Homo sapiens OX=9606 GN=GOLPH3 PE=1 SV=1	GOLPH3	0.977	0.843678
Q9H4G0	Band 4.1-like protein 1 OS=Homo sapiens OX=9606 GN=EPB41L1 PE=1 SV=2	EPB41L1	0.85	0.102549
Q9H4L4	Sentrin-specific protease 3 OS=Homo sapiens OX=9606 GN=SENP3 PE=1 SV=2	SENP3	1.161	0.176491
Q9H4M9	EH domain-containing protein 1 OS=Homo sapiens OX=9606 GN=EHD1 PE=1 SV=2	EHD1	1.172	0.069555
Q9H501	ESF1 homolog OS=Homo sapiens OX=9606 GN=ESF1 PE=1 SV=1	ESF1	0.964	0.50751
Q9H553	Alpha-1,3/1,6-mannosyltransferase ALG2 OS=Homo sapiens OX=9606 GN=ALG2 PE=1 SV=1	ALG2	1.049	0.495762
Q9H583	HEAT repeat-containing protein 1 OS=Homo sapiens OX=9606 GN=HEATR1 PE=1 SV=3	HEATR1	0.983	0.794264
Q9H5K3	Protein O-mannose kinase OS=Homo sapiens OX=9606 GN=POMK PE=1 SV=1	POMK	1.037	0.702679
Q9H5Q4	Dimethyladenosine transferase 2, mitochondrial OS=Homo sapiens OX=9606 GN=TFB2M PE=1	TFB2M	0.953	0.435716
Q9H5V8	CUB domain-containing protein 1 OS=Homo sapiens OX=9606 GN=CDCP1 PE=1 SV=3	CDCP1	1.062	0.655604
Q9H5Y7	SLIT and NTRK-like protein 6 OS=Homo sapiens OX=9606 GN=SLITRK6 PE=2 SV=3	SLITRK6	1.094	0.241557
Q9H6E4	Coiled-coil domain-containing protein 134 OS=Homo sapiens OX=9606 GN=CCDC134 PE=1 SV=1	CCDC134	1.053	0.465111
Q9H6F5	Coiled-coil domain-containing protein 86 OS=Homo sapiens OX=9606 GN=CCDC86 PE=1 SV=1	CCDC86	0.949	0.512508
Q9H6H4	Receptor expression-enhancing protein 4 OS=Homo sapiens OX=9606 GN=REEP4 PE=1	REEP4	0.895	0.340943
Q9H6K4	Optic atrophy 3 protein OS=Homo sapiens OX=9606 GN=OPA3 PE=1 SV=1	OPA3	0.917	0.390834
Q9H6R0	ATP-dependent RNA helicase DHX33 OS=Homo sapiens OX=9606 GN=DHX33 PE=1	DHX33	0.969	
Q9H6R4	Nucleolar protein 6 OS=Homo sapiens OX=9606 GN=NOL6 PE=1 SV=2	NOL6	0.988	0.834249

Q9H6S3	Epidermal growth factor receptor kinase substrate 8-like protein 2 OS=Homo sapiens OX=9606 GN=EPS8L2 PE=1 SV=2	EPS8L2	1.054	0.68831
Q9H6T3	RNA polymerase II-associated protein 3 OS=Homo sapiens OX=9606 GN=RPAP3 PE=1	RPAP3	1.013	0.894137
Q9H6V9	Lipid droplet-associated hydrolase OS=Homo sapiens OX=9606 GN=LDAH PE=1 SV=1	LDAH	1.007	0.953104
Q9H6X2	Anthrax toxin receptor 1 OS=Homo sapiens OX=9606 GN=ANTXR1 PE=1 SV=2	ANTXR1	0.915	0.177574
Q9H6Y2	WD repeat-containing protein 55 OS=Homo sapiens OX=9606 GN=WDR55 PE=1 SV=2	WDR55	0.001	0.001
Q9H6Z4	Ran-binding protein 3 OS=Homo sapiens OX=9606 GN=RANBP3 PE=1 SV=1	RANBP3	0.857	0.314198
Q9H773	dCTP pyrophosphatase 1 OS=Homo sapiens OX=9606 GN=DCTPP1 PE=1 SV=1	DCTPP1	0.878	0.310925
Q9H7B2	Ribosome production factor 2 homolog OS=Homo sapiens OX=9606 GN=RPF2 PE=1 SV=2	RPF2	1.22	0.472188
Q9H7B4	Histone-lysine N-methyltransferase SMYD3 OS=Homo sapiens OX=9606 GN=SMYD3 PE=1	SMYD3	1000	0.001
Q9H7D7	WD repeat-containing protein 26 OS=Homo sapiens OX=9606 GN=WDR26 PE=1 SV=3	WDR26	1.129	0.279643
Q9H7F0	Polyamine-transporting ATPase 13A3 OS=Homo sapiens OX=9606 GN=ATP13A3 PE=1	ATP13A3	1.043	0.586239
Q9H7N4	Splicing factor, arginine/serine-rich 19 OS=Homo sapiens OX=9606 GN=SCAF1 PE=1	SCAF1	0.87	0.004589
Q9H7Z7	Prostaglandin E synthase 2 OS=Homo sapiens OX=9606 GN=PTGES2 PE=1 SV=1	PTGES2	1.076	0.331587
Q9H814	Phosphorylated adapter RNA export protein OS=Homo sapiens OX=9606 GN=PHAX PE=1 SV=1	PHAX	0.738	0.360561
Q9H845	Complex I assembly factor ACAD9, mitochondrial OS=Homo sapiens OX=9606 GN=ACAD9	ACAD9	0.929	0.335359
Q9H857	5'-nucleotidase domain-containing protein 2 OS=Homo sapiens OX=9606 GN=NT5DC2 PE=1 SV=1	NT5DC2	1.061	0.530499
Q9H8H0	Nucleolar protein 11 OS=Homo sapiens OX=9606 GN=NOL11 PE=1 SV=1	NOL11	1.042	0.2936

Q9H8H2	Probable ATP-dependent RNA helicase DDX31 OS=Homo sapiens OX=9606 GN=DDX31	DDX31	1.13	0.383742
Q9H8H3	Methyltransferase-like protein 7A OS=Homo sapiens OX=9606 GN=METTL7A PE=1 SV=1	METTL7A	0.861	0.13577
Q9H8V3	Protein ECT2 OS=Homo sapiens OX=9606 GN=ECT2 PE=1 SV=4	ECT2	1.115	0.556924
Q9H8Y8	Golgi reassembly-stacking protein 2 OS=Homo sapiens OX=9606 GN=GORASP2 PE=1 SV=3	GORASP2	1.023	0.771502
Q9H910	Jupiter microtubule associated homolog 2 OS=Homo sapiens OX=9606 GN=JPT2 PE=1 SV=1	JPT2	0.798	0.159058
Q9H936	Mitochondrial glutamate carrier 1 OS=Homo sapiens OX=9606 GN=SLC25A22 PE=1 SV=1	SLC25A22	0.933	0.093284
Q9H944	Mediator of RNA polymerase II transcription subunit 20 OS=Homo sapiens OX=9606 GN=MED20 PE=1 SV=1	MED20	1.062	0.461357
Q9H9A5	CCR4-NOT transcription complex subunit 10 OS=Homo sapiens OX=9606 GN=CNOT10 PE=1 SV=1	CNOT10	0.66	
Q9H9B1	Histone-lysine N-methyltransferase EHMT1 OS=Homo sapiens OX=9606 GN=EHMT1 PE=1	EHMT1	0.884	0.289655
Q9H9B4	Sideroflexin-1 OS=Homo sapiens OX=9606 GN=SFXN1 PE=1	SFXN1	0.959	0.417456
Q9H9E3	Conserved oligomeric Golgi complex subunit 4 OS=Homo sapiens OX=9606 GN=COG4 PE=1 SV=3	COG4	1.009	0.785177
Q9H9J2	39S ribosomal protein L44, mitochondrial OS=Homo sapiens OX=9606 GN=MRPL44 PE=1 SV=1	MRPL44	0.895	0.145437
Q9H9L3	Interferon-stimulated 20 kDa exonuclease-like 2 OS=Homo sapiens OX=9606 GN=ISG20L2 PE=1 SV=1	ISG20L2	1.012	0.776228
Q9H9P8	L-2-hydroxyglutarate dehydrogenase, mitochondrial OS=Homo sapiens OX=9606 GN=L2HGDH PE=1 SV=3	L2HGDH	1.047	0.271722
Q9H9Q4	Non-homologous end-joining factor 1 OS=Homo sapiens OX=9606 GN=NHEJ1 PE=1	NHEJ1	1.037	0.731178
Q9H9T3	Elongator complex protein 3 OS=Homo sapiens OX=9606 GN=ELP3 PE=1 SV=2	ELP3	0.805	

Q9H9Y6	DNA-directed RNA polymerase I subunit RPA2 OS=Homo sapiens OX=9606 GN=POLR1B PE=1 SV=2	POLR1B	0.944	0.692247
Q9HAB3	Solute carrier family 52, riboflavin transporter, member 2 OS=Homo sapiens OX=9606 GN=SLC52A2 PE=1 SV=1	SLC52A2	1.053	
Q9HAF1	Chromatin modification-related protein MEAF6 OS=Homo sapiens OX=9606 GN=MEAF6 PE=1	MEAF6	1.136	0.495717
Q9HAN9	Nicotinamide/nicotinic acid mononucleotide adenylyltransferase 1 OS=Homo sapiens OX=9606 GN=NMNAT1	NMNAT1	0.945	0.65228
Q9HAU0	Pleckstrin homology domain-containing family A member 5 OS=Homo sapiens OX=9606 GN=PLEKHA5 PE=1 SV=1	PLEKHA5	0.843	0.066495
Q9HAV0	Guanine nucleotide-binding protein subunit beta-4 OS=Homo sapiens OX=9606 GN=GNB4	GNB4	1.118	0.307545
Q9HAV4	Exportin-5 OS=Homo sapiens OX=9606 GN=XPO5 PE=1 SV=1	XPO5	1.025	0.856203
Q9HAV7	GrpE protein homolog 1, mitochondrial OS=Homo sapiens OX=9606 GN=GRPEL1 PE=1 SV=2	GRPEL1	1.056	0.290145
Q9HAW8	UDP-glucuronosyltransferase 1A10 OS=Homo sapiens OX=9606 GN=UGT1A10 PE=1	UGT1A10	1.078	0.413972
Q9HB07	MYG1 exonuclease OS=Homo sapiens OX=9606 GN=MYG1 PE=1 SV=3	MYG1	0.954	0.650808
Q9HB40	Retinoid-inducible serine carboxypeptidase OS=Homo sapiens OX=9606 GN=SCPEP1	SCPEP1	1.062	0.425698
Q9HB63	Netrin-4 OS=Homo sapiens OX=9606 GN=NTN4 PE=1 SV=2	NTN4	1.185	
Q9HB71	Calcyclin-binding protein OS=Homo sapiens OX=9606 GN=CACYBP PE=1 SV=2	CACYBP	1.106	0.427264
Q9HBH5	Retinol dehydrogenase 14 OS=Homo sapiens OX=9606 GN=RDH14 PE=1 SV=1	RDH14	0.875	0.192324
Q9HBI6	Cytochrome P450 4F11 OS=Homo sapiens OX=9606 GN=CYP4F11 PE=1 SV=3	CYP4F11	1.458	
Q9HC07	Transmembrane protein 165 OS=Homo sapiens OX=9606 GN=TMEM165 PE=1 SV=1	TMEM165	0.88	0.407279

Q9HC35	Echinoderm microtubule-associated protein-like 4 OS=Homo sapiens OX=9606	EML4	1.337	0.003871
Q9HC36	rRNA methyltransferase 3, mitochondrial OS=Homo sapiens OX=9606 GN=MRM3 PE=1	MRM3	0.793	0.047454
Q9HC38	Glyoxalase domain-containing protein 4 OS=Homo sapiens OX=9606 GN=GLOD4 PE=1	GLOD4	1.048	0.874184
Q9HC52	Chromobox protein homolog 8 OS=Homo sapiens OX=9606 GN=CBX8 PE=1 SV=3	CBX8	0.953	
Q9HCC0	Methylcrotonoyl-CoA carboxylase beta chain, mitochondrial OS=Homo sapiens OX=9606 GN=MCCC2 PE=1 SV=1	MCCC2	1.007	0.883401
Q9HCD5	Nuclear receptor coactivator 5 OS=Homo sapiens OX=9606 GN=NCOA5 PE=1 SV=2	NCOA5	0.969	0.525449
Q9HCD6	Protein TANC2 OS=Homo sapiens OX=9606 GN=TANC2 PE=1	TANC2	1000	0.001
Q9HCG8	Pre-mRNA-splicing factor CWC22 homolog OS=Homo sapiens OX=9606 GN=CWC22 PE=1	CWC22	1.021	
Q9HCN8	Stromal cell-derived factor 2-like protein 1 OS=Homo sapiens OX=9606 GN=SDF2L1 PE=1	SDF2L1	1.07	0.377845
Q9HCS7	Pre-mRNA-splicing factor SYF1 OS=Homo sapiens OX=9606 GN=XAB2 PE=1 SV=2	XAB2	0.952	0.685961
Q9HCU5	Prolactin regulatory element-binding protein OS=Homo sapiens OX=9606 GN=PREB PE=1 SV=2	PREB	1.071	0.556859
Q9HCY8	Protein S100-A14 OS=Homo sapiens OX=9606 GN=S100A14 PE=1 SV=1	S100A14	0.972	0.753465
Q9HD26	Golgi-associated PDZ and coiled-coil motif-containing protein OS=Homo sapiens OX=9606 GN=GOPC PE=1 SV=1	GOPC	0.716	0.006095
Q9HD33	39S ribosomal protein L47, mitochondrial OS=Homo sapiens OX=9606 GN=MRPL47 PE=1 SV=2	MRPL47	0.995	0.903782
Q9HD45	Transmembrane 9 superfamily member 3 OS=Homo sapiens OX=9606 GN=TM9SF3 PE=1	TM9SF3	0.92	0.341271
Q9HDC9	Adipocyte plasma membrane-associated protein OS=Homo sapiens OX=9606 GN=APMAP PE=1 SV=2	APMAP	1.006	0.907548

Q9NNW7	Thioredoxin reductase 2, mitochondrial OS=Homo sapiens OX=9606 GN=TXNRD2 PE=1 SV=3	TXNRD2	0.918	0.078183
Q9NP66	High mobility group protein 20A OS=Homo sapiens OX=9606 GN=HMG20A PE=1 SV=1	HMG20A	0.921	0.351821
Q9NP72	Ras-related protein Rab-18 OS=Homo sapiens OX=9606 GN=RAB18 PE=1 SV=1	RAB18	0.995	0.684604
Q9NP77	RNA polymerase II subunit A C-terminal domain phosphatase SSU72 OS=Homo sapiens OX=9606 GN=SSU72 PE=1 SV=1	SSU72	0.839	0.025091
Q9NP79	Vacuolar protein sorting-associated protein VTA1 homolog OS=Homo sapiens OX=9606 GN=VTA1 PE=1 SV=1	VTA1	0.942	0.316218
Q9NP81	Serine--tRNA ligase, mitochondrial OS=Homo sapiens OX=9606 GN=SARS2 PE=1	SARS2	0.879	0.089831
Q9NP84	Tumor necrosis factor receptor superfamily member 12A OS=Homo sapiens OX=9606 GN=TNFRSF12A PE=1 SV=1	TNFRSF12A	1.083	0.412801
Q9NP92	39S ribosomal protein S30, mitochondrial OS=Homo sapiens OX=9606 GN=MRPS30 PE=1 SV=2	MRPS30	0.968	0.707212
Q9NP97	Dynein light chain roadblock-type 1 OS=Homo sapiens OX=9606 GN=DYNLRB1 PE=1 SV=3	DYNLRB1	1.402	0.162587
Q9NPA0	ER membrane protein complex subunit 7 OS=Homo sapiens OX=9606 GN=EMC7 PE=1 SV=1	EMC7	0.974	0.753025
Q9NPA8	Transcription and mRNA export factor ENY2 OS=Homo sapiens OX=9606 GN=ENY2 PE=1 SV=1	ENY2	0.815	0.010089
Q9NPD3	Exosome complex component RRP41 OS=Homo sapiens OX=9606 GN=EXOSC4 PE=1 SV=3	EXOSC4	0.979	0.594334
Q9NPE3	H/ACA ribonucleoprotein complex subunit 3 OS=Homo sapiens OX=9606 GN=NOP10 PE=1	NOP10	1.106	0.271529
Q9NPF5	DNA methyltransferase 1-associated protein 1 OS=Homo sapiens OX=9606 GN=DMAP1	DMAP1	1.007	0.856229
Q9NPH0	Lysophosphatidic acid phosphatase type 6 OS=Homo sapiens OX=9606 GN=ACP6 PE=1 SV=3	ACP6	1.279	0.200602

Q9NPH3	Interleukin-1 receptor accessory protein OS=Homo sapiens OX=9606 GN=IL1RAP PE=1	IL1RAP	0.985	0.843409
Q9NPI1	Bromodomain-containing protein 7 OS=Homo sapiens OX=9606 GN=BRD7 PE=1 SV=1	BRD7	1.033	0.725706
Q9NPJ6	Mediator of RNA polymerase II transcription subunit 4 OS=Homo sapiens OX=9606 GN=MED4 PE=1 SV=1	MED4	0.882	0.050531
Q9NPL8	Complex I assembly factor TIMMDC1, mitochondrial OS=Homo sapiens OX=9606 GN=TIMMDC1 PE=1 SV=2	TIMMDC1	0.835	0.026675
Q9NPR2	Semaphorin-4B OS=Homo sapiens OX=9606 GN=SEMA4B PE=1 SV=4	SEMA4B	1.008	0.919685
Q9NQ29	Putative RNA-binding protein Luc7-like 1 OS=Homo sapiens OX=9606 GN=LUC7L PE=1	LUC7L	1.261	
Q9NQ50	39S ribosomal protein L40, mitochondrial OS=Homo sapiens OX=9606 GN=MRPL40 PE=1 SV=1	MRPL40	0.964	0.78485
Q9NQ55	Suppressor of SWI4 1 homolog OS=Homo sapiens OX=9606 GN=PPAN PE=2 SV=1	PPAN	1.08	0.729089
Q9NQC3	Reticulon-4 OS=Homo sapiens OX=9606 GN=RTN4 PE=1 SV=2	RTN4	0.929	0.141375
Q9NQE9	Adenosine 5'-monophosphoramidase HINT3 OS=Homo sapiens OX=9606	HINT3	1.035	0.734494
Q9NQG1	Protein MANBAL OS=Homo sapiens OX=9606 GN=MANBAL PE=1 SV=1	MANBAL	1.305	0.213994
Q9NQG5	Regulation of nuclear pre-mRNA domain-containing protein 1B OS=Homo sapiens OX=9606 GN=RPRD1B PE=1 SV=1	RPRD1B	0.894	0.214981
Q9NQH7	Xaa-Pro aminopeptidase 3 OS=Homo sapiens OX=9606 GN=XPNPEP3 PE=1 SV=1	XPNPEP3	1.014	0.807558
Q9NQP4	Prefoldin subunit 4 OS=Homo sapiens OX=9606 GN=PFDN4 PE=1 SV=1	PFDN4	1.096	0.788723
Q9NQR4	Omega-amidase NIT2 OS=Homo sapiens OX=9606 GN=NIT2 PE=1 SV=1	NIT2	1.084	0.436484
Q9NQS7	Inner centromere protein OS=Homo sapiens OX=9606 GN=INCENP PE=1 SV=3	INCENP	1.161	0.004819

Q9NQT4	Exosome complex component RRP46 OS=Homo sapiens OX=9606 GN=EXOSC5 PE=1 SV=1	EXOSC5	0.853	0.050909
Q9NQT5	Exosome complex component RRP40 OS=Homo sapiens OX=9606 GN=EXOSC3 PE=1 SV=3	EXOSC3	0.908	0.224898
Q9NQT8	Kinesin-like protein KIF13B OS=Homo sapiens OX=9606 GN=KIF13B PE=1 SV=2	KIF13B	0.954	0.632983
Q9NQW6	Anillin OS=Homo sapiens OX=9606 GN=ANLN PE=1 SV=2	ANLN	0.9	0.208992
Q9NQX7	Integral membrane protein 2C OS=Homo sapiens OX=9606 GN=ITM2C PE=1 SV=1	ITM2C	1.018	0.619358
Q9NQZ2	Something about silencing protein 10 OS=Homo sapiens OX=9606 GN=UTP3 PE=1 SV=1	UTP3	0.923	0.405401
Q9NR12	PDZ and LIM domain protein 7 OS=Homo sapiens OX=9606 GN=PDLIM7 PE=1 SV=1	PDLIM7	1.125	0.32445
Q9NR28	Diablo homolog, mitochondrial OS=Homo sapiens OX=9606 GN=DIABLO PE=1 SV=1	DIABLO	1.052	0.362505
Q9NR30	Nucleolar RNA helicase 2 OS=Homo sapiens OX=9606 GN=DDX21 PE=1 SV=5	DDX21	0.983	0.827917
Q9NR31	GTP-binding protein SAR1a OS=Homo sapiens OX=9606 GN=SAR1A PE=1 SV=1	SAR1A	1.136	0.253206
Q9NR33	DNA polymerase epsilon subunit 4 OS=Homo sapiens OX=9606 GN=POLE4 PE=1 SV=2	POLE4	1000	0.001
Q9NR45	Sialic acid synthase OS=Homo sapiens OX=9606 GN=NANS PE=1 SV=2	NANS	0.842	0.097855
Q9NR50	Translation initiation factor eIF-2B subunit gamma OS=Homo sapiens OX=9606 GN=EIF2B3 PE=1	EIF2B3	1.146	0.415197
Q9NR56	Muscleblind-like protein 1 OS=Homo sapiens OX=9606 GN=MBNL1 PE=1 SV=2	MBNL1	0.913	0.389295
Q9NR77	Peroxisomal membrane protein 2 OS=Homo sapiens OX=9606 GN=PXMP2 PE=1 SV=3	PXMP2	1.013	0.849949
Q9NRF9	DNA polymerase epsilon subunit 3 OS=Homo sapiens OX=9606 GN=POLE3 PE=1 SV=1	POLE3	0.729	0.027053
Q9NRG0	Chromatin accessibility complex protein 1 OS=Homo sapiens OX=9606 GN=CHRAC1 PE=1 SV=1	CHRAC1	1.296	0.045637

Q9NRG9	Aladin OS=Homo sapiens OX=9606 GN=AAAS PE=1 SV=1	AAAS	1.006	0.932173
Q9NRJ7	Protocadherin beta-16 OS=Homo sapiens OX=9606 GN=PCDHB16 PE=1 SV=4	PCDHB16	0.967	0.298573
Q9NRL2	Bromodomain adjacent to zinc finger domain protein 1A OS=Homo sapiens OX=9606 GN=BAZ1A PE=1 SV=2	BAZ1A	1.095	0.578203
Q9NRL3	Striatin-4 OS=Homo sapiens OX=9606 GN=STRN4 PE=1	STRN4	1.023	0.889871
Q9NRN7	L-aminoadipate-semialdehyde dehydrogenase-phosphopantetheinyl transferase OS=Homo sapiens OX=9606 GN=AASDHPPT PE=1 SV=2	AASDHPPT	1.609	
Q9NRP2	COX assembly mitochondrial protein 2 homolog OS=Homo sapiens OX=9606 GN=CMC2 PE=1 SV=1	CMC2	0.86	0.0273
Q9NRR8	CDC42 small effector protein 1 OS=Homo sapiens OX=9606 GN=CDC42SE1 PE=1 SV=1	CDC42SE1	1.257	0.267984
Q9NRV9	Heme-binding protein 1 OS=Homo sapiens OX=9606 GN=HEBP1 PE=1 SV=1	HEBP1	1.157	0.206209
Q9NRW7	Vacuolar protein sorting-associated protein 45 OS=Homo sapiens OX=9606 GN=VPS45 PE=1 SV=1	VPS45	1.034	0.549999
Q9NRX1	RNA-binding protein PNO1 OS=Homo sapiens OX=9606 GN=PNO1 PE=1 SV=1	PNO1	0.969	0.773431
Q9NRX2	39S ribosomal protein L17, mitochondrial OS=Homo sapiens OX=9606 GN=MRPL17 PE=1 SV=1	MRPL17	1.012	0.906324
Q9NRX5	Serine incorporator 1 OS=Homo sapiens OX=9606 GN=SERINC1 PE=1 SV=1	SERINC1	0.91	0.395256
Q9NRY2	SOSS complex subunit C OS=Homo sapiens OX=9606 GN=INIP PE=1 SV=1	INIP	0.875	0.368938
Q9NRY6	Phospholipid scramblase 3 OS=Homo sapiens OX=9606 GN=PLSCR3 PE=1 SV=2	PLSCR3	1.065	0.524613
Q9NS00	Glycoprotein-N-acetylgalactosamine 3-beta-galactosyltransferase 1 OS=Homo sapiens OX=9606 GN=C1GALT1	C1GALT1	0.905	0.373502
Q9NS69	Mitochondrial import receptor subunit TOM22 homolog OS=Homo sapiens OX=9606 GN=TOMM22 PE=1 SV=3	TOMM22	0.924	0.140694

Q9NS91	E3 ubiquitin-protein ligase RAD18 OS=Homo sapiens OX=9606 GN=RAD18 PE=1 SV=2	RAD18	1.01	0.950615
Q9NSD9	Phenylalanine--tRNA ligase beta subunit OS=Homo sapiens OX=9606 GN=FARSB PE=1	FARSB	1.018	0.813572
Q9NSE4	Isoleucine--tRNA ligase, mitochondrial OS=Homo sapiens OX=9606 GN=IARS2 PE=1 SV=2	IARS2	0.975	0.623169
Q9NTI5	Sister chromatid cohesion protein PDS5 homolog B OS=Homo sapiens OX=9606 GN=PDS5B	PDS5B	0.874	0.325383
Q9NTJ3	Structural maintenance of chromosomes protein 4 OS=Homo sapiens OX=9606 GN=SMC4 PE=1 SV=2	SMC4	1.014	0.799217
Q9NTJ5	Phosphatidylinositol-3-phosphatase SAC1 OS=Homo sapiens OX=9606 GN=SACM1L	SACM1L	0.99	0.891142
Q9NTK5	Obg-like ATPase 1 OS=Homo sapiens OX=9606 GN=OLA1 PE=1 SV=2	OLA1	1.058	0.599325
Q9NTX5	Ethylmalonyl-CoA decarboxylase OS=Homo sapiens OX=9606 GN=ECHDC1 PE=1 SV=2	ECHDC1	0.456	0.191891
Q9NTZ6	RNA-binding protein 12 OS=Homo sapiens OX=9606 GN=RBM12 PE=1 SV=1	RBM12	0.922	0.454055
Q9NU22	Midasin OS=Homo sapiens OX=9606 GN=MDN1 PE=1 SV=2	MDN1	0.926	0.180748
Q9NU23	LYR motif-containing protein 2 OS=Homo sapiens OX=9606 GN=LYRM2 PE=1 SV=1	LYRM2	0.877	0.014544
Q9NUJ1	Palmitoyl-protein thioesterase ABHD10, mitochondrial OS=Homo sapiens OX=9606 GN=ABHD10 PE=1 SV=1	ABHD10	0.974	0.730605
Q9NUM4	Transmembrane protein 106B OS=Homo sapiens OX=9606 GN=TMEM106B PE=1 SV=2	TMEM106B	0.999	0.917858
Q9NUN5	Lysosomal cobalamin transport escort protein LMBD1 OS=Homo sapiens OX=9606 GN=LMBRD1 PE=1 SV=1	LMBRD1	0.982	0.891998
Q9NUP9	Protein lin-7 homolog C OS=Homo sapiens OX=9606 GN=LIN7C PE=1 SV=1	LIN7C	0.859	0.188938
Q9NUQ2	1-acyl-sn-glycerol-3-phosphate acyltransferase epsilon OS=Homo sapiens OX=9606 GN=AGPAT5 PE=1 SV=3	AGPAT5	1.108	0.105043

Q9NUQ6	SPATS2-like protein OS=Homo sapiens OX=9606 GN=SPATS2L PE=1 SV=2	SPATS2L	1.075	0.245813
Q9NUQ7	Ufm1-specific protease 2 OS=Homo sapiens OX=9606 GN=UFSP2 PE=1 SV=3	UFSP2	0.947	0.557735
Q9NUQ9	CYFIP-related Rac1 interactor B OS=Homo sapiens OX=9606 GN=CYRIB PE=1 SV=1	CYRIB	1.013	0.808748
Q9NUU7	ATP-dependent RNA helicase DDX19A OS=Homo sapiens OX=9606 GN=DDX19A PE=1 SV=1	DDX19A	1.015	0.813867
Q9NV06	DDB1- and CUL4-associated factor 13 OS=Homo sapiens OX=9606 GN=DCAF13 PE=1	DCAF13	0.984	0.82707
Q9NV31	U3 small nucleolar ribonucleoprotein protein IMP3 OS=Homo sapiens OX=9606	IMP3	0.886	0.249638
Q9NV56	MRG/MORF4L-binding protein OS=Homo sapiens OX=9606 GN=MRGBP PE=1 SV=1	MRGBP	0.91	0.320406
Q9NV70	Exocyst complex component 1 OS=Homo sapiens OX=9606 GN=EXOC1 PE=1 SV=4	EXOC1	1.026	0.903725
Q9NV88	Integrator complex subunit 9 OS=Homo sapiens OX=9606 GN=INTS9 PE=1 SV=2	INTS9	1.145	0.067542
Q9NV92	NEDD4 family-interacting protein 2 OS=Homo sapiens OX=9606 GN=NDFIP2 PE=1 SV=2	NDFIP2	1.284	0.235342
Q9NV96	Cell cycle control protein 50A OS=Homo sapiens OX=9606 GN=TMEM30A PE=1 SV=1	TMEM30A	0.931	0.368172
Q9NVA2	Septin-11 OS=Homo sapiens OX=9606 GN=SEPTIN11 PE=1 SV=3	SEPTIN11	0.936	0.32223
Q9NVD7	Alpha-parvin OS=Homo sapiens OX=9606 GN=PARVA PE=1	PARVA	1.323	0.092682
Q9NVH0	Exonuclease 3'-5' domain-containing protein 2 OS=Homo sapiens OX=9606 GN=EXD2	EXD2	1.013	0.890866
Q9NVH1	DnaJ homolog subfamily C member 11 OS=Homo sapiens OX=9606 GN=DNAJC11 PE=1	DNAJC11	0.995	0.971153
Q9NVH2	Integrator complex subunit 7 OS=Homo sapiens OX=9606 GN=INTS7 PE=1 SV=1	INTS7	1.149	0.43445
Q9NVI1	Fanconi anemia group I protein OS=Homo sapiens OX=9606 GN=FANCI PE=1 SV=4	FANCI	0.952	0.695097

Q9NVI7	ATPase family AAA domain-containing protein 3A OS=Homo sapiens OX=9606 GN=ATAD3A PE=1 SV=2	ATAD3A	0.959	0.567565
Q9NVJ2	ADP-ribosylation factor-like protein 8B OS=Homo sapiens OX=9606 GN=ARL8B PE=1	ARL8B	0.975	0.786649
Q9NVM6	DnaJ homolog subfamily C member 17 OS=Homo sapiens OX=9606 GN=DNAJC17 PE=1	DNAJC17	1.129	
Q9NVM9	Integrator complex subunit 13 OS=Homo sapiens OX=9606 GN=INTS13 PE=1 SV=2	INTS13	0.953	0.501323
Q9NVP1	ATP-dependent RNA helicase DDX18 OS=Homo sapiens OX=9606 GN=DDX18 PE=1	DDX18	1.001	0.928936
Q9NVR2	Integrator complex subunit 10 OS=Homo sapiens OX=9606 GN=INTS10 PE=1 SV=2	INTS10	0.994	0.949389
Q9NVS2	39S ribosomal protein S18a, mitochondrial OS=Homo sapiens OX=9606 GN=MRPS18A PE=1 SV=1	MRPS18A	1.43	0.383287
Q9NVS9	Pyridoxine-5'-phosphate oxidase OS=Homo sapiens OX=9606 GN=PNPO PE=1 SV=1	PNPO	1.097	0.582433
Q9NVU7	Protein SDA1 homolog OS=Homo sapiens OX=9606 GN=SDAD1 PE=1 SV=3	SDAD1	0.974	0.814406
Q9NVV4	Poly(A) RNA polymerase, mitochondrial OS=Homo sapiens OX=9606 GN=MTPAP PE=1	MTPAP	0.972	0.748625
Q9NVX2	Notchless protein homolog 1 OS=Homo sapiens OX=9606 GN=NLE1 PE=1 SV=4	NLE1	0.947	0.379191
Q9NW13	RNA-binding protein 28 OS=Homo sapiens OX=9606 GN=RBM28 PE=1 SV=3	RBM28	1.019	0.946432
Q9NW15	Anoctamin-10 OS=Homo sapiens OX=9606 GN=ANO10 PE=1	ANO10	0.984	0.889937
Q9NW64	Pre-mRNA-splicing factor RBM22 OS=Homo sapiens OX=9606 GN=RBM22 PE=1 SV=1	RBM22	0.913	0.303851
Q9NW81	Distal membrane-arm assembly complex protein 2 OS=Homo sapiens OX=9606 GN=DMAC2 PE=1 SV=3	DMAC2	1.004	
Q9NW82	WD repeat-containing protein 70 OS=Homo sapiens OX=9606 GN=WDR70 PE=1 SV=1	WDR70	0.965	
Q9NWB6	Arginine and glutamate-rich protein 1 OS=Homo sapiens OX=9606 GN=ARGLU1 PE=1	ARGLU1	0.948	0.299577

Q9NWD8	Transmembrane protein 248 OS=Homo sapiens OX=9606 GN=TMEM248 PE=1 SV=1	TMEM248	1.193	0.217108
Q9NWH9	SAFB-like transcription modulator OS=Homo sapiens OX=9606 GN=SLTM PE=1 SV=2	SLTM	1.095	0.461543
Q9NWM8	Peptidyl-prolyl cis-trans isomerase FKBP14 OS=Homo sapiens OX=9606 GN=FKBP14 PE=1	FKBP14	0.896	0.26689
Q9NWT1	p21-activated protein kinase-interacting protein 1 OS=Homo sapiens OX=9606 GN=PAK1IP1 PE=1 SV=2	PAK1IP1	1.119	0.391755
Q9NWU1	3-oxoacyl-[acyl-carrier-protein] synthase, mitochondrial OS=Homo sapiens OX=9606 GN=OXSM PE=1 SV=1	OXSM	1.11	0.08738
Q9NWU2	Glucose-induced degradation protein 8 homolog OS=Homo sapiens OX=9606 GN=GID8 PE=1	GID8	1.005	0.897243
Q9NWU5	39S ribosomal protein L22, mitochondrial OS=Homo sapiens OX=9606 GN=MRPL22 PE=1 SV=1	MRPL22	1.167	0.095883
Q9NWW5	Ceroid-lipofuscinosis neuronal protein 6 OS=Homo sapiens OX=9606 GN=CLN6 PE=1 SV=1	CLN6	0.883	
Q9NX00	Transmembrane protein 160 OS=Homo sapiens OX=9606 GN=TMEM160 PE=1 SV=1	TMEM160	0.94	0.544112
Q9NX02	NACHT, LRR and PYD domains-containing protein 2 OS=Homo sapiens OX=9606 GN=NLRP2 PE=1 SV=1	NLRP2	1.14	0.256322
Q9NX14	NADH dehydrogenase [ubiquinone] 1 beta subcomplex subunit 11, mitochondrial OS=Homo sapiens OX=9606 GN=NDUFB11 PE=1 SV=1	NDUFB11	0.972	0.670195
Q9NX18	Succinate dehydrogenase assembly factor 2, mitochondrial OS=Homo sapiens OX=9606 GN=SDHAF2 PE=1 SV=1	SDHAF2	1.031	0.753865
Q9NX20	39S ribosomal protein L16, mitochondrial OS=Homo sapiens OX=9606 GN=MRPL16 PE=1 SV=1	MRPL16	0.87	0.352263
Q9NX24	H/ACA ribonucleoprotein complex subunit 2 OS=Homo sapiens OX=9606 GN=NHP2 PE=1 SV=1	NHP2	0.969	0.653586
Q9NX40	OCIA domain-containing protein 1 OS=Homo sapiens OX=9606 GN=OCIAD1 PE=1 SV=1	OCIAD1	0.986	0.85447

Q9NX47	E3 ubiquitin-protein ligase MARCHF5 OS=Homo sapiens OX=9606 GN=MARCHF5 PE=1 SV=1	MARCHF5	1.026	0.744413
Q9NX55	Huntingtin-interacting protein K OS=Homo sapiens OX=9606 GN=HYPK PE=1 SV=2	HYPK	1.289	0.198463
Q9NX58	Cell growth-regulating nucleolar protein OS=Homo sapiens OX=9606 GN=LYAR PE=1 SV=2	LYAR	0.887	0.350403
Q9NX62	Golgi-resident adenosine 3',5'-bisphosphate 3'-phosphatase OS=Homo sapiens OX=9606 GN=BPNT2 PE=1 SV=1	BPNT2	1.058	0.435909
Q9NX63	MICOS complex subunit MIC19 OS=Homo sapiens OX=9606 GN=CHCHD3 PE=1 SV=1	CHCHD3	1.041	0.568932
Q9NX76	CKLF-like MARVEL transmembrane domain-containing protein 6 OS=Homo sapiens OX=9606 GN=CMTM6 PE=1	CMTM6	1.323	0.007782
Q9NXE4	Sphingomyelin phosphodiesterase 4 OS=Homo sapiens OX=9606 GN=SMPD4 PE=1 SV=3	SMPD4	1.033	0.607554
Q9NXF1	Testis-expressed protein 10 OS=Homo sapiens OX=9606 GN=TEX10 PE=1 SV=2	TEX10	1.011	0.913133
Q9NXG2	THUMP domain-containing protein 1 OS=Homo sapiens OX=9606 GN=THUMPD1 PE=1	THUMPD1	0.903	0.320956
Q9NXV2	BTB/POZ domain-containing protein KCTD5 OS=Homo sapiens OX=9606 GN=KCTD5 PE=1	KCTD5	1.296	0.180214
Q9NXV6	CDKN2A-interacting protein OS=Homo sapiens OX=9606 GN=CDKN2AIP PE=1 SV=3	CDKN2AIP	1.003	0.934424
Q9NXW2	DnaJ homolog subfamily B member 12 OS=Homo sapiens OX=9606 GN=DNAJB12 PE=1	DNAJB12	1.029	0.814822
Q9NXX6	Non-structural maintenance of chromosomes element 4 homolog A OS=Homo sapiens OX=9606 GN=NSMCE4A PE=1 SV=2	NSMCE4A	0.871	0.182899
Q9NY12	H/ACA ribonucleoprotein complex subunit 1 OS=Homo sapiens OX=9606 GN=GAR1 PE=1 SV=1	GAR1	1.074	0.417927
Q9NY27	Serine/threonine-protein phosphatase 4 regulatory subunit 2 OS=Homo sapiens OX=9606 GN=PPP4R2 PE=1 SV=3	PPP4R2	0.724	0.002424
Q9NY33	Dipeptidyl peptidase 3 OS=Homo sapiens OX=9606 GN=DPP3 PE=1 SV=2	DPP3	0.994	0.955179

Q9NY35	Claudin domain-containing protein 1 OS=Homo sapiens OX=9606 GN=CLDND1 PE=1 SV=1	CLDND1	0.892	0.176781
Q9NY61	Protein AATF OS=Homo sapiens OX=9606 GN=AATF PE=1 SV=1	AATF	0.978	0.748307
Q9NY93	Probable ATP-dependent RNA helicase DDX56 OS=Homo sapiens OX=9606 GN=DDX56	DDX56	1.027	0.780959
Q9NYB0	Telomeric repeat-binding factor 2-interacting protein 1 OS=Homo sapiens OX=9606 GN=TERF2IP PE=1 SV=1	TERF2IP	0.965	
Q9NYF8	Bcl-2-associated transcription factor 1 OS=Homo sapiens OX=9606 GN=BCLAF1 PE=1	BCLAF1	1.031	0.664424
Q9NYH9	U3 small nucleolar RNA-associated protein 6 homolog OS=Homo sapiens OX=9606 GN=UTP6 PE=1 SV=2	UTP6	0.966	0.68488
Q9NYJ1	Cytochrome c oxidase assembly factor 4 homolog, mitochondrial OS=Homo sapiens OX=9606 GN=COA4 PE=1 SV=2	COA4	1.006	
Q9NYK5	39S ribosomal protein L39, mitochondrial OS=Homo sapiens OX=9606 GN=MRPL39 PE=1 SV=3	MRPL39	1.087	0.18637
Q9NYL4	Peptidyl-prolyl cis-trans isomerase FKBP11 OS=Homo sapiens OX=9606 GN=FKBP11 PE=1	FKBP11	1	0.908155
Q9NYL9	Tropomodulin-3 OS=Homo sapiens OX=9606 GN=TMOD3	TMOD3	1	0.989017
Q9NYM9	BET1-like protein OS=Homo sapiens OX=9606 GN=BET1L PE=1 SV=1	BET1L	1.123	
Q9NYU2	UDP-glucose:glycoprotein glucosyltransferase 1 OS=Homo sapiens OX=9606 GN=UGGT1 PE=1 SV=3	UGGT1	1.014	0.745043
Q9NYV4	Cyclin-dependent kinase 12 OS=Homo sapiens OX=9606 GN=CDK12 PE=1 SV=2	CDK12	0.972	0.655164
Q9NYY8	FAST kinase domain-containing protein 2, mitochondrial OS=Homo sapiens OX=9606 GN=FASTKD2 PE=1 SV=1	FASTKD2	0.973	0.614951
Q9NZ01	Very-long-chain enoyl-CoA reductase OS=Homo sapiens OX=9606 GN=TECR PE=1 SV=1	TECR	1.087	0.501126
Q9NZ08	Endoplasmic reticulum aminopeptidase 1 OS=Homo sapiens OX=9606 GN=ERAP1	ERAP1	0.995	0.575047

Q9NZ45	CDGSH iron-sulfur domain-containing protein 1 OS=Homo sapiens OX=9606 GN=CISD1 PE=1 SV=1	CISD1	0.955	0.710655
Q9NZB2	Constitutive coactivator of PPAR-gamma-like protein 1 OS=Homo sapiens OX=9606 GN=FAM120A PE=1 SV=2	FAM120A	0.881	0.077378
Q9NZC3	Glycerophosphodiester phosphodiesterase 1 OS=Homo sapiens OX=9606 GN=GDE1 PE=1 SV=1	GDE1	1.203	
Q9NZD8	Maspardin OS=Homo sapiens OX=9606 GN=SPG21 PE=1 SV=1	SPG21	1.121	0.160452
Q9NZI8	Insulin-like growth factor 2 mRNA-binding protein 1 OS=Homo sapiens OX=9606 GN=IGF2BP1 PE=1 SV=2	IGF2BP1	1.067	0.290246
Q9NZJ7	Mitochondrial carrier homolog 1 OS=Homo sapiens OX=9606 GN=MTCH1 PE=1 SV=1	MTCH1	1.063	0.243598
Q9NZL4	Hsp70-binding protein 1 OS=Homo sapiens OX=9606 GN=HSPBP1 PE=1 SV=2	HSPBP1	1.012	0.871748
Q9NZL9	Methionine adenosyltransferase 2 subunit beta OS=Homo sapiens OX=9606 GN=MAT2B PE=1	MAT2B	0.915	0.51116
Q9NZM1	Myoferlin OS=Homo sapiens OX=9606 GN=MYOF PE=1	MYOF	1.015	0.603477
Q9NZM5	Ribosome biogenesis protein NOP53 OS=Homo sapiens OX=9606 GN=NOP53 PE=1	NOP53	0.948	0.46148
Q9NZN4	EH domain-containing protein 2 OS=Homo sapiens OX=9606 GN=EHD2 PE=1 SV=2	EHD2	0.846	0.197919
Q9NZN8	CCR4-NOT transcription complex subunit 2 OS=Homo sapiens OX=9606 GN=CNOT2 PE=1	CNOT2	0.905	0.383712
Q9NZT1	Calmodulin-like protein 5 OS=Homo sapiens OX=9606 GN=CALML5 PE=1 SV=2	CALML5	1	0.99893
Q9NZT2	Opioid growth factor receptor OS=Homo sapiens OX=9606 GN=OGFR PE=1 SV=3	OGFR	1.063	
Q9NZV1	Cysteine-rich motor neuron 1 protein OS=Homo sapiens OX=9606 GN=CRIM1 PE=1	CRIM1	1.027	0.679716
Q9NZW5	Protein PALS2 OS=Homo sapiens OX=9606 GN=PALS2 PE=1	PALS2	0.835	0.279597
Q9P000	COMM domain-containing protein 9 OS=Homo sapiens OX=9606 GN=COMMD9 PE=1 SV=2	COMMD9	1.01	0.917814

Q9P013	Spliceosome-associated protein CWC15 homolog OS=Homo sapiens OX=9606 GN=CWC15	CWC15	0.958	0.799813
Q9P015	39S ribosomal protein L15, mitochondrial OS=Homo sapiens OX=9606 GN=MRPL15 PE=1 SV=1	MRPL15	1.08	0.603011
Q9P016	Thymocyte nuclear protein 1 OS=Homo sapiens OX=9606 GN=THYN1 PE=1 SV=1	THYN1	0.699	0.072089
Q9P032	NADH dehydrogenase [ubiquinone] 1 alpha subcomplex assembly factor 4 OS=Homo sapiens OX=9606 GN=NDUFAF4	NDUFAF4	1.056	0.578054
Q9P035	Very-long-chain (3R)-3-hydroxyacyl-CoA dehydratase 3 OS=Homo sapiens OX=9606 GN=HACD3 PE=1 SV=2	HACD3	1.06	0.633348
Q9P086	Mediator of RNA polymerase II transcription subunit 11 OS=Homo sapiens OX=9606 GN=MED11 PE=1 SV=2	MED11	0.881	
Q9P0I2	ER membrane protein complex subunit 3 OS=Homo sapiens OX=9606 GN=EMC3 PE=1 SV=3	EMC3	1.053	0.22115
Q9P0J0	NADH dehydrogenase [ubiquinone] 1 alpha subcomplex subunit 13 OS=Homo sapiens OX=9606 GN=NDUFA13 PE=1	NDUFA13	1.075	0.383172
Q9P0J1	[Pyruvate dehydrogenase [acetyl-transferring]]-phosphatase 1, mitochondrial OS=Homo sapiens OX=9606 GN=PDP1 PE=1 SV=3	PDP1	0.945	0.662627
Q9P0K7	Ankycorbin OS=Homo sapiens OX=9606 GN=RAI14 PE=1 SV=2	RAI14	1.073	0.375976
Q9P0L0	Vesicle-associated membrane protein-associated protein A OS=Homo sapiens OX=9606 GN=VAPA PE=1 SV=3	VAPA	0.937	0.451751
Q9P0M9	39S ribosomal protein L27, mitochondrial OS=Homo sapiens OX=9606 GN=MRPL27 PE=1 SV=1	MRPL27	0.907	0.446708
Q9P0S9	Transmembrane protein 14C OS=Homo sapiens OX=9606 GN=TMEM14C PE=1 SV=1	TMEM14C	1.259	0.023176
Q9P0T7	Proton-transporting V-type ATPase complex assembly regulator TMEM9 OS=Homo sapiens OX=9606 GN=TMEM9	TMEM9	0.816	0.09182

Q9P0U1	Mitochondrial import receptor subunit TOM7 homolog OS=Homo sapiens OX=9606 GN=TOMM7 PE=1 SV=1	TOMM7	1.084	0.60591
Q9P0V9	Septin-10 OS=Homo sapiens OX=9606 GN=SEPTIN10 PE=1 SV=2	SEPTIN10	0.795	0.099939
Q9P1F3	Costars family protein ABRACL OS=Homo sapiens OX=9606 GN=ABRACL PE=1 SV=1	ABRACL	1.19	0.458186
Q9P1Y6	PHD and RING finger domain-containing protein 1 OS=Homo sapiens OX=9606 GN=PHRF1 PE=1 SV=3	PHRF1	1.533	
Q9P206	Uncharacterized protein KIAA1522 OS=Homo sapiens OX=9606 GN=KIAA1522 PE=1	KIAA1522	0.919	0.327499
Q9P258	Protein RCC2 OS=Homo sapiens OX=9606 GN=RCC2 PE=1 SV=2	RCC2	0.801	0.10597
Q9P265	Disco-interacting protein 2 homolog B OS=Homo sapiens OX=9606 GN=DIP2B PE=1 SV=3	DIP2B	1.075	0.228338
Q9P287	BRCA2 and CDKN1A-interacting protein OS=Homo sapiens OX=9606 GN=BCCIP PE=1	BCCIP	0.871	0.196174
Q9P289	Serine/threonine-protein kinase 26 OS=Homo sapiens OX=9606 GN=STK26 PE=1 SV=2	STK26	1.05	0.7277
Q9P2B2	Prostaglandin F2 receptor negative regulator OS=Homo sapiens OX=9606 GN=PTGFRN PE=1 SV=2	PTGFRN	0.923	0.169018
Q9P2D3	HEAT repeat-containing protein 5B OS=Homo sapiens OX=9606 GN=HEATR5B PE=1 SV=2	HEATR5B	1000	0.001
Q9P2E9	Ribosome-binding protein 1 OS=Homo sapiens OX=9606 GN=RRBP1 PE=1 SV=5	RRBP1	0.971	0.693244
Q9P2I0	Cleavage and polyadenylation specificity factor subunit 2 OS=Homo sapiens OX=9606 GN=CPSF2 PE=1 SV=2	CPSF2	0.942	0.420223
Q9P2J5	Leucine--tRNA ligase, cytoplasmic OS=Homo sapiens OX=9606 GN=LARS1 PE=1 SV=2	LARS1	0.983	0.854773
Q9P2M7	Cingulin OS=Homo sapiens OX=9606 GN=CGN PE=1 SV=2	CGN	0.707	0.012319
Q9P2N5	RNA-binding protein 27 OS=Homo sapiens OX=9606 GN=RBM27 PE=1 SV=2	RBM27	0.803	0.001694

Q9P2R7	Succinate--CoA ligase [ADP-forming] subunit beta, mitochondrial OS=Homo sapiens OX=9606 GN=SUCLA2 PE=1	SUCLA2	0.99	0.786979
Q9P2W9	Syntaxin-18 OS=Homo sapiens OX=9606 GN=STX18 PE=1	STX18	1.071	0.61995
Q9P2X0	Dolichol-phosphate mannosyltransferase subunit 3 OS=Homo sapiens OX=9606 GN=DPM3 PE=1 SV=2	DPM3	1.099	0.388893
Q9UBB4	Ataxin-10 OS=Homo sapiens OX=9606 GN=ATXN10 PE=1 SV=1	ATXN10	1	0.948307
Q9UBB5	Methyl-CpG-binding domain protein 2 OS=Homo sapiens OX=9606 GN=MBD2 PE=1 SV=1	MBD2	0.944	0.521334
Q9UBB9	Tuftelin-interacting protein 11 OS=Homo sapiens OX=9606 GN=TFIP11 PE=1 SV=1	TFIP11	0.82	0.027925
Q9UBD5	Origin recognition complex subunit 3 OS=Homo sapiens OX=9606 GN=ORC3 PE=1 SV=1	ORC3	0.879	0.277975
Q9UBE0	SUMO-activating enzyme subunit 1 OS=Homo sapiens OX=9606 GN=SAE1 PE=1 SV=1	SAE1	0.907	0.53583
Q9UBF2	Coatomer subunit gamma-2 OS=Homo sapiens OX=9606 GN=COPG2 PE=1 SV=1	COPG2	0.966	0.769745
Q9UBI6	Guanine nucleotide-binding protein G(I)/G(S)/G(O) subunit gamma-12 OS=Homo sapiens OX=9606 GN=GNG12 PE=1	GNG12	0.85	0.061705
Q9UBL3	Set1/Ash2 histone methyltransferase complex subunit ASH2 OS=Homo sapiens OX=9606 GN=ASH2L PE=1	ASH2L	0.904	0.212172
Q9UBM7	7-dehydrocholesterol reductase OS=Homo sapiens OX=9606 GN=DHCR7 PE=1 SV=1	DHCR7	1.028	0.809785
Q9UBN6	Tumor necrosis factor receptor superfamily member 10D OS=Homo sapiens OX=9606 GN=TNFRSF10D PE=1 SV=1	TNFRSF10D	1.058	0.41035
Q9UBQ0	Vacuolar protein sorting-associated protein 29 OS=Homo sapiens OX=9606 GN=VPS29 PE=1 SV=1	VPS29	0.999	0.948534
Q9UBQ5	Eukaryotic translation initiation factor 3 subunit K OS=Homo sapiens OX=9606 GN=EIF3K PE=1 SV=1	EIF3K	1.031	0.779199

Q9UBQ7	Glyoxylate reductase/hydroxypyruvate reductase OS=Homo sapiens OX=9606 GN=GRHPR PE=1	GRHPR	0.899	0.346506
Q9UBR2	Cathepsin Z OS=Homo sapiens OX=9606 GN=CTSZ PE=1 SV=1	CTSZ	0.991	0.927578
Q9UBS4	DnaJ homolog subfamily B member 11 OS=Homo sapiens OX=9606 GN=DNAJB11 PE=1	DNAJB11	0.991	0.933221
Q9UBT2	SUMO-activating enzyme subunit 2 OS=Homo sapiens OX=9606 GN=UBA2 PE=1 SV=2	UBA2	0.905	0.344106
Q9UBU8	Mortality factor 4-like protein 1 OS=Homo sapiens OX=9606 GN=MORF4L1 PE=1 SV=2	MORF4L1	0.822	0.333603
Q9UBU9	Nuclear RNA export factor 1 OS=Homo sapiens OX=9606 GN=NXF1 PE=1 SV=1	NXF1	0.947	0.357418
Q9UBV2	Protein sel-1 homolog 1 OS=Homo sapiens OX=9606 GN=SEL1L PE=1 SV=3	SEL1L	1.1	0.084087
Q9UBW7	Zinc finger MYM-type protein 2 OS=Homo sapiens OX=9606 GN=ZMYM2 PE=1 SV=1	ZMYM2	0.965	0.498431
Q9UBW8	COP9 signalosome complex subunit 7a OS=Homo sapiens OX=9606 GN=COPS7A PE=1	COPS7A	0.873	0.136455
Q9UBX3	Mitochondrial dicarboxylate carrier OS=Homo sapiens OX=9606 GN=SLC25A10 PE=1	SLC25A10	0.988	0.80029
Q9UDW1	Cytochrome b-c1 complex subunit 9 OS=Homo sapiens OX=9606 GN=UQCR10 PE=1 SV=3	UQCR10	0.897	0.057001
Q9UDX5	Mitochondrial fission process protein 1 OS=Homo sapiens OX=9606 GN=MTFP1 PE=1	MTFP1	1.086	0.330501
Q9UDY2	Tight junction protein ZO-2 OS=Homo sapiens OX=9606 GN=TJP2 PE=1 SV=2	TJP2	1.124	0.103427
Q9UEU0	Vesicle transport through interaction with t-SNAREs homolog 1B OS=Homo sapiens OX=9606 GN=VTI1B PE=1 SV=3	VTI1B	0.991	0.917429
Q9UEW8	STE20/SPS1-related proline-alanine-rich protein kinase OS=Homo sapiens OX=9606 GN=STK39 PE=1 SV=3	STK39	0.928	
Q9UEY8	Gamma-adducin OS=Homo sapiens OX=9606 GN=ADD3	ADD3	0.94	0.479798
Q9UFC0	Leucine-rich repeat and WD repeat-containing protein 1 OS=Homo sapiens OX=9606 GN=LRWD1 PE=1 SV=2	LRWD1	0.868	0.14072

Q9UFG5	UPF0449 protein C19orf25 OS=Homo sapiens OX=9606 GN=C19orf25 PE=1 SV=2	C19orf25	1.036	0.976233
Q9UFN0	Protein NipSnap homolog 3A OS=Homo sapiens OX=9606 GN=NIPSNAP3A PE=1 SV=2	NIPSNAP3A	1.139	0.349389
Q9UG63	ATP-binding cassette sub-family F member 2 OS=Homo sapiens OX=9606 GN=ABCF2 PE=1	ABCF2	0.919	0.43588
Q9UGI8	Testin OS=Homo sapiens OX=9606 GN=TES PE=1 SV=1	TES	1.003	0.932156
Q9UGM6	Tryptophan--tRNA ligase, mitochondrial OS=Homo sapiens OX=9606 GN=WARS2 PE=1	WARS2	1.061	0.651612
Q9UGP8	Translocation protein SEC63 homolog OS=Homo sapiens OX=9606 GN=SEC63 PE=1 SV=2	SEC63	1.014	0.867848
Q9UH17	DNA dC->dU-editing enzyme APOBEC-3B OS=Homo sapiens OX=9606 GN=APOBEC3B PE=1 SV=1	APOBEC3B	0.891	0.275958
Q9UH99	SUN domain-containing protein 2 OS=Homo sapiens OX=9606 GN=SUN2 PE=1 SV=3	SUN2	0.914	0.514706
Q9UHA4	Ragulator complex protein LAMTOR3 OS=Homo sapiens OX=9606 GN=LAMTOR3 PE=1 SV=1	LAMTOR3	1.255	0.088503
Q9UHB6	LIM domain and actin-binding protein 1 OS=Homo sapiens OX=9606 GN=LIMA1 PE=1	LIMA1	1.007	0.948298
Q9UHB9	Signal recognition particle subunit SRP68 OS=Homo sapiens OX=9606 GN=SRP68 PE=1 SV=2	SRP68	1.024	0.815058
Q9UHD1	Cysteine and histidine-rich domain-containing protein 1 OS=Homo sapiens OX=9606 GN=CHORDC1 PE=1 SV=2	CHORDC1	1.146	0.039433
Q9UHD8	Septin-9 OS=Homo sapiens OX=9606 GN=SEPTIN9 PE=1 SV=2	SEPTIN9	1.039	0.519295
Q9UHG3	Prenylcysteine oxidase 1 OS=Homo sapiens OX=9606 GN=PCYOX1 PE=1 SV=3	PCYOX1	1.075	0.29739
Q9UHN6	Cell surface hyaluronidase OS=Homo sapiens OX=9606 GN=CEMIP2 PE=1 SV=1	CEMIP2	1.003	0.897964
Q9UHQ4	B-cell receptor-associated protein 29 OS=Homo sapiens OX=9606 GN=BCAP29 PE=1 SV=2	BCAP29	1.029	0.339058

Q9UHR4	Brain-specific angiogenesis inhibitor 1-associated protein 2-like protein 1 OS=Homo sapiens OX=9606 GN=BAIAP2L1 PE=1	BAIAP2L1	0.983	0.789687
Q9UHR5	SAP30-binding protein OS=Homo sapiens OX=9606 GN=SAP30BP PE=1 SV=1	SAP30BP	0.925	0.049439
Q9UHV9	Prefoldin subunit 2 OS=Homo sapiens OX=9606 GN=PFDN2 PE=1 SV=1	PFDN2	0.776	0.230034
Q9UHX1	Poly(U)-binding-splicing factor PUF60 OS=Homo sapiens OX=9606 GN=PUF60 PE=1 SV=1	PUF60	0.931	0.124571
Q9UHY1	Nuclear receptor-binding protein OS=Homo sapiens OX=9606 GN=NRBP1 PE=1 SV=1	NRBP1	1.14	0.172886
Q9UHY7	Enolase-phosphatase E1 OS=Homo sapiens OX=9606 GN=ENOPH1 PE=1 SV=1	ENOPH1	1.072	0.630569
Q9UI09	NADH dehydrogenase [ubiquinone] 1 alpha subcomplex subunit 12 OS=Homo sapiens OX=9606 GN=NDUFA12 PE=1	NDUFA12	1.146	0.249126
Q9UI10	Translation initiation factor eIF-2B subunit delta OS=Homo sapiens OX=9606 GN=EIF2B4 PE=1	EIF2B4	1.136	0.396266
Q9UI12	V-type proton ATPase subunit H OS=Homo sapiens OX=9606 GN=ATP6V1H PE=1 SV=1	ATP6V1H	1.066	0.140138
Q9UI14	Prenylated Rab acceptor protein 1 OS=Homo sapiens OX=9606 GN=RABAC1 PE=1 SV=1	RABAC1	0.885	0.175057
Q9UI30	Multifunctional methyltransferase subunit TRM112-like protein OS=Homo sapiens OX=9606 GN=TRMT112 PE=1 SV=1	TRMT112	0.923	0.445154
Q9UIA9	Exportin-7 OS=Homo sapiens OX=9606 GN=XPO7 PE=1 SV=3	XPO7	1.194	0.135138
Q9UIF9	Bromodomain adjacent to zinc finger domain protein 2A OS=Homo sapiens OX=9606 GN=BAZ2A PE=1 SV=4	BAZ2A	0.893	0.068041
Q9UIG0	Tyrosine-protein kinase BAZ1B OS=Homo sapiens OX=9606 GN=BAZ1B PE=1 SV=2	BAZ1B	0.977	0.802705
Q9UII2	ATPase inhibitor, mitochondrial OS=Homo sapiens OX=9606 GN=ATP5IF1 PE=1 SV=1	ATP5IF1	1.113	0.67687
Q9UIJ7	GTP:AMP phosphotransferase AK3, mitochondrial OS=Homo sapiens OX=9606 GN=AK3 PE=1	AK3	1.058	0.301574

Q9UIL1	Short coiled-coil protein OS=Homo sapiens OX=9606 GN=SCOC PE=1 SV=2	SCOC	0.793	0.32862
Q9UIQ6	Leucyl-cystinyl aminopeptidase OS=Homo sapiens OX=9606 GN=LNPEP PE=1 SV=3	LNPEP	1.046	0.469793
Q9UIS9	Methyl-CpG-binding domain protein 1 OS=Homo sapiens OX=9606 GN=MBD1 PE=1 SV=2	MBD1	0.971	0.828622
Q9UIV1	CCR4-NOT transcription complex subunit 7 OS=Homo sapiens OX=9606 GN=CNOT7 PE=1	CNOT7	1.127	
Q9UIW2	Plexin-A1 OS=Homo sapiens OX=9606 GN=PLXNA1 PE=1 SV=3	PLXNA1	0.975	0.753549
Q9UJ72	Annexin A10 OS=Homo sapiens OX=9606 GN=ANXA10 PE=1 SV=3	ANXA10	1.097	0.201766
Q9UJ83	2-hydroxyacyl-CoA lyase 1 OS=Homo sapiens OX=9606 GN=HACL1 PE=1 SV=2	HACL1	1.057	0.495847
Q9UJA5	tRNA (adenine(58)-N(1))-methyltransferase non-catalytic subunit TRM6 OS=Homo sapiens OX=9606 GN=TRMT6 PE=1	TRMT6	0.959	0.740714
Q9UJS0	Calcium-binding mitochondrial carrier protein Aralar2 OS=Homo sapiens OX=9606 GN=SLC25A13 PE=1 SV=2	SLC25A13	1.017	0.721717
Q9UJU6	Drebrin-like protein OS=Homo sapiens OX=9606 GN=DBNL PE=1 SV=1	DBNL	0.956	0.516619
Q9UJV9	Probable ATP-dependent RNA helicase DDX41 OS=Homo sapiens OX=9606 GN=DDX41	DDX41	1.115	0.248572
Q9UJX3	Anaphase-promoting complex subunit 7 OS=Homo sapiens OX=9606 GN=ANAPC7 PE=1 SV=4	ANAPC7	1.142	0.036326
Q9UJX5	Anaphase-promoting complex subunit 4 OS=Homo sapiens OX=9606 GN=ANAPC4 PE=1 SV=2	ANAPC4	1.077	
Q9UJZ1	Stomatin-like protein 2, mitochondrial OS=Homo sapiens OX=9606 GN=STOML2 PE=1 SV=1	STOML2	0.971	0.535964
Q9UK45	U6 snRNA-associated Sm-like protein LSm7 OS=Homo sapiens OX=9606 GN=LSM7 PE=1 SV=1	LSM7	0.856	0.071507
Q9UK58	Cyclin-L1 OS=Homo sapiens OX=9606 GN=CCNL1 PE=1	CCNL1	0.825	0.089664

Q9UK59	Lariat debranching enzyme OS=Homo sapiens OX=9606 GN=DBR1 PE=1 SV=2	DBR1	0.883	0.522277
Q9UK61	Protein TASOR OS=Homo sapiens OX=9606 GN=TASOR	TASOR	0.885	0.405236
Q9UK76	Jupiter microtubule associated homolog 1 OS=Homo sapiens OX=9606 GN=JPT1 PE=1 SV=3	JPT1	0.794	0.204358
Q9UKA9	Polypyrimidine tract-binding protein 2 OS=Homo sapiens OX=9606 GN=PTBP2 PE=1	PTBP2	0.812	0.024513
Q9UKD2	mRNA turnover protein 4 homolog OS=Homo sapiens OX=9606 GN=MRTO4 PE=1 SV=2	MRTO4	0.986	0.848656
Q9UKF6	Cleavage and polyadenylation specificity factor subunit 3 OS=Homo sapiens OX=9606 GN=CPSF3 PE=1 SV=1	CPSF3	0.918	0.255046
Q9UKG9	Peroxisomal carnitine O-octanoyltransferase OS=Homo sapiens OX=9606 GN=CROT PE=1 SV=2	CROT	0.91	0.182585
Q9UKJ3	G patch domain-containing protein 8 OS=Homo sapiens OX=9606 GN=GPATCH8 PE=1 SV=2	GPATCH8	1.134	0.540339
Q9UKK9	ADP-sugar pyrophosphatase OS=Homo sapiens OX=9606 GN=NUDT5 PE=1 SV=1	NUDT5	1.013	0.9091
Q9UKL0	REST corepressor 1 OS=Homo sapiens OX=9606 GN=RCOR1 PE=1 SV=2	RCOR1	0.885	0.004023
Q9UKM7	Endoplasmic reticulum mannosyl-oligosaccharide 1,2-alpha-mannosidase OS=Homo sapiens OX=9606 GN=MAN1B1 PE=1 SV=2	MAN1B1	0.938	0.377589
Q9UKM9	RNA-binding protein Raly OS=Homo sapiens OX=9606 GN=RALY PE=1 SV=1	RALY	0.948	0.528049
Q9UKN8	General transcription factor 3C polypeptide 4 OS=Homo sapiens OX=9606 GN=GTF3C4 PE=1	GTF3C4	0.87	0.015183
Q9UKR5	Ergosterol biosynthetic protein 28 homolog OS=Homo sapiens OX=9606 GN=ERG28 PE=1	ERG28	0.995	
Q9UKS6	Protein kinase C and casein kinase substrate in neurons protein 3 OS=Homo sapiens OX=9606 GN=PACSIN3 PE=1 SV=2	PACSIN3	0.907	0.028144
Q9UKV3	Apoptotic chromatin condensation inducer in the nucleus OS=Homo sapiens OX=9606 GN=ACIN1 PE=1 SV=2	ACIN1	0.894	0.076466

Q9UKV5	E3 ubiquitin-protein ligase AMFR OS=Homo sapiens OX=9606 GN=AMFR PE=1 SV=2	AMFR	1.013	0.877304
Q9UKV8	Protein argonaute-2 OS=Homo sapiens OX=9606 GN=AGO2 PE=1 SV=3	AGO2	0.962	0.891748
Q9UKX7	Nuclear pore complex protein Nup50 OS=Homo sapiens OX=9606 GN=NUP50 PE=1	NUP50	0.937	0.261862
Q9UKY7	Protein CDV3 homolog OS=Homo sapiens OX=9606 GN=CDV3 PE=1 SV=1	CDV3	1.18	0.16508
Q9UKZ1	CCR4-NOT transcription complex subunit 11 OS=Homo sapiens OX=9606 GN=CNOT11 PE=1 SV=1	CNOT11	1.078	0.450691
Q9UL25	Ras-related protein Rab-21 OS=Homo sapiens OX=9606 GN=RAB21 PE=1 SV=3	RAB21	0.909	0.089886
Q9UL26	Ras-related protein Rab-22A OS=Homo sapiens OX=9606 GN=RAB22A PE=1 SV=2	RAB22A	0.914	0.384164
Q9UL40	Zinc finger protein 346 OS=Homo sapiens OX=9606 GN=ZNF346 PE=1 SV=1	ZNF346	1.024	0.848297
Q9UL46	Proteasome activator complex subunit 2 OS=Homo sapiens OX=9606 GN=PSME2 PE=1	PSME2	1.071	0.509112
Q9ULA0	Aspartyl aminopeptidase OS=Homo sapiens OX=9606 GN=DNPEP PE=1 SV=2	DNPEP	1.033	0.852405
Q9ULC3	Ras-related protein Rab-23 OS=Homo sapiens OX=9606 GN=RAB23 PE=1 SV=1	RAB23	1.011	0.881325
Q9ULC4	Malignant T-cell-amplified sequence 1 OS=Homo sapiens OX=9606 GN=MCTS1 PE=1	MCTS1	1.149	0.158189
Q9ULC5	Long-chain-fatty-acid--CoA ligase 5 OS=Homo sapiens OX=9606 GN=ACSL5 PE=1 SV=1	ACSL5	0.95	0.398787
Q9ULF5	Zinc transporter ZIP10 OS=Homo sapiens OX=9606 GN=SLC39A10 PE=1 SV=2	SLC39A10	0.948	0.7372
Q9ULG6	Cell cycle progression protein 1 OS=Homo sapiens OX=9606 GN=CCPG1 PE=1 SV=3	CCPG1	1.13	0.441555
Q9ULH0	Kinase D-interacting substrate of 220 kDa OS=Homo sapiens OX=9606 GN=KIDINS220 PE=1 SV=3	KIDINS220	0.957	0.683588

Q9ULK4	Mediator of RNA polymerase II transcription subunit 23 OS=Homo sapiens OX=9606 GN=MED23 PE=1 SV=2	MED23	1.003	0.987412
Q9ULR0	Pre-mRNA-splicing factor ISY1 homolog OS=Homo sapiens OX=9606 GN=ISY1 PE=1 SV=3	ISY1	0.887	0.211739
Q9ULT8	E3 ubiquitin-protein ligase HECTD1 OS=Homo sapiens OX=9606 GN=HECTD1 PE=1	HECTD1	2.021	
Q9ULU4	Protein kinase C-binding protein 1 OS=Homo sapiens OX=9606 GN=ZMYND8 PE=1 SV=2	ZMYND8	0.875	0.039504
Q9ULV4	Coronin-1C OS=Homo sapiens OX=9606 GN=CORO1C PE=1 SV=1	CORO1C	1.038	0.477832
Q9ULW0	Targeting protein for Xklp2 OS=Homo sapiens OX=9606 GN=TPX2 PE=1 SV=2	TPX2	0.987	0.820642
Q9ULW3	Activator of basal transcription 1 OS=Homo sapiens OX=9606 GN=ABT1 PE=1 SV=1	ABT1	1.361	0.247618
Q9ULX6	A-kinase anchor protein 8-like OS=Homo sapiens OX=9606 GN=AKAP8L PE=1 SV=4	AKAP8L	1.006	0.924274
Q9ULX9	Transcription factor MafF OS=Homo sapiens OX=9606 GN=MAFF PE=1 SV=2	MAFF	1.139	0.1657
Q9UM00	Calcium load-activated calcium channel OS=Homo sapiens OX=9606 GN=TMCO1 PE=1	TMCO1	0.905	0.308514
Q9UMN6	Histone-lysine N-methyltransferase 2B OS=Homo sapiens OX=9606 GN=KMT2B PE=1 SV=1	KMT2B	1000	0.001
Q9UMS0	NFU1 iron-sulfur cluster scaffold homolog, mitochondrial OS=Homo sapiens OX=9606 GN=NFU1 PE=1 SV=2	NFU1	0.928	0.002108
Q9UMS4	Pre-mRNA-processing factor 19 OS=Homo sapiens OX=9606 GN=PRPF19 PE=1 SV=1	PRPF19	0.94	0.510615
Q9UMX0	Ubiquilin-1 OS=Homo sapiens OX=9606 GN=UBQLN1 PE=1 SV=2	UBQLN1	0.9	0.108426
Q9UMX5	Neudesin OS=Homo sapiens OX=9606 GN=NENF PE=1 SV=1	NENF	1.113	0.075324
Q9UMY1	Nucleolar protein 7 OS=Homo sapiens OX=9606 GN=NOL7 PE=1 SV=2	NOL7	0.806	0.116362
Q9UMY4	Sorting nexin-12 OS=Homo sapiens OX=9606 GN=SNX12	SNX12	1.079	0.504605

Q9UN70	Protocadherin gamma-C3 OS=Homo sapiens OX=9606 GN=PCDHGC3 PE=1 SV=1	PCDHGC3	0.958	0.409768
Q9UN81	LINE-1 retrotransposable element ORF1 protein OS=Homo sapiens OX=9606 GN=L1RE1 PE=1	L1RE1	1.102	0.244618
Q9UN86	Ras GTPase-activating protein-binding protein 2 OS=Homo sapiens OX=9606 GN=G3BP2	G3BP2	0.952	0.530381
Q9UNE7	E3 ubiquitin-protein ligase CHIP OS=Homo sapiens OX=9606 GN=STUB1 PE=1 SV=2	STUB1	0.99	0.849041
Q9UNF0	Protein kinase C and casein kinase substrate in neurons protein 2 OS=Homo sapiens OX=9606 GN=PACSIN2 PE=1 SV=2	PACSIN2	1.131	0.184951
Q9UNF1	Melanoma-associated antigen D2 OS=Homo sapiens OX=9606 GN=MAGED2 PE=1 SV=2	MAGED2	1.15	0.252132
Q9UNH7	Sorting nexin-6 OS=Homo sapiens OX=9606 GN=SNX6 PE=1 SV=1	SNX6	1.032	0.730506
Q9UNK0	Syntaxin-8 OS=Homo sapiens OX=9606 GN=STX8 PE=1 SV=2	STX8	0.99	0.900781
Q9UNL2	Translocon-associated protein subunit gamma OS=Homo sapiens OX=9606 GN=SSR3 PE=1 SV=1	SSR3	1.067	0.23301
Q9UNM6	26S proteasome non-ATPase regulatory subunit 13 OS=Homo sapiens OX=9606 GN=PSMD13 PE=1 SV=2	PSMD13	1.002	0.987405
Q9UNN8	Endothelial protein C receptor OS=Homo sapiens OX=9606 GN=PROCR PE=1 SV=1	PROCR	0.573	0.002212
Q9UNP9	Peptidyl-prolyl cis-trans isomerase E OS=Homo sapiens OX=9606 GN=PPIE PE=1 SV=1	PPIE	0.92	0.421126
Q9UNQ2	Probable dimethyladenosine transferase OS=Homo sapiens OX=9606 GN=DIMT1 PE=1	DIMT1	1.035	0.659461
Q9UNS2	COP9 signalosome complex subunit 3 OS=Homo sapiens OX=9606 GN=COPS3 PE=1	COPS3	1.093	0.346587
Q9UNW1	Multiple inositol polyphosphate phosphatase 1 OS=Homo sapiens OX=9606 GN=MINPP1 PE=1 SV=1	MINPP1	0.998	0.992016
Q9UNX4	WD repeat-containing protein 3 OS=Homo sapiens OX=9606 GN=WDR3 PE=1 SV=1	WDR3	0.927	0.373553
Q9UNZ2	NSFL1 cofactor p47 OS=Homo sapiens OX=9606 GN=NSFL1C PE=1 SV=2	NSFL1C	0.916	0.246926

Q9UP83	Conserved oligomeric Golgi complex subunit 5 OS=Homo sapiens OX=9606 GN=COG5 PE=1 SV=3	COG5	0.878	0.101276
Q9UP95	Solute carrier family 12 member 4 OS=Homo sapiens OX=9606 GN=SLC12A4 PE=1 SV=2	SLC12A4	1.158	0.257602
Q9UPN3	Microtubule-actin cross-linking factor 1, isoforms 1/2/3/5 OS=Homo sapiens OX=9606 GN=MACF1 PE=1 SV=4	MACF1	1.164	0.052883
Q9UPT8	Zinc finger CCCH domain-containing protein 4 OS=Homo sapiens OX=9606 GN=ZC3H4 PE=1 SV=3	ZC3H4	0.977	0.697423
Q9UPY5	Cystine/glutamate transporter OS=Homo sapiens OX=9606 GN=SLC7A11 PE=1 SV=1	SLC7A11	1.096	0.659829
Q9UPY8	Microtubule-associated protein RP/EB family member 3 OS=Homo sapiens OX=9606 GN=MAPRE3 PE=1 SV=1	MAPRE3	1.106	0.668902
Q9UQ35	Serine/arginine repetitive matrix protein 2 OS=Homo sapiens OX=9606 GN=SRRM2 PE=1	SRRM2	0.933	0.32127
Q9UQ80	Proliferation-associated protein 2G4 OS=Homo sapiens OX=9606 GN=PA2G4 PE=1 SV=3	PA2G4	0.948	0.515195
Q9UQB8	Brain-specific angiogenesis inhibitor 1-associated protein 2 OS=Homo sapiens OX=9606 GN=BAIAP2 PE=1 SV=1	BAIAP2	1.05	0.143637
Q9UQE7	Structural maintenance of chromosomes protein 3 OS=Homo sapiens OX=9606 GN=SMC3 PE=1 SV=2	SMC3	0.953	0.197425
Q9UQR1	Zinc finger protein 148 OS=Homo sapiens OX=9606 GN=ZNF148 PE=1 SV=2	ZNF148	0.791	0.034164
Q9Y221	60S ribosome subunit biogenesis protein NIP7 homolog OS=Homo sapiens OX=9606 GN=NIP7 PE=1 SV=1	NIP7	0.973	0.707831
Q9Y224	RNA transcription, translation and transport factor protein OS=Homo sapiens OX=9606 GN=RTRAF PE=1 SV=1	RTRAF	0.987	0.806334
Q9Y230	RuvB-like 2 OS=Homo sapiens OX=9606 GN=RUVBL2 PE=1 SV=3	RUVBL2	0.987	0.571374

Q9Y241	HIG1 domain family member 1A, mitochondrial OS=Homo sapiens OX=9606 GN=HIGD1A PE=1 SV=1	HIGD1A	1.007	0.993546
Q9Y262	Eukaryotic translation initiation factor 3 subunit L OS=Homo sapiens OX=9606 GN=EIF3L PE=1 SV=1	EIF3L	0.994	0.913047
Q9Y265	RuvB-like 1 OS=Homo sapiens OX=9606 GN=RUVBL1 PE=1 SV=1	RUVBL1	0.93	0.129862
Q9Y266	Nuclear migration protein nudC OS=Homo sapiens OX=9606 GN=NUDC PE=1 SV=1	NUDC	1.125	0.218558
Q9Y276	Mitochondrial chaperone BCS1 OS=Homo sapiens OX=9606 GN=BCS1L PE=1 SV=1	BCS1L	1.033	0.734616
Q9Y277	Voltage-dependent anion-selective channel protein 3 OS=Homo sapiens OX=9606 GN=VDAC3	VDAC3	1.035	0.364682
Q9Y281	Cofilin-2 OS=Homo sapiens OX=9606 GN=CFL2 PE=1 SV=1	CFL2	1.198	0.196062
Q9Y282	Endoplasmic reticulum-Golgi intermediate compartment protein 3 OS=Homo sapiens OX=9606 GN=ERGIC3 PE=1 SV=1	ERGIC3	1.022	0.647329
Q9Y285	Phenylalanine--tRNA ligase alpha subunit OS=Homo sapiens OX=9606 GN=FARSA PE=1	FARSA	1.037	0.660678
Q9Y287	Integral membrane protein 2B OS=Homo sapiens OX=9606 GN=ITM2B PE=1 SV=1	ITM2B	1.054	0.467453
Q9Y289	Sodium-dependent multivitamin transporter OS=Homo sapiens OX=9606 GN=SLC5A6 PE=1	SLC5A6	1.243	
Q9Y295	Developmentally-regulated GTP-binding protein 1 OS=Homo sapiens OX=9606 GN=DRG1	DRG1	0.975	0.732572
Q9Y2A7	Nck-associated protein 1 OS=Homo sapiens OX=9606 GN=NCKAP1 PE=1 SV=1	NCKAP1	1.129	0.291272
Q9Y2B0	Protein canopy homolog 2 OS=Homo sapiens OX=9606 GN=CNPY2 PE=1 SV=1	CNPY2	1.008	0.873041
Q9Y2D5	A-kinase anchor protein 2 OS=Homo sapiens OX=9606 GN=AKAP2 PE=1 SV=3	AKAP2	1.009	0.913381
Q9Y2G5	GDP-fucose protein O-fucosyltransferase 2 OS=Homo sapiens OX=9606 GN=POFUT2 PE=1 SV=3	POFUT2	1.001	

Q9Y2H6	Fibronectin type-III domain-containing protein 3A OS=Homo sapiens OX=9606 GN=FNDC3A PE=1 SV=4	FNDC3A	1.004	0.959105
Q9Y2K7	Lysine-specific demethylase 2A OS=Homo sapiens OX=9606 GN=KDM2A PE=1 SV=3	KDM2A	0.964	0.228418
Q9Y2L1	Exosome complex exonuclease RRP44 OS=Homo sapiens OX=9606 GN=DIS3 PE=1 SV=2	DIS3	0.839	0.201895
Q9Y2P8	RNA 3'-terminal phosphate cyclase-like protein OS=Homo sapiens OX=9606 GN=RCL1	RCL1	1.1	0.458121
Q9Y2Q3	Glutathione S-transferase kappa 1 OS=Homo sapiens OX=9606 GN=GSTK1 PE=1 SV=3	GSTK1	1.022	0.788701
Q9Y2Q5	Ragulator complex protein LAMTOR2 OS=Homo sapiens OX=9606 GN=LAMTOR2 PE=1 SV=1	LAMTOR2	1.041	0.411809
Q9Y2Q9	28S ribosomal protein S28, mitochondrial OS=Homo sapiens OX=9606 GN=MRPS28 PE=1 SV=1	MRPS28	0.982	0.803256
Q9Y2R0	Cytochrome c oxidase assembly factor 3 homolog, mitochondrial OS=Homo sapiens OX=9606 GN=COA3 PE=1 SV=1	COA3	0.908	0.215791
Q9Y2R4	Probable ATP-dependent RNA helicase DDX52 OS=Homo sapiens OX=9606 GN=DDX52	DDX52	1.322	0.343871
Q9Y2R5	28S ribosomal protein S17, mitochondrial OS=Homo sapiens OX=9606 GN=MRPS17 PE=1 SV=1	MRPS17	0.992	0.875905
Q9Y2R9	28S ribosomal protein S7, mitochondrial OS=Homo sapiens OX=9606 GN=MRPS7 PE=1	MRPS7	0.966	0.373318
Q9Y2S7	Polymerase delta-interacting protein 2 OS=Homo sapiens OX=9606 GN=POLDIP2 PE=1	POLDIP2	1.004	0.916883
Q9Y2T2	AP-3 complex subunit mu-1 OS=Homo sapiens OX=9606 GN=AP3M1 PE=1 SV=1	AP3M1	1.174	
Q9Y2U8	Inner nuclear membrane protein Man1 OS=Homo sapiens OX=9606 GN=LEMD3 PE=1	LEMD3	0.951	0.548305
Q9Y2V2	Calcium-regulated heat-stable protein 1 OS=Homo sapiens OX=9606 GN=CARHSP1 PE=1 SV=2	CARHSP1	0.951	

Q9Y2W1	Thyroid hormone receptor-associated protein 3 OS=Homo sapiens OX=9606 GN=THRAP3 PE=1 SV=2	THRAP3	0.942	0.092374
Q9Y2W2	WW domain-binding protein 11 OS=Homo sapiens OX=9606 GN=WBP11 PE=1 SV=1	WBP11	0.857	0.094877
Q9Y2X0	Mediator of RNA polymerase II transcription subunit 16 OS=Homo sapiens OX=9606 GN=MED16 PE=1 SV=2	MED16	0.972	
Q9Y2X3	Nucleolar protein 58 OS=Homo sapiens OX=9606 GN=NOP58 PE=1 SV=1	NOP58	0.991	0.871208
Q9Y2Z0	Protein SGT1 homolog OS=Homo sapiens OX=9606 GN=SUGT1 PE=1 SV=3	SUGT1	1.026	0.942787
Q9Y2Z2	Protein MTO1 homolog, mitochondrial OS=Homo sapiens OX=9606 GN=MTO1 PE=1 SV=2	MTO1	0.912	0.307337
Q9Y2Z4	Tyrosine--tRNA ligase, mitochondrial OS=Homo sapiens OX=9606 GN=YARS2 PE=1	YARS2	0.901	0.175692
Q9Y2Z9	Ubiquinone biosynthesis monooxygenase COQ6, mitochondrial OS=Homo sapiens OX=9606 GN=COQ6 PE=1 SV=2	COQ6	0.94	0.669832
Q9Y305	Acyl-coenzyme A thioesterase 9, mitochondrial OS=Homo sapiens OX=9606 GN=ACOT9 PE=1	ACOT9	1.057	0.447316
Q9Y312	Protein AAR2 homolog OS=Homo sapiens OX=9606 GN=AAR2 PE=1 SV=2	AAR2	0.833	
Q9Y314	Nitric oxide synthase-interacting protein OS=Homo sapiens OX=9606 GN=NOSIP PE=1	NOSIP	0.908	0.253811
Q9Y320	Thioredoxin-related transmembrane protein 2 OS=Homo sapiens OX=9606	TMX2	0.954	0.522507
Q9Y324	rRNA-processing protein FCF1 homolog OS=Homo sapiens OX=9606 GN=FCF1 PE=1 SV=1	FCF1	1.2	0.306237
Q9Y333	U6 snRNA-associated Sm-like protein LSm2 OS=Homo sapiens OX=9606 GN=LSM2 PE=1 SV=1	LSM2	0.927	0.454163
Q9Y371	Endophilin-B1 OS=Homo sapiens OX=9606 GN=SH3GLB1 PE=1 SV=1	SH3GLB1	1.055	
Q9Y375	Complex I intermediate-associated protein 30, mitochondrial OS=Homo sapiens OX=9606 GN=NDUFAF1 PE=1 SV=2	NDUFAF1	0.915	0.355644

Q9Y376	Calcium-binding protein 39 OS=Homo sapiens OX=9606 GN=CAB39 PE=1 SV=1	CAB39	0.963	0.683268
Q9Y383	Putative RNA-binding protein Luc7-like 2 OS=Homo sapiens OX=9606 GN=LUC7L2 PE=1	LUC7L2	0.975	0.669221
Q9Y385	Ubiquitin-conjugating enzyme E2 J1 OS=Homo sapiens OX=9606 GN=UBE2J1 PE=1 SV=2	UBE2J1	1.184	0.051704
Q9Y388	RNA-binding motif protein, X-linked 2 OS=Homo sapiens OX=9606 GN=RBMX2 PE=1	RBMX2	0.797	
Q9Y394	Dehydrogenase/reductase SDR family member 7 OS=Homo sapiens OX=9606 GN=DHRS7	DHRS7	1.115	0.027476
Q9Y399	28S ribosomal protein S2, mitochondrial OS=Homo sapiens OX=9606 GN=MRPS2 PE=1	MRPS2	1.078	0.412937
Q9Y3A2	Probable U3 small nucleolar RNA-associated protein 11 OS=Homo sapiens OX=9606 GN=UTP11 PE=1 SV=2	UTP11	1.336	
Q9Y3A3	MOB-like protein phocein OS=Homo sapiens OX=9606 GN=MOB4 PE=1 SV=1	MOB4	1.089	0.450655
Q9Y3A4	Ribosomal RNA-processing protein 7 homolog A OS=Homo sapiens OX=9606 GN=RRP7A	RRP7A	0.981	0.728377
Q9Y3A5	Ribosome maturation protein SBDS OS=Homo sapiens OX=9606 GN=SBDS PE=1 SV=4	SBDS	1.045	0.471154
Q9Y3A6	Transmembrane emp24 domain-containing protein 5 OS=Homo sapiens OX=9606 GN=TMED5 PE=1 SV=1	TMED5	1.077	0.363689
Q9Y3B2	Exosome complex component CSL4 OS=Homo sapiens OX=9606 GN=EXOSC1 PE=1	EXOSC1	0.963	0.662772
Q9Y3B3	Transmembrane emp24 domain-containing protein 7 OS=Homo sapiens OX=9606 GN=TMED7 PE=1 SV=2	TMED7	1.01	0.811379
Q9Y3B4	Splicing factor 3B subunit 6 OS=Homo sapiens OX=9606 GN=SF3B6 PE=1 SV=1	SF3B6	1.02	0.855879
Q9Y3B7	39S ribosomal protein L11, mitochondrial OS=Homo sapiens OX=9606 GN=MRPL11 PE=1 SV=1	MRPL11	1	0.972882
Q9Y3B8	Oligoribonuclease, mitochondrial OS=Homo sapiens OX=9606 GN=REXO2 PE=1 SV=3	REXO2	1.213	0.184517

Q9Y3B9	RRP15-like protein OS=Homo sapiens OX=9606 GN=RRP15 PE=1 SV=2	RRP15	0.953	0.564309
Q9Y3C1	Nucleolar protein 16 OS=Homo sapiens OX=9606 GN=NOP16 PE=1 SV=2	NOP16	0.937	0.558574
Q9Y3C4	EKC/KEOPS complex subunit TPRKB OS=Homo sapiens OX=9606 GN=TPRKB PE=1	TPRKB	0.971	0.924721
Q9Y3C6	Peptidyl-prolyl cis-trans isomerase-like 1 OS=Homo sapiens OX=9606 GN=PPIL1	PPIL1	0.922	0.428217
Q9Y3D3	28S ribosomal protein S16, mitochondrial OS=Homo sapiens OX=9606 GN=MRPS16 PE=1 SV=1	MRPS16	0.924	0.176824
Q9Y3D5	28S ribosomal protein S18c, mitochondrial OS=Homo sapiens OX=9606 GN=MRPS18C PE=1 SV=1	MRPS18C	1.011	0.866162
Q9Y3D6	Mitochondrial fission 1 protein OS=Homo sapiens OX=9606 GN=FIS1 PE=1 SV=2	FIS1	0.777	0.067234
Q9Y3D7	Mitochondrial import inner membrane translocase subunit TIM16 OS=Homo sapiens OX=9606 GN=PAM16 PE=1	PAM16	1.021	0.931205
Q9Y3D9	28S ribosomal protein S23, mitochondrial OS=Homo sapiens OX=9606 GN=MRPS23 PE=1 SV=2	MRPS23	1.029	0.415338
Q9Y3E0	Vesicle transport protein GOT1B OS=Homo sapiens OX=9606 GN=GOLT1B PE=1 SV=1	GOLT1B	1.142	0.211695
Q9Y3E5	Peptidyl-tRNA hydrolase 2, mitochondrial OS=Homo sapiens OX=9606 GN=PTRH2 PE=1	PTRH2	1.003	0.927666
Q9Y3E7	Charged multivesicular body protein 3 OS=Homo sapiens OX=9606 GN=CHMP3 PE=1	CHMP3	1.446	
Q9Y3F4	Serine-threonine kinase receptor-associated protein OS=Homo sapiens OX=9606 GN=STRAP PE=1 SV=1	STRAP	0.98	0.821724
Q9Y3I0	RNA-splicing ligase RtcB homolog OS=Homo sapiens OX=9606 GN=RTCB PE=1 SV=1	RTCB	1.007	0.889411
Q9Y3L5	Ras-related protein Rap-2c OS=Homo sapiens OX=9606 GN=RAP2C PE=1 SV=1	RAP2C	0.929	0.595702

Q9Y3Q3	Transmembrane emp24 domain-containing protein 3 OS=Homo sapiens OX=9606 GN=TMED3 PE=1 SV=1	TMED3	1.135	0.139855
Q9Y3T9	Nucleolar complex protein 2 homolog OS=Homo sapiens OX=9606 GN=NOC2L PE=1	NOC2L	0.989	0.882762
Q9Y3U8	60S ribosomal protein L36 OS=Homo sapiens OX=9606 GN=RPL36 PE=1 SV=3	RPL36	1.081	0.92638
Q9Y3Y2	Chromatin target of PRMT1 protein OS=Homo sapiens OX=9606 GN=CHTOP PE=1	CHTOP	1.146	0.819377
Q9Y446	Plakophilin-3 OS=Homo sapiens OX=9606 GN=PKP3 PE=1 SV=1	PKP3	0.963	0.341185
Q9Y490	Talin-1 OS=Homo sapiens OX=9606 GN=TLN1 PE=1 SV=3	TLN1	1.019	0.667454
Q9Y4A5	Transformation/transcription domain-associated protein OS=Homo sapiens OX=9606 GN=TRRAP PE=1 SV=3	TRRAP	1.032	0.775893
Q9Y4C8	Probable RNA-binding protein 19 OS=Homo sapiens OX=9606 GN=RBM19 PE=1 SV=3	RBM19	1.048	0.602088
Q9Y4D1	Disheveled-associated activator of morphogenesis 1 OS=Homo sapiens OX=9606 GN=DAAM1	DAAM1	1.008	
Q9Y4E8	Ubiquitin carboxyl-terminal hydrolase 15 OS=Homo sapiens OX=9606 GN=USP15 PE=1 SV=3	USP15	1.038	0.715647
Q9Y4K1	Beta/gamma crystallin domain-containing protein 1 OS=Homo sapiens OX=9606 GN=CRYBG1 PE=1 SV=3	CRYBG1	0.918	0.17879
Q9Y4L1	Hypoxia up-regulated protein 1 OS=Homo sapiens OX=9606 GN=HYOU1 PE=1 SV=1	HYOU1	1.024	0.557027
Q9Y4P3	Transducin beta-like protein 2 OS=Homo sapiens OX=9606 GN=TBL2 PE=1 SV=1	TBL2	1.008	0.96664
Q9Y4W2	Ribosomal biogenesis protein LAS1L OS=Homo sapiens OX=9606 GN=LAS1L PE=1	LAS1L	0.946	0.48861
Q9Y4W6	AFG3-like protein 2 OS=Homo sapiens OX=9606 GN=AFG3L2 PE=1 SV=2	AFG3L2	0.963	0.638345
Q9Y4X5	E3 ubiquitin-protein ligase ARIH1 OS=Homo sapiens OX=9606 GN=ARIH1 PE=1 SV=2	ARIH1	1.027	0.833736
Q9Y4Z0	U6 snRNA-associated Sm-like protein LSm4 OS=Homo sapiens OX=9606 GN=LSM4 PE=1 SV=1	LSM4	0.9	0.332867

Q9Y508	E3 ubiquitin-protein ligase RNF114 OS=Homo sapiens OX=9606 GN=RNF114 PE=1	RNF114	0.937	0.554625
Q9Y512	Sorting and assembly machinery component 50 homolog OS=Homo sapiens OX=9606 GN=SAMM50 PE=1 SV=3	SAMM50	1.033	0.62121
Q9Y520	Protein PRRC2C OS=Homo sapiens OX=9606 GN=PRRC2C PE=1 SV=4	PRRC2C	0.99	0.856526
Q9Y570	Protein phosphatase methylesterase 1 OS=Homo sapiens OX=9606 GN=PPME1 PE=1 SV=3	PPME1	0.953	0.653782
Q9Y580	RNA-binding protein 7 OS=Homo sapiens OX=9606 GN=RBM7 PE=1 SV=1	RBM7	0.922	0.409215
Q9Y5A9	YTH domain-containing family protein 2 OS=Homo sapiens OX=9606 GN=YTHDF2 PE=1 SV=2	YTHDF2	1.045	0.577618
Q9Y5B0	RNA polymerase II subunit A C-terminal domain phosphatase OS=Homo sapiens OX=9606 GN=CTDP1 PE=1 SV=3	CTDP1	0.824	
Q9Y5B6	PAX3- and PAX7-binding protein 1 OS=Homo sapiens OX=9606 GN=PAXBP1 PE=1 SV=2	PAXBP1	1.045	0.507216
Q9Y5B9	FACT complex subunit SPT16 OS=Homo sapiens OX=9606 GN=SUPT16H PE=1 SV=1	SUPT16H	0.873	0.113696
Q9Y5G3	Protocadherin gamma-B1 OS=Homo sapiens OX=9606 GN=PCDHGB1 PE=1 SV=1	PCDHGB1	1.018	0.761565
Q9Y5J1	U3 small nucleolar RNA-associated protein 18 homolog OS=Homo sapiens OX=9606 GN=UTP18 PE=1 SV=3	UTP18	0.951	0.564242
Q9Y5J6	Mitochondrial import inner membrane translocase subunit Tim10 B OS=Homo sapiens OX=9606 GN=TIMM10B PE=1 SV=1	TIMM10B	1.141	0.291284
Q9Y5J7	Mitochondrial import inner membrane translocase subunit Tim9 OS=Homo sapiens OX=9606 GN=TIMM9 PE=1	TIMM9	0.977	0.707205
Q9Y5J9	Mitochondrial import inner membrane translocase subunit Tim8 B OS=Homo sapiens OX=9606 GN=TIMM8B PE=1	TIMM8B	0.89	0.335622

Q9Y5K5	Ubiquitin carboxyl-terminal hydrolase isozyme L5 OS=Homo sapiens OX=9606 GN=UCHL5 PE=1 SV=3	UCHL5	1.027	0.640821
Q9Y5K6	CD2-associated protein OS=Homo sapiens OX=9606 GN=CD2AP PE=1 SV=1	CD2AP	1.131	0.232002
Q9Y5L0	Transportin-3 OS=Homo sapiens OX=9606 GN=TNPO3 PE=1	TNPO3	1.235	0.14744
Q9Y5L4	Mitochondrial import inner membrane translocase subunit Tim13 OS=Homo sapiens OX=9606 GN=TIMM13 PE=1	TIMM13	0.989	0.889251
Q9Y5M8	Signal recognition particle receptor subunit beta OS=Homo sapiens OX=9606 GN=SRPRB PE=1	SRPRB	0.968	0.615537
Q9Y5Q8	General transcription factor 3C polypeptide 5 OS=Homo sapiens OX=9606 GN=GTF3C5 PE=1	GTF3C5	0.979	0.80851
Q9Y5Q9	General transcription factor 3C polypeptide 3 OS=Homo sapiens OX=9606 GN=GTF3C3 PE=1	GTF3C3	0.936	0.30729
Q9Y5S2	Serine/threonine-protein kinase MRCK beta OS=Homo sapiens OX=9606 GN=CDC42BPB PE=1 SV=2	CDC42BPB	1.004	0.995422
Q9Y5S9	RNA-binding protein 8A OS=Homo sapiens OX=9606 GN=RBM8A PE=1 SV=1	RBM8A	0.9	0.04594
Q9Y5U2	Protein TSSC4 OS=Homo sapiens OX=9606 GN=TSSC4 PE=1	TSSC4	0.958	0.782984
Q9Y5U8	Mitochondrial pyruvate carrier 1 OS=Homo sapiens OX=9606 GN=MPC1 PE=1 SV=1	MPC1	0.993	0.871059
Q9Y5U9	Immediate early response 3-interacting protein 1 OS=Homo sapiens OX=9606 GN=IER3IP1 PE=1 SV=1	IER3IP1	0.957	0.679333
Q9Y5Y6	Suppressor of tumorigenicity 14 protein OS=Homo sapiens OX=9606 GN=ST14 PE=1 SV=2	ST14	0.828	0.112539
Q9Y606	tRNA pseudouridine synthase A OS=Homo sapiens OX=9606 GN=PUS1 PE=1 SV=3	PUS1	0.976	0.693854
Q9Y617	Phosphoserine aminotransferase OS=Homo sapiens OX=9606 GN=PSAT1 PE=1 SV=2	PSAT1	0.998	0.999355
Q9Y618	Nuclear receptor corepressor 2 OS=Homo sapiens OX=9606 GN=NCOR2 PE=1 SV=3	NCOR2	0.877	0.022251
Q9Y624	Junctional adhesion molecule A OS=Homo sapiens OX=9606 GN=F11R PE=1 SV=1	F11R	0.907	0.272526

Q9Y639	Neuroplastin OS=Homo sapiens OX=9606 GN=NPTN PE=1 SV=2	NPTN	0.955	0.506713
Q9Y653	Adhesion G-protein coupled receptor G1 OS=Homo sapiens OX=9606 GN=ADGRG1 PE=1 SV=2	ADGRG1	1.045	0.684155
Q9Y657	Spindlin-1 OS=Homo sapiens OX=9606 GN=SPIN1 PE=1 SV=3	SPIN1	0.891	0.332895
Q9Y673	Dolichyl-phosphate beta-glucosyltransferase OS=Homo sapiens OX=9606 GN=ALG5 PE=1 SV=1	ALG5	1.019	0.975387
Q9Y676	28S ribosomal protein S18b, mitochondrial OS=Homo sapiens OX=9606 GN=MRPS18B PE=1 SV=1	MRPS18B	1.041	0.564327
Q9Y678	Coatomer subunit gamma-1 OS=Homo sapiens OX=9606 GN=COPG1 PE=1 SV=1	COPG1	1.105	0.314653
Q9Y679	Lipid droplet-regulating VLDL assembly factor AUP1 OS=Homo sapiens OX=9606 GN=AUP1 PE=1 SV=2	AUP1	0.99	0.827534
Q9Y680	Peptidyl-prolyl cis-trans isomerase FKBP7 OS=Homo sapiens OX=9606 GN=FKBP7 PE=1	FKBP7	1.036	0.769172
Q9Y696	Chloride intracellular channel protein 4 OS=Homo sapiens OX=9606 GN=CLIC4 PE=1 SV=4	CLIC4	0.985	0.925693
Q9Y697	Cysteine desulfurase, mitochondrial OS=Homo sapiens OX=9606 GN=NFS1 PE=1 SV=3	NFS1	1.112	0.213415
Q9Y6A4	Cilia- and flagella-associated protein 20 OS=Homo sapiens OX=9606 GN=CFAP20 PE=1	CFAP20	1.016	0.869919
Q9Y6A9	Signal peptidase complex subunit 1 OS=Homo sapiens OX=9606 GN=SPCS1 PE=1 SV=5	SPCS1	0.825	0.110057
Q9Y6B6	GTP-binding protein SAR1b OS=Homo sapiens OX=9606 GN=SAR1B PE=1 SV=1	SAR1B	1.017	0.862723
Q9Y6C9	Mitochondrial carrier homolog 2 OS=Homo sapiens OX=9606 GN=MTCH2 PE=1 SV=1	MTCH2	1.123	0.220572
Q9Y6D5	Brefeldin A-inhibited guanine nucleotide-exchange protein 2 OS=Homo sapiens OX=9606 GN=ARFGEF2 PE=1 SV=3	ARFGEF2	1.309	0.263824
Q9Y6D6	Brefeldin A-inhibited guanine nucleotide-exchange protein 1 OS=Homo sapiens OX=9606 GN=ARFGEF1 PE=1 SV=2	ARFGEF1	1.492	0.273083

Q9Y6D9	Mitotic spindle assembly checkpoint protein MAD1 OS=Homo sapiens OX=9606 GN=MAD1L1 PE=1 SV=2	MAD1L1	0.735	0.041335
Q9Y6E0	Serine/threonine-protein kinase 24 OS=Homo sapiens OX=9606 GN=STK24 PE=1 SV=1	STK24	1.16	0.287564
Q9Y6E2	Basic leucine zipper and W2 domain-containing protein 2 OS=Homo sapiens OX=9606 GN=BZW2 PE=1 SV=1	BZW2	0.937	0.194304
Q9Y6G3	39S ribosomal protein L42, mitochondrial OS=Homo sapiens OX=9606 GN=MRPL42 PE=1 SV=1	MRPL42	1.042	0.848461
Q9Y6G9	Cytoplasmic dynein 1 light intermediate chain 1 OS=Homo sapiens OX=9606 GN=DYNC1LI1 PE=1 SV=3	DYNC1LI1	1.274	0.040702
Q9Y6H1	Coiled-coil-helix-coiled-coil-helix domain-containing protein 2 OS=Homo sapiens OX=9606 GN=CHCHD2 PE=1 SV=1	CHCHD2	0.751	0.031862
Q9Y6I3	Epsin-1 OS=Homo sapiens OX=9606 GN=EPN1 PE=1 SV=2	EPN1	0.935	0.506669
Q9Y6I4	Ubiquitin carboxyl-terminal hydrolase 3 OS=Homo sapiens OX=9606 GN=USP3 PE=1 SV=2	USP3	0.676	
Q9Y6I9	Testis-expressed protein 264 OS=Homo sapiens OX=9606 GN=TEX264 PE=1 SV=1	TEX264	0.945	0.553054
Q9Y6K0	Choline/ethanolaminephosphotransferase 1 OS=Homo sapiens OX=9606 GN=CEPT1 PE=1	CEPT1	1.072	
Q9Y6K5	2'-5'-oligoadenylate synthase 3 OS=Homo sapiens OX=9606 GN=OAS3 PE=1 SV=3	OAS3	1.05	0.674258
Q9Y6M1	Insulin-like growth factor 2 mRNA-binding protein 2 OS=Homo sapiens OX=9606 GN=IGF2BP2 PE=1 SV=2	IGF2BP2	1.033	0.751411
Q9Y6M5	Zinc transporter 1 OS=Homo sapiens OX=9606 GN=SLC30A1 PE=1 SV=3	SLC30A1	0.982	0.877126
Q9Y6M7	Sodium bicarbonate cotransporter 3 OS=Homo sapiens OX=9606 GN=SLC4A7 PE=1 SV=2	SLC4A7	0.945	0.392213
Q9Y6M9	NADH dehydrogenase [ubiquinone] 1 beta subcomplex subunit 9 OS=Homo sapiens OX=9606 GN=NDUFB9 PE=1	NDUFB9	0.998	0.936996

Q9Y6N1	Cytochrome c oxidase assembly protein COX11, mitochondrial OS=Homo sapiens OX=9606 GN=COX11 PE=1 SV=3	COX11	1.138	0.258502
Q9Y6Q5	AP-1 complex subunit mu-2 OS=Homo sapiens OX=9606 GN=AP1M2 PE=1 SV=4	AP1M2	0.978	0.665584
Q9Y6V7	Probable ATP-dependent RNA helicase DDX49 OS=Homo sapiens OX=9606 GN=DDX49	DDX49	1.027	0.837025
Q9Y6W5	Wiskott-Aldrich syndrome protein family member 2 OS=Homo sapiens OX=9606 GN=WASF2	WASF2	1.008	0.99706
Q9Y6X3	MAU2 chromatid cohesion factor homolog OS=Homo sapiens OX=9606 GN=MAU2 PE=1 SV=2	MAU2	0.924	0.205486
Q9Y6X4	Soluble lamin-associated protein of 75 kDa OS=Homo sapiens OX=9606 GN=FAM169A PE=1 SV=2	FAM169A	1.04	0.749316
Q9Y6X9	ATPase MORC2 OS=Homo sapiens OX=9606 GN=MORC2	MORC2	0.869	0.161188
Q9Y6Y8	SEC23-interacting protein OS=Homo sapiens OX=9606 GN=SEC23IP PE=1 SV=1	SEC23IP	1.047	0.657943

Significantly upregulated proteins (FC > 1.5, *P* < 0.05)

Gene name	IFIT3/NC Ratio	IFIT3/NC P value
IFIT3	1000	0.001
TMEM63A	1000	0.001
CCNDBP1	1000	0.001
LYPLA2	1.58	0.007719478
FN1	1.835	0.016962937
PXN	1.587	0.027711177
CASP4	1000	0.001
MGAT5	1000	0.001
GPNMB	1.758	0.007767013
MFSD5	1000	0.001
PKN3	1000	0.001
SLC27A1	1000	0.001
TBC1D5	1000	0.001
SMG1	1000	0.001
KLC2	1000	0.001
SMYD3	1000	0.001
TANC2	1000	0.001
POLE4	1000	0.001
HEATR5B	1000	0.001
KMT2B	1000	0.001

Supplementary file S3:

Mass spectrometry results

Gene names	Protein names	Peptides	Unique peptides	Coverage [%]	Score	MW [kDa]	# AA
ACTB	Actin, cytoplasmic 1 OS=Homo sapiens OX=9606 GN=ACTB PE=1 SV=1	9	9	21	66.12	41.7	375
ALB	Albumin OS=Homo sapiens OX=9606 GN=ALB PE=1 SV=2	9	9	12	33.69	69.3	609
ALDOA	Fructose-bisphosphate aldolase A OS=Homo sapiens OX=9606 GN=ALDOA PE=1 SV=2	8	8	19	30.55	39.4	364
ALYREF	THO complex subunit 4 OS=Homo sapiens OX=9606 GN=ALYREF PE=1 SV=3	2	2	8	11.04	26.9	257
ANXA1	Annexin A1 OS=Homo sapiens OX=9606 GN=ANXA1 PE=1 SV=2	5	5	16	17.3	38.7	346
ANXA2P2	Putative annexin A2-like protein OS=Homo sapiens OX=9606 GN=ANXA2P2 PE=5 SV=2	5	5	16	17.63	38.6	339
AZGP1	Zinc-alpha-2-glycoprotein OS=Homo sapiens OX=9606 GN=AZGP1 PE=1 SV=2	2	2	8	4.64	34.2	298

BCAP31	B-cell receptor-associated protein 31 OS=Homo sapiens OX=9606 GN=BCAP31 PE=1 SV=3	2	2	9	6.57	28	246
CAPRIN1	Caprin-1 OS=Homo sapiens OX=9606 GN=CAPRIN1 PE=1 SV=2	18	18	26	122.1	78.3	709
CASP14	Caspase-14 OS=Homo sapiens OX=9606 GN=CASP14 PE=1 SV=2	2	2	9	7.96	27.7	242
CDK9	Cyclin-dependent kinase 9 OS=Homo sapiens OX=9606 GN=CDK9 PE=1 SV=3	3	2	9	10.42	42.8	372
CHTOP	Chromatin target of PRMT1 protein OS=Homo sapiens OX=9606 GN=CHTOP PE=1 SV=2	4	4	26	38.43	26.4	248
CLIC1	Chloride intracellular channel protein 1 OS=Homo sapiens OX=9606 GN=CLIC1 PE=1 SV=4	3	3	12	7.07	26.9	241
CTTN	Src substrate cortactin OS=Homo sapiens OX=9606 GN=CTTN PE=1 SV=2	18	18	27	59.25	61.5	550
DCD	Dermcidin OS=Homo sapiens OX=9606 GN=DCD PE=1 SV=2	2	2	20	4.91	11.3	110

DDX1	ATP-dependent RNA helicase DDX1 OS=Homo sapiens OX=9606 GN=DDX1 PE=1 SV=2	26	26	31	142.21	82.4	740
DDX17	Probable ATP-dependent RNA helicase DDX17 OS=Homo sapiens OX=9606 GN=DDX17 PE=1 SV=2	11	10	15	46.27	80.2	729
DDX3X	ATP-dependent RNA helicase DDX3X OS=Homo sapiens OX=9606 GN=DDX3X PE=1 SV=3	16	15	24	61.32	73.2	662
EEF1A2	Elongation factor 1-alpha 2 OS=Homo sapiens OX=9606 GN=EEF1A2 PE=1 SV=1	4	4	8	14.55	50.4	463
EEF1D	Elongation factor 1-delta OS=Homo sapiens OX=9606 GN=EEF1D PE=1 SV=5	3	3	18	15.7	31.1	281
EEF2	Elongation factor 2 OS=Homo sapiens OX=9606 GN=EEF2 PE=1 SV=4	5	5	6	14.3	95.3	858
ETFB	Electron transfer flavoprotein subunit beta OS=Homo sapiens OX=9606 GN=ETFB PE=1 SV=3	4	4	15	11.91	27.8	255
EWSR1	RNA-binding protein EWS OS=Homo sapiens OX=9606 GN=EWSR1 PE=1 SV=1	6	6	13	43.54	68.4	656

EZR	Ezrin OS=Homo sapiens OX=9606 GN=EZR PE=1 SV=4	8	6	11	28.83	69.4	586
FBL	rRNA 2'-O-methyltransferase fibrillarin OS=Homo sapiens OX=9606 GN=FBL PE=1 SV=2	5	5	16	25.02	33.8	321
FUBP1	Far upstream element-binding protein 1 OS=Homo sapiens OX=9606 GN=FUBP1 PE=1 SV=3	4	4	6	8.64	67.5	644
FUS	RNA-binding protein FUS OS=Homo sapiens OX=9606 GN=FUS PE=1 SV=1	3	2	6	14.3	53.4	526
GAPDH	Glyceraldehyde-3-phosphate dehydrogenase OS=Homo sapiens OX=9606 GN=GAPDH PE=1	7	7	23	40.82	36	335
H1-3	Histone H1.3 OS=Homo sapiens OX=9606 GN=H1-3 PE=1 SV=2	6	4	19	21.37	22.3	221
H1-5	Histone H1.5 OS=Homo sapiens OX=9606 GN=H1-5 PE=1 SV=3	5	3	13	12.15	22.6	226
HMGB1	High mobility group protein B1 OS=Homo sapiens OX=9606 GN=HMGB1 PE=1 SV=3	3	3	14	12.3	24.9	215

HNRNPA0	Heterogeneous nuclear ribonucleoprotein A0 OS=Homo sapiens OX=9606 GN=HNRNPA0 PE=1 SV=1	5	4	13	17.41	30.8	305
HNRNPA1	Heterogeneous nuclear ribonucleoprotein A1 OS=Homo sapiens OX=9606 GN=HNRNPA1 PE=1 SV=5	5	4	15	27.36	38.7	372
HNRNPA2B1	Heterogeneous nuclear ribonucleoproteins A2/B1 OS=Homo sapiens OX=9606 GN=HNRNPA2B1 PE=1 SV=2	12	11	41	77.91	37.4	353
HNRNPA3	Heterogeneous nuclear ribonucleoprotein A3 OS=Homo sapiens OX=9606 GN=HNRNPA3 PE=1 SV=2	4	3	11	18.27	39.6	378
HNRNPAB	Heterogeneous nuclear ribonucleoprotein A/B OS=Homo sapiens OX=9606 GN=HNRNPAB PE=1 SV=2	4	3	12	21.64	36.2	332
HNRNPD	Heterogeneous nuclear ribonucleoprotein D0 OS=Homo sapiens OX=9606 GN=HNRNPD PE=1 SV=1	8	7	22	35.16	38.4	355
HNRNPDL	Heterogeneous nuclear ribonucleoprotein D-like OS=Homo sapiens OX=9606 GN=HNRNPDL PE=1 SV=3	3	2	6	17.53	46.4	420
HNRNPH3	Heterogeneous nuclear ribonucleoprotein H3 OS=Homo sapiens OX=9606 GN=HNRNPH3 PE=1 SV=2	6	6	23	27.05	36.9	346

HNRNPM	Heterogeneous nuclear ribonucleoprotein M OS=Homo sapiens OX=9606 GN=HNRNPM PE=1 SV=3	12	12	19	40.06	77.5	730
HNRNPR	Heterogeneous nuclear ribonucleoprotein R OS=Homo sapiens OX=9606 GN=HNRNPR PE=1 SV=1	7	2	10	22.98	70.9	633
HNRNPU	Heterogeneous nuclear ribonucleoprotein U OS=Homo sapiens OX=9606 GN=HNRNPU PE=1 SV=6	19	19	17	110.34	90.5	825
HNRNPUL 1	Heterogeneous nuclear ribonucleoprotein U-like protein 1 OS=Homo sapiens OX=9606 GN=HNRNPUL1	8	8	11	26.61	95.7	856
HNRNPUL 2	Heterogeneous nuclear ribonucleoprotein U-like protein 2 OS=Homo sapiens OX=9606 GN=HNRNPUL2	9	9	11	24.16	85.1	747
HRNR	Hornerin OS=Homo sapiens OX=9606 GN=HRNR PE=1 SV=2	8	8	8	27.66	282.2	2850
HSP90AA1	Heat shock protein HSP 90-alpha OS=Homo sapiens OX=9606 GN=HSP90AA1 PE=1 SV=5	13	7	17	49.44	84.6	732
HSP90AB1	Heat shock protein HSP 90-beta OS=Homo sapiens OX=9606 GN=HSP90AB1 PE=1 SV=4	16	10	21	78.97	83.2	724

HSP90B1	Endoplasmin OS=Homo sapiens OX=9606 GN=HSP90B1 PE=1 SV=1	7	7	8	19.6	92.4	803
HSPA1B	Heat shock 70 kDa protein 1B OS=Homo sapiens OX=9606 GN=HSPA1B PE=1 SV=1	13	10	21	64.27	70	641
HSPA5	Endoplasmic reticulum chaperone BiP OS=Homo sapiens OX=9606 GN=HSPA5 PE=1 SV=2	13	12	20	63.66	72.3	654
HSPA8	Heat shock cognate 71 kDa protein OS=Homo sapiens OX=9606 GN=HSPA8 PE=1 SV=1	14	11	22	98.43	70.9	646
HSPA9	Stress-70 protein, mitochondrial OS=Homo sapiens OX=9606 GN=HSPA9 PE=1 SV=2	7	7	11	30.4	73.6	679
HSPB1	Heat shock protein beta-1 OS=Homo sapiens OX=9606 GN=HSPB1 PE=1 SV=2	3	3	19	14.75	22.8	205
IDH1	Isocitrate dehydrogenase [NADP] cytoplasmic OS=Homo sapiens OX=9606 GN=IDH1 PE=1 SV=2	2	2	6	9.26	46.6	414
IFIT2	Interferon-induced protein with tetratricopeptide repeats 2 OS=Homo sapiens OX=9606 GN=IFIT2 PE=1 SV=1	5	4	11	12.51	54.6	472

IFIT3	Interferon-induced protein with tetratricopeptide repeats 3 OS=Homo sapiens OX=9606 GN=IFIT3 PE=1 SV=1	16	15	32	106.8	56	490
IGHG1	Immunoglobulin heavy constant gamma 1 OS=Homo sapiens OX=9606 GN=IGHG1 PE=1 SV=1	3	3	9	9.02	36.1	330
JUP	Junction plakoglobin OS=Homo sapiens OX=9606 GN=JUP PE=1 SV=3	4	4	6	15.15	81.7	745
KHSRP	Far upstream element-binding protein 2 OS=Homo sapiens OX=9606 GN=KHSRP PE=1 SV=4	4	4	6	13.09	73.1	711
KRT1	Keratin, type II cytoskeletal 1 OS=Homo sapiens OX=9606 GN=KRT1 PE=1 SV=6	33	27	48	312.06	66	644
KRT10	Keratin, type I cytoskeletal 10 OS=Homo sapiens OX=9606 GN=KRT10 PE=1 SV=6	27	22	34	261.11	58.8	584
KRT14	Keratin, type I cytoskeletal 14 OS=Homo sapiens OX=9606 GN=KRT14 PE=1 SV=4	22	5	48	121.4	51.5	472
KRT16	Keratin, type I cytoskeletal 16 OS=Homo sapiens OX=9606 GN=KRT16 PE=1 SV=4	18	6	40	93.77	51.2	473

KRT17	Keratin, type I cytoskeletal 17 OS=Homo sapiens OX=9606 GN=KRT17 PE=1 SV=2	14	3	27	79.31	48.1	432
KRT18	Keratin, type I cytoskeletal 18 OS=Homo sapiens OX=9606 GN=KRT18 PE=1 SV=2	10	8	28	53.77	48	430
KRT19	Keratin, type I cytoskeletal 19 OS=Homo sapiens OX=9606 GN=KRT19 PE=1 SV=4	23	14	51	169.8	44.1	400
KRT2	Keratin, type II cytoskeletal 2 epidermal OS=Homo sapiens OX=9606 GN=KRT2 PE=1 SV=2	30	24	45	266.79	65.4	639
KRT5	Keratin, type II cytoskeletal 5 OS=Homo sapiens OX=9606 GN=KRT5 PE=1 SV=3	27	16	38	128.56	62.3	590
KRT6A	Keratin, type II cytoskeletal 6A OS=Homo sapiens OX=9606 GN=KRT6A PE=1 SV=3	20	3	34	98.73	60	564
KRT6B	Keratin, type II cytoskeletal 6B OS=Homo sapiens OX=9606 GN=KRT6B PE=1 SV=5	17	2	30	106.53	60	564
KRT7	Keratin, type II cytoskeletal 7 OS=Homo sapiens OX=9606 GN=KRT7 PE=1 SV=5	8	5	17	26.1	51.4	469

KRT77	Keratin, type II cytoskeletal 1b OS=Homo sapiens OX=9606 GN=KRT77 PE=1 SV=3	5	2	9	27.52	61.9	578
KRT8	Keratin, type II cytoskeletal 8 OS=Homo sapiens OX=9606 GN=KRT8 PE=1 SV=7	13	8	21	58.21	53.7	483
KRT80	Keratin, type II cytoskeletal 80 OS=Homo sapiens OX=9606 GN=KRT80 PE=1 SV=2	4	4	9	9.88	50.5	452
KRT9	Keratin, type I cytoskeletal 9 OS=Homo sapiens OX=9606 GN=KRT9 PE=1 SV=3	18	18	29	125.12	62	623
L1RE1	LINE-1 retrotransposable element ORF1 protein OS=Homo sapiens OX=9606 GN=L1RE1 PE=1 SV=1	3	3	10	9.17	40	338
LASP1	LIM and SH3 domain protein 1 OS=Homo sapiens OX=9606 GN=LASP1 PE=1 SV=2	2	2	9	5.25	29.7	261
LDHA	L-lactate dehydrogenase A chain OS=Homo sapiens OX=9606 GN=LDHA PE=1 SV=2	4	2	12	11.91	36.7	332
LDHB	L-lactate dehydrogenase B chain OS=Homo sapiens OX=9606 GN=LDHB PE=1 SV=2	7	5	23	24.72	36.6	334

LMNA	Prelamin-A/C OS=Homo sapiens OX=9606 GN=LMNA PE=1 SV=1	7	7	13	23.52	74.1	664
LUC7L2	Putative RNA-binding protein Luc7-like 2 OS=Homo sapiens OX=9606 GN=LUC7L2 PE=1 SV=2	2	2	6	8.74	46.5	392
MDH2	Malate dehydrogenase, mitochondrial OS=Homo sapiens OX=9606 GN=MDH2 PE=1 SV=3	4	4	14	18.12	35.5	338
MISP	Mitotic interactor and substrate of PLK1 OS=Homo sapiens OX=9606 GN=MISP PE=1 SV=1	3	3	6	7.43	75.3	679
mmunoglobulin	Immunoglobulin lambda-1 light chain OS=Homo sapiens OX=9606 PE=1 SV=1	2	2	10	8.27	22.8	216
NCL	Nucleolin OS=Homo sapiens OX=9606 GN=NCL PE=1 SV=3	6	6	8	15.05	76.6	710
PABPC1	Polyadenylate-binding protein 1 OS=Homo sapiens OX=9606 GN=PABPC1 PE=1 SV=2	4	4	6	9.28	70.6	636
PCBP1	Poly(rC)-binding protein 1 OS=Homo sapiens OX=9606 GN=PCBP1 PE=1 SV=2	4	2	16	16.73	37.5	356

PDCD6IP	Programmed cell death 6-interacting protein OS=Homo sapiens OX=9606 GN=PDCD6IP PE=1 SV=1	6	6	9	19.04	96	868
PGAM1	Phosphoglycerate mutase 1 OS=Homo sapiens OX=9606 GN=PGAM1 PE=1 SV=2	3	3	15	11.22	28.8	254
PHB1	Prohibitin 1 OS=Homo sapiens OX=9606 GN=PHB1 PE=1 SV=1	4	4	15	11.03	29.8	272
PHB2	Prohibitin-2 OS=Homo sapiens OX=9606 GN=PHB2 PE=1 SV=2	4	4	15	11.9	33.3	299
PKM	Pyruvate kinase PKM OS=Homo sapiens OX=9606 GN=PKM PE=1 SV=4	6	6	14	21.09	57.9	531
PLIN4	Perilipin-4 OS=Homo sapiens OX=9606 GN=PLIN4 PE=1 SV=2	4	4	7	9.54	134.3	1357
PNN	Pinin OS=Homo sapiens OX=9606 GN=PNN PE=1 SV=5	11	11	12	34.3	81.6	717
PRDX2	Peroxiredoxin-2 OS=Homo sapiens OX=9606 GN=PRDX2 PE=1 SV=5	3	3	15	9.65	21.9	198

PSMA1	Proteasome subunit alpha type-1 OS=Homo sapiens OX=9606 GN=PSMA1 PE=1 SV=1	2	2	13	5.84	29.5	263
PTHLH	Parathyroid hormone-related protein OS=Homo sapiens OX=9606 GN=PTHLH PE=1 SV=1	5	5	20	23.99	20.2	177
RANBP1	Ran-specific GTPase-activating protein OS=Homo sapiens OX=9606 GN=RANBP1 PE=1 SV=1	2	2	11	4.68	23.3	201
RBM14	RNA-binding protein 14 OS=Homo sapiens OX=9606 GN=RBM14 PE=1 SV=2	7	7	14	22.95	69.4	669
RBMX	RNA-binding motif protein, X chromosome OS=Homo sapiens OX=9606 GN=RBMX PE=1 SV=3	13	9	27	68.19	42.3	391
RPL10A	60S ribosomal protein L10a OS=Homo sapiens OX=9606 GN=RPL10A PE=1 SV=2	3	3	16	6.83	24.8	217
RPL13	60S ribosomal protein L13 OS=Homo sapiens OX=9606 GN=RPL13 PE=1 SV=4	7	7	31	27.64	24.2	211
RPL13A	60S ribosomal protein L13a OS=Homo sapiens OX=9606 GN=RPL13A PE=1 SV=2	5	5	21	13.31	23.6	203

RPL14	60S ribosomal protein L14 OS=Homo sapiens OX=9606 GN=RPL14 PE=1 SV=4	2	2	9	4.91	23.4	215
RPL15	60S ribosomal protein L15 OS=Homo sapiens OX=9606 GN=RPL15 PE=1 SV=2	2	2	8	4.27	24.1	204
RPL17	60S ribosomal protein L17 OS=Homo sapiens OX=9606 GN=RPL17 PE=1 SV=3	2	2	10	4.5	21.4	184
RPL19	60S ribosomal protein L19 OS=Homo sapiens OX=9606 GN=RPL19 PE=1 SV=1	8	8	31	25.64	23.5	196
RPL24	60S ribosomal protein L24 OS=Homo sapiens OX=9606 GN=RPL24 PE=1 SV=1	3	3	14	10.94	17.8	157
RPL5	60S ribosomal protein L5 OS=Homo sapiens OX=9606 GN=RPL5 PE=1 SV=3	3	3	11	7.16	34.3	297
RPL7	60S ribosomal protein L7 OS=Homo sapiens OX=9606 GN=RPL7 PE=1 SV=1	7	7	18	17.14	29.2	248
RPL7A	60S ribosomal protein L7a OS=Homo sapiens OX=9606 GN=RPL7A PE=1 SV=2	7	7	23	27.95	30	266

RPL8	60S ribosomal protein L8 OS=Homo sapiens OX=9606 GN=RPL8 PE=1 SV=2	5	5	14	15.68	28	257
RPLP0	60S acidic ribosomal protein P0 OS=Homo sapiens OX=9606 GN=RPLP0 PE=1 SV=1	4	4	17	10.2	34.3	317
RPS19	40S ribosomal protein S19 OS=Homo sapiens OX=9606 GN=RPS19 PE=1 SV=2	2	2	13	4.26	16.1	145
RPS2	40S ribosomal protein S2 OS=Homo sapiens OX=9606 GN=RPS2 PE=1 SV=2	4	4	13	13.47	31.3	293
RPS3	40S ribosomal protein S3 OS=Homo sapiens OX=9606 GN=RPS3 PE=1 SV=2	4	4	23	15.6	26.7	243
RPS3A	40S ribosomal protein S3a OS=Homo sapiens OX=9606 GN=RPS3A PE=1 SV=2	5	5	14	17.85	29.9	264
RPS4X	40S ribosomal protein S4, X isoform OS=Homo sapiens OX=9606 GN=RPS4X PE=1 SV=2	4	4	15	9.97	29.6	263
RPS6	40S ribosomal protein S6 OS=Homo sapiens OX=9606 GN=RPS6 PE=1 SV=1	6	6	23	25.36	28.7	249

RPS8	40S ribosomal protein S8 OS=Homo sapiens OX=9606 GN=RPS8 PE=1 SV=2	4	4	16	14.51	24.2	208
RPSA	40S ribosomal protein SA OS=Homo sapiens OX=9606 GN=RPSA PE=1 SV=4	3	3	11	8.24	32.8	295
RTCB	RNA-splicing ligase RtcB homolog OS=Homo sapiens OX=9606 GN=RTCB PE=1 SV=1	7	7	16	19.97	55.2	505
RTF2	Replication termination factor 2 OS=Homo sapiens OX=9606 GN=RTF2 PE=1 SV=3	2	2	8	5.15	33.9	306
RTRAF	RNA transcription, translation and transport factor protein OS=Homo sapiens OX=9606 GN=RTRAF PE=1	5	5	19	19.38	28.1	244
SARNP	SAP domain-containing ribonucleoprotein OS=Homo sapiens OX=9606 GN=SARNP PE=1	2	2	10	3.92	23.7	210
SERBP1	Plasminogen activator inhibitor 1 RNA-binding protein OS=Homo sapiens OX=9606 GN=SERBP1 PE=1	3	3	7	9.22	44.9	408
SF1	Splicing factor 1 OS=Homo sapiens OX=9606 GN=SF1 PE=1 SV=4	4	4	8	10.71	68.3	639

SFN	14-3-3 protein sigma OS=Homo sapiens OX=9606 GN=SFN PE=1 SV=1	5	4	26	17.12	27.8	248
SFPQ	Splicing factor, proline- and glutamine-rich OS=Homo sapiens OX=9606 GN=SFPQ PE=1 SV=2	18	18	26	107.97	76.1	707
SPOP	Speckle-type POZ protein OS=Homo sapiens OX=9606 GN=SPOP PE=1 SV=1	3	3	11	14.29	42.1	374
SYNCRIP	Heterogeneous nuclear ribonucleoprotein Q OS=Homo sapiens OX=9606 GN=SYNCRIP PE=1 SV=2	10	5	15	38.66	69.6	623
TAF15	TATA-binding protein-associated factor 2N OS=Homo sapiens OX=9606 GN=TAF15 PE=1 SV=1	3	2	11	8.11	61.8	592
TALDO1	Transaldolase OS=Homo sapiens OX=9606 GN=TALDO1 PE=1 SV=2	6	6	18	13.85	37.5	337
TKT	Transketolase OS=Homo sapiens OX=9606 GN=TKT PE=1 SV=3	3	3	6	10.75	67.8	623
TOP1	DNA topoisomerase 1 OS=Homo sapiens OX=9606 GN=TOP1 PE=1 SV=2	5	5	7	14.02	90.7	765

TPD52L2	Tumor protein D54 OS=Homo sapiens OX=9606 GN=TPD52L2 PE=1 SV=2	2	2	12	5.54	22.2	206
TPI1	Triosephosphate isomerase OS=Homo sapiens OX=9606 GN=TPI1 PE=1 SV=4	5	5	24	21.57	26.7	249
TPM3	Tropomyosin alpha-3 chain OS=Homo sapiens OX=9606 GN=TPM3 PE=1 SV=2	3	3	14	8.51	32.9	285
TRIM21	E3 ubiquitin-protein ligase TRIM21 OS=Homo sapiens OX=9606 GN=TRIM21 PE=1 SV=1	3	3	6	8.82	54.1	475
TSFM	Elongation factor Ts, mitochondrial OS=Homo sapiens OX=9606 GN=TSFM PE=1 SV=2	2	2	7	4.47	35.4	325
VDAC1	Voltage-dependent anion-selective channel protein 1 OS=Homo sapiens OX=9606 GN=VDAC1 PE=1 SV=2	2	2	9	5.35	30.8	283
XRCC6	X-ray repair cross-complementing protein 6 OS=Homo sapiens OX=9606 GN=XRCC6 PE=1 SV=2	5	5	8	22.46	69.8	609
YWHAE	14-3-3 protein epsilon OS=Homo sapiens OX=9606 GN=YWHAE PE=1 SV=1	5	4	22	17.83	29.2	255

YWHAG	14-3-3 protein gamma OS=Homo sapiens OX=9606 GN=YWHAG PE=1 SV=2	5	3	23	23.03	28.3	247
YWHAQ	14-3-3 protein theta OS=Homo sapiens OX=9606 GN=YWHAQ PE=1 SV=1	3	2	13	10.48	27.7	245
YWHAZ	14-3-3 protein zeta/delta OS=Homo sapiens OX=9606 GN=YWHAZ PE=1 SV=1	8	7	35	41.32	27.7	245
ZC3HAV1	Zinc finger CCCH-type antiviral protein 1 OS=Homo sapiens OX=9606 GN=ZC3HAV1 PE=1 SV=3	5	5	7	14.3	101.4	902
ZNF326	DBIRD complex subunit ZNF326 OS=Homo sapiens OX=9606 GN=ZNF326 PE=1 SV=2	7	7	13	21.44	65.6	582

Gene names	Location
ACTB	
ALB	Localized to the Golgi apparatus (enhanced)
ANXA1	Localized to the Plasma membrane (supported), Cytosol (supported)
CTTN	Localized to the Plasma membrane (supported), Cytosol (approved)
EZR	Localized to the Plasma membrane (supported)
HMGB1	Localized to the Nucleoplasm (supported)
HNRNPU	Localized to the Nucleoplasm (supported)
HSP90AA1	Localized to the Cytosol (enhanced)
HSP90B1	Localized to the Endoplasmic reticulum (enhanced)
HSPA5	Localized to the Cytosol (approved)
HSPA8	Localized to the Nucleoplasm (approved)
HSPB1	Localized to the Plasma membrane (enhanced), Cytosol (enhanced)
JUP	Localized to the Plasma membrane (supported), Cell Junctions (enhanced)

KRT1	
KRT14	Localized to the Intermediate filaments (enhanced)
KRT16	Localized to the Intermediate filaments (approved)
KRT17	Localized to the Intermediate filaments (enhanced)
KRT19	Localized to the Intermediate filaments (supported)
KRT2	
KRT5	Localized to the Intermediate filaments (approved)
KRT6A	Localized to the Intermediate filaments (enhanced)
KRT6B	Localized to the Intermediate filaments (approved)
KRT7	Localized to the Intermediate filaments (approved), Cytosol (supported)
KRT8	Localized to the Intermediate filaments (supported)
LASP1	Localized to the Plasma membrane (supported), Cytosol (supported)
LMNA	Localized to the Nuclear speckles (supported)
MDH2	Localized to the Mitochondria (enhanced)
MISP	Localized to the Plasma membrane (enhanced), Focal adhesion sites (enhanced)
PABPC1	Localized to the Cytosol (supported)
PDCD6IP	Localized to the Vesicles (approved)
RPL7A	Localized to the Nucleoli (approved)
RPSA	Localized to the Plasma membrane (supported), Cytosol (supported)
YWHAE	Localized to the Cytosol (supported)
YWHAG	Localized to the Cytosol (approved)
YWHAQ	Localized to the Cytosol (supported)
YWHAZ	
HSP90AB1	Localized to the Cytosol (supported)
KRT9	
RTCB	Localized to the Nucleoplasm (supported);In addition localized to the Vesicles (approved), Cytosol (supported)

Supplementary file S4:

Genes associated with Focal adhesion signaling pathway

Gene Symbol	Description	Category	Uniprot ID	Gifts	GC Id	Relevance score	GeneCards Link
ITGB2	Integrin Subunit Beta 2	Protein Coding	P05107	54	GC21M044885	156.395386	https://www.genecards.org/cgi-bin/carddisp.pl?gene=ITGB2
PTK2	Protein Tyrosine Kinase 2	Protein Coding	Q05397	51	GC08M140657	104.144821	https://www.genecards.org/cgi-bin/carddisp.pl?gene=PTK2
FERMT3	FERM Domain Containing Kindlin 3	Protein Coding	Q86UX7	48	GC11P081824	93.4111023	https://www.genecards.org/cgi-bin/carddisp.pl?gene=FERMT3
INF2	Inverted Formin 2	Protein Coding	Q27J81	45	GC14P113818	77.4274063	https://www.genecards.org/cgi-bin/carddisp.pl?gene=INF2
ICAM1	Intercellular Adhesion Molecule 1	Protein Coding	P05362	53	GC19P095217	76.6736145	https://www.genecards.org/cgi-bin/carddisp.pl?gene=ICAM1
TSC1	TSC Complex Subunit 1	Protein Coding	Q92574	51	GC09M132891	73.324295	https://www.genecards.org/cgi-bin/carddisp.pl?gene=TSC1
GRIN2A	Glutamate Ionotropic Receptor NMDA Type Subunit 2A	Protein Coding	Q12879	55	GC16M009753	73.0587311	https://www.genecards.org/cgi-bin/carddisp.pl?gene=GRIN2A
TSC2	TSC Complex Subunit 2	Protein Coding	P49815	54	GC16P059813	70.1224213	https://www.genecards.org/cgi-bin/carddisp.pl?gene=TSC2
VCAM1	Vascular Cell Adhesion Molecule 1	Protein Coding	P19320	50	GC01P100719	66.5908203	https://www.genecards.org/cgi-bin/carddisp.pl?gene=VCAM1
CNTNAP2	Contactin Associated Protein 2	Protein Coding	Q9UHC6	49	GC07P146116	65.085762	https://www.genecards.org/cgi-bin/carddisp.pl?gene=CNTNAP2
PXN	Paxillin	Protein Coding	P49023	49	GC12M120210	57.1633453	https://www.genecards.org/cgi-bin/carddisp.pl?gene=PXN
NPHS1	NPHS1 Adhesion Molecule, Nephrin	Protein Coding	O60500	51	GC19M035825	56.642807	https://www.genecards.org/cgi-bin/carddisp.pl?gene=NPHS1
ITGB1	Integrin Subunit Beta 1	Protein Coding	P05556	53	GC10M035059	54.8247719	https://www.genecards.org/cgi-bin/carddisp.pl?gene=ITGB1
ACTN4	Actinin Alpha 4	Protein Coding	O43707	50	GC19P038647	54.0784111	https://www.genecards.org/cgi-bin/carddisp.pl?gene=ACTN4
SLC35C1	Solute Carrier Family 35 Member C1	Protein Coding	Q96A29	45	GC11P047178	54.0224152	https://www.genecards.org/cgi-bin/carddisp.pl?gene=SLC35C1
TRPV3	Transient Receptor Potential Cation Channel Subfamily V Member 3	Protein Coding	Q8NET8	46	GC17M015068	51.5632286	https://www.genecards.org/cgi-bin/carddisp.pl?gene=TRPV3
CD2AP	CD2 Associated Protein	Protein Coding	Q9Y5K6	47	GC06P119158	51.5564957	https://www.genecards.org/cgi-bin/carddisp.pl?gene=CD2AP
SRC	SRC Proto-Oncogene, Non-Receptor Tyrosine Kinase	Protein Coding	P12931	54	GC20P037344	51.3313675	https://www.genecards.org/cgi-bin/carddisp.pl?gene=SRC
TRPC6	Transient Receptor Potential Cation Channel Subfamily C Member 6	Protein Coding	Q9Y210	53	GC11M101451	49.8532448	https://www.genecards.org/cgi-bin/carddisp.pl?gene=TRPC6
MTOR	Mechanistic Target Of Rapamycin Kinase	Protein Coding	P42345	59	GC01M011106	47.5844841	https://www.genecards.org/cgi-bin/carddisp.pl?gene=MTOR
SELE	Selectin E	Protein Coding	P16581	47	GC01M169722	46.2983017	https://www.genecards.org/cgi-bin/carddisp.pl?gene=SELE
PTK2B	Protein Tyrosine Kinase 2 Beta	Protein Coding	Q14289	53	GC08P027311	45.6278305	https://www.genecards.org/cgi-bin/carddisp.pl?gene=PTK2B
PAX2	Paired Box 2	Protein Coding	Q02962	50	GC10P100735	44.0059967	https://www.genecards.org/cgi-bin/carddisp.pl?gene=PAX2

NCAM1	Neural Cell Adhesion Molecule 1	Protein Coding	P13591	52	GC11P112961	43.8765182	https://www.genecards.org/cgi-bin/carddisp.pl?gene=NCAM1
VCL	Vinculin	Protein Coding	P18206	51	GC10P073995	43.2142715	https://www.genecards.org/cgi-bin/carddisp.pl?gene=VCL
ITGA4	Integrin Subunit Alpha 4	Protein Coding	P13612	52	GC02P181456	42.8804817	https://www.genecards.org/cgi-bin/carddisp.pl?gene=ITGA4
NPHS2	NPHS2 Stomatin Family Member, Podocin	Protein Coding	Q9NP85	46	GC01M179554	41.095993	https://www.genecards.org/cgi-bin/carddisp.pl?gene=NPHS2
PECAM1	Platelet And Endothelial Cell Adhesion Molecule 1	Protein Coding	P16284	45	GC17M064319	39.4796867	https://www.genecards.org/cgi-bin/carddisp.pl?gene=PECAM1
ITGAL	Integrin Subunit Alpha L	Protein Coding	P20701	50	GC16P030472	39.4063683	https://www.genecards.org/cgi-bin/carddisp.pl?gene=ITGAL
TLN1	Talin 1	Protein Coding	Q9Y490	46	GC09M035696	37.9474907	https://www.genecards.org/cgi-bin/carddisp.pl?gene=TLN1
ITGB3	Integrin Subunit Beta 3	Protein Coding	P05106	55	GC17P093864	37.1249161	https://www.genecards.org/cgi-bin/carddisp.pl?gene=ITGB3
ICAM2	Intercellular Adhesion Molecule 2	Protein Coding	P13598	48	GC17M064002	36.0598679	https://www.genecards.org/cgi-bin/carddisp.pl?gene=ICAM2
APOL1	Apolipoprotein L1	Protein Coding	O14791	48	GC22P036253	35.9274712	https://www.genecards.org/cgi-bin/carddisp.pl?gene=APOL1
CTNNB1	Catenin Beta 1	Protein Coding	P35222	57	GC03P041194	35.8802643	https://www.genecards.org/cgi-bin/carddisp.pl?gene=CTNNB1
CDH2	Cadherin 2	Protein Coding	P19022	55	GC18M032084	35.7129745	https://www.genecards.org/cgi-bin/carddisp.pl?gene=CDH2
BCAR1	BCAR1 Scaffold Protein, Cas Family Member	Protein Coding	P56945	47	GC16M075228	35.1642075	https://www.genecards.org/cgi-bin/carddisp.pl?gene=BCAR1
SELP	Selectin P	Protein Coding	P16109	48	GC01M169558	35.024498	https://www.genecards.org/cgi-bin/carddisp.pl?gene=SELP
NPRL2	NPR2 Like, GATOR1 Complex Subunit	Protein Coding	Q8WTW4	44	GC03M053211	34.3836441	https://www.genecards.org/cgi-bin/carddisp.pl?gene=NPRL2
ITGAV	Integrin Subunit Alpha V	Protein Coding	P06756	51	GC02P186589	34.0845795	https://www.genecards.org/cgi-bin/carddisp.pl?gene=ITGAV
CDH1	Cadherin 1	Protein Coding	P12830	53	GC16P068737	33.312233	https://www.genecards.org/cgi-bin/carddisp.pl?gene=CDH1
CEACAM1	CEA Cell Adhesion Molecule 1	Protein Coding	P13688	48	GC19M042507	33.2133865	https://www.genecards.org/cgi-bin/carddisp.pl?gene=CEACAM1
SELL	Selectin L	Protein Coding	P14151	46	GC01M169690	32.9996758	https://www.genecards.org/cgi-bin/carddisp.pl?gene=SELL
VTN	Vitronectin	Protein Coding	P04004	47	GC17M067441	32.9221687	https://www.genecards.org/cgi-bin/carddisp.pl?gene=VTN
EPCAM	Epithelial Cell Adhesion Molecule	Protein Coding	P16422	52	GC02P047345	32.521431	https://www.genecards.org/cgi-bin/carddisp.pl?gene=EPCAM
MAPK1	Mitogen-Activated Protein Kinase 1	Protein Coding	P28482	57	GC22M021759	32.4780617	https://www.genecards.org/cgi-bin/carddisp.pl?gene=MAPK1
PLCE1	Phospholipase C Epsilon 1	Protein Coding	Q9P212	48	GC10P093993	32.3963814	https://www.genecards.org/cgi-bin/carddisp.pl?gene=PLCE1
SCN1A	Sodium Voltage-Gated Channel Alpha Subunit 1	Protein Coding	P35498	51	GC02M165989	32.3695107	https://www.genecards.org/cgi-bin/carddisp.pl?gene=SCN1A
L1CAM	L1 Cell Adhesion Molecule	Protein Coding	P32004	51	GC0XM153864	32.2868385	https://www.genecards.org/cgi-bin/carddisp.pl?gene=L1CAM

COL4A4	Collagen Type IV Alpha 4 Chain	Protein Coding	P53420	47	GC02M2 26973	32.1595688	https://www.genecards.org/cgi-bin/carddisp.pl?gene=COL4A4
FN1	Fibronectin 1	Protein Coding	P02751	54	GC02M2 15360	31.2260857	https://www.genecards.org/cgi-bin/carddisp.pl?gene=FN1
WT1	WT1 Transcription Factor	Protein Coding	P19544	52	GC11M0 32365	30.9351959	https://www.genecards.org/cgi-bin/carddisp.pl?gene=WT1
SCN2A	Sodium Voltage-Gated Channel Alpha Subunit 2	Protein Coding	Q99250	52	GC02P1 65194	30.6433697	https://www.genecards.org/cgi-bin/carddisp.pl?gene=SCN2A
MYO1E	Myosin IE	Protein Coding	Q12965	48	GC15M0 59132	30.5742435	https://www.genecards.org/cgi-bin/carddisp.pl?gene=MYO1E
ITGA5	Integrin Subunit Alpha 5	Protein Coding	P08648	52	GC12M0 55460	30.4760818	https://www.genecards.org/cgi-bin/carddisp.pl?gene=ITGA5
CRB2	Crumbs Cell Polarity Complex Component 2	Protein Coding	Q5IJ48	45	GC09P1 23356	29.7559032	https://www.genecards.org/cgi-bin/carddisp.pl?gene=CRB2
KRT16	Keratin 16	Protein Coding	P08779	46	GC17M0 41609	29.6759968	https://www.genecards.org/cgi-bin/carddisp.pl?gene=KRT16
AKT1	AKT Serine/Threonine Kinase 1	Protein Coding	P31749	57	GC14M1 04769	29.5945625	https://www.genecards.org/cgi-bin/carddisp.pl?gene=AKT1
CD44	CD44 Molecule (Indian Blood Group)	Protein Coding	P16070	52	GC11P0 35139	28.7256546	https://www.genecards.org/cgi-bin/carddisp.pl?gene=CD44
TNF	Tumor Necrosis Factor	Protein Coding	P01375	55	GC06P1 18895	28.6265373	https://www.genecards.org/cgi-bin/carddisp.pl?gene=TNF
FERMT2	FERM Domain Containing Kindlin 2	Protein Coding	Q96AC1	43	GC14M0 52857	28.6234207	https://www.genecards.org/cgi-bin/carddisp.pl?gene=FERMT2
ALCAM	Activated Leukocyte Cell Adhesion Molecule	Protein Coding	Q13740	46	GC03P1 05366	28.5841465	https://www.genecards.org/cgi-bin/carddisp.pl?gene=ALCAM
RHOA	Ras Homolog Family Member A	Protein Coding	P61586	53	GC03M0 49359	28.3908386	https://www.genecards.org/cgi-bin/carddisp.pl?gene=RHOA
LAMB2	Laminin Subunit Beta 2	Protein Coding	P55268	50	GC03M0 49121	28.2007694	https://www.genecards.org/cgi-bin/carddisp.pl?gene=LAMB2
LMX1B	LIM Homeobox Transcription Factor 1 Beta	Protein Coding	O60663	47	GC09P1 26618	27.9186115	https://www.genecards.org/cgi-bin/carddisp.pl?gene=LMX1B
VWF	Von Willebrand Factor	Protein Coding	P04275	52	GC12M0 06422	27.9020424	https://www.genecards.org/cgi-bin/carddisp.pl?gene=VWF
DSG1	Desmoglein 1	Protein Coding	Q02413	46	GC18P0 31318	27.8697739	https://www.genecards.org/cgi-bin/carddisp.pl?gene=DSG1
ICAM3	Intercellular Adhesion Molecule 3	Protein Coding	P32942	47	GC19M0 17186	27.8205109	https://www.genecards.org/cgi-bin/carddisp.pl?gene=ICAM3
ILK	Integrin Linked Kinase	Protein Coding	Q13418	48	GC11P0 07159	27.6848526	https://www.genecards.org/cgi-bin/carddisp.pl?gene=ILK
ITGAM	Integrin Subunit Alpha M	Protein Coding	P11215	51	GC16P0 60874	27.5597725	https://www.genecards.org/cgi-bin/carddisp.pl?gene=ITGAM
MCAM	Melanoma Cell Adhesion Molecule	Protein Coding	P43121	45	GC11M1 19308	27.4481678	https://www.genecards.org/cgi-bin/carddisp.pl?gene=MCAM
CADM1	Cell Adhesion Molecule 1	Protein Coding	Q9BY67	48	GC11M1 15169	27.2768497	https://www.genecards.org/cgi-bin/carddisp.pl?gene=CADM1
CEACAM6	CEA Cell Adhesion Molecule 6	Protein Coding	P40199	44	GC19P0 41750	27.155571	https://www.genecards.org/cgi-bin/carddisp.pl?gene=CEACAM6
PTEN	Phosphatase And Tensin Homolog	Protein Coding	P60484	55	GC10P1 04451	27.0331383	https://www.genecards.org/cgi-bin/carddisp.pl?gene=PTEN

ZYX	Zyxin	Protein Coding	Q15942	49	GC07P143381	26.9733887	https://www.genecards.org/cgi-bin/carddisp.pl?gene=ZYX
TWIST2	Twist Family BHLH Transcription Factor 2	Protein Coding	Q8WVJ9	47	GC02P238848	26.8090954	https://www.genecards.org/cgi-bin/carddisp.pl?gene=TWIST2
RAC1	Rac Family Small GTPase 1	Protein Coding	P63000	52	GC07P009178	26.6603088	https://www.genecards.org/cgi-bin/carddisp.pl?gene=RAC1
ITGA3	Integrin Subunit Alpha 3	Protein Coding	P26006	52	GC17P050055	26.6076431	https://www.genecards.org/cgi-bin/carddisp.pl?gene=ITGA3
SCN3A	Sodium Voltage-Gated Channel Alpha Subunit 3	Protein Coding	Q9NY46	51	GC02M165087	26.5086613	https://www.genecards.org/cgi-bin/carddisp.pl?gene=SCN3A
VASP	Vasodilator Stimulated Phosphoprotein	Protein Coding	P50552	47	GC19P096186	26.0897026	https://www.genecards.org/cgi-bin/carddisp.pl?gene=VASP
SMARCAL1	SWI/SNF Related, Matrix Associated, Actin Dependent Regulator Of Chromatin, Subfamily A Like 1	Protein Coding	Q9NZC9	49	GC02P216412	26.067728	https://www.genecards.org/cgi-bin/carddisp.pl?gene=SMARCAL1
PARVA	Parvin Alpha	Protein Coding	Q9NVD7	46	GC11P012398	25.9796715	https://www.genecards.org/cgi-bin/carddisp.pl?gene=PARVA
NRCAM	Neuronal Cell Adhesion Molecule	Protein Coding	Q92823	48	GC07M108147	25.9049892	https://www.genecards.org/cgi-bin/carddisp.pl?gene=NRCAM
FERMT1	FERM Domain Containing Kindlin 1	Protein Coding	Q9BQL6	47	GC20M006074	25.8593025	https://www.genecards.org/cgi-bin/carddisp.pl?gene=FERMT1
KIRREL2	Kirre Like Nephrin Family Adhesion Molecule 2	Protein Coding	Q6UWL6	45	GC19P095867	25.8391972	https://www.genecards.org/cgi-bin/carddisp.pl?gene=KIRREL2
ITGA2	Integrin Subunit Alpha 2	Protein Coding	P17301	49	GC05P052989	25.6418171	https://www.genecards.org/cgi-bin/carddisp.pl?gene=ITGA2
ITGB4	Integrin Subunit Beta 4	Protein Coding	P16144	53	GC17P075721	25.5415382	https://www.genecards.org/cgi-bin/carddisp.pl?gene=ITGB4
MADCAM1	Mucosal Vascular Addressin Cell Adhesion Molecule 1	Protein Coding	Q13477	44	GC19P094709	25.4380035	https://www.genecards.org/cgi-bin/carddisp.pl?gene=MADCAM1
CEACAM5	CEA Cell Adhesion Molecule 5	Protein Coding	P06731	47	GC19P096057	24.6229649	https://www.genecards.org/cgi-bin/carddisp.pl?gene=CEACAM5
FYN	FYN Proto-Oncogene, Src Family Tyrosine Kinase	Protein Coding	P06241	51	GC06M111660	24.5676689	https://www.genecards.org/cgi-bin/carddisp.pl?gene=FYN
ADGRG1	Adhesion G Protein-Coupled Receptor G1	Protein Coding	Q9Y653	46	GC16P057610	24.5484104	https://www.genecards.org/cgi-bin/carddisp.pl?gene=ADGRG1
ANLN	Anillin, Actin Binding Protein	Protein Coding	Q9NQW6	47	GC07P036389	24.0536137	https://www.genecards.org/cgi-bin/carddisp.pl?gene=ANLN
VEGFA	Vascular Endothelial Growth Factor A	Protein Coding	P15692	53	GC06P043770	23.9623184	https://www.genecards.org/cgi-bin/carddisp.pl?gene=VEGFA
RELN	Reelin	Protein Coding	P78509	48	GC07M103471	23.6615829	https://www.genecards.org/cgi-bin/carddisp.pl?gene=RELN
MYH9	Myosin Heavy Chain 9	Protein Coding	P35579	51	GC22M036281	23.3555946	https://www.genecards.org/cgi-bin/carddisp.pl?gene=MYH9
CHL1	Cell Adhesion Molecule L1 Like	Protein Coding	O00533	47	GC03P000213	23.2727413	https://www.genecards.org/cgi-bin/carddisp.pl?gene=CHL1
KRT6C	Keratin 6C	Protein Coding	P48668	42	GC12M052468	23.2426453	https://www.genecards.org/cgi-bin/carddisp.pl?gene=KRT6C
EGFR	Epidermal Growth Factor Receptor	Protein Coding	P00533	58	GC07P055019	23.1548805	https://www.genecards.org/cgi-bin/carddisp.pl?gene=EGFR
EZR	Ezrin	Protein Coding	P15311	50	GC06M158765	23.1438637	https://www.genecards.org/cgi-bin/carddisp.pl?gene=EZR

CTNNA1	Catenin Alpha 1	Protein Coding	P35221	50	GC05P138653	22.7066345	https://www.genecards.org/cgi-bin/carddisp.pl?gene=CTNNA1
GIT1	GIT ArfGAP 1	Protein Coding	Q9Y2X7	46	GC17M029573	22.7034035	https://www.genecards.org/cgi-bin/carddisp.pl?gene=GIT1
JUP	Junction Plakoglobin	Protein Coding	P14923	52	GC17M041754	22.614727	https://www.genecards.org/cgi-bin/carddisp.pl?gene=JUP
TGFB1	Transforming Growth Factor Beta 1	Protein Coding	P01137	56	GC19M041301	22.5771198	https://www.genecards.org/cgi-bin/carddisp.pl?gene=TGFB1
ITGA6	Integrin Subunit Alpha 6	Protein Coding	P23229	53	GC02P172427	22.5215569	https://www.genecards.org/cgi-bin/carddisp.pl?gene=ITGA6
DSCAM	DS Cell Adhesion Molecule	Protein Coding	O60469	45	GC21M040010	22.4881382	https://www.genecards.org/cgi-bin/carddisp.pl?gene=DSCAM
PIK3CA	Phosphatidylinositol-4,5-Bisphosphate 3-Kinase Catalytic Subunit Alpha	Protein Coding	P42336	55	GC03P179148	22.4577618	https://www.genecards.org/cgi-bin/carddisp.pl?gene=PIK3CA
IL6	Interleukin 6	Protein Coding	P05231	54	GC07P022725	22.4123268	https://www.genecards.org/cgi-bin/carddisp.pl?gene=IL6
PAK1	P21 (RAC1) Activated Kinase 1	Protein Coding	Q13153	54	GC11M117322	22.3487854	https://www.genecards.org/cgi-bin/carddisp.pl?gene=PAK1
BCAM	Basal Cell Adhesion Molecule (Lutheran Blood Group)	Protein Coding	P50895	44	GC19P096168	22.2954388	https://www.genecards.org/cgi-bin/carddisp.pl?gene=BCAM
LIMS1	LIM Zinc Finger Domain Containing 1	Protein Coding	P48059	45	GC02P108534	22.2805481	https://www.genecards.org/cgi-bin/carddisp.pl?gene=LIMS1
CRK	CRK Proto-Oncogene, Adaptor Protein	Protein Coding	P46108	48	GC17M001420	22.241333	https://www.genecards.org/cgi-bin/carddisp.pl?gene=CRK
ADGRE5	Adhesion G Protein-Coupled Receptor E5	Protein Coding	P48960	47	GC19P095438	22.2224216	https://www.genecards.org/cgi-bin/carddisp.pl?gene=ADGRE5
KCNQ2	Potassium Voltage-Gated Channel Subfamily Q Member 2	Protein Coding	O43526	51	GC20M063400	22.2052155	https://www.genecards.org/cgi-bin/carddisp.pl?gene=KCNQ2
COL4A5	Collagen Type IV Alpha 5 Chain	Protein Coding	P29400	47	GC0XP108439	22.0854816	https://www.genecards.org/cgi-bin/carddisp.pl?gene=COL4A5
NECTIN1	Nectin Cell Adhesion Molecule 1	Protein Coding	Q15223	50	GC11M120809	21.9359913	https://www.genecards.org/cgi-bin/carddisp.pl?gene=NECTIN1
AXDND1	Axonemal Dynein Light Chain Domain Containing 1	Protein Coding	Q5T1B0	36	GC01P179366	21.8061562	https://www.genecards.org/cgi-bin/carddisp.pl?gene=AXDND1
MAPK3	Mitogen-Activated Protein Kinase 3	Protein Coding	P27361	52	GC16M042426	21.6488571	https://www.genecards.org/cgi-bin/carddisp.pl?gene=MAPK3
COL4A3	Collagen Type IV Alpha 3 Chain	Protein Coding	Q01955	50	GC02P227164	21.580883	https://www.genecards.org/cgi-bin/carddisp.pl?gene=COL4A3
ARHGAP26	Rho GTPase Activating Protein 26	Protein Coding	Q9UNA1	50	GC05P142770	21.4713669	https://www.genecards.org/cgi-bin/carddisp.pl?gene=ARHGAP26
ITGAX	Integrin Subunit Alpha X	Protein Coding	P20702	50	GC16P060885	21.3919163	https://www.genecards.org/cgi-bin/carddisp.pl?gene=ITGAX
PVR	PVR Cell Adhesion Molecule	Protein Coding	P15151	48	GC19P096159	21.2682495	https://www.genecards.org/cgi-bin/carddisp.pl?gene=PVR
ADGRV1	Adhesion G Protein-Coupled Receptor V1	Protein Coding	Q8WXG9	46	GC05P090529	21.050396	https://www.genecards.org/cgi-bin/carddisp.pl?gene=ADGRV1
GRB2	Growth Factor Receptor Bound Protein 2	Protein Coding	P62993	50	GC17M075318	21.0322609	https://www.genecards.org/cgi-bin/carddisp.pl?gene=GRB2
LAMA5	Laminin Subunit Alpha 5	Protein Coding	O15230	48	GC20M062307	20.8934765	https://www.genecards.org/cgi-bin/carddisp.pl?gene=LAMA5

TGFB1I1	Transforming Growth Factor Beta 1 Induced Transcript 1	Protein Coding	O43294	46	GC16P060891	20.8171959	https://www.genecards.org/cgi-bin/carddisp.pl?gene=TGFB1I1
IL1B	Interleukin 1 Beta	Protein Coding	P01584	51	GC02M112829	20.6575527	https://www.genecards.org/cgi-bin/carddisp.pl?gene=IL1B
CTNND1	Catenin Delta 1	Protein Coding	O60716	48	GC11P058457	20.5905323	https://www.genecards.org/cgi-bin/carddisp.pl?gene=CTNND1
PLAUR	Plasminogen Activator, Urokinase Receptor	Protein Coding	Q03405	48	GC19M043646	20.5279732	https://www.genecards.org/cgi-bin/carddisp.pl?gene=PLAUR
ROCK1	Rho Associated Coiled-Coil Containing Protein Kinase 1	Protein Coding	Q13464	53	GC18M031986	20.5080414	https://www.genecards.org/cgi-bin/carddisp.pl?gene=ROCK1
ITGB7	Integrin Subunit Beta 7	Protein Coding	P26010	49	GC12M053191	20.4284286	https://www.genecards.org/cgi-bin/carddisp.pl?gene=ITGB7
CDC42	Cell Division Cycle 42	Protein Coding	P60953	54	GC01P022052	20.3594856	https://www.genecards.org/cgi-bin/carddisp.pl?gene=CDC42
ESAM	Endothelial Cell Adhesion Molecule	Protein Coding	Q96AP7	45	GC11M124752	20.3225727	https://www.genecards.org/cgi-bin/carddisp.pl?gene=ESAM
PCDH19	Protocadherin 19	Protein Coding	Q8TAB3	46	GC0XM100291	20.1127586	https://www.genecards.org/cgi-bin/carddisp.pl?gene=PCDH19
NCAM2	Neural Cell Adhesion Molecule 2	Protein Coding	O15394	44	GC21P020998	20.1064262	https://www.genecards.org/cgi-bin/carddisp.pl?gene=NCAM2
NUP93	Nucleoporin 93	Protein Coding	Q8N1F7	45	GC16P061470	20.0560722	https://www.genecards.org/cgi-bin/carddisp.pl?gene=NUP93
SLC12A5	Solute Carrier Family 12 Member 5	Protein Coding	Q9H2X9	52	GC20P046021	19.9954128	https://www.genecards.org/cgi-bin/carddisp.pl?gene=SLC12A5
CTTN	Cortactin	Protein Coding	Q14247	47	GC11P070398	19.9832726	https://www.genecards.org/cgi-bin/carddisp.pl?gene=CTTN
SHC1	SHC Adaptor Protein 1	Protein Coding	P29353	47	GC01M154962	19.9636955	https://www.genecards.org/cgi-bin/carddisp.pl?gene=SHC1
NEDD9	Neural Precursor Cell Expressed, Developmentally Down-Regulated 9	Protein Coding	Q14511	46	GC06M011183	19.8928814	https://www.genecards.org/cgi-bin/carddisp.pl?gene=NEDD9
NECTIN2	Nectin Cell Adhesion Molecule 2	Protein Coding	Q92692	48	GC19P096169	19.8707962	https://www.genecards.org/cgi-bin/carddisp.pl?gene=NECTIN2
ITGB5	Integrin Subunit Beta 5	Protein Coding	P18084	50	GC03M124761	19.8370609	https://www.genecards.org/cgi-bin/carddisp.pl?gene=ITGB5
CADM3	Cell Adhesion Molecule 3	Protein Coding	Q8N126	46	GC01P159187	19.7701511	https://www.genecards.org/cgi-bin/carddisp.pl?gene=CADM3
THBS1	Thrombospondin 1	Protein Coding	P07996	49	GC15P039581	19.6396694	https://www.genecards.org/cgi-bin/carddisp.pl?gene=THBS1
PIK3CG	Phosphatidylinositol-4,5-Bisphosphate 3-Kinase Catalytic Subunit Gamma	Protein Coding	P48736	52	GC07P106865	19.6316986	https://www.genecards.org/cgi-bin/carddisp.pl?gene=PIK3CG
KIRREL3	Kirre Like Nephrin Family Adhesion Molecule 3	Protein Coding	Q8IZU9	46	GC11M126423	19.5647011	https://www.genecards.org/cgi-bin/carddisp.pl?gene=KIRREL3
ITGA2B	Integrin Subunit Alpha 2b	Protein Coding	P08514	55	GC17M068275	19.5612526	https://www.genecards.org/cgi-bin/carddisp.pl?gene=ITGA2B
CEACAM3	CEA Cell Adhesion Molecule 3	Protein Coding	P40198	46	GC19P041796	19.4510612	https://www.genecards.org/cgi-bin/carddisp.pl?gene=CEACAM3
KIRREL1	Kirre Like Nephrin Family Adhesion Molecule 1	Protein Coding	Q96J84	44	GC01P159018	19.3630524	https://www.genecards.org/cgi-bin/carddisp.pl?gene=KIRREL1
ARHGEF7	Rho Guanine Nucleotide Exchange Factor 7	Protein Coding	Q14155	46	GC13P111114	19.3393364	https://www.genecards.org/cgi-bin/carddisp.pl?gene=ARHGEF7

FLNA	Filamin A	Protein Coding	P21333	52	GC0XM154348	19.3325539	https://www.genecards.org/cgi-bin/carddisp.pl?gene=FLNA
PIK3R1	Phosphoinositide-3-Kinase Regulatory Subunit 1	Protein Coding	P27986	55	GC05P068215	19.289917	https://www.genecards.org/cgi-bin/carddisp.pl?gene=PIK3R1
VIM	Vimentin	Protein Coding	P08670	54	GC10P017227	19.2610607	https://www.genecards.org/cgi-bin/carddisp.pl?gene=VIM
CADM2	Cell Adhesion Molecule 2	Protein Coding	Q8N3J6	43	GC03P084969	19.2385941	https://www.genecards.org/cgi-bin/carddisp.pl?gene=CADM2
LPXN	Leupaxin	Protein Coding	O60711	44	GC11M116566	19.0243931	https://www.genecards.org/cgi-bin/carddisp.pl?gene=LPXN
ACTN1	Actinin Alpha 1	Protein Coding	P12814	52	GC14M068874	18.9612637	https://www.genecards.org/cgi-bin/carddisp.pl?gene=ACTN1
FBLIM1	Filamin Binding LIM Protein 1	Protein Coding	Q8WUP2	41	GC01P015756	18.9095879	https://www.genecards.org/cgi-bin/carddisp.pl?gene=FBLIM1
CD36	CD36 Molecule	Protein Coding	P16671	53	GC07P080369	18.8981762	https://www.genecards.org/cgi-bin/carddisp.pl?gene=CD36
MSN	Moesin	Protein Coding	P26038	51	GC0XP065588	18.7823181	https://www.genecards.org/cgi-bin/carddisp.pl?gene=MSN
ROCK2	Rho Associated Coiled-Coil Containing Protein Kinase 2	Protein Coding	O75116	50	GC02M011409	18.6703529	https://www.genecards.org/cgi-bin/carddisp.pl?gene=ROCK2
ITGA1	Integrin Subunit Alpha 1	Protein Coding	P56199	47	GC05P052788	18.6664581	https://www.genecards.org/cgi-bin/carddisp.pl?gene=ITGA1
PIP5K1C	Phosphatidylinositol-4-Phosphate 5-Kinase Type 1 Gamma	Protein Coding	O60331	52	GC19M016664	18.5665741	https://www.genecards.org/cgi-bin/carddisp.pl?gene=PIP5K1C
SLC25A22	Solute Carrier Family 25 Member 22	Protein Coding	Q9H936	44	GC11M009580	18.4741554	https://www.genecards.org/cgi-bin/carddisp.pl?gene=SLC25A22
CDKL5	Cyclin Dependent Kinase Like 5	Protein Coding	O76039	47	GC0XP018425	18.3972664	https://www.genecards.org/cgi-bin/carddisp.pl?gene=CDKL5
CAV1	Caveolin 1	Protein Coding	Q03135	52	GC07P116524	18.2902889	https://www.genecards.org/cgi-bin/carddisp.pl?gene=CAV1
HEPACAM	Hepatic And Glial Cell Adhesion Molecule	Protein Coding	Q14CZ8	44	GC11M124919	18.2100658	https://www.genecards.org/cgi-bin/carddisp.pl?gene=HEPACAM
NECTIN4	Nectin Cell Adhesion Molecule 4	Protein Coding	Q96NY8	48	GC01M161089	18.1845741	https://www.genecards.org/cgi-bin/carddisp.pl?gene=NECTIN4
NECTIN3	Nectin Cell Adhesion Molecule 3	Protein Coding	Q9NQS3	44	GC03P111071	18.180521	https://www.genecards.org/cgi-bin/carddisp.pl?gene=NECTIN3
ADGRE2	Adhesion G Protein-Coupled Receptor E2	Protein Coding	Q9UHX3	46	GC19M017540	18.1584702	https://www.genecards.org/cgi-bin/carddisp.pl?gene=ADGRE2
CNTN2	Contactin 2	Protein Coding	Q02246	49	GC01P205043	18.0967598	https://www.genecards.org/cgi-bin/carddisp.pl?gene=CNTN2
CEACAM4	CEA Cell Adhesion Molecule 4	Protein Coding	O75871	38	GC19M085578	17.8864155	https://www.genecards.org/cgi-bin/carddisp.pl?gene=CEACAM4
CXADR	CXADR Ig-Like Cell Adhesion Molecule	Protein Coding	P78310	47	GC21P017513	17.6599312	https://www.genecards.org/cgi-bin/carddisp.pl?gene=CXADR
CDH5	Cadherin 5	Protein Coding	P33151	50	GC16P066366	17.5856094	https://www.genecards.org/cgi-bin/carddisp.pl?gene=CDH5
PTPRA	Protein Tyrosine Phosphatase Receptor Type A	Protein Coding	P18433	50	GC20P002864	17.5549088	https://www.genecards.org/cgi-bin/carddisp.pl?gene=PTPRA
SELPLG	Selectin P Ligand	Protein Coding	Q14242	47	GC12M108621	17.5203762	https://www.genecards.org/cgi-bin/carddisp.pl?gene=SELPLG

IFNG	Interferon Gamma	Protein Coding	P01579	54	GC12M068154	17.5080681	https://www.genecards.org/cgi-bin/carddisp.pl?gene=IFNG
PRKCA	Protein Kinase C Alpha	Protein Coding	P17252	54	GC17P066302	17.4780102	https://www.genecards.org/cgi-bin/carddisp.pl?gene=PRKCA
F11R	F11 Receptor	Protein Coding	Q9Y624	46	GC01M160995	17.4358006	https://www.genecards.org/cgi-bin/carddisp.pl?gene=F11R
ADGRB1	Adhesion G Protein-Coupled Receptor B1	Protein Coding	O14514	41	GC08P142449	17.3879642	https://www.genecards.org/cgi-bin/carddisp.pl?gene=ADGRB1
DSP	Desmoplakin	Protein Coding	P15924	54	GC06P007541	17.3126125	https://www.genecards.org/cgi-bin/carddisp.pl?gene=DSP
SDK1	Sidekick Cell Adhesion Molecule 1	Protein Coding	Q7Z5N4	41	GC07P008864	17.265913	https://www.genecards.org/cgi-bin/carddisp.pl?gene=SDK1
SPP1	Secreted Phosphoprotein 1	Protein Coding	P10451	49	GC04P087975	17.2390804	https://www.genecards.org/cgi-bin/carddisp.pl?gene=SPP1
SPAM1	Sperm Adhesion Molecule 1	Protein Coding	P38567	45	GC07P123925	17.1508007	https://www.genecards.org/cgi-bin/carddisp.pl?gene=SPAM1
APBB1IP	Amyloid Beta Precursor Protein Binding Family B Member 1 Interacting Protein	Protein Coding	Q7Z5R6	43	GC10P026462	17.1391315	https://www.genecards.org/cgi-bin/carddisp.pl?gene=APBB1IP
FAT1	FAT Atypical Cadherin 1	Protein Coding	Q14517	44	GC04M186587	17.0451527	https://www.genecards.org/cgi-bin/carddisp.pl?gene=FAT1
PLCB1	Phospholipase C Beta 1	Protein Coding	Q9NQ66	51	GC20P008061	17.0268269	https://www.genecards.org/cgi-bin/carddisp.pl?gene=PLCB1
CEACAM7	CEA Cell Adhesion Molecule 7	Protein Coding	Q14002	41	GC19M041673	17.0040913	https://www.genecards.org/cgi-bin/carddisp.pl?gene=CEACAM7
CXCL8	C-X-C Motif Chemokine Ligand 8	Protein Coding	P10145	47	GC04P073740	16.9734516	https://www.genecards.org/cgi-bin/carddisp.pl?gene=CXCL8
CD151	CD151 Molecule (Raph Blood Group)	Protein Coding	P48509	47	GC11P005728	16.9656029	https://www.genecards.org/cgi-bin/carddisp.pl?gene=CD151
ADGRD1	Adhesion G Protein-Coupled Receptor D1	Protein Coding	Q6QNK2	43	GC12P130953	16.9085293	https://www.genecards.org/cgi-bin/carddisp.pl?gene=ADGRD1
MAPK8	Mitogen-Activated Protein Kinase 8	Protein Coding	P45983	53	GC10P048306	16.8791637	https://www.genecards.org/cgi-bin/carddisp.pl?gene=MAPK8
CDON	Cell Adhesion Associated, Oncogene Regulated	Protein Coding	Q4KMG0	50	GC11M125955	16.7819958	https://www.genecards.org/cgi-bin/carddisp.pl?gene=CDON
NCK1	NCK Adaptor Protein 1	Protein Coding	P16333	47	GC03P136862	16.7645931	https://www.genecards.org/cgi-bin/carddisp.pl?gene=NCK1
PARVB	Parvin Beta	Protein Coding	Q9HBI1	44	GC22P043999	16.6879368	https://www.genecards.org/cgi-bin/carddisp.pl?gene=PARVB
RAC2	Rac Family Small GTPase 2	Protein Coding	P15153	55	GC22M072640	16.64328	https://www.genecards.org/cgi-bin/carddisp.pl?gene=RAC2
ITGB1BP1	Integrin Subunit Beta 1 Binding Protein 1	Protein Coding	O14713	42	GC02M009391	16.6130524	https://www.genecards.org/cgi-bin/carddisp.pl?gene=ITGB1BP1
H19	H19 Imprinted Maternally Expressed Transcript	RNA Gene		28	GC11M001995	16.5690746	https://www.genecards.org/cgi-bin/carddisp.pl?gene=H19
RHOD	Ras Homolog Family Member D	Protein Coding	O00212	41	GC11P082069	16.4864311	https://www.genecards.org/cgi-bin/carddisp.pl?gene=RHOD
ADGRL1	Adhesion G Protein-Coupled Receptor L1	Protein Coding	O94910	45	GC19M014147	16.3983612	https://www.genecards.org/cgi-bin/carddisp.pl?gene=ADGRL1
SCARB2	Scavenger Receptor Class B Member 2	Protein Coding	Q14108	49	GC04M076158	16.2903385	https://www.genecards.org/cgi-bin/carddisp.pl?gene=SCARB2

ITGB6	Integrin Subunit Beta 6	Protein Coding	P18564	52	GC02M160099	16.2859612	https://www.genecards.org/cgi-bin/carddisp.pl?gene=ITGB6
SPTAN1	Spectrin Alpha, Non-Erythrocytic 1	Protein Coding	Q13813	52	GC09P128552	16.2658691	https://www.genecards.org/cgi-bin/carddisp.pl?gene=SPTAN1
ADGRG5	Adhesion G Protein-Coupled Receptor G5	Protein Coding	Q8IZF4	40	GC16P057529	16.2149124	https://www.genecards.org/cgi-bin/carddisp.pl?gene=ADGRG5
RDX	Radixin	Protein Coding	P35241	51	GC11M109864	16.1584949	https://www.genecards.org/cgi-bin/carddisp.pl?gene=RDX
PTPN11	Protein Tyrosine Phosphatase Non-Receptor Type 11	Protein Coding	Q06124	57	GC12P112418	16.1513615	https://www.genecards.org/cgi-bin/carddisp.pl?gene=PTPN11
RAP1A	RAP1A, Member Of RAS Oncogene Family	Protein Coding	P62834	48	GC01P111542	16.1236343	https://www.genecards.org/cgi-bin/carddisp.pl?gene=RAP1A
PLCG1	Phospholipase C Gamma 1	Protein Coding	P19174	51	GC20P041136	16.0845089	https://www.genecards.org/cgi-bin/carddisp.pl?gene=PLCG1
PIGA	Phosphatidylinositol Glycan Anchor Biosynthesis Class A	Protein Coding	P37287	47	GC0XM015319	16.0504398	https://www.genecards.org/cgi-bin/carddisp.pl?gene=PIGA
PEAK1	Pseudopodium Enriched Atypical Kinase 1	Protein Coding	Q9H792	39	GC15M077100	16.0220947	https://www.genecards.org/cgi-bin/carddisp.pl?gene=PEAK1
EYA1	EYA Transcriptional Coactivator And Phosphatase 1	Protein Coding	Q99502	47	GC08M071210	16.0126553	https://www.genecards.org/cgi-bin/carddisp.pl?gene=EYA1
LGI1	Leucine Rich Glioma Inactivated 1	Protein Coding	O95970	48	GC10P093757	15.9514256	https://www.genecards.org/cgi-bin/carddisp.pl?gene=LGI1
MECP2	Methyl-CpG Binding Protein 2	Protein Coding	P51608	50	GC0XM154021	15.9434586	https://www.genecards.org/cgi-bin/carddisp.pl?gene=MECP2
DSPP	Dentin Sialophosphoprotein	Protein Coding	Q9NZW4	43	GC04P087608	15.8947039	https://www.genecards.org/cgi-bin/carddisp.pl?gene=DSPP
SRPX2	Sushi Repeat Containing Protein X-Linked 2	Protein Coding	O60687	44	GC0XP100648	15.8439589	https://www.genecards.org/cgi-bin/carddisp.pl?gene=SRPX2
ADGRF5	Adhesion G Protein-Coupled Receptor F5	Protein Coding	Q8IZF2	44	GC06M046852	15.8359718	https://www.genecards.org/cgi-bin/carddisp.pl?gene=ADGRF5
EPHA2	EPH Receptor A2	Protein Coding	P29317	56	GC01M016124	15.6921196	https://www.genecards.org/cgi-bin/carddisp.pl?gene=EPHA2
CCL2	C-C Motif Chemokine Ligand 2	Protein Coding	P13500	52	GC17P034255	15.6849136	https://www.genecards.org/cgi-bin/carddisp.pl?gene=CCL2
CADM4	Cell Adhesion Molecule 4	Protein Coding	Q8NFZ8	41	GC19M043622	15.6795406	https://www.genecards.org/cgi-bin/carddisp.pl?gene=CADM4
ABCC8	ATP Binding Cassette Subfamily C Member 8	Protein Coding	Q09428	50	GC11M017392	15.6403847	https://www.genecards.org/cgi-bin/carddisp.pl?gene=ABCC8
LAMA3	Laminin Subunit Alpha 3	Protein Coding	Q16787	49	GC18P023689	15.6240082	https://www.genecards.org/cgi-bin/carddisp.pl?gene=LAMA3
LOC126860216	BRD4-Independent Group 4 Enhancer GRCh37_chr7:147868766-147869965	Functional Element		3	GC07P154773	15.614234	https://www.genecards.org/cgi-bin/carddisp.pl?gene=LOC126860216
EMP2	Epithelial Membrane Protein 2	Protein Coding	P54851	46	GC16M014429	15.5864229	https://www.genecards.org/cgi-bin/carddisp.pl?gene=EMP2
TLN2	Talin 2	Protein Coding	Q9Y4G6	44	GC15P062390	15.5684738	https://www.genecards.org/cgi-bin/carddisp.pl?gene=TLN2
MMP2	Matrix Metallopeptidase 2	Protein Coding	P08253	56	GC16P061423	15.5417366	https://www.genecards.org/cgi-bin/carddisp.pl?gene=MMP2
AKT3	AKT Serine/Threonine Kinase 3	Protein Coding	Q9Y243	58	GC01M243488	15.5385284	https://www.genecards.org/cgi-bin/carddisp.pl?gene=AKT3

ITGAD	Integrin Subunit Alpha D	Protein Coding	Q13349	42	GC16P060888	15.4713011	https://www.genecards.org/cgi-bin/carddisp.pl?gene=ITGAD
MPZ	Myelin Protein Zero	Protein Coding	P25189	48	GC01M161304	15.4179516	https://www.genecards.org/cgi-bin/carddisp.pl?gene=MPZ
GABRG2	Gamma-Aminobutyric Acid Type A Receptor Subunit Gamma2	Protein Coding	P18507	52	GC05P162000	15.3697119	https://www.genecards.org/cgi-bin/carddisp.pl?gene=GABRG2
COQ8B	Coenzyme Q8B	Protein Coding	Q96D53	43	GC19M085545	15.3458223	https://www.genecards.org/cgi-bin/carddisp.pl?gene=COQ8B
MMP9	Matrix Metallopeptidase 9	Protein Coding	P14780	57	GC20P046008	15.3276129	https://www.genecards.org/cgi-bin/carddisp.pl?gene=MMP9
SDCCAG8	SHH Signaling And Ciliogenesis Regulator SDCCAG8	Protein Coding	Q86SQ7	45	GC01P243255	15.2187071	https://www.genecards.org/cgi-bin/carddisp.pl?gene=SDCCAG8
MUC1	Mucin 1, Cell Surface Associated	Protein Coding	P15941	52	GC01M155185	15.1868486	https://www.genecards.org/cgi-bin/carddisp.pl?gene=MUC1
ADGRG6	Adhesion G Protein-Coupled Receptor G6	Protein Coding	Q86SQ4	46	GC06P142301	15.1700859	https://www.genecards.org/cgi-bin/carddisp.pl?gene=ADGRG6
ADGRL2	Adhesion G Protein-Coupled Receptor L2	Protein Coding	O95490	44	GC01P081306	15.079917	https://www.genecards.org/cgi-bin/carddisp.pl?gene=ADGRL2
GRB7	Growth Factor Receptor Bound Protein 7	Protein Coding	Q14451	47	GC17P093563	15.0654144	https://www.genecards.org/cgi-bin/carddisp.pl?gene=GRB7
ERBB2	Erb-B2 Receptor Tyrosine Kinase 2	Protein Coding	P04626	58	GC17P039687	15.0566063	https://www.genecards.org/cgi-bin/carddisp.pl?gene=ERBB2
ADGRB2	Adhesion G Protein-Coupled Receptor B2	Protein Coding	O60241	44	GC01M031727	15.0117674	https://www.genecards.org/cgi-bin/carddisp.pl?gene=ADGRB2
AOC3	Amine Oxidase Copper Containing 3	Protein Coding	Q16853	49	GC17P042851	15.0017204	https://www.genecards.org/cgi-bin/carddisp.pl?gene=AOC3
CD209	CD209 Molecule	Protein Coding	Q9NNX6	47	GC19M007739	14.9991865	https://www.genecards.org/cgi-bin/carddisp.pl?gene=CD209
AMIGO1	Adhesion Molecule With Ig Like Domain 1	Protein Coding	Q86WK6	40	GC01M109504	14.9918442	https://www.genecards.org/cgi-bin/carddisp.pl?gene=AMIGO1
GIT2	GIT ArfGAP 2	Protein Coding	Q14161	45	GC12M109929	14.9889221	https://www.genecards.org/cgi-bin/carddisp.pl?gene=GIT2
ADGRA1	Adhesion G Protein-Coupled Receptor A1	Protein Coding	Q86SQ6	41	GC10P133087	14.9513884	https://www.genecards.org/cgi-bin/carddisp.pl?gene=ADGRA1
SCN1B	Sodium Voltage-Gated Channel Beta Subunit 1	Protein Coding	Q07699	49	GC19P035030	14.8512125	https://www.genecards.org/cgi-bin/carddisp.pl?gene=SCN1B
TNC	Tenascin C	Protein Coding	P24821	53	GC09M115019	14.8406191	https://www.genecards.org/cgi-bin/carddisp.pl?gene=TNC
CEACAM19	CEA Cell Adhesion Molecule 19	Protein Coding	Q7Z692	34	GC19P096158	14.8106422	https://www.genecards.org/cgi-bin/carddisp.pl?gene=CEACAM19
KDR	Kinase Insert Domain Receptor	Protein Coding	P35968	57	GC04M055078	14.7181683	https://www.genecards.org/cgi-bin/carddisp.pl?gene=KDR
EGF	Epidermal Growth Factor	Protein Coding	P01133	54	GC04P109912	14.7162724	https://www.genecards.org/cgi-bin/carddisp.pl?gene=EGF
PDPK1	3-Phosphoinositide Dependent Protein Kinase 1	Protein Coding	O15530	52	GC16P002537	14.5960045	https://www.genecards.org/cgi-bin/carddisp.pl?gene=PDPK1
TNS1	Tensin 1	Protein Coding	Q9HBL0	45	GC02M217799	14.5913849	https://www.genecards.org/cgi-bin/carddisp.pl?gene=TNS1
PAK4	P21 (RAC1) Activated Kinase 4	Protein Coding	O96013	52	GC19P039125	14.5673199	https://www.genecards.org/cgi-bin/carddisp.pl?gene=PAK4

CDH3	Cadherin 3	Protein Coding	P22223	51	GC16P069005	14.5668468	https://www.genecards.org/cgi-bin/carddisp.pl?gene=CDH3
CFH	Complement Factor H	Protein Coding	P08603	51	GC01P196621	14.5249844	https://www.genecards.org/cgi-bin/carddisp.pl?gene=CFH
DSG1-AS1	DSG1 Antisense RNA 1	RNA Gene		16	GC18M032112	14.505352	https://www.genecards.org/cgi-bin/carddisp.pl?gene=DSG1-AS1
LPP	LIM Domain Containing Preferred Translocation Partner In Lipoma	Protein Coding	Q93052	48	GC03P188153	14.4671431	https://www.genecards.org/cgi-bin/carddisp.pl?gene=LPP
ADGRF1	Adhesion G Protein-Coupled Receptor F1	Protein Coding	Q5T601	39	GC06M046997	14.4246454	https://www.genecards.org/cgi-bin/carddisp.pl?gene=ADGRF1
MAP2K1	Mitogen-Activated Protein Kinase Kinase 1	Protein Coding	Q02750	58	GC15P066386	14.3890076	https://www.genecards.org/cgi-bin/carddisp.pl?gene=MAP2K1
LAMC2	Laminin Subunit Gamma 2	Protein Coding	Q13753	51	GC01P183186	14.3753023	https://www.genecards.org/cgi-bin/carddisp.pl?gene=LAMC2
ADGRG4	Adhesion G Protein-Coupled Receptor G4	Protein Coding	Q8IZF6	35	GC0XP136300	14.354928	https://www.genecards.org/cgi-bin/carddisp.pl?gene=ADGRG4
SYNPO2	Synaptopodin 2	Protein Coding	Q9UMS6	41	GC04P118850	14.3395977	https://www.genecards.org/cgi-bin/carddisp.pl?gene=SYNPO2
BOC	BOC Cell Adhesion Associated, Oncogene Regulated	Protein Coding	Q9BWV1	45	GC03P113347	14.3339386	https://www.genecards.org/cgi-bin/carddisp.pl?gene=BOC
PTPN12	Protein Tyrosine Phosphatase Non-Receptor Type 12	Protein Coding	Q05209	51	GC07P077537	14.2738743	https://www.genecards.org/cgi-bin/carddisp.pl?gene=PTPN12
ADGRG3	Adhesion G Protein-Coupled Receptor G3	Protein Coding	Q86Y34	42	GC16P061521	14.2703323	https://www.genecards.org/cgi-bin/carddisp.pl?gene=ADGRG3
ADGRB3	Adhesion G Protein-Coupled Receptor B3	Protein Coding	O60242	43	GC06P068635	14.2173738	https://www.genecards.org/cgi-bin/carddisp.pl?gene=ADGRB3
ARHGAP35	Rho GTPase Activating Protein 35	Protein Coding	Q9NRY4	44	GC19P046860	14.1890335	https://www.genecards.org/cgi-bin/carddisp.pl?gene=ARHGAP35
ABL1	ABL Proto-Oncogene 1, Non-Receptor Tyrosine Kinase	Protein Coding	P00519	56	GC09P130713	14.1881485	https://www.genecards.org/cgi-bin/carddisp.pl?gene=ABL1
TOR1A	Torsin Family 1 Member A	Protein Coding	O14656	50	GC09M129812	14.186594	https://www.genecards.org/cgi-bin/carddisp.pl?gene=TOR1A
CSF3	Colony Stimulating Factor 3	Protein Coding	P09919	44	GC17P040015	14.186038	https://www.genecards.org/cgi-bin/carddisp.pl?gene=CSF3
ENG	Endoglin	Protein Coding	P17813	52	GC09M127815	14.1760559	https://www.genecards.org/cgi-bin/carddisp.pl?gene=ENG
ITGA7	Integrin Subunit Alpha 7	Protein Coding	Q13683	50	GC12M055684	14.1452427	https://www.genecards.org/cgi-bin/carddisp.pl?gene=ITGA7
NFASC	Neurofascin	Protein Coding	O94856	47	GC01P204828	14.0468369	https://www.genecards.org/cgi-bin/carddisp.pl?gene=NFASC
CXCL12	C-X-C Motif Chemokine Ligand 12	Protein Coding	P48061	48	GC10M044370	14.0468225	https://www.genecards.org/cgi-bin/carddisp.pl?gene=CXCL12
TMX2-CTNND1	TMX2-CTNND1 Readthrough (NMD Candidate)	RNA Gene		17	GC11P057712	14.0017986	https://www.genecards.org/cgi-bin/carddisp.pl?gene=TMX2-CTNND1
COL4A1	Collagen Type IV Alpha 1 Chain	Protein Coding	P02462	51	GC13M110148	13.9275475	https://www.genecards.org/cgi-bin/carddisp.pl?gene=COL4A1
DAG1	Dystroglycan 1	Protein Coding	Q14118	50	GC03P054954	13.9187412	https://www.genecards.org/cgi-bin/carddisp.pl?gene=DAG1
ADGRA2	Adhesion G Protein-Coupled Receptor A2	Protein Coding	Q96PE1	42	GC08P037851	13.8392057	https://www.genecards.org/cgi-bin/carddisp.pl?gene=ADGRA2

CD4	CD4 Molecule	Protein Coding	P01730	53	GC12P006786	13.8289614	https://www.genecards.org/cgi-bin/carddisp.pl?gene=CD4
MAPK10	Mitogen-Activated Protein Kinase 10	Protein Coding	P53779	52	GC04M085990	13.8225136	https://www.genecards.org/cgi-bin/carddisp.pl?gene=MAPK10
JUN	Jun Proto-Oncogene, AP-1 Transcription Factor Subunit	Protein Coding	P05412	53	GC01M058780	13.8120394	https://www.genecards.org/cgi-bin/carddisp.pl?gene=JUN
BRAF	B-Raf Proto-Oncogene, Serine/Threonine Kinase	Protein Coding	P15056	57	GC07M140757	13.8108845	https://www.genecards.org/cgi-bin/carddisp.pl?gene=BRAF
GP6	Glycoprotein VI Platelet	Protein Coding	Q9HCN6	49	GC19M055013	13.8041668	https://www.genecards.org/cgi-bin/carddisp.pl?gene=GP6
TEK	TEK Receptor Tyrosine Kinase	Protein Coding	Q02763	55	GC09P027109	13.7329254	https://www.genecards.org/cgi-bin/carddisp.pl?gene=TEK
CD58	CD58 Molecule	Protein Coding	P19256	44	GC01M116514	13.7137833	https://www.genecards.org/cgi-bin/carddisp.pl?gene=CD58
SNAP25	Synaptosome Associated Protein 25	Protein Coding	P60880	53	GC20P010216	13.5866385	https://www.genecards.org/cgi-bin/carddisp.pl?gene=SNAP25
POSTN	Periostin	Protein Coding	Q15063	46	GC13M037562	13.536768	https://www.genecards.org/cgi-bin/carddisp.pl?gene=POSTN
ADGRL4	Adhesion G Protein-Coupled Receptor L4	Protein Coding	Q9HBW9	41	GC01M078889	13.4654293	https://www.genecards.org/cgi-bin/carddisp.pl?gene=ADGRL4
PLAU	Plasminogen Activator, Urokinase	Protein Coding	P00749	55	GC10P073909	13.422924	https://www.genecards.org/cgi-bin/carddisp.pl?gene=PLAU
MAPK14	Mitogen-Activated Protein Kinase 14	Protein Coding	Q16539	54	GC06P119053	13.3928337	https://www.genecards.org/cgi-bin/carddisp.pl?gene=MAPK14
TRIP6	Thyroid Hormone Receptor Interactor 6	Protein Coding	Q15654	42	GC07P100867	13.2603664	https://www.genecards.org/cgi-bin/carddisp.pl?gene=TRIP6
PLEK	Pleckstrin	Protein Coding	P08567	44	GC02P068365	13.2360611	https://www.genecards.org/cgi-bin/carddisp.pl?gene=PLEK
SLC2A1	Solute Carrier Family 2 Member 1	Protein Coding	P11166	56	GC01M042925	13.2131729	https://www.genecards.org/cgi-bin/carddisp.pl?gene=SLC2A1
TLR4	Toll Like Receptor 4	Protein Coding	O00206	54	GC09P117704	13.1826344	https://www.genecards.org/cgi-bin/carddisp.pl?gene=TLR4
ACTB	Actin Beta	Protein Coding	P60709	53	GC07M005527	13.1419325	https://www.genecards.org/cgi-bin/carddisp.pl?gene=ACTB
PTPRO	Protein Tyrosine Phosphatase Receptor Type O	Protein Coding	Q16827	50	GC12P034014	13.055398	https://www.genecards.org/cgi-bin/carddisp.pl?gene=PTPRO
CD9	CD9 Molecule	Protein Coding	P21926	47	GC12P033698	13.0261183	https://www.genecards.org/cgi-bin/carddisp.pl?gene=CD9
PODXL	Podocalyxin Like	Protein Coding	O00592	47	GC07M131500	12.970808	https://www.genecards.org/cgi-bin/carddisp.pl?gene=PODXL
IGF1R	Insulin Like Growth Factor 1 Receptor	Protein Coding	P08069	58	GC15P098648	12.9683552	https://www.genecards.org/cgi-bin/carddisp.pl?gene=IGF1R
PLEC	Plectin	Protein Coding	Q15149	47	GC08M146323	12.965023	https://www.genecards.org/cgi-bin/carddisp.pl?gene=PLEC
THSD1	Thrombospondin Type 1 Domain Containing 1	Protein Coding	Q9NS62	44	GC13M052377	12.9459114	https://www.genecards.org/cgi-bin/carddisp.pl?gene=THSD1
RASSF5	Ras Association Domain Family Member 5	Protein Coding	Q8WWW0	42	GC01P206507	12.9380226	https://www.genecards.org/cgi-bin/carddisp.pl?gene=RASSF5
FLNC	Filamin C	Protein Coding	Q14315	48	GC07P128830	12.8981133	https://www.genecards.org/cgi-bin/carddisp.pl?gene=FLNC

WAS	WASP Actin Nucleation Promoting Factor	Protein Coding	P42768	51	GC0XP048676	12.8584204	https://www.genecards.org/cgi-bin/carddisp.pl?gene=WAS
CCND1	Cyclin D1	Protein Coding	P24385	57	GC11P069641	12.8582106	https://www.genecards.org/cgi-bin/carddisp.pl?gene=CCND1
ITGA9	Integrin Subunit Alpha 9	Protein Coding	Q13797	46	GC03P037468	12.8444424	https://www.genecards.org/cgi-bin/carddisp.pl?gene=ITGA9
ITGA8	Integrin Subunit Alpha 8	Protein Coding	P53708	48	GC10M015513	12.8191853	https://www.genecards.org/cgi-bin/carddisp.pl?gene=ITGA8
MMP14	Matrix Metallopeptidase 14	Protein Coding	P50281	54	GC14P041738	12.7604294	https://www.genecards.org/cgi-bin/carddisp.pl?gene=MMP14
ATP1A3	ATPase Na+/K+ Transporting Subunit Alpha 3	Protein Coding	P13637	52	GC19M041966	12.6967211	https://www.genecards.org/cgi-bin/carddisp.pl?gene=ATP1A3
MET	MET Proto-Oncogene, Receptor Tyrosine Kinase	Protein Coding	P08581	58	GC07P116672	12.6810036	https://www.genecards.org/cgi-bin/carddisp.pl?gene=MET
CDH11	Cadherin 11	Protein Coding	P55287	50	GC16M064943	12.6792574	https://www.genecards.org/cgi-bin/carddisp.pl?gene=CDH11
SERPINE1	Serpin Family E Member 1	Protein Coding	P05121	54	GC07P101127	12.6329117	https://www.genecards.org/cgi-bin/carddisp.pl?gene=SERPINE1
PARVG	Parvin Gamma	Protein Coding	Q9HBI0	39	GC22P044172	12.6182575	https://www.genecards.org/cgi-bin/carddisp.pl?gene=PARVG
CERNA3	Competing Endogenous LncRNA 3 For MiR-645	RNA Gene		13	GC08P056096	12.6073198	https://www.genecards.org/cgi-bin/carddisp.pl?gene=CERNA3
RRAS	RAS Related	Protein Coding	P10301	46	GC19M049635	12.5739708	https://www.genecards.org/cgi-bin/carddisp.pl?gene=RRAS
HRAS	HRas Proto-Oncogene, GTPase	Protein Coding	P01112	55	GC11M009562	12.5653152	https://www.genecards.org/cgi-bin/carddisp.pl?gene=HRAS
PDGFB	Platelet Derived Growth Factor Subunit B	Protein Coding	P01127	54	GC22M073492	12.5454817	https://www.genecards.org/cgi-bin/carddisp.pl?gene=PDGFB
PDGFRB	Platelet Derived Growth Factor Receptor Beta	Protein Coding	P09619	57	GC05M150113	12.5312691	https://www.genecards.org/cgi-bin/carddisp.pl?gene=PDGFRB
BCL2	BCL2 Apoptosis Regulator	Protein Coding	P10415	54	GC18M063123	12.518034	https://www.genecards.org/cgi-bin/carddisp.pl?gene=BCL2
IGF1	Insulin Like Growth Factor 1	Protein Coding	P05019	52	GC12M102395	12.5097504	https://www.genecards.org/cgi-bin/carddisp.pl?gene=IGF1
PPFIA1	PTPRF Interacting Protein Alpha 1	Protein Coding	Q13136	41	GC11P070270	12.4790239	https://www.genecards.org/cgi-bin/carddisp.pl?gene=PPFIA1
ACTG1	Actin Gamma 1	Protein Coding	P63261	51	GC17M081509	12.470027	https://www.genecards.org/cgi-bin/carddisp.pl?gene=ACTG1
DSG2	Desmoglein 2	Protein Coding	Q14126	49	GC18P031498	12.4674358	https://www.genecards.org/cgi-bin/carddisp.pl?gene=DSG2
CRKL	CRK Like Proto-Oncogene, Adaptor Protein	Protein Coding	P46109	50	GC22P020917	12.4567242	https://www.genecards.org/cgi-bin/carddisp.pl?gene=CRKL
CDKN3	Cyclin Dependent Kinase Inhibitor 3	Protein Coding	Q16667	45	GC14P054398	12.425663	https://www.genecards.org/cgi-bin/carddisp.pl?gene=CDKN3
IBSP	Integrin Binding Sialoprotein	Protein Coding	P21815	42	GC04P087799	12.40135	https://www.genecards.org/cgi-bin/carddisp.pl?gene=IBSP
TJP1	Tight Junction Protein 1	Protein Coding	Q07157	48	GC15M029699	12.3266726	https://www.genecards.org/cgi-bin/carddisp.pl?gene=TJP1
TP53	Tumor Protein P53	Protein Coding	P04637	57	GC17M007661	12.3240471	https://www.genecards.org/cgi-bin/carddisp.pl?gene=TP53

ALB	Albumin	Protein Coding	P02768	52	GC04P0 73397	12.3067083	https://www.genecards.org/cgi-bin/carddisp.pl?gene=ALB
CSK	C-Terminal Src Kinase	Protein Coding	P41240	51	GC15P0 74782	12.3059349	https://www.genecards.org/cgi-bin/carddisp.pl?gene=CSK
NID1	Nidogen 1	Protein Coding	P14543	47	GC01M2 35975	12.3016024	https://www.genecards.org/cgi-bin/carddisp.pl?gene=NID1
THY1	Thy-1 Cell Surface Antigen	Protein Coding	P04216	46	GC11M1 19505	12.2696476	https://www.genecards.org/cgi-bin/carddisp.pl?gene=THY1
CD47	CD47 Molecule	Protein Coding	Q08722	48	GC03M1 08043	12.2417192	https://www.genecards.org/cgi-bin/carddisp.pl?gene=CD47
HSPG2	Heparan Sulfate Proteoglycan 2	Protein Coding	P98160	52	GC01M0 21822	12.2321301	https://www.genecards.org/cgi-bin/carddisp.pl?gene=HSPG2
TNS2	Tensin 2	Protein Coding	Q63HR2	41	GC12P0 53046	12.2304945	https://www.genecards.org/cgi-bin/carddisp.pl?gene=TNS2
CIB1	Calcium And Integrin Binding 1	Protein Coding	Q99828	46	GC15M0 90229	12.2040844	https://www.genecards.org/cgi-bin/carddisp.pl?gene=CIB1
RET	Ret Proto-Oncogene	Protein Coding	P07949	58	GC10P0 43899	12.1948624	https://www.genecards.org/cgi-bin/carddisp.pl?gene=RET
CCN2	Cellular Communication Network Factor 2	Protein Coding	P29279	50	GC06M1 31948	12.1777287	https://www.genecards.org/cgi-bin/carddisp.pl?gene=CCN2
KRAS	KRAS Proto-Oncogene, GTPase	Protein Coding	P01116	56	GC12M0 27012	12.1749458	https://www.genecards.org/cgi-bin/carddisp.pl?gene=KRAS
GFAP	Glial Fibrillary Acidic Protein	Protein Coding	P14136	52	GC17M0 68297	12.1711464	https://www.genecards.org/cgi-bin/carddisp.pl?gene=GFAP
TGFBI	Transforming Growth Factor Beta Induced	Protein Coding	Q15582	50	GC05P1 36027	12.1403008	https://www.genecards.org/cgi-bin/carddisp.pl?gene=TGFBI
CPA6	Carboxypeptidase A6	Protein Coding	Q8N4T0	47	GC08M0 67422	12.1399269	https://www.genecards.org/cgi-bin/carddisp.pl?gene=CPA6
SDC4	Syndecan 4	Protein Coding	P31431	48	GC20M0 45325	12.0900373	https://www.genecards.org/cgi-bin/carddisp.pl?gene=SDC4
ADAM10	ADAM Metallopeptidase Domain 10	Protein Coding	O14672	57	GC15M0 58588	12.0796509	https://www.genecards.org/cgi-bin/carddisp.pl?gene=ADAM10
NF2	NF2, Moesin-Ezrin-Radixin Like (MERLIN) Tumor Suppressor	Protein Coding	P35240	52	GC22P0 29603	12.0774202	https://www.genecards.org/cgi-bin/carddisp.pl?gene=NF2
ZFYVE21	Zinc Finger FYVE-Type Containing 21	Protein Coding	Q9BQ24	36	GC14P1 03715	12.0613899	https://www.genecards.org/cgi-bin/carddisp.pl?gene=ZFYVE21
SYK	Spleen Associated Tyrosine Kinase	Protein Coding	P43405	53	GC09P0 96881	12.0461283	https://www.genecards.org/cgi-bin/carddisp.pl?gene=SYK
PRRT2	Proline Rich Transmembrane Protein 2	Protein Coding	Q7Z6L0	44	GC16P0 29811	12.0159473	https://www.genecards.org/cgi-bin/carddisp.pl?gene=PRRT2
SDCBP	Syndecan Binding Protein	Protein Coding	O00560	44	GC08P0 58539	11.9725389	https://www.genecards.org/cgi-bin/carddisp.pl?gene=SDCBP
CRP	C-Reactive Protein	Protein Coding	P02741	49	GC01M1 59734	11.9439068	https://www.genecards.org/cgi-bin/carddisp.pl?gene=CRP
BDNF-AS	BDNF Antisense RNA	RNA Gene		22	GC11P0 27466	11.9267178	https://www.genecards.org/cgi-bin/carddisp.pl?gene=BDNF-AS
RASGRP2	RAS Guanyl Releasing Protein 2	Protein Coding	Q7LDG7	47	GC11M0 64726	11.9218683	https://www.genecards.org/cgi-bin/carddisp.pl?gene=RASGRP2
TNS4	Tensin 4	Protein Coding	Q8IZW8	42	GC17M0 40475	11.9036942	https://www.genecards.org/cgi-bin/carddisp.pl?gene=TNS4

HSP90AA1	Heat Shock Protein 90 Alpha Family Class A Member 1	Protein Coding	P07900	55	GC14M102080	11.8946133	https://www.genecards.org/cgi-bin/carddisp.pl?gene=HSP90AA1
SLC35A2	Solute Carrier Family 35 Member A2	Protein Coding	P78381	44	GC0XM048903	11.8883629	https://www.genecards.org/cgi-bin/carddisp.pl?gene=SLC35A2
CD80	CD80 Molecule	Protein Coding	P33681	47	GC03M119524	11.8748989	https://www.genecards.org/cgi-bin/carddisp.pl?gene=CD80
GJA1	Gap Junction Protein Alpha 1	Protein Coding	P17302	54	GC06P123193	11.8624401	https://www.genecards.org/cgi-bin/carddisp.pl?gene=GJA1
CASK	Calcium/Calmodulin Dependent Serine Protein Kinase	Protein Coding	O14936	53	GC0XM041514	11.7900391	https://www.genecards.org/cgi-bin/carddisp.pl?gene=CASK
SORBS1	Sorbin And SH3 Domain Containing 1	Protein Coding	Q9BX66	45	GC10M095311	11.7871323	https://www.genecards.org/cgi-bin/carddisp.pl?gene=SORBS1
SORBS3	Sorbin And SH3 Domain Containing 3	Protein Coding	O60504	42	GC08P022544	11.7583237	https://www.genecards.org/cgi-bin/carddisp.pl?gene=SORBS3
ADAMTSL1	ADAMTS Like 1	Protein Coding	Q8N6G6	45	GC09P017906	11.7239542	https://www.genecards.org/cgi-bin/carddisp.pl?gene=ADAMTSL1
CAPN2	Calpain 2	Protein Coding	P17655	51	GC01P223701	11.7051773	https://www.genecards.org/cgi-bin/carddisp.pl?gene=CAPN2
INS	Insulin	Protein Coding	P01308	51	GC11M002159	11.6840582	https://www.genecards.org/cgi-bin/carddisp.pl?gene=INS
GP1BA	Glycoprotein Ib Platelet Subunit Alpha	Protein Coding	P07359	51	GC17P004932	11.683588	https://www.genecards.org/cgi-bin/carddisp.pl?gene=GP1BA
ZEB1	Zinc Finger E-Box Binding Homeobox 1	Protein Coding	P37275	52	GC10P031318	11.6751642	https://www.genecards.org/cgi-bin/carddisp.pl?gene=ZEB1
ANTXR1	ANTXR Cell Adhesion Molecule 1	Protein Coding	Q9H6X2	49	GC02P068977	11.6453552	https://www.genecards.org/cgi-bin/carddisp.pl?gene=ANTXR1
RAF1	Raf-1 Proto-Oncogene, Serine/Threonine Kinase	Protein Coding	P04049	58	GC03M012583	11.6168995	https://www.genecards.org/cgi-bin/carddisp.pl?gene=RAF1
IL17A	Interleukin 17A	Protein Coding	Q16552	47	GC06P052186	11.6046991	https://www.genecards.org/cgi-bin/carddisp.pl?gene=IL17A
HGF	Hepatocyte Growth Factor	Protein Coding	P14210	55	GC07M081699	11.5564785	https://www.genecards.org/cgi-bin/carddisp.pl?gene=HGF
CD2	CD2 Molecule	Protein Coding	P06729	48	GC01P116754	11.4952555	https://www.genecards.org/cgi-bin/carddisp.pl?gene=CD2
PKD1	Polycystin 1, Transient Receptor Potential Channel Interacting	Protein Coding	P98161	50	GC16M014080	11.4930592	https://www.genecards.org/cgi-bin/carddisp.pl?gene=PKD1
ITGA11	Integrin Subunit Alpha 11	Protein Coding	Q9UKX5	46	GC15M068296	11.4901333	https://www.genecards.org/cgi-bin/carddisp.pl?gene=ITGA11
CCN1	Cellular Communication Network Factor 1	Protein Coding	O00622	44	GC01P085585	11.4662209	https://www.genecards.org/cgi-bin/carddisp.pl?gene=CCN1
CDKN1B	Cyclin Dependent Kinase Inhibitor 1B	Protein Coding	P46527	52	GC12P033950	11.4554644	https://www.genecards.org/cgi-bin/carddisp.pl?gene=CDKN1B
GABRA1	Gamma-Aminobutyric Acid Type A Receptor Subunit Alpha1	Protein Coding	P14867	51	GC05P161847	11.4384127	https://www.genecards.org/cgi-bin/carddisp.pl?gene=GABRA1
GSK3B	Glycogen Synthase Kinase 3 Beta	Protein Coding	P49841	54	GC03M119821	11.4055099	https://www.genecards.org/cgi-bin/carddisp.pl?gene=GSK3B
HCK	HCK Proto-Oncogene, Src Family Tyrosine Kinase	Protein Coding	P08631	52	GC20P032052	11.3859921	https://www.genecards.org/cgi-bin/carddisp.pl?gene=HCK
GRIN1	Glutamate Ionotropic Receptor NMDA Type Subunit 1	Protein Coding	Q05586	54	GC09P137138	11.3781404	https://www.genecards.org/cgi-bin/carddisp.pl?gene=GRIN1

FLT1	Fms Related Receptor Tyrosine Kinase 1	Protein Coding	P17948	54	GC13M028300	11.3711414	https://www.genecards.org/cgi-bin/carddisp.pl?gene=FLT1
ENAH	ENAH Actin Regulator	Protein Coding	Q8N8S7	45	GC01M225486	11.3703241	https://www.genecards.org/cgi-bin/carddisp.pl?gene=ENAH
CHRNA4	Cholinergic Receptor Nicotinic Alpha 4 Subunit	Protein Coding	P43681	53	GC20M063343	11.3622084	https://www.genecards.org/cgi-bin/carddisp.pl?gene=CHRNA4
IQGAP1	IQ Motif Containing GTPase Activating Protein 1	Protein Coding	P46940	47	GC15P090388	11.3382092	https://www.genecards.org/cgi-bin/carddisp.pl?gene=IQGAP1
CDH13	Cadherin 13	Protein Coding	P55290	48	GC16P082626	11.3152008	https://www.genecards.org/cgi-bin/carddisp.pl?gene=CDH13
CXCR4	C-X-C Motif Chemokine Receptor 4	Protein Coding	P61073	55	GC02M136114	11.2994747	https://www.genecards.org/cgi-bin/carddisp.pl?gene=CXCR4
DST	Dystonin	Protein Coding	Q03001	46	GC06M056457	11.2624035	https://www.genecards.org/cgi-bin/carddisp.pl?gene=DST
ACP1	Acid Phosphatase 1	Protein Coding	P24666	47	GC02P000619	11.2492332	https://www.genecards.org/cgi-bin/carddisp.pl?gene=ACP1
SIRPA	Signal Regulatory Protein Alpha	Protein Coding	P78324	49	GC20P001894	11.2280607	https://www.genecards.org/cgi-bin/carddisp.pl?gene=SIRPA
ANTXR2	ANTXR Cell Adhesion Molecule 2	Protein Coding	P58335	49	GC04M079901	11.2193623	https://www.genecards.org/cgi-bin/carddisp.pl?gene=ANTXR2
IL10	Interleukin 10	Protein Coding	P22301	51	GC01M206767	11.199295	https://www.genecards.org/cgi-bin/carddisp.pl?gene=IL10
ACE	Angiotensin I Converting Enzyme	Protein Coding	P12821	55	GC17P063477	11.1630058	https://www.genecards.org/cgi-bin/carddisp.pl?gene=ACE
FAM107A	Family With Sequence Similarity 107 Member A	Protein Coding	O95990	40	GC03M058849	11.1424103	https://www.genecards.org/cgi-bin/carddisp.pl?gene=FAM107A
PPFIBP1	PPFIA Binding Protein 1	Protein Coding	Q86W92	46	GC12P027523	11.1099052	https://www.genecards.org/cgi-bin/carddisp.pl?gene=PPFIBP1
APC	APC Regulator Of WNT Signaling Pathway	Protein Coding	P25054	53	GC05P112707	11.0866623	https://www.genecards.org/cgi-bin/carddisp.pl?gene=APC
MME	Membrane Metalloendopeptidase	Protein Coding	P08473	53	GC03P155024	11.0834827	https://www.genecards.org/cgi-bin/carddisp.pl?gene=MME
LYN	LYN Proto-Oncogene, Src Family Tyrosine Kinase	Protein Coding	P07948	52	GC08P055879	11.0819931	https://www.genecards.org/cgi-bin/carddisp.pl?gene=LYN
ARHGAP24	Rho GTPase Activating Protein 24	Protein Coding	Q8N264	44	GC04P085475	11.0493679	https://www.genecards.org/cgi-bin/carddisp.pl?gene=ARHGAP24
STXBP1	Syntaxin Binding Protein 1	Protein Coding	P61764	53	GC09P127605	11.0331364	https://www.genecards.org/cgi-bin/carddisp.pl?gene=STXBP1
BSG	Basigin (Ok Blood Group)	Protein Coding	P35613	48	GC19P000571	10.965167	https://www.genecards.org/cgi-bin/carddisp.pl?gene=BSG
CSF1	Colony Stimulating Factor 1	Protein Coding	P09603	48	GC01P109911	10.9636822	https://www.genecards.org/cgi-bin/carddisp.pl?gene=CSF1
CDKN1A	Cyclin Dependent Kinase Inhibitor 1A	Protein Coding	P38936	53	GC06P119063	10.9458637	https://www.genecards.org/cgi-bin/carddisp.pl?gene=CDKN1A
LGALS3	Galectin 3	Protein Coding	P17931	48	GC14P055124	10.9384632	https://www.genecards.org/cgi-bin/carddisp.pl?gene=LGALS3
DLC1	DLC1 Rho GTPase Activating Protein	Protein Coding	Q96QB1	48	GC08M013083	10.9236364	https://www.genecards.org/cgi-bin/carddisp.pl?gene=DLC1
LCK	LCK Proto-Oncogene, Src Family Tyrosine Kinase	Protein Coding	P06239	55	GC01P032251	10.9142036	https://www.genecards.org/cgi-bin/carddisp.pl?gene=LCK

APP	Amyloid Beta Precursor Protein	Protein Coding	P05067	54	GC21M025880	10.9136648	https://www.genecards.org/cgi-bin/carddisp.pl?gene=APP
IL2	Interleukin 2	Protein Coding	P60568	49	GC04M122451	10.8936796	https://www.genecards.org/cgi-bin/carddisp.pl?gene=IL2
GNB1	G Protein Subunit Beta 1	Protein Coding	P62873	49	GC01M001785	10.8759537	https://www.genecards.org/cgi-bin/carddisp.pl?gene=GNB1
CXCR2	C-X-C Motif Chemokine Receptor 2	Protein Coding	P25025	54	GC02P218125	10.865942	https://www.genecards.org/cgi-bin/carddisp.pl?gene=CXCR2
NRP1	Neuropilin 1	Protein Coding	O14786	51	GC10M033177	10.8627548	https://www.genecards.org/cgi-bin/carddisp.pl?gene=NRP1
PIK3R2	Phosphoinositide-3-Kinase Regulatory Subunit 2	Protein Coding	O00459	53	GC19P018153	10.8621445	https://www.genecards.org/cgi-bin/carddisp.pl?gene=PIK3R2
TGM2	Transglutaminase 2	Protein Coding	P21980	51	GC20M038127	10.85851	https://www.genecards.org/cgi-bin/carddisp.pl?gene=TGM2
CSF1R	Colony Stimulating Factor 1 Receptor	Protein Coding	P07333	55	GC05M150053	10.856863	https://www.genecards.org/cgi-bin/carddisp.pl?gene=CSF1R
CASP3	Caspase 3	Protein Coding	P42574	53	GC04M184627	10.8549976	https://www.genecards.org/cgi-bin/carddisp.pl?gene=CASP3
CACNA1A	Calcium Voltage-Gated Channel Subunit Alpha1 A	Protein Coding	O00555	52	GC19M013206	10.8439913	https://www.genecards.org/cgi-bin/carddisp.pl?gene=CACNA1A
CLDN1	Claudin 1	Protein Coding	O95832	50	GC03M190305	10.843935	https://www.genecards.org/cgi-bin/carddisp.pl?gene=CLDN1
DSG3	Desmoglein 3	Protein Coding	P32926	45	GC18P031447	10.8268967	https://www.genecards.org/cgi-bin/carddisp.pl?gene=DSG3
CX3CL1	C-X3-C Motif Chemokine Ligand 1	Protein Coding	P78423	47	GC16P057372	10.7838354	https://www.genecards.org/cgi-bin/carddisp.pl?gene=CX3CL1
SVIL	Supervillin	Protein Coding	O95425	45	GC10M034989	10.7652187	https://www.genecards.org/cgi-bin/carddisp.pl?gene=SVIL
JAK2	Janus Kinase 2	Protein Coding	O60674	57	GC09P004985	10.7075939	https://www.genecards.org/cgi-bin/carddisp.pl?gene=JAK2
PPP1CB	Protein Phosphatase 1 Catalytic Subunit Beta	Protein Coding	P62140	50	GC02P028757	10.7058964	https://www.genecards.org/cgi-bin/carddisp.pl?gene=PPP1CB
ACTA1	Actin Alpha 1, Skeletal Muscle	Protein Coding	P68133	51	GC01M230051	10.6624746	https://www.genecards.org/cgi-bin/carddisp.pl?gene=ACTA1
AFDN	Afadin, Adherens Junction Formation Factor	Protein Coding	P55196	46	GC06P167827	10.6565151	https://www.genecards.org/cgi-bin/carddisp.pl?gene=AFDN
ITGB8	Integrin Subunit Beta 8	Protein Coding	P26012	45	GC07P020329	10.5977964	https://www.genecards.org/cgi-bin/carddisp.pl?gene=ITGB8
CALR	Calreticulin	Protein Coding	P27797	55	GC19P012938	10.5887136	https://www.genecards.org/cgi-bin/carddisp.pl?gene=CALR
CCR6	C-C Motif Chemokine Receptor 6	Protein Coding	P51684	47	GC06P167111	10.5837736	https://www.genecards.org/cgi-bin/carddisp.pl?gene=CCR6
SYNPO	Synaptopodin	Protein Coding	Q8N3V7	43	GC05P150601	10.5796003	https://www.genecards.org/cgi-bin/carddisp.pl?gene=SYNPO
PIK3CD	Phosphatidylinositol-4,5-Bisphosphate 3-Kinase Catalytic Subunit Delta	Protein Coding	O00329	56	GC01P009640	10.5758514	https://www.genecards.org/cgi-bin/carddisp.pl?gene=PIK3CD
MYOC	Myocilin	Protein Coding	Q99972	46	GC01M171604	10.5708294	https://www.genecards.org/cgi-bin/carddisp.pl?gene=MYOC
NRXN1	Neurexin 1	Protein Coding	Q9ULB1	52	GC02M049918	10.5424862	https://www.genecards.org/cgi-bin/carddisp.pl?gene=NRXN1

CD99	CD99 Molecule (Xg Blood Group)	Protein Coding	P14209	42	GC0XP002691	10.5213547	https://www.genecards.org/cgi-bin/carddisp.pl?gene=CD99
PALLD	Palladin, Cytoskeletal Associated Protein	Protein Coding	Q8WX93	46	GC04P168497	10.5113554	https://www.genecards.org/cgi-bin/carddisp.pl?gene=PALLD
FGFR1	Fibroblast Growth Factor Receptor 1	Protein Coding	P11362	58	GC08M038400	10.4738026	https://www.genecards.org/cgi-bin/carddisp.pl?gene=FGFR1
GRP	Gastrin Releasing Peptide	Protein Coding	P07492	44	GC18P059220	10.4694071	https://www.genecards.org/cgi-bin/carddisp.pl?gene=GRP
FGFR2	Fibroblast Growth Factor Receptor 2	Protein Coding	P21802	59	GC10M121478	10.4640417	https://www.genecards.org/cgi-bin/carddisp.pl?gene=FGFR2
COL2A1	Collagen Type II Alpha 1 Chain	Protein Coding	P02458	53	GC12M047972	10.4513893	https://www.genecards.org/cgi-bin/carddisp.pl?gene=COL2A1
LIMS2	LIM Zinc Finger Domain Containing 2	Protein Coding	Q7Z4I7	44	GC02M127638	10.4257326	https://www.genecards.org/cgi-bin/carddisp.pl?gene=LIMS2
RASA1	RAS P21 Protein Activator 1	Protein Coding	P20936	50	GC05P087267	10.3979874	https://www.genecards.org/cgi-bin/carddisp.pl?gene=RASA1
LAMB1	Laminin Subunit Beta 1	Protein Coding	P07942	53	GC07M107923	10.3651648	https://www.genecards.org/cgi-bin/carddisp.pl?gene=LAMB1
PDCD10	Programmed Cell Death 10	Protein Coding	Q9BUL8	45	GC03M167683	10.3568287	https://www.genecards.org/cgi-bin/carddisp.pl?gene=PDCD10
DNM1	Dynamin 1	Protein Coding	Q05193	53	GC09P128191	10.3443689	https://www.genecards.org/cgi-bin/carddisp.pl?gene=DNM1
NOS2	Nitric Oxide Synthase 2	Protein Coding	P35228	53	GC17M027756	10.3263092	https://www.genecards.org/cgi-bin/carddisp.pl?gene=NOS2
SBF2	SET Binding Factor 2	Protein Coding	Q86WG5	42	GC11M010171	10.320611	https://www.genecards.org/cgi-bin/carddisp.pl?gene=SBF2
IL1A	Interleukin 1 Alpha	Protein Coding	P01583	49	GC02M112773	10.310482	https://www.genecards.org/cgi-bin/carddisp.pl?gene=IL1A
STAT3	Signal Transducer And Activator Of Transcription 3	Protein Coding	P40763	57	GC17M042313	10.2981033	https://www.genecards.org/cgi-bin/carddisp.pl?gene=STAT3
TNS3	Tensin 3	Protein Coding	Q68CZ2	41	GC07M047281	10.296236	https://www.genecards.org/cgi-bin/carddisp.pl?gene=TNS3
CHRNB2	Cholinergic Receptor Nicotinic Beta 2 Subunit	Protein Coding	P17787	49	GC01P158777	10.2766504	https://www.genecards.org/cgi-bin/carddisp.pl?gene=CHRNB2
CYP1B1	Cytochrome P450 Family 1 Subfamily B Member 1	Protein Coding	Q16678	51	GC02M038066	10.2674618	https://www.genecards.org/cgi-bin/carddisp.pl?gene=CYP1B1
ANGPT1	Angiopoietin 1	Protein Coding	Q15389	50	GC08M107246	10.255167	https://www.genecards.org/cgi-bin/carddisp.pl?gene=ANGPT1
ATP2A2	ATPase Sarcoplasmic/Endoplasmic Reticulum Ca2+ Transporting 2	Protein Coding	P16615	54	GC12P110280	10.2543354	https://www.genecards.org/cgi-bin/carddisp.pl?gene=ATP2A2
KCNJ11	Potassium Inwardly Rectifying Channel Subfamily J Member 11	Protein Coding	Q14654	50	GC11M017865	10.2505169	https://www.genecards.org/cgi-bin/carddisp.pl?gene=KCNJ11
NOTCH1	Notch Receptor 1	Protein Coding	P46531	55	GC09M138467	10.2447529	https://www.genecards.org/cgi-bin/carddisp.pl?gene=NOTCH1
TRIM15	Tripartite Motif Containing 15	Protein Coding	Q9C019	37	GC06P118859	10.2412567	https://www.genecards.org/cgi-bin/carddisp.pl?gene=TRIM15
NUP133	Nucleoporin 133	Protein Coding	Q8WUM0	46	GC01M230053	10.1951008	https://www.genecards.org/cgi-bin/carddisp.pl?gene=NUP133
DOCK1	Dedicator Of Cytokinesis 1	Protein Coding	Q14185	49	GC10P126905	10.1899719	https://www.genecards.org/cgi-bin/carddisp.pl?gene=DOCK1

TES	Testin LIM Domain Protein	Protein Coding	Q9UGI8	44	GC07P116210	10.1851454	https://www.genecards.org/cgi-bin/carddisp.pl?gene=TES
ADRM1	ADRM1 26S Proteasome Ubiquitin Receptor	Protein Coding	Q16186	43	GC20P062302	10.1847916	https://www.genecards.org/cgi-bin/carddisp.pl?gene=ADRM1
LAMA1	Laminin Subunit Alpha 1	Protein Coding	P25391	50	GC18M006941	10.1776476	https://www.genecards.org/cgi-bin/carddisp.pl?gene=LAMA1
PIP5K1A	Phosphatidylinositol-4-Phosphate 5-Kinase Type 1 Alpha	Protein Coding	Q99755	47	GC01P158588	10.1740074	https://www.genecards.org/cgi-bin/carddisp.pl?gene=PIP5K1A
NOS3	Nitric Oxide Synthase 3	Protein Coding	P29474	53	GC07P154525	10.1358624	https://www.genecards.org/cgi-bin/carddisp.pl?gene=NOS3
TNR	Tenascin R	Protein Coding	Q92752	47	GC01M175291	10.115613	https://www.genecards.org/cgi-bin/carddisp.pl?gene=TNR
FGR	FGR Proto-Oncogene, Src Family Tyrosine Kinase	Protein Coding	P09769	51	GC01M030208	10.1075048	https://www.genecards.org/cgi-bin/carddisp.pl?gene=FGR
CTNND2	Catenin Delta 2	Protein Coding	Q9UQB3	46	GC05M010971	10.095787	https://www.genecards.org/cgi-bin/carddisp.pl?gene=CTNND2
FLNB	Filamin B	Protein Coding	O75369	51	GC03P058008	10.0932274	https://www.genecards.org/cgi-bin/carddisp.pl?gene=FLNB
PTPRU	Protein Tyrosine Phosphatase Receptor Type U	Protein Coding	Q92729	46	GC01P029236	10.076786	https://www.genecards.org/cgi-bin/carddisp.pl?gene=PTPRU
PTPRF	Protein Tyrosine Phosphatase Receptor Type F	Protein Coding	P10586	53	GC01P043614	10.0737581	https://www.genecards.org/cgi-bin/carddisp.pl?gene=PTPRF
LAMA2	Laminin Subunit Alpha 2	Protein Coding	P24043	48	GC06P128863	10.0669193	https://www.genecards.org/cgi-bin/carddisp.pl?gene=LAMA2
PKP2	Plakophilin 2	Protein Coding	Q99959	47	GC12M032790	10.0567684	https://www.genecards.org/cgi-bin/carddisp.pl?gene=PKP2
DPP4	Dipeptidyl Peptidase 4	Protein Coding	P27487	53	GC02M161992	10.0367947	https://www.genecards.org/cgi-bin/carddisp.pl?gene=DPP4
LINC01672	Long Intergenic Non-Protein Coding RNA 1672	RNA Gene		13	GC01P008900	10.0341072	https://www.genecards.org/cgi-bin/carddisp.pl?gene=LINC01672
GNAO1	G Protein Subunit Alpha O1	Protein Coding	P09471	50	GC16P061440	9.98262405	https://www.genecards.org/cgi-bin/carddisp.pl?gene=GNAO1
DSC2	Desmocollin 2	Protein Coding	Q02487	49	GC18M031058	9.98128414	https://www.genecards.org/cgi-bin/carddisp.pl?gene=DSC2
IL4	Interleukin 4	Protein Coding	P05112	50	GC05P132673	9.97125912	https://www.genecards.org/cgi-bin/carddisp.pl?gene=IL4
NUP205	Nucleoporin 205	Protein Coding	Q92621	42	GC07P135557	9.93860054	https://www.genecards.org/cgi-bin/carddisp.pl?gene=NUP205
GRIN2B	Glutamate Ionotropic Receptor NMDA Type Subunit 2B	Protein Coding	Q13224	55	GC12M013437	9.92779064	https://www.genecards.org/cgi-bin/carddisp.pl?gene=GRIN2B
IL2RA	Interleukin 2 Receptor Subunit Alpha	Protein Coding	P01589	54	GC10M006010	9.90362644	https://www.genecards.org/cgi-bin/carddisp.pl?gene=IL2RA
SLC4A11	Solute Carrier Family 4 Member 11	Protein Coding	Q8NBS3	44	GC20M003931	9.88841438	https://www.genecards.org/cgi-bin/carddisp.pl?gene=SLC4A11
NPHP1	Nephrocystin 1	Protein Coding	O15259	47	GC02M110122	9.88482857	https://www.genecards.org/cgi-bin/carddisp.pl?gene=NPHP1
LAMC1	Laminin Subunit Gamma 1	Protein Coding	P11047	47	GC01P183023	9.87281609	https://www.genecards.org/cgi-bin/carddisp.pl?gene=LAMC1
FGF2	Fibroblast Growth Factor 2	Protein Coding	P09038	49	GC04P122826	9.8617754	https://www.genecards.org/cgi-bin/carddisp.pl?gene=FGF2

COL17A1	Collagen Type XVII Alpha 1 Chain	Protein Coding	Q9UMD9	48	GC10M104031	9.84636116	https://www.genecards.org/cgi-bin/carddisp.pl?gene=COL17A1
KCNA1	Potassium Voltage-Gated Channel Subfamily A Member 1	Protein Coding	Q09470	49	GC12P033689	9.83690548	https://www.genecards.org/cgi-bin/carddisp.pl?gene=KCNA1
MACF1	Microtubule Actin Crosslinking Factor 1	Protein Coding	O94854	46	GC01P039082	9.79134655	https://www.genecards.org/cgi-bin/carddisp.pl?gene=MACF1
COL8A2	Collagen Type VIII Alpha 2 Chain	Protein Coding	P25067	44	GC01M036095	9.76687145	https://www.genecards.org/cgi-bin/carddisp.pl?gene=COL8A2
TUBA1A	Tubulin Alpha 1a	Protein Coding	Q71U36	52	GC12M049184	9.75322342	https://www.genecards.org/cgi-bin/carddisp.pl?gene=TUBA1A
LAMB3	Laminin Subunit Beta 3	Protein Coding	Q13751	50	GC01M209614	9.75304317	https://www.genecards.org/cgi-bin/carddisp.pl?gene=LAMB3
NUP107	Nucleoporin 107	Protein Coding	P57740	47	GC12P068686	9.73222923	https://www.genecards.org/cgi-bin/carddisp.pl?gene=NUP107
ANXA1	Annexin A1	Protein Coding	P04083	51	GC09P073151	9.72944164	https://www.genecards.org/cgi-bin/carddisp.pl?gene=ANXA1
COL1A1	Collagen Type I Alpha 1 Chain	Protein Coding	P02452	53	GC17M068598	9.72496891	https://www.genecards.org/cgi-bin/carddisp.pl?gene=COL1A1
PRKD1	Protein Kinase D1	Protein Coding	Q15139	52	GC14M029576	9.71006203	https://www.genecards.org/cgi-bin/carddisp.pl?gene=PRKD1
GRHL2	Grainyhead Like Transcription Factor 2	Protein Coding	Q6ISB3	45	GC08P101492	9.70255756	https://www.genecards.org/cgi-bin/carddisp.pl?gene=GRHL2
LAMC3	Laminin Subunit Gamma 3	Protein Coding	Q9Y6N6	45	GC09P131009	9.686059	https://www.genecards.org/cgi-bin/carddisp.pl?gene=LAMC3
RAPGEF1	Rap Guanine Nucleotide Exchange Factor 1	Protein Coding	Q13905	45	GC09M131576	9.61592102	https://www.genecards.org/cgi-bin/carddisp.pl?gene=RAPGEF1
DYNC1H1	Dynein Cytoplasmic 1 Heavy Chain 1	Protein Coding	Q14204	49	GC14P113788	9.61311531	https://www.genecards.org/cgi-bin/carddisp.pl?gene=DYNC1H1
FER	FER Tyrosine Kinase	Protein Coding	P16591	50	GC05P108747	9.61230564	https://www.genecards.org/cgi-bin/carddisp.pl?gene=FER
GAL	Galanin And GMAP Prepropeptide	Protein Coding	P22466	48	GC11P082144	9.6026535	https://www.genecards.org/cgi-bin/carddisp.pl?gene=GAL
RAC3	Rac Family Small GTPase 3	Protein Coding	P60763	49	GC17P082031	9.59687996	https://www.genecards.org/cgi-bin/carddisp.pl?gene=RAC3
PCDH12	Protocadherin 12	Protein Coding	Q9NPG4	44	GC05M141943	9.59054089	https://www.genecards.org/cgi-bin/carddisp.pl?gene=PCDH12
AGT	Angiotensinogen	Protein Coding	P01019	53	GC01M230690	9.58939171	https://www.genecards.org/cgi-bin/carddisp.pl?gene=AGT
MICAL1	Microtubule Associated Monooxygenase, Calponin And LIM Domain Containing 1	Protein Coding	Q8TDZ2	46	GC06M109444	9.58634758	https://www.genecards.org/cgi-bin/carddisp.pl?gene=MICAL1
THBS2	Thrombospondin 2	Protein Coding	P35442	50	GC06M169215	9.57202435	https://www.genecards.org/cgi-bin/carddisp.pl?gene=THBS2
UTRN	Utrophin	Protein Coding	P46939	44	GC06P144285	9.55677414	https://www.genecards.org/cgi-bin/carddisp.pl?gene=UTRN
PPP2CA	Protein Phosphatase 2 Catalytic Subunit Alpha	Protein Coding	P67775	53	GC05M134194	9.54868126	https://www.genecards.org/cgi-bin/carddisp.pl?gene=PPP2CA
DNM2	Dynamin 2	Protein Coding	P50570	53	GC19P010718	9.54353905	https://www.genecards.org/cgi-bin/carddisp.pl?gene=DNM2
KRT8	Keratin 8	Protein Coding	P05787	50	GC12M052897	9.53429127	https://www.genecards.org/cgi-bin/carddisp.pl?gene=KRT8

CASS4	Cas Scaffold Protein Family Member 4	Protein Coding	Q9NQ75	38	GC20P056411	9.51735973	https://www.genecards.org/cgi-bin/carddisp.pl?gene=CASS4
AKT2	AKT Serine/Threonine Kinase 2	Protein Coding	P31751	57	GC19M040230	9.51130104	https://www.genecards.org/cgi-bin/carddisp.pl?gene=AKT2
MTMR2	Myotubularin Related Protein 2	Protein Coding	Q13614	49	GC11M117552	9.50742626	https://www.genecards.org/cgi-bin/carddisp.pl?gene=MTMR2
CCL5	C-C Motif Chemokine Ligand 5	Protein Coding	P13501	47	GC17M035871	9.48224068	https://www.genecards.org/cgi-bin/carddisp.pl?gene=CCL5
ADAM15	ADAM Metallopeptidase Domain 15	Protein Coding	Q13444	47	GC01P155050	9.48200607	https://www.genecards.org/cgi-bin/carddisp.pl?gene=ADAM15
PTK7	Protein Tyrosine Kinase 7 (Inactive)	Protein Coding	Q13308	48	GC06P043076	9.47875786	https://www.genecards.org/cgi-bin/carddisp.pl?gene=PTK7
PIK3CB	Phosphatidylinositol-4,5-Bisphosphate 3-Kinase Catalytic Subunit Beta	Protein Coding	P42338	51	GC03M138652	9.45123577	https://www.genecards.org/cgi-bin/carddisp.pl?gene=PIK3CB
YWHAQ	Tyrosine 3-Monooxygenase/Tryptophan 5-Monooxygenase Activation Protein Theta	Protein Coding	P27348	50	GC02M009583	9.44953823	https://www.genecards.org/cgi-bin/carddisp.pl?gene=YWHAQ
SIGLEC5	Sialic Acid Binding Ig Like Lectin 5	Protein Coding	O15389	44	GC19M085981	9.43880081	https://www.genecards.org/cgi-bin/carddisp.pl?gene=SIGLEC5
ARF6	ADP Ribosylation Factor 6	Protein Coding	P62330	47	GC14P049938	9.43566704	https://www.genecards.org/cgi-bin/carddisp.pl?gene=ARF6
ATP1A2	ATPase Na+/K+ Transporting Subunit Alpha 2	Protein Coding	P50993	52	GC01P160115	9.43227673	https://www.genecards.org/cgi-bin/carddisp.pl?gene=ATP1A2
TUBB2B	Tubulin Beta 2B Class IIb	Protein Coding	Q9BVA1	47	GC06M003611	9.41961288	https://www.genecards.org/cgi-bin/carddisp.pl?gene=TUBB2B
GNA12	G Protein Subunit Alpha 12	Protein Coding	Q03113	43	GC07M002728	9.40472794	https://www.genecards.org/cgi-bin/carddisp.pl?gene=GNA12
BCAR3	BCAR3 Adaptor Protein, NSP Family Member	Protein Coding	O75815	42	GC01M093561	9.3794384	https://www.genecards.org/cgi-bin/carddisp.pl?gene=BCAR3
NF1	Neurofibromin 1	Protein Coding	P21359	52	GC17P031094	9.34495354	https://www.genecards.org/cgi-bin/carddisp.pl?gene=NF1
MFF-DT	MFF Divergent Transcript	RNA Gene		15	GC02M227223	9.34166336	https://www.genecards.org/cgi-bin/carddisp.pl?gene=MFF-DT
THBS4	Thrombospondin 4	Protein Coding	P35443	46	GC05P079991	9.33898163	https://www.genecards.org/cgi-bin/carddisp.pl?gene=THBS4
MMP1	Matrix Metallopeptidase 1	Protein Coding	P03956	53	GC11M117619	9.31787491	https://www.genecards.org/cgi-bin/carddisp.pl?gene=MMP1
COL4A2	Collagen Type IV Alpha 2 Chain	Protein Coding	P08572	50	GC13P110305	9.30985928	https://www.genecards.org/cgi-bin/carddisp.pl?gene=COL4A2
MT-CYB	Mitochondrially Encoded Cytochrome B	Protein Coding	P00156	35	GCMTP014749	9.28732109	https://www.genecards.org/cgi-bin/carddisp.pl?gene=MT-CYB
SPARC	Secreted Protein Acidic And Cysteine Rich	Protein Coding	P09486	52	GC05M151661	9.25194263	https://www.genecards.org/cgi-bin/carddisp.pl?gene=SPARC
ESR1	Estrogen Receptor 1	Protein Coding	P03372	57	GC06P151656	9.21712875	https://www.genecards.org/cgi-bin/carddisp.pl?gene=ESR1
PPP3CA	Protein Phosphatase 3 Catalytic Subunit Alpha	Protein Coding	Q08209	55	GC04M101024	9.21635628	https://www.genecards.org/cgi-bin/carddisp.pl?gene=PPP3CA
PTPRJ	Protein Tyrosine Phosphatase Receptor Type J	Protein Coding	Q12913	52	GC11P048002	9.2124157	https://www.genecards.org/cgi-bin/carddisp.pl?gene=PTPRJ
VAV1	Vav Guanine Nucleotide Exchange Factor 1	Protein Coding	P15498	48	GC19P006772	9.2072506	https://www.genecards.org/cgi-bin/carddisp.pl?gene=VAV1

AGRN	Agrin	Protein Coding	O00468	48	GC01P001020	9.19837379	https://www.genecards.org/cgi-bin/carddisp.pl?gene=AGRN
ADAM17	ADAM Metallopeptidase Domain 17	Protein Coding	P78536	54	GC02M009488	9.18112755	https://www.genecards.org/cgi-bin/carddisp.pl?gene=ADAM17
PCNA	Proliferating Cell Nuclear Antigen	Protein Coding	P12004	54	GC20M005114	9.17477226	https://www.genecards.org/cgi-bin/carddisp.pl?gene=PCNA
IL5	Interleukin 5	Protein Coding	P05113	48	GC05M132541	9.12557793	https://www.genecards.org/cgi-bin/carddisp.pl?gene=IL5
VAV2	Vav Guanine Nucleotide Exchange Factor 2	Protein Coding	P52735	46	GC09M133761	9.11048889	https://www.genecards.org/cgi-bin/carddisp.pl?gene=VAV2
TIMP1	TIMP Metallopeptidase Inhibitor 1	Protein Coding	P01033	48	GC0XP053104	9.07226944	https://www.genecards.org/cgi-bin/carddisp.pl?gene=TIMP1
BMX	BMX Non-Receptor Tyrosine Kinase	Protein Coding	P51813	49	GC0XP015392	9.05320168	https://www.genecards.org/cgi-bin/carddisp.pl?gene=BMX
CR1	Complement C3b/C4b Receptor 1 (Knops Blood Group)	Protein Coding	P17927	48	GC01P207496	9.03752232	https://www.genecards.org/cgi-bin/carddisp.pl?gene=CR1
PI4KA	Phosphatidylinositol 4-Kinase Alpha	Protein Coding	P42356	52	GC22M020707	9.02501297	https://www.genecards.org/cgi-bin/carddisp.pl?gene=PI4KA
PTPN6	Protein Tyrosine Phosphatase Non-Receptor Type 6	Protein Coding	P29350	53	GC12P033753	9.02248955	https://www.genecards.org/cgi-bin/carddisp.pl?gene=PTPN6
SCARNA5	Small Cajal Body-Specific RNA 5	RNA Gene		21	GC02P233275	9.01887512	https://www.genecards.org/cgi-bin/carddisp.pl?gene=SCARNA5
CAMK2B	Calcium/Calmodulin Dependent Protein Kinase II Beta	Protein Coding	Q13554	53	GC07M044977	9.01820946	https://www.genecards.org/cgi-bin/carddisp.pl?gene=CAMK2B
MYLK	Myosin Light Chain Kinase	Protein Coding	Q15746	55	GC03M123610	9.0125103	https://www.genecards.org/cgi-bin/carddisp.pl?gene=MYLK
KRT14	Keratin 14	Protein Coding	P02533	50	GC17M041582	8.99256229	https://www.genecards.org/cgi-bin/carddisp.pl?gene=KRT14
SH3KBP1	SH3 Domain Containing Kinase Binding Protein 1	Protein Coding	Q96B97	46	GC0XM019552	8.98357677	https://www.genecards.org/cgi-bin/carddisp.pl?gene=SH3KBP1
KCNB1	Potassium Voltage-Gated Channel Subfamily B Member 1	Protein Coding	Q14721	51	GC20M049293	8.98016071	https://www.genecards.org/cgi-bin/carddisp.pl?gene=KCNB1
KRIT1	KRIT1 Ankyrin Repeat Containing	Protein Coding	O00522	45	GC07M092198	8.9779892	https://www.genecards.org/cgi-bin/carddisp.pl?gene=KRIT1
DDR1	Discoidin Domain Receptor Tyrosine Kinase 1	Protein Coding	Q08345	50	GC06P118876	8.95867252	https://www.genecards.org/cgi-bin/carddisp.pl?gene=DDR1
PMP22	Peripheral Myelin Protein 22	Protein Coding	Q01453	47	GC17M015229	8.93700123	https://www.genecards.org/cgi-bin/carddisp.pl?gene=PMP22
MGAT5	Alpha-1,6-Mannosylglycoprotein 6-Beta-N-Acetylglucosaminyltransferase	Protein Coding	Q09328	45	GC02P134119	8.90307522	https://www.genecards.org/cgi-bin/carddisp.pl?gene=MGAT5
EDN1	Endothelin 1	Protein Coding	P05305	52	GC06P012236	8.89871788	https://www.genecards.org/cgi-bin/carddisp.pl?gene=EDN1
XIAP	X-Linked Inhibitor Of Apoptosis	Protein Coding	P98170	55	GC0XP123859	8.89147377	https://www.genecards.org/cgi-bin/carddisp.pl?gene=XIAP
PRKCD	Protein Kinase C Delta	Protein Coding	Q05655	55	GC03P053156	8.87282181	https://www.genecards.org/cgi-bin/carddisp.pl?gene=PRKCD
DSC3	Desmocollin 3	Protein Coding	Q14574	46	GC18M032107	8.84280109	https://www.genecards.org/cgi-bin/carddisp.pl?gene=DSC3
MKI67	Marker Of Proliferation Ki-67	Protein Coding	P46013	48	GC10M128096	8.83417416	https://www.genecards.org/cgi-bin/carddisp.pl?gene=MKI67

VEGFC	Vascular Endothelial Growth Factor C	Protein Coding	P49767	51	GC04M176683	8.81834984	https://www.genecards.org/cgi-bin/carddisp.pl?gene=VEGFC
COL1A2	Collagen Type I Alpha 2 Chain	Protein Coding	P08123	51	GC07P094394	8.81819725	https://www.genecards.org/cgi-bin/carddisp.pl?gene=COL1A2
CDK5	Cyclin Dependent Kinase 5	Protein Coding	Q00535	56	GC07M151053	8.81015396	https://www.genecards.org/cgi-bin/carddisp.pl?gene=CDK5
NFKB1	Nuclear Factor Kappa B Subunit 1	Protein Coding	P19838	56	GC04P102501	8.81003189	https://www.genecards.org/cgi-bin/carddisp.pl?gene=NFKB1
KIT	KIT Proto-Oncogene, Receptor Tyrosine Kinase	Protein Coding	P10721	57	GC04P054657	8.79793549	https://www.genecards.org/cgi-bin/carddisp.pl?gene=KIT
HIF1A	Hypoxia Inducible Factor 1 Subunit Alpha	Protein Coding	Q16665	52	GC14P061695	8.7964859	https://www.genecards.org/cgi-bin/carddisp.pl?gene=HIF1A
WASL	WASP Like Actin Nucleation Promoting Factor	Protein Coding	O00401	46	GC07M123681	8.79453087	https://www.genecards.org/cgi-bin/carddisp.pl?gene=WASL
CBL	Cbl Proto-Oncogene	Protein Coding	P22681	54	GC11P119206	8.78489685	https://www.genecards.org/cgi-bin/carddisp.pl?gene=CBL
KLF8	KLF Transcription Factor 8	Protein Coding	O95600	41	GC0XP056368	8.78002739	https://www.genecards.org/cgi-bin/carddisp.pl?gene=KLF8
PPP1R12A	Protein Phosphatase 1 Regulatory Subunit 12A	Protein Coding	O14974	48	GC12M079773	8.76148129	https://www.genecards.org/cgi-bin/carddisp.pl?gene=PPP1R12A
ITGAE	Integrin Subunit Alpha E	Protein Coding	P38570	44	GC17M015093	8.74651718	https://www.genecards.org/cgi-bin/carddisp.pl?gene=ITGAE
CD34	CD34 Molecule	Protein Coding	P28906	49	GC01M207880	8.73776817	https://www.genecards.org/cgi-bin/carddisp.pl?gene=CD34
PAX6	Paired Box 6	Protein Coding	P26367	51	GC11M031784	8.72616386	https://www.genecards.org/cgi-bin/carddisp.pl?gene=PAX6
PTPRC	Protein Tyrosine Phosphatase Receptor Type C	Protein Coding	P08575	55	GC01P198607	8.7251482	https://www.genecards.org/cgi-bin/carddisp.pl?gene=PTPRC
MAP4K4	Mitogen-Activated Protein Kinase Kinase Kinase Kinase 4	Protein Coding	O95819	51	GC02P101696	8.72330952	https://www.genecards.org/cgi-bin/carddisp.pl?gene=MAP4K4
FGFR3	Fibroblast Growth Factor Receptor 3	Protein Coding	P22607	58	GC04P002600	8.72259331	https://www.genecards.org/cgi-bin/carddisp.pl?gene=FGFR3
SGPL1	Sphingosine-1-Phosphate Lyase 1	Protein Coding	O95470	50	GC10P070815	8.71813583	https://www.genecards.org/cgi-bin/carddisp.pl?gene=SGPL1
DDR2	Discoidin Domain Receptor Tyrosine Kinase 2	Protein Coding	Q16832	55	GC01P162631	8.70607185	https://www.genecards.org/cgi-bin/carddisp.pl?gene=DDR2
ITGA10	Integrin Subunit Alpha 10	Protein Coding	O75578	41	GC01M145891	8.68827057	https://www.genecards.org/cgi-bin/carddisp.pl?gene=ITGA10
GP5	Glycoprotein V Platelet	Protein Coding	P40197	45	GC03M194395	8.6703558	https://www.genecards.org/cgi-bin/carddisp.pl?gene=GP5
ACTC1	Actin Alpha Cardiac Muscle 1	Protein Coding	P68032	48	GC15M034790	8.66498184	https://www.genecards.org/cgi-bin/carddisp.pl?gene=ACTC1
THBD	Thrombomodulin	Protein Coding	P07204	50	GC20M023026	8.66247177	https://www.genecards.org/cgi-bin/carddisp.pl?gene=THBD
F3	Coagulation Factor III, Tissue Factor	Protein Coding	P13726	49	GC01M094782	8.66141605	https://www.genecards.org/cgi-bin/carddisp.pl?gene=F3
ADAM9	ADAM Metallopeptidase Domain 9	Protein Coding	Q13443	52	GC08P038996	8.63359642	https://www.genecards.org/cgi-bin/carddisp.pl?gene=ADAM9
INSR	Insulin Receptor	Protein Coding	P06213	57	GC19M007112	8.60558128	https://www.genecards.org/cgi-bin/carddisp.pl?gene=INSR

ASAP1	ArfGAP With SH3 Domain, Ankyrin Repeat And PH Domain 1	Protein Coding	Q9ULH1	44	GC08M130052	8.57804489	https://www.genecards.org/cgi-bin/carddisp.pl?gene=ASAP1
RHOU	Ras Homolog Family Member U	Protein Coding	Q7L0Q8	40	GC01P228644	8.5727272	https://www.genecards.org/cgi-bin/carddisp.pl?gene=RHOU
XPC	XPC Complex Subunit, DNA Damage Recognition And Repair Factor	Protein Coding	Q01831	50	GC03M024965	8.5586462	https://www.genecards.org/cgi-bin/carddisp.pl?gene=XPC
IRS1	Insulin Receptor Substrate 1	Protein Coding	P35568	51	GC02M226731	8.54753304	https://www.genecards.org/cgi-bin/carddisp.pl?gene=IRS1
RACK1	Receptor For Activated C Kinase 1	Protein Coding	P63244	47	GC05M182417	8.53693962	https://www.genecards.org/cgi-bin/carddisp.pl?gene=RACK1
EFNB2	Ephrin B2	Protein Coding	P52799	48	GC13M106489	8.51414299	https://www.genecards.org/cgi-bin/carddisp.pl?gene=EFNB2
PHLDB2	Pleckstrin Homology Like Domain Family B Member 2	Protein Coding	Q86SQ0	40	GC03P111732	8.49014282	https://www.genecards.org/cgi-bin/carddisp.pl?gene=PHLDB2
AVIL	Advillin	Protein Coding	O75366	41	GC12M058992	8.48683929	https://www.genecards.org/cgi-bin/carddisp.pl?gene=AVIL
KRT5	Keratin 5	Protein Coding	P13647	50	GC12M052514	8.48107529	https://www.genecards.org/cgi-bin/carddisp.pl?gene=KRT5
CORO2B	Coronin 2B	Protein Coding	Q9UQ03	41	GC15P142879	8.48086548	https://www.genecards.org/cgi-bin/carddisp.pl?gene=CORO2B
HLA-DRB1	Major Histocompatibility Complex, Class II, DR Beta 1	Protein Coding	P01911	50	GC06M087309	8.48021889	https://www.genecards.org/cgi-bin/carddisp.pl?gene=HLA-DRB1
F2R	Coagulation Factor II Thrombin Receptor	Protein Coding	P25116	50	GC05P076716	8.4607296	https://www.genecards.org/cgi-bin/carddisp.pl?gene=F2R
KRT1	Keratin 1	Protein Coding	P04264	51	GC12M052674	8.46036816	https://www.genecards.org/cgi-bin/carddisp.pl?gene=KRT1
JAG1	Jagged Canonical Notch Ligand 1	Protein Coding	P78504	54	GC20M010637	8.45678425	https://www.genecards.org/cgi-bin/carddisp.pl?gene=JAG1
AGTR1	Angiotensin II Receptor Type 1	Protein Coding	P30556	54	GC03P148697	8.44354057	https://www.genecards.org/cgi-bin/carddisp.pl?gene=AGTR1
GCK	Glucokinase	Protein Coding	P35557	53	GC07M044978	8.44033146	https://www.genecards.org/cgi-bin/carddisp.pl?gene=GCK
FLRT2	Fibronectin Leucine Rich Transmembrane Protein 2	Protein Coding	O43155	42	GC14P085530	8.43797493	https://www.genecards.org/cgi-bin/carddisp.pl?gene=FLRT2
CLASP2	Cytoplasmic Linker Associated Protein 2	Protein Coding	O75122	43	GC03M033537	8.42948055	https://www.genecards.org/cgi-bin/carddisp.pl?gene=CLASP2
UBE3A	Ubiquitin Protein Ligase E3A	Protein Coding	Q05086	51	GC15M025333	8.42638969	https://www.genecards.org/cgi-bin/carddisp.pl?gene=UBE3A
APOE	Apolipoprotein E	Protein Coding	P02649	53	GC19P096171	8.42607689	https://www.genecards.org/cgi-bin/carddisp.pl?gene=APOE
DOCK8	Dedicator Of Cytokinesis 8	Protein Coding	Q8NF50	48	GC09P000236	8.41679001	https://www.genecards.org/cgi-bin/carddisp.pl?gene=DOCK8
TACSTD2	Tumor Associated Calcium Signal Transducer 2	Protein Coding	P09758	48	GC01M058575	8.41555595	https://www.genecards.org/cgi-bin/carddisp.pl?gene=TACSTD2
RELA	RELA Proto-Oncogene, NF-KB Subunit	Protein Coding	Q04206	54	GC11M065653	8.40407562	https://www.genecards.org/cgi-bin/carddisp.pl?gene=RELA
KCNMA1	Potassium Calcium-Activated Channel Subfamily M Alpha 1	Protein Coding	Q12791	52	GC10M076869	8.3941555	https://www.genecards.org/cgi-bin/carddisp.pl?gene=KCNMA1
LRP5	LDL Receptor Related Protein 5	Protein Coding	O75197	53	GC11P068298	8.386693	https://www.genecards.org/cgi-bin/carddisp.pl?gene=LRP5

GCH1	GTP Cyclohydrolase 1	Protein Coding	P30793	51	GC14M054842	8.38274479	https://www.genecards.org/cgi-bin/carddisp.pl?gene=GCH1
KANK2	KN Motif And Ankyrin Repeat Domains 2	Protein Coding	Q63ZY3	43	GC19M017230	8.37773323	https://www.genecards.org/cgi-bin/carddisp.pl?gene=KANK2
CDH15	Cadherin 15	Protein Coding	P55291	48	GC16P089171	8.3759613	https://www.genecards.org/cgi-bin/carddisp.pl?gene=CDH15
ARF1	ADP Ribosylation Factor 1	Protein Coding	P84077	49	GC01P228082	8.34679317	https://www.genecards.org/cgi-bin/carddisp.pl?gene=ARF1
CDCP1	CUB Domain Containing Protein 1	Protein Coding	Q9H5V8	42	GC03M045082	8.34279442	https://www.genecards.org/cgi-bin/carddisp.pl?gene=CDCP1
CTNNA2	Catenin Alpha 2	Protein Coding	P26232	46	GC02P079185	8.34089375	https://www.genecards.org/cgi-bin/carddisp.pl?gene=CTNNA2
HSPB1	Heat Shock Protein Family B (Small) Member 1	Protein Coding	P04792	55	GC07P076302	8.33064556	https://www.genecards.org/cgi-bin/carddisp.pl?gene=HSPB1
FLT4	Fms Related Receptor Tyrosine Kinase 4	Protein Coding	P35916	55	GC05M182380	8.32199764	https://www.genecards.org/cgi-bin/carddisp.pl?gene=FLT4
JAK1	Janus Kinase 1	Protein Coding	P23458	57	GC01M064833	8.32056522	https://www.genecards.org/cgi-bin/carddisp.pl?gene=JAK1
MTDH	Metadherin	Protein Coding	Q86UE4	44	GC08P097644	8.31976604	https://www.genecards.org/cgi-bin/carddisp.pl?gene=MTDH
PLG	Plasminogen	Protein Coding	P00747	53	GC06P160702	8.31033802	https://www.genecards.org/cgi-bin/carddisp.pl?gene=PLG
TIAM1	TIAM Rac1 Associated GEF 1	Protein Coding	Q13009	48	GC21M031118	8.30708122	https://www.genecards.org/cgi-bin/carddisp.pl?gene=TIAM1
CAPN1	Calpain 1	Protein Coding	P07384	53	GC11P081930	8.29683685	https://www.genecards.org/cgi-bin/carddisp.pl?gene=CAPN1
PTGS2	Prostaglandin-Endoperoxide Synthase 2	Protein Coding	P35354	53	GC01M186671	8.26989555	https://www.genecards.org/cgi-bin/carddisp.pl?gene=PTGS2
DCN	Decorin	Protein Coding	P07585	51	GC12M091140	8.26745796	https://www.genecards.org/cgi-bin/carddisp.pl?gene=DCN
RAP1B	RAP1B, Member Of RAS Oncogene Family	Protein Coding	P61224	47	GC12P068610	8.26567268	https://www.genecards.org/cgi-bin/carddisp.pl?gene=RAP1B
PLEKHH2	Pleckstrin Homology, MyTH4 And FERM Domain Containing H2	Protein Coding	Q8IVE3	41	GC02P043600	8.25141907	https://www.genecards.org/cgi-bin/carddisp.pl?gene=PLEKHH2
SOD1	Superoxide Dismutase 1	Protein Coding	P00441	56	GC21P031659	8.24687958	https://www.genecards.org/cgi-bin/carddisp.pl?gene=SOD1
ADAM22	ADAM Metallopeptidase Domain 22	Protein Coding	Q9P0K1	45	GC07P087934	8.24580765	https://www.genecards.org/cgi-bin/carddisp.pl?gene=ADAM22
LAMA4	Laminin Subunit Alpha 4	Protein Coding	Q16363	49	GC06M112107	8.23276806	https://www.genecards.org/cgi-bin/carddisp.pl?gene=LAMA4
ADAM12	ADAM Metallopeptidase Domain 12	Protein Coding	O43184	49	GC10M126012	8.22742653	https://www.genecards.org/cgi-bin/carddisp.pl?gene=ADAM12
CX3CR1	C-X3-C Motif Chemokine Receptor 1	Protein Coding	P49238	46	GC03M039279	8.21930313	https://www.genecards.org/cgi-bin/carddisp.pl?gene=CX3CR1
EFNA5	Ephrin A5	Protein Coding	P52803	48	GC05M107376	8.16787815	https://www.genecards.org/cgi-bin/carddisp.pl?gene=EFNA5
LIMD1	LIM Domain Containing 1	Protein Coding	Q9UGP4	42	GC03P045555	8.15275574	https://www.genecards.org/cgi-bin/carddisp.pl?gene=LIMD1
HSP90B1	Heat Shock Protein 90 Beta Family Member 1	Protein Coding	P14625	51	GC12P103930	8.14950085	https://www.genecards.org/cgi-bin/carddisp.pl?gene=HSP90B1

GJB2	Gap Junction Protein Beta 2	Protein Coding	P29033	49	GC13M020187	8.14462566	https://www.genecards.org/cgi-bin/carddisp.pl?gene=GJB2
CDH23	Cadherin Related 23	Protein Coding	Q9H251	49	GC10P071396	8.14246655	https://www.genecards.org/cgi-bin/carddisp.pl?gene=CDH23
ACTR3	Actin Related Protein 3	Protein Coding	P61158	45	GC02P113889	8.13790607	https://www.genecards.org/cgi-bin/carddisp.pl?gene=ACTR3
MAGI2	Membrane Associated Guanylate Kinase, WW And PDZ Domain Containing 2	Protein Coding	Q86UL8	46	GC07M078017	8.13421535	https://www.genecards.org/cgi-bin/carddisp.pl?gene=MAGI2
EPHB4	EPH Receptor B4	Protein Coding	P54760	57	GC07M104513	8.13224411	https://www.genecards.org/cgi-bin/carddisp.pl?gene=EPHB4
KRT6A	Keratin 6A	Protein Coding	P02538	47	GC12M052488	8.12733173	https://www.genecards.org/cgi-bin/carddisp.pl?gene=KRT6A
REN	Renin	Protein Coding	P00797	52	GC01M204154	8.11625576	https://www.genecards.org/cgi-bin/carddisp.pl?gene=REN
CFL1	Cofilin 1	Protein Coding	P23528	50	GC11M065823	8.11473274	https://www.genecards.org/cgi-bin/carddisp.pl?gene=CFL1
BMP4	Bone Morphogenetic Protein 4	Protein Coding	P12644	53	GC14M053949	8.11399364	https://www.genecards.org/cgi-bin/carddisp.pl?gene=BMP4
NLGN1	Neuroligin 1	Protein Coding	Q8N2Q7	48	GC03P173396	8.1127882	https://www.genecards.org/cgi-bin/carddisp.pl?gene=NLGN1
KITLG	KIT Ligand	Protein Coding	P21583	51	GC12M088492	8.10717297	https://www.genecards.org/cgi-bin/carddisp.pl?gene=KITLG
TUFT1	Tuftelin 1	Protein Coding	Q9NNX1	42	GC01P158601	8.10534763	https://www.genecards.org/cgi-bin/carddisp.pl?gene=TUFT1
FOCAD	Focadhesin	Protein Coding	Q5VW36	37	GC09P020742	8.10464478	https://www.genecards.org/cgi-bin/carddisp.pl?gene=FOCAD
LGALS1	Galectin 1	Protein Coding	P09382	47	GC22P037675	8.09479332	https://www.genecards.org/cgi-bin/carddisp.pl?gene=LGALS1
TLR3	Toll Like Receptor 3	Protein Coding	O15455	54	GC04P186059	8.0893631	https://www.genecards.org/cgi-bin/carddisp.pl?gene=TLR3
EPHB2	EPH Receptor B2	Protein Coding	P29323	55	GC01P022710	8.08431816	https://www.genecards.org/cgi-bin/carddisp.pl?gene=EPHB2
CTNNA3	Catenin Alpha 3	Protein Coding	Q9UI47	45	GC10M065912	8.08012009	https://www.genecards.org/cgi-bin/carddisp.pl?gene=CTNNA3
MISP	Mitotic Spindle Positioning	Protein Coding	Q8IVT2	36	GC19P094721	8.07803345	https://www.genecards.org/cgi-bin/carddisp.pl?gene=MISP
FHL2	Four And A Half LIM Domains 2	Protein Coding	Q14192	50	GC02M105357	8.07074738	https://www.genecards.org/cgi-bin/carddisp.pl?gene=FHL2
FLOT2	Flotillin 2	Protein Coding	Q14254	44	GC17M067446	8.06339073	https://www.genecards.org/cgi-bin/carddisp.pl?gene=FLOT2
DAPK3	Death Associated Protein Kinase 3	Protein Coding	O43293	47	GC19M003958	8.05567837	https://www.genecards.org/cgi-bin/carddisp.pl?gene=DAPK3
SPG7	SPG7 Matrix AAA Peptidase Subunit, Paraplegin	Protein Coding	Q9UQ90	47	GC16P092478	8.05129814	https://www.genecards.org/cgi-bin/carddisp.pl?gene=SPG7
MYC	MYC Proto-Oncogene, BHLH Transcription Factor	Protein Coding	P01106	56	GC08P127735	8.04256439	https://www.genecards.org/cgi-bin/carddisp.pl?gene=MYC
ANXA5	Annexin A5	Protein Coding	P08758	50	GC04M121667	8.01410294	https://www.genecards.org/cgi-bin/carddisp.pl?gene=ANXA5
CD63	CD63 Molecule	Protein Coding	P08962	46	GC12M055727	8.00440884	https://www.genecards.org/cgi-bin/carddisp.pl?gene=CD63

PRKCE	Protein Kinase C Epsilon	Protein Coding	Q02156	52	GC02P045651	7.99942827	https://www.genecards.org/cgi-bin/carddisp.pl?gene=PRKCE
WNT1	Wnt Family Member 1	Protein Coding	P04628	51	GC12P051005	7.99150276	https://www.genecards.org/cgi-bin/carddisp.pl?gene=WNT1
PPARG	Peroxisome Proliferator Activated Receptor Gamma	Protein Coding	P37231	56	GC03P012287	7.99104118	https://www.genecards.org/cgi-bin/carddisp.pl?gene=PPARG
CAV2	Caveolin 2	Protein Coding	P51636	46	GC07P116287	7.98303032	https://www.genecards.org/cgi-bin/carddisp.pl?gene=CAV2
NDUFB11	NADH:Ubiquinone Oxidoreductase Subunit B11	Protein Coding	Q9NX14	44	GC0XM047142	7.97105408	https://www.genecards.org/cgi-bin/carddisp.pl?gene=NDUFB11
ANOS1	Anosmin 1	Protein Coding	P23352	44	GC0XM008528	7.96871281	https://www.genecards.org/cgi-bin/carddisp.pl?gene=ANOS1
PRICKLE1	Prickle Planar Cell Polarity Protein 1	Protein Coding	Q96MT3	48	GC12M042456	7.96568298	https://www.genecards.org/cgi-bin/carddisp.pl?gene=PRICKLE1
TNFSF11	TNF Superfamily Member 11	Protein Coding	O14788	53	GC13P042562	7.96323586	https://www.genecards.org/cgi-bin/carddisp.pl?gene=TNFSF11
DRD5	Dopamine Receptor D5	Protein Coding	P21918	50	GC04P009783	7.9577179	https://www.genecards.org/cgi-bin/carddisp.pl?gene=DRD5
FBLN7	Fibulin 7	Protein Coding	Q53RD9	41	GC02P128851	7.94371414	https://www.genecards.org/cgi-bin/carddisp.pl?gene=FBLN7
PTPRM	Protein Tyrosine Phosphatase Receptor Type M	Protein Coding	P28827	47	GC18P007557	7.93782425	https://www.genecards.org/cgi-bin/carddisp.pl?gene=PTPRM
DIAPH1	Diaphanous Related Formin 1	Protein Coding	O60610	50	GC05M141516	7.93698597	https://www.genecards.org/cgi-bin/carddisp.pl?gene=DIAPH1
COL3A1	Collagen Type III Alpha 1 Chain	Protein Coding	P02461	51	GC02P188974	7.9228363	https://www.genecards.org/cgi-bin/carddisp.pl?gene=COL3A1
AJUBA	Ajuba LIM Protein	Protein Coding	Q96IF1	44	GC14M022971	7.91798115	https://www.genecards.org/cgi-bin/carddisp.pl?gene=AJUBA
PGR	Progesterone Receptor	Protein Coding	P06401	52	GC11M117590	7.90114975	https://www.genecards.org/cgi-bin/carddisp.pl?gene=PGR
CALCA	Calcitonin Related Polypeptide Alpha	Protein Coding	P06881	47	GC11M014966	7.90066719	https://www.genecards.org/cgi-bin/carddisp.pl?gene=CALCA
KRT7	Keratin 7	Protein Coding	P08729	45	GC12P052232	7.88806343	https://www.genecards.org/cgi-bin/carddisp.pl?gene=KRT7
NRXN3	Neurexin 3	Protein Coding	Q9Y4C0	47	GC14P078170	7.87505484	https://www.genecards.org/cgi-bin/carddisp.pl?gene=NRXN3
ATP1B2	ATPase Na+/K+ Transporting Subunit Beta 2	Protein Coding	P14415	47	GC17P092378	7.85157728	https://www.genecards.org/cgi-bin/carddisp.pl?gene=ATP1B2
EMCN	Endomucin	Protein Coding	Q9ULC0	40	GC04M100395	7.84681225	https://www.genecards.org/cgi-bin/carddisp.pl?gene=EMCN
VAV3	Vav Guanine Nucleotide Exchange Factor 3	Protein Coding	Q9UKW4	46	GC01M107571	7.84275198	https://www.genecards.org/cgi-bin/carddisp.pl?gene=VAV3
TNXB	Tenascin XB	Protein Coding	P22105	47	GC06M087233	7.82258606	https://www.genecards.org/cgi-bin/carddisp.pl?gene=TNXB
PTPN1	Protein Tyrosine Phosphatase Non-Receptor Type 1	Protein Coding	P18031	53	GC20P050510	7.82194042	https://www.genecards.org/cgi-bin/carddisp.pl?gene=PTPN1
SGCE	Sarcoglycan Epsilon	Protein Coding	O43556	46	GC07M094524	7.81602383	https://www.genecards.org/cgi-bin/carddisp.pl?gene=SGCE
THBS3	Thrombospondin 3	Protein Coding	P49746	44	GC01M155195	7.8142705	https://www.genecards.org/cgi-bin/carddisp.pl?gene=THBS3

PTPN3	Protein Tyrosine Phosphatase Non-Receptor Type 3	Protein Coding	P26045	47	GC09M109375	7.81194162	https://www.genecards.org/cgi-bin/carddisp.pl?gene=PTPN3
GNB2	G Protein Subunit Beta 2	Protein Coding	P62879	47	GC07P100673	7.80134153	https://www.genecards.org/cgi-bin/carddisp.pl?gene=GNB2
MMP3	Matrix Metallopeptidase 3	Protein Coding	P08254	53	GC11M102835	7.79847288	https://www.genecards.org/cgi-bin/carddisp.pl?gene=MMP3
EPHA3	EPH Receptor A3	Protein Coding	P29320	50	GC03P089077	7.79587364	https://www.genecards.org/cgi-bin/carddisp.pl?gene=EPHA3
CLEC4M	C-Type Lectin Domain Family 4 Member M	Protein Coding	Q9H2X3	43	GC19P007763	7.78057098	https://www.genecards.org/cgi-bin/carddisp.pl?gene=CLEC4M
EPM2A	EPM2A Glucan Phosphatase, Laforin	Protein Coding	B3EWF7	46	GC06M145382	7.78027296	https://www.genecards.org/cgi-bin/carddisp.pl?gene=EPM2A
SUFU	SUFU Negative Regulator Of Hedgehog Signaling	Protein Coding	Q9UMX1	47	GC10P104919	7.77584219	https://www.genecards.org/cgi-bin/carddisp.pl?gene=SUFU
ADAMTS13	ADAM Metallopeptidase With Thrombospondin Type 1 Motif 13	Protein Coding	Q76LX8	51	GC09P133414	7.76171303	https://www.genecards.org/cgi-bin/carddisp.pl?gene=ADAMTS13
CNTN1	Contactin 1	Protein Coding	Q12860	50	GC12P040692	7.75446463	https://www.genecards.org/cgi-bin/carddisp.pl?gene=CNTN1
NLGN3	Neuroligin 3	Protein Coding	Q9NZ94	47	GC0XP071144	7.75319767	https://www.genecards.org/cgi-bin/carddisp.pl?gene=NLGN3
CD40	CD40 Molecule	Protein Coding	P25942	54	GC20P046118	7.74203014	https://www.genecards.org/cgi-bin/carddisp.pl?gene=CD40
SDHA	Succinate Dehydrogenase Complex Flavoprotein Subunit A	Protein Coding	P31040	52	GC05P000220	7.7405591	https://www.genecards.org/cgi-bin/carddisp.pl?gene=SDHA
COMP	Cartilage Oligomeric Matrix Protein	Protein Coding	P49747	50	GC19M018783	7.72659779	https://www.genecards.org/cgi-bin/carddisp.pl?gene=COMP
KNG1	Kininogen 1	Protein Coding	P01042	52	GC03P186717	7.72515726	https://www.genecards.org/cgi-bin/carddisp.pl?gene=KNG1
MIR7-3HG	MIR7-3 Host Gene	RNA Gene	Q8N6C7	27	GC19P094936	7.72038364	https://www.genecards.org/cgi-bin/carddisp.pl?gene=MIR7-3HG
CCN3	Cellular Communication Network Factor 3	Protein Coding	P48745	44	GC08P119416	7.71192551	https://www.genecards.org/cgi-bin/carddisp.pl?gene=CCN3
LRP1	LDL Receptor Related Protein 1	Protein Coding	Q07954	52	GC12P057128	7.6918869	https://www.genecards.org/cgi-bin/carddisp.pl?gene=LRP1
MIF	Macrophage Migration Inhibitory Factor	Protein Coding	P14174	51	GC22P023894	7.69079685	https://www.genecards.org/cgi-bin/carddisp.pl?gene=MIF
BMP6	Bone Morphogenetic Protein 6	Protein Coding	P22004	48	GC06P007726	7.68323994	https://www.genecards.org/cgi-bin/carddisp.pl?gene=BMP6
SDC1	Syndecan 1	Protein Coding	P18827	46	GC02M020200	7.67429686	https://www.genecards.org/cgi-bin/carddisp.pl?gene=SDC1
MDM2	MDM2 Proto-Oncogene	Protein Coding	Q00987	57	GC12P068808	7.6656723	https://www.genecards.org/cgi-bin/carddisp.pl?gene=MDM2
F2	Coagulation Factor II, Thrombin	Protein Coding	P00734	53	GC11P047208	7.66337442	https://www.genecards.org/cgi-bin/carddisp.pl?gene=F2
SORBS2	Sorbin And SH3 Domain Containing 2	Protein Coding	O94875	45	GC04M185585	7.65657568	https://www.genecards.org/cgi-bin/carddisp.pl?gene=SORBS2
PPP1CA	Protein Phosphatase 1 Catalytic Subunit Alpha	Protein Coding	P62136	50	GC11M116984	7.65225697	https://www.genecards.org/cgi-bin/carddisp.pl?gene=PPP1CA
BMP7	Bone Morphogenetic Protein 7	Protein Coding	P18075	50	GC20M057168	7.64922905	https://www.genecards.org/cgi-bin/carddisp.pl?gene=BMP7

PRKCB	Protein Kinase C Beta	Protein Coding	P05771	52	GC16P060465	7.64250231	https://www.genecards.org/cgi-bin/carddisp.pl?gene=PRKCB
DOCK7	Dedicator Of Cytokinesis 7	Protein Coding	Q96N67	46	GC01M062454	7.63717222	https://www.genecards.org/cgi-bin/carddisp.pl?gene=DOCK7
RHOB	Ras Homolog Family Member B	Protein Coding	P62745	48	GC02P020447	7.62153864	https://www.genecards.org/cgi-bin/carddisp.pl?gene=RHOB
PAK2	P21 (RAC1) Activated Kinase 2	Protein Coding	Q13177	52	GC03P196739	7.59932518	https://www.genecards.org/cgi-bin/carddisp.pl?gene=PAK2
SLC9A1	Solute Carrier Family 9 Member A1	Protein Coding	P19634	53	GC01M030165	7.58357143	https://www.genecards.org/cgi-bin/carddisp.pl?gene=SLC9A1
CD81	CD81 Molecule	Protein Coding	P60033	50	GC11P005810	7.58330154	https://www.genecards.org/cgi-bin/carddisp.pl?gene=CD81
GABRB3	Gamma-Aminobutyric Acid Type A Receptor Subunit Beta3	Protein Coding	P28472	52	GC15M026543	7.5829854	https://www.genecards.org/cgi-bin/carddisp.pl?gene=GABRB3
EFNA1	Ephrin A1	Protein Coding	P20827	47	GC01P155127	7.57839489	https://www.genecards.org/cgi-bin/carddisp.pl?gene=EFNA1
ARPC2	Actin Related Protein 2/3 Complex Subunit 2	Protein Coding	O15144	46	GC02P218217	7.56661987	https://www.genecards.org/cgi-bin/carddisp.pl?gene=ARPC2
MPZL1	Myelin Protein Zero Like 1	Protein Coding	O95297	44	GC01P167721	7.56633997	https://www.genecards.org/cgi-bin/carddisp.pl?gene=MPZL1
MAPK8IP3	Mitogen-Activated Protein Kinase 8 Interacting Protein 3	Protein Coding	Q9UPT6	45	GC16P001706	7.54339409	https://www.genecards.org/cgi-bin/carddisp.pl?gene=MAPK8IP3
RND3	Rho Family GTPase 3	Protein Coding	P61587	45	GC02M150468	7.51858807	https://www.genecards.org/cgi-bin/carddisp.pl?gene=RND3
PDGFA	Platelet Derived Growth Factor Subunit A	Protein Coding	P04085	47	GC07M000497	7.51438951	https://www.genecards.org/cgi-bin/carddisp.pl?gene=PDGFA
ETS1	ETS Proto-Oncogene 1, Transcription Factor	Protein Coding	P14921	51	GC11M128458	7.50561237	https://www.genecards.org/cgi-bin/carddisp.pl?gene=ETS1
BCR	BCR Activator Of RhoGEF And GTPase	Protein Coding	P11274	55	GC22P023179	7.5007658	https://www.genecards.org/cgi-bin/carddisp.pl?gene=BCR
SOS1	SOS Ras/Rac Guanine Nucleotide Exchange Factor 1	Protein Coding	Q07889	54	GC02M039197	7.49663019	https://www.genecards.org/cgi-bin/carddisp.pl?gene=SOS1
FHL1	Four And A Half LIM Domains 1	Protein Coding	Q13642	48	GC0XP136146	7.49615955	https://www.genecards.org/cgi-bin/carddisp.pl?gene=FHL1
MAG	Myelin Associated Glycoprotein	Protein Coding	P20916	51	GC19P035292	7.48227501	https://www.genecards.org/cgi-bin/carddisp.pl?gene=MAG
VCAN	Versican	Protein Coding	P13611	51	GC05P083471	7.48012877	https://www.genecards.org/cgi-bin/carddisp.pl?gene=VCAN
SIGLEC7	Sialic Acid Binding Ig Like Lectin 7	Protein Coding	Q9Y286	46	GC19P051142	7.4789896	https://www.genecards.org/cgi-bin/carddisp.pl?gene=SIGLEC7
CCNA2	Cyclin A2	Protein Coding	P20248	48	GC04M121816	7.47177696	https://www.genecards.org/cgi-bin/carddisp.pl?gene=CCNA2
STAT1	Signal Transducer And Activator Of Transcription 1	Protein Coding	P42224	56	GC02M190908	7.46247768	https://www.genecards.org/cgi-bin/carddisp.pl?gene=STAT1
DSC1	Desmocollin 1	Protein Coding	Q08554	42	GC18M031129	7.45032024	https://www.genecards.org/cgi-bin/carddisp.pl?gene=DSC1
WNT5A	Wnt Family Member 5A	Protein Coding	P41221	54	GC03M055465	7.44844341	https://www.genecards.org/cgi-bin/carddisp.pl?gene=WNT5A
FBN1	Fibrillin 1	Protein Coding	P35555	51	GC15M048408	7.43830776	https://www.genecards.org/cgi-bin/carddisp.pl?gene=FBN1

MIR21	MicroRNA 21	RNA Gene		25	GC17P0 94092	7.43179512	https://www.genecards.org/cgi-bin/carddisp.pl?gene=MIR21
PFN1	Profilin 1	Protein Coding	P07737	50	GC17M0 04945	7.42264509	https://www.genecards.org/cgi-bin/carddisp.pl?gene=PFN1
DSG4	Desmoglein 4	Protein Coding	Q86SJ6	45	GC18P0 31377	7.41059923	https://www.genecards.org/cgi-bin/carddisp.pl?gene=DSG4
PSTPIP1	Proline-Serine-Threonine Phosphatase Interacting Protein 1	Protein Coding	O43586	47	GC15P0 76993	7.40396595	https://www.genecards.org/cgi-bin/carddisp.pl?gene=PSTPIP1
CCR5	C-C Motif Chemokine Receptor 5	Protein Coding	P51681	50	GC03P0 54799	7.4017477	https://www.genecards.org/cgi-bin/carddisp.pl?gene=CCR5
MYLK2	Myosin Light Chain Kinase 2	Protein Coding	Q9H1R3	50	GC20P0 31819	7.39897251	https://www.genecards.org/cgi-bin/carddisp.pl?gene=MYLK2
NINJ1	Ninjurin 1	Protein Coding	Q92982	45	GC09M0 93121	7.3861351	https://www.genecards.org/cgi-bin/carddisp.pl?gene=NINJ1
FYB1	FYN Binding Protein 1	Protein Coding	O15117	46	GC05M0 39105	7.37160587	https://www.genecards.org/cgi-bin/carddisp.pl?gene=FYB1
DCC	DCC Netrin 1 Receptor	Protein Coding	P43146	50	GC18P0 52340	7.37094402	https://www.genecards.org/cgi-bin/carddisp.pl?gene=DCC
STARD8	StAR Related Lipid Transfer Domain Containing 8	Protein Coding	Q92502	41	GC0XP0 68647	7.36850262	https://www.genecards.org/cgi-bin/carddisp.pl?gene=STARD8
IGFBP7	Insulin Like Growth Factor Binding Protein 7	Protein Coding	Q16270	50	GC04M0 57030	7.36764717	https://www.genecards.org/cgi-bin/carddisp.pl?gene=IGFBP7
GP1BB	Glycoprotein Ib Platelet Subunit Beta	Protein Coding	P13224	47	GC22P0 60235	7.36657858	https://www.genecards.org/cgi-bin/carddisp.pl?gene=GP1BB
PNKD	PNKD Metallo-Beta-Lactamase Domain Containing	Protein Coding	Q8N490	45	GC02P2 18270	7.36289597	https://www.genecards.org/cgi-bin/carddisp.pl?gene=PNKD
ARHGAP5	Rho GTPase Activating Protein 5	Protein Coding	Q13017	46	GC14P0 32076	7.35947704	https://www.genecards.org/cgi-bin/carddisp.pl?gene=ARHGAP 5
RPS6KB1	Ribosomal Protein S6 Kinase B1	Protein Coding	P23443	53	GC17P0 59893	7.3590107	https://www.genecards.org/cgi-bin/carddisp.pl?gene=RPS6KB1
KRT19	Keratin 19	Protein Coding	P08727	47	GC17M0 41523	7.33779335	https://www.genecards.org/cgi-bin/carddisp.pl?gene=KRT19
SLC6A1	Solute Carrier Family 6 Member 1	Protein Coding	P30531	50	GC03P0 19297	7.33713055	https://www.genecards.org/cgi-bin/carddisp.pl?gene=SLC6A1
CELSR2	Cadherin EGF LAG Seven-Pass G-Type Receptor 2	Protein Coding	Q9HCU 4	45	GC01P1 09286	7.33395386	https://www.genecards.org/cgi-bin/carddisp.pl?gene=CELSR2
SBF1	SET Binding Factor 1	Protein Coding	O95248	45	GC22M0 72797	7.33378458	https://www.genecards.org/cgi-bin/carddisp.pl?gene=SBF1
CSTA	Cystatin A	Protein Coding	P01040	47	GC03P1 22325	7.32753468	https://www.genecards.org/cgi-bin/carddisp.pl?gene=CSTA
FUT7	Fucosyltransferase 7	Protein Coding	Q11130	45	GC09M1 37030	7.3270483	https://www.genecards.org/cgi-bin/carddisp.pl?gene=FUT7
ARHGAP10	Rho GTPase Activating Protein 10	Protein Coding	A1A4S6	42	GC04P1 47732	7.32450294	https://www.genecards.org/cgi-bin/carddisp.pl?gene=ARHGAP 10
MAPK7	Mitogen-Activated Protein Kinase 7	Protein Coding	Q13164	51	GC17P0 92919	7.32313156	https://www.genecards.org/cgi-bin/carddisp.pl?gene=MAPK7
CYTH1	Cytohesin 1	Protein Coding	Q15438	45	GC17M0 78674	7.31981707	https://www.genecards.org/cgi-bin/carddisp.pl?gene=CYTH1
IL1RAPL2	Interleukin 1 Receptor Accessory Protein Like 2	Protein Coding	Q9NP60	42	GC0XP1 04566	7.31443882	https://www.genecards.org/cgi-bin/carddisp.pl?gene=IL1RAPL2

PRNP	Prion Protein	Protein Coding	F7VJQ1	52	GC20P004686	7.31143284	https://www.genecards.org/cgi-bin/carddisp.pl?gene=PRNP
PSEN1	Presenilin 1	Protein Coding	P49768	55	GC14P073136	7.31049824	https://www.genecards.org/cgi-bin/carddisp.pl?gene=PSEN1
KRT17	Keratin 17	Protein Coding	Q04695	48	GC17M041619	7.30809259	https://www.genecards.org/cgi-bin/carddisp.pl?gene=KRT17
MBP	Myelin Basic Protein	Protein Coding	P02686	48	GC18M076978	7.29292154	https://www.genecards.org/cgi-bin/carddisp.pl?gene=MBP
NEXN	Nexilin F-Actin Binding Protein	Protein Coding	Q0ZGT2	43	GC01P077944	7.29247952	https://www.genecards.org/cgi-bin/carddisp.pl?gene=NEXN
FOS	Fos Proto-Oncogene, AP-1 Transcription Factor Subunit	Protein Coding	P01100	54	GC14P075278	7.29242659	https://www.genecards.org/cgi-bin/carddisp.pl?gene=FOS
IRF6	Interferon Regulatory Factor 6	Protein Coding	O14896	46	GC01M209785	7.28815365	https://www.genecards.org/cgi-bin/carddisp.pl?gene=IRF6
CLMP	CXADR Like Membrane Protein	Protein Coding	Q9H6B4	45	GC11M123069	7.28395271	https://www.genecards.org/cgi-bin/carddisp.pl?gene=CLMP
CDH17	Cadherin 17	Protein Coding	Q12864	46	GC08M094127	7.28224993	https://www.genecards.org/cgi-bin/carddisp.pl?gene=CDH17
PPP1CC	Protein Phosphatase 1 Catalytic Subunit Gamma	Protein Coding	P36873	49	GC12M110709	7.27948427	https://www.genecards.org/cgi-bin/carddisp.pl?gene=PPP1CC
SLK	STE20 Like Kinase	Protein Coding	Q9H2G2	47	GC10P103967	7.27622986	https://www.genecards.org/cgi-bin/carddisp.pl?gene=SLK
PKP1	Plakophilin 1	Protein Coding	Q13835	45	GC01P201283	7.24380541	https://www.genecards.org/cgi-bin/carddisp.pl?gene=PKP1
RAB21	RAB21, Member RAS Oncogene Family	Protein Coding	Q9UL25	41	GC12P071754	7.23922396	https://www.genecards.org/cgi-bin/carddisp.pl?gene=RAB21
NEO1	Neogenin 1	Protein Coding	Q92859	46	GC15P073051	7.23758888	https://www.genecards.org/cgi-bin/carddisp.pl?gene=NEO1
IKBKB	Inhibitor Of Nuclear Factor Kappa B Kinase Subunit Beta	Protein Coding	O14920	57	GC08P042271	7.23559189	https://www.genecards.org/cgi-bin/carddisp.pl?gene=IKBKB
CD40LG	CD40 Ligand	Protein Coding	P29965	53	GC0XP136649	7.23067856	https://www.genecards.org/cgi-bin/carddisp.pl?gene=CD40LG
GNA13	G Protein Subunit Alpha 13	Protein Coding	Q14344	48	GC17M065009	7.22970104	https://www.genecards.org/cgi-bin/carddisp.pl?gene=GNA13
EFNB1	Ephrin B1	Protein Coding	P98172	50	GC0XP068828	7.21229076	https://www.genecards.org/cgi-bin/carddisp.pl?gene=EFNB1
DAAM2	Dishevelled Associated Activator Of Morphogenesis 2	Protein Coding	Q86T65	43	GC06P039792	7.2075038	https://www.genecards.org/cgi-bin/carddisp.pl?gene=DAAM2
ARHGEF28	Rho Guanine Nucleotide Exchange Factor 28	Protein Coding	Q8N1W1	40	GC05P073626	7.17286587	https://www.genecards.org/cgi-bin/carddisp.pl?gene=ARHGEF28
GNAI1	G Protein Subunit Alpha I1	Protein Coding	P63096	51	GC07P082939	7.16458035	https://www.genecards.org/cgi-bin/carddisp.pl?gene=GNAI1
PTCH1	Patched 1	Protein Coding	Q13635	54	GC09M095442	7.15652514	https://www.genecards.org/cgi-bin/carddisp.pl?gene=PTCH1
PDGFRA	Platelet Derived Growth Factor Receptor Alpha	Protein Coding	P16234	57	GC04P054229	7.14797401	https://www.genecards.org/cgi-bin/carddisp.pl?gene=PDGFRA
CDKN2A	Cyclin Dependent Kinase Inhibitor 2A	Protein Coding	Q8N726	54	GC09M021967	7.14327288	https://www.genecards.org/cgi-bin/carddisp.pl?gene=CDKN2A
CTSL	Cathepsin L	Protein Coding	P07711	51	GC09P087725	7.12631464	https://www.genecards.org/cgi-bin/carddisp.pl?gene=CTSL

FGF13	Fibroblast Growth Factor 13	Protein Coding	Q92913	46	GC0XM138615	7.1216898	https://www.genecards.org/cgi-bin/carddisp.pl?gene=FGF13
SDHB	Succinate Dehydrogenase Complex Iron Sulfur Subunit B	Protein Coding	P21912	52	GC01M018919	7.12136745	https://www.genecards.org/cgi-bin/carddisp.pl?gene=SDHB
ANGPT2	Angiopoietin 2	Protein Coding	O15123	52	GC08M006499	7.11881733	https://www.genecards.org/cgi-bin/carddisp.pl?gene=ANGPT2
FSCN1	Fascin Actin-Bundling Protein 1	Protein Coding	Q16658	47	GC07P005592	7.11852026	https://www.genecards.org/cgi-bin/carddisp.pl?gene=FSCN1
GRIA3	Glutamate Ionotropic Receptor AMPA Type Subunit 3	Protein Coding	P42263	53	GC0XP123184	7.11828995	https://www.genecards.org/cgi-bin/carddisp.pl?gene=GRIA3
EMSLR	E2F1 MRNA Stabilizing LncRNA	RNA Gene		14	GC07P104345	7.11704731	https://www.genecards.org/cgi-bin/carddisp.pl?gene=EMSLR
MTHFR	Methylenetetrahydrofolate Reductase	Protein Coding	P42898	51	GC01M011785	7.09840775	https://www.genecards.org/cgi-bin/carddisp.pl?gene=MTHFR
DCX	Doublecortin	Protein Coding	O43602	48	GC0XM111293	7.09588528	https://www.genecards.org/cgi-bin/carddisp.pl?gene=DCX
RPSA	Ribosomal Protein SA	Protein Coding	P08865	50	GC03P039406	7.09179783	https://www.genecards.org/cgi-bin/carddisp.pl?gene=RPSA
TERT	Telomerase Reverse Transcriptase	Protein Coding	O14746	55	GC05M001253	7.08672619	https://www.genecards.org/cgi-bin/carddisp.pl?gene=TERT
CDKN1C	Cyclin Dependent Kinase Inhibitor 1C	Protein Coding	P49918	48	GC11M009679	7.07707739	https://www.genecards.org/cgi-bin/carddisp.pl?gene=CDKN1C
CDH12	Cadherin 12	Protein Coding	P55289	45	GC05M021750	7.07505798	https://www.genecards.org/cgi-bin/carddisp.pl?gene=CDH12
AQP4	Aquaporin 4	Protein Coding	P55087	50	GC18M026852	7.07347488	https://www.genecards.org/cgi-bin/carddisp.pl?gene=AQP4
CELSR3	Cadherin EGF LAG Seven-Pass G-Type Receptor 3	Protein Coding	Q9NYQ7	42	GC03M048641	7.0714488	https://www.genecards.org/cgi-bin/carddisp.pl?gene=CELSR3
ARPC3	Actin Related Protein 2/3 Complex Subunit 3	Protein Coding	O15145	47	GC12M110434	7.06594753	https://www.genecards.org/cgi-bin/carddisp.pl?gene=ARPC3
NRG1	Neuregulin 1	Protein Coding	Q02297	52	GC08P031639	7.05895424	https://www.genecards.org/cgi-bin/carddisp.pl?gene=NRG1
TBK1	TANK Binding Kinase 1	Protein Coding	Q9UHD2	52	GC12P064451	7.05798769	https://www.genecards.org/cgi-bin/carddisp.pl?gene=TBK1
LMNA	Lamin A/C	Protein Coding	P02545	53	GC01P156082	7.04870415	https://www.genecards.org/cgi-bin/carddisp.pl?gene=LMNA
SRCIN1	SRC Kinase Signaling Inhibitor 1	Protein Coding	Q9C0H9	39	GC17M038530	7.04348898	https://www.genecards.org/cgi-bin/carddisp.pl?gene=SRCIN1
FLRT3	Fibronectin Leucine Rich Transmembrane Protein 3	Protein Coding	Q9NZU0	48	GC20M014322	7.03571892	https://www.genecards.org/cgi-bin/carddisp.pl?gene=FLRT3
CSF2	Colony Stimulating Factor 2	Protein Coding	P04141	48	GC05P132073	7.03548384	https://www.genecards.org/cgi-bin/carddisp.pl?gene=CSF2
AFAP1	Actin Filament Associated Protein 1	Protein Coding	Q8N556	42	GC04M007758	7.03202009	https://www.genecards.org/cgi-bin/carddisp.pl?gene=AFAP1
GSN	Gelsolin	Protein Coding	P06396	52	GC09P121201	7.02821064	https://www.genecards.org/cgi-bin/carddisp.pl?gene=GSN
LAYN	Layilin	Protein Coding	Q6UX15	39	GC11P112286	7.02640152	https://www.genecards.org/cgi-bin/carddisp.pl?gene=LAYN
DAB2	DAB Adaptor Protein 2	Protein Coding	P98082	47	GC05M039371	7.02568102	https://www.genecards.org/cgi-bin/carddisp.pl?gene=DAB2

HSPA8	Heat Shock Protein Family A (Hsp70) Member 8	Protein Coding	P11142	53	GC11M123057	7.02191734	https://www.genecards.org/cgi-bin/carddisp.pl?gene=HSPA8
ELN	Elastin	Protein Coding	P15502	47	GC07P074027	7.0202651	https://www.genecards.org/cgi-bin/carddisp.pl?gene=ELN
PACS2	Phosphofurin Acidic Cluster Sorting Protein 2	Protein Coding	Q86VP3	44	GC14P105300	7.00493193	https://www.genecards.org/cgi-bin/carddisp.pl?gene=PACS2
GLI3	GLI Family Zinc Finger 3	Protein Coding	P10071	52	GC07M041960	6.99610138	https://www.genecards.org/cgi-bin/carddisp.pl?gene=GLI3
HRG	Histidine Rich Glycoprotein	Protein Coding	P04196	49	GC03P186660	6.99565125	https://www.genecards.org/cgi-bin/carddisp.pl?gene=HRG
CCM2	CCM2 Scaffold Protein	Protein Coding	Q9BSQ5	46	GC07P044999	6.99491024	https://www.genecards.org/cgi-bin/carddisp.pl?gene=CCM2
RAB10	RAB10, Member RAS Oncogene Family	Protein Coding	P61026	45	GC02P026033	6.99362183	https://www.genecards.org/cgi-bin/carddisp.pl?gene=RAB10
ARHGEF2	Rho/Rac Guanine Nucleotide Exchange Factor 2	Protein Coding	Q92974	50	GC01M155946	6.99171591	https://www.genecards.org/cgi-bin/carddisp.pl?gene=ARHGEF2
SBF2-AS1	SBF2 Antisense RNA 1	RNA Gene		18	GC11P009758	6.97901297	https://www.genecards.org/cgi-bin/carddisp.pl?gene=SBF2-AS1
PLAT	Plasminogen Activator, Tissue Type	Protein Coding	P00750	52	GC08M042174	6.97690725	https://www.genecards.org/cgi-bin/carddisp.pl?gene=PLAT
STK11	Serine/Threonine Kinase 11	Protein Coding	Q15831	54	GC19P001177	6.94418049	https://www.genecards.org/cgi-bin/carddisp.pl?gene=STK11
IL3	Interleukin 3	Protein Coding	P08700	48	GC05P132060	6.93048668	https://www.genecards.org/cgi-bin/carddisp.pl?gene=IL3
EPHB6	EPH Receptor B6	Protein Coding	O15197	48	GC07P154340	6.90920734	https://www.genecards.org/cgi-bin/carddisp.pl?gene=EPHB6
ST3GAL3	ST3 Beta-Galactoside Alpha-2,3-Sialyltransferase 3	Protein Coding	Q11203	49	GC01P043705	6.90653801	https://www.genecards.org/cgi-bin/carddisp.pl?gene=ST3GAL3
BDNF	Brain Derived Neurotrophic Factor	Protein Coding	P23560	53	GC11M027654	6.90039063	https://www.genecards.org/cgi-bin/carddisp.pl?gene=BDNF
WWOX	WW Domain Containing Oxidoreductase	Protein Coding	Q9NZC7	49	GC16P078099	6.89933252	https://www.genecards.org/cgi-bin/carddisp.pl?gene=WWOX
NCK2	NCK Adaptor Protein 2	Protein Coding	O43639	46	GC02P105744	6.88579035	https://www.genecards.org/cgi-bin/carddisp.pl?gene=NCK2
ZAP70	Zeta Chain Of T Cell Receptor Associated Protein Kinase 70	Protein Coding	P43403	55	GC02P097873	6.88250113	https://www.genecards.org/cgi-bin/carddisp.pl?gene=ZAP70
MAP2K2	Mitogen-Activated Protein Kinase Kinase 2	Protein Coding	P36507	58	GC19M004090	6.85557175	https://www.genecards.org/cgi-bin/carddisp.pl?gene=MAP2K2
ACTN2	Actinin Alpha 2	Protein Coding	P35609	50	GC01P236686	6.85302305	https://www.genecards.org/cgi-bin/carddisp.pl?gene=ACTN2
ARPC5	Actin Related Protein 2/3 Complex Subunit 5	Protein Coding	O15511	44	GC01M185260	6.83820677	https://www.genecards.org/cgi-bin/carddisp.pl?gene=ARPC5
TUBB	Tubulin Beta Class I	Protein Coding	P07437	53	GC06P118873	6.83813572	https://www.genecards.org/cgi-bin/carddisp.pl?gene=TUBB
SEMA3A	Semaphorin 3A	Protein Coding	Q14563	50	GC07M083955	6.83022213	https://www.genecards.org/cgi-bin/carddisp.pl?gene=SEMA3A
YES1	YES Proto-Oncogene 1, Src Family Tyrosine Kinase	Protein Coding	P07947	51	GC18M000721	6.82981873	https://www.genecards.org/cgi-bin/carddisp.pl?gene=YES1
MSLN	Mesothelin	Protein Coding	Q13421	47	GC16P059755	6.82978344	https://www.genecards.org/cgi-bin/carddisp.pl?gene=MSLN

ANXA2	Annexin A2	Protein Coding	P07355	51	GC15M060347	6.82550812	https://www.genecards.org/cgi-bin/carddisp.pl?gene=ANXA2
MARCKS	Myristoylated Alanine Rich Protein Kinase C Substrate	Protein Coding	P29966	44	GC06P113857	6.8244772	https://www.genecards.org/cgi-bin/carddisp.pl?gene=MARCKS
RAPGEF2	Rap Guanine Nucleotide Exchange Factor 2	Protein Coding	Q9Y4G8	45	GC04P159106	6.81142378	https://www.genecards.org/cgi-bin/carddisp.pl?gene=RAPGEF2
HNF4A	Hepatocyte Nuclear Factor 4 Alpha	Protein Coding	P41235	53	GC20P044355	6.80645037	https://www.genecards.org/cgi-bin/carddisp.pl?gene=HNF4A
MYLK3	Myosin Light Chain Kinase 3	Protein Coding	Q32MK0	45	GC16M047228	6.80057192	https://www.genecards.org/cgi-bin/carddisp.pl?gene=MYLK3
MYO9A	Myosin IXA	Protein Coding	B2RTY4	42	GC15M071822	6.79762554	https://www.genecards.org/cgi-bin/carddisp.pl?gene=MYO9A
MMP13	Matrix Metallopeptidase 13	Protein Coding	P45452	53	GC11M102942	6.79472303	https://www.genecards.org/cgi-bin/carddisp.pl?gene=MMP13
YAP1	Yes1 Associated Transcriptional Regulator	Protein Coding	P46937	51	GC11P102110	6.79462385	https://www.genecards.org/cgi-bin/carddisp.pl?gene=YAP1
NCKAP1	NCK Associated Protein 1	Protein Coding	Q9Y2A7	43	GC02M182909	6.79104757	https://www.genecards.org/cgi-bin/carddisp.pl?gene=NCKAP1
TP63	Tumor Protein P63	Protein Coding	Q9H3D4	50	GC03P189598	6.78833866	https://www.genecards.org/cgi-bin/carddisp.pl?gene=TP63
FMN1	Formin 1	Protein Coding	Q68DA7	42	GC15M032765	6.77647209	https://www.genecards.org/cgi-bin/carddisp.pl?gene=FMN1
NOX4	NADPH Oxidase 4	Protein Coding	Q9NPH5	46	GC11M089324	6.77162266	https://www.genecards.org/cgi-bin/carddisp.pl?gene=NOX4
IL18	Interleukin 18	Protein Coding	Q14116	47	GC11M112143	6.76911211	https://www.genecards.org/cgi-bin/carddisp.pl?gene=IL18
RSU1	Ras Suppressor Protein 1	Protein Coding	Q15404	44	GC10M016672	6.75992632	https://www.genecards.org/cgi-bin/carddisp.pl?gene=RSU1
LIMA1	LIM Domain And Actin Binding 1	Protein Coding	Q9UHB6	44	GC12M050175	6.75733328	https://www.genecards.org/cgi-bin/carddisp.pl?gene=LIMA1
PAFAH1B1	Platelet Activating Factor Acetylhydrolase 1b Regulatory Subunit 1	Protein Coding	P43034	50	GC17P002593	6.75493622	https://www.genecards.org/cgi-bin/carddisp.pl?gene=PAFAH1B1
MT-ND1	Mitochondrially Encoded NADH:Ubiquinone Oxidoreductase Core Subunit 1	Protein Coding	P03886	34	GCMTP003309	6.75302076	https://www.genecards.org/cgi-bin/carddisp.pl?gene=MT-ND1
PDPN	Podoplanin	Protein Coding	Q86YL7	45	GC01P013583	6.75284624	https://www.genecards.org/cgi-bin/carddisp.pl?gene=PDPN
HSPA5	Heat Shock Protein Family A (Hsp70) Member 5	Protein Coding	P11021	52	GC09M125234	6.7506361	https://www.genecards.org/cgi-bin/carddisp.pl?gene=HSPA5
ARHGEF11	Rho Guanine Nucleotide Exchange Factor 11	Protein Coding	O15085	45	GC01M156934	6.74910879	https://www.genecards.org/cgi-bin/carddisp.pl?gene=ARHGEF11
SEPTIN9	Septin 9	Protein Coding	Q9UHD8	45	GC17P094545	6.73883724	https://www.genecards.org/cgi-bin/carddisp.pl?gene=SEPTIN9
FAP	Fibroblast Activation Protein Alpha	Protein Coding	Q12884	50	GC02M162170	6.7359848	https://www.genecards.org/cgi-bin/carddisp.pl?gene=FAP
NOC3L	NOC3 Like DNA Replication Regulator	Protein Coding	Q8WTT2	40	GC10M094333	6.73078251	https://www.genecards.org/cgi-bin/carddisp.pl?gene=NOC3L
PRUNE1	Prune Exopolyphosphatase 1	Protein Coding	Q86TP1	44	GC01P151008	6.7298069	https://www.genecards.org/cgi-bin/carddisp.pl?gene=PRUNE1
IFNB1	Interferon Beta 1	Protein Coding	P01574	47	GC09M021077	6.72644711	https://www.genecards.org/cgi-bin/carddisp.pl?gene=IFNB1

SIK1	Salt Inducible Kinase 1	Protein Coding	P57059	50	GC21M043414	6.72145987	https://www.genecards.org/cgi-bin/carddisp.pl?gene=SIK1
CAT	Catalase	Protein Coding	P04040	55	GC11P034460	6.71817017	https://www.genecards.org/cgi-bin/carddisp.pl?gene=CAT
PPP6R2	Protein Phosphatase 6 Regulatory Subunit 2	Protein Coding	O75170	41	GC22P050330	6.71515274	https://www.genecards.org/cgi-bin/carddisp.pl?gene=PPP6R2
CD79A	CD79a Molecule	Protein Coding	P11912	51	GC19P041877	6.7139678	https://www.genecards.org/cgi-bin/carddisp.pl?gene=CD79A
DLG4	Discs Large MAGUK Scaffold Protein 4	Protein Coding	P78352	51	GC17M015314	6.69058895	https://www.genecards.org/cgi-bin/carddisp.pl?gene=DLG4
TLR2	Toll Like Receptor 2	Protein Coding	O60603	55	GC04P153684	6.68681955	https://www.genecards.org/cgi-bin/carddisp.pl?gene=TLR2
PLXNB1	Plexin B1	Protein Coding	O43157	46	GC03M048403	6.68231249	https://www.genecards.org/cgi-bin/carddisp.pl?gene=PLXNB1
EDNRB	Endothelin Receptor Type B	Protein Coding	P24530	53	GC13M077895	6.6804781	https://www.genecards.org/cgi-bin/carddisp.pl?gene=EDNRB
GJB1	Gap Junction Protein Beta 1	Protein Coding	P08034	50	GC0XP071212	6.66955471	https://www.genecards.org/cgi-bin/carddisp.pl?gene=GJB1
CSPG4	Chondroitin Sulfate Proteoglycan 4	Protein Coding	Q6UVK1	50	GC15M075674	6.66530323	https://www.genecards.org/cgi-bin/carddisp.pl?gene=CSPG4
ARPC1B	Actin Related Protein 2/3 Complex Subunit 1B	Protein Coding	O15143	47	GC07P099374	6.66132545	https://www.genecards.org/cgi-bin/carddisp.pl?gene=ARPC1B
OCLN	Occludin	Protein Coding	Q16625	48	GC05P069492	6.65785789	https://www.genecards.org/cgi-bin/carddisp.pl?gene=OCLN
KRT2	Keratin 2	Protein Coding	P35908	45	GC12M052757	6.65414715	https://www.genecards.org/cgi-bin/carddisp.pl?gene=KRT2
SYNGAP1	Synaptic Ras GTPase Activating Protein 1	Protein Coding	Q96PV0	51	GC06P118990	6.63766241	https://www.genecards.org/cgi-bin/carddisp.pl?gene=SYNGAP1
PAK3	P21 (RAC1) Activated Kinase 3	Protein Coding	O75914	52	GC0XP110944	6.63586617	https://www.genecards.org/cgi-bin/carddisp.pl?gene=PAK3
LIMCH1	LIM And Calponin Homology Domains 1	Protein Coding	Q9UPQ0	39	GC04P041362	6.6229744	https://www.genecards.org/cgi-bin/carddisp.pl?gene=LIMCH1
CD28	CD28 Molecule	Protein Coding	P10747	51	GC02P203706	6.62181234	https://www.genecards.org/cgi-bin/carddisp.pl?gene=CD28
NGF	Nerve Growth Factor	Protein Coding	P01138	54	GC01M115285	6.61443663	https://www.genecards.org/cgi-bin/carddisp.pl?gene=NGF
ESR2	Estrogen Receptor 2	Protein Coding	Q92731	52	GC14M064084	6.6121397	https://www.genecards.org/cgi-bin/carddisp.pl?gene=ESR2
NEXMIF	Neurite Extension And Migration Factor	Protein Coding	Q5QGS0	36	GC0XM074778	6.5906496	https://www.genecards.org/cgi-bin/carddisp.pl?gene=NEXMIF
G6PC3	Glucose-6-Phosphatase Catalytic Subunit 3	Protein Coding	Q9BUM1	44	GC17P044070	6.58456945	https://www.genecards.org/cgi-bin/carddisp.pl?gene=G6PC3
SCRIB	Scribble Planar Cell Polarity Protein	Protein Coding	C0HLS1	43	GC08M146307	6.5778904	https://www.genecards.org/cgi-bin/carddisp.pl?gene=SCRIB
PVALB	Parvalbumin	Protein Coding	P20472	44	GC22M036800	6.57577229	https://www.genecards.org/cgi-bin/carddisp.pl?gene=PVALB
NRXN2	Neurexin 2	Protein Coding	Q9P2S2	42	GC11M116819	6.55404186	https://www.genecards.org/cgi-bin/carddisp.pl?gene=NRXN2
FGB	Fibrinogen Beta Chain	Protein Coding	P02675	50	GC04P154673	6.5529871	https://www.genecards.org/cgi-bin/carddisp.pl?gene=FGB

WASF1	WASP Family Member 1	Protein Coding	Q92558	48	GC06M110099	6.55174828	https://www.genecards.org/cgi-bin/carddisp.pl?gene=WASF1
ALPP	Alkaline Phosphatase, Placental	Protein Coding	P05187	47	GC02P232378	6.54115343	https://www.genecards.org/cgi-bin/carddisp.pl?gene=ALPP
NLGN2	Neuroligin 2	Protein Coding	Q8NFZ4	44	GC17P092360	6.52793694	https://www.genecards.org/cgi-bin/carddisp.pl?gene=NLGN2
TUBB3	Tubulin Beta 3 Class III	Protein Coding	Q13509	52	GC16P092506	6.52670193	https://www.genecards.org/cgi-bin/carddisp.pl?gene=TUBB3
NEBL	Nebulette	Protein Coding	O76041	43	GC10M020779	6.52493095	https://www.genecards.org/cgi-bin/carddisp.pl?gene=NEBL
PON1	Paraoxonase 1	Protein Coding	P27169	51	GC07M095297	6.52300024	https://www.genecards.org/cgi-bin/carddisp.pl?gene=PON1
TNFRSF11A	TNF Receptor Superfamily Member 11a	Protein Coding	Q9Y6Q6	51	GC18P062325	6.51304483	https://www.genecards.org/cgi-bin/carddisp.pl?gene=TNFRSF11A
PLXNC1	Plexin C1	Protein Coding	O60486	44	GC12P094150	6.51146841	https://www.genecards.org/cgi-bin/carddisp.pl?gene=PLXNC1
IFI27	Interferon Alpha Inducible Protein 27	Protein Coding	P40305	41	GC14P094104	6.50568295	https://www.genecards.org/cgi-bin/carddisp.pl?gene=IFI27
HNRNPU	Heterogeneous Nuclear Ribonucleoprotein U	Protein Coding	Q00839	47	GC01M244844	6.50513983	https://www.genecards.org/cgi-bin/carddisp.pl?gene=HNRNPU
ARHGAP31	Rho GTPase Activating Protein 31	Protein Coding	Q2M1Z3	45	GC03P119294	6.5032382	https://www.genecards.org/cgi-bin/carddisp.pl?gene=ARHGAP31
CASP8	Caspase 8	Protein Coding	Q14790	55	GC02P201233	6.50313091	https://www.genecards.org/cgi-bin/carddisp.pl?gene=CASP8
KCTD7	Potassium Channel Tetramerization Domain Containing 7	Protein Coding	Q96MP8	42	GC07P066628	6.48138952	https://www.genecards.org/cgi-bin/carddisp.pl?gene=KCTD7
YWHAG	Tyrosine 3-Monooxygenase/Tryptophan 5-Monooxygenase Activation Protein Gamma	Protein Coding	P61981	52	GC07M078254	6.4767971	https://www.genecards.org/cgi-bin/carddisp.pl?gene=YWHAG
PPIA	Peptidylprolyl Isomerase A	Protein Coding	P62937	49	GC07P044811	6.4725666	https://www.genecards.org/cgi-bin/carddisp.pl?gene=PPIA
FAS	Fas Cell Surface Death Receptor	Protein Coding	P25445	53	GC10P104467	6.47078419	https://www.genecards.org/cgi-bin/carddisp.pl?gene=FAS
LPAR3	Lysophosphatidic Acid Receptor 3	Protein Coding	Q9UBY5	46	GC01M084811	6.47073269	https://www.genecards.org/cgi-bin/carddisp.pl?gene=LPAR3
S1PR1	Sphingosine-1-Phosphate Receptor 1	Protein Coding	P21453	49	GC01P101236	6.46573973	https://www.genecards.org/cgi-bin/carddisp.pl?gene=S1PR1
PTPRT	Protein Tyrosine Phosphatase Receptor Type T	Protein Coding	O14522	47	GC20M042124	6.46476841	https://www.genecards.org/cgi-bin/carddisp.pl?gene=PTPRT
OLR1	Oxidized Low Density Lipoprotein Receptor 1	Protein Coding	P78380	46	GC12M026826	6.46452475	https://www.genecards.org/cgi-bin/carddisp.pl?gene=OLR1
MT-ND5	Mitochondrially Encoded NADH:Ubiquinone Oxidoreductase Core Subunit 5	Protein Coding	P03915	36	GCMTP012339	6.46074867	https://www.genecards.org/cgi-bin/carddisp.pl?gene=MT-ND5
ARAP2	ArfGAP With RhoGAP Domain, Ankyrin Repeat And PH Domain 2	Protein Coding	Q8WZ64	39	GC04M035950	6.44819069	https://www.genecards.org/cgi-bin/carddisp.pl?gene=ARAP2
SLC9A5	Solute Carrier Family 9 Member A5	Protein Coding	Q14940	41	GC16P067371	6.43441486	https://www.genecards.org/cgi-bin/carddisp.pl?gene=SLC9A5
PTPRS	Protein Tyrosine Phosphatase Receptor Type S	Protein Coding	Q13332	50	GC19M016733	6.42879248	https://www.genecards.org/cgi-bin/carddisp.pl?gene=PTPRS
CSNK2A1	Casein Kinase 2 Alpha 1	Protein Coding	P68400	54	GC20M000472	6.42228699	https://www.genecards.org/cgi-bin/carddisp.pl?gene=CSNK2A1

CLDN3	Claudin 3	Protein Coding	O15551	44	GC07M073768	6.41720295	https://www.genecards.org/cgi-bin/carddisp.pl?gene=CLDN3
NLGN4X	Neuroligin 4 X-Linked	Protein Coding	Q8N0W4	47	GC0XM005840	6.40185833	https://www.genecards.org/cgi-bin/carddisp.pl?gene=NLGN4X
HMOX1	Heme Oxygenase 1	Protein Coding	P09601	55	GC22P035380	6.39531231	https://www.genecards.org/cgi-bin/carddisp.pl?gene=HMOX1
FES	FES Proto-Oncogene, Tyrosine Kinase	Protein Coding	P07332	51	GC15P090883	6.3926096	https://www.genecards.org/cgi-bin/carddisp.pl?gene=FES
CLDN7	Claudin 7	Protein Coding	O95471	44	GC17M007259	6.38861895	https://www.genecards.org/cgi-bin/carddisp.pl?gene=CLDN7
DNMT1	DNA Methyltransferase 1	Protein Coding	P26358	56	GC19M010133	6.3783083	https://www.genecards.org/cgi-bin/carddisp.pl?gene=DNMT1
PDCD6IP	Programmed Cell Death 6 Interacting Protein	Protein Coding	Q8WUM4	47	GC03P033798	6.37792063	https://www.genecards.org/cgi-bin/carddisp.pl?gene=PDCD6IP
PKP3	Plakophilin 3	Protein Coding	Q9Y446	42	GC11P005654	6.36995649	https://www.genecards.org/cgi-bin/carddisp.pl?gene=PKP3
DKK1	Dickkopf WNT Signaling Pathway Inhibitor 1	Protein Coding	O94907	48	GC10P052314	6.36802101	https://www.genecards.org/cgi-bin/carddisp.pl?gene=DKK1
KRT6B	Keratin 6B	Protein Coding	P04259	45	GC12M052446	6.36769629	https://www.genecards.org/cgi-bin/carddisp.pl?gene=KRT6B
PRKN	Parkin RBR E3 Ubiquitin Protein Ligase	Protein Coding	O60260	52	GC06M161348	6.35251188	https://www.genecards.org/cgi-bin/carddisp.pl?gene=PRKN
SAMD12	Sterile Alpha Motif Domain Containing 12	Protein Coding	Q8N8I0	40	GC08M118131	6.35043049	https://www.genecards.org/cgi-bin/carddisp.pl?gene=SAMD12
SH3TC2	SH3 Domain And Tetratricopeptide Repeats 2	Protein Coding	Q8TF17	41	GC05M148923	6.34945059	https://www.genecards.org/cgi-bin/carddisp.pl?gene=SH3TC2
SYP	Synaptophysin	Protein Coding	P08247	50	GC0XM049187	6.34555531	https://www.genecards.org/cgi-bin/carddisp.pl?gene=SYP
S100B	S100 Calcium Binding Protein B	Protein Coding	P04271	48	GC21M052965	6.33727837	https://www.genecards.org/cgi-bin/carddisp.pl?gene=S100B
YWHAZ	Tyrosine 3-Monooxygenase/Tryptophan 5-Monooxygenase Activation Protein Zeta	Protein Coding	P63104	51	GC08M100917	6.33675766	https://www.genecards.org/cgi-bin/carddisp.pl?gene=YWHAZ
GAB1	GRB2 Associated Binding Protein 1	Protein Coding	Q13480	48	GC04P143336	6.32714176	https://www.genecards.org/cgi-bin/carddisp.pl?gene=GAB1
MPO	Myeloperoxidase	Protein Coding	P05164	56	GC17M058269	6.32506943	https://www.genecards.org/cgi-bin/carddisp.pl?gene=MPO
ADIPOQ	Adiponectin, C1Q And Collagen Domain Containing	Protein Coding	Q15848	50	GC03P186842	6.31659794	https://www.genecards.org/cgi-bin/carddisp.pl?gene=ADIPOQ
CAMK2G	Calcium/Calmodulin Dependent Protein Kinase II Gamma	Protein Coding	Q13555	51	GC10M073812	6.31637192	https://www.genecards.org/cgi-bin/carddisp.pl?gene=CAMK2G
ANGPTL4	Angiopoietin Like 4	Protein Coding	Q9BY76	47	GC19P008363	6.31525517	https://www.genecards.org/cgi-bin/carddisp.pl?gene=ANGPTL4
NT5E	5'-Nucleotidase Ecto	Protein Coding	P21589	55	GC06P085449	6.31474257	https://www.genecards.org/cgi-bin/carddisp.pl?gene=NT5E
VSX1	Visual System Homeobox 1	Protein Coding	Q9NZR4	42	GC20M025070	6.31107664	https://www.genecards.org/cgi-bin/carddisp.pl?gene=VSX1
HSPA1A	Heat Shock Protein Family A (Hsp70) Member 1A	Protein Coding	P0DMV8	50	GC06P118924	6.30858231	https://www.genecards.org/cgi-bin/carddisp.pl?gene=HSPA1A
CHGA	Chromogranin A	Protein Coding	P10645	47	GC14P092972	6.30622339	https://www.genecards.org/cgi-bin/carddisp.pl?gene=CHGA

BIRC3	Baculoviral IAP Repeat Containing 3	Protein Coding	Q13489	50	GC11P102317	6.3027935	https://www.genecards.org/cgi-bin/carddisp.pl?gene=BIRC3
SHH	Sonic Hedgehog Signaling Molecule	Protein Coding	Q15465	54	GC07M155799	6.30264044	https://www.genecards.org/cgi-bin/carddisp.pl?gene=SHH
GAST	Gastrin	Protein Coding	P01350	43	GC17P041712	6.28717422	https://www.genecards.org/cgi-bin/carddisp.pl?gene=GAST
PGR-AS1	PGR Antisense RNA 1	RNA Gene		11	GC11P101131	6.28641129	https://www.genecards.org/cgi-bin/carddisp.pl?gene=PGR-AS1
PDLIM5	PDZ And LIM Domain 5	Protein Coding	Q96HC4	44	GC04P094451	6.2793169	https://www.genecards.org/cgi-bin/carddisp.pl?gene=PDLIM5
LRP6	LDL Receptor Related Protein 6	Protein Coding	O75581	51	GC12M026857	6.27439833	https://www.genecards.org/cgi-bin/carddisp.pl?gene=LRP6
CORO1C	Coronin 1C	Protein Coding	Q9ULV4	43	GC12M108645	6.27344608	https://www.genecards.org/cgi-bin/carddisp.pl?gene=CORO1C
ACTR2	Actin Related Protein 2	Protein Coding	P61160	47	GC02P065227	6.26797676	https://www.genecards.org/cgi-bin/carddisp.pl?gene=ACTR2
YWHAE	Tyrosine 3-Monooxygenase/Tryptophan 5-Monooxygenase Activation Protein Epsilon	Protein Coding	P62258	53	GC17M014951	6.26455116	https://www.genecards.org/cgi-bin/carddisp.pl?gene=YWHAE
PDLIM1	PDZ And LIM Domain 1	Protein Coding	O00151	44	GC10M095237	6.26445007	https://www.genecards.org/cgi-bin/carddisp.pl?gene=PDLIM1
MAPT	Microtubule Associated Protein Tau	Protein Coding	P10636	53	GC17P045894	6.25999928	https://www.genecards.org/cgi-bin/carddisp.pl?gene=MAPT
IL13	Interleukin 13	Protein Coding	P35225	47	GC05P132656	6.25686502	https://www.genecards.org/cgi-bin/carddisp.pl?gene=IL13
GNAQ	G Protein Subunit Alpha Q	Protein Coding	P50148	52	GC09M077716	6.2496047	https://www.genecards.org/cgi-bin/carddisp.pl?gene=GNAQ
TUBB4A	Tubulin Beta 4A Class IVa	Protein Coding	P04350	51	GC19M016791	6.24310255	https://www.genecards.org/cgi-bin/carddisp.pl?gene=TUBB4A
ARPC5L	Actin Related Protein 2/3 Complex Subunit 5 Like	Protein Coding	Q9BPX5	40	GC09P124862	6.233284	https://www.genecards.org/cgi-bin/carddisp.pl?gene=ARPC5L
BAD	BCL2 Associated Agonist Of Cell Death	Protein Coding	Q92934	47	GC11M116809	6.2327795	https://www.genecards.org/cgi-bin/carddisp.pl?gene=BAD
HID1	HID1 Domain Containing	Protein Coding	Q8IV36	39	GC17M074950	6.23222303	https://www.genecards.org/cgi-bin/carddisp.pl?gene=HID1
SPN	Sialophorin	Protein Coding	P16150	44	GC16P029662	6.22829151	https://www.genecards.org/cgi-bin/carddisp.pl?gene=SPN
SNCA	Synuclein Alpha	Protein Coding	P37840	54	GC04M089724	6.22572327	https://www.genecards.org/cgi-bin/carddisp.pl?gene=SNCA
IQSEC1	IQ Motif And Sec7 Domain ArfGEF 1	Protein Coding	Q6DN90	44	GC03M024956	6.22524548	https://www.genecards.org/cgi-bin/carddisp.pl?gene=IQSEC1
GDNF	Glial Cell Derived Neurotrophic Factor	Protein Coding	P39905	53	GC05M037812	6.21628571	https://www.genecards.org/cgi-bin/carddisp.pl?gene=GDNF
FLOT1	Flotillin 1	Protein Coding	O75955	45	GC06M087133	6.21262169	https://www.genecards.org/cgi-bin/carddisp.pl?gene=FLOT1
CXCR3	C-X-C Motif Chemokine Receptor 3	Protein Coding	P49682	47	GC0XM071615	6.21124792	https://www.genecards.org/cgi-bin/carddisp.pl?gene=CXCR3
ELK1	ETS Transcription Factor ELK1	Protein Coding	P19419	48	GC0XM047635	6.21122837	https://www.genecards.org/cgi-bin/carddisp.pl?gene=ELK1
CD99L2	CD99 Molecule Like 2	Protein Coding	Q8TCZ2	38	GC0XM150766	6.21060753	https://www.genecards.org/cgi-bin/carddisp.pl?gene=CD99L2

KCNA2	Potassium Voltage-Gated Channel Subfamily A Member 2	Protein Coding	P16389	50	GC01M110519	6.18779802	https://www.genecards.org/cgi-bin/carddisp.pl?gene=KCNA2
SRF	Serum Response Factor	Protein Coding	P11831	47	GC06P043171	6.18537617	https://www.genecards.org/cgi-bin/carddisp.pl?gene=SRF
PTPN21	Protein Tyrosine Phosphatase Non-Receptor Type 21	Protein Coding	Q16825	42	GC14M088465	6.18332577	https://www.genecards.org/cgi-bin/carddisp.pl?gene=PTPN21
PLXNB3	Plexin B3	Protein Coding	Q9ULL4	41	GC0XP153764	6.18285942	https://www.genecards.org/cgi-bin/carddisp.pl?gene=PLXNB3
PTPN14	Protein Tyrosine Phosphatase Non-Receptor Type 14	Protein Coding	Q15678	47	GC01M214348	6.17310429	https://www.genecards.org/cgi-bin/carddisp.pl?gene=PTPN14
COL6A3	Collagen Type VI Alpha 3 Chain	Protein Coding	P12111	49	GC02M237324	6.17288446	https://www.genecards.org/cgi-bin/carddisp.pl?gene=COL6A3
CDH9	Cadherin 9	Protein Coding	Q9ULB4	43	GC05M026916	6.17245388	https://www.genecards.org/cgi-bin/carddisp.pl?gene=CDH9
NOD2	Nucleotide Binding Oligomerization Domain Containing 2	Protein Coding	Q9HC29	51	GC16P050693	6.1685648	https://www.genecards.org/cgi-bin/carddisp.pl?gene=NOD2
STRADA	STE20 Related Adaptor Alpha	Protein Coding	Q7RTN6	48	GC17M068950	6.16183805	https://www.genecards.org/cgi-bin/carddisp.pl?gene=STRADA
SQSTM1	Sequestosome 1	Protein Coding	Q13501	53	GC05P179806	6.15329361	https://www.genecards.org/cgi-bin/carddisp.pl?gene=SQSTM1
EGR2	Early Growth Response 2	Protein Coding	P11161	48	GC10M062811	6.15253401	https://www.genecards.org/cgi-bin/carddisp.pl?gene=EGR2
CCR7	C-C Motif Chemokine Receptor 7	Protein Coding	P32248	48	GC17M067978	6.14732552	https://www.genecards.org/cgi-bin/carddisp.pl?gene=CCR7
INPPL1	Inositol Polyphosphate Phosphatase Like 1	Protein Coding	O15357	52	GC11P072223	6.1414361	https://www.genecards.org/cgi-bin/carddisp.pl?gene=INPPL1
BLZF1	Basic Leucine Zipper Nuclear Factor 1	Protein Coding	Q9H2G9	41	GC01P169367	6.1361475	https://www.genecards.org/cgi-bin/carddisp.pl?gene=BLZF1
PRKCG	Protein Kinase C Gamma	Protein Coding	P05129	54	GC19P053879	6.13410616	https://www.genecards.org/cgi-bin/carddisp.pl?gene=PRKCG
GNE	Glucosamine (UDP-N-Acetyl)-2-Epimerase/N-Acetylmannosamine Kinase	Protein Coding	Q9Y223	46	GC09M036214	6.13253927	https://www.genecards.org/cgi-bin/carddisp.pl?gene=GNE
SLC12A2	Solute Carrier Family 12 Member 2	Protein Coding	P55011	53	GC05P128083	6.12981987	https://www.genecards.org/cgi-bin/carddisp.pl?gene=SLC12A2
NOS1	Nitric Oxide Synthase 1	Protein Coding	P29475	53	GC12M117208	6.12304974	https://www.genecards.org/cgi-bin/carddisp.pl?gene=NOS1
CD82	CD82 Molecule	Protein Coding	P27701	47	GC11P044586	6.11776543	https://www.genecards.org/cgi-bin/carddisp.pl?gene=CD82
AGER	Advanced Glycosylation End-Product Specific Receptor	Protein Coding	Q15109	50	GC06M032180	6.10722494	https://www.genecards.org/cgi-bin/carddisp.pl?gene=AGER
PWAR1	Prader Willi/Angelman Region RNA 1	RNA Gene		16	GC15P025135	6.10632563	https://www.genecards.org/cgi-bin/carddisp.pl?gene=PWAR1
HLA-DQB1	Major Histocompatibility Complex, Class II, DQ Beta 1	Protein Coding	P01920	46	GC06M087318	6.10401678	https://www.genecards.org/cgi-bin/carddisp.pl?gene=HLA-DQB1
TRAF3	TNF Receptor Associated Factor 3	Protein Coding	Q13114	51	GC14P113792	6.09662056	https://www.genecards.org/cgi-bin/carddisp.pl?gene=TRAF3
DES	Desmin	Protein Coding	P17661	51	GC02P219418	6.09528351	https://www.genecards.org/cgi-bin/carddisp.pl?gene=DES
ABO	ABO, Alpha 1-3-N-Acetylgalactosaminyltransferase And Alpha 1-3-Galactosyltransferase	Protein Coding	P16442	41	GC09M133250	6.08791828	https://www.genecards.org/cgi-bin/carddisp.pl?gene=ABO

MYL9	Myosin Light Chain 9	Protein Coding	P24844	47	GC20P0 36541	6.08763123	https://www.genecards.org/cgi-bin/carddisp.pl?gene=MYL9
RHOJ	Ras Homolog Family Member J	Protein Coding	Q9H4E5	41	GC14P0 63204	6.08163214	https://www.genecards.org/cgi-bin/carddisp.pl?gene=RHOJ
ALOX5	Arachidonate 5-Lipoxygenase	Protein Coding	P09917	51	GC10P0 45374	6.07854176	https://www.genecards.org/cgi-bin/carddisp.pl?gene=ALOX5
SLC1A2	Solute Carrier Family 1 Member 2	Protein Coding	P43004	53	GC11M0 35267	6.07394886	https://www.genecards.org/cgi-bin/carddisp.pl?gene=SLC1A2
RHOC	Ras Homolog Family Member C	Protein Coding	P08134	45	GC01M1 12701	6.07378101	https://www.genecards.org/cgi-bin/carddisp.pl?gene=RHOC
NRAS	NRAS Proto-Oncogene, GTPase	Protein Coding	P01111	54	GC01M1 14704	6.07075405	https://www.genecards.org/cgi-bin/carddisp.pl?gene=NRAS
SP1	Sp1 Transcription Factor	Protein Coding	P08047	49	GC12P0 53380	6.06956577	https://www.genecards.org/cgi-bin/carddisp.pl?gene=SP1
OVOL2	Ovo Like Zinc Finger 2	Protein Coding	Q9BRP0	40	GC20M0 17956	6.06148338	https://www.genecards.org/cgi-bin/carddisp.pl?gene=OVOL2
HNRNPK	Heterogeneous Nuclear Ribonucleoprotein K	Protein Coding	P61978	48	GC09M1 05580	6.05791569	https://www.genecards.org/cgi-bin/carddisp.pl?gene=HNRNPK
DCHS1	Dachsous Cadherin-Related 1	Protein Coding	Q96JQ0	41	GC11M0 06621	6.05659914	https://www.genecards.org/cgi-bin/carddisp.pl?gene=DCHS1
FGA	Fibrinogen Alpha Chain	Protein Coding	P02671	53	GC04M1 54583	6.05474472	https://www.genecards.org/cgi-bin/carddisp.pl?gene=FGA
POMC	Proopiomelanocortin	Protein Coding	P01189	51	GC02M0 25160	6.05328655	https://www.genecards.org/cgi-bin/carddisp.pl?gene=POMC
MAP2K4	Mitogen-Activated Protein Kinase Kinase 4	Protein Coding	P45985	51	GC17P0 12020	6.05096102	https://www.genecards.org/cgi-bin/carddisp.pl?gene=MAP2K4
ANXA6	Annexin A6	Protein Coding	P08133	46	GC05M1 51100	6.05064678	https://www.genecards.org/cgi-bin/carddisp.pl?gene=ANXA6
PF4	Platelet Factor 4	Protein Coding	P02776	44	GC04M0 73980	6.04947567	https://www.genecards.org/cgi-bin/carddisp.pl?gene=PF4
BMP2	Bone Morphogenetic Protein 2	Protein Coding	P12643	50	GC20P0 06767	6.04526472	https://www.genecards.org/cgi-bin/carddisp.pl?gene=BMP2
ELANE	Elastase, Neutrophil Expressed	Protein Coding	P08246	54	GC19P0 94739	6.04270744	https://www.genecards.org/cgi-bin/carddisp.pl?gene=ELANE
COL4A6	Collagen Type IV Alpha 6 Chain	Protein Coding	Q14031	47	GC0XM 108155	6.03447056	https://www.genecards.org/cgi-bin/carddisp.pl?gene=COL4A6
VEGFD	Vascular Endothelial Growth Factor D	Protein Coding	O43915	44	GC0XM 015345	6.03327465	https://www.genecards.org/cgi-bin/carddisp.pl?gene=VEGFD
RALA	RAS Like Proto-Oncogene A	Protein Coding	P11233	50	GC07P0 39622	6.03221321	https://www.genecards.org/cgi-bin/carddisp.pl?gene=RALA
ALG13	ALG13 UDP-N-Acetylglucosaminyltransferase Subunit	Protein Coding	Q9NP73	43	GC0XP1 11665	6.03005314	https://www.genecards.org/cgi-bin/carddisp.pl?gene=ALG13
PTPRB	Protein Tyrosine Phosphatase Receptor Type B	Protein Coding	P23467	48	GC12M0 70516	6.0137887	https://www.genecards.org/cgi-bin/carddisp.pl?gene=PTPRB
ADD1	Adducin 1	Protein Coding	P35611	47	GC04P0 02866	6.01061535	https://www.genecards.org/cgi-bin/carddisp.pl?gene=ADD1
PRKG1	Protein Kinase CGMP-Dependent 1	Protein Coding	Q13976	54	GC10P0 50991	6.00879574	https://www.genecards.org/cgi-bin/carddisp.pl?gene=PRKG1
C3	Complement C3	Protein Coding	P01024	53	GC19M0 06677	5.99641752	https://www.genecards.org/cgi-bin/carddisp.pl?gene=C3

MEG8	Maternally Expressed 8, Small Nucleolar RNA Host Gene	RNA Gene		21	GC14P114434	5.99502563	https://www.genecards.org/cgi-bin/carddisp.pl?gene=MEG8
IL1RN	Interleukin 1 Receptor Antagonist	Protein Coding	P18510	53	GC02P128885	5.98850155	https://www.genecards.org/cgi-bin/carddisp.pl?gene=IL1RN
CLN5	CLN5 Intracellular Trafficking Protein	Protein Coding	O75503	44	GC13P076990	5.98713636	https://www.genecards.org/cgi-bin/carddisp.pl?gene=CLN5
B2M	Beta-2-Microglobulin	Protein Coding	P61769	53	GC15P044711	5.98251581	https://www.genecards.org/cgi-bin/carddisp.pl?gene=B2M
ACTN3	Actinin Alpha 3	Protein Coding	Q08043	43	GC11P066546	5.96520138	https://www.genecards.org/cgi-bin/carddisp.pl?gene=ACTN3
GAD1	Glutamate Decarboxylase 1	Protein Coding	Q99259	53	GC02P170813	5.9642477	https://www.genecards.org/cgi-bin/carddisp.pl?gene=GAD1
PCDH10	Protocadherin 10	Protein Coding	Q9P2E7	44	GC04P133149	5.95911741	https://www.genecards.org/cgi-bin/carddisp.pl?gene=PCDH10
MDH2	Malate Dehydrogenase 2	Protein Coding	P40926	51	GC07P076048	5.95504522	https://www.genecards.org/cgi-bin/carddisp.pl?gene=MDH2
CSF3R	Colony Stimulating Factor 3 Receptor	Protein Coding	Q99062	51	GC01M036466	5.95078135	https://www.genecards.org/cgi-bin/carddisp.pl?gene=CSF3R
OLFM4	Olfactomedin 4	Protein Coding	Q6UX06	44	GC13P053028	5.94954586	https://www.genecards.org/cgi-bin/carddisp.pl?gene=OLFM4
MYH10	Myosin Heavy Chain 10	Protein Coding	P35580	49	GC17M008474	5.94231129	https://www.genecards.org/cgi-bin/carddisp.pl?gene=MYH10
GPSM2	G Protein Signaling Modulator 2	Protein Coding	P81274	45	GC01P108875	5.93695068	https://www.genecards.org/cgi-bin/carddisp.pl?gene=GPSM2
PRL	Prolactin	Protein Coding	P01236	47	GC06M022287	5.93557739	https://www.genecards.org/cgi-bin/carddisp.pl?gene=PRL
MT-CO1	Mitochondrially Encoded Cytochrome C Oxidase I	Protein Coding	P00395	35	GCMTP005906	5.93069792	https://www.genecards.org/cgi-bin/carddisp.pl?gene=MT-CO1
CSTB	Cystatin B	Protein Coding	P04080	50	GC21M043772	5.92848635	https://www.genecards.org/cgi-bin/carddisp.pl?gene=CSTB
MIR204	MicroRNA 204	RNA Gene		23	GC09M070809	5.91989851	https://www.genecards.org/cgi-bin/carddisp.pl?gene=MIR204
DUSP22	Dual Specificity Phosphatase 22	Protein Coding	Q9NRW4	40	GC06P000529	5.91561985	https://www.genecards.org/cgi-bin/carddisp.pl?gene=DUSP22
TESK1	Testis Associated Actin Remodelling Kinase 1	Protein Coding	Q15569	44	GC09P035605	5.91489697	https://www.genecards.org/cgi-bin/carddisp.pl?gene=TESK1
BCL2L1	BCL2 Like 1	Protein Coding	Q07817	52	GC20M031664	5.90850353	https://www.genecards.org/cgi-bin/carddisp.pl?gene=BCL2L1
SST	Somatostatin	Protein Coding	P61278	44	GC03M187668	5.90653419	https://www.genecards.org/cgi-bin/carddisp.pl?gene=SST
MYL2	Myosin Light Chain 2	Protein Coding	P10916	52	GC12M110910	5.89662075	https://www.genecards.org/cgi-bin/carddisp.pl?gene=MYL2
TGFA	Transforming Growth Factor Alpha	Protein Coding	P01135	50	GC02M070447	5.89624643	https://www.genecards.org/cgi-bin/carddisp.pl?gene=TGFA
PNKP	Polynucleotide Kinase 3'-Phosphatase	Protein Coding	Q96T60	51	GC19M085868	5.88629675	https://www.genecards.org/cgi-bin/carddisp.pl?gene=PNKP
RPL7A	Ribosomal Protein L7a	Protein Coding	P62424	45	GC09P133348	5.8843317	https://www.genecards.org/cgi-bin/carddisp.pl?gene=RPL7A
SKAP1	Src Kinase Associated Phosphoprotein 1	Protein Coding	Q86WV1	44	GC17M048133	5.87735319	https://www.genecards.org/cgi-bin/carddisp.pl?gene=SKAP1

CD14	CD14 Molecule	Protein Coding	P08571	50	GC05M140631	5.87388039	https://www.genecards.org/cgi-bin/carddisp.pl?gene=CD14
EFHC1	EF-Hand Domain Containing 1	Protein Coding	Q5JVL4	45	GC06P052362	5.87172365	https://www.genecards.org/cgi-bin/carddisp.pl?gene=EFHC1
RBFOX3	RNA Binding Fox-1 Homolog 3	Protein Coding	A6NFN3	41	GC17M079089	5.87152672	https://www.genecards.org/cgi-bin/carddisp.pl?gene=RBFOX3
SPTLC1	Serine Palmitoyltransferase Long Chain Base Subunit 1	Protein Coding	O15269	50	GC09M105787	5.86349726	https://www.genecards.org/cgi-bin/carddisp.pl?gene=SPTLC1
ATP1B1	ATPase Na+/K+ Transporting Subunit Beta 1	Protein Coding	P05026	52	GC01P169105	5.85589218	https://www.genecards.org/cgi-bin/carddisp.pl?gene=ATP1B1
SAFB	Scaffold Attachment Factor B	Protein Coding	Q15424	43	GC19P005623	5.85034084	https://www.genecards.org/cgi-bin/carddisp.pl?gene=SAFB
NTN1	Netrin 1	Protein Coding	O95631	50	GC17P092446	5.83989143	https://www.genecards.org/cgi-bin/carddisp.pl?gene=NTN1
TIMP2	TIMP Metallopeptidase Inhibitor 2	Protein Coding	P16035	45	GC17M078852	5.83410358	https://www.genecards.org/cgi-bin/carddisp.pl?gene=TIMP2
TNFRSF1A	TNF Receptor Superfamily Member 1A	Protein Coding	P19438	53	GC12M006328	5.83406639	https://www.genecards.org/cgi-bin/carddisp.pl?gene=TNFRSF1A
IGF2R	Insulin Like Growth Factor 2 Receptor	Protein Coding	P11717	50	GC06P159969	5.82844782	https://www.genecards.org/cgi-bin/carddisp.pl?gene=IGF2R
PIAS1	Protein Inhibitor Of Activated STAT 1	Protein Coding	O75925	49	GC15P068054	5.82787895	https://www.genecards.org/cgi-bin/carddisp.pl?gene=PIAS1
LSAMP	Limbic System Associated Membrane Protein	Protein Coding	Q13449	41	GC03M115802	5.82304239	https://www.genecards.org/cgi-bin/carddisp.pl?gene=LSAMP
KANSL1	KAT8 Regulatory NSL Complex Subunit 1	Protein Coding	Q7Z3B3	44	GC17M068371	5.81792784	https://www.genecards.org/cgi-bin/carddisp.pl?gene=KANSL1
P4HB	Prolyl 4-Hydroxylase Subunit Beta	Protein Coding	P07237	53	GC17M081843	5.81252384	https://www.genecards.org/cgi-bin/carddisp.pl?gene=P4HB
CD46	CD46 Molecule	Protein Coding	P15529	51	GC01P207752	5.81169462	https://www.genecards.org/cgi-bin/carddisp.pl?gene=CD46
SIGLEC1	Sialic Acid Binding Ig Like Lectin 1	Protein Coding	Q9BZZ2	44	GC20M003686	5.8056736	https://www.genecards.org/cgi-bin/carddisp.pl?gene=SIGLEC1
CREB1	CAMP Responsive Element Binding Protein 1	Protein Coding	P16220	53	GC02P207529	5.80258179	https://www.genecards.org/cgi-bin/carddisp.pl?gene=CREB1
MT-ND4	Mitochondrially Encoded NADH:Ubiquinone Oxidoreductase Core Subunit 4	Protein Coding	P03905	35	GCMTP010762	5.79917955	https://www.genecards.org/cgi-bin/carddisp.pl?gene=MT-ND4
SLC3A2	Solute Carrier Family 3 Member 2	Protein Coding	P08195	47	GC11P062856	5.79301071	https://www.genecards.org/cgi-bin/carddisp.pl?gene=SLC3A2
PTPA	Protein Phosphatase 2 Phosphatase Activator	Protein Coding	Q15257	46	GC09P135865	5.77040482	https://www.genecards.org/cgi-bin/carddisp.pl?gene=PTPA
CAPN5	Calpain 5	Protein Coding	O15484	46	GC11P077066	5.76998091	https://www.genecards.org/cgi-bin/carddisp.pl?gene=CAPN5
SPTBN1	Spectrin Beta, Non-Erythrocytic 1	Protein Coding	Q01082	50	GC02P054456	5.7681675	https://www.genecards.org/cgi-bin/carddisp.pl?gene=SPTBN1
AFG2B	AFG2 AAA ATPase Homolog B	Protein Coding	Q9BVQ7	39	GC15P045402	5.76544189	https://www.genecards.org/cgi-bin/carddisp.pl?gene=AFG2B
EPHA1	EPH Receptor A1	Protein Coding	P21709	51	GC07M143390	5.76404095	https://www.genecards.org/cgi-bin/carddisp.pl?gene=EPHA1
CHAD	Chondroadherin	Protein Coding	O15335	41	GC17M050464	5.75727558	https://www.genecards.org/cgi-bin/carddisp.pl?gene=CHAD

CHUK	Component Of Inhibitor Of Nuclear Factor Kappa B Kinase Complex	Protein Coding	O15111	53	GC10M100328	5.7431469	https://www.genecards.org/cgi-bin/carddisp.pl?gene=CHUK
SERPINC1	Serpin Family C Member 1	Protein Coding	P01008	53	GC01M174856	5.74031734	https://www.genecards.org/cgi-bin/carddisp.pl?gene=SERPINC1
TAC1	Tachykinin Precursor 1	Protein Coding	P20366	44	GC07P097734	5.73909187	https://www.genecards.org/cgi-bin/carddisp.pl?gene=TAC1
MARCHF6	Membrane Associated Ring-CH-Type Finger 6	Protein Coding	O60337	39	GC05P010663	5.73807001	https://www.genecards.org/cgi-bin/carddisp.pl?gene=MARCHF6
MIR34A	MicroRNA 34a	RNA Gene		23	GC01M011962	5.72647619	https://www.genecards.org/cgi-bin/carddisp.pl?gene=MIR34A
MIR155	MicroRNA 155	RNA Gene		22	GC21P025573	5.71884251	https://www.genecards.org/cgi-bin/carddisp.pl?gene=MIR155
BGLAP	Bone Gamma-Carboxyglutamate Protein	Protein Coding	P02818	44	GC01P156242	5.70446777	https://www.genecards.org/cgi-bin/carddisp.pl?gene=BGLAP
PMM2	Phosphomannomutase 2	Protein Coding	O15305	50	GC16P008788	5.69566536	https://www.genecards.org/cgi-bin/carddisp.pl?gene=PMM2
ARHGEF9	Cdc42 Guanine Nucleotide Exchange Factor 9	Protein Coding	O43307	45	GC0XM063634	5.69518375	https://www.genecards.org/cgi-bin/carddisp.pl?gene=ARHGEF9
PGF	Placental Growth Factor	Protein Coding	P49763	48	GC14M074941	5.69283676	https://www.genecards.org/cgi-bin/carddisp.pl?gene=PGF
CDK4	Cyclin Dependent Kinase 4	Protein Coding	P11802	57	GC12M058990	5.68672085	https://www.genecards.org/cgi-bin/carddisp.pl?gene=CDK4
TGFB2	Transforming Growth Factor Beta 2	Protein Coding	P61812	54	GC01P218345	5.68411922	https://www.genecards.org/cgi-bin/carddisp.pl?gene=TGFB2
PPP2R1A	Protein Phosphatase 2 Scaffold Subunit Aalpha	Protein Coding	P30153	52	GC19P096484	5.684062	https://www.genecards.org/cgi-bin/carddisp.pl?gene=PPP2R1A
DRD2	Dopamine Receptor D2	Protein Coding	P14416	52	GC11M113409	5.68357468	https://www.genecards.org/cgi-bin/carddisp.pl?gene=DRD2
CASP9	Caspase 9	Protein Coding	P55211	51	GC01M015491	5.67002678	https://www.genecards.org/cgi-bin/carddisp.pl?gene=CASP9
SNORD118	Small Nucleolar RNA, C/D Box 118	RNA Gene		21	GC17M015416	5.66773319	https://www.genecards.org/cgi-bin/carddisp.pl?gene=SNORD118
CDH6	Cadherin 6	Protein Coding	P55285	46	GC05P031229	5.66377449	https://www.genecards.org/cgi-bin/carddisp.pl?gene=CDH6
FBLN1	Fibulin 1	Protein Coding	P23142	48	GC22P045502	5.66133928	https://www.genecards.org/cgi-bin/carddisp.pl?gene=FBLN1
FGFR4	Fibroblast Growth Factor Receptor 4	Protein Coding	P22455	55	GC05P177086	5.65985775	https://www.genecards.org/cgi-bin/carddisp.pl?gene=FGFR4
ADA	Adenosine Deaminase	Protein Coding	P00813	54	GC20M044620	5.6541667	https://www.genecards.org/cgi-bin/carddisp.pl?gene=ADA
PTPRD	Protein Tyrosine Phosphatase Receptor Type D	Protein Coding	P23468	48	GC09M008307	5.65028334	https://www.genecards.org/cgi-bin/carddisp.pl?gene=PTPRD
FALEC	Focally Amplified Long Non-Coding RNA In Epithelial Cancer	RNA Gene		17	GC01P158537	5.64861345	https://www.genecards.org/cgi-bin/carddisp.pl?gene=FALEC
CCL4	C-C Motif Chemokine Ligand 4	Protein Coding	P13236	44	GC17P036103	5.64851046	https://www.genecards.org/cgi-bin/carddisp.pl?gene=CCL4
HPSE	Heparanase	Protein Coding	Q9Y251	48	GC04M083292	5.63170958	https://www.genecards.org/cgi-bin/carddisp.pl?gene=HPSE
CXCL1	C-X-C Motif Chemokine Ligand 1	Protein Coding	P09341	46	GC04P073869	5.62813854	https://www.genecards.org/cgi-bin/carddisp.pl?gene=CXCL1

NFKBIA	NFKB Inhibitor Alpha	Protein Coding	P25963	54	GC14M035401	5.62208509	https://www.genecards.org/cgi-bin/carddisp.pl?gene=NFKBIA
ASAH1	N-Acylsphingosine Amidohydrolase 1	Protein Coding	Q13510	53	GC08M018055	5.60697556	https://www.genecards.org/cgi-bin/carddisp.pl?gene=ASAH1
TRC-GCA24-1	TRNA-Cys (GCA) 24-1	RNA Gene		8	GC17M067898	5.60627127	https://www.genecards.org/cgi-bin/carddisp.pl?gene=TRC-GCA24-1
CD55	CD55 Molecule (Cromer Blood Group)	Protein Coding	P08174	52	GC01P207321	5.60257626	https://www.genecards.org/cgi-bin/carddisp.pl?gene=CD55
LIMK1	LIM Domain Kinase 1	Protein Coding	P53667	52	GC07P074082	5.58976316	https://www.genecards.org/cgi-bin/carddisp.pl?gene=LIMK1
ST8SIA2	ST8 Alpha-N-Acetyl-Neuraminide Alpha-2,8-Sialyltransferase 2	Protein Coding	Q92186	45	GC15P092393	5.58934832	https://www.genecards.org/cgi-bin/carddisp.pl?gene=ST8SIA2
FLII	FLII Actin Remodeling Protein	Protein Coding	Q13045	47	GC17M018244	5.58717346	https://www.genecards.org/cgi-bin/carddisp.pl?gene=FLII
AP2M1	Adaptor Related Protein Complex 2 Subunit Mu 1	Protein Coding	Q96CW1	50	GC03P184174	5.58269691	https://www.genecards.org/cgi-bin/carddisp.pl?gene=AP2M1
MAGI1	Membrane Associated Guanylate Kinase, WW And PDZ Domain Containing 1	Protein Coding	Q96QZ7	44	GC03M065330	5.57873821	https://www.genecards.org/cgi-bin/carddisp.pl?gene=MAGI1
LASP1	LIM And SH3 Protein 1	Protein Coding	Q14847	46	GC17P038869	5.57871246	https://www.genecards.org/cgi-bin/carddisp.pl?gene=LASP1
TOLLIP	Toll Interacting Protein	Protein Coding	Q9H0E2	48	GC11M001274	5.57262039	https://www.genecards.org/cgi-bin/carddisp.pl?gene=TOLLIP
PABPC1	Poly(A) Binding Protein Cytoplasmic 1	Protein Coding	P11940	46	GC08M100685	5.57225037	https://www.genecards.org/cgi-bin/carddisp.pl?gene=PABPC1
MT-CO2	Mitochondrially Encoded Cytochrome C Oxidase II	Protein Coding	P00403	37	GCMTP007587	5.56805229	https://www.genecards.org/cgi-bin/carddisp.pl?gene=MT-CO2
PLA2G6	Phospholipase A2 Group VI	Protein Coding	O60733	51	GC22M074272	5.56640863	https://www.genecards.org/cgi-bin/carddisp.pl?gene=PLA2G6
COL5A1	Collagen Type V Alpha 1 Chain	Protein Coding	P20908	50	GC09P134641	5.56609726	https://www.genecards.org/cgi-bin/carddisp.pl?gene=COL5A1
TJP2	Tight Junction Protein 2	Protein Coding	Q9UDY2	48	GC09P069121	5.56602907	https://www.genecards.org/cgi-bin/carddisp.pl?gene=TJP2
CLASP1	Cytoplasmic Linker Associated Protein 1	Protein Coding	Q7Z460	45	GC02M121337	5.56507063	https://www.genecards.org/cgi-bin/carddisp.pl?gene=CLASP1
NPM1	Nucleophosmin 1	Protein Coding	P06748	54	GC05P171387	5.56149864	https://www.genecards.org/cgi-bin/carddisp.pl?gene=NPM1
CYCS	Cytochrome C, Somatic	Protein Coding	P99999	52	GC07M025118	5.55900764	https://www.genecards.org/cgi-bin/carddisp.pl?gene=CYCS
FGD1	FYVE, RhoGEF And PH Domain Containing 1	Protein Coding	P98174	44	GC0XM054488	5.5571866	https://www.genecards.org/cgi-bin/carddisp.pl?gene=FGD1
MTHFS	Methenyltetrahydrofolate Synthetase	Protein Coding	P49914	46	GC15M079833	5.5546627	https://www.genecards.org/cgi-bin/carddisp.pl?gene=MTHFS
MAPK9	Mitogen-Activated Protein Kinase 9	Protein Coding	P45984	52	GC05M180288	5.54451227	https://www.genecards.org/cgi-bin/carddisp.pl?gene=MAPK9
LOX	Lysyl Oxidase	Protein Coding	P28300	51	GC05M122063	5.54211712	https://www.genecards.org/cgi-bin/carddisp.pl?gene=LOX
DMD	Dystrophin	Protein Coding	P11532	49	GC0XM031097	5.53881788	https://www.genecards.org/cgi-bin/carddisp.pl?gene=DMD
BIRC2	Baculoviral IAP Repeat Containing 2	Protein Coding	Q13490	51	GC11P102347	5.53688717	https://www.genecards.org/cgi-bin/carddisp.pl?gene=BIRC2

PTK6	Protein Tyrosine Kinase 6	Protein Coding	Q13882	50	GC20M063528	5.53294563	https://www.genecards.org/cgi-bin/carddisp.pl?gene=PTK6
YWHAB	Tyrosine 3-Monooxygenase/Tryptophan 5-Monooxygenase Activation Protein Beta	Protein Coding	P31946	51	GC20P044885	5.53233719	https://www.genecards.org/cgi-bin/carddisp.pl?gene=YWHAB
CCL11	C-C Motif Chemokine Ligand 11	Protein Coding	P51671	48	GC17P034285	5.52414799	https://www.genecards.org/cgi-bin/carddisp.pl?gene=CCL11
CORO1B	Coronin 1B	Protein Coding	Q9BR76	40	GC11M067435	5.51913452	https://www.genecards.org/cgi-bin/carddisp.pl?gene=CORO1B
KMT2B	Lysine Methyltransferase 2B	Protein Coding	Q9UMN6	43	GC19P095860	5.51907825	https://www.genecards.org/cgi-bin/carddisp.pl?gene=KMT2B
HMGB1	High Mobility Group Box 1	Protein Coding	P09429	52	GC13M030456	5.51845169	https://www.genecards.org/cgi-bin/carddisp.pl?gene=HMGB1
EFS	Embryonal Fyn-Associated Substrate	Protein Coding	O43281	40	GC14M023356	5.51523447	https://www.genecards.org/cgi-bin/carddisp.pl?gene=EFS
TH	Tyrosine Hydroxylase	Protein Coding	P07101	54	GC11M002163	5.5127449	https://www.genecards.org/cgi-bin/carddisp.pl?gene=TH
TIMP3	TIMP Metallopeptidase Inhibitor 3	Protein Coding	P35625	47	GC22P060747	5.51194382	https://www.genecards.org/cgi-bin/carddisp.pl?gene=TIMP3
PRKACA	Protein Kinase CAMP-Activated Catalytic Subunit Alpha	Protein Coding	P17612	55	GC19M017498	5.51163483	https://www.genecards.org/cgi-bin/carddisp.pl?gene=PRKACA
CDSN	Corneodesmosin	Protein Coding	Q15517	45	GC06M031115	5.51073742	https://www.genecards.org/cgi-bin/carddisp.pl?gene=CDSN
VEGFB	Vascular Endothelial Growth Factor B	Protein Coding	P49765	47	GC11P064234	5.50823641	https://www.genecards.org/cgi-bin/carddisp.pl?gene=VEGFB
CD96	CD96 Molecule	Protein Coding	P40200	47	GC03P111292	5.50445414	https://www.genecards.org/cgi-bin/carddisp.pl?gene=CD96
ACTG2	Actin Gamma 2, Smooth Muscle	Protein Coding	P63267	47	GC02P073892	5.50029278	https://www.genecards.org/cgi-bin/carddisp.pl?gene=ACTG2
SMAD3	SMAD Family Member 3	Protein Coding	P84022	56	GC15P067063	5.49954367	https://www.genecards.org/cgi-bin/carddisp.pl?gene=SMAD3
WNT3A	Wnt Family Member 3A	Protein Coding	P56704	51	GC01P230596	5.49596214	https://www.genecards.org/cgi-bin/carddisp.pl?gene=WNT3A
MDK	Midkine	Protein Coding	P21741	47	GC11P046380	5.49594641	https://www.genecards.org/cgi-bin/carddisp.pl?gene=MDK
PLCG2	Phospholipase C Gamma 2	Protein Coding	P16885	55	GC16P081773	5.49444389	https://www.genecards.org/cgi-bin/carddisp.pl?gene=PLCG2
MIR30A	MicroRNA 30a	RNA Gene		20	GC06M071403	5.49411678	https://www.genecards.org/cgi-bin/carddisp.pl?gene=MIR30A
AXL	AXL Receptor Tyrosine Kinase	Protein Coding	P30530	55	GC19P041219	5.49396563	https://www.genecards.org/cgi-bin/carddisp.pl?gene=AXL
PPP2R5D	Protein Phosphatase 2 Regulatory Subunit B'Delta	Protein Coding	Q14738	50	GC06P119122	5.49199677	https://www.genecards.org/cgi-bin/carddisp.pl?gene=PPP2R5D
NR3C1	Nuclear Receptor Subfamily 3 Group C Member 1	Protein Coding	P04150	53	GC05M143277	5.4824667	https://www.genecards.org/cgi-bin/carddisp.pl?gene=NR3C1
IFNA1	Interferon Alpha 1	Protein Coding	P01562	42	GC09P021678	5.47562027	https://www.genecards.org/cgi-bin/carddisp.pl?gene=IFNA1
EPHB3	EPH Receptor B3	Protein Coding	P54753	50	GC03P184561	5.46053648	https://www.genecards.org/cgi-bin/carddisp.pl?gene=EPHB3
NOTCH3	Notch Receptor 3	Protein Coding	Q9UM47	54	GC19M015159	5.45979881	https://www.genecards.org/cgi-bin/carddisp.pl?gene=NOTCH3

SCARF2	Scavenger Receptor Class F Member 2	Protein Coding	Q96GP6	43	GC22M020424	5.45757103	https://www.genecards.org/cgi-bin/carddisp.pl?gene=SCARF2
LINC02605	Long Intergenic Non-Protein Coding RNA 2605	RNA Gene		13	GC08P078838	5.44802475	https://www.genecards.org/cgi-bin/carddisp.pl?gene=LINC02605
AR	Androgen Receptor	Protein Coding	P10275	55	GC0XP067544	5.4459734	https://www.genecards.org/cgi-bin/carddisp.pl?gene=AR
FOXP2	Forkhead Box P2	Protein Coding	O15409	47	GC07P114086	5.44474602	https://www.genecards.org/cgi-bin/carddisp.pl?gene=FOXP2
RAPGEF3	Rap Guanine Nucleotide Exchange Factor 3	Protein Coding	O95398	48	GC12M047736	5.43908501	https://www.genecards.org/cgi-bin/carddisp.pl?gene=RAPGEF3
IRS2	Insulin Receptor Substrate 2	Protein Coding	Q9Y4H2	48	GC13M109752	5.43901587	https://www.genecards.org/cgi-bin/carddisp.pl?gene=IRS2
IVL	Involucrin	Protein Coding	P07476	42	GC01P158682	5.43293381	https://www.genecards.org/cgi-bin/carddisp.pl?gene=IVL
EPHB1	EPH Receptor B1	Protein Coding	P54762	51	GC03P134795	5.43040895	https://www.genecards.org/cgi-bin/carddisp.pl?gene=EPHB1
DNM1L	Dynamin 1 Like	Protein Coding	O00429	52	GC12P032679	5.43021345	https://www.genecards.org/cgi-bin/carddisp.pl?gene=DNM1L
LRFN1	Leucine Rich Repeat And Fibronectin Type III Domain Containing 1	Protein Coding	Q9P244	41	GC19M085520	5.42801189	https://www.genecards.org/cgi-bin/carddisp.pl?gene=LRFN1
CHD2	Chromodomain Helicase DNA Binding Protein 2	Protein Coding	O14647	47	GC15P143532	5.42653227	https://www.genecards.org/cgi-bin/carddisp.pl?gene=CHD2
CALB2	Calbindin 2	Protein Coding	P22676	43	GC16P071358	5.42604589	https://www.genecards.org/cgi-bin/carddisp.pl?gene=CALB2
FUT3	Fucosyltransferase 3 (Lewis Blood Group)	Protein Coding	P21217	44	GC19M016767	5.42501879	https://www.genecards.org/cgi-bin/carddisp.pl?gene=FUT3
UNC93B1	Unc-93 Homolog B1, TLR Signaling Regulator	Protein Coding	Q9H1C4	42	GC11M067991	5.41825962	https://www.genecards.org/cgi-bin/carddisp.pl?gene=UNC93B1
CNN2	Calponin 2	Protein Coding	Q99439	43	GC19P001026	5.40308094	https://www.genecards.org/cgi-bin/carddisp.pl?gene=CNN2
NPHP4	Nephrocystin 4	Protein Coding	O75161	44	GC01M011746	5.3990221	https://www.genecards.org/cgi-bin/carddisp.pl?gene=NPHP4
RTN4	Reticulon 4	Protein Coding	Q9NQC3	48	GC02M054934	5.3887887	https://www.genecards.org/cgi-bin/carddisp.pl?gene=RTN4
CLDN5	Claudin 5	Protein Coding	O00501	44	GC22M019523	5.3850131	https://www.genecards.org/cgi-bin/carddisp.pl?gene=CLDN5
GLB1	Galactosidase Beta 1	Protein Coding	P16278	52	GC03M032963	5.38030767	https://www.genecards.org/cgi-bin/carddisp.pl?gene=GLB1
RHBDF2	Rhomboid 5 Homolog 2	Protein Coding	Q6PJF5	46	GC17M076470	5.37884617	https://www.genecards.org/cgi-bin/carddisp.pl?gene=RHBDF2
CUBN	Cubilin	Protein Coding	O60494	49	GC10M016824	5.37872791	https://www.genecards.org/cgi-bin/carddisp.pl?gene=CUBN
PLK1	Polo Like Kinase 1	Protein Coding	P53350	54	GC16P060461	5.37736845	https://www.genecards.org/cgi-bin/carddisp.pl?gene=PLK1
CLDN2	Claudin 2	Protein Coding	P57739	45	GC0XP106900	5.37578392	https://www.genecards.org/cgi-bin/carddisp.pl?gene=CLDN2
MAP1B	Microtubule Associated Protein 1B	Protein Coding	P46821	48	GC05P072107	5.37365532	https://www.genecards.org/cgi-bin/carddisp.pl?gene=MAP1B
ADAM8	ADAM Metallopeptidase Domain 8	Protein Coding	P78325	47	GC10M133262	5.3609705	https://www.genecards.org/cgi-bin/carddisp.pl?gene=ADAM8

NPC1	NPC Intracellular Cholesterol Transporter 1	Protein Coding	O15118	53	GC18M023506	5.36016655	https://www.genecards.org/cgi-bin/carddisp.pl?gene=NPC1
SNAI2	Snail Family Transcriptional Repressor 2	Protein Coding	O43623	47	GC08M048917	5.35854769	https://www.genecards.org/cgi-bin/carddisp.pl?gene=SNAI2
CXCL10	C-X-C Motif Chemokine Ligand 10	Protein Coding	P02778	48	GC04M076021	5.35842896	https://www.genecards.org/cgi-bin/carddisp.pl?gene=CXCL10
CCL21	C-C Motif Chemokine Ligand 21	Protein Coding	O00585	45	GC09M034709	5.35658741	https://www.genecards.org/cgi-bin/carddisp.pl?gene=CCL21
S100A9	S100 Calcium Binding Protein A9	Protein Coding	P06702	47	GC01P153357	5.35232687	https://www.genecards.org/cgi-bin/carddisp.pl?gene=S100A9
SMAD5-AS1	SMAD5 Antisense RNA 1	RNA Gene	Q9Y6J3	22	GC05M136129	5.35144186	https://www.genecards.org/cgi-bin/carddisp.pl?gene=SMAD5-AS1
EPHA8	EPH Receptor A8	Protein Coding	P29322	48	GC01P022563	5.35007572	https://www.genecards.org/cgi-bin/carddisp.pl?gene=EPHA8
DAB1	DAB Adaptor Protein 1	Protein Coding	O75553	47	GC01M056994	5.3490119	https://www.genecards.org/cgi-bin/carddisp.pl?gene=DAB1
MYD88	MYD88 Innate Immune Signal Transduction Adaptor	Protein Coding	Q99836	52	GC03P038233	5.34898615	https://www.genecards.org/cgi-bin/carddisp.pl?gene=MYD88
EGFL7	EGF Like Domain Multiple 7	Protein Coding	Q9UHF1	44	GC09P136658	5.34486723	https://www.genecards.org/cgi-bin/carddisp.pl?gene=EGFL7
IGF2	Insulin Like Growth Factor 2	Protein Coding	P01344	52	GC11M009655	5.32730389	https://www.genecards.org/cgi-bin/carddisp.pl?gene=IGF2
TTR	Transthyretin	Protein Coding	P02766	52	GC18P031557	5.32695103	https://www.genecards.org/cgi-bin/carddisp.pl?gene=TTR
GNRH1	Gonadotropin Releasing Hormone 1	Protein Coding	P01148	44	GC08M025419	5.32036495	https://www.genecards.org/cgi-bin/carddisp.pl?gene=GNRH1
KDM6A	Lysine Demethylase 6A	Protein Coding	O15550	51	GC0XP044873	5.30239773	https://www.genecards.org/cgi-bin/carddisp.pl?gene=KDM6A
KISS1	KiSS-1 Metastasis Suppressor	Protein Coding	Q15726	44	GC01M204190	5.30059767	https://www.genecards.org/cgi-bin/carddisp.pl?gene=KISS1
SLC26A5-AS1	SLC26A5 Antisense RNA 1	RNA Gene		15	GC07P104426	5.29239178	https://www.genecards.org/cgi-bin/carddisp.pl?gene=SLC26A5-AS1
BVES	Blood Vessel Epicardial Substance	Protein Coding	Q8NE79	45	GC06M105096	5.28327703	https://www.genecards.org/cgi-bin/carddisp.pl?gene=BVES
RIPK4	Receptor Interacting Serine/Threonine Kinase 4	Protein Coding	P57078	47	GC21M041739	5.27938318	https://www.genecards.org/cgi-bin/carddisp.pl?gene=RIPK4
PTPRK	Protein Tyrosine Phosphatase Receptor Type K	Protein Coding	Q15262	47	GC06M127949	5.27852678	https://www.genecards.org/cgi-bin/carddisp.pl?gene=PTPRK
CHRM1	Cholinergic Receptor Muscarinic 1	Protein Coding	P11229	48	GC11M116754	5.27531528	https://www.genecards.org/cgi-bin/carddisp.pl?gene=CHRM1
CCL3	C-C Motif Chemokine Ligand 3	Protein Coding	P10147	44	GC17M036088	5.27285671	https://www.genecards.org/cgi-bin/carddisp.pl?gene=CCL3
SDHD	Succinate Dehydrogenase Complex Subunit D	Protein Coding	O14521	47	GC11P112316	5.27160501	https://www.genecards.org/cgi-bin/carddisp.pl?gene=SDHD
TRA-TGC7-1	TRNA-Ala (Anticodon TGC) 7-1	RNA Gene		10	GC06M087020	5.27155399	https://www.genecards.org/cgi-bin/carddisp.pl?gene=TRA-TGC7-1
NOS1AP	Nitric Oxide Synthase 1 Adaptor Protein	Protein Coding	O75052	43	GC01P162069	5.27069759	https://www.genecards.org/cgi-bin/carddisp.pl?gene=NOS1AP
UFSP2	UFM1 Specific Peptidase 2	Protein Coding	Q9NUQ7	43	GC04M185399	5.26985788	https://www.genecards.org/cgi-bin/carddisp.pl?gene=UFSP2

TNFRSF11B	TNF Receptor Superfamily Member 11b	Protein Coding	O00300	51	GC08M118923	5.26500416	https://www.genecards.org/cgi-bin/carddisp.pl?gene=TNFRSF11B
KCNT2	Potassium Sodium-Activated Channel Subfamily T Member 2	Protein Coding	Q6UVM3	42	GC01M196225	5.26446581	https://www.genecards.org/cgi-bin/carddisp.pl?gene=KCNT2
RAVER1	Ribonucleoprotein, PTB Binding 1	Protein Coding	Q8IY67	38	GC19M010316	5.26275826	https://www.genecards.org/cgi-bin/carddisp.pl?gene=RAVER1
CDC25C	Cell Division Cycle 25C	Protein Coding	P30307	51	GC05M138285	5.26217127	https://www.genecards.org/cgi-bin/carddisp.pl?gene=CDC25C
F2RL1	F2R Like Trypsin Receptor 1	Protein Coding	P55085	47	GC05P076818	5.25609589	https://www.genecards.org/cgi-bin/carddisp.pl?gene=F2RL1
NLRP3	NLR Family Pyrin Domain Containing 3	Protein Coding	Q96P20	52	GC01P247416	5.24989414	https://www.genecards.org/cgi-bin/carddisp.pl?gene=NLRP3
TUBA1B	Tubulin Alpha 1b	Protein Coding	P68363	47	GC12M049127	5.24650621	https://www.genecards.org/cgi-bin/carddisp.pl?gene=TUBA1B
TNK2	Tyrosine Kinase Non Receptor 2	Protein Coding	Q07912	50	GC03M195863	5.24220181	https://www.genecards.org/cgi-bin/carddisp.pl?gene=TNK2
GRIK2	Glutamate Ionotropic Receptor Kainate Type Subunit 2	Protein Coding	Q13002	53	GC06P100962	5.24114656	https://www.genecards.org/cgi-bin/carddisp.pl?gene=GRIK2
DUSP19	Dual Specificity Phosphatase 19	Protein Coding	Q8WTR2	42	GC02P183078	5.22881222	https://www.genecards.org/cgi-bin/carddisp.pl?gene=DUSP19
PSAP	Prosaposin	Protein Coding	P07602	51	GC10M071816	5.22511482	https://www.genecards.org/cgi-bin/carddisp.pl?gene=PSAP
RB1	RB Transcriptional Corepressor 1	Protein Coding	P06400	52	GC13P048303	5.21758986	https://www.genecards.org/cgi-bin/carddisp.pl?gene=RB1
MAPRE1	Microtubule Associated Protein RP/EB Family Member 1	Protein Coding	Q15691	48	GC20P032819	5.21656609	https://www.genecards.org/cgi-bin/carddisp.pl?gene=MAPRE1
ERBB3	Erb-B2 Receptor Tyrosine Kinase 3	Protein Coding	P21860	57	GC12P059071	5.21492195	https://www.genecards.org/cgi-bin/carddisp.pl?gene=ERBB3
SCN2B	Sodium Voltage-Gated Channel Beta Subunit 2	Protein Coding	O60939	50	GC11M120478	5.21392632	https://www.genecards.org/cgi-bin/carddisp.pl?gene=SCN2B
RHOG	Ras Homolog Family Member G	Protein Coding	P84095	44	GC11M009723	5.2137394	https://www.genecards.org/cgi-bin/carddisp.pl?gene=RHOG
NEU1	Neuraminidase 1	Protein Coding	Q99519	50	GC06M031857	5.21045542	https://www.genecards.org/cgi-bin/carddisp.pl?gene=NEU1
SMARCE1	SWI/SNF Related, Matrix Associated, Actin Dependent Regulator Of Chromatin, Subfamily E, Member 1	Protein Coding	Q969G3	49	GC17M040624	5.20307207	https://www.genecards.org/cgi-bin/carddisp.pl?gene=SMARCE1
ENO2	Enolase 2	Protein Coding	P09104	51	GC12P006913	5.20161152	https://www.genecards.org/cgi-bin/carddisp.pl?gene=ENO2
EPO	Erythropoietin	Protein Coding	P01588	46	GC07P100720	5.19451332	https://www.genecards.org/cgi-bin/carddisp.pl?gene=EPO
ENTPD5	Ectonucleoside Triphosphate Diphosphohydrolase 5 (Inactive)	Protein Coding	O75356	48	GC14M074007	5.19417763	https://www.genecards.org/cgi-bin/carddisp.pl?gene=ENTPD5
CSRP1	Cysteine And Glycine Rich Protein 1	Protein Coding	P21291	46	GC01M201484	5.19205379	https://www.genecards.org/cgi-bin/carddisp.pl?gene=CSRP1
ELP4	Elongator Acetyltransferase Complex Subunit 4	Protein Coding	Q96EB1	43	GC11P031509	5.18366146	https://www.genecards.org/cgi-bin/carddisp.pl?gene=ELP4
CLTC	Clathrin Heavy Chain	Protein Coding	Q00610	49	GC17P059619	5.18196821	https://www.genecards.org/cgi-bin/carddisp.pl?gene=CLTC
FUT4	Fucosyltransferase 4	Protein Coding	P22083	42	GC11P094544	5.17623234	https://www.genecards.org/cgi-bin/carddisp.pl?gene=FUT4

PIK3C2A	Phosphatidylinositol-4-Phosphate 3-Kinase Catalytic Subunit Type 2 Alpha	Protein Coding	O00443	50	GC11M017858	5.17398214	https://www.genecards.org/cgi-bin/carddisp.pl?gene=PIK3C2A
LAMP2	Lysosomal Associated Membrane Protein 2	Protein Coding	P13473	48	GC0XM120426	5.16142225	https://www.genecards.org/cgi-bin/carddisp.pl?gene=LAMP2
PROCR	Protein C Receptor	Protein Coding	Q9UNN8	45	GC20P035171	5.15877962	https://www.genecards.org/cgi-bin/carddisp.pl?gene=PROCR
PIK3R3	Phosphoinositide-3-Kinase Regulatory Subunit 3	Protein Coding	Q92569	48	GC01M046041	5.15375853	https://www.genecards.org/cgi-bin/carddisp.pl?gene=PIK3R3
ADRB2	Adrenoceptor Beta 2	Protein Coding	P07550	51	GC05P149798	5.15341234	https://www.genecards.org/cgi-bin/carddisp.pl?gene=ADRB2
PRKCZ	Protein Kinase C Zeta	Protein Coding	Q05513	52	GC01P008428	5.15224648	https://www.genecards.org/cgi-bin/carddisp.pl?gene=PRKCZ
EDIL3	EGF Like Repeats And Discoidin Domains 3	Protein Coding	O43854	44	GC05M083940	5.15005636	https://www.genecards.org/cgi-bin/carddisp.pl?gene=EDIL3
STIM1	Stromal Interaction Molecule 1	Protein Coding	Q13586	53	GC11P005883	5.14958382	https://www.genecards.org/cgi-bin/carddisp.pl?gene=STIM1
SURF1	SURF1 Cytochrome C Oxidase Assembly Factor	Protein Coding	Q15526	45	GC09M133351	5.14870739	https://www.genecards.org/cgi-bin/carddisp.pl?gene=SURF1
CLEC12A	C-Type Lectin Domain Family 12 Member A	Protein Coding	Q5QGZ9	43	GC12P009951	5.14310551	https://www.genecards.org/cgi-bin/carddisp.pl?gene=CLEC12A
PPIB	Peptidylprolyl Isomerase B	Protein Coding	P23284	53	GC15M064155	5.14244652	https://www.genecards.org/cgi-bin/carddisp.pl?gene=PPIB
CCND3	Cyclin D3	Protein Coding	P30281	50	GC06M041934	5.13982201	https://www.genecards.org/cgi-bin/carddisp.pl?gene=CCND3
NDUFS7	NADH:Ubiquinone Oxidoreductase Core Subunit S7	Protein Coding	O75251	50	GC19P094787	5.13758755	https://www.genecards.org/cgi-bin/carddisp.pl?gene=NDUFS7
SLC6A3	Solute Carrier Family 6 Member 3	Protein Coding	Q01959	53	GC05M001392	5.13667202	https://www.genecards.org/cgi-bin/carddisp.pl?gene=SLC6A3
PCDHGC3	Protocadherin Gamma Subfamily C, 3	Protein Coding	Q9UN70	42	GC05P141475	5.13649893	https://www.genecards.org/cgi-bin/carddisp.pl?gene=PCDHGC3
OSBPL3	Oxysterol Binding Protein Like 3	Protein Coding	Q9H4L5	42	GC07M024836	5.13361645	https://www.genecards.org/cgi-bin/carddisp.pl?gene=OSBPL3
NDUFS4	NADH:Ubiquinone Oxidoreductase Subunit S4	Protein Coding	O43181	46	GC05P053560	5.132092	https://www.genecards.org/cgi-bin/carddisp.pl?gene=NDUFS4
EPHA4	EPH Receptor A4	Protein Coding	P54764	54	GC02M221418	5.1281929	https://www.genecards.org/cgi-bin/carddisp.pl?gene=EPHA4
MEGF10	Multiple EGF Like Domains 10	Protein Coding	Q96KG7	45	GC05P127230	5.12670946	https://www.genecards.org/cgi-bin/carddisp.pl?gene=MEGF10
PPP2CB	Protein Phosphatase 2 Catalytic Subunit Beta	Protein Coding	P62714	48	GC08M030775	5.11600256	https://www.genecards.org/cgi-bin/carddisp.pl?gene=PPP2CB
BTK	Bruton Tyrosine Kinase	Protein Coding	Q06187	57	GC0XM101349	5.11518955	https://www.genecards.org/cgi-bin/carddisp.pl?gene=BTK
SOD2-OT1	SOD2 Overlapping Transcript 1	RNA Gene		13	GC06M159772	5.11376286	https://www.genecards.org/cgi-bin/carddisp.pl?gene=SOD2-OT1
CYFIP1	Cytoplasmic FMR1 Interacting Protein 1	Protein Coding	Q7L576	44	GC15M022867	5.10390663	https://www.genecards.org/cgi-bin/carddisp.pl?gene=CYFIP1
PCDH8	Protocadherin 8	Protein Coding	O95206	43	GC13M052842	5.10049629	https://www.genecards.org/cgi-bin/carddisp.pl?gene=PCDH8
KRT9	Keratin 9	Protein Coding	P35527	45	GC17M041565	5.099473	https://www.genecards.org/cgi-bin/carddisp.pl?gene=KRT9

IL32	Interleukin 32	Protein Coding	P24001	42	GC16P059852	5.09670591	https://www.genecards.org/cgi-bin/carddisp.pl?gene=IL32
LGALS8	Galectin 8	Protein Coding	O00214	44	GC01P236518	5.09519672	https://www.genecards.org/cgi-bin/carddisp.pl?gene=LGALS8
SPON1	Spondin 1	Protein Coding	Q9HCB6	41	GC11P013964	5.0933466	https://www.genecards.org/cgi-bin/carddisp.pl?gene=SPON1
ZMPSTE24	Zinc Metallopeptidase STE24	Protein Coding	O75844	45	GC01P040258	5.08471394	https://www.genecards.org/cgi-bin/carddisp.pl?gene=ZMPSTE24
NPHP3-ACAD11	NPHP3-ACAD11 Readthrough (NMD Candidate)	RNA Gene		16	GC03M132558	5.08329153	https://www.genecards.org/cgi-bin/carddisp.pl?gene=NPHP3-ACAD11
SUMO1	Small Ubiquitin Like Modifier 1	Protein Coding	P63165	50	GC02M202206	5.08049011	https://www.genecards.org/cgi-bin/carddisp.pl?gene=SUMO1
ATP7A	ATPase Copper Transporting Alpha	Protein Coding	Q04656	50	GC0XP078103	5.07720089	https://www.genecards.org/cgi-bin/carddisp.pl?gene=ATP7A
NKX2-1	NK2 Homeobox 1	Protein Coding	P43699	51	GC14M036516	5.07647419	https://www.genecards.org/cgi-bin/carddisp.pl?gene=NKX2-1
ASAP2	ArfGAP With SH3 Domain, Ankyrin Repeat And PH Domain 2	Protein Coding	O43150	42	GC02P009206	5.07558012	https://www.genecards.org/cgi-bin/carddisp.pl?gene=ASAP2
CD59	CD59 Molecule (CD59 Blood Group)	Protein Coding	P13987	50	GC11M033709	5.07468367	https://www.genecards.org/cgi-bin/carddisp.pl?gene=CD59
PRKAR2A	Protein Kinase CAMP-Dependent Type II Regulatory Subunit Alpha	Protein Coding	P13861	49	GC03M048744	5.0724411	https://www.genecards.org/cgi-bin/carddisp.pl?gene=PRKAR2A
PDHA1	Pyruvate Dehydrogenase E1 Subunit Alpha 1	Protein Coding	P08559	51	GC0XP019343	5.06460094	https://www.genecards.org/cgi-bin/carddisp.pl?gene=PDHA1
NME1	NME/NM23 Nucleoside Diphosphate Kinase 1	Protein Coding	P15531	48	GC17P093982	5.05743694	https://www.genecards.org/cgi-bin/carddisp.pl?gene=NME1
AURKA	Aurora Kinase A	Protein Coding	O14965	55	GC20M056370	5.05653095	https://www.genecards.org/cgi-bin/carddisp.pl?gene=AURKA
SOX10	SRY-Box Transcription Factor 10	Protein Coding	P56693	49	GC22M074371	5.05607796	https://www.genecards.org/cgi-bin/carddisp.pl?gene=SOX10
CLU	Clusterin	Protein Coding	P10909	51	GC08M027596	5.05421925	https://www.genecards.org/cgi-bin/carddisp.pl?gene=CLU
ALG2	ALG2 Alpha-1,3/1,6-Mannosyltransferase	Protein Coding	Q9H553	45	GC09M099216	5.05359364	https://www.genecards.org/cgi-bin/carddisp.pl?gene=ALG2
CDK1	Cyclin Dependent Kinase 1	Protein Coding	P06493	51	GC10P060772	5.04963779	https://www.genecards.org/cgi-bin/carddisp.pl?gene=CDK1
DIXDC1	DIX Domain Containing 1	Protein Coding	Q155Q3	39	GC11P111927	5.04199886	https://www.genecards.org/cgi-bin/carddisp.pl?gene=DIXDC1
S100A8	S100 Calcium Binding Protein A8	Protein Coding	P05109	46	GC01M158702	5.0379138	https://www.genecards.org/cgi-bin/carddisp.pl?gene=S100A8
PKHD1	PKHD1 Ciliary IPT Domain Containing Fibrocystin/Polyductin	Protein Coding	P08F94	42	GC06M087621	5.03730297	https://www.genecards.org/cgi-bin/carddisp.pl?gene=PKHD1
ITGBL1	Integrin Subunit Beta Like 1	Protein Coding	O95965	40	GC13P101454	5.03715038	https://www.genecards.org/cgi-bin/carddisp.pl?gene=ITGBL1
CNN3	Calponin 3	Protein Coding	Q15417	40	GC01M094896	5.03621006	https://www.genecards.org/cgi-bin/carddisp.pl?gene=CNN3
ROBO1	Roundabout Guidance Receptor 1	Protein Coding	Q9Y6N7	48	GC03M078597	5.02759647	https://www.genecards.org/cgi-bin/carddisp.pl?gene=ROBO1
FLRT1	Fibronectin Leucine Rich Transmembrane Protein 1	Protein Coding	Q9NZU1	41	GC11P081806	5.02700901	https://www.genecards.org/cgi-bin/carddisp.pl?gene=FLRT1

PPFIA3	PTPRF Interacting Protein Alpha 3	Protein Coding	O75145	41	GC19P049119	5.02304173	https://www.genecards.org/cgi-bin/carddisp.pl?gene=PPFIA3
PKP4	Plakophilin 4	Protein Coding	Q99569	45	GC02P158456	5.01832247	https://www.genecards.org/cgi-bin/carddisp.pl?gene=PKP4
PRKRA	Protein Activator Of Interferon Induced Protein Kinase EIF2AK2	Protein Coding	O75569	46	GC02M178431	5.01826382	https://www.genecards.org/cgi-bin/carddisp.pl?gene=PRKRA
APOA1	Apolipoprotein A1	Protein Coding	P02647	54	GC11M116835	5.01451397	https://www.genecards.org/cgi-bin/carddisp.pl?gene=APOA1
COL10A1	Collagen Type X Alpha 1 Chain	Protein Coding	Q03692	45	GC06M116118	5.01109838	https://www.genecards.org/cgi-bin/carddisp.pl?gene=COL10A1
RTCB	RNA 2',3'-Cyclic Phosphate And 5'-OH Ligase	Protein Coding	Q9Y3I0	42	GC22M032387	5.00660801	https://www.genecards.org/cgi-bin/carddisp.pl?gene=RTCB
HSP90AB1	Heat Shock Protein 90 Alpha Family Class B Member 1	Protein Coding	P08238	52	GC06P044246	5.00438881	https://www.genecards.org/cgi-bin/carddisp.pl?gene=HSP90AB1
PCDHGC4	Protocadherin Gamma Subfamily C, 4	Protein Coding	Q9Y5F7	41	GC05P141484	5.00308704	https://www.genecards.org/cgi-bin/carddisp.pl?gene=PCDHGC4
GAS6	Growth Arrest Specific 6	Protein Coding	Q14393	47	GC13M113820	5.00229931	https://www.genecards.org/cgi-bin/carddisp.pl?gene=GAS6
