# Supplementary material for: IFIT3 promotes lymph node metastasis by interacting with LASP1 to activate FAK-ERK signaling in esophageal squamous cell carcinoma
Source: Cell Death Dis. 2025 Dec 18;17(1):110. doi: 10.1038/s41419-025-08327-z (PMC12847741; doi:10.1038/s41419-025-08327-z)
Supplement: Supplementary file 2 — Raw Data-Western Blot [file 41419_2025_8327_MOESM2_ESM.pdf]

Figure 2

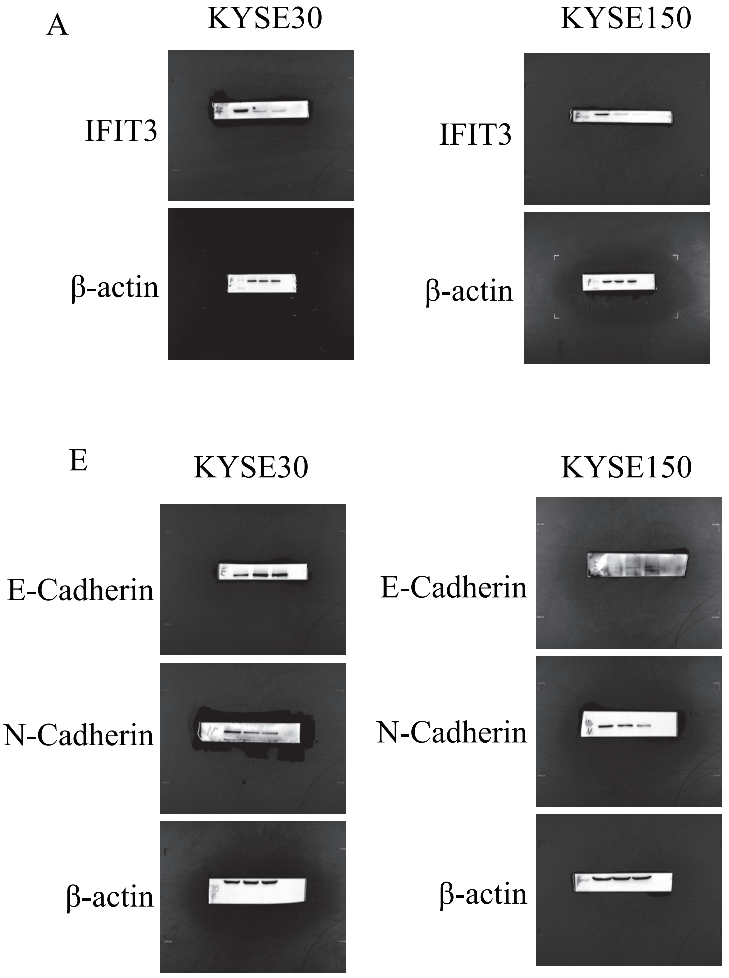
A
KYSE30
KYSE150
IFIT3
IFIT3
β-actin
β-actin
E
KYSE30
KYSE150
E-Cadherin
E-Cadherin
N-Cadherin
N-Cadherin
β-actin
β-actin

Figure 3

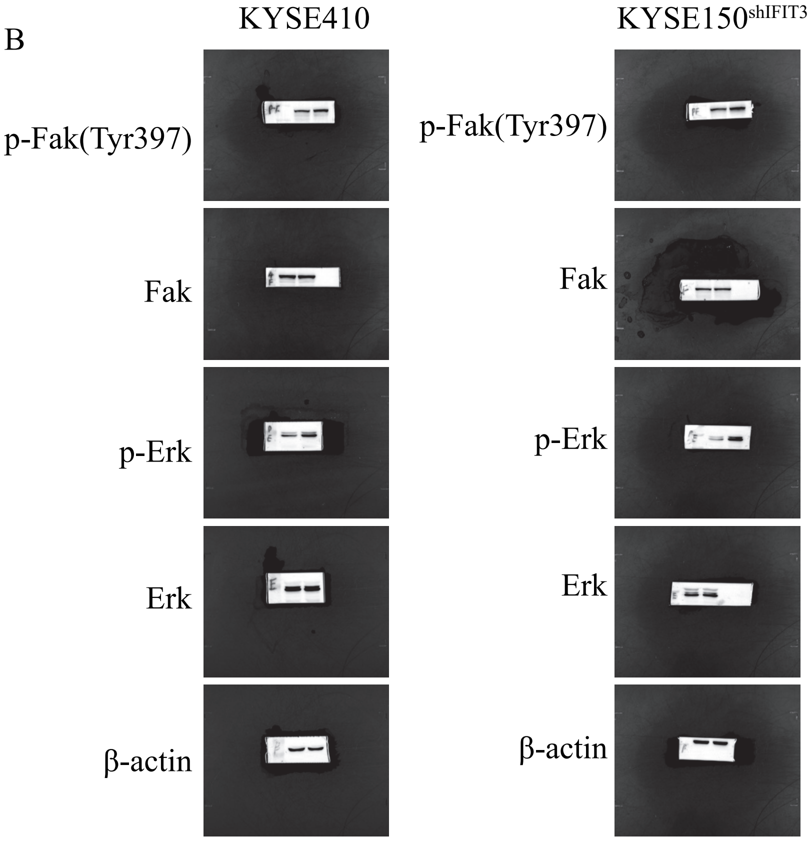
B
KYSE410
KYSE150shIFIT3
p-Fak(Tyr397)
Fak
p-Erk
Erk
β-actin
p-Fak(Tyr397)
Fak
p-Erk
Erk
β-actin

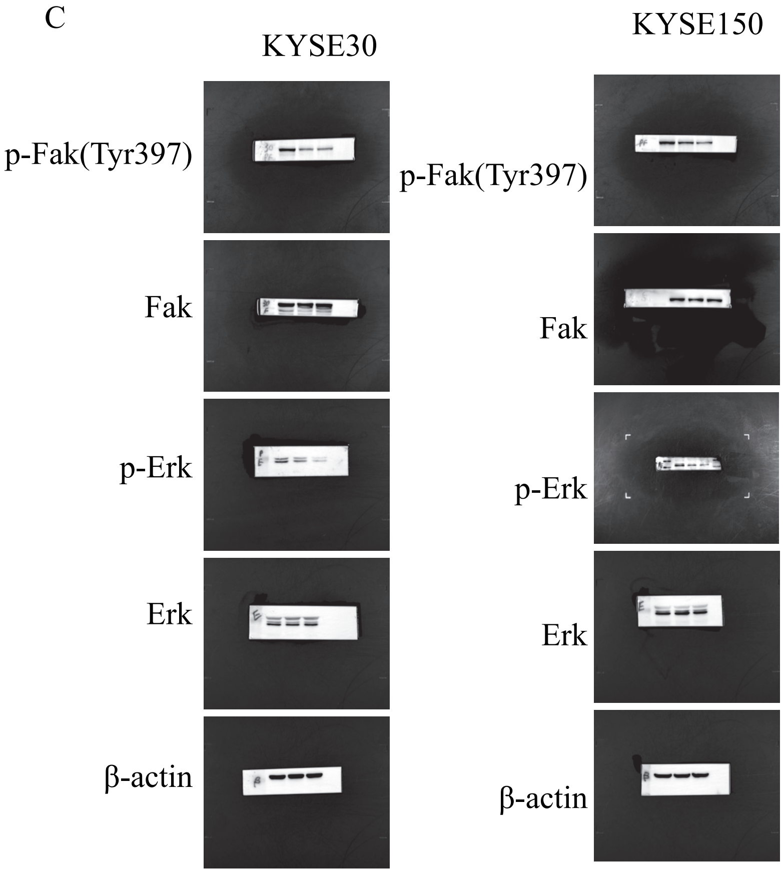
C
KYSE30
KYSE150
p-Fak(Tyr397)
Fak
p-Erk
Erk
β-actin
p-Fak(Tyr397)
Fak
p-Erk
Erk
β-actin

Figure 3

H

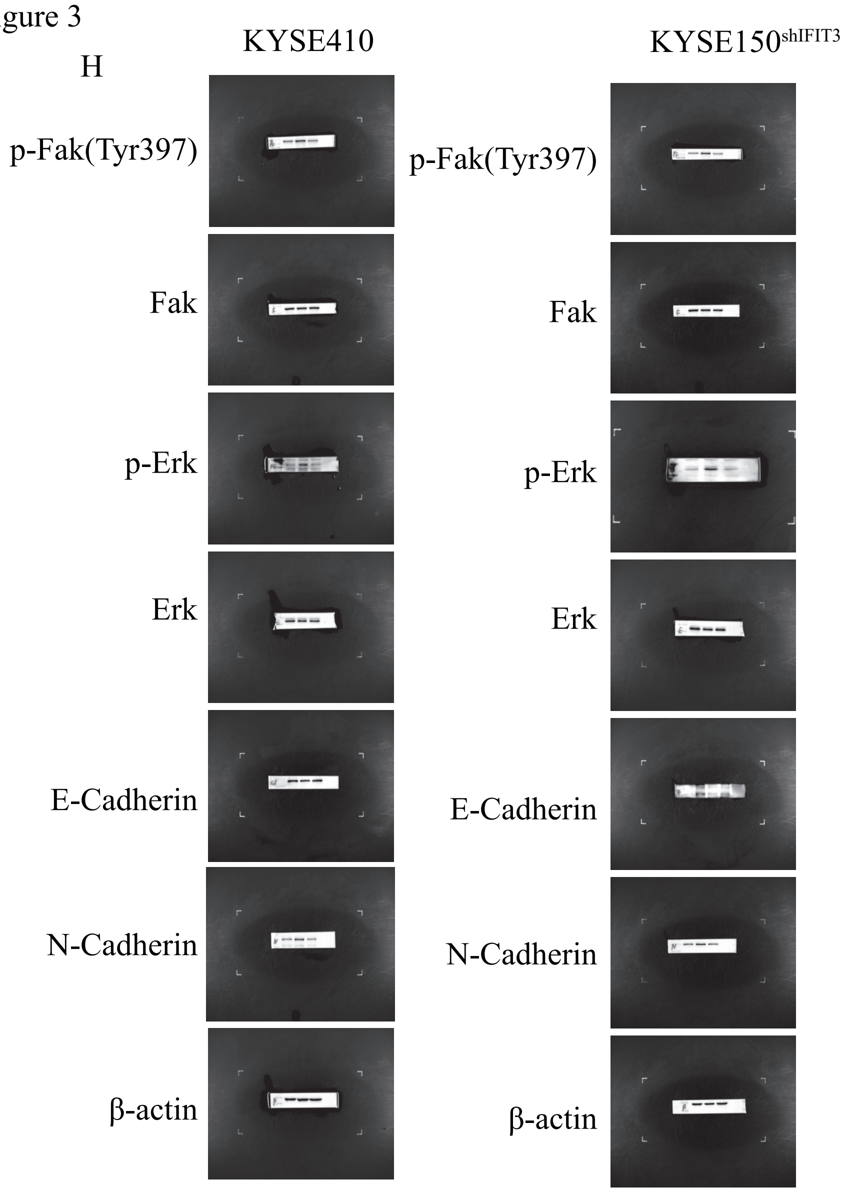
KYSE410
KYSE150shIFIT3
p-Fak(Tyr397)
Fak
p-Erk
Erk
E-Cadherin
N-Cadherin
β-actin

M

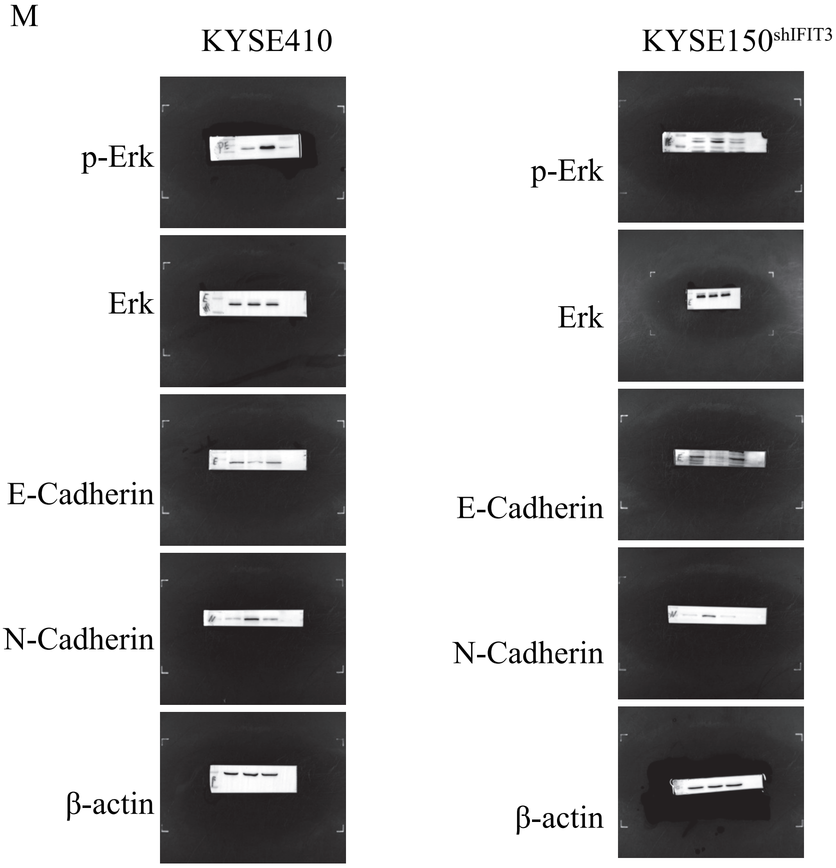
KYSE410
KYSE150shIFIT3
p-Erk
Erk
E-Cadherin
N-Cadherin
β-actin

C

IP IFIT3
IB IFIT3

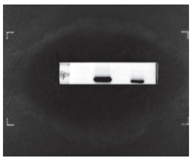

IP LASP1
IB LASP1

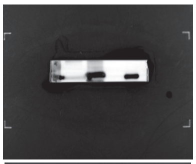

IP IFIT3
IB LASP1

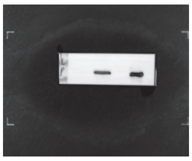

IP LASP1
IB IFIT3

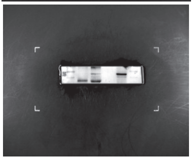

D

IP IFIT3
IB IFIT3

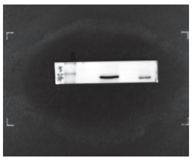

IP LASP1
IB LASP1

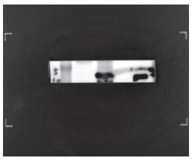

IP IFIT3
IB LASP1

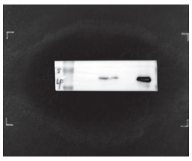

IP LASP1
IB IFIT3

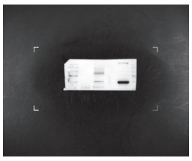

E

IP Flag
IB Flag

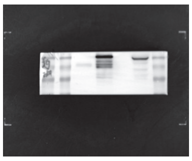

IP HA
IB HA

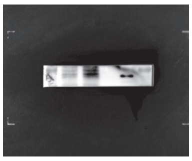

IP Flag
IB HA

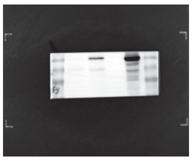

IP HA
IB Flag

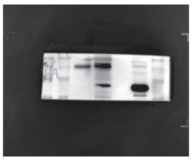

H

IP HA
IB HA

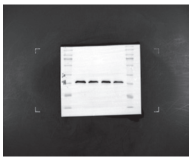

IP HA
IB Flag

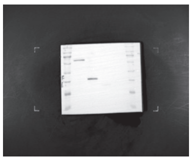

Input HA

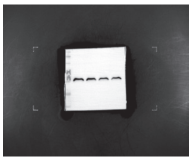

Input Flag

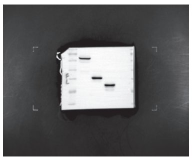

I

IP Flag
IB HA

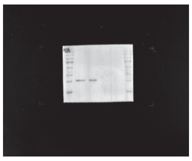

IP Flag
IB Flag

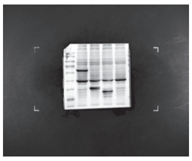

Input HA

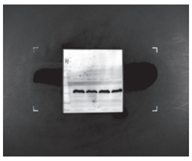

Input Flag

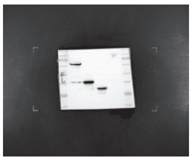

Figure 5

A

KYSE410

KYSE150

LASP1

LASP1

β-actin

β-actin

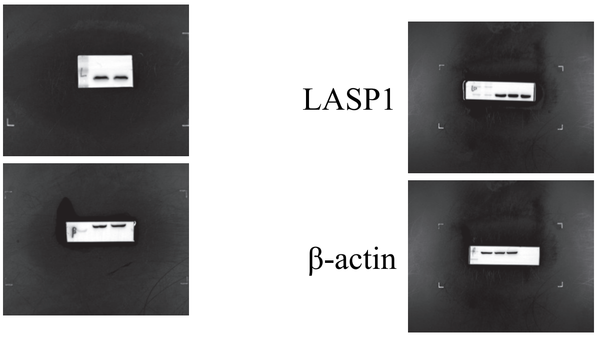

KYSE410

KYSE150

IFIT3

IFIT3

β-actin

β-actin

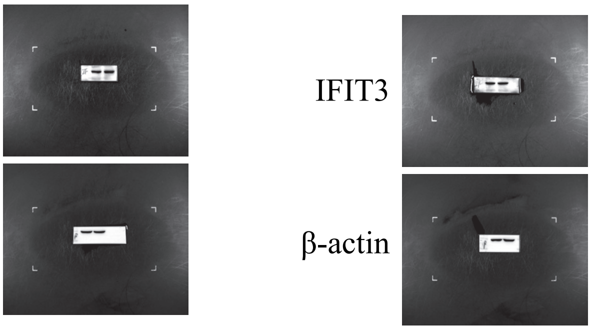

G

IP LASP1
IB Talin1

IP LASP1
IB LASP1

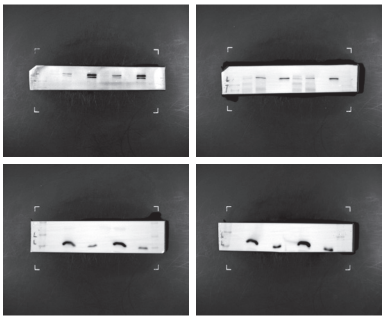

H

IP Talin1
IB Integrinβ1

IP Talin1
IB Talin1

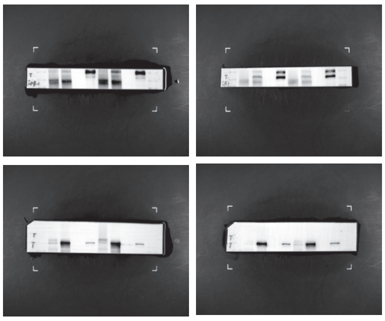

I

IP Talin1
IB Integrinβ1

IP Talin1
IB Talin1

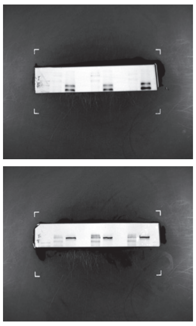

J

Input

Integrinβ1

Talin1

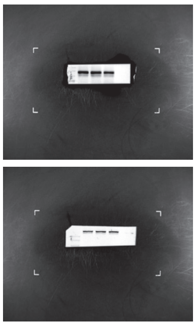

Figure 5

K

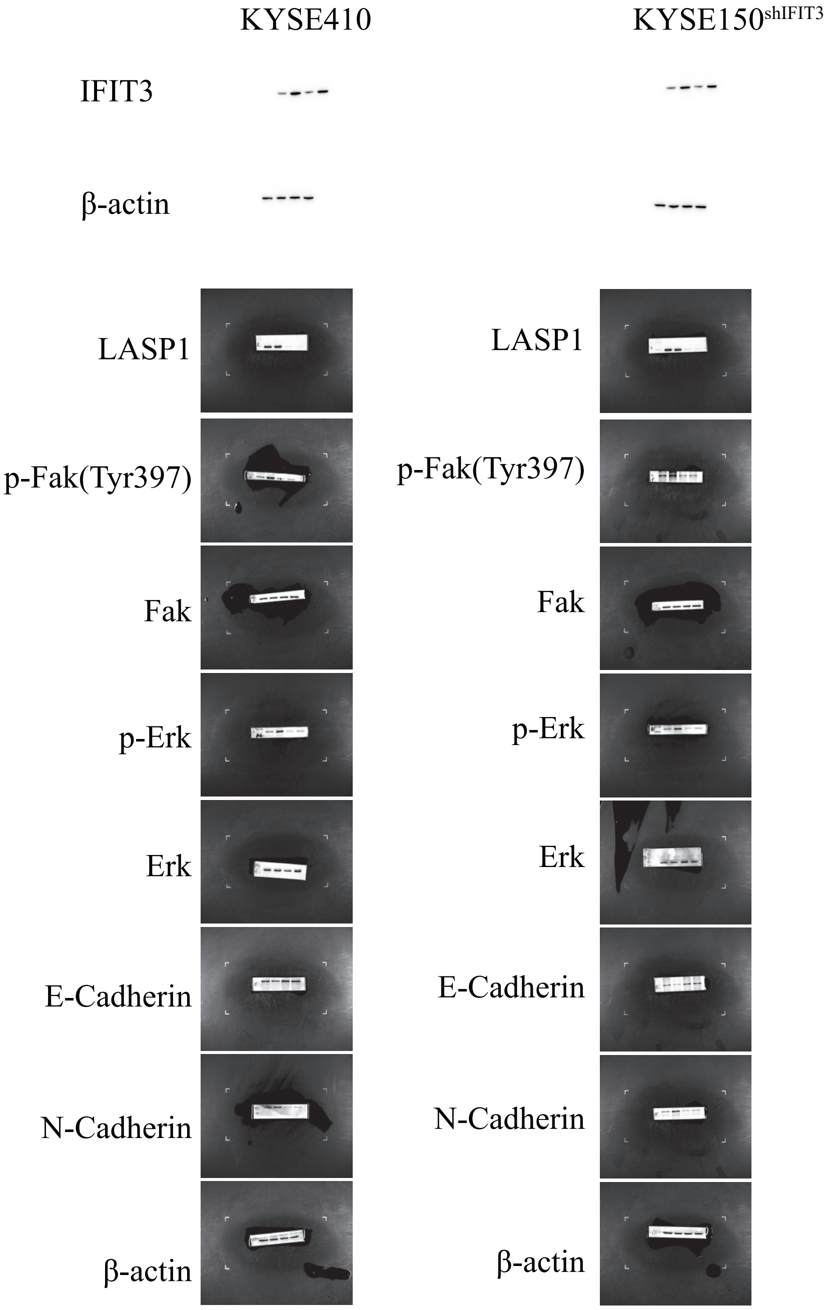
KYSE410
KYSE150shIFIT3
IFIT3
β-actin
LASP1
p-Fak(Tyr397)
Fak
p-Erk
Erk
E-Cadherin
N-Cadherin
β-actin
LASP1
p-Fak(Tyr397)
Fak
p-Erk
Erk
E-Cadherin
N-Cadherin
β-actin

Figure 5

L

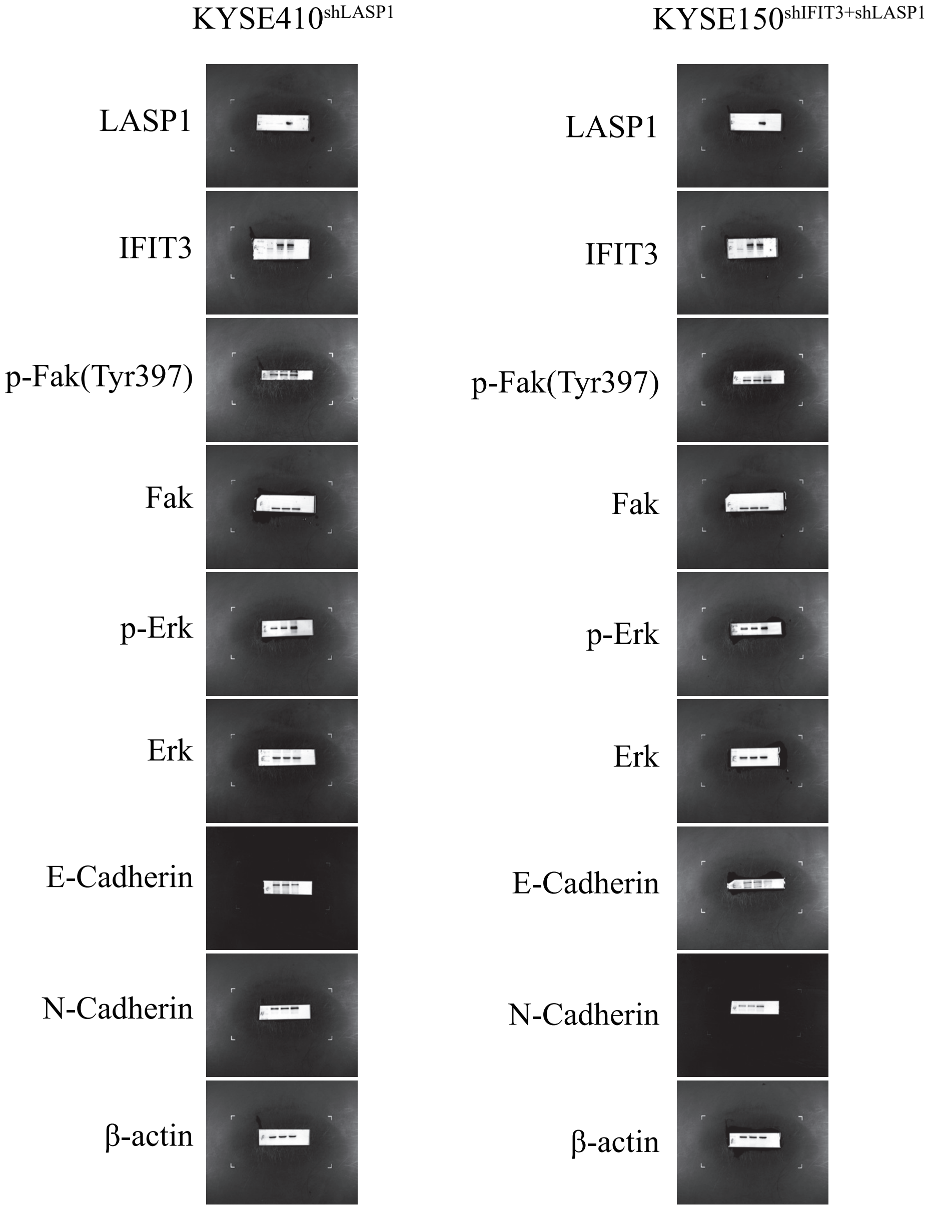
KYSE410shLASP1
KYSE150shIFIT3+shLASP1
LASP1
IFIT3
p-Fak(Tyr397)
Fak
p-Erk
Erk
E-Cadherin
N-Cadherin
β-actin
LASP1
IFIT3
p-Fak(Tyr397)
Fak
p-Erk
Erk
E-Cadherin
N-Cadherin
β-actin

Figure S2

A

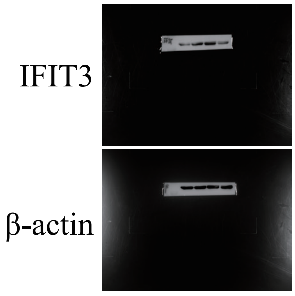

B

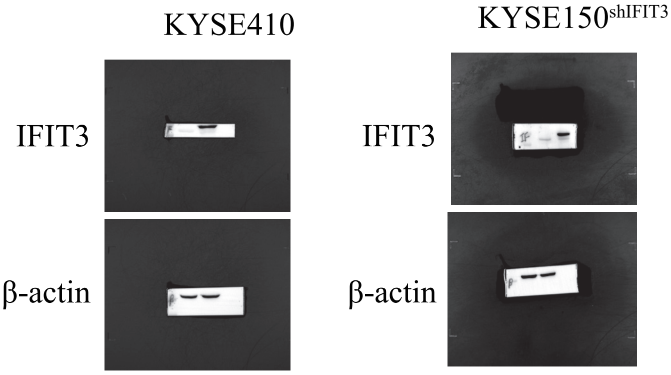

I

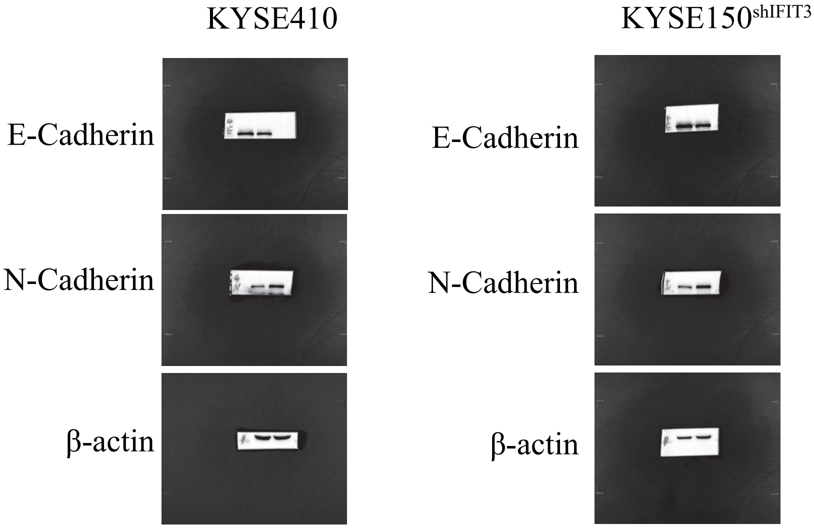

Figure S5

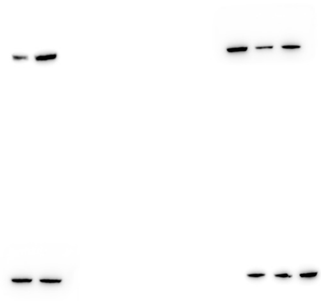

LASP1

β-actin
