# Supplementary figures and images for: IFIT3 promotes lymph node metastasis by interacting with LASP1 to activate FAK-ERK signaling in esophageal squamous cell carcinoma
Source: Cell Death Dis. 2025 Dec 18;17(1):110. doi: 10.1038/s41419-025-08327-z (PMC12847741; doi:10.1038/s41419-025-08327-z)

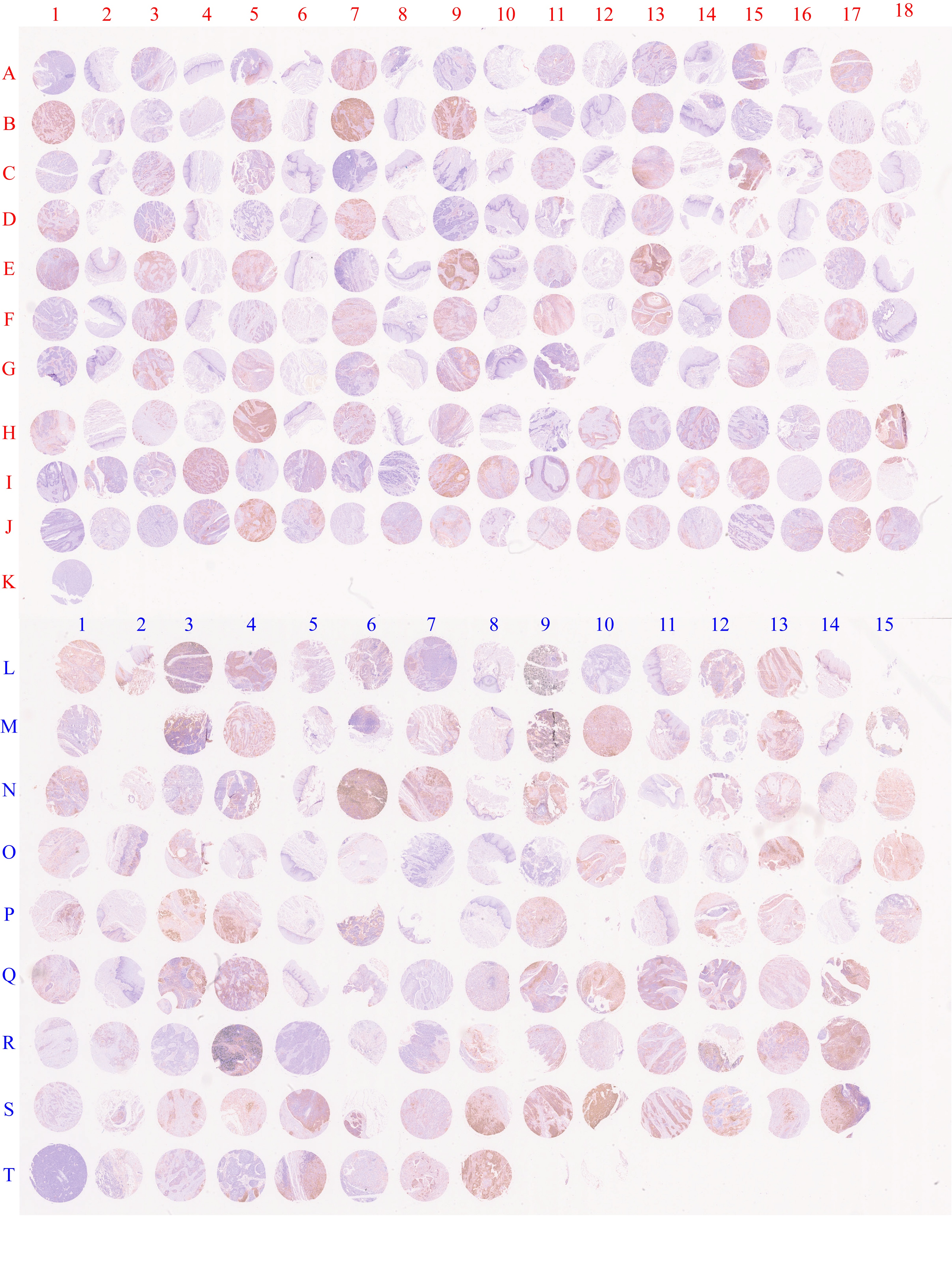

Supplement: Supplementary file 3 — Supplementary Information [file 41419_2025_8327_MOESM3_ESM.jpg]

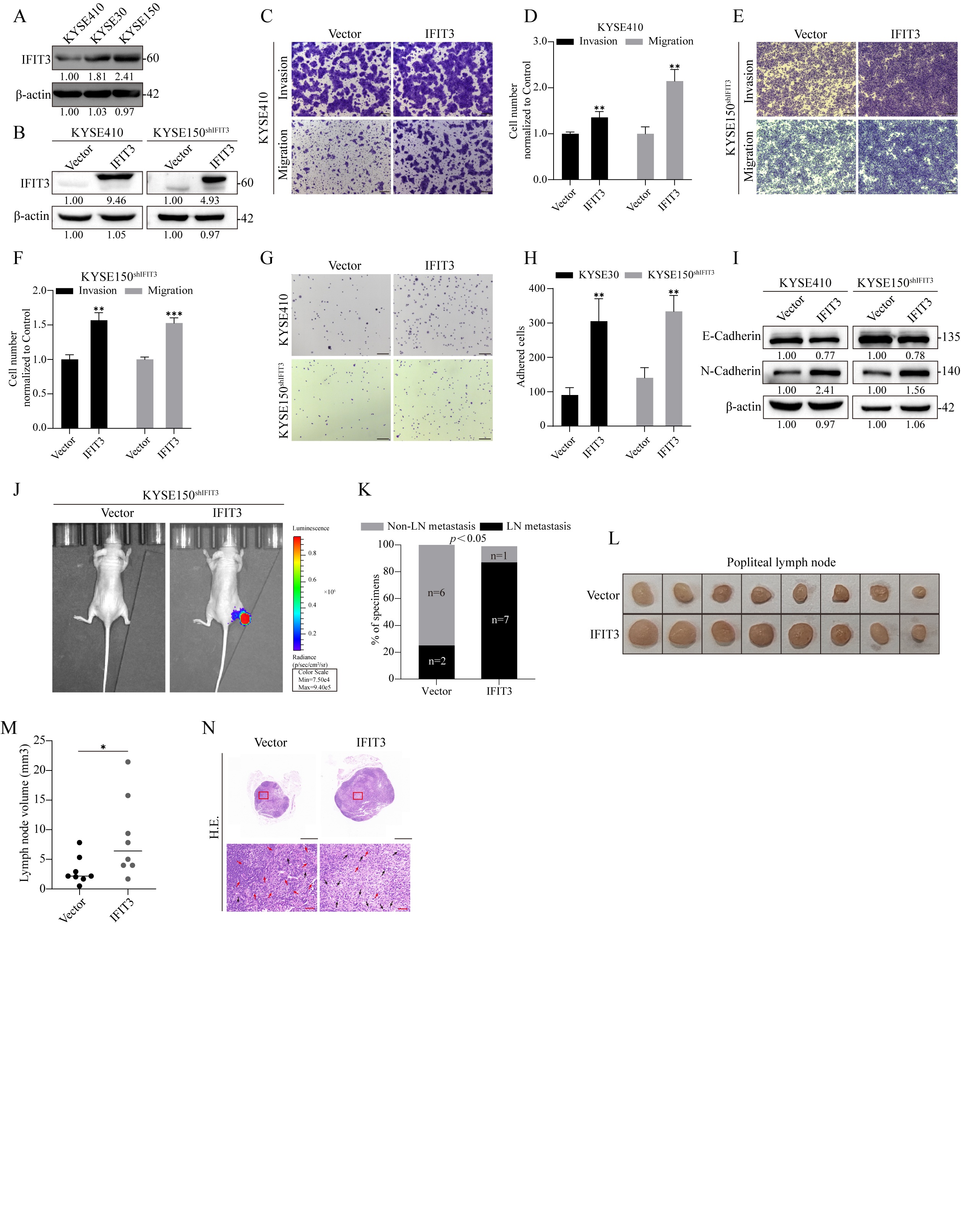

Supplement: Supplementary file 4 — Supplementary Information [file 41419_2025_8327_MOESM4_ESM.jpg]

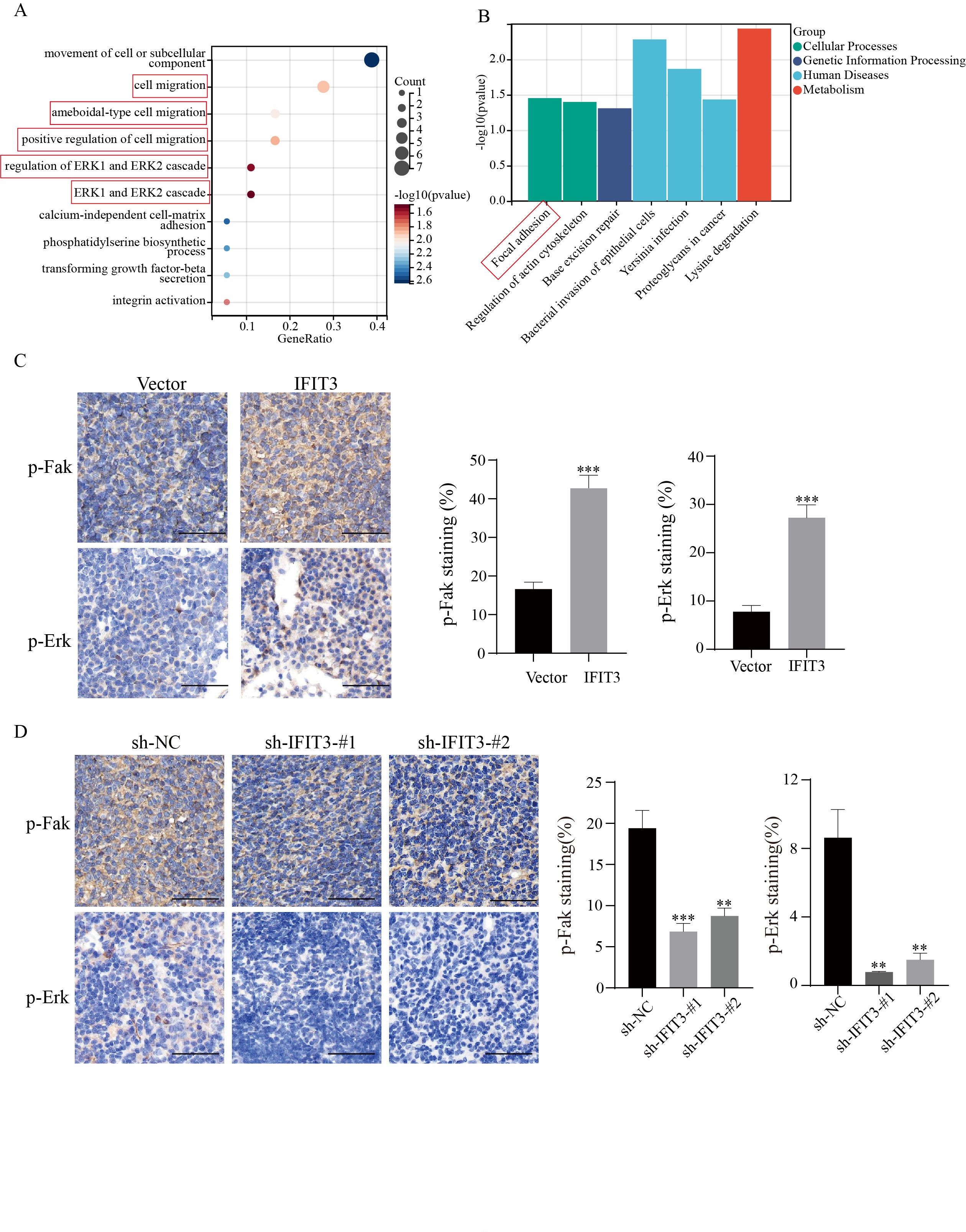

Supplement: Supplementary file 5 — Supplementary Information [file 41419_2025_8327_MOESM5_ESM.jpg]

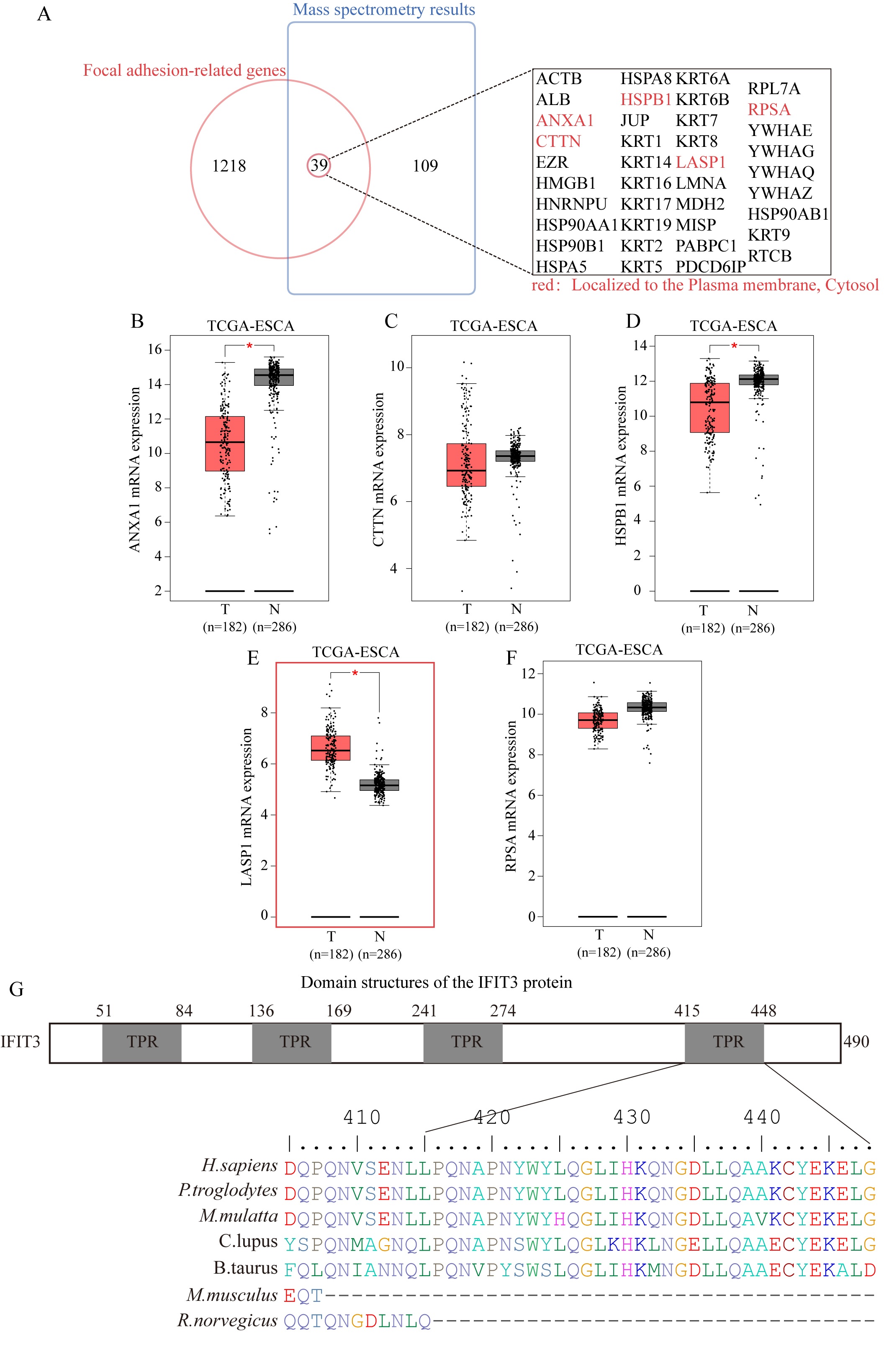

Supplement: Supplementary file 6 — Supplementary Information [file 41419_2025_8327_MOESM6_ESM.jpg]

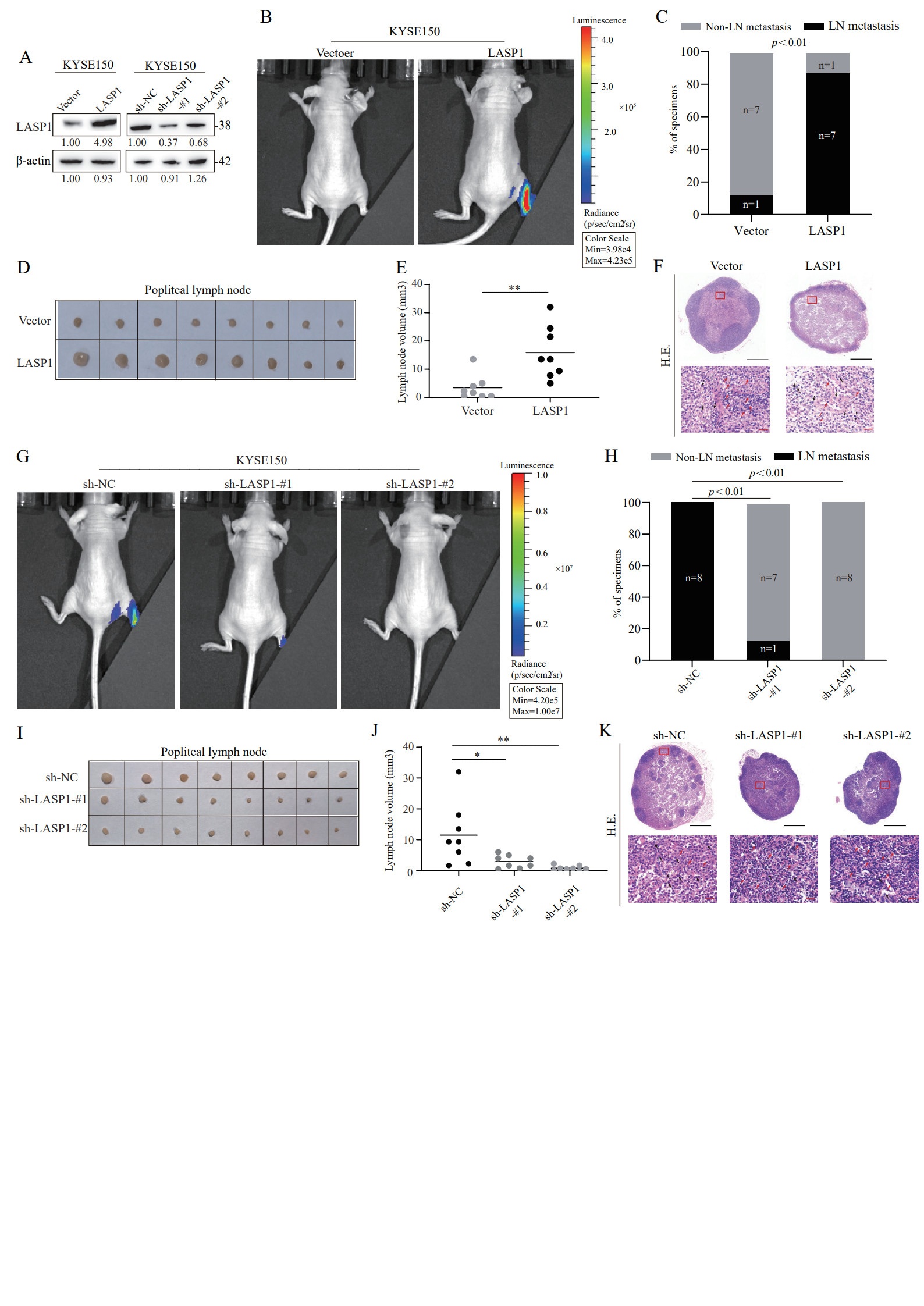

Supplement: Supplementary file 7 — Supplementary Information [file 41419_2025_8327_MOESM7_ESM.jpg]

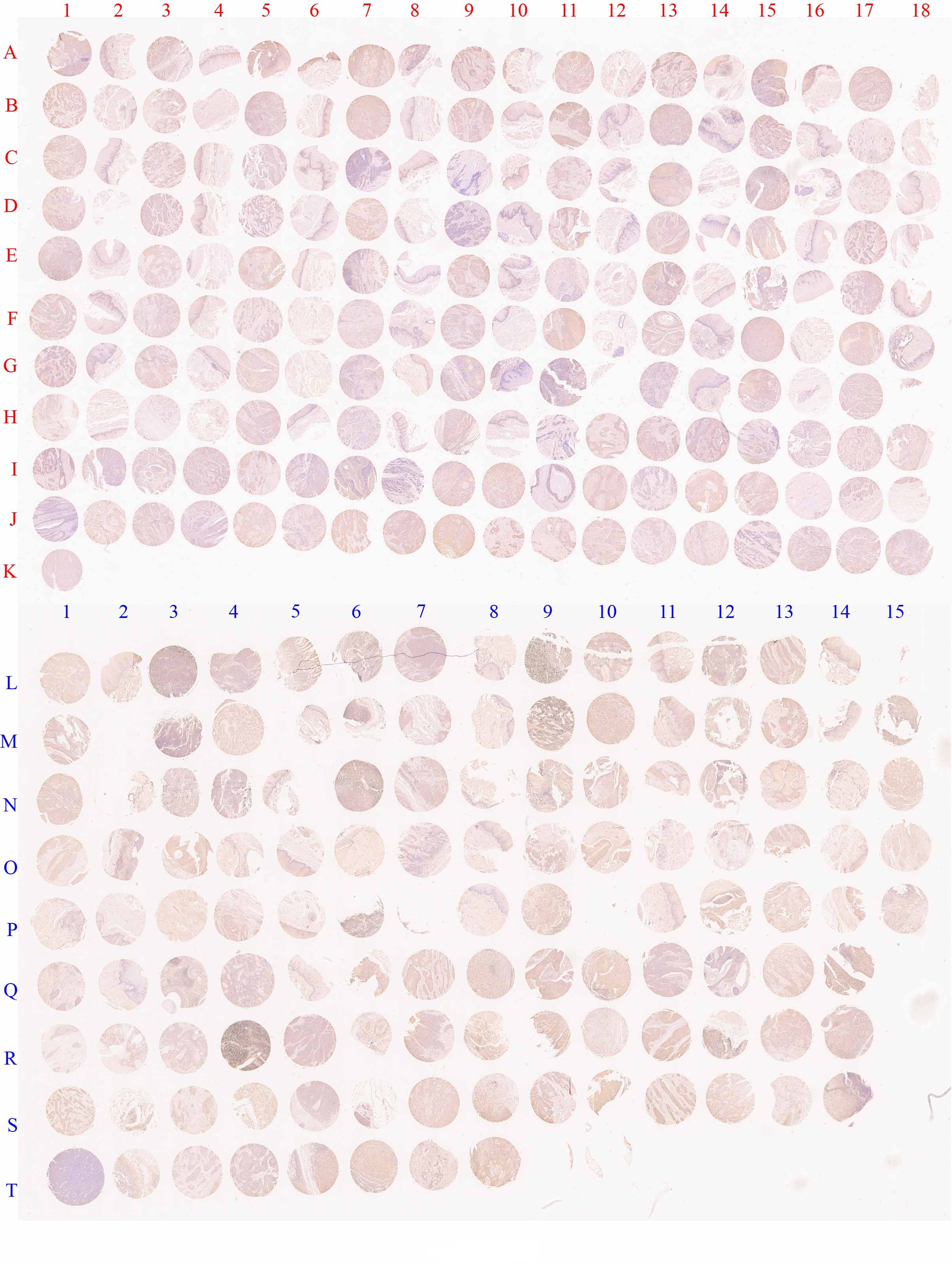

Supplement: Supplementary file 8 — Supplementary Information [file 41419_2025_8327_MOESM8_ESM.jpg]

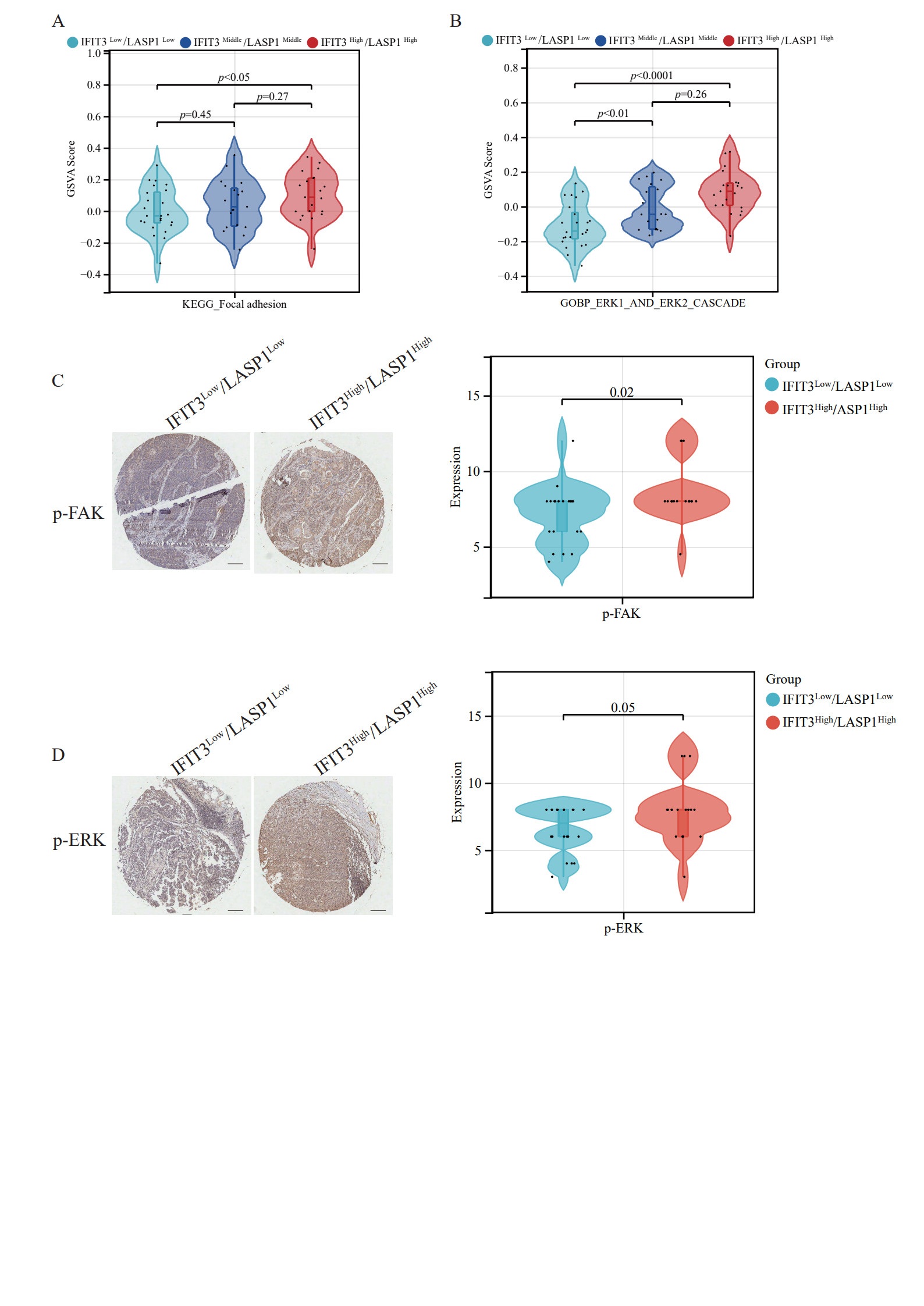

Supplement: Supplementary file 9 — Supplementary Information [file 41419_2025_8327_MOESM9_ESM.jpg]
